# Tumor endothelial marker 8 promotes cancer progression and metastasis

**DOI:** 10.18632/oncotarget.25734

**Published:** 2018-07-10

**Authors:** Anette M. Høye, Sofie D. Tolstrup, Edward R. Horton, Monica Nicolau, Helen Frost, Jung H. Woo, Jeremy P. Mauldin, Arthur E. Frankel, Thomas R. Cox, Janine T. Erler

**Affiliations:** ^1^ Biotech Research and Innovation Centre (BRIC), Faculty of Health and Medical Sciences, University of Copenhagen (UCPH), Copenhagen, Denmark; ^2^ Baylor Scott and White Health, Temple, TX, USA; ^3^ University of South Alabama Mitchell Cancer Institute, Mobile, AL, USA; ^4^ The Garvan Institute of Medical Research and The Kinghorn Cancer Centre, Cancer Division, St Vincent’s Clinical School, Faculty of Medicine, UNSW, Sydney, Australia

**Keywords:** tumor endothelial marker 8, cancer, angiogenesis, metastasis

## Abstract

Every year more than 8 million people suffer from cancer-related deaths worldwide [1]. Metastasis, the spread of cancer to distant sites, accounts for 90% of these deaths. A promising target for blocking tumor progression, without causing severe side effects [2], is Tumor Endothelial Marker 8 (TEM8), an integrin-like cell surface protein expressed predominantly in the tumor endothelium and in cancer cells [3, 4]. Here, we have investigated the role of TEM8 in cancer progression, angiogenesis and metastasis in invasive breast cancer, and validated the main findings and important results in colorectal cancer. We show that the loss of TEM8 in cancer cells results in inhibition of cancer progression, reduction in tumor angiogenesis and reduced metastatic burden in breast cancer mouse models. Furthermore, we show that TEM8 regulates cancer progression by affecting the expression levels of cell cycle-related genes. Taken together, our findings may have broad clinical and therapeutic potential for breast and colorectal primary tumor and metastasis treatment by targeting TEM8.

## INTRODUCTION

Breast cancer is the fifth leading cause of cancer death, and colorectal cancer (CRC) is the fourth leading cause of cancer death, worldwide [1]. Breast cancer is the most frequently diagnosed cancer in women, while CRC is the second and third most frequently diagnosed cancer in women and men, respectively [1]. Despite continuous advances in prevention, detection and therapy of breast cancer; 5% of patients have metastases at the time of diagnosis and 30% of patients develop metastases during treatment [5]. In CRC, between 15–25% of CRC patients present with metastasis at the time of diagnosis [6]. Metastatic cancer is nearly incurable, and the use of end-stage chemotherapy, which is often the standard of care, may be even worse for patients compared to no treatment [7]. Therefore, there is a chronic need for new treatments to prevent and to cure metastatic cancer. Targeting specific components of the metastatic cascade might offer new hopes for therapeutic strategies [8].

Tumor Endothelial Marker 8 (TEM8), also known as Anthrax Receptor 1 (ANTXR1), is highly up-regulated in the tumor endothelium and is expressed in many cancer types, including breast cancer and CRC [4]. The physiological function of TEM8 is not yet fully understood, although it has been found to be a functional anthrax toxin receptor [9]. Initial studies of TEM8 knock out (KO) mice found TEM8 to be dispensable in a normal physiological state [10]. However, in a cancer setting TEM8 has been shown to be required for optimal tumor growth and angiogenesis (the formation of new blood vessels) [2]. TEM8 has been shown to bind collagen I and the C5 domain of collagen α3(VI), which are both pro-angiogenic extracellular matrix (ECM) components [11, 12]. The role of TEM8 in angiogenesis was further elucidated when it was shown that TEM8 interacts with vascular endothelial growth factor receptor 2 (VEGFR2) and thus modulates downstream VEGF signaling [13, 14]. Primary tumor growth and metastasis are highly dependent on angiogenesis, since tumors can only grow to a size of a few millimeters without forming new blood vessels to supply the expanding tumor with oxygen and nutrients [15]. Tumor angiogenesis is hypothesized to be an important factor for the escape and spread of metastatic cells due to the formation of leaky blood vessels, therefore, treatments that target TEM8 could potentially distinguish between physiological and pathological angiogenesis and inhibit cancer progression without causing severe side effects.

More recently, a second TEM8 KO mouse was generated and TEM8 was characterized as an essential component in controlling endothelial and fibroblast homeostasis in the skin [16]. The skin of TEM8 KO mice presented with altered levels of endothelial basement membrane components including members of collagen type I and VI, as well as hyperproliferative and leaky blood vessels [16]. These findings contradict previous findings showing no role for TEM8 in a normal physiological state. In osteosarcoma cells the knock-down of TEM8 led to reduced cell proliferation [17], showing that TEM8 can have dual roles depending on the setting. Furthermore, TEM8 was found to interact with lipoprotein receptor-related protein 6 (LRP6); modulating signaling down-stream of Wnt, a protein that induces both cell proliferation and migration [18, 19]. TEM8 was shown to be a functional marker for cancer stem cells in breast cancer, by activating Wnt signaling and by acting in a signaling network with collagen VI, impacting breast cancer stem cell characteristics and metastatic potential by positively regulating tumor growth [20]. TEM8 was associated with a more invasive and aggressive phenotype in breast cancer [20] and was found to be upregulated in invasive breast cancer [3]. Furthermore, we found that TEM8 expression is higher in tumors compared to normal tissue in both breast cancer and colon cancer patient samples.

We used CRISPR/Cas9 to create TEM8 KO cells and found that tumor progression *in vivo* is significantly inhibited when cancer cell TEM8 expression is lost. Using microarrays, we demonstrate the altered expression pattern of many genes in cancer cells upon TEM8 KO, the majority of which are genes involved in cell cycle regulation. Importantly, we show that tumor angiogenesis is reduced and the metastatic burden is significantly lowered in breast cancer when TEM8 is disrupted in cancer cells. These data highlight the role of cancer cell-derived TEM8 in driving cancer progression and further demonstrate its potential as a therapeutic target to fight the disease.

## RESULTS

### Expression of TEM8 is associated with disease in breast and colorectal cancer

TEM8 has previously been suggested to be specifically expressed in the tumor microenvironment [21]. We therefore explored the expression levels of TEM8 and its possible association with the disease phenotype in breast cancer and colorectal cancer. We used Disease-Specific Genomic Analysis (DSGA) [22], a computational data analysis method that mathematically identifies the signature of healthy cells from either one cell type or tissue, the normal component (*NcT*). DSGA then highlights aberrant expression signatures of diseased cells or tissue, the Disease component (*DcT*), by comparing it to the healthy signature.

**Figure 1:**
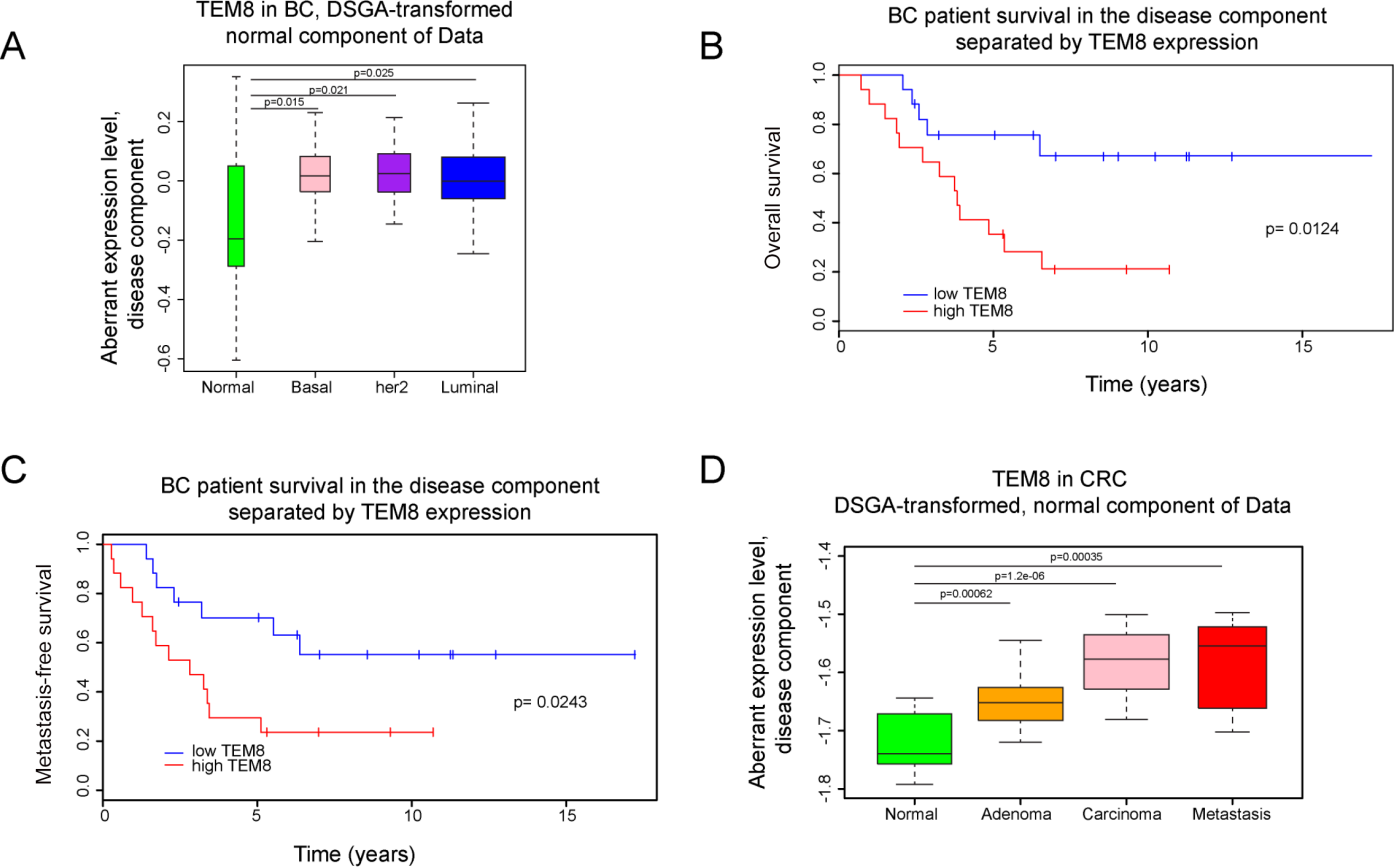
Expression of TEM8 is associated with disease in breast and colorectal cancer (**A**) DSGA-transformation of the NKI breast cancer (BC) data set, as described in Materials and Methods. Expression of TEM8 in the “normal-like” cell states of tumors (*NcT)* from basal (*n* = 49), her2 positive (*n* = 53), and luminal breast cancers (*n* = 193) are significantly higher than in normal breast tissue (*n* = 13). (**B**–**C**) Kaplan–Meyer survival curves, death (*p* = 0.0124) and metastasis (*p* = 0.0243), of breast cancer patients with her2-overexpressing breast tumors based on TEM8 expression. Group 1 is the bottom 33% and Group 2 is the top 33% of TEM8 expression in the disease component (*DcT)*. (**D**) DSGA-transformation of the CRC data set. Expression of TEM8 in the “normal-like” cell states of tumors (*NcT)* from adenoma (*n* = 17), carcinoma (*n* = 17), and metastasis (*n* = 11) are significantly higher compared to normal (normal crypt and surface epithelium combined, *n* = 13) samples.

To perform the DSGA-decomposition [22] we combined gene expression microarray data from a breast cancer cohort at the Dutch Cancer Institute (NKI) of 295 breast cancer tumors [23] and 13 normal breast tissue samples (BCN) (comprising 10 pathologically normal breast samples distant to tumors in breast cancer patients and 3 reduction mammoplasties [22]). We found the expression of TEM8 in the *normal-like cell states* of tumors, *NcT,* to be significantly higher than in cell states of normal breast tissue (Figure 1A). This difference was independent of the breast cancer molecular subtype. No difference in the distributions of aberrant cell state (*DcT*) compared to normal tissue was observed (data not shown). These results are in agreement with previous reports [2, 3, 20]. While the overall distribution of TEM8 was similar to normal in the disease component of tumors, patients with her2-overexpressing tumors that contained high levels of TEM8 presented with a significantly worse association with overall survival (Figure 1B) and metastasis (Figure 1C).

Next, we analyzed the normal component and disease component of a publicly available colorectal cancer data set consisting of 13 normal tissue samples (7 samples from normal colonic crypts (NC) and 6 samples from normal colonic surface epithelium (NS)), 17 adenomas, 17 carcinomas and 11 metastases [24]. We performed 3 independent DSGA data decompositions: using the combined normal samples, using only the normal colonic crypt samples, and using only the normal colonic surface epithelium samples. The results were similar, showing the behavior of TEM8 to be the same, whether tumors originate from crypt cells, surface cells, or a combination of the two. We found that more aggressive tumors had higher TEM8 in the normal component compared to normal and adenomas (Figure 1D). As with the breast cancer data there was no significant difference in the disease component in the CRC data set. Unfortunately, no survival data was available to further explore the association of TEM8 levels in the disease component. Overall, these data demonstrate that TEM8 expression is increased in both breast and CRC tumors compared to healthy tissue, and that high levels of TEM8 are associated with worse outcome in terms of patient survival and metastasis.

### TEM8 reduces the expression levels of cell cycle-related genes

The effect of TEM8 on cancer cell growth has previously been assessed by growing human tumor xenografts in a TEM8 KO mouse model [2]. Host-derived TEM8 was found to positively influence the growth of primary tumors, however tumor growth was still observed in the TEM8 KO mouse suggesting that tumor-derived TEM8 could also promote growth.

**Figure 2:**
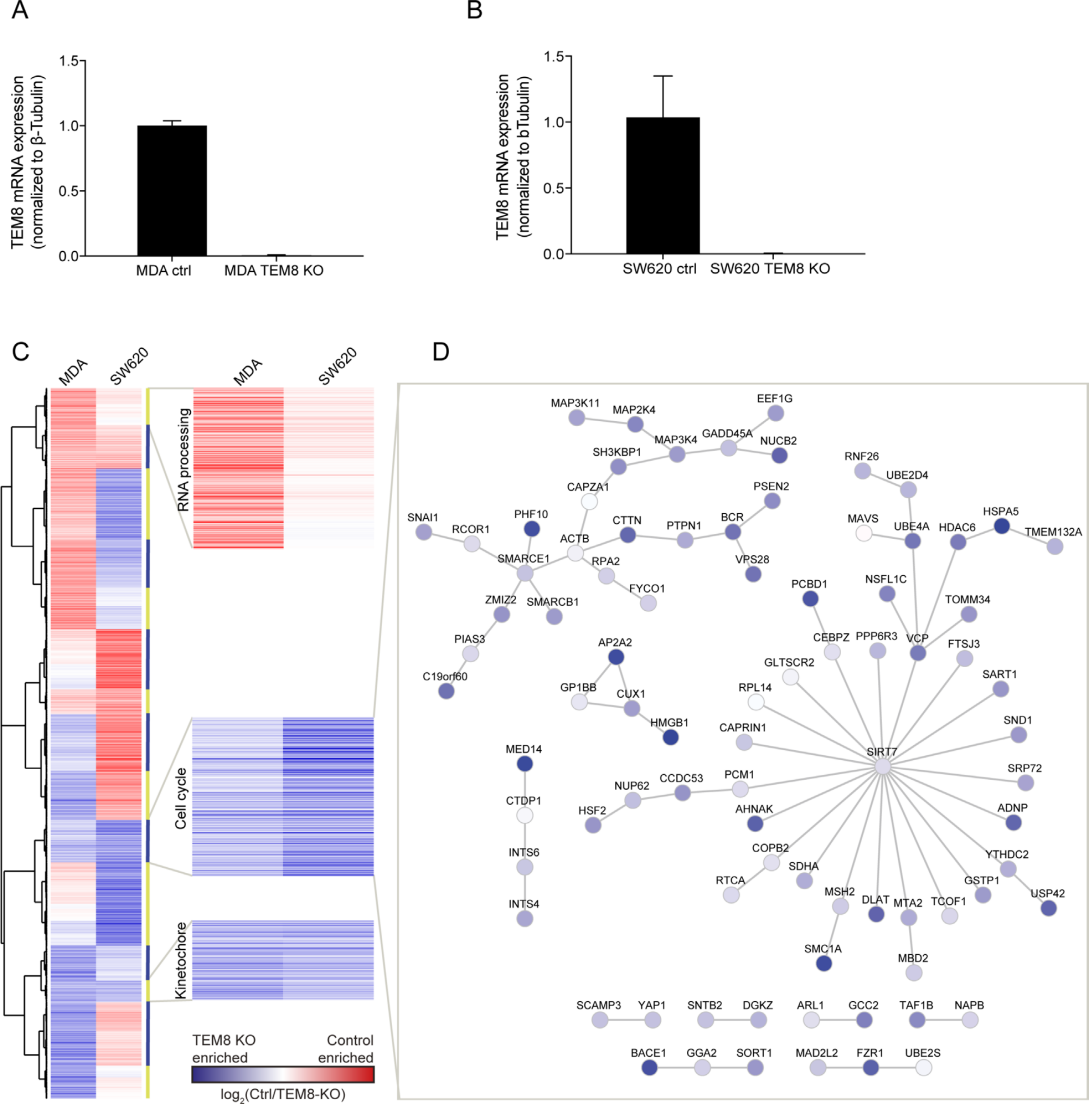
The expression of cell cycle-related genes is enriched in TEM8 KO cancer cells (**A** and **B**) Real-time quantitative PCR evaluating the mRNA expression levels of TEM8 confirms knockdown in MDA TEM8 KO and SW620 TEM8 KO cell lines compared to their control (ctrl) cell lines, MDA and SW620 respectively. *n* = 3 independent experiments, Statistical significance was assessed by One-way ANOVA, *p* < 0.0001. (**C**) Clustering analysis of gene expression in MDA and SW620 TEM8 KO cell lines (*n* = 3 independent experiments). The average of values for probes corresponding to one gene was used in the comparison. Proteins involved in blocking cell cycle and division are highlighted. (**D**) Network analysis of proteins interacting in the cell cycle cluster where similar in both MDA and SW620 cell lines. Node color here depicts values from SW620 control and SW620 TEM8 KO cell lines.

To assess this and to investigate if there are molecular changes caused by the loss of TEM8, we created TEM8 KO clones of the human metastatic breast cancer cell line MDA-MB-231 (MDA) and the human metastatic colorectal cancer cell line SW620 using the CRISPR/Cas9 gene-editing technique. Clones from each cell line with no detectable TEM8 mRNA were chosen for further analysis (Figure 2A, 2B). The selected cell lines, termed MDA TEM8 KO and SW620 TEM8 KO, were Sanger sequenced and examined for indels and the indels were found to be homozygous (Supplementary Figure 1). The MDA TEM8 KO cell line had an insertion and the SW620 cell line had a deletion leading to premature stop codons in exon 1 (Supplementary Figure 1).

Using the generated TEM8 KO cell lines, we carried out a microarray to compare gene expression in TEM8 KO cell lines with their respective controls (Supplementary Table 1). We performed a network analysis, where the gene names from the microarray were mapped onto a human interactome (see Materials and Methods), to assess whether TEM8 regulates the expression levels of potential interaction partners and their wider interaction networks. The analysis revealed no overall trend in the changed mRNA expression in response to a TEM8 KO, particularly when comparing between the two cancer types, but showed that the TEM8 KO affected many of the genes interacting with the TEM8 interaction partners (Supplementary Figure 2).

Next, we performed gene clustering analysis on the gene expression data to determine if there are any groups of genes that are affected by the loss of TEM8 in common to both breast and CRC cells (Figure 2C). Two clusters contained genes that were up-regulated in both MDA and SW620 TEM8 KO cell lines, and an additional cluster which contained genes that were down-regulated (Figure 2C). We performed functional gene ontological analysis of the gene clusters (Supplementary Table 2), and found that the two up-regulated clusters contained genes involved in cell cycle regulation and kinetochore assembly, whereas the down-regulated cluster contained genes involved in RNA processing (Figure 2C).

As an example we performed an interaction network analysis of the cell cycle-related cluster, which showed several cancer-related pathways affected by the loss of TEM8 such as members of the MAP kinase pathway (Figure 2D), which have previously been shown to be modulated by TEM8 in osteosarcoma cells [17]. The tumor suppressor p53 (USP42) [25] was also enriched in TEM8 KO cells (Figure 2D). In addition, the GTPase BCR that activates Cdc42 and Rac1, both proteins involved in migration and invasion of cells [26], is enriched in TEM8 KO cells. Similarly, we observed an enrichment of SMARCE1 and SMARCB1 (Figure 2D), two proteins that are core components of the BAF (SWI/SNF) chromatin remodeling complex and have been implicated as inhibitors of tumor formation [27]. Interestingly, SMARCB1 was previously found to be among the top candidate genes in a genetic screen for TEM8 interaction partners [28]. Taken together, these data suggest that the loss of TEM8 alters the gene expression profiles of MDA and SW620 cells, with common changes occurring in cell cycle-related genes known to be important for cancer progression.

### TEM8 affects *in vivo* tumor growth

Next, we investigated the importance of TEM8 in cancer proliferation since the expression of cell cycle-regulated genes was found to be increased upon loss of TEM8. We first compared proliferation of the TEM8 KO cells with control cells in both 2D and 3D proliferation assays. In the 2D proliferation assay, the MDA TEM8 KO cells proliferated significantly more slowly than MDA control cells (Supplementary Figure 3A). In a 3D proliferation assay MDA TEM8 KO cells showed a not significant trend of slowed proliferation compared to MDA control cells (Supplementary Figure 3B). Conversely, the control and TEM8 KO SW620 cells showed no difference in either 2D or 3D proliferation (Supplementary Figure 3C–3D). Hence, KO of TEM8 affects proliferation of breast cancer cells, but not colon cancer cells, *in vitro.*

**Figure 3:**
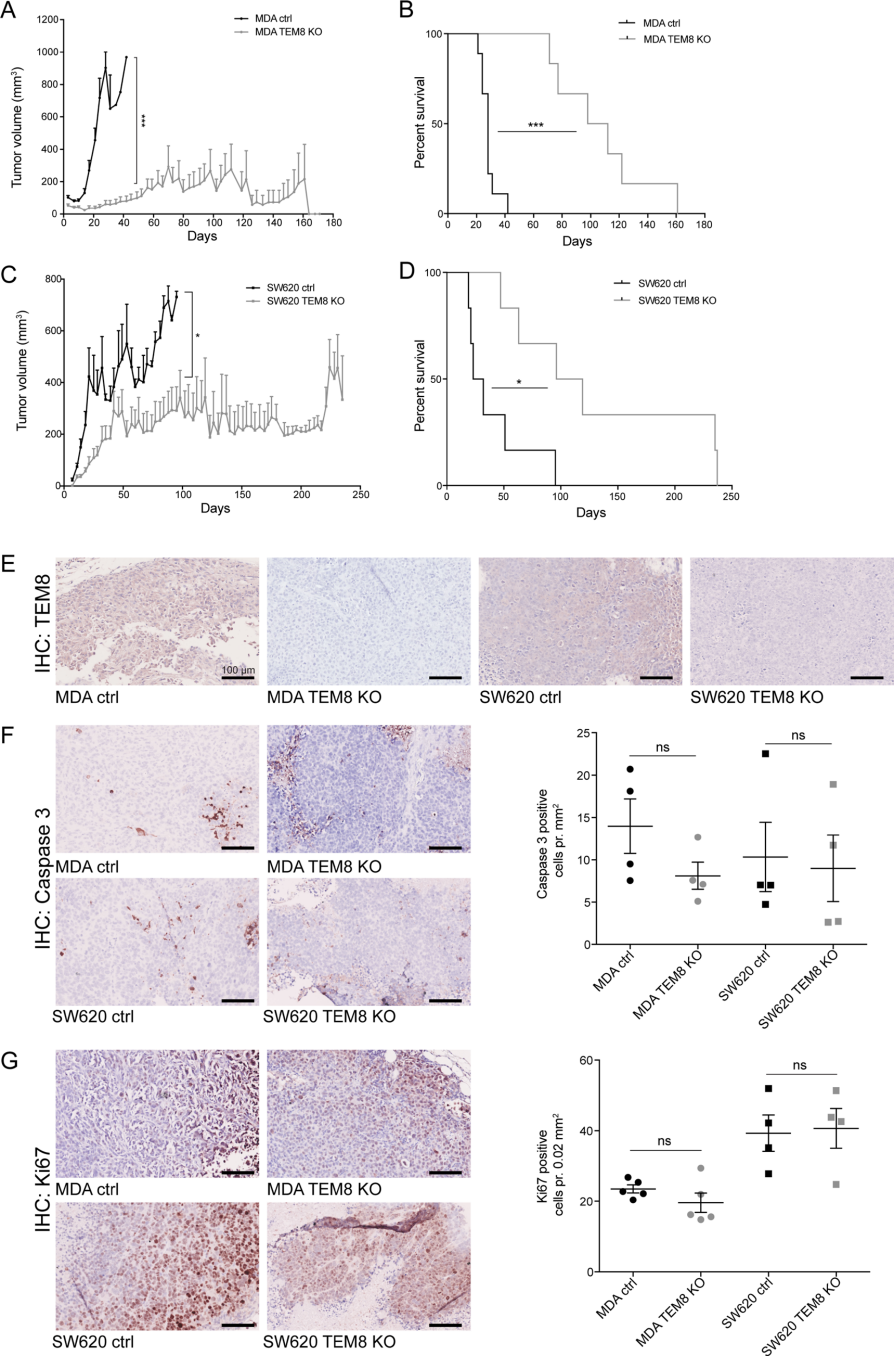
The knockout of TEM8 inhibits breast and colorectal cancer growth and prolongs survival *in vivo* (**A**) Average volume of MDA orthotopic tumors over time. Statistical significance was assessed by Unpaired two-tailed *t*-test. The graph represents two independent experiments of *n* = 4 and *n* = 5 tumors per group, *p* = 0.0002. (**B**) MDA survival curves. Statistical significance was assessed by Log rank test, *p ≤* 0.0001. (**C**) Average volume of SW620 subcutaneous tumors over time. Statistical significance was assessed by Unpaired two-tailed *t*-test, two independent experiments of *n* = 6 tumors per group, *p* = 0.0382, (**D**) SW620 survival curves. Statistical significance was assessed by Log rank test, two independent experiments of *n* = 3 mice per group, *p* = 0.0108. (**E**) TEM8 expression in TEM8 KO and control tumors was assessed by IHC. (**F**) IHC of cleaved caspase 3 in TEM8 KO and control tumors (left). Quantification of cleaved caspase 3 expression (right). Statistical significance was assessed by Unpaired two-tailed *t*-test *n* = 4 tumors, not significant (ns). (**G**) IHC of Ki67 expression in TEM8 KO and control tumors (left). Quantification of Ki67 expression (right). Statistical significance was assessed by Unpaired two-tailed *t*-test, *n* = 4 tumors, ns. (A+C) Data represented as mean + SEM. (**F**–**G**) Data represented as mean ± SEM.

To study the importance of TEM8 in cancer proliferation *in vivo* we used an orthotopic breast cancer model where either MDA control or MDA TEM8 KO cells were injected into the fat pad of mice. Tumor growth was dramatically reduced when TEM8 was knocked out compared to control (Figure 3A). At the experimental endpoint (160 days), 33% of the mice injected with MDA TEM8 KO cells were tumor-free. In the remaining 66% of the mice injected with MDA TEM8 KO cells, tumor growth was significantly slowed compared to controls which all developed tumors (Figure 3A). Consequently, the median survival rate was prolonged to 100 days, more than 4 times longer than the control group (24 days, Figure 3B, *p* < 0.0001). These results are consistent with the *in vitro* proliferation data, and suggest that TEM8 plays a role in breast cancer proliferation *in vivo*.

To validate our findings in a second *in vivo* cancer model, we tested the effects of TEM8 KO on subcutaneous tumor growth of the SW620 cells. Similar to the breast cancer model, tumor growth was significantly inhibited when TEM8 was knocked out (Figure 3C). In particular, when SW620 TEM8 KO tumors reached a size of around 200 mm^2^, their further growth was stalled. The median survival for the SW620 control mice was 27.5 days while in contrast, the SW620 TEM8 KO group had a median survival of 107.5 days (4 times longer than the control group, Figure 3D, *p* = 0.0108). These results demonstrate a significantly prolonged survival for the mice bearing SW620 TEM8 KO tumors and indicate that TEM8 is important for CRC tumor growth *in vivo.* Interestingly, the differences between the CRC *in vitro* and *in vivo* data suggest that the effect of TEM8 depends on the surrounding microenvironment.

To confirm that the tumors did not express TEM8 *in vivo* at the experimental end points, tumor sections were stained for TEM8 by immunohistochemistry (IHC). TEM8 was only expressed in control and not in TEM8 KO tumors (Figure 3E). Furthermore, both breast and CRC tumors were assessed by IHC for cells undergoing apoptosis (Caspase-3) and proliferation (Ki67) to evaluate if this could explain the *in vitro* proliferation and *in vivo* survival data. The number of cells expressing cleaved caspase-3 (Figure 3F) or Ki67 (Figure 3G) showed no change between control and TEM8 KO tumors, despite the differences seen in tumor volume and survival (Figure 3A–3D), suggesting that other mechanisms are responsible for these effects.

### TEM8 promotes tumor angiogenesis *in vivo* and stimulates endothelial cell migration *in vitro*

**Figure 4:**
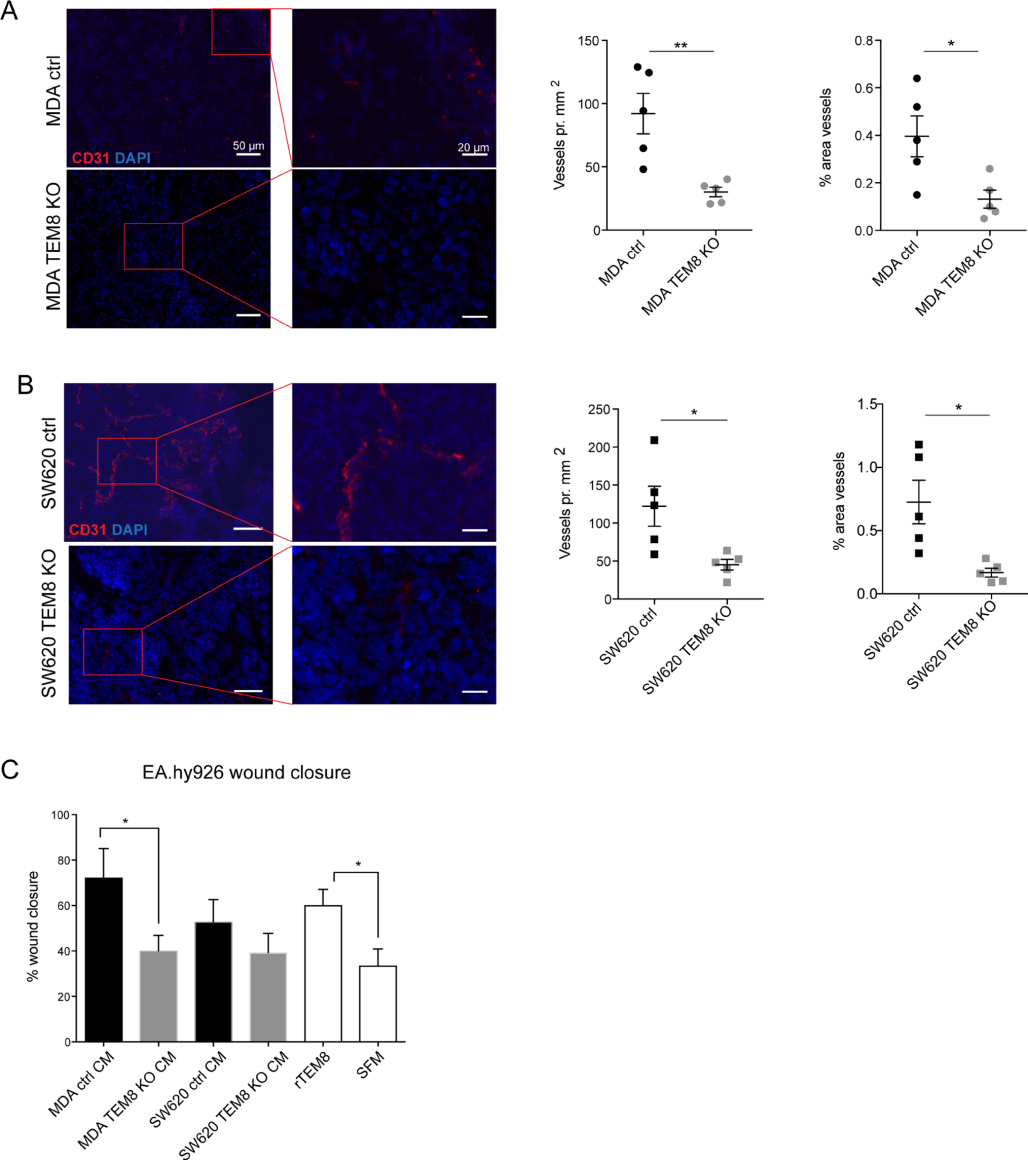
TEM8 promotes vessel formation in breast and colorectal tumors (**A**) Left, representative micrographs and corresponding magnified areas showing CD31 (red) and cell nuclei (DAPI, blue) in orthotopic MDA tumors. Rigth, quantification of vessel number and vessel area. Statistical significance was assessed by unpaired two tailed *t*-test, *n* = 5 mice, vessel number: *p* = 0.0054, area: *p* = 0.0225. (**B**) Left, representative micrographs and corresponding magnified areas showing CD31 (red) and cell nuclei (DAPI, blue) in subcutaneous SW620 tumors. Right, quantification of vessel number and vessel area. Statistical significance was assessed by unpaired two tailed *t*-test, *n* = 5 mice, vessel number: *p* = 0.0222, area: *p* = 0.0130. (**C**) Percent wound closure in endothelial EA.hy926 cells after eleven hours of migration treated with CM from TEM8 control or TEM8 KO cells, or recombinant TEM8 (rTEM8) as indicated. Serum free medium (SFM) was included as a negative control. Statistical significance was assessed by unpaired two-tailed *t*-test between MDA ctrl and TEM8 KO CM (*p* = 0.454) and unpaired two-tailed *t*-test between rTEM8 and SFM treated EA.hy926 cells (TEM8, *p* = 0.0261), *n* = 3 independent experiments.

TEM8 was originally discovered in human tumor endothelium [4] and has been associated with tumor angiogenesis [2]. Since we did not observe any differences in apoptosis or proliferation markers we hypothesized that altered angiogenesis in TEM8 KO tumors might explain the observed differences in tumor burden. Tumor sections were stained for the blood vessel marker CD31, and blood vessel number and area were quantified. Fewer vessels were observed in CD31 stainings of MDA TEM8 KO tumors compared to MDA control tumors (Figure 4A), and quantification of both vessel numbers and vessel area showed significantly reduced tumor angiogenesis in MDA TEM8 KO tumors (Figure 4A). Quantifications of CD31 staining of SW620 control and TEM8 KO tumors also showed a significant reduction in vessel number and area (Figure 4B).

As well as being a cell surface protein, TEM8 has been proposed to be secreted [29]. Thus, we investigated whether cancer cell-secreted TEM8 could stimulate endothelial cell migration and proliferation *in vitro*, which may explain the differences between the observed *in vitro* and *in vivo* CRC growth results. To do this, we treated EA.hy926 human umbilical vein cells with conditioned media (CM) from either control or TEM8 KO cells. In addition, we treated EA.hy926 cells with recombinant TEM8 (rTEM8) to evaluate the effect of soluble TEM8 alone on endothelial migration. Compared to treating EA.hy926 cells with CM from MDA control cells, there was a significant decrease in endothelial cell migration upon treatment with CM from MDA TEM8 KO cells (Figure 4C). A similar although not statistically significant trend was observed on endothelial cell migration upon treatment with CM from SW620 control or SW620 TEM8 KO cells (Figure 4C). Furthermore, EA.hy926 cells treated with rTEM8 displayed significantly increased migration compared to EA.hy926 cells treated with serum free medium (SFM) (Figure 4C), demonstrating that secreted TEM8 was able to stimulate migration in EA.hy926 cells. In contrast, EA.hy926 cell proliferation was not altered upon CM stimulation with or without TEM8, or stimulation with rTEM8 (Supplementary Figure 4).

Together with the *in vivo* data, these results support a role for tumor-derived TEM8 in promoting angiogenesis through increased migration, but not proliferation, of endothelial cells.

### The loss of TEM8 inhibits breast cancer metastasis

**Figure 5:**
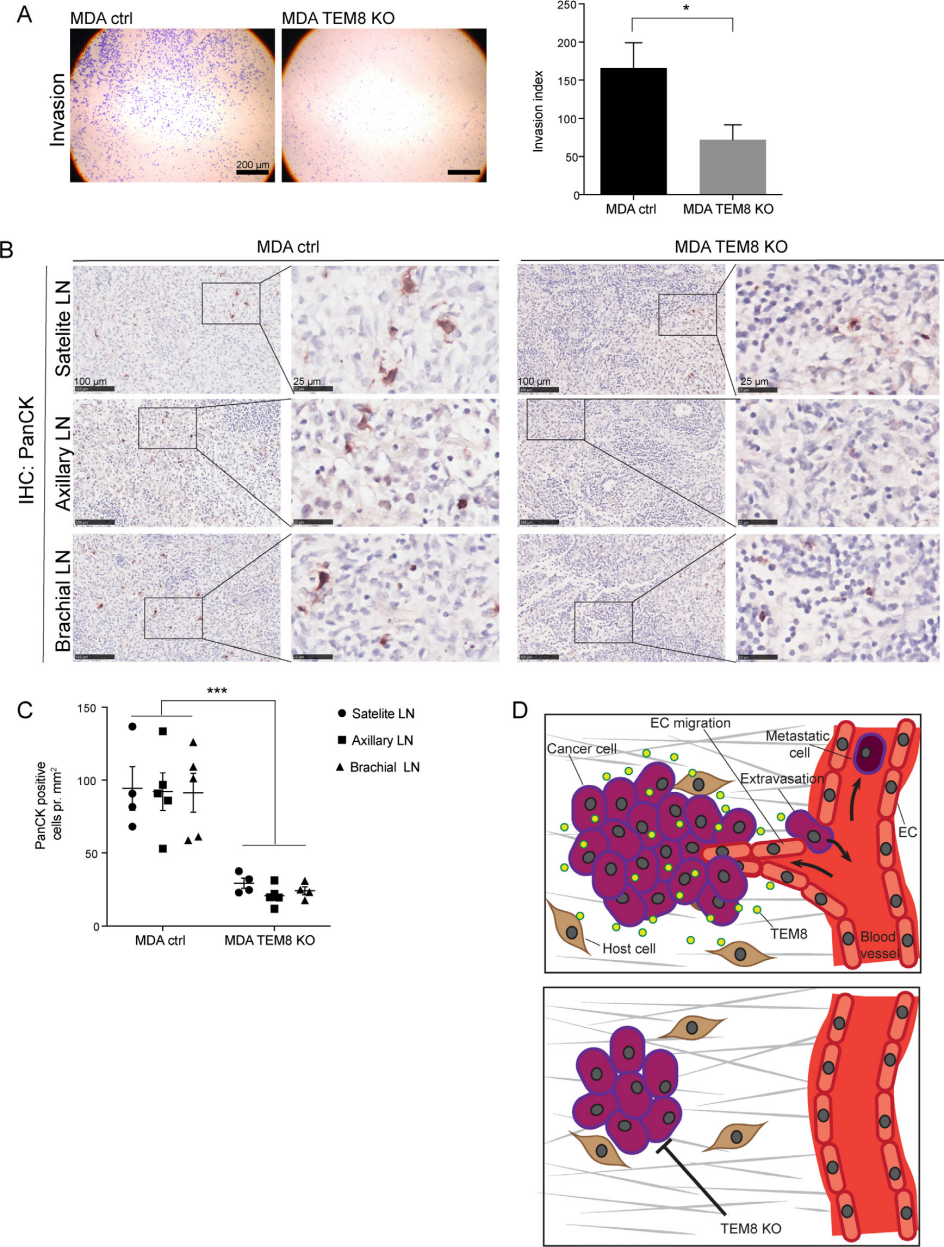
The knockout of TEM8 reduces lymph node metastasis (**A**) Representative micrographs of MDA cell invasion through Matrigel coated Boyden chamber invasion inserts (left) and the corresponding invasion index (right). Statistical significance was assessed by unpaired two tailed *t*-test, *n* = 4 independent experiments, *p* = 0.0279. (**B**) IHC of Cytokeratin (PanCK) expression in lymph nodes (LN) from orthotopic MDA control and TEM8 KO tumor bearing mice. Right panels show magnified areas. (**C**) Quantification of cytokeratin expressing cells in lymph nodes. Statistical significance was assessed by unpaired two-tailed *t*-test between all MDA control lymph nodes and all MDA TEM8 KO lymph nodes (three LNs per mouse, four mice in each group), *p ≤* 0.0001. (**D**) Suggested mechanism behind the impact of TEM8 on breast and CRC cancer progression. TEM8 expressed by cancer cells stimulates cancer cell proliferation. Cancer cell-secreted TEM8 affects endothelial cell (EC) migration and thereby increases tumor angiogenesis. This increase in tumor angiogenesis creates a favorable escape route for cancer cells embarking on the metastatic cascade. On the other hand, disruption of TEM8 induces the expression of cell cycle and kinetochore-related genes, resulting in reduced cancer cell proliferation and reduced angiogenesis, leading to reduced primary tumor growth and metastasis.

As angiogenesis is important in the invasion-metastasis cascade, and disseminating tumor cells typically escape the primary tumor via blood and lymphatic vessels [30], the anti-angiogenic effects of the TEM8 KO seen in breast and CRC tumors could have an effect on metastasis. We first used a Matrigel invasion assay to assess the importance of TEM8 for the invasive capacity of MDA cells and found that MDA TEM8 KO cells invaded significantly less compared to MDA control cells (Figure 5A). These *in vitro* results suggest that TEM8 may affect the metastatic burden of breast cancer *in vivo*.

To assess this possibility, we stained satellite, axillary and brachial lymph nodes collected from orthotopic breast tumor-bearing mice for the presence of tumor cells using a broad-spectrum cytokeratin (PanCK) antibody. Cytokeratin staining is routinely used in the clinic to detect and stage breast cancer metastasis [31, 32]. The cytokeratin staining showed a significant decrease in the number of cancer cells present in all lymph node types from mice with MDA TEM8 KO tumors compared to lymph nodes from mice with control tumors (Figure 5B–5C).

These findings demonstrate that TEM8 plays a role in breast tumor cell invasion and metastasis.

## DISCUSSION

In this study, we show that TEM8 regulates the expression of multiple genes. In particular, we observed that the most common expression changes conserved between breast and colorectal cancer are involved in regulation of the cell cycle. In line with the microarray results we show that TEM8 regulates cancer cell proliferation and primary tumor growth. Since TEM8 KO tumors presented with fewer blood vessels we hypothesize that TEM8 contributes to the regulation of angiogenesis, likely by being secreted by cancer cells to alter endothelial cell migration and thereby supporting growth of the tumor (Figure 5D). Moreover, we confirm that TEM8 is an important player in driving tumor cell invasion and metastatic dissemination in breast cancer.

To our knowledge this is the first study to utilize CRISPR/Cas9 to permanently disrupt the *TEM8* gene in cancer cells to study its tumorigenic effects. Since TEM8 has been shown to promote tumor angiogenesis [2, 4], which is also important for tumor growth [15], we assessed blood vessel number in TEM8 KO tumors. We hypothesized that reduced angiogenesis could explain the significantly slower growth of TEM8 KO tumors compared to controls, and found that tumor angiogenesis indeed was significantly reduced when TEM8 expression was disrupted. Furthermore, CM from TEM8 KO cells did not stimulate endothelial cell migration to the same extent as CM from TEM8-expressing cells. One could speculate that reduction in tumor angiogenesis due to loss of TEM8 could lead to an increase in hypoxic areas, which in turn could initiate pro-metastatic programs [33]. However, our data show reduced lymph node metastasis (Figure 5B–5C) suggesting this is not the case here.

Cancer cells are known to secrete pro-angiogenic signals such as vascular endothelial growth factor A (VEGFA) and induce an angiogenic-switch by engaging the tumor microenvironment [34]. We have previously shown that SW620 cells express LOX which stimulates secretion of VEGF into the CM, thereby affecting angiogenesis [35]. MDA-MB-231 cells can also secrete VEGF into the CM [36]. Combined targeting of TEM8 and VEGFR2 was more efficacious compared to targeting either one alone [2]. This suggests that TEM8 likely functions together with other factors, such as VEGF, to promote endothelial cell migration and angiogenesis.

Most angiogenic inhibitors that have been discovered to date cannot distinguish between physiological and cancer-related angiogenesis, and this has been a problem when using anti-angiogenic drugs in the clinic. As few physiological roles for TEM8 have been confirmed and TEM8 is mainly expressed by cancer cells and cancer-associated vasculature [4, 37], TEM8 could be a promising target for blocking tumor angiogenesis without eliciting the same side-effects and toxicity as other anti-angiogenic cancer therapies [38–40]. In agreement with our data and this hypothesis, another study showed that treatment with a TEM8-targeting antibody inhibited tumor proliferation and reduced the number of tumor blood vessels, without targeting normal physiology in mice [2].

The observed reduction of tumor blood vessel number in TEM8 KO tumors led us to investigate whether TEM8 is also important for metastasis, as disseminating cancer cells leave the primary tumor by entering the blood stream through the tumor blood vessel system [8]. We found that the number of disseminating tumor cells present in the lymph nodes of breast cancer-bearing nude mice was dramatically reduced when TEM8 expression was abolished (Figure 5), confirming that TEM8 supports metastasis. In line with our data, knock-down of TEM8 was previously shown to reduce lung metastasis and invasion of TMD-231 breast cancer cells into Matrigel [20], and overexpression of TEM8 in a 4T1 murine model of breast cancer resulted in increased colony formation in lymph and lung clonogenic assays [3]. Correlating with these results, treatment with an antibody-like TEM8 targeting molecule was found to inhibit liver metastasis of MCF-7 xenografts in mice [41], indicating that targeting TEM8 could be an anti-metastasis therapeutic approach in breast cancer. It has not yet been established where in the metastatic cascade TEM8 plays a role, and in order to investigate TEM8’s role in metastasis further, it would be interesting to assess the different metastatic steps; invasion (tumor cells escaping the primary tumor), circulation in the bloodstream, extravasation at secondary sites and initiation of angiogenesis at secondary sites [8]. Our *in vitro* and angiogenesis data show that breast tumor cell invasion was reduced in TEM8 KO cells, suggesting that TEM8 could be important in the first step of the metastatic cascade: escaping from the primary tumor (Figure 5D).

Our microarray data showed that the expression of genes regulating the cell cycle was up-regulated in TEM8 KO cancer cells. Up-regulation of this gene cluster could explain the reduced cell division and thus reduced proliferation and tumor growth of TEM8 KO cells compared to control cells. In support of our results, the loss of TEM8 has previously been linked to reduced proliferation by increasing p21 and p27 levels and by suppressing levels of cyclin D1 in osteosarcoma cell lines [20]. Similarly, cell cycle-related pathways were altered in a microarray analysis of breast cancer TMD-231 TEM8 shRNA cells [20].

Furthermore, a cluster of genes involved in kinetochore was up-regulated in both TEM8 KO cell lines, suggestive of a change in cell division and protein levels in the cancer cells. A network analysis showed that the KO of TEM8 changed the expression of several genes in close network proximity with TEM8 (Supplementary Figure 2), indicating that TEM8 plays a regulatory role driving cancer progression in both breast and colorectal cancer.

In summary, we found TEM8 to be upregulated in the normal-like cell states of tumors, and to be significantly higher in tumors than in cell states of normal breast and colorectal tissue. Experiments performed using CRISPR/Cas9 engineered TEM8 KO metastatic breast and colorectal cancer cell lines highlights a role for TEM8 in cancer progression, tumor angiogenesis and local metastasis. We propose that TEM8 targeting may provide a viable therapeutic treatment strategy against breast cancer and CRC tumor burden and metastasis. TEM8 was originally thought not to be expressed in normal tissue [4, 10], although it has since been shown by PCR to be broadly expressed, five splice variants of TEM8 have been detected [29], and studies in the most recent TEM8 KO mouse show a role for TEM8 during development [16]. However, we verified that TEM8 is up-regulated in cancer tissue compared to healthy, and treatment with a TEM8 antibody was reported to result in inhibited angiogenesis, reduced tumor growth and increased survival with no toxicity effects in a cancer setting [2]. TEM8 targeting could therefore be an alternative approach to more specifically block tumor angiogenesis compared to current clinically approved anti-angiogenic therapies. Further clinical studies are needed to explore the full potential of TEM8-targeted therapy.

## MATERIALS AND METHODS

### Disease-specific genomic analysis

Disease-Specific Genomic Analysis (DSGA) [22], is a computational data analysis method that provides a decomposition of log-transformed tumor data *T* into a *normal component* (*NcT)* and a *disease component* (*DcT)*. The normal component *NcT* is the best approximation of the tumor signature by a “normal-like” cell state. The disease component *DcT* is the deviation from this normal-like state, thereby highlighting the “aberrant” cell state. *T* = *NcT + DcT.* For breast cancer analysis gene expression microarray data from a breast cancer cohort at the Dutch Cancer Institute (NKI) (*n* = 295) [23] and normal samples (normal breast tissue (BCN) [22] (*n* = 13), pathologically normal breast distant to tumors in breast cancer patients (*n* = 10), and reduction mammoplasties (*n* = 3)) were used. Luminal tumors were not separated into molecular types (Luminal A and Luminal B). The separation of tumors into basal-like, her2-overexpressing and Luminal was based on ESR1 and ERBB2 status in the disease component, both of which have a clear bimodal distribution, allowing assignment of status based on *DcT* component of gene expression. Of the 295 tumors, 53 were her2-overexpressing by *DcT* analysis. One was borderline, ERBB2 overexpressing and thus was not included in the survival analysis. The same was observed when tumors were not separated by ESR1 and her2 status.

For analysis of TEM8 expression in CRC the published GSE77953 affymetrix HG-U133A data set [24] and ANTXR1 probe 220092_s_at were used. The CRC data set consisted of normal tissue samples (normal colonic crypts (*n* = 7) and normal colonic surface epithelium (*n* = 6)) and CRC samples (adenomas (*n* = 17), carcinomas (*n* = 17) and metastases (*n* = 11)).

All analyses and plots were performed using R version 3.4.4 with standard Bioconductor version 3.6 and the following packages: DSGA [22], survival, RMA, and ggpubr.

### Cell lines

The metastatic breast cancer cell line MDA-MB-231 (MDA) was derived from a patient with breast adenocarcinoma [42], the human metastatic CRC cell line SW620 was derived from a lymph node metastasis from a patient with colon adenocarcinoma [43], the HEK-293 cell line was derived from human embryonic kidney cells [44], and the EA.hy926 cell line was derived from human umbilical vein cells [45]. All cell lines were maintained at 37° C and 5% CO_2_ in Dulbecco’s modified Eagle medium (1x) GlutaMAX (DMEM, Gibco, ThermoFisher Scientific, Waltham, MA, USA) containing 10% Fetal Bovine Serum (FBS; Gibco) and 1% Penicillin Streptavidin (Gibco). All cell lines were validated by short tandem repeat (STR) analysis and routinely tested for mycoplasma.

### CRISPR/Cas9 mediated generation of TEM8 KO cells

MDA and SW620 TEM8 KO cells were generated using the plasmids pSPCas9 (BB)-2A-Puro (PX459) V2.0 (Addgene plasmid #62988) and pSpCas9n(BB)-2A-GFP (PX461) V2-0 (Addgene plasmid #48140), both a gift from Feng Zhang, according to published protocols [46, 47]. gRNAs targeting the first exon of TEM8 were designed using http://crispr.mit.edu. gRNAs were cloned into PX459 and PX461 CRISPR/Cas9 plasmids and cells were transfected using Lipofectamine 2000 as described previously [46]. To create MDA TEM8 KO the following gRNAs and plasmid were used: TEM8 KO gRNA1 Forward (5′-CACCGCAGGTCAAATCCGCCGTAGC-3′)/ TEM8 KO gRNA1 Reverse (5′-AAACGCTACGGCGGATTTGACCTGC-3′) and PX459. To create SW620 TEM8 KO the following gRNAs and plasmid were used: TEM8 KO gRNA2 Forward (5′-CACCGTGCTCATCTGCGCCGGGCAA-3′)/ TEM8 KO gRNA2 Reverse (5′-AAACTTGCCCGGCGCAGATGAGCAC-3′), TEM8 KO gRNA3 Forward (5′-CACCGCCACTGGAAGCCGATGCCG-3′)/TEM8 KO gRNA3 Reverse (5′-AAACCGGCATCGGCTTCCAGTGGC-3′) and PX461. Successfully transfected cells were either selected using 4 µg/ml puromycin or FACS sorted for GFP expression as appropriate. Selected TEM8 KO cell lines were verified by Sanger sequencing, The primer pair TEM8 forward (5′-GCGAGGGGGAATAAAGGACC-3′)/ TEM8 reverse (5′-TAATGCCTTCCGTGGGACAG-3′) was used for sequencing. TEM8 gDNA was amplified by polymerase chain reaction (PCR) using the Phusion Flash High-Fidelity PCR Master Mix (ThermoFisher Scientific). PCR amplicons were purified using GFX PCR DNA and Gel band Purification Kit (GE Healthcare Life Science, Cleveland, OH, USA) according to manufacturer’s instructions. The PCR amplicons were sequenced at GATC-Biotech (Constance, Germany) and ABI sequencing files were analyzed by Geneious [48] and by CRISPR ID [49].

### RNA and DNA extraction

Two hundred and fifty thousand MDA or 6.25 × 10^5^ SW620 cells were seeded in a 6-well plate format. The cells were incubated at 37° C overnight. RNA or genomic DNA was collected according to the manufacturer’s instructions using an RNeasy kit (Qiagen, Venlo, Netherlands) or collected using a genomic DNA (gDNA) kit (Qiagen), respectively.

### RT-qPCR

Primers targeting the TEM8 DNA sequence downstream of the TEM8 DNA indels were designed in order to detect changes in mRNA expression levels caused by TEM8-targeting CRISPR/Cas9: TEM8 forward (5′-TGGGTCCTACTGAGGAAAGG-3′)/ TEM8 reverse (5′-GACCCTGGTGAAGTTGATGC-3′) and β-tubulin forward (5′-GCGAGATGTACGAAGACGAC-3′)/β-tubulin reverse (5′-TTTAGACACTGCTGGCTTCG-3′). 2 µg RNA from the CRISPR modified cancer cells was reverse translated into complementary DNA (cDNA) with Moloney Leukemia Virus Reverse Transcriptase (ThermoFisher Scientific). 10 µl LightCycler 480 SYBR Green Master (Roche, Basel, Switzerland) pr. 50–100 µg cDNA and 0.5 µM primers was used as detection method. Samples were run in a Lightcycler 480 II (Roche). Tubulin was used as an internal expression control. Samples were run in triplicate.

### Gene expression microarray

A One-Color SurePrint G3 Human Gene Expression version 3 microarray (Agilent, Santa Clara, CA, USA), comparing gene expression in MDA-MB-231 and SW620 TEM8 KO cell lines to their respective control cell lines, was carried out according to manufacturer’s instructions (*n* = 3). The comparison of log fold differences in gene expression between TEM8 KO and control cell lines were found by using the average value for probes corresponding to one gene. The pair-wise comparison was performed on the average values of the three individual repeats.

### Bioinformatics analyses of microarray data

Gene expression values from both MDA and SW620 cell lines were hierarchically clustered on the basis of uncentred Pearson correlation using Cluster 3.0 [50] (C Clustering Library, version 1.52) and visualized using Java TreeView [51] (version 1.1.6r4). Fifteen clusters were chosen on the basis of a Pearson correlation threshold greater than 0.8 (Figure 2C). Functional enrichment analysis of clustered genes was performed using DAVID [52] (version 6.7) using biological process and cellular component gene ontology categories. Keywords with fold enrichment ≥1.5, Bonferroni-corrected *P* value < 0.05, EASE score (modified Fisher’s exact test) < 0.05 and at least two genes per keyword were considered significantly over-represented. Interaction network analysis was performed using Cytoscape [53] (version 3.0.2). Gene names were mapped onto a merged human interactome consisting of protein–protein interactions reported in the Protein Interaction Network Analysis database [54], the MatrixDB database [55] and the literature-curated integrin adhesome [56, 57]. TEM8, the TEM8-binding proteins BRCA1, HDAC2 and LRP6; and their associated interacting proteins were extracted and displayed as networks. Node color represents expression changes between control and TEM8 KO cells in MDA (Supplementary Figure 2A) and SW620 (Supplementary Figure 2B) cell lines.

### *In vivo* studies

Female CD1 homozygous nude mice (Scanbur, Karlslunde, Denmark) were used for all *in vivo* experiments. Mice were received at an age of 6–8 weeks and kept for 1 week before starting experiments. All experiments were carried out under authorization and guidance according to the Danish Inspectorate for Animal Experimentation.

### Mammary fat pad tumors

Ten million MDA control or TEM8 KO cells in 100 μl PBS were injected into the inguinal 4th right mammary fat pad of 8–10 week old female nude mice (two individual repeats of five mice per cell line). 1 ml syringes and 25 gauge needles (BD Biosciences) were used for the injections. Tumor volume and body weight were measured twice weekly using a digital caliper (Faithfull tools, Norwich, UK). Mice were sacrificed using cervical dislocation when tumors reached a size of 1000 mm^3^. Tumors were collected and a part of each tumor was snap frozen in dry ice and kept for protein, DNA and RNA analyses and immunofluorescence (IF). A second part of the tumor and lymph nodes (satellite, axillary and brachial) were embedded in paraffin after 24 h in formalin (Sigma Aldrich) and stored for immunohistochemistry (IHC).

### Subcutaneous CRC tumors

SW620 control or TEM8 KO cells were implanted as subcutaneous tumors in 8–10 week old female nude mice (two repeats of six tumors, 3 mice per cell line) as previously described [58]. Tumor volume and body weight were measured twice weekly using a caliper. Mice were sacrificed and tumors collected and processed as described above.

### Immunohistochemistry

Formalin-fixed, paraffin-embedded tumors were cut into 3 μm sections on a Microm HM355S microtome (Thermo Scientific) and placed on Superfrost slides. Sections were deparaffinized in xylene, hydrated in ethanol and rinsed in tap water. Antigens were retrieved with TEG Buffer (10 mM Tris, 0.5 mM EGTA, pH 9.0 in ultra pure water) at 98° C for 15 min (Ki67 and Caspase 3) or with Proteinase K Buffer (50 mM Tris, 0,5 M EDTA, pH 8,0, 0.5 ug/ml Proteinase K) at 37° C for 15 min (PanCytokeratin). For TEM8 IHC no antigen retrieval was performed. Endogen peroxidases was blocked with 1% H2O2 (VWR International, Søborg, Denmark) in MQ for 15 min. Slides were rinsed in TBS with 0.5% Triton-X 100 (TBS-Tx) and incubated in 100 μl Antibody Diluent with background reducing agents (DAKO, Copenhagen, DK) and primary antibodies (rabbit anti-human TEM8 (ab21270, Abcam) at 1:300 dilution, rabbit anti-human cleaved caspase 3 (D175, Cell Signaling Technology) at 1:500 dilution, rabbit anti-human Ki67 (ab92742, Abcam) at 1:1000 dilution, hamster anti-human CD31/ PECAM-1 (MA3105, Thermo Fisher) at 1:100 dilution, rabbit anti-human PanCytokeratin (PanCK) (ab9377, Abcam) at 1:100 dilution) as appropriate at 4° C overnight. Slides were rinsed in TBS-Tx and incubated in 100 μl EnVision+ System–HRP secondary antibody (DAKO) corresponding to the host of the primary antibody for 45 min. Subsequently slides were rinsed in TBS-Tx and incubated in 150 μl NovaRED (Vector laboratories, Burlingame, CA, USA) for 10 min to visualize antibody-positive cells followed by rinsing in MQ water and incubation in 100 μl Meyers Hematoxyline (Sigma Aldrich) for exactly 30 sec to visualize cell nuclei. Slides were rinsed in tap water, dehydrated in ethanol and mounted on coverslips with DPX mount for histology (Sigma Aldrich). Slides were scanned with a NanoZoomer digital slide scanner (Hamamatsu, Bridgewater, NJ, USA) and the number of protein-expressing cells in each tumor was quantified manually with the NDP view 2 software (Hamamatsu).

### Immunofluorescence

Snap frozen tumors were cut into 5 μm sections and placed on Superfrost slides. Sections were fixed in 4% PFA for 10 min, rinsed in TBS with 0.05% Tween (TBST) and permeabilized using 1% TBS-Tx for 30 min. Unspecific protein binding was blocked with 5% normal serum from the host of the secondary antibody diluted in TBST for 30 min. Slides were incubated in primary antibody diluted in TBST at 4° C overnight. Slides were then rinsed in TBST and incubated with secondary antibody diluted in TBST for 1 h. Finally, slides were incubated with Dapi 1:1000 (ThermoFisher Scientific) for 10 min to visualize nuclei, and mounted using glass coverslips. Tumor sections were visualized on a DeltaVision (GE Healthcare Lifescience), and Velocity software (PerkinElmer, Waltham, MA, USA) was employed to evaluate protein expression levels.

### Conditioned media

Conditioned media (CM) was collected from cells as previously described [35]. Briefly, cells where seeded in complete media, after 24 h cells were washed twice with PBS and serum free media was added. After 24 h conditioned media (CM) was harvested. CM was added to complete media at a ratio of 1:20 and added to EA.hy926 cells. Complete media with 10 ng/ml recombinant TEM8 (Novus Biologicals, Littleton, CO, USA) or serum free medium (SFM) as negative control were added as appropriate.

### Scratch wound assay

One hundred and seventy five thousand EA.hy926 cells were seeded in a 12-well plate format in complete medium and incubated for 16 h. Cells were serum deprived in medium with 3% FBS for 3 h. Scratches were made in the EA.hy926 cell layer using a sterile pipette tip, and wells were subsequently rinsed in medium with 3% FBS. Wells were supplemented with 3% FBS medium with CM in duplicate and incubated in an IncuCyte at 37° C. Percentage wound closure was calculated using ImageJ software.

### Boyden chamber cell invasion assay

Twenty four-well format migration or invasion inserts with a pore size of 8.0 µm with no coating or Matrigel coating, respectively (Corning, NY, USA) were used for this assay. Cells were serum starved overnight. Inserts were rehydrated in 500 μl serum free medium for 2 h. 2.5 × 10^4^ cells in 400 μl serum free medium were added to the inner wells in duplicate. 600 μl complete medium was added to the outer well to promote migration or invasion. Inserts were then incubated at 37° C. After 24 h, the medium was aspirated and non-migrating cells were removed from inner wells using cotton buds before fixing cells by adding 750 μl methanol for 15 min, followed by 600 μl 0.1% Crystal Violet for 20 min at room temperature for visualization. Invading or migrating cells were imaged and counted on an Olympus BX51 microscope (Olympus, Tokyo, Japan) with a UIS2 4×/0.10 Plan C N ∞/-/FN22 objective. An invasion index was used to separate the cells invading from chemotactic (migrating) cells^25^: *Invasion index* = (invading cells/migrating cells) * 100.

### Statistics

For multiple comparisons of DSGA data one-sided Wilcoxon rank sum test was used for normal components, and two-sided Wilcoxon rank sum test was used for disease components. Kaplan–Meyer survival curves were used to compare the top and bottom thirds of TEM8 aberrant expression in patients with her2-overexpressing tumors. *P*-values were obtained using log-rank test. Experiments with two groups were analyzed with unpaired two-sided Student’s *t*-test and studies with three or more groups were analyzed with one-way ANOVA followed by Dunnett’s multiple comparison tests. Error bars represents standard error of the mean. Differences were considered significant at *p* < 0.05. Asterisks indicate significant differences and refer to the following *p*-values: ^*^ = *p* < 0.05, ^**^ = *p* < 0.01, ^***^ = *p* < 0.001, ^****^ = *p* < 0.0001, ns = non-significant.

## SUPPLEMENTARY MATERIALS FIGURES AND TABLES






