# Supplementary material for: Tumor endothelial marker 8 promotes cancer progression and metastasis
Source: Oncotarget. 2018 Jul 10;9(53):30173–88. doi: 10.18632/oncotarget.25734 (PMC6059023; doi:10.18632/oncotarget.25734)
Supplement: Supplementary file 1 [file oncotarget-09-30173-s001.pdf]

Tumor endothelial marker 8 promotes cancer progression and metastasis

SUPPLEMENTARY MATERIALS

2D proliferation assay

Five hundred cells were seeded in a 96-well plate format in technical triplicate and incubated at 37° C overnight. Plates were placed in an IncuCyte® ZOOM (Essen Bioscience, Ann Arbor, MI, USA) and incubated at 37° C for 7 days. Medium was changed after 3.5 days. Throughout the incubation period, four phase object confluence images were acquired from each well every other hour. Each phase object confluence point (an average of the four images from each well) was normalized to the start point confluence of the corresponding cell line. Data are from three independent experiments. For endothelial cell stimulation experiments using CM, 1000 EA.hy926 cells seeded in technical triplicate were treated with the different CMs as appropriate. Medium was changed every second day. Data are from three independent experiments.

3D proliferation assay

Twenty-four well plates were coated with 200 µl 1.8 mg/ml rat-tail collagen I (BD Bioscience, Franklin Lakes, NJ, USA) and incubated for 30 min at 37° C. 4×10^4 cells in 100 µl medium were mixed with 100 µl 1.8 mg/ml rat-tail collagen I and added to the collagen I-coated wells. The gels were incubated for 4 h at 37° C before 1 ml complete medium was added. Gels were incubated at 37° C for 10 days, then dissolved by treatment with 1 mg/ml collagenase type I (Invitrogen, ThermoFisher Scientific) for 1–2 hours at 37° C. The collagenase was inactivated using 0.25% trypsin and 1 mM EDTA. Cells were centrifuged, resuspended in 1 ml complete media and counted. Each condition was performed in technical duplicate. Data are from four independent experiments.

Supplementary Table 1: Quantitative expression values between control and TEM8 KO cells. See Supplementary_Table_1

Supplementary Table 2: Functional enrichment analysis of co-clustered genes. See Supplementary_Table_2

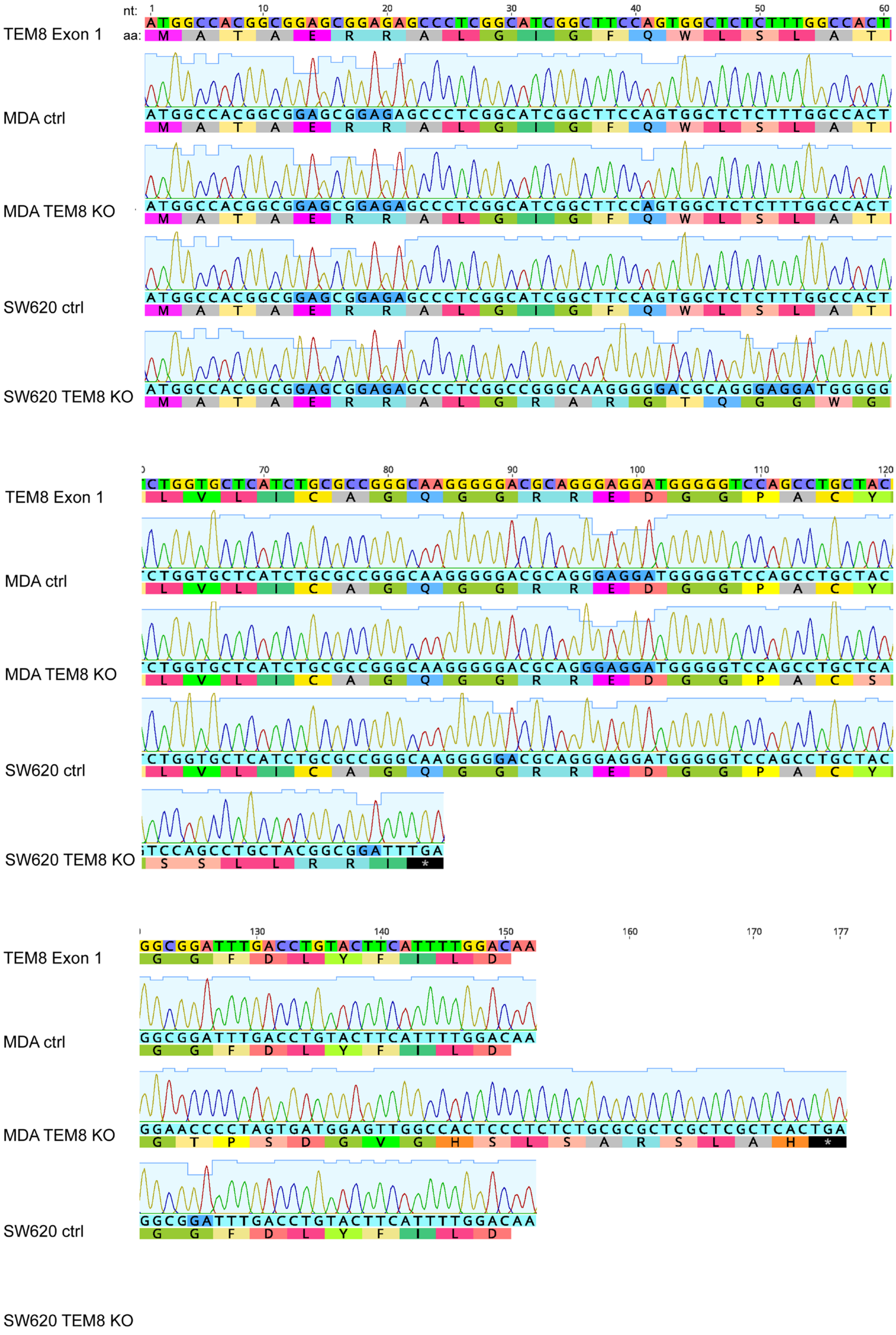

Supplementary Figure 1: Indels of TEM8 KO cell lines. ABI sequencing files of MDA ctrl, MDA TEM8 KO, SW620 ctrl and SW620 TEM8 KO cell lines compared to exon 1 of TEM8 (NM_032208.2). Nucleotide (nt); amino acid (aa).

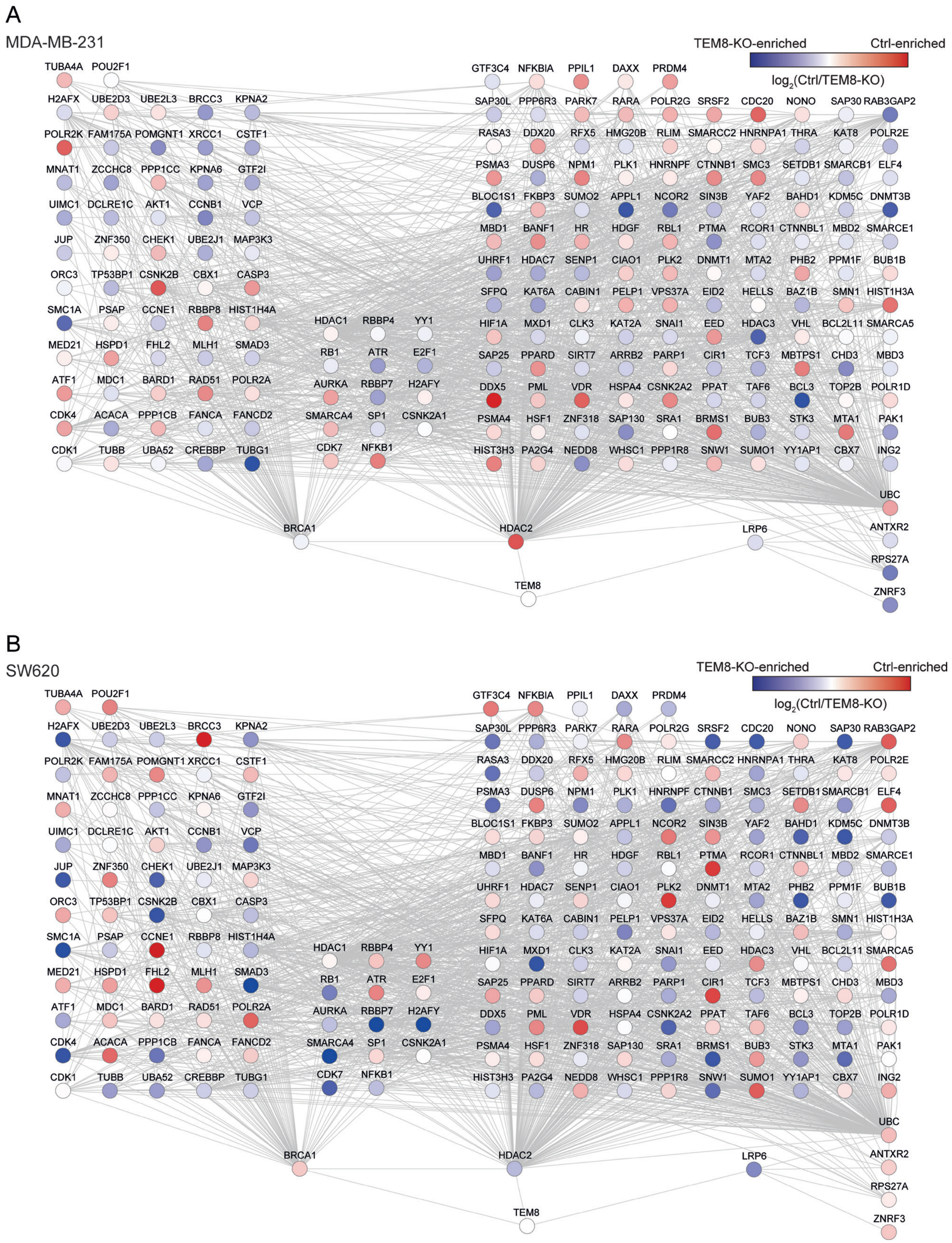

Supplementary Figure 2: Gene expression regulations in the TEM8 network in MDA and SW620 TEM8 KO cells. (**A**) Gene expression regulation in the TEM8 network in MDA TEM8 KO cells. Gene expression changes are marked in red (enriched in control cells) and blue (enriched in TEM8 KO cells). (**B**) Gene expression regulation in the TEM8 network in SW620 TEM8 KO cells. Gene expression changes are marked in red (enriched in control cells) and blue (enriched in TEM8 KO cells).

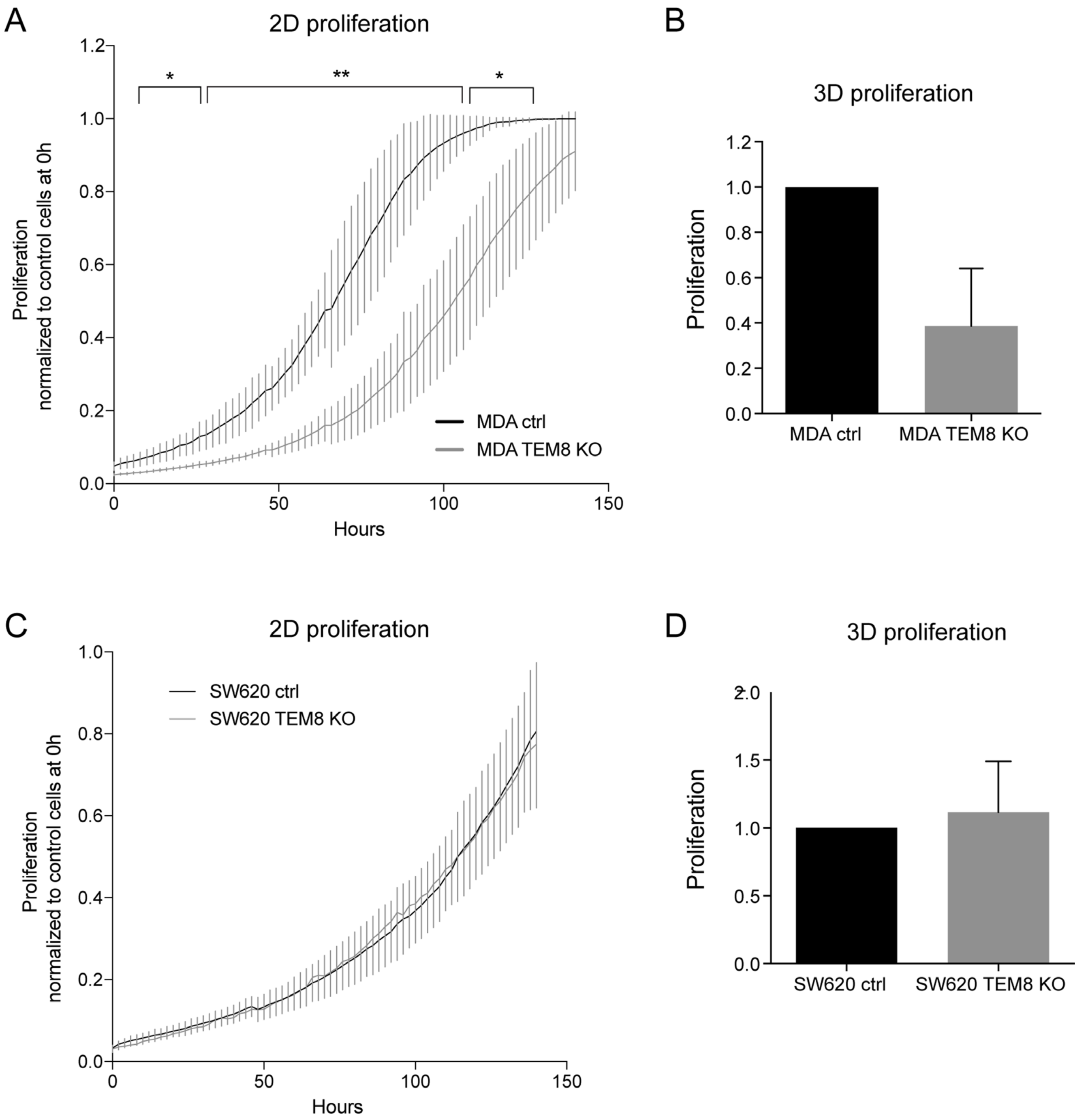

Supplementary Figure 3: Proliferation of MDA cells is influenced by TEM8. (**A**) 2D Proliferation of MDA control and MDA TEM8 KO cells. Statistical significance was assessed by Unpaired two tailed *t*-test at 10 time points, $n = 3$ independent experiments. (**B**) 3D proliferation of MDA control and MDA TEM8 KO cells in collagen I. Statistical significance was assessed by Unpaired two tailed *t*-test, $n = 4$ independent experiments, $p = 0.0732$. Proliferation was normalized to MDA control. (**C**) 2D proliferation of SW620 control and SW620 TEM8 KO cells. Statistical significance was assessed by unpaired two-tailed *t*-test at 10 time points, $n = 3$ independent experiments, ns. (**D**) 3D proliferation in collagen I. Proliferation was normalized to SW620 control. Statistical significance was assessed by unpaired two-tailed t-test, $n = 4$ independent experiments, ns.

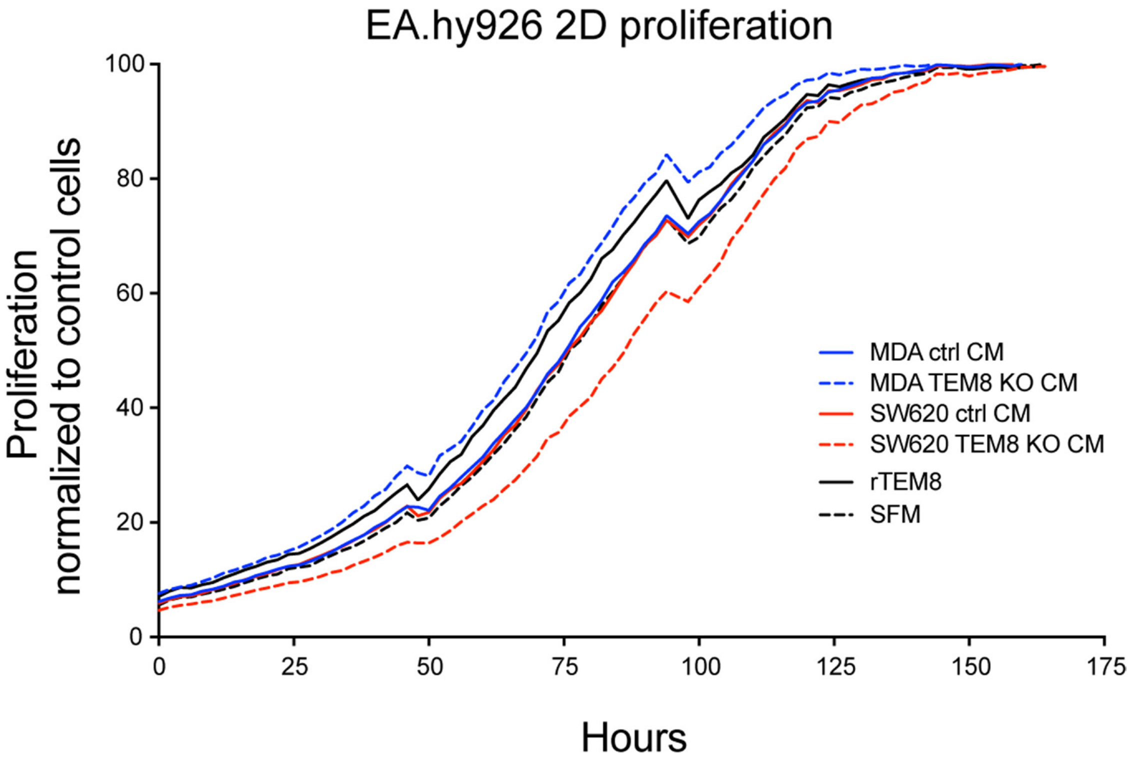

Supplementary Figure 4: Proliferation of CM treated EA.hy926. 2D proliferation of EA.hy926 cells treated with CM from TEM8 control or KO cells or recombinant rTEM8 as indicated. Serum free medium (SFM) was included as a negative control. n = 3 independent experiments, ns.
