# Supplementary material for: Tumor endothelial marker 8 promotes cancer progression and metastasis
Source: Oncotarget. 2018 Jul 10;9(53):30173–88. doi: 10.18632/oncotarget.25734 (PMC6059023; doi:10.18632/oncotarget.25734)
Supplement: Supplementary file 2 [file oncotarget-09-30173-s002.docx]

| **Supplementary Table 1.** Quantitative expression values between control and TEM8 KO cells. | | | | | | | | | | | |
| --- | --- | --- | --- | --- | --- | --- | --- | --- | --- | --- | --- |
|  |  |  |  | **MDA** | | | | **SW620** | | | |
| **Row Col** | **Probe Name** | **Systematic Name** | **Gene Symbol** | **log2FC ctrl vs KO** | **p-value ctrl vs KO** | **FDR ctrl vs KO** | **Bonf ctrl vs KO** | **log2FC ctrl vs KO** | **p-value ctrl vs KO** | **FDR ctrl vs KO** | **Bonf ctrl vs KO** |
| 7_163 | A_33_P3294002 | NM_017436 | A4GALT | -0.041305674 | 0.753316784 | 0.942109358 | 1 | 0.169771855 | 0.490437175 | 0.7464713 | 1 |
| 223_49 | A_33_P3292179 | NM_080283 | ABCA9 | -0.21166373 | 0.125722868 | 0.551421275 | 1 | 0.558876511 | 0.007058426 | 0.0748926 | 1 |
| 247_125 | A_23_P201918 | NM_012089 | ABCB10 | 0.03879997 | 0.766581277 | 0.945318698 | 1 | -0.139964393 | 0.235291344 | 0.5230919 | 1 |
| 247_114 | A_23_P5441 | NM_005689 | ABCB6 | 0.135514802 | 0.463450224 | 0.836159749 | 1 | -0.097432349 | 0.560161478 | 0.7943981 | 1 |
| 238_36 | A_23_P171258 | NM_004299 | ABCB7 | 0.032926727 | 0.785396559 | 0.94877096 | 1 | -0.019999651 | 0.894358767 | 0.9633162 | 1 |
| 149_156 | A_33_P3336780 | NM_001282291 | ABCB8 | -0.199061718 | 0.176625428 | 0.616374573 | 1 | -0.128920173 | 0.316187993 | 0.603456 | 1 |
| 110_94 | A_23_P258221 | NM_005688 | ABCC5 | -0.178300366 | 0.222637511 | 0.667816468 | 1 | 0.459432584 | 0.00195966 | 0.0332433 | 1 |
| 142_33 | A_33_P3298062 | NM_001023587 | ABCC5 | -0.095181156 | 0.473950837 | 0.839074131 | 1 | 0.020975278 | 0.882217637 | 0.9592556 | 1 |
| 32_160 | A_22_P00007933 | NR_046570 | ABCC5-AS1 | 0.014911267 | 0.923832781 | 0.983297021 | 1 | -0.000771859 | 0.995614746 | 0.9977042 | 1 |
| 340_49 | A_33_P3307955 | NM_001122674 | ABCD3 | -0.004998059 | 0.975697659 | 0.995056415 | 1 | -0.258162243 | 0.113150306 | 0.3655391 | 1 |
| 195_164 | A_23_P37327 | NM_005050 | ABCD4 | 0.105124638 | 0.437010979 | 0.826037245 | 1 | -0.073325682 | 0.592143112 | 0.8144146 | 1 |
| 375_118 | A_32_P108474 | NM_002940 | ABCE1 | 0.096874204 | 0.489746392 | 0.846353913 | 1 | 0.072607248 | 0.512143964 | 0.7608088 | 1 |
| 133_124 | A_23_P251660 | NM_001025091 | ABCF1 | 0.276882385 | 0.085881481 | 0.479280818 | 1 | -0.189024054 | 0.24671633 | 0.533927 | 1 |
| 347_75 | A_24_P251688 | NM_018358 | ABCF3 | 0.067411143 | 0.601377383 | 0.893153916 | 1 | -0.037803192 | 0.717227374 | 0.8837108 | 1 |
| 188_30 | A_23_P315933 | NM_148912 | ABHD11 | -0.285749698 | 0.068620152 | 0.451122877 | 1 | -0.089716508 | 0.500720462 | 0.7549066 | 1 |
| 103_84 | A_33_P3386765 | NM_015407 | ABHD14A | -0.015810037 | 0.914772412 | 0.980806062 | 1 | -0.291517071 | 0.114501444 | 0.3665867 | 1 |
| 195_60 | A_33_P3283713 | NM_015407 | ABHD14A | 0.182856422 | 0.313617563 | 0.74498319 | 1 | -0.175327136 | 0.330862432 | 0.6174232 | 1 |
| 109_96 | A_33_P3266265 | NM_001025780 | ABHD17B | -0.034041443 | 0.789335425 | 0.94877096 | 1 | -0.041556013 | 0.73465738 | 0.8930709 | 1 |
| 53_52 | A_23_P250294 | NM_016006 | ABHD5 | 0.057455329 | 0.635462779 | 0.902449007 | 1 | 0.089115739 | 0.422784278 | 0.6983342 | 1 |
| 359_31 | A_23_P211850 | NM_020676 | ABHD6 | -0.188542768 | 0.203840214 | 0.650388028 | 1 | -0.043875664 | 0.720931279 | 0.8858641 | 1 |
| 251_122 | A_33_P3302165 | NM_024527 | ABHD8 | -0.520361405 | 0.007273801 | 0.229117243 | 1 | 0.104687671 | 0.687561376 | 0.8689581 | 1 |
| 378_63 | A_23_P202520 | NM_001003408 | ABLIM1 | -0.093404105 | 0.565250652 | 0.880639801 | 1 | -0.057252487 | 0.672369823 | 0.8618809 | 1 |
| 83_91 | A_22_P00011158 | ENST00000453660 | ABO | -0.158189925 | 0.244142256 | 0.69010536 | 1 | 0.300837971 | 0.019886788 | 0.1374719 | 1 |
| 76_56 | A_23_P503182 | NM_021962 | ABR | 0.016146711 | 0.899713317 | 0.97618995 | 1 | -0.254764907 | 0.130039853 | 0.391291 | 1 |
| 212_145 | A_32_P160972 | NM_021243 | ABRACL | -0.257094595 | 0.080363226 | 0.468656078 | 1 | 0.570232056 | 0.004596502 | 0.057055 | 1 |
| 251_26 | A_33_P3297444 | NM_032548 | ABTB1 | -0.583910832 | 0.001336205 | 0.120457642 | 1 | 0.266055126 | 0.057764147 | 0.2535541 | 1 |
| 270_91 | A_33_P3358295 | NM_032548 | ABTB1 | -0.055129591 | 0.743985652 | 0.941128659 | 1 | 0.07182555 | 0.695227558 | 0.8731685 | 1 |
| 10_104 | A_23_P356616 | NM_145804 | ABTB2 | 0.457676959 | 0.008198986 | 0.234464792 | 1 | 0.067699909 | 0.671218275 | 0.8616372 | 1 |
| 379_108 | A_33_P3334225 | NM_198839 | ACACA | -0.167835737 | 0.473488935 | 0.838824187 | 1 | 0.366510582 | 0.017161685 | 0.1264031 | 1 |
| 58_35 | A_33_P3334220 | NM_001093 | ACACB | -0.630949536 | 0.000640533 | 0.089689296 | 1 | 0.986030519 | 0.000148018 | 0.006619 | 1 |
| 145_5 | A_23_P132405 | NM_014049 | ACAD9 | -0.281659922 | 0.091689903 | 0.49154825 | 1 | -0.079646389 | 0.609041109 | 0.8215262 | 1 |
| 164_12 | A_23_P96761 | NM_000016 | ACADM | -0.294706355 | 0.050225363 | 0.406002234 | 1 | 0.094541268 | 0.428361255 | 0.7020955 | 1 |
| 217_143 | A_33_P3248992 | NM_001609 | ACADSB | -0.071931504 | 0.554660004 | 0.875569848 | 1 | 0.01865932 | 0.894728644 | 0.9633162 | 1 |
| 24_119 | A_23_P207650 | NM_000018 | ACADVL | -0.197090411 | 0.21829519 | 0.665316989 | 1 | 0.086395435 | 0.53185166 | 0.7734338 | 1 |
| 231_16 | A_33_P3218741 | A_33_P3218741 | ACAN | -0.042638527 | 0.81312583 | 0.955007122 | 1 | 0.151807437 | 0.200064785 | 0.4832215 | 1 |
| 149_16 | A_33_P3398564 | NM_030649 | ACAP3 | -0.228909776 | 0.179757652 | 0.620483827 | 1 | 0.00221967 | 0.985603051 | 0.9949293 | 1 |
| 42_102 | A_33_P3230290 | NM_030649 | ACAP3 | 0.015113613 | 0.912536972 | 0.980010578 | 1 | 0.355199256 | 0.026501404 | 0.162875 | 1 |
| 326_49 | A_24_P203678 | NM_000019 | ACAT1 | -0.078678611 | 0.612278836 | 0.897674904 | 1 | -0.03031638 | 0.83427051 | 0.9374603 | 1 |
| 14_161 | A_23_P24515 | NM_000019 | ACAT1 | 0.552905013 | 0.019406233 | 0.305830187 | 1 | -0.521494472 | 0.019704302 | 0.1370527 | 1 |
| 359_164 | A_23_P31135 | NM_005891 | ACAT2 | 0.011851887 | 0.938074557 | 0.987634793 | 1 | 0.161342126 | 0.234241304 | 0.5220035 | 1 |
| 117_115 | A_23_P97795 | NM_145698 | ACBD5 | -0.203657227 | 0.152357754 | 0.59042598 | 1 | -0.33925073 | 0.020094545 | 0.1380127 | 1 |
| 97_51 | A_23_P52127 | NM_032360 | ACBD6 | -0.158259987 | 0.211659522 | 0.65826814 | 1 | -0.230328659 | 0.056452493 | 0.250462 | 1 |
| 151_149 | A_23_P72584 | NM_001039844 | ACBD7 | 0.03581859 | 0.812812096 | 0.955007122 | 1 | 0.166295695 | 0.458451561 | 0.7211225 | 1 |
| 78_55 | A_33_P3359071 | NM_001082486 | ACD | 0.010249544 | 0.940871702 | 0.988335297 | 1 | -0.028277914 | 0.804245351 | 0.9231312 | 1 |
| 158_57 | A_33_P3415042 | NM_001303274 | ACLY | 0.137894041 | 0.289391594 | 0.727382494 | 1 | 0.038754879 | 0.744033179 | 0.8965225 | 1 |
| 24_14 | A_23_P103149 | NM_001098 | ACO2 | -0.245305253 | 0.097445851 | 0.500605281 | 1 | 0.081168308 | 0.471619157 | 0.732537 | 1 |
| 192_47 | A_23_P103149 | NM_001098 | ACO2 | -0.151303853 | 0.278314075 | 0.718873421 | 1 | -0.033076442 | 0.767084063 | 0.9073239 | 1 |
| 374_97 | A_23_P103149 | NM_001098 | ACO2 | -0.148778311 | 0.300267389 | 0.736692697 | 1 | 0.055841706 | 0.602451226 | 0.8190176 | 1 |
| 270_76 | A_23_P103149 | NM_001098 | ACO2 | -0.109227537 | 0.379054432 | 0.790179752 | 1 | 0.033129593 | 0.803984138 | 0.9231312 | 1 |
| 293_140 | A_23_P103149 | NM_001098 | ACO2 | -0.074662013 | 0.590575478 | 0.88758105 | 1 | 0.076272524 | 0.504653938 | 0.7573621 | 1 |
| 174_79 | A_23_P103149 | NM_001098 | ACO2 | -0.048105478 | 0.726498747 | 0.936348816 | 1 | 0.092295397 | 0.566904714 | 0.7987945 | 1 |
| 76_155 | A_23_P103149 | NM_001098 | ACO2 | -0.000759298 | 0.995735243 | 0.998909536 | 1 | -0.007274531 | 0.947951074 | 0.9816413 | 1 |
| 243_65 | A_23_P103149 | NM_001098 | ACO2 | 0.00491086 | 0.969150078 | 0.994075478 | 1 | 0.067929343 | 0.661789407 | 0.8560259 | 1 |
| 62_81 | A_23_P103149 | NM_001098 | ACO2 | 0.025907309 | 0.833786925 | 0.961426617 | 1 | -0.017733155 | 0.888480283 | 0.9612584 | 1 |
| 197_60 | A_23_P103149 | NM_001098 | ACO2 | 0.131056035 | 0.336332035 | 0.762411026 | 1 | 0.043833291 | 0.755914408 | 0.9022593 | 1 |
| 263_32 | A_24_P161036 | NM_001037161 | ACOT1 | -0.041136581 | 0.746671541 | 0.941470372 | 1 | -0.107891374 | 0.411042982 | 0.686914 | 1 |
| 189_153 | A_23_P31116 | NM_018473 | ACOT13 | 0.044370075 | 0.721358699 | 0.935358583 | 1 | 0.63160244 | 0.001297944 | 0.0259848 | 1 |
| 244_38 | A_23_P125624 | NM_001037171 | ACOT9 | -0.167920221 | 0.205211789 | 0.65172692 | 1 | -0.997806989 | 4.18086E-06 | 0.0012709 | 0.04702 |
| 11_15 | A_24_P42501 | NM_001037171 | ACOT9 | 0.288263601 | 0.055508286 | 0.422185511 | 1 | -0.798821214 | 6.48936E-05 | 0.0043316 | 0.72986 |
| 235_57 | A_33_P3244803 | NM_001185039 | ACOX1 | -0.196519535 | 0.17163572 | 0.611722159 | 1 | -0.305281536 | 0.035068672 | 0.1922632 | 1 |
| 108_34 | A_23_P56798 | NM_004300 | ACP1 | 0.178946179 | 0.184689886 | 0.62763442 | 1 | -0.203938417 | 0.146513836 | 0.4149625 | 1 |
| 95_148 | A_32_P218332 | NM_174917 | ACSF3 | -0.078561646 | 0.618575054 | 0.89867047 | 1 | 0.0562474 | 0.660507931 | 0.8551551 | 1 |
| 245_72 | A_24_P248606 | NM_004457 | ACSL3 | -0.044746049 | 0.724887618 | 0.936348816 | 1 | 0.293771169 | 0.024202023 | 0.1547471 | 1 |
| 117_29 | A_32_P137939 | NM_001101 | ACTB | -0.139941721 | 0.346539897 | 0.768672438 | 1 | -0.00400601 | 0.981606171 | 0.9934423 | 1 |
| 332_163 | A_23_P135769 | NM_001101 | ACTB | 0.120346799 | 0.439431873 | 0.827404459 | 1 | -0.05749729 | 0.746151822 | 0.8976273 | 1 |
| 295_99 | A_32_P156963 | NM_001614 | ACTG1 | 0.251401696 | 0.221852156 | 0.667441137 | 1 | 0.095258592 | 0.781639831 | 0.9136251 | 1 |
| 295_142 | A_33_P3667484 | NR_024438 | ACTG1P4 | 0.180535142 | 0.206792483 | 0.654291494 | 1 | -0.054223076 | 0.685552951 | 0.868541 | 1 |
| 200_104 | A_23_P69249 | ENST00000461125 | ACTL6A | 0.579103194 | 0.031990451 | 0.346715011 | 1 | 0.016434957 | 0.939599296 | 0.9788735 | 1 |
| 35_71 | A_23_P105957 | NM_001102 | ACTN1 | 0.386308123 | 0.041009571 | 0.381801274 | 1 | -0.273724466 | 0.235001356 | 0.5228606 | 1 |
| 196_83 | A_23_P101655 | NM_004924 | ACTN4 | 0.218172666 | 0.177929505 | 0.617531975 | 1 | -0.062658927 | 0.681837527 | 0.8673936 | 1 |
| 311_121 | A_24_P278367 | NM_005736 | ACTR1A | -0.026775823 | 0.883692104 | 0.972820007 | 1 | -0.290873614 | 0.024913903 | 0.1579519 | 1 |
| 171_3 | A_23_P108785 | NM_005721 | ACTR3 | 0.07540605 | 0.566610964 | 0.880639801 | 1 | -0.042084345 | 0.776871027 | 0.9112879 | 1 |
| 27_19 | A_21_P0014389 | NM_005721 | ACTR3 | 0.106905602 | 0.423838279 | 0.819346019 | 1 | -0.10485727 | 0.380269261 | 0.6625641 | 1 |
| 71_42 | A_23_P123193 | NM_020445 | ACTR3B | 0.284617303 | 0.07925527 | 0.468455945 | 1 | 0.05950207 | 0.657818314 | 0.8536394 | 1 |
| 233_163 | A_23_P57868 | NM_000666 | ACY1 | 0.033857733 | 0.817571181 | 0.956101362 | 1 | -0.232423508 | 0.097126111 | 0.3391423 | 1 |
| 261_147 | A_23_P163143 | NM_001302617 | ACYP1 | 0.05442879 | 0.68585621 | 0.921904762 | 1 | -0.313315266 | 0.025907623 | 0.1610741 | 1 |
| 249_136 | A_23_P69339 | NM_001607 | ACAA1 | -0.213463977 | 0.171334125 | 0.611486797 | 1 | -0.393875069 | 0.017271179 | 0.1268772 | 1 |
| 283_155 | A_23_P89799 | NM_006111 | ACAA2 | -0.040447579 | 0.739600572 | 0.940422987 | 1 | 0.05994593 | 0.653053952 | 0.8501039 | 1 |
| 300_109 | A_23_P210482 | NM_000022 | ADA | -0.303790166 | 0.052898403 | 0.414085647 | 1 | -0.263825826 | 0.24105844 | 0.5286587 | 1 |
| 350_88 | A_33_P3380693 | NM_207197 | ADAM15 | -0.369438746 | 0.013122783 | 0.267459687 | 1 | 0.212701107 | 0.187909092 | 0.4686051 | 1 |
| 347_112 | A_23_P374082 | NM_033274 | ADAM19 | 0.321203265 | 0.028313223 | 0.333836104 | 1 | -0.442499966 | 0.029752976 | 0.1748337 | 1 |
| 77_76 | A_24_P300777 | NM_001109 | ADAM8 | 0.674142253 | 0.004897224 | 0.19718968 | 1 | 0.387288722 | 0.010724983 | 0.0956098 | 1 |
| 317_63 | A_23_P11005 | NM_014272 | ADAMTS7 | 0.066925609 | 0.656760132 | 0.910923281 | 1 | 0.208661351 | 0.107925441 | 0.3564868 | 1 |
| 225_5 | A_33_P3245489 | NM_213604 | ADAMTSL5 | -0.255301305 | 0.149487492 | 0.58620836 | 1 | 0.3032647 | 0.084713405 | 0.3135687 | 1 |
| 188_146 | A_24_P362317 | NM_001111 | ADAR | -0.002275787 | 0.986188848 | 0.995873498 | 1 | -0.037048557 | 0.751663969 | 0.9007747 | 1 |
| 194_69 | A_23_P211207 | NM_001112 | ADARB1 | -0.250566405 | 0.236014275 | 0.681859997 | 1 | -0.081848944 | 0.521470056 | 0.7670191 | 1 |
| 198_98 | A_22_P00017547 | NR_033387 | ADARB2-AS1 | 0.112837936 | 0.510062578 | 0.856630606 | 1 | -0.065221016 | 0.696456314 | 0.8734794 | 1 |
| 78_62 | A_33_P3602006 | NM_001286259 | ADAT2 | 0.011511134 | 0.959519954 | 0.992682116 | 1 | 0.096758388 | 0.511090643 | 0.7601476 | 1 |
| 230_10 | A_33_P3268793 | NM_138422 | ADAT3 | -0.171629193 | 0.22071573 | 0.667236886 | 1 | -0.38835616 | 0.003857044 | 0.0513485 | 1 |
| 302_75 | A_23_P67864 | NM_004036 | ADCY3 | 0.045424945 | 0.726416967 | 0.936348816 | 1 | -0.161055312 | 0.217581031 | 0.5038667 | 1 |
| 192_143 | A_23_P158925 | NM_145290 | ADGRA3 | 0.240005621 | 0.115529899 | 0.537023243 | 1 | 0.163450315 | 0.224958589 | 0.5136235 | 1 |
| 164_116 | A_33_P3269218 | NM_001294335 | ADGRB2 | -0.39606337 | 0.016916886 | 0.29259326 | 1 | 0.300565404 | 0.07275188 | 0.2880114 | 1 |
| 75_8 | A_23_P502312 | NM_078481 | ADGRE5 | -0.087648998 | 0.486678596 | 0.845462918 | 1 | -0.318952499 | 0.071228222 | 0.2841071 | 1 |
| 60_23 | A_23_P206280 | NM_201525 | ADGRG1 | -0.391678276 | 0.010871869 | 0.256586468 | 1 | -0.126296074 | 0.29272141 | 0.5846631 | 1 |
| 195_65 | A_33_P3305102 | NM_170776 | ADGRG3 | -0.019748477 | 0.92554986 | 0.983946976 | 1 | 0.001286161 | 0.996104643 | 0.9977042 | 1 |
| 336_105 | A_23_P148194 | NM_018269 | ADI1 | 0.401518584 | 0.041240408 | 0.382615991 | 1 | 0.145587536 | 0.390417115 | 0.6712047 | 1 |
| 10_150 | A_23_P48121 | NM_024551 | ADIPOR2 | 0.190930256 | 0.145030017 | 0.580405522 | 1 | -0.11005864 | 0.515812256 | 0.7627321 | 1 |
| 110_109 | A_23_P161439 | NM_006829 | ADIRF | -0.803294084 | 0.0005929 | 0.085984592 | 1 | -0.935903316 | 6.68676E-06 | 0.0013453 | 0.07521 |
| 301_14 | A_23_P112596 | NM_001123 | ADK | -0.344853451 | 0.025163347 | 0.321320639 | 1 | -0.197122234 | 0.106830827 | 0.3545371 | 1 |
| 318_34 | A_23_P112596 | NM_001123 | ADK | -0.333613609 | 0.022809674 | 0.313925645 | 1 | -0.233134532 | 0.083759719 | 0.3115222 | 1 |
| 252_35 | A_23_P112596 | NM_001123 | ADK | -0.319800982 | 0.068061016 | 0.450109361 | 1 | -0.277514704 | 0.050284972 | 0.2342558 | 1 |
| 214_17 | A_23_P112596 | NM_001123 | ADK | -0.194875415 | 0.14880314 | 0.585876178 | 1 | -0.286973128 | 0.039454226 | 0.2046194 | 1 |
| 11_64 | A_23_P112596 | NM_001123 | ADK | -0.167376363 | 0.244086797 | 0.69010536 | 1 | -0.179048988 | 0.159190988 | 0.4317973 | 1 |
| 104_84 | A_23_P112596 | NM_001123 | ADK | -0.157026313 | 0.328839419 | 0.759416455 | 1 | -0.273098666 | 0.04490028 | 0.2193011 | 1 |
| 76_141 | A_23_P112596 | NM_001123 | ADK | -0.149730868 | 0.25661668 | 0.699039445 | 1 | -0.293051004 | 0.033371129 | 0.186636 | 1 |
| 280_66 | A_23_P112596 | NM_001123 | ADK | -0.110137364 | 0.397291695 | 0.80129262 | 1 | -0.18980973 | 0.102692249 | 0.3480212 | 1 |
| 49_163 | A_23_P112596 | NM_001123 | ADK | -0.105827874 | 0.428383678 | 0.820934424 | 1 | -0.343243699 | 0.018921941 | 0.134099 | 1 |
| 121_94 | A_23_P112596 | NM_001123 | ADK | -0.036940553 | 0.821249129 | 0.957518252 | 1 | -0.229452268 | 0.116616675 | 0.3702958 | 1 |
| 271_63 | A_24_P134319 | NM_001282531 | ADNP | -0.125945166 | 0.335757962 | 0.762359625 | 1 | -0.362107803 | 0.021583822 | 0.143609 | 1 |
| 222_22 | A_23_P55477 | NM_000676 | ADORA2B | 0.168690798 | 0.321530783 | 0.753097268 | 1 | -0.15017876 | 0.245618426 | 0.5327303 | 1 |
| 29_96 | A_33_P3377194 | NM_033304 | ADRA1A | 0.106623763 | 0.621725562 | 0.89867047 | 1 | 0.04292791 | 0.843698181 | 0.9414469 | 1 |
| 22_49 | A_23_P33326 | NM_000679 | ADRA1B | 0.235819292 | 0.3448062 | 0.7684824 | 1 | -0.128029929 | 0.551239834 | 0.7881763 | 1 |
| 262_71 | A_23_P68665 | NM_007002 | ADRM1 | 0.19165266 | 0.187471448 | 0.630397286 | 1 | -0.33514366 | 0.066164973 | 0.2730853 | 1 |
| 90_87 | A_23_P155103 | NM_000026 | ADSL | 0.172354572 | 0.252242162 | 0.695994114 | 1 | -0.285462092 | 0.063789438 | 0.2667107 | 1 |
| 326_12 | A_23_P165061 | NM_198969 | AES | -0.390792295 | 0.046064381 | 0.396857348 | 1 | -0.232033242 | 0.099915648 | 0.3434127 | 1 |
| 345_13 | A_23_P102542 | NM_203437 | AFTPH | -0.198491303 | 0.123106712 | 0.547330642 | 1 | 0.013164689 | 0.916393192 | 0.9710598 | 1 |
| 30_43 | A_23_P428382 | NM_203437 | AFTPH | -0.139133219 | 0.287468409 | 0.72693776 | 1 | -0.014741512 | 0.91961431 | 0.9713137 | 1 |
| 311_155 | A_23_P7099 | NM_000027 | AGA | -0.036225094 | 0.77521282 | 0.946541303 | 1 | 0.325682048 | 0.014618903 | 0.1154099 | 1 |
| 292_51 | A_23_P111452 | NM_031946 | AGAP3 | -0.063312706 | 0.749430793 | 0.941597731 | 1 | 0.046731694 | 0.778996945 | 0.9124535 | 1 |
| 125_147 | A_33_P3240543 | NM_001042535 | AGAP3 | 0.510182714 | 0.04792155 | 0.400534962 | 1 | -0.601963466 | 0.001282886 | 0.0259848 | 1 |
| 48_17 | A_33_P3227666 | NM_018046 | AGGF1 | -0.346914996 | 0.037798815 | 0.371467292 | 1 | 0.173741091 | 0.286058531 | 0.5772057 | 1 |
| 298_38 | A_23_P200298 | NM_000028 | AGL | -0.052452662 | 0.679270317 | 0.919351731 | 1 | 0.144915969 | 0.23112902 | 0.5195539 | 1 |
| 300_82 | A_23_P103720 | NM_024758 | AGMAT | 0.152221637 | 0.346725768 | 0.768817672 | 1 | -0.351614354 | 0.007817988 | 0.0800081 | 1 |
| 107_149 | A_23_P103720 | NM_024758 | AGMAT | 0.165738136 | 0.242175379 | 0.688329554 | 1 | -0.399270681 | 0.005371976 | 0.0633319 | 1 |
| 30_33 | A_23_P103720 | NM_024758 | AGMAT | 0.180381592 | 0.224963447 | 0.669395387 | 1 | -0.382049156 | 0.01043097 | 0.0938499 | 1 |
| 277_153 | A_23_P103720 | NM_024758 | AGMAT | 0.209203779 | 0.248504199 | 0.694011498 | 1 | -0.437783064 | 0.003869429 | 0.0513485 | 1 |
| 275_156 | A_23_P103720 | NM_024758 | AGMAT | 0.220705502 | 0.211280637 | 0.657798949 | 1 | -0.45111285 | 0.003643521 | 0.049551 | 1 |
| 71_67 | A_23_P103720 | NM_024758 | AGMAT | 0.225444639 | 0.18171283 | 0.622415463 | 1 | -0.337721902 | 0.008704737 | 0.0851323 | 1 |
| 173_57 | A_23_P103720 | NM_024758 | AGMAT | 0.246009607 | 0.102878518 | 0.514394138 | 1 | -0.329839292 | 0.015747467 | 0.1205662 | 1 |
| 217_159 | A_23_P103720 | NM_024758 | AGMAT | 0.310823945 | 0.060859759 | 0.430408929 | 1 | -0.454550228 | 0.003552183 | 0.0490144 | 1 |
| 136_88 | A_23_P103720 | NM_024758 | AGMAT | 0.32837451 | 0.04282982 | 0.390076378 | 1 | -0.39009224 | 0.003841956 | 0.0513485 | 1 |
| 354_100 | A_23_P103720 | NM_024758 | AGMAT | 0.337292077 | 0.028218992 | 0.333724089 | 1 | -0.412173817 | 0.004011271 | 0.0525158 | 1 |
| 82_161 | A_33_P3402116 | NM_012199 | AGO1 | -0.01124709 | 0.948662683 | 0.990231079 | 1 | 0.344181076 | 0.046659049 | 0.2236296 | 1 |
| 284_62 | A_33_P3380587 | NM_012199 | AGO1 | 0.049247489 | 0.723979121 | 0.936348816 | 1 | 0.174919522 | 0.167856098 | 0.4448345 | 1 |
| 72_14 | A_23_P112159 | NM_012154 | AGO2 | -0.105731294 | 0.469351131 | 0.836960042 | 1 | 0.083908024 | 0.50485165 | 0.7573621 | 1 |
| 350_9 | A_23_P112159 | NM_012154 | AGO2 | 0.020376974 | 0.886130613 | 0.973024604 | 1 | -0.082583855 | 0.486998061 | 0.7440928 | 1 |
| 103_42 | A_23_P112159 | NM_012154 | AGO2 | 0.030240013 | 0.816096339 | 0.956101362 | 1 | -0.037851497 | 0.72882644 | 0.8897331 | 1 |
| 81_43 | A_23_P112159 | NM_012154 | AGO2 | 0.032079334 | 0.813739446 | 0.955007122 | 1 | 0.082242576 | 0.450471148 | 0.7182691 | 1 |
| 116_44 | A_23_P112159 | NM_012154 | AGO2 | 0.064302335 | 0.630836782 | 0.901004617 | 1 | 0.09803146 | 0.372838675 | 0.6556459 | 1 |
| 30_27 | A_23_P112159 | NM_012154 | AGO2 | 0.076434349 | 0.563593612 | 0.879316217 | 1 | 0.202243974 | 0.138164871 | 0.4035245 | 1 |
| 16_145 | A_23_P112159 | NM_012154 | AGO2 | 0.132551767 | 0.375883616 | 0.789123966 | 1 | -0.026929648 | 0.820795603 | 0.9311769 | 1 |
| 353_116 | A_23_P112159 | NM_012154 | AGO2 | 0.15956596 | 0.258584034 | 0.700376544 | 1 | -0.072768999 | 0.526444391 | 0.7702046 | 1 |
| 168_81 | A_23_P112159 | NM_012154 | AGO2 | 0.195996753 | 0.128729621 | 0.557016968 | 1 | -0.042755719 | 0.727444256 | 0.8888613 | 1 |
| 127_107 | A_23_P112159 | NM_012154 | AGO2 | 0.241545483 | 0.104846693 | 0.518151082 | 1 | -0.086860421 | 0.448280645 | 0.7166534 | 1 |
| 356_3 | A_33_P3244882 | NM_024852 | AGO3 | -0.030946432 | 0.799141828 | 0.951548003 | 1 | 0.177029783 | 0.179349957 | 0.4577014 | 1 |
| 355_29 | A_23_P97021 | NM_024852 | AGO3 | 0.031428634 | 0.812236492 | 0.954976822 | 1 | 0.244711478 | 0.090826161 | 0.3253524 | 1 |
| 62_73 | A_23_P258093 | NM_006411 | AGPAT1 | -0.100059589 | 0.566558502 | 0.880639801 | 1 | 0.032778587 | 0.797472397 | 0.9204689 | 1 |
| 128_18 | A_33_P3360540 | NM_006412 | AGPAT2 | -0.209391897 | 0.132454105 | 0.561823086 | 1 | -0.064436339 | 0.570286886 | 0.8009511 | 1 |
| 270_58 | A_24_P344711 | NM_020132 | AGPAT3 | 0.062959981 | 0.612975517 | 0.897816866 | 1 | -0.204763005 | 0.119636609 | 0.3753821 | 1 |
| 358_4 | A_33_P3217218 | NM_018361 | AGPAT5 | -0.098138036 | 0.495041395 | 0.849990629 | 1 | -0.512016234 | 0.006063523 | 0.068237 | 1 |
| 292_73 | A_23_P368645 | NM_003659 | AGPS | -0.157894069 | 0.306796452 | 0.739887567 | 1 | 0.629436446 | 0.000158507 | 0.0067784 | 1 |
| 224_101 | A_32_P27706 | NM_003659 | AGPS | -0.010528832 | 0.945920721 | 0.99002169 | 1 | 0.017930136 | 0.942931694 | 0.9799726 | 1 |
| 111_127 | A_22_P00000867 | NM_198576 | AGRN | -0.369578454 | 0.017066197 | 0.29259326 | 1 | 0.207782941 | 0.102429972 | 0.3475203 | 1 |
| 160_15 | A_23_P343411 | NM_198576 | AGRN | -0.144132109 | 0.281530748 | 0.722517117 | 1 | -0.088310458 | 0.444661375 | 0.7140135 | 1 |
| 159_159 | A_23_P368126 | NM_006621 | AHCYL1 | 0.187846604 | 0.230237082 | 0.676515722 | 1 | -0.524131807 | 0.001404425 | 0.0271869 | 1 |
| 304_127 | A_23_P115331 | NM_001029882 | AHDC1 | -0.09389581 | 0.491255828 | 0.847539796 | 1 | -0.108286534 | 0.359332574 | 0.6439473 | 1 |
| 294_28 | A_23_P21363 | NM_024060 | AHNAK | -0.180828728 | 0.214916561 | 0.661348172 | 1 | -0.386772717 | 0.008586932 | 0.0846426 | 1 |
| 39_143 | A_24_P943393 | NM_001620 | AHNAK | 0.015339519 | 0.961484706 | 0.993286853 | 1 | 0.385930906 | 0.013601943 | 0.1106954 | 1 |
| 213_56 | A_23_P117599 | NM_012111 | AHSA1 | 0.160114033 | 0.272609615 | 0.714985188 | 1 | 0.102556001 | 0.525253054 | 0.7698099 | 1 |
| 168_44 | A_23_P117599 | NM_012111 | AHSA1 | 0.191667667 | 0.177033798 | 0.616374573 | 1 | 0.017876369 | 0.910271692 | 0.9684822 | 1 |
| 342_90 | A_23_P117599 | NM_012111 | AHSA1 | 0.196583644 | 0.196592319 | 0.642839304 | 1 | -0.042472808 | 0.751652128 | 0.9007747 | 1 |
| 106_88 | A_23_P372467 | NM_152392 | AHSA2 | -0.134982524 | 0.365671028 | 0.781583876 | 1 | 0.080130573 | 0.552028209 | 0.788426 | 1 |
| 190_69 | A_23_P392384 | NM_001185095 | AIF1L | -0.541986744 | 0.011164918 | 0.256586468 | 1 | 0.311761337 | 0.044279183 | 0.2174707 | 1 |
| 18_19 | A_23_P72537 | NM_004208 | AIFM1 | -0.041010295 | 0.744553332 | 0.941128659 | 1 | 0.535881379 | 0.000744249 | 0.0188103 | 1 |
| 187_111 | A_32_P75094 | NM_032797 | AIFM2 | 0.016730458 | 0.907899418 | 0.978414074 | 1 | -0.289021686 | 0.164021841 | 0.4394363 | 1 |
| 278_14 | A_23_P93431 | NM_016108 | AIG1 | -0.468208353 | 0.003206188 | 0.170557997 | 1 | -0.125221877 | 0.377954148 | 0.6599674 | 1 |
| 155_81 | A_33_P3416568 | THC2526086 | AIG1 | 0.092730195 | 0.522288741 | 0.862597809 | 1 | -0.078853361 | 0.573982996 | 0.8035333 | 1 |
| 250_5 | A_23_P121686 | NM_004757 | AIMP1 | -0.098776746 | 0.465625083 | 0.836223448 | 1 | -0.036735173 | 0.769508776 | 0.9081774 | 1 |
| 374_3 | A_23_P121686 | NM_004757 | AIMP1 | 0.005854414 | 0.968503307 | 0.994075478 | 1 | 0.020437179 | 0.881881533 | 0.9592381 | 1 |
| 357_37 | A_23_P121686 | NM_004757 | AIMP1 | 0.057952193 | 0.705027498 | 0.927376665 | 1 | 0.013257362 | 0.916457052 | 0.9710598 | 1 |
| 365_60 | A_23_P121686 | NM_004757 | AIMP1 | 0.094187756 | 0.552846211 | 0.875569848 | 1 | 0.029321312 | 0.826813694 | 0.9338627 | 1 |
| 375_56 | A_23_P121686 | NM_004757 | AIMP1 | 0.133663715 | 0.416419315 | 0.814227569 | 1 | -0.022123765 | 0.858369768 | 0.9492411 | 1 |
| 147_155 | A_23_P121686 | NM_004757 | AIMP1 | 0.237718298 | 0.084791804 | 0.478632531 | 1 | -0.132322755 | 0.319178544 | 0.6059183 | 1 |
| 37_88 | A_23_P121686 | NM_004757 | AIMP1 | 0.253788095 | 0.066323531 | 0.44515331 | 1 | -0.075519421 | 0.549993473 | 0.7874341 | 1 |
| 7_90 | A_23_P121686 | NM_004757 | AIMP1 | 0.288327992 | 0.074673084 | 0.460772354 | 1 | 0.10086855 | 0.441337961 | 0.7115701 | 1 |
| 51_122 | A_23_P121686 | NM_004757 | AIMP1 | 0.304729876 | 0.106284478 | 0.521088485 | 1 | -0.090090489 | 0.484090198 | 0.7419754 | 1 |
| 163_61 | A_23_P121686 | NM_004757 | AIMP1 | 0.420355036 | 0.008424764 | 0.234464792 | 1 | -0.021704234 | 0.885699269 | 0.9608259 | 1 |
| 65_88 | A_23_P70991 | NM_006303 | AIMP2 | 0.285616578 | 0.077256319 | 0.464579439 | 1 | -0.490922365 | 0.002210813 | 0.0357256 | 1 |
| 173_125 | A_23_P75380 | NM_003977 | AIP | 0.146617124 | 0.357958962 | 0.775503482 | 1 | -0.205503241 | 0.193211404 | 0.475191 | 1 |
| 230_23 | A_23_P54055 | NM_032876 | AJUBA | 0.202576712 | 0.198333191 | 0.644907571 | 1 | -0.064427415 | 0.605599205 | 0.8203033 | 1 |
| 314_31 | A_23_P217088 | NM_000476 | AK1 | -0.097648069 | 0.481787432 | 0.842298291 | 1 | -0.286337441 | 0.122042068 | 0.3793751 | 1 |
| 143_36 | A_24_P500891 | NM_013411 | AK2 | -0.12028669 | 0.401287182 | 0.804688139 | 1 | 0.03731568 | 0.740410334 | 0.8949226 | 1 |
| 252_144 | A_33_P3392580 | NM_013411 | AK2 | 0.001411824 | 0.99453879 | 0.998639041 | 1 | -0.260148119 | 0.222813862 | 0.5108006 | 1 |
| 342_55 | A_24_P179903 | NM_001625 | AK2 | 0.107036546 | 0.479283528 | 0.841586126 | 1 | 0.409575785 | 0.010112167 | 0.0921649 | 1 |
| 138_145 | A_23_P200404 | NM_001625 | AK2 | 0.25790708 | 0.157788936 | 0.595969916 | 1 | 0.123215024 | 0.425338536 | 0.6994857 | 1 |
| 241_156 | A_33_P3263061 | NM_016282 | AK3 | 0.099665409 | 0.519816352 | 0.861706872 | 1 | -0.425584039 | 0.005954926 | 0.0676516 | 1 |
| 185_142 | A_32_P108655 | NM_001005353 | AK4 | -0.14139737 | 0.328773578 | 0.759416455 | 1 | 0.140802363 | 0.362585541 | 0.6470961 | 1 |
| 277_93 | A_23_P21734 | NM_001015891 | AK6 | 0.24708928 | 0.200462677 | 0.647411539 | 1 | -0.248967535 | 0.094405219 | 0.3330538 | 1 |
| 244_107 | A_32_P132438 | NM_003488 | AKAP1 | 0.06537338 | 0.646906419 | 0.907045535 | 1 | -0.211562629 | 0.16570621 | 0.4417392 | 1 |
| 294_29 | A_23_P342668 | NM_005088 | AKAP17A | -0.221883874 | 0.111841051 | 0.52983507 | 1 | -0.546737322 | 0.001693846 | 0.0303355 | 1 |
| 1_144 | A_33_P3417950 | NM_005858 | AKAP8 | 0.072827794 | 0.64325844 | 0.906600677 | 1 | -0.164395103 | 0.233874726 | 0.5214887 | 1 |
| 167_40 | A_23_P309261 | NM_005751 | AKAP9 | 0.037547521 | 0.793044018 | 0.94877096 | 1 | 0.255671507 | 0.034796114 | 0.1917951 | 1 |
| 225_134 | A_23_P53152 | NM_020642 | AKIP1 | 0.185161358 | 0.157871854 | 0.595969916 | 1 | -0.20774631 | 0.102742542 | 0.3480558 | 1 |
| 166_22 | A_33_P3354267 | NM_024595 | AKIRIN1 | 0.16933008 | 0.227079446 | 0.672406856 | 1 | 0.007299765 | 0.95422577 | 0.9841389 | 1 |
| 296_161 | A_23_P138541 | NM_003739 | AKR1C3 | 0.902908825 | 0.000292834 | 0.059491419 | 1 | -1.32243188 | 6.16798E-06 | 0.0013178 | 0.06937 |
| 344_99 | A_23_P115356 | NM_003689 | AKR7A2 | 0.049569262 | 0.730645457 | 0.936838487 | 1 | 0.005097374 | 0.970641761 | 0.98964 | 1 |
| 256_60 | A_23_P115356 | NM_003689 | AKR7A2 | 0.092534088 | 0.456858231 | 0.83358833 | 1 | -0.069972877 | 0.672947826 | 0.8624474 | 1 |
| 177_49 | A_23_P115356 | NM_003689 | AKR7A2 | 0.113077549 | 0.412084444 | 0.812009855 | 1 | -0.074660857 | 0.647740856 | 0.8466172 | 1 |
| 181_39 | A_23_P115356 | NM_003689 | AKR7A2 | 0.116175802 | 0.36664624 | 0.781989605 | 1 | 0.028845513 | 0.832558488 | 0.9366595 | 1 |
| 14_89 | A_23_P115356 | NM_003689 | AKR7A2 | 0.160911214 | 0.209919468 | 0.656207072 | 1 | -0.134881894 | 0.361983795 | 0.6467032 | 1 |
| 311_85 | A_23_P115356 | NM_003689 | AKR7A2 | 0.169173106 | 0.264948594 | 0.707490253 | 1 | -0.065127729 | 0.674694891 | 0.8639751 | 1 |
| 99_20 | A_23_P115356 | NM_003689 | AKR7A2 | 0.179554949 | 0.186340738 | 0.629507173 | 1 | -0.056730681 | 0.703997666 | 0.8766152 | 1 |
| 378_80 | A_23_P115356 | NM_003689 | AKR7A2 | 0.18664965 | 0.198293874 | 0.644907571 | 1 | -0.089929369 | 0.496788018 | 0.7519008 | 1 |
| 264_145 | A_23_P115356 | NM_003689 | AKR7A2 | 0.216915298 | 0.121421892 | 0.543714677 | 1 | -0.159414853 | 0.199056127 | 0.4821849 | 1 |
| 24_114 | A_23_P115356 | NM_003689 | AKR7A2 | 0.220449441 | 0.091634559 | 0.49154825 | 1 | -0.05076313 | 0.649323434 | 0.8474567 | 1 |
| 106_68 | A_33_P3217998 | NR_040288 | AKR7L | 0.111827154 | 0.448586592 | 0.830710542 | 1 | -0.180860628 | 0.292501333 | 0.5845349 | 1 |
| 105_49 | A_33_P3275235 | NM_005163 | AKT1 | -0.053508139 | 0.693440249 | 0.924234842 | 1 | 0.087933363 | 0.468074626 | 0.7297542 | 1 |
| 153_12 | A_23_P208870 | NM_001626 | AKT2 | -0.036210183 | 0.76770231 | 0.945351992 | 1 | -0.060507157 | 0.61808188 | 0.8268784 | 1 |
| 226_98 | A_23_P1361 | NM_002860 | ALDH18A1 | 0.203372974 | 0.128653896 | 0.556951584 | 1 | -0.002025518 | 0.988137166 | 0.9961979 | 1 |
| 320_47 | A_23_P36753 | NM_000690 | ALDH2 | 0.092649834 | 0.555438598 | 0.87578993 | 1 | -0.851365689 | 0.000192333 | 0.0076168 | 1 |
| 27_82 | A_33_P3336622 | NM_001031806 | ALDH3A2 | -0.13746179 | 0.353475387 | 0.772780895 | 1 | -0.131377006 | 0.371659717 | 0.6550662 | 1 |
| 214_57 | A_23_P170337 | NM_003748 | ALDH4A1 | 0.068448522 | 0.575443426 | 0.883311119 | 1 | 0.13843843 | 0.393939148 | 0.6741782 | 1 |
| 236_47 | A_23_P70231 | NM_001182 | ALDH7A1 | -0.22080212 | 0.16463513 | 0.60427748 | 1 | 0.179565774 | 0.178201757 | 0.4568562 | 1 |
| 149_44 | A_24_P385280 | NM_000696 | ALDH9A1 | -0.121695998 | 0.413427108 | 0.812055632 | 1 | -0.090862191 | 0.429544921 | 0.7028065 | 1 |
| 363_135 | A_23_P88963 | NM_000034 | ALDOA | 0.351033917 | 0.084343291 | 0.478632531 | 1 | -0.44060671 | 0.103351786 | 0.3493831 | 1 |
| 228_82 | A_32_P169131 | NM_019109 | ALG1 | -0.028585834 | 0.813391992 | 0.955007122 | 1 | 0.231887279 | 0.182796714 | 0.4617957 | 1 |
| 213_92 | A_23_P66306 | NM_019109 | ALG1 | 0.171300536 | 0.25046182 | 0.694859005 | 1 | 0.063258551 | 0.704441361 | 0.8766152 | 1 |
| 12_9 | A_33_P3230876 | NM_001004127 | ALG11 | -0.263646642 | 0.062972152 | 0.435656913 | 1 | -0.082491046 | 0.487721056 | 0.7447928 | 1 |
| 103_30 | A_23_P11279 | NM_018466 | ALG13 | 0.156826291 | 0.234816993 | 0.680918975 | 1 | 0.36064583 | 0.036219245 | 0.1954332 | 1 |
| 191_62 | A_23_P22672 | NM_001257231 | ALG13 | 0.169893592 | 0.253507823 | 0.697590309 | 1 | 0.546623558 | 0.000990374 | 0.0221446 | 1 |
| 353_45 | A_22_P00004279 | NM_144988 | ALG14 | -0.013317398 | 0.925446762 | 0.983923238 | 1 | -0.176623563 | 0.167015753 | 0.4438625 | 1 |
| 88_93 | A_33_P3297050 | NM_033087 | ALG2 | -0.122086597 | 0.409254154 | 0.810123176 | 1 | -0.081555291 | 0.586221115 | 0.8110447 | 1 |
| 226_88 | A_24_P414269 | NM_005787 | ALG3 | 0.108587171 | 0.384309697 | 0.793334649 | 1 | 0.24056725 | 0.147079081 | 0.4160459 | 1 |
| 39_10 | A_23_P151436 | NM_013338 | ALG5 | 0.522506251 | 0.028335785 | 0.333836104 | 1 | -0.550185622 | 0.001300298 | 0.0259848 | 1 |
| 298_146 | A_23_P35168 | NM_013339 | ALG6 | -0.029258149 | 0.817299673 | 0.956101362 | 1 | -0.053171431 | 0.645902526 | 0.8458856 | 1 |
| 347_137 | A_23_P13554 | NM_001007027 | ALG8 | -0.043841878 | 0.72091727 | 0.935358583 | 1 | 0.28308351 | 0.071582629 | 0.2848867 | 1 |
| 99_37 | A_32_P28939 | NM_001001655 | ALKBH2 | 0.128626103 | 0.37452309 | 0.788281609 | 1 | 0.387529745 | 0.047530518 | 0.2263233 | 1 |
| 141_104 | A_24_P91472 | NM_032306 | ALKBH7 | -0.119426294 | 0.536747154 | 0.868231819 | 1 | -0.028602832 | 0.854346569 | 0.9472155 | 1 |
| 94_99 | A_23_P312174 | NM_015120 | ALMS1 | -0.378217883 | 0.026725021 | 0.329053972 | 1 | 0.31466176 | 0.018646345 | 0.1330682 | 1 |
| 62_93 | A_23_P144531 | AK026323 | ALPK1 | 0.062105922 | 0.74642741 | 0.941470372 | 1 | -0.054653797 | 0.661465065 | 0.8558032 | 1 |
| 59_21 | A_24_P131580 | NM_031313 | ALPPL2 | 0.000741605 | 0.996355614 | 0.999144229 | 1 | 0.467876775 | 0.006562179 | 0.0716552 | 1 |
| 135_91 | A_23_P152984 | NM_005782 | ALYREF | 0.045242828 | 0.786090018 | 0.94877096 | 1 | -0.029259849 | 0.879770575 | 0.9583932 | 1 |
| 210_158 | A_23_P353742 | NM_017749 | AMBRA1 | -0.076712702 | 0.588733096 | 0.88673919 | 1 | -0.189958232 | 0.133871455 | 0.3972612 | 1 |
| 286_73 | A_24_P371962 | NM_001634 | AMD1 | 0.039002581 | 0.81576246 | 0.955999835 | 1 | 0.085223933 | 0.511910164 | 0.7606193 | 1 |
| 47_6 | A_33_P3491294 | NM_015944 | AMDHD2 | -0.187020788 | 0.247021446 | 0.692251037 | 1 | 0.276272209 | 0.078688163 | 0.3023184 | 1 |
| 328_14 | A_23_P308150 | NM_152424 | AMER1 | -0.152200126 | 0.367387475 | 0.781989605 | 1 | 0.271260688 | 0.068929771 | 0.2790688 | 1 |
| 149_2 | A_24_P135444 | NM_001144 | AMFR | 0.053295751 | 0.700890106 | 0.925755532 | 1 | -0.086497408 | 0.476389037 | 0.7357797 | 1 |
| 18_114 | A_23_P14083 | NM_181847 | AMIGO2 | -0.004263763 | 0.977044753 | 0.995182187 | 1 | 0.6853546 | 0.000280477 | 0.0098272 | 1 |
| 257_75 | A_32_P201773 | NM_015365 | AMMECR1 | 0.0536264 | 0.674364707 | 0.918166391 | 1 | 0.466483274 | 0.000998222 | 0.0222317 | 1 |
| 51_84 | A_33_P3234347 | NM_001171689 | AMMECR1 | 0.092160277 | 0.499072021 | 0.851671485 | 1 | 0.109753637 | 0.369516378 | 0.6534271 | 1 |
| 74_121 | A_23_P40039 | NM_031445 | AMMECR1L | 0.007647672 | 0.960895631 | 0.993286853 | 1 | -0.040665128 | 0.753509989 | 0.9012104 | 1 |
| 176_69 | A_23_P48455 | NM_030943 | AMN | -0.017629526 | 0.894079347 | 0.975081715 | 1 | 0.065232136 | 0.601251026 | 0.8185598 | 1 |
| 352_135 | A_23_P166686 | NM_016201 | AMOTL2 | -0.007895706 | 0.955363304 | 0.991314091 | 1 | 0.423666021 | 0.009363572 | 0.0878458 | 1 |
| 23_105 | A_33_P3380837 | NM_133463 | AMZ1 | -0.075853138 | 0.531070285 | 0.865842607 | 1 | 0.099963155 | 0.506369539 | 0.7577939 | 1 |
| 180_33 | A_23_P15564 | NM_016627 | AMZ2 | -0.042924045 | 0.756487919 | 0.942463951 | 1 | 0.339579265 | 0.009713999 | 0.0902175 | 1 |
| 239_12 | A_21_P0011603 | NM_016627 | AMZ2 | 0.035934186 | 0.770977341 | 0.945351992 | 1 | -0.034732937 | 0.769936108 | 0.9081774 | 1 |
| 98_102 | A_33_P3223472 | NM_016627 | AMZ2 | 0.113433722 | 0.495824899 | 0.850005476 | 1 | -0.017608516 | 0.891150888 | 0.9619458 | 1 |
| 250_8 | A_23_P17204 | NM_022662 | ANAPC1 | 0.121879345 | 0.40434117 | 0.806612891 | 1 | -0.039111065 | 0.774819315 | 0.9099293 | 1 |
| 271_150 | A_24_P186346 | NM_022662 | ANAPC1 | 0.143816513 | 0.31421755 | 0.745268067 | 1 | -0.099411751 | 0.437068773 | 0.7087244 | 1 |
| 46_78 | A_23_P250994 | NM_014885 | ANAPC10 | 0.382620806 | 0.021782712 | 0.309199145 | 1 | -0.161630845 | 0.229457859 | 0.5173842 | 1 |
| 368_126 | A_23_P27147 | NM_001002244 | ANAPC11 | -0.235696523 | 0.085274763 | 0.478632531 | 1 | 0.096178152 | 0.398554312 | 0.6770186 | 1 |
| 330_107 | A_32_P16854 | NM_015391 | ANAPC13 | 0.029247051 | 0.811162557 | 0.954780472 | 1 | 0.128190771 | 0.252860409 | 0.5409211 | 1 |
| 347_154 | A_24_P244410 | NM_014042 | ANAPC15 | -0.152777005 | 0.342727804 | 0.767452043 | 1 | -0.685106776 | 0.000196054 | 0.0077099 | 1 |
| 370_66 | A_23_P317800 | NM_013367 | ANAPC4 | -0.105905842 | 0.472008376 | 0.838682328 | 1 | -0.044346949 | 0.726831779 | 0.888579 | 1 |
| 34_119 | A_23_P204448 | NM_016237 | ANAPC5 | 0.086287212 | 0.611870323 | 0.897508041 | 1 | 0.079100214 | 0.609474425 | 0.8220121 | 1 |
| 333_123 | A_23_P92642 | NM_024668 | ANKHD1 | 0.141470698 | 0.403943351 | 0.806298198 | 1 | -0.48774164 | 0.002054319 | 0.0342803 | 1 |
| 110_142 | A_23_P58443 | NM_020690 | ANKHD1-EIF4EBP3 | -0.146869747 | 0.308932032 | 0.741558623 | 1 | -0.019861899 | 0.872890338 | 0.9558366 | 1 |
| 222_132 | A_33_P3315554 | NM_001282771 | ANKMY1 | -0.052448907 | 0.678608415 | 0.919181603 | 1 | 0.497041965 | 0.035326597 | 0.1930482 | 1 |
| 167_23 | A_24_P324405 | NM_013275 | ANKRD11 | -0.390432366 | 0.079153191 | 0.468212318 | 1 | 0.132836785 | 0.262669472 | 0.5531255 | 1 |
| 178_31 | A_33_P3280044 | NM_001256182 | ANKRD11 | -0.349558452 | 0.205571884 | 0.652325265 | 1 | 0.270826583 | 0.218165064 | 0.5048757 | 1 |
| 222_37 | A_24_P179183 | NM_015208 | ANKRD12 | -0.293726963 | 0.050442372 | 0.406166182 | 1 | 0.006383893 | 0.956982663 | 0.9850131 | 1 |
| 188_161 | A_33_P3235189 | NM_152345 | ANKRD13B | 0.021843929 | 0.906205372 | 0.978062713 | 1 | -0.034143159 | 0.848541067 | 0.9444119 | 1 |
| 53_68 | A_33_P3228285 | NM_207354 | ANKRD13D | -0.178024317 | 0.215847579 | 0.662310915 | 1 | 0.019732499 | 0.857545833 | 0.9491063 | 1 |
| 261_145 | A_33_P3267502 | NM_001009941 | ANKRD16 | -0.119350893 | 0.414694702 | 0.812829671 | 1 | -0.317187313 | 0.051934849 | 0.2383881 | 1 |
| 232_83 | A_33_P3412538 | NM_032217 | ANKRD17 | -0.032863642 | 0.80399605 | 0.952380385 | 1 | 0.331100745 | 0.026644385 | 0.1633391 | 1 |
| 121_150 | A_24_P217365 | NM_015199 | ANKRD28 | 0.125470946 | 0.410348167 | 0.811503469 | 1 | -0.285984361 | 0.057159466 | 0.2523048 | 1 |
| 290_106 | A_32_P516342 | NM_182608 | ANKRD33 | 0.035445245 | 0.822830789 | 0.957894143 | 1 | -0.014796035 | 0.926532052 | 0.973352 | 1 |
| 376_159 | A_21_P0011808 | NM_001164315 | ANKRD36 | -0.047565544 | 0.69760324 | 0.925106933 | 1 | -0.194319613 | 0.102701366 | 0.3480212 | 1 |
| 264_98 | A_33_P3421418 | ENST00000456556 | ANKRD36C | -0.094759537 | 0.474905893 | 0.839626091 | 1 | -0.046339301 | 0.700230827 | 0.8751652 | 1 |
| 113_98 | A_24_P237586 | NM_181726 | ANKRD37 | 0.040190913 | 0.773771302 | 0.946321166 | 1 | 0.107806802 | 0.478725536 | 0.7373874 | 1 |
| 364_91 | A_33_P3294053 | NM_016466 | ANKRD39 | -0.230573646 | 0.077081742 | 0.464396529 | 1 | 0.037966194 | 0.771848696 | 0.9087179 | 1 |
| 87_8 | A_33_P3278144 | NM_001300977 | ANKRD42 | 0.134260142 | 0.376010793 | 0.78918168 | 1 | -0.098992391 | 0.429246942 | 0.7025233 | 1 |
| 379_6 | A_33_P3219697 | NM_198493 | ANKRD45 | -0.284428341 | 0.070737977 | 0.455459851 | 1 | 0.109469015 | 0.379695149 | 0.6619797 | 1 |
| 133_51 | A_23_P24365 | NM_017704 | ANKRD49 | 0.145398658 | 0.346349749 | 0.768530536 | 1 | -0.025787253 | 0.82012131 | 0.9309091 | 1 |
| 1_66 | A_33_P3267305 | NM_173595 | ANKRD52 | -0.10565323 | 0.469960555 | 0.836960042 | 1 | -0.118834172 | 0.342498844 | 0.6288779 | 1 |
| 275_98 | A_21_P0000033 | NM_001162435 | ANKRD66 | 0.067617212 | 0.648824637 | 0.90775139 | 1 | 0.060069921 | 0.614767956 | 0.8247012 | 1 |
| 261_158 | A_33_P3263666 | NM_152326 | ANKRD9 | -0.138860666 | 0.322544203 | 0.753592919 | 1 | 0.20758019 | 0.286958487 | 0.5782902 | 1 |
| 288_44 | A_23_P156748 | NM_015245 | ANKS1A | -0.042919723 | 0.760694302 | 0.943087521 | 1 | -0.00251657 | 0.984350293 | 0.9949293 | 1 |
| 263_74 | A_33_P3211153 | NM_133450 | ANKS3 | -0.055486568 | 0.744561558 | 0.941128659 | 1 | -0.093406809 | 0.575044105 | 0.8040913 | 1 |
| 304_147 | A_23_P362183 | NM_173551 | ANKS6 | -0.116846971 | 0.41775542 | 0.815429664 | 1 | -0.306858053 | 0.035710797 | 0.1938086 | 1 |
| 18_67 | A_33_P3371224 | NM_001204831 | ANO10 | -0.062816238 | 0.604694278 | 0.894738941 | 1 | 0.452278368 | 0.003219769 | 0.0460028 | 1 |
| 184_100 | A_33_P3328426 | NM_001204831 | ANO10 | 0.007508254 | 0.95419522 | 0.991053178 | 1 | 0.564191964 | 0.005211582 | 0.0618951 | 1 |
| 184_49 | A_23_P389118 | NM_001025356 | ANO6 | 0.091657948 | 0.479765799 | 0.841905353 | 1 | -0.00440847 | 0.969048769 | 0.9889135 | 1 |
| 381_115 | A_23_P14649 | NR_026808 | ANP32A-IT1 | -0.116022695 | 0.454411346 | 0.832937561 | 1 | 0.022025469 | 0.871265457 | 0.9549871 | 1 |
| 328_98 | A_23_P20615 | NM_006401 | ANP32B | 0.011612473 | 0.946454511 | 0.99002169 | 1 | -0.167273246 | 0.352529639 | 0.6373774 | 1 |
| 113_74 | A_24_P225468 | NM_030920 | ANP32E | -0.038358364 | 0.755914895 | 0.942463951 | 1 | -0.259834794 | 0.042991409 | 0.2148043 | 1 |
| 24_94 | A_33_P3407299 | NM_030920 | ANP32E | 0.092033647 | 0.538015358 | 0.868480508 | 1 | -0.192831069 | 0.144704783 | 0.4122776 | 1 |
| 1_25 | A_33_P6455786 | NM_058172 | ANTXR2 | -0.062369174 | 0.628569123 | 0.900268731 | 1 | 0.096877304 | 0.466418338 | 0.7282809 | 1 |
| 352_89 | A_22_P00001320 | NM_001278688 | ANTXRL | 0.177847694 | 0.181253909 | 0.622415463 | 1 | -0.076683893 | 0.602979165 | 0.8190176 | 1 |
| 381_145 | A_23_P94501 | NM_000700 | ANXA1 | 0.303118909 | 0.038403281 | 0.372771925 | 1 | 1.049229616 | 4.2385E-05 | 0.0034531 | 0.4767 |
| 343_151 | A_23_P35399 | NM_145869 | ANXA11 | 0.050030092 | 0.817800136 | 0.956101362 | 1 | -0.69359777 | 0.009082006 | 0.0867845 | 1 |
| 236_8 | A_23_P146644 | NM_001002857 | ANXA2 | -0.015846064 | 0.922432611 | 0.983082909 | 1 | -0.398141102 | 0.032155932 | 0.1828401 | 1 |
| 90_53 | A_23_P121716 | NM_005139 | ANXA3 | 0.282986448 | 0.091855238 | 0.49164346 | 1 | -0.404098326 | 0.027341347 | 0.1661171 | 1 |
| 331_147 | A_23_P16976 | NM_001153 | ANXA4 | -0.068398321 | 0.605722651 | 0.894904051 | 1 | -0.169691457 | 0.160324807 | 0.4336636 | 1 |
| 292_14 | A_23_P69720 | NM_001154 | ANXA5 | 0.027342277 | 0.866423349 | 0.968594039 | 1 | -0.460515983 | 0.03643769 | 0.1961199 | 1 |
| 99_103 | A_33_P3329949 | NM_004034 | ANXA7 | -0.001681959 | 0.989656348 | 0.997022299 | 1 | 0.240415647 | 0.074939706 | 0.2927152 | 1 |
| 207_156 | A_33_P3317593 | NM_001127 | AP1B1 | -0.129665974 | 0.370556619 | 0.784043555 | 1 | -0.175268035 | 0.200062636 | 0.4832215 | 1 |
| 138_36 | A_24_P381962 | NM_001030007 | AP1G1 | 0.055759054 | 0.66581975 | 0.91515471 | 1 | -0.107422384 | 0.361672634 | 0.6467032 | 1 |
| 377_81 | A_33_P3275707 | NM_001039569 | AP1S3 | -0.165846768 | 0.230690965 | 0.677338474 | 1 | 0.532671725 | 0.001482789 | 0.027732 | 1 |
| 368_134 | A_24_P932418 | NM_012305 | AP2A2 | -0.351014628 | 0.031616794 | 0.345728535 | 1 | -0.514506038 | 0.002144936 | 0.0349625 | 1 |
| 299_160 | A_33_P3378800 | NM_004068 | AP2M1 | -0.183468462 | 0.177109474 | 0.616374573 | 1 | 0.021230328 | 0.858623787 | 0.9492411 | 1 |
| 337_136 | A_33_P3346663 | NM_001301078 | AP2S1 | -0.190049828 | 0.163846028 | 0.603672445 | 1 | -0.006146666 | 0.955616708 | 0.984413 | 1 |
| 206_57 | A_23_P136635 | NM_003664 | AP3B1 | 0.052702943 | 0.711053131 | 0.930633257 | 1 | -0.075761258 | 0.577318519 | 0.8049965 | 1 |
| 332_80 | A_33_P3571120 | NM_001261826 | AP3D1 | -0.168530466 | 0.217619327 | 0.663921522 | 1 | 0.336556725 | 0.01186092 | 0.1018319 | 1 |
| 185_53 | A_24_P64039 | NM_006803 | AP3M2 | 0.053822214 | 0.727128416 | 0.936348816 | 1 | -0.00589278 | 0.955529727 | 0.984413 | 1 |
| 383_43 | A_33_P3286254 | NM_001284 | AP3S1 | -0.331417887 | 0.03893609 | 0.374228433 | 1 | 0.355121242 | 0.075774062 | 0.295196 | 1 |
| 180_99 | A_23_P69958 | NM_001284 | AP3S1 | 0.131226066 | 0.396183008 | 0.800737669 | 1 | 0.484700231 | 0.019988749 | 0.137768 | 1 |
| 190_98 | A_24_P287691 | NM_005829 | AP3S2 | 0.352189903 | 0.022123733 | 0.311573782 | 1 | -0.140132881 | 0.334638721 | 0.6209473 | 1 |
| 3_64 | A_23_P160729 | NM_006594 | AP4B1 | 0.104835446 | 0.432293884 | 0.822997979 | 1 | -0.114257093 | 0.408240861 | 0.6851844 | 1 |
| 94_61 | A_24_P929369 | NM_007347 | AP4E1 | -0.052325471 | 0.673635629 | 0.918062293 | 1 | 0.088796054 | 0.506394787 | 0.7577939 | 1 |
| 172_84 | A_23_P37265 | NM_018229 | AP5M1 | 0.297804881 | 0.033507313 | 0.35402089 | 1 | 0.240377752 | 0.058288169 | 0.2548316 | 1 |
| 6_36 | A_23_P57137 | NM_018347 | AP5S1 | 0.057208626 | 0.666093994 | 0.915428366 | 1 | 0.02663753 | 0.809552523 | 0.9255909 | 1 |
| 382_19 | A_33_P3215487 | NM_014855 | AP5Z1 | -0.468949396 | 0.019606916 | 0.306116626 | 1 | -0.214305601 | 0.215260013 | 0.5015537 | 1 |
| 210_39 | A_23_P256682 | NM_014481 | APEX2 | 0.045338481 | 0.727461288 | 0.936348816 | 1 | -0.643623193 | 0.000107555 | 0.005771 | 1 |
| 181_30 | A_33_P3300395 | NM_199294 | APITD1 | -0.008044852 | 0.954792924 | 0.991077659 | 1 | -0.021543654 | 0.879336581 | 0.9582221 | 1 |
| 136_61 | A_23_P202939 | NM_001642 | APLP2 | 0.154977025 | 0.592037947 | 0.888531018 | 1 | 0.172040289 | 0.399419543 | 0.677591 | 1 |
| 111_87 | A_33_P3262181 | NM_001006666 | APOBEC3F | 0.009287979 | 0.950469153 | 0.99027625 | 1 | 0.335128421 | 0.04138967 | 0.2105426 | 1 |
| 163_131 | A_24_P109214 | NM_001645 | APOC1 | -0.373581879 | 0.013474387 | 0.270290692 | 1 | 0.71968781 | 0.000279248 | 0.0098147 | 1 |
| 222_53 | A_33_P3223592 | NM_001302688 | APOE | -0.115546682 | 0.347148163 | 0.768954722 | 1 | 0.569763705 | 0.002583126 | 0.0398267 | 1 |
| 158_65 | A_24_P87931 | NM_145343 | APOL1 | 0.008077506 | 0.952097432 | 0.990752085 | 1 | -0.024208842 | 0.81869019 | 0.9305516 | 1 |
| 129_145 | A_24_P48898 | NM_145637 | APOL2 | -0.181367069 | 0.210686615 | 0.656919463 | 1 | 0.091260134 | 0.521772819 | 0.7670191 | 1 |
| 350_66 | A_23_P61127 | NM_024122 | APOO | -0.202517792 | 0.163555678 | 0.603384225 | 1 | -0.965447018 | 0.000169615 | 0.0070606 | 1 |
| 265_12 | A_23_P136986 | NM_198450 | APOOL | 0.009788606 | 0.934643614 | 0.986418035 | 1 | 0.18444943 | 0.226681257 | 0.5148774 | 1 |
| 288_126 | A_23_P162879 | NM_032374 | APOPT1 | 0.234788244 | 0.143632711 | 0.57813643 | 1 | -0.194330952 | 0.128944502 | 0.3900476 | 1 |
| 216_99 | A_33_P3508822 | NM_000484 | APP | 0.178236616 | 0.343906518 | 0.767452043 | 1 | 0.314175902 | 0.063439768 | 0.2664375 | 1 |
| 267_109 | A_23_P207280 | NM_006380 | APPBP2 | -0.026794564 | 0.839659426 | 0.96243236 | 1 | 0.076820344 | 0.592929767 | 0.8148067 | 1 |
| 286_14 | A_23_P166663 | NM_012096 | APPL1 | -0.457947265 | 0.008118713 | 0.234464792 | 1 | -0.112576662 | 0.396463125 | 0.6759357 | 1 |
| 127_81 | A_33_P3360097 | NM_000485 | APRT | 0.07639356 | 0.543348799 | 0.870823107 | 1 | -0.052500686 | 0.695564479 | 0.873377 | 1 |
| 183_163 | A_19_P00811196 | NR_038361 | APTR | 0.125694978 | 0.387148657 | 0.795587877 | 1 | -0.021191119 | 0.860380359 | 0.9500931 | 1 |
| 165_52 | A_19_P00316423 | AI421806 | APTX | 0.014272635 | 0.920441184 | 0.981873714 | 1 | 1.289708746 | 2.10041E-07 | 0.0002625 | 0.00236 |
| 38_79 | A_33_P3344308 | NM_001195249 | APTX | 0.096920264 | 0.480120642 | 0.841921645 | 1 | -0.003247737 | 0.976924398 | 0.9917383 | 1 |
| 111_95 | A_23_P252775 | NM_175073 | APTX | 0.198854641 | 0.137801331 | 0.569705352 | 1 | 0.00848602 | 0.949675658 | 0.9823418 | 1 |
| 241_78 | A_24_P316305 | ENST00000156471 | AQR | -0.150236124 | 0.234477101 | 0.680743575 | 1 | -0.088222154 | 0.433668534 | 0.7071246 | 1 |
| 96_32 | A_33_P3362353 | NM_001256197 | ARAF | 0.039387035 | 0.751792008 | 0.942109358 | 1 | -0.309686027 | 0.019089455 | 0.1344516 | 1 |
| 363_30 | A_23_P167389 | NM_022481 | ARAP3 | -0.628251366 | 0.000456124 | 0.074522943 | 1 | 0.11496201 | 0.402184656 | 0.6801754 | 1 |
| 296_158 | A_23_P259071 | NM_001657 | AREG | 0.540941422 | 0.026502199 | 0.327952685 | 1 | 0.931622086 | 6.14861E-06 | 0.0013178 | 0.06915 |
| 117_69 | A_24_P256307 | NM_001659 | ARF3 | 0.010006227 | 0.954468836 | 0.991053178 | 1 | -0.386524003 | 0.129246125 | 0.3904462 | 1 |
| 352_23 | A_23_P431789 | NM_001660 | ARF4 | 0.144048553 | 0.336296598 | 0.762411026 | 1 | -0.15502508 | 0.268845516 | 0.5592206 | 1 |
| 277_14 | A_33_P3260777 | NM_001662 | ARF5 | -0.53908216 | 0.00484389 | 0.196694236 | 1 | 0.314606601 | 0.104308678 | 0.3510352 | 1 |
| 295_63 | A_23_P210379 | NM_175609 | ARFGAP1 | -0.085024294 | 0.505092511 | 0.85467065 | 1 | -0.237406417 | 0.069203311 | 0.2798267 | 1 |
| 159_6 | A_33_P3272483 | NM_032389 | ARFGAP2 | -0.010871196 | 0.945229568 | 0.989845255 | 1 | -0.286566974 | 0.037113909 | 0.1978294 | 1 |
| 375_156 | A_23_P68970 | NM_014570 | ARFGAP3 | -0.038441531 | 0.753756932 | 0.942109358 | 1 | -0.137836547 | 0.222730564 | 0.5108006 | 1 |
| 147_64 | A_24_P166094 | NM_001025595 | ARFIP1 | 0.133363809 | 0.329480053 | 0.760244507 | 1 | -0.260970021 | 0.034995008 | 0.1921821 | 1 |
| 291_140 | A_23_P139228 | NM_012402 | ARFIP2 | 0.075903554 | 0.553579052 | 0.875569848 | 1 | -0.179835812 | 0.169203642 | 0.4463024 | 1 |
| 309_74 | A_33_P3242748 | NM_001267549 | ARFRP1 | 0.061603696 | 0.656048793 | 0.910663902 | 1 | -0.701573801 | 0.001204855 | 0.0252346 | 1 |
| 281_88 | A_33_P3242743 | NM_001267549 | ARFRP1 | 0.084203304 | 0.51584267 | 0.860004647 | 1 | -0.22221449 | 0.248252506 | 0.5355889 | 1 |
| 261_127 | A_23_P128728 | NM_001172 | ARG2 | 0.568780792 | 0.001880441 | 0.138833508 | 1 | -0.531529894 | 0.000938972 | 0.0215057 | 1 |
| 108_27 | A_23_P162782 | NM_018011 | ARGLU1 | -0.233576593 | 0.123229243 | 0.547475797 | 1 | 0.034621677 | 0.802716609 | 0.9228701 | 1 |
| 347_77 | A_23_P314070 | NM_004308 | ARHGAP1 | -0.007640055 | 0.950678873 | 0.99027625 | 1 | 0.106478247 | 0.436029834 | 0.7081054 | 1 |
| 24_164 | A_23_P149775 | NM_018287 | ARHGAP12 | -0.129705472 | 0.347305433 | 0.769110122 | 1 | 0.000503791 | 0.996481868 | 0.9977238 | 1 |
| 347_134 | A_23_P75310 | NM_021226 | ARHGAP22 | 0.254637332 | 0.160672613 | 0.599483544 | 1 | 0.405553109 | 0.029049071 | 0.1717744 | 1 |
| 366_155 | A_33_P3228558 | NM_001282290 | ARHGAP27 | -0.185047562 | 0.236895437 | 0.683043837 | 1 | 0.043485993 | 0.710360605 | 0.8795214 | 1 |
| 131_40 | A_33_P3313411 | NM_001172630 | ARHGAP33 | -0.12116192 | 0.379362042 | 0.790179752 | 1 | 0.08398226 | 0.585048233 | 0.8105282 | 1 |
| 261_101 | A_33_P3282181 | NM_001164741 | ARHGAP4 | 0.126803087 | 0.488391887 | 0.846195759 | 1 | 1.018191599 | 8.51773E-05 | 0.0050687 | 0.95799 |
| 34_17 | A_33_P3299110 | NM_152432 | ARHGAP42 | 0.500828364 | 0.122680411 | 0.546432456 | 1 | 0.051935663 | 0.798253766 | 0.9207823 | 1 |
| 182_28 | A_23_P207766 | NM_004309 | ARHGDIA | 0.265396098 | 0.072952097 | 0.459040517 | 1 | -0.451498579 | 0.004855297 | 0.0592272 | 1 |
| 11_5 | A_23_P151075 | NM_001175 | ARHGDIB | -0.122335006 | 0.417628933 | 0.815313407 | 1 | -0.064918186 | 0.627655275 | 0.8333068 | 1 |
| 10_144 | A_33_P3292864 | AK090448 | ARHGEF1; LOC100505585 | 0.028780592 | 0.846146296 | 0.964667249 | 1 | 0.010258649 | 0.932298243 | 0.9756731 | 1 |
| 355_3 | A_23_P216282 | NM_014629 | ARHGEF10 | -0.482920018 | 0.005179223 | 0.20287232 | 1 | 0.186631501 | 0.230296865 | 0.5183408 | 1 |
| 322_105 | A_33_P3799936 | NM_018125 | ARHGEF10L | -0.507028745 | 0.001693732 | 0.132263101 | 1 | -0.73216974 | 0.000147733 | 0.006619 | 1 |
| 46_7 | A_23_P50357 | NM_015318 | ARHGEF18 | -0.021303353 | 0.87476639 | 0.970720179 | 1 | 0.038339091 | 0.759171526 | 0.9032757 | 1 |
| 140_16 | A_23_P332326 | NM_153213 | ARHGEF19 | -0.110276664 | 0.44229935 | 0.828716248 | 1 | 0.152191515 | 0.197683247 | 0.4807229 | 1 |
| 322_144 | A_23_P253221 | NM_032995 | ARHGEF4 | 0.133446888 | 0.499664299 | 0.8517872 | 1 | 0.20286499 | 0.31157351 | 0.6004496 | 1 |
| 129_158 | A_33_P3291877 | NM_020732 | ARID1B | 0.052800559 | 0.692866601 | 0.924234842 | 1 | -0.06403218 | 0.569793764 | 0.8006585 | 1 |
| 65_9 | A_23_P88580 | NM_006465 | ARID3B | 0.136803094 | 0.411168898 | 0.812009855 | 1 | -0.263571822 | 0.03104699 | 0.1787029 | 1 |
| 95_74 | A_24_P94651 | NM_006321 | ARIH2 | -0.158685865 | 0.226693569 | 0.671754088 | 1 | -0.230916174 | 0.151838539 | 0.4204156 | 1 |
| 193_57 | A_23_P61881 | NM_006321 | ARIH2 | 0.261061269 | 0.083207762 | 0.476333154 | 1 | -0.034210938 | 0.82494803 | 0.9336255 | 1 |
| 382_117 | A_24_P338648 | NM_001177 | ARL1 | -0.035474773 | 0.793386753 | 0.94877096 | 1 | -0.074040291 | 0.536739616 | 0.7774257 | 1 |
| 102_7 | A_33_P3407638 | NM_152316 | ARL14EP | -0.232073972 | 0.104338528 | 0.517420159 | 1 | -0.073403143 | 0.53576603 | 0.7766156 | 1 |
| 69_88 | A_32_P207180 | NM_001040025 | ARL16 | 0.260665843 | 0.117533627 | 0.53981859 | 1 | -0.164327318 | 0.255872357 | 0.544934 | 1 |
| 162_111 | A_33_P3328511 | NM_001039083 | ARL17B | 0.091360597 | 0.463669279 | 0.836183443 | 1 | -0.12091634 | 0.283606469 | 0.5743375 | 1 |
| 69_89 | A_23_P98252 | NM_001667 | ARL2 | 0.203026757 | 0.150169066 | 0.586756096 | 1 | -0.228463981 | 0.185227635 | 0.4647011 | 1 |
| 79_85 | A_23_P15182 | NM_012106 | ARL2BP | 0.044710644 | 0.790554819 | 0.94877096 | 1 | -0.015479303 | 0.896118642 | 0.9633162 | 1 |
| 335_90 | A_23_P145761 | NM_005738 | ARL4A | 0.022248088 | 0.861447232 | 0.96731062 | 1 | -0.284496452 | 0.020177199 | 0.1380371 | 1 |
| 279_122 | A_32_P806841 | NM_005738 | ARL4A | 0.0866263 | 0.554043796 | 0.875569848 | 1 | -0.289941692 | 0.047240378 | 0.225428 | 1 |
| 87_5 | A_33_P3323722 | NM_001282431 | ARL4C | 0.007935963 | 0.975052035 | 0.995011522 | 1 | 0.196067141 | 0.147402762 | 0.4163504 | 1 |
| 93_116 | A_24_P257348 | NM_006407 | ARL6IP5 | 0.184179402 | 0.215945994 | 0.662310915 | 1 | -0.086240497 | 0.460026011 | 0.7224734 | 1 |
| 31_21 | A_23_P138271 | NM_138795 | ARL8A | 0.009875546 | 0.941598171 | 0.988655536 | 1 | -0.016171921 | 0.915128704 | 0.9704901 | 1 |
| 130_31 | A_23_P385217 | NM_018184 | ARL8B | 0.045014296 | 0.74647948 | 0.941470372 | 1 | 0.027636383 | 0.82335848 | 0.932608 | 1 |
| 144_6 | A_33_P3213432 | NM_031905 | ARMC10 | -0.080366732 | 0.546654639 | 0.871908135 | 1 | 0.021324311 | 0.875890122 | 0.9570714 | 1 |
| 128_30 | A_32_P90080 | NM_031905 | ARMC10 | -0.015974972 | 0.911506365 | 0.979345742 | 1 | 0.077574006 | 0.497362921 | 0.7523659 | 1 |
| 36_148 | A_23_P32913 | NM_031905 | ARMC10 | 0.102252379 | 0.497258584 | 0.850906598 | 1 | -0.087530593 | 0.502244263 | 0.7563734 | 1 |
| 136_15 | A_33_P3221563 | NM_024742 | ARMC5 | -0.230348213 | 0.264740793 | 0.707490253 | 1 | 0.38101222 | 0.00864229 | 0.0848166 | 1 |
| 142_106 | A_33_P3221568 | NM_024742 | ARMC5 | 0.143407502 | 0.617912154 | 0.89867047 | 1 | 0.141747327 | 0.65807231 | 0.8538693 | 1 |
| 154_61 | A_23_P425750 | NM_033415 | ARMC6 | 0.206283165 | 0.160281196 | 0.598752215 | 1 | -0.054203876 | 0.723595344 | 0.8871476 | 1 |
| 46_34 | A_23_P15375 | ENST00000245543 | ARMC7 | -0.104982539 | 0.397821704 | 0.801565331 | 1 | -0.192879376 | 0.138473255 | 0.4036889 | 1 |
| 286_89 | A_33_P3425356 | NM_015396 | ARMC8 | -0.00639309 | 0.957997748 | 0.99229078 | 1 | 0.110618188 | 0.313914019 | 0.6015453 | 1 |
| 110_104 | A_23_P209731 | NM_025139 | ARMC9 | 0.087200688 | 0.509932959 | 0.856625408 | 1 | 0.229280173 | 0.172859311 | 0.4507648 | 1 |
| 55_56 | A_23_P85188 | NM_022838 | ARMCX5 | 0.032990958 | 0.786504018 | 0.94877096 | 1 | 0.442826493 | 0.004147171 | 0.0537984 | 1 |
| 153_91 | A_23_P62351 | NM_019007 | ARMCX6 | 0.637739212 | 0.002397775 | 0.15293036 | 1 | 0.488241283 | 0.018758267 | 0.1336972 | 1 |
| 346_65 | A_23_P83579 | NM_014862 | ARNT2 | 0.158504479 | 0.345754382 | 0.7684824 | 1 | -0.452603024 | 0.006680306 | 0.072583 | 1 |
| 152_12 | A_24_P72479 | NM_006409 | ARPC1A | 0.048784719 | 0.743373659 | 0.941128659 | 1 | 0.040684942 | 0.737813882 | 0.8935278 | 1 |
| 206_24 | A_23_P102122 | NM_152862 | ARPC2 | -0.139549868 | 0.28789748 | 0.727175432 | 1 | 0.600075876 | 0.00042793 | 0.0128345 | 1 |
| 85_57 | A_24_P167473 | NM_001278556 | ARPC3 | -0.168891531 | 0.229579951 | 0.67569854 | 1 | 0.033863703 | 0.792985991 | 0.9185081 | 1 |
| 289_78 | A_23_P380928 | NM_001198793 | ARPC4-TTLL3 | 0.036190855 | 0.849269657 | 0.965069985 | 1 | -0.088395427 | 0.693408089 | 0.8721495 | 1 |
| 109_162 | A_23_P126803 | NM_005717 | ARPC5 | -0.125046611 | 0.375544705 | 0.789123966 | 1 | 0.121668214 | 0.29839414 | 0.588469 | 1 |
| 224_103 | A_33_P3338909 | NM_001270439 | ARPC5 | 0.301161949 | 0.058538368 | 0.425739937 | 1 | 0.005928699 | 0.969512882 | 0.9890302 | 1 |
| 130_125 | A_24_P29975 | NM_030978 | ARPC5L | 0.121360143 | 0.594424344 | 0.889919794 | 1 | -0.184371816 | 0.28158842 | 0.5725954 | 1 |
| 74_66 | A_24_P404033 | NM_182616 | ARPIN | 0.023560135 | 0.883180672 | 0.972820007 | 1 | 0.106105864 | 0.395210214 | 0.6754185 | 1 |
| 94_36 | A_23_P205768 | NM_006628 | ARPP19 | 0.041569599 | 0.750874671 | 0.942061365 | 1 | 0.235608253 | 0.078544804 | 0.3019352 | 1 |
| 185_146 | A_23_P158829 | NM_004313 | ARRB2 | -0.090241781 | 0.481710398 | 0.842298291 | 1 | -0.006339907 | 0.962108216 | 0.98689 | 1 |
| 256_2 | A_23_P135995 | NM_022786 | ARV1 | -0.355815511 | 0.030469919 | 0.34142035 | 1 | -0.089003885 | 0.488789699 | 0.7457159 | 1 |
| 193_117 | A_33_P3378531 | NM_020682 | AS3MT | 0.064170517 | 0.623361186 | 0.899250294 | 1 | -0.001377727 | 0.991206728 | 0.9974469 | 1 |
| 338_139 | A_33_P3284463 | NM_177924 | ASAH1 | -0.169235069 | 0.280544269 | 0.721858583 | 1 | 0.289679967 | 0.050139287 | 0.2340272 | 1 |
| 223_77 | A_23_P165360 | NM_001040445 | ASB1 | 0.253187998 | 0.098762219 | 0.503608772 | 1 | 0.26189544 | 0.050040638 | 0.2338818 | 1 |
| 70_46 | A_33_P3290124 | NM_001142459 | ASB10 | -0.191715863 | 0.282598293 | 0.724220286 | 1 | 0.165459964 | 0.185040718 | 0.4644394 | 1 |
| 47_107 | A_24_P278299 | NM_024701 | ASB13 | 0.242735763 | 0.149559644 | 0.58620836 | 1 | 0.252316967 | 0.07975654 | 0.3040752 | 1 |
| 94_16 | A_33_P3349474 | A_33_P3349474 | ASB3 | -0.276296399 | 0.118503222 | 0.540937779 | 1 | 0.477545299 | 0.006914214 | 0.0740401 | 1 |
| 205_159 | A_23_P125643 | NM_001031739 | ASB9 | -0.125411121 | 0.393860589 | 0.799268648 | 1 | -0.0752847 | 0.601100947 | 0.8185598 | 1 |
| 308_31 | A_23_P119254 | NM_018154 | ASF1B | -0.097734888 | 0.441207938 | 0.82834854 | 1 | -0.201979053 | 0.100909682 | 0.3447902 | 1 |
| 139_154 | A_23_P204751 | NM_020039 | ASIC1 | -0.256864011 | 0.086763262 | 0.481406415 | 1 | 0.207376844 | 0.114799994 | 0.3668055 | 1 |
| 41_43 | A_33_P3250730 | NM_182847 | ASIC4 | 0.125263731 | 0.384135029 | 0.79324867 | 1 | 0.044617227 | 0.801092859 | 0.9221523 | 1 |
| 56_37 | A_23_P159539 | NM_004192 | ASMTL | -0.339029058 | 0.018948093 | 0.302514544 | 1 | -0.101492347 | 0.362914583 | 0.6473751 | 1 |
| 352_103 | A_23_P145694 | NM_001673 | ASNS | -0.01065679 | 0.956003947 | 0.991603121 | 1 | 0.918394087 | 0.002847341 | 0.0427557 | 1 |
| 152_114 | A_23_P91001 | NM_019048 | ASNSD1 | -0.211941078 | 0.154143921 | 0.593483326 | 1 | 0.277784439 | 0.044962007 | 0.2193011 | 1 |
| 300_16 | A_24_P295245 | NM_032467 | ASPH | -0.204018811 | 0.115308087 | 0.537023243 | 1 | 0.231952079 | 0.068528923 | 0.2783027 | 1 |
| 322_113 | A_33_P3314276 | NM_181718 | ASPHD1 | -0.484375009 | 0.002356778 | 0.151106696 | 1 | 0.319333585 | 0.02731755 | 0.1661171 | 1 |
| 181_53 | A_23_P52017 | NM_018136 | ASPM | -0.292090057 | 0.098402953 | 0.502618353 | 1 | 0.196113734 | 0.120422098 | 0.3764825 | 1 |
| 204_42 | A_33_P3288159 | NM_018136 | ASPM | -0.031486703 | 0.819780494 | 0.956800927 | 1 | 0.050476579 | 0.697488339 | 0.8739701 | 1 |
| 288_48 | A_33_P3228573 | NM_001251888 | ASPSCR1 | -0.535237971 | 0.003347805 | 0.171703571 | 1 | 0.281275991 | 0.100920174 | 0.3447902 | 1 |
| 86_10 | A_23_P203391 | NM_001083926 | ASRGL1 | -0.403784146 | 0.046981378 | 0.399349089 | 1 | -0.059455499 | 0.601937953 | 0.8190172 | 1 |
| 275_117 | A_33_P3234580 | NM_000050 | ASS1 | -0.527325104 | 0.006371977 | 0.219275219 | 1 | 0.364671832 | 0.054313561 | 0.2450319 | 1 |
| 280_76 | A_24_P83738 | NM_198188 | ASTN2 | 0.083945463 | 0.51346614 | 0.858619701 | 1 | 0.031399965 | 0.851026361 | 0.9453144 | 1 |
| 190_95 | A_23_P36464 | NM_018164 | ASUN | 0.345426496 | 0.02469657 | 0.318700868 | 1 | -0.216340648 | 0.268925158 | 0.5592426 | 1 |
| 266_131 | A_33_P3436316 | NM_001164603 | ASXL1 | -0.170071083 | 0.284157175 | 0.724791187 | 1 | 0.238438701 | 0.073379204 | 0.2892786 | 1 |
| 373_145 | A_23_P210300 | NM_018263 | ASXL2 | 0.001791189 | 0.990780452 | 0.997659735 | 1 | 0.079364384 | 0.471004507 | 0.7320879 | 1 |
| 372_60 | A_23_P75288 | NM_032810 | ATAD1 | -0.159831418 | 0.291164528 | 0.728607744 | 1 | 0.227152834 | 0.069903954 | 0.2813922 | 1 |
| 342_37 | A_33_P3217238 | NM_014109 | ATAD2 | -0.098484114 | 0.542723213 | 0.870410285 | 1 | 0.330656452 | 0.043184933 | 0.2153193 | 1 |
| 101_76 | A_33_P3349469 | NM_018188 | ATAD3A | 0.052043331 | 0.674240264 | 0.918166391 | 1 | 0.175170863 | 0.205188588 | 0.487816 | 1 |
| 39_120 | A_33_P3331588 | NM_031921 | ATAD3B | 0.03582485 | 0.844504298 | 0.964376494 | 1 | 0.162759315 | 0.249634197 | 0.5368329 | 1 |
| 231_162 | A_33_P3385477 | NM_031921 | ATAD3B | 0.072159473 | 0.618007874 | 0.89867047 | 1 | 0.17358341 | 0.164908867 | 0.4405759 | 1 |
| 71_17 | A_33_P3402474 | NM_001031722 | ATAT1 | -0.211319765 | 0.292636079 | 0.730061993 | 1 | -0.165444341 | 0.349776508 | 0.6349155 | 1 |
| 186_160 | A_33_P3294252 | NM_005171 | ATF1 | 0.223479373 | 0.094707434 | 0.498686523 | 1 | -0.177493236 | 0.177116346 | 0.4554247 | 1 |
| 59_7 | A_33_P3214096 | NM_001040619 | ATF3 | -0.000324847 | 0.998190549 | 0.999808735 | 1 | 0.064907524 | 0.700241091 | 0.8751652 | 1 |
| 240_141 | A_23_P120933 | NM_001675 | ATF4 | 0.069920632 | 0.627234154 | 0.900268731 | 1 | 0.373147166 | 0.03560472 | 0.1935458 | 1 |
| 337_120 | A_21_P0000168 | NM_001206682 | ATF7 | 0.267430813 | 0.065289245 | 0.442847206 | 1 | -0.455928048 | 0.00206236 | 0.0342805 | 1 |
| 205_24 | A_23_P112950 | NM_018179 | ATF7IP | -0.19870676 | 0.120876036 | 0.543077746 | 1 | 0.109686693 | 0.399459944 | 0.677591 | 1 |
| 336_35 | A_23_P112950 | NM_018179 | ATF7IP | -0.181022253 | 0.205345566 | 0.651946752 | 1 | 0.214535779 | 0.187770438 | 0.4683642 | 1 |
| 332_30 | A_23_P112950 | NM_018179 | ATF7IP | -0.172432576 | 0.249993309 | 0.694615167 | 1 | 0.188062292 | 0.162720436 | 0.4373039 | 1 |
| 65_61 | A_23_P112950 | NM_018179 | ATF7IP | -0.146574238 | 0.261251047 | 0.703328232 | 1 | 0.186363276 | 0.220612859 | 0.5080791 | 1 |
| 18_90 | A_23_P112950 | NM_018179 | ATF7IP | -0.121969416 | 0.362804639 | 0.779088927 | 1 | -0.02022471 | 0.878359273 | 0.9582221 | 1 |
| 307_134 | A_23_P112950 | NM_018179 | ATF7IP | -0.09214331 | 0.531218818 | 0.865842607 | 1 | 0.022193406 | 0.867031081 | 0.9531691 | 1 |
| 143_115 | A_23_P112950 | NM_018179 | ATF7IP | -0.057629189 | 0.660856002 | 0.912353222 | 1 | -0.00339947 | 0.981159039 | 0.9934423 | 1 |
| 174_58 | A_23_P112950 | NM_018179 | ATF7IP | -0.047341254 | 0.710515284 | 0.930633257 | 1 | 0.009695483 | 0.951650112 | 0.983258 | 1 |
| 3_103 | A_23_P112950 | NM_018179 | ATF7IP | -0.024705692 | 0.852946028 | 0.965079421 | 1 | 0.165006432 | 0.40446185 | 0.6827157 | 1 |
| 310_118 | A_23_P112950 | NM_018179 | ATF7IP | 0.059518256 | 0.664475366 | 0.914064838 | 1 | 0.122602506 | 0.450863481 | 0.7185093 | 1 |
| 306_51 | A_19_P00809417 | ENST00000570163 | ATF7IP2 | -0.401386737 | 0.00803242 | 0.234464792 | 1 | -0.143725524 | 0.351068664 | 0.6362217 | 1 |
| 55_117 | A_23_P20970 | NM_004707 | ATG12 | -0.03744305 | 0.769566212 | 0.945351992 | 1 | 0.388903325 | 0.017768653 | 0.1292509 | 1 |
| 179_102 | A_33_P3257312 | NM_015104 | ATG2A | 0.038813988 | 0.763364739 | 0.944092309 | 1 | 0.142749431 | 0.419341014 | 0.6952135 | 1 |
| 257_160 | A_23_P212706 | NM_022488 | ATG3 | 0.235296445 | 0.093826257 | 0.496736838 | 1 | -0.312165435 | 0.027620713 | 0.1671061 | 1 |
| 324_133 | A_24_P182764 | NM_178326 | ATG4B | -0.098949442 | 0.502678386 | 0.854062497 | 1 | 0.202148619 | 0.168341521 | 0.4454911 | 1 |
| 340_14 | A_23_P111381 | NM_004849 | ATG5 | -0.032857005 | 0.789619594 | 0.94877096 | 1 | 0.022879451 | 0.836300627 | 0.9381575 | 1 |
| 161_3 | A_23_P68087 | NM_004044 | ATIC | 0.183896738 | 0.195141543 | 0.641277117 | 1 | 0.406091763 | 0.023916476 | 0.1542366 | 1 |
| 50_153 | A_23_P209619 | NM_022374 | ATL2 | 0.057031941 | 0.752126735 | 0.942109358 | 1 | -0.111261024 | 0.334926599 | 0.6212124 | 1 |
| 17_29 | A_23_P105028 | NM_015459 | ATL3 | 0.068837963 | 0.58126833 | 0.883808109 | 1 | 0.045593445 | 0.74051193 | 0.8949226 | 1 |
| 4_98 | A_24_P167984 | NM_015251 | ATMIN | 0.26702434 | 0.087751638 | 0.483706087 | 1 | -0.038991389 | 0.848503694 | 0.9444119 | 1 |
| 184_115 | A_23_P13885 | NM_001007026 | ATN1 | 0.109182257 | 0.373836495 | 0.787569195 | 1 | -0.217835 | 0.071320106 | 0.2842704 | 1 |
| 89_16 | A_23_P144877 | NM_004045 | ATOX1 | -0.15533533 | 0.298174239 | 0.734106423 | 1 | 0.181331116 | 0.141458701 | 0.4082592 | 1 |
| 334_68 | A_33_P3298942 | ENST00000326831 | ATP10B | -0.141928933 | 0.362179044 | 0.778646623 | 1 | 0.268351226 | 0.294961349 | 0.5859887 | 1 |
| 108_58 | A_24_P183094 | NM_024524 | ATP13A3 | 0.133471109 | 0.364919015 | 0.781135731 | 1 | 0.052042981 | 0.698991638 | 0.874605 | 1 |
| 44_136 | A_23_P1072 | NM_000701 | ATP1A1 | 0.407878155 | 0.028082135 | 0.333708589 | 1 | -0.11077604 | 0.53716241 | 0.7778377 | 1 |
| 356_78 | A_21_P0000543 | NR_027645 | ATP1A1-AS1 | -0.142085923 | 0.446646344 | 0.830187024 | 1 | 0.308771716 | 0.109287628 | 0.3583551 | 1 |
| 123_85 | A_21_P0000544 | NR_027646 | ATP1A1-AS1 | -0.067156435 | 0.680447992 | 0.919745252 | 1 | 0.407503311 | 0.027824646 | 0.1673964 | 1 |
| 78_133 | A_23_P62932 | NM_001677 | ATP1B1 | -0.199271718 | 0.172692189 | 0.612857861 | 1 | -0.086814859 | 0.529273929 | 0.7719664 | 1 |
| 175_137 | A_23_P68007 | NM_001679 | ATP1B3 | 0.046642271 | 0.732189422 | 0.937415492 | 1 | 0.012634908 | 0.906172908 | 0.9671405 | 1 |
| 384_151 | A_22_P00012736 | NR_046287 | ATP2A1-AS1 | -0.134016795 | 0.307691001 | 0.740336569 | 1 | -0.072483608 | 0.559229977 | 0.7942406 | 1 |
| 109_53 | A_24_P73290 | NM_001681 | ATP2A2 | 0.002336044 | 0.984725312 | 0.995672988 | 1 | -0.020843501 | 0.849411665 | 0.9450036 | 1 |
| 248_118 | A_24_P245358 | NM_001001937 | ATP5A1 | 0.532009031 | 0.046296009 | 0.398016002 | 1 | -0.436306464 | 0.085408524 | 0.314529 | 1 |
| 106_54 | A_23_P33216 | NM_001686 | ATP5B | 0.297450056 | 0.114929902 | 0.536427612 | 1 | 0.008170168 | 0.978652564 | 0.9924178 | 1 |
| 169_13 | A_23_P5089 | NM_001001975 | ATP5D | -0.103023156 | 0.416961282 | 0.814662764 | 1 | -0.498544291 | 0.002303012 | 0.0366365 | 1 |
| 57_16 | A_23_P252322 | NM_006886 | ATP5E | -0.183792081 | 0.232097781 | 0.678037209 | 1 | 0.237188977 | 0.066321704 | 0.2735314 | 1 |
| 193_113 | A_23_P46275 | NM_001688 | ATP5F1 | 0.472040808 | 0.008352959 | 0.234464792 | 1 | -0.285798237 | 0.033849628 | 0.1882823 | 1 |
| 191_1 | A_23_P164228 | NM_005175 | ATP5G1 | 0.237643774 | 0.149265744 | 0.585999127 | 1 | -0.373225038 | 0.006998263 | 0.0745417 | 1 |
| 338_117 | A_23_P87616 | NM_001002031 | ATP5G2 | 0.212480316 | 0.134228275 | 0.56431697 | 1 | -0.041948582 | 0.704285549 | 0.8766152 | 1 |
| 80_7 | A_23_P56680 | NM_001002258 | ATP5G3 | 0.133981165 | 0.33552227 | 0.762359625 | 1 | 0.308975991 | 0.054966466 | 0.2471843 | 1 |
| 213_91 | A_23_P77818 | NM_006356 | ATP5H | -0.206088634 | 0.189726485 | 0.632544514 | 1 | 0.500132912 | 0.00752359 | 0.077917 | 1 |
| 184_143 | A_24_P302998 | NM_007100 | ATP5I | 0.039758661 | 0.782170954 | 0.948624895 | 1 | -0.141575659 | 0.269627066 | 0.5598109 | 1 |
| 360_32 | A_23_P154832 | NM_001003703 | ATP5J | -0.116730628 | 0.387941805 | 0.795892662 | 1 | 0.081634331 | 0.595559447 | 0.8162347 | 1 |
| 106_41 | A_23_P215832 | NM_004889 | ATP5J2 | 0.04212572 | 0.747767736 | 0.941597731 | 1 | -0.229538196 | 0.116051133 | 0.3692297 | 1 |
| 227_119 | A_24_P343377 | NM_001003714 | ATP5J2 | 0.187184968 | 0.150952174 | 0.587883436 | 1 | -0.214917001 | 0.102411794 | 0.3475203 | 1 |
| 152_76 | A_23_P75622 | NM_006476 | ATP5L | 0.049235734 | 0.749072062 | 0.941597731 | 1 | -0.080821627 | 0.655684231 | 0.8521388 | 1 |
| 178_99 | A_33_P3791123 | NM_001165877 | ATP5L2 | 0.11072409 | 0.574779559 | 0.882971575 | 1 | -0.129578693 | 0.395205322 | 0.6754185 | 1 |
| 17_18 | A_23_P143474 | NM_001697 | ATP5O | -0.153980436 | 0.26280096 | 0.705163281 | 1 | 0.199981364 | 0.079398797 | 0.3036376 | 1 |
| 336_58 | A_24_P118231 | NM_001003803 | ATP5S | 0.048676957 | 0.699683602 | 0.925289905 | 1 | 0.078171006 | 0.570032245 | 0.8006935 | 1 |
| 111_2 | A_23_P107795 | NM_018035 | ATP5SL | -0.293492148 | 0.032496363 | 0.349092326 | 1 | -0.017883872 | 0.890168769 | 0.9618338 | 1 |
| 292_74 | A_23_P107795 | NM_018035 | ATP5SL | -0.176047136 | 0.163232187 | 0.603384225 | 1 | -0.045959127 | 0.708467671 | 0.879001 | 1 |
| 43_122 | A_23_P107795 | NM_018035 | ATP5SL | -0.125508984 | 0.343111329 | 0.767452043 | 1 | -0.158763567 | 0.263367719 | 0.5536629 | 1 |
| 225_78 | A_23_P107795 | NM_018035 | ATP5SL | -0.114029234 | 0.426427817 | 0.820784274 | 1 | 0.034070452 | 0.836693411 | 0.9384016 | 1 |
| 316_94 | A_23_P107795 | NM_018035 | ATP5SL | -0.111209727 | 0.410506517 | 0.811684855 | 1 | -0.034327968 | 0.776077197 | 0.9107549 | 1 |
| 76_97 | A_23_P107795 | NM_018035 | ATP5SL | -0.106147543 | 0.407660251 | 0.808758102 | 1 | -0.027906436 | 0.827987389 | 0.9344144 | 1 |
| 155_51 | A_23_P107795 | NM_018035 | ATP5SL | -0.100151983 | 0.461537113 | 0.834947603 | 1 | -0.057695488 | 0.677690324 | 0.8655072 | 1 |
| 99_123 | A_23_P107795 | NM_018035 | ATP5SL | -0.072752844 | 0.565532974 | 0.880639801 | 1 | -0.156082354 | 0.215698861 | 0.5020623 | 1 |
| 173_84 | A_23_P107795 | NM_018035 | ATP5SL | -0.061442334 | 0.645547797 | 0.907045535 | 1 | -0.004274897 | 0.976128489 | 0.9916274 | 1 |
| 212_119 | A_23_P107795 | NM_018035 | ATP5SL | 0.061767935 | 0.635149424 | 0.902340549 | 1 | -0.150815629 | 0.30312706 | 0.593208 | 1 |
| 229_164 | A_23_P250462 | NM_001183 | ATP6AP1 | 0.021691133 | 0.889536258 | 0.973789602 | 1 | 0.706960767 | 0.001006549 | 0.0222353 | 1 |
| 257_8 | A_23_P11353 | NM_005765 | ATP6AP2 | -0.452417396 | 0.003389365 | 0.172038518 | 1 | -0.433593178 | 0.00470226 | 0.0577362 | 1 |
| 60_7 | A_23_P11353 | NM_005765 | ATP6AP2 | -0.407401976 | 0.006927422 | 0.225693626 | 1 | -0.461020216 | 0.004935944 | 0.0596931 | 1 |
| 138_16 | A_23_P11353 | NM_005765 | ATP6AP2 | -0.393414781 | 0.009097266 | 0.240080283 | 1 | -0.384221298 | 0.009635204 | 0.0896337 | 1 |
| 128_5 | A_23_P11353 | NM_005765 | ATP6AP2 | -0.343401884 | 0.017980827 | 0.296237697 | 1 | -0.557521386 | 0.000665778 | 0.0174449 | 1 |
| 356_85 | A_23_P11353 | NM_005765 | ATP6AP2 | -0.322744973 | 0.025383159 | 0.321845715 | 1 | -0.460411864 | 0.002845321 | 0.0427557 | 1 |
| 35_124 | A_23_P11353 | NM_005765 | ATP6AP2 | -0.31593859 | 0.022509721 | 0.31323834 | 1 | -0.527575726 | 0.004001916 | 0.0525158 | 1 |
| 40_105 | A_23_P11353 | NM_005765 | ATP6AP2 | -0.22728208 | 0.084867129 | 0.478632531 | 1 | -0.5598247 | 0.001246233 | 0.0255773 | 1 |
| 336_76 | A_23_P11353 | NM_005765 | ATP6AP2 | -0.217539497 | 0.115984384 | 0.537495321 | 1 | -0.545972693 | 0.000592179 | 0.0162073 | 1 |
| 274_76 | A_23_P11353 | NM_005765 | ATP6AP2 | -0.208964779 | 0.16941896 | 0.608706095 | 1 | -0.461939654 | 0.004678457 | 0.0576958 | 1 |
| 162_107 | A_23_P11353 | NM_005765 | ATP6AP2 | -0.142379312 | 0.31169652 | 0.743773338 | 1 | -0.465464093 | 0.01825442 | 0.1314388 | 1 |
| 206_15 | A_24_P333733 | NM_005177 | ATP6V0A1 | -0.1848209 | 0.200625827 | 0.647493287 | 1 | -0.198934953 | 0.110130264 | 0.3598591 | 1 |
| 302_118 | A_23_P137814 | NM_004047 | ATP6V0B | 0.300414661 | 0.063276199 | 0.43623693 | 1 | -0.545069768 | 0.012151813 | 0.1031483 | 1 |
| 74_93 | A_23_P54816 | NM_001694 | ATP6V0C | 0.072752852 | 0.582386396 | 0.883808109 | 1 | -0.113519803 | 0.358661004 | 0.6432563 | 1 |
| 244_113 | A_23_P54636 | NM_004691 | ATP6V0D1 | 0.26406537 | 0.055357466 | 0.422185511 | 1 | -0.426379933 | 0.042375449 | 0.2132424 | 1 |
| 215_155 | A_32_P146659 | NR_027040 | ATP6V0E2-AS1 | 0.081013249 | 0.566327985 | 0.880639801 | 1 | 0.19094224 | 0.278282332 | 0.5695244 | 1 |
| 383_64 | A_24_P396994 | NM_001690 | ATP6V1A | -0.078342435 | 0.569094959 | 0.881307748 | 1 | -0.057038352 | 0.742966584 | 0.8959092 | 1 |
| 322_129 | A_33_P3380897 | NM_001695 | ATP6V1C1 | -0.176718296 | 0.312775883 | 0.744404144 | 1 | 0.043518674 | 0.726692112 | 0.888579 | 1 |
| 114_79 | A_23_P146058 | NM_001695 | ATP6V1C1 | -0.12480425 | 0.327645428 | 0.758096222 | 1 | 0.370624684 | 0.006123484 | 0.0685964 | 1 |
| 286_104 | A_33_P3387463 | NM_015994 | ATP6V1D | -0.11803551 | 0.355289095 | 0.774004626 | 1 | 0.070352602 | 0.562079904 | 0.7958803 | 1 |
| 302_132 | A_23_P93623 | NM_004231 | ATP6V1F | 0.019792088 | 0.88219551 | 0.972820007 | 1 | -0.058507957 | 0.695704155 | 0.873377 | 1 |
| 12_158 | A_23_P20882 | NM_004888 | ATP6V1G1 | 0.504502714 | 0.010893097 | 0.256586468 | 1 | -0.227916837 | 0.200629915 | 0.4835834 | 1 |
| 31_53 | A_33_P3410700 | ENST00000361851 | ATP8; ATP6; COX2 | -0.816385366 | 0.010700707 | 0.255433159 | 1 | -0.327521264 | 0.18849392 | 0.4692322 | 1 |
| 77_135 | A_23_P337726 | ENST00000361899 | ATP8; ATP6; COX2 | -0.524091556 | 0.117181584 | 0.53981859 | 1 | -1.045918041 | 5.92295E-06 | 0.0013178 | 0.06662 |
| 145_94 | A_24_P350200 | ENST00000416718 | ATP8; ATP6; COX3 | -0.03551902 | 0.848359221 | 0.965069985 | 1 | -0.035691792 | 0.869944299 | 0.9542093 | 1 |
| 330_1 | A_23_P126716 | NM_178191 | ATPIF1 | -0.325939708 | 0.027455323 | 0.332289262 | 1 | 0.034622547 | 0.807962453 | 0.9250895 | 1 |
| 86_138 | A_33_P3228385 | NM_016311 | ATPIF1 | -0.137398936 | 0.30129518 | 0.736806173 | 1 | -0.125990685 | 0.376540735 | 0.6584194 | 1 |
| 56_6 | A_23_P136058 | NM_001184 | ATR | -0.207759483 | 0.121005358 | 0.543252217 | 1 | 0.286031239 | 0.024066436 | 0.1546423 | 1 |
| 42_163 | A_23_P28652 | NM_016085 | ATRAID | 0.07102172 | 0.573752008 | 0.882728841 | 1 | -0.086744239 | 0.475943941 | 0.7355842 | 1 |
| 277_66 | A_33_P3343473 | NM_207303 | ATRNL1 | 0.004571921 | 0.980257272 | 0.995429381 | 1 | -0.037159751 | 0.853758716 | 0.9469649 | 1 |
| 80_33 | A_33_P3319943 | NM_000489 | ATRX | -0.115748861 | 0.395701995 | 0.800591379 | 1 | -0.099627546 | 0.420897527 | 0.6965338 | 1 |
| 370_45 | A_33_P3612589 | NM_000489 | ATRX | -0.072238278 | 0.568943235 | 0.881307748 | 1 | -0.000280502 | 0.998426271 | 0.9989592 | 1 |
| 79_138 | A_33_P3231367 | NM_013236 | ATXN10 | -0.054229647 | 0.67294926 | 0.9176033 | 1 | -0.267023372 | 0.104226142 | 0.3508624 | 1 |
| 139_105 | A_24_P391368 | NM_013236 | ATXN10 | 0.294231017 | 0.043106656 | 0.390137659 | 1 | -0.178055363 | 0.164917281 | 0.4405759 | 1 |
| 183_86 | A_33_P3369969 | NM_001137675 | ATXN1L | 0.28863416 | 0.068517006 | 0.450747228 | 1 | 0.123308629 | 0.37173562 | 0.6550662 | 1 |
| 230_83 | A_23_P389907 | NM_002973 | ATXN2 | -0.039344673 | 0.786277029 | 0.94877096 | 1 | 0.361239155 | 0.033657364 | 0.18777 | 1 |
| 60_52 | A_32_P155091 | NM_145714 | ATXN2L | -0.235584741 | 0.075209883 | 0.461626338 | 1 | 0.236152657 | 0.074312335 | 0.2914194 | 1 |
| 215_5 | A_33_P3369956 | NM_148416 | ATXN2L | -0.087457181 | 0.50229582 | 0.853951304 | 1 | 0.021302889 | 0.848041655 | 0.9441179 | 1 |
| 93_13 | A_22_P00001843 | NM_004993 | ATXN3 | -0.260425867 | 0.089769755 | 0.487767685 | 1 | 0.071449148 | 0.618435266 | 0.8269577 | 1 |
| 327_86 | A_23_P20852 | NM_001698 | AUH | -0.196343059 | 0.125253518 | 0.550879305 | 1 | -0.210434453 | 0.083786768 | 0.3115222 | 1 |
| 149_141 | A_23_P160537 | NM_024037 | AUNIP | -0.086212256 | 0.549558538 | 0.873756475 | 1 | 0.171119357 | 0.19623233 | 0.4783323 | 1 |
| 3_67 | A_23_P131866 | NM_198433 | AURKA | 0.204253335 | 0.144074993 | 0.578530626 | 1 | -0.119268151 | 0.531277696 | 0.7730606 | 1 |
| 89_163 | A_33_P3240518 | NM_001127230 | AURKAIP1 | 0.075704988 | 0.66790714 | 0.916365018 | 1 | -0.432992373 | 0.016832386 | 0.1245737 | 1 |
| 195_137 | A_33_P3230017 | NR_001587 | AURKAPS1 | 0.078754288 | 0.548807627 | 0.873756475 | 1 | -0.115450816 | 0.339186387 | 0.6259021 | 1 |
| 267_23 | A_23_P100074 | NM_020371 | AVEN | -0.052749539 | 0.670668456 | 0.916686207 | 1 | -0.008662562 | 0.938231987 | 0.9784988 | 1 |
| 269_42 | A_23_P100074 | NM_020371 | AVEN | 0.01311013 | 0.919980357 | 0.981834263 | 1 | 0.024820857 | 0.837420038 | 0.938842 | 1 |
| 97_146 | A_23_P100074 | NM_020371 | AVEN | 0.018516627 | 0.8838434 | 0.972820007 | 1 | -0.061881508 | 0.599851714 | 0.818059 | 1 |
| 48_20 | A_23_P100074 | NM_020371 | AVEN | 0.045609914 | 0.734566476 | 0.93861213 | 1 | 0.022395328 | 0.828879092 | 0.9347493 | 1 |
| 335_63 | A_23_P100074 | NM_020371 | AVEN | 0.080518341 | 0.581919525 | 0.883808109 | 1 | 0.032965011 | 0.793293582 | 0.9185806 | 1 |
| 349_159 | A_23_P100074 | NM_020371 | AVEN | 0.129813513 | 0.311894659 | 0.743773338 | 1 | -0.033909566 | 0.768408829 | 0.9077701 | 1 |
| 372_65 | A_23_P100074 | NM_020371 | AVEN | 0.137848453 | 0.406036261 | 0.807696561 | 1 | -0.083314195 | 0.511573211 | 0.7603626 | 1 |
| 358_141 | A_23_P100074 | NM_020371 | AVEN | 0.160755285 | 0.21943936 | 0.666470871 | 1 | -0.05092059 | 0.650494727 | 0.8485472 | 1 |
| 288_84 | A_23_P100074 | NM_020371 | AVEN | 0.162993476 | 0.203015105 | 0.649115486 | 1 | 0.047145772 | 0.719180766 | 0.8846797 | 1 |
| 177_67 | A_23_P100074 | NM_020371 | AVEN | 0.327571794 | 0.030566584 | 0.341616626 | 1 | 0.095225263 | 0.576927042 | 0.8048695 | 1 |
| 28_40 | A_23_P1492 | NM_021732 | AVPI1 | 0.186079338 | 0.416499106 | 0.814227569 | 1 | -0.499628132 | 0.002102797 | 0.0346936 | 1 |
| 289_72 | A_33_P3396891 | NM_021732 | AVPI1 | 0.190741745 | 0.303439088 | 0.738354373 | 1 | -0.543274462 | 0.004556649 | 0.0567537 | 1 |
| 75_65 | A_23_P208389 | NM_021913 | AXL | 0.088701793 | 0.673754431 | 0.918084617 | 1 | 0.070663718 | 0.549064261 | 0.7866657 | 1 |
| 147_42 | A_33_P3322288 | NM_001134433 | AZI2 | 0.11772572 | 0.361707482 | 0.77846455 | 1 | -0.147323165 | 0.23294552 | 0.5208625 | 1 |
| 191_87 | A_33_P3319491 | NM_015878 | AZIN1 | 0.146154213 | 0.308057575 | 0.740627072 | 1 | -0.131352088 | 0.248349662 | 0.5355889 | 1 |
| 371_79 | A_23_P37441 | NM_004048 | B2M | 0.398270856 | 0.175897521 | 0.616374573 | 1 | -0.242961425 | 0.295275038 | 0.5861204 | 1 |
| 342_70 | A_23_P37441 | NM_004048 | B2M | 0.406939169 | 0.131682353 | 0.561823086 | 1 | -0.25792173 | 0.223779556 | 0.5121792 | 1 |
| 77_67 | A_23_P37441 | NM_004048 | B2M | 0.419869482 | 0.164586147 | 0.60427748 | 1 | -0.281901458 | 0.307954497 | 0.5962064 | 1 |
| 250_58 | A_23_P37441 | NM_004048 | B2M | 0.427148279 | 0.121904267 | 0.544672485 | 1 | -0.352579188 | 0.114648647 | 0.3666767 | 1 |
| 117_152 | A_23_P37441 | NM_004048 | B2M | 0.458087416 | 0.131860766 | 0.561823086 | 1 | -0.285484402 | 0.179731194 | 0.4580641 | 1 |
| 284_98 | A_23_P37441 | NM_004048 | B2M | 0.492932489 | 0.091146055 | 0.490601632 | 1 | -0.252098993 | 0.32197696 | 0.6084864 | 1 |
| 196_53 | A_23_P37441 | NM_004048 | B2M | 0.543883948 | 0.043362015 | 0.390521637 | 1 | -0.293568914 | 0.178440386 | 0.4568562 | 1 |
| 97_107 | A_23_P37441 | NM_004048 | B2M | 0.552304761 | 0.049472665 | 0.404754052 | 1 | -0.302983038 | 0.133870961 | 0.3972612 | 1 |
| 216_87 | A_23_P37441 | NM_004048 | B2M | 0.554231263 | 0.09681297 | 0.50015929 | 1 | -0.260532626 | 0.296839165 | 0.587256 | 1 |
| 152_95 | A_23_P37441 | NM_004048 | B2M | 0.68507744 | 0.027699 | 0.333098929 | 1 | -0.308439121 | 0.226820038 | 0.5148774 | 1 |
| 87_93 | A_23_P334751 | NM_152490 | B3GALNT2 | 0.060236594 | 0.654567366 | 0.909665926 | 1 | -0.112746574 | 0.318270213 | 0.6051097 | 1 |
| 50_101 | A_33_P3278347 | NR_026542 | B3GALT5-AS1 | 0.121421063 | 0.389770548 | 0.796942735 | 1 | -0.038525521 | 0.788560123 | 0.9163904 | 1 |
| 196_25 | A_33_P3407042 | NM_080605 | B3GALT6 | -0.063630391 | 0.687571373 | 0.922029409 | 1 | -0.875571167 | 0.000444964 | 0.0132045 | 1 |
| 325_127 | A_33_P3225685 | NM_001288723 | B3GAT3 | -0.295604169 | 0.044055427 | 0.390521637 | 1 | 0.001294264 | 0.991770932 | 0.9974469 | 1 |
| 22_60 | A_23_P78980 | NM_014256 | B3GNT3 | -0.326414435 | 0.023014604 | 0.314662077 | 1 | 0.201509549 | 0.0744479 | 0.291646 | 1 |
| 30_38 | A_24_P913716 | NM_145236 | B3GNT7 | 0.045307681 | 0.738584441 | 0.940367439 | 1 | 0.109503357 | 0.476529913 | 0.7358085 | 1 |
| 297_55 | A_33_P3846177 | NM_001478 | B4GALNT1 | -0.350472619 | 0.048598176 | 0.402931587 | 1 | -0.403403406 | 0.017058143 | 0.1257228 | 1 |
| 333_5 | A_23_P149206 | NM_003780 | B4GALT2 | -0.035659784 | 0.810182881 | 0.954234541 | 1 | -0.39734989 | 0.003420863 | 0.0477943 | 1 |
| 22_142 | A_23_P103919 | NM_003779 | B4GALT3 | 0.140969515 | 0.33815507 | 0.764199026 | 1 | -0.236560808 | 0.097379572 | 0.3395003 | 1 |
| 238_139 | A_24_P239731 | NM_004776 | B4GALT5 | -0.028501303 | 0.8338099 | 0.961426617 | 1 | -0.272594485 | 0.026520732 | 0.1629048 | 1 |
| 220_155 | A_23_P86900 | NM_006876 | B4GAT1 | 0.044240355 | 0.774913898 | 0.946321166 | 1 | -0.23038316 | 0.070479969 | 0.2821796 | 1 |
| 77_4 | A_24_P176714 | NM_015681 | B9D1 | -0.255435304 | 0.099674934 | 0.506777983 | 1 | -0.181478479 | 0.168963138 | 0.4460427 | 1 |
| 296_102 | A_23_P218476 | NM_030578 | B9D2 | -0.316883936 | 0.025305121 | 0.321845715 | 1 | -0.176536911 | 0.179278423 | 0.4577014 | 1 |
| 47_128 | A_23_P119714 | NM_001033549 | BABAM1 | 0.111354277 | 0.458447609 | 0.833745766 | 1 | -0.298275998 | 0.053061926 | 0.2415166 | 1 |
| 24_93 | A_23_P52806 | NM_012104 | BACE1 | -0.315378384 | 0.139463052 | 0.571643688 | 1 | -0.654742429 | 0.001272063 | 0.0259653 | 1 |
| 161_49 | A_33_P3240941 | NM_012104 | BACE1 | -0.1560488 | 0.289009329 | 0.727382494 | 1 | -0.308611557 | 0.018312174 | 0.1316019 | 1 |
| 16_102 | A_23_P154875 | NM_012105 | BACE2 | 0.124599158 | 0.407982366 | 0.808864993 | 1 | -0.55063812 | 0.001069967 | 0.0231968 | 1 |
| 148_8 | A_33_P3262043 | NM_004322 | BAD | -0.207312065 | 0.19996656 | 0.646494862 | 1 | -0.346217479 | 0.016654806 | 0.1239507 | 1 |
| 36_60 | A_23_P146654 | NM_004323 | BAG1 | 0.304756429 | 0.046850278 | 0.399349089 | 1 | -0.182854571 | 0.103038068 | 0.3486369 | 1 |
| 132_158 | A_33_P3290919 | NM_001172415 | BAG1 | 0.350972014 | 0.021727758 | 0.309199145 | 1 | -0.346560655 | 0.007293013 | 0.0768113 | 1 |
| 206_92 | A_33_P3290924 | NM_001172415 | BAG1 | 0.456441404 | 0.005692099 | 0.211438012 | 1 | -0.394396664 | 0.079281266 | 0.3036011 | 1 |
| 186_42 | A_23_P356554 | NM_004282 | BAG2 | 0.297750468 | 0.065894631 | 0.443753002 | 1 | 0.01084598 | 0.93486963 | 0.9767282 | 1 |
| 273_117 | A_24_P130026 | NM_001015049 | BAG5 | 0.234046897 | 0.095176996 | 0.498686523 | 1 | -0.320455198 | 0.017424691 | 0.1275882 | 1 |
| 242_40 | A_23_P111141 | NM_004639 | BAG6 | 0.144499245 | 0.455050045 | 0.832937561 | 1 | -0.465218423 | 0.04115492 | 0.2096329 | 1 |
| 24_116 | A_33_P3275668 | NM_004639 | BAG6 | 0.14900068 | 0.317222539 | 0.748229895 | 1 | -0.254636676 | 0.066867515 | 0.2752782 | 1 |
| 103_1 | A_23_P117928 | NM_014952 | BAHD1 | -0.130299108 | 0.345741996 | 0.7684824 | 1 | -0.349966713 | 0.014347694 | 0.1136662 | 1 |
| 268_74 | A_23_P117928 | NM_014952 | BAHD1 | -0.076102802 | 0.577498343 | 0.883311119 | 1 | -0.354424149 | 0.020686034 | 0.1404926 | 1 |
| 98_67 | A_23_P117928 | NM_014952 | BAHD1 | 0.023527154 | 0.8927286 | 0.975006258 | 1 | -0.380968547 | 0.07034659 | 0.2820635 | 1 |
| 102_120 | A_23_P117928 | NM_014952 | BAHD1 | 0.05524144 | 0.686871417 | 0.922029409 | 1 | -0.451674414 | 0.004601128 | 0.057055 | 1 |
| 1_118 | A_23_P117928 | NM_014952 | BAHD1 | 0.064745345 | 0.630819676 | 0.901004617 | 1 | -0.366811859 | 0.043293921 | 0.2155497 | 1 |
| 310_158 | A_23_P117928 | NM_014952 | BAHD1 | 0.082461705 | 0.55491418 | 0.875569848 | 1 | -0.345068838 | 0.021619609 | 0.143609 | 1 |
| 133_43 | A_23_P117928 | NM_014952 | BAHD1 | 0.092943134 | 0.531965061 | 0.86624761 | 1 | -0.32462665 | 0.037519985 | 0.1994269 | 1 |
| 16_89 | A_23_P117928 | NM_014952 | BAHD1 | 0.147532212 | 0.294338855 | 0.732509894 | 1 | -0.321537623 | 0.054844694 | 0.2467353 | 1 |
| 195_117 | A_23_P117928 | NM_014952 | BAHD1 | 0.190208594 | 0.14669089 | 0.581701959 | 1 | -0.46506569 | 0.005607654 | 0.0653568 | 1 |
| 148_66 | A_23_P117928 | NM_014952 | BAHD1 | 0.310635504 | 0.063716846 | 0.437788275 | 1 | -0.400839841 | 0.060595099 | 0.2593761 | 1 |
| 235_77 | A_33_P3285156 | NR_026857 | BAIAP2-AS1 | -0.362603063 | 0.023217448 | 0.315256111 | 1 | -0.309012183 | 0.039395357 | 0.2046194 | 1 |
| 270_143 | A_33_P3262575 | NM_018842 | BAIAP2L1 | -0.156695016 | 0.244185011 | 0.69010536 | 1 | 0.212539383 | 0.083352718 | 0.310905 | 1 |
| 142_135 | A_23_P145357 | NM_001188 | BAK1 | 0.062902675 | 0.682380737 | 0.920314282 | 1 | -0.313240776 | 0.017262905 | 0.1268772 | 1 |
| 146_88 | A_23_P52207 | NM_012342 | BAMBI | 0.112895929 | 0.371821409 | 0.78563284 | 1 | -0.725130501 | 0.000543849 | 0.0152917 | 1 |
| 19_129 | A_23_P47208 | NM_003860 | BANF1 | 0.257103761 | 0.110037488 | 0.527124137 | 1 | -0.239222705 | 0.119374317 | 0.3750164 | 1 |
| 87_1 | A_23_P22263 | NM_079837 | BANP | -0.262093408 | 0.087320571 | 0.482640286 | 1 | 0.271098965 | 0.0423255 | 0.2130863 | 1 |
| 289_73 | A_23_P67771 | NM_000465 | BARD1 | 0.09408697 | 0.454039341 | 0.832937561 | 1 | 0.053290694 | 0.651905309 | 0.8490979 | 1 |
| 287_12 | A_23_P128974 | NM_006399 | BATF | 0.605264091 | 0.020365822 | 0.306188729 | 1 | -0.898630405 | 1.48515E-05 | 0.0017961 | 0.16703 |
| 372_26 | A_23_P208706 | NM_138764 | BAX | -0.372915774 | 0.024807194 | 0.319019375 | 1 | -0.085134821 | 0.461463867 | 0.7240631 | 1 |
| 336_20 | A_23_P208706 | NM_138764 | BAX | -0.252101404 | 0.123416783 | 0.547584317 | 1 | -0.109300883 | 0.347185107 | 0.6330783 | 1 |
| 137_9 | A_23_P208706 | NM_138764 | BAX | -0.220967042 | 0.164285668 | 0.604083309 | 1 | -0.159220167 | 0.163843865 | 0.4390641 | 1 |
| 351_44 | A_23_P208706 | NM_138764 | BAX | -0.209093881 | 0.154397687 | 0.593561622 | 1 | -0.118511089 | 0.288147309 | 0.5792383 | 1 |
| 314_77 | A_23_P208706 | NM_138764 | BAX | -0.149842971 | 0.420038648 | 0.817246292 | 1 | -0.103752695 | 0.438137564 | 0.7091284 | 1 |
| 320_128 | A_23_P208706 | NM_138764 | BAX | -0.084977801 | 0.57103212 | 0.882279506 | 1 | -0.056970262 | 0.652563478 | 0.8496621 | 1 |
| 69_83 | A_23_P208706 | NM_138764 | BAX | -0.075529594 | 0.634074873 | 0.901795536 | 1 | -0.16131919 | 0.19945007 | 0.4829275 | 1 |
| 1_15 | A_23_P208706 | NM_138764 | BAX | -0.042133424 | 0.786344158 | 0.94877096 | 1 | -0.123195461 | 0.346542169 | 0.6325201 | 1 |
| 189_148 | A_23_P208706 | NM_138764 | BAX | 0.008133086 | 0.963144342 | 0.993286853 | 1 | -0.205559037 | 0.119318638 | 0.3750164 | 1 |
| 132_102 | A_23_P208706 | NM_138764 | BAX | 0.043647574 | 0.803274637 | 0.952359283 | 1 | -0.258411326 | 0.110010902 | 0.3596781 | 1 |
| 103_2 | A_23_P76799 | NM_013448 | BAZ1A | -0.097477223 | 0.475054021 | 0.839626091 | 1 | -0.01928812 | 0.874976343 | 0.9569555 | 1 |
| 36_8 | A_23_P76799 | NM_013448 | BAZ1A | -0.024285061 | 0.850615515 | 0.965069985 | 1 | -0.078173486 | 0.480227989 | 0.7383628 | 1 |
| 49_9 | A_23_P76799 | NM_013448 | BAZ1A | -0.017302818 | 0.896329248 | 0.975614984 | 1 | -0.066280321 | 0.567368096 | 0.79888 | 1 |
| 246_140 | A_23_P76799 | NM_013448 | BAZ1A | -0.00646774 | 0.957892146 | 0.99229078 | 1 | -0.152148394 | 0.200690235 | 0.4835834 | 1 |
| 93_162 | A_23_P76799 | NM_013448 | BAZ1A | 0.0076311 | 0.950414172 | 0.99027625 | 1 | -0.088355942 | 0.447203247 | 0.7160727 | 1 |
| 242_100 | A_23_P76799 | NM_013448 | BAZ1A | 0.050770261 | 0.709506045 | 0.929907687 | 1 | -0.108413789 | 0.358343181 | 0.6431991 | 1 |
| 134_126 | A_23_P76799 | NM_013448 | BAZ1A | 0.054410526 | 0.6566877 | 0.910923281 | 1 | -0.120237098 | 0.277648123 | 0.5687994 | 1 |
| 16_162 | A_23_P76799 | NM_013448 | BAZ1A | 0.103256916 | 0.424283926 | 0.819636344 | 1 | -0.070016021 | 0.564673543 | 0.7975491 | 1 |
| 10_162 | A_23_P76799 | NM_013448 | BAZ1A | 0.109059635 | 0.420825862 | 0.817356138 | 1 | -0.164789739 | 0.196178758 | 0.4783323 | 1 |
| 225_119 | A_23_P76799 | NM_013448 | BAZ1A | 0.140745335 | 0.288331327 | 0.727382494 | 1 | -0.198351846 | 0.081528175 | 0.3074941 | 1 |
| 176_143 | A_33_P3321577 | NM_032408 | BAZ1B | -0.143586127 | 0.350534905 | 0.771478158 | 1 | 0.137605745 | 0.32764953 | 0.6137313 | 1 |
| 112_164 | A_33_P3316983 | NM_001195304 | BBIP1 | -0.24757505 | 0.073566357 | 0.459518683 | 1 | -0.084548977 | 0.442969683 | 0.7127439 | 1 |
| 73_24 | A_23_P99967 | NM_033028 | BBS4 | 0.056185758 | 0.674920765 | 0.918338519 | 1 | 0.176495644 | 0.30804262 | 0.5962064 | 1 |
| 160_161 | A_23_P252642 | NM_152384 | BBS5 | -0.036965627 | 0.797948621 | 0.951453193 | 1 | -0.017193323 | 0.896624901 | 0.9633493 | 1 |
| 71_28 | A_23_P82351 | NM_198428 | BBS9 | -0.248548071 | 0.074948984 | 0.461185525 | 1 | 0.247675996 | 0.094227274 | 0.3327635 | 1 |
| 82_76 | A_23_P121356 | NM_020235 | BBX | -0.268909579 | 0.110035554 | 0.527124137 | 1 | 0.58747264 | 0.002119384 | 0.0346968 | 1 |
| 197_89 | A_33_P3287562 | NM_018844 | BCAP29 | -0.072821417 | 0.569163692 | 0.881307748 | 1 | 0.246002493 | 0.068988844 | 0.2792075 | 1 |
| 94_102 | A_32_P4364 | NM_005745 | BCAP31 | 0.096584084 | 0.443486088 | 0.829470233 | 1 | 0.797568149 | 4.06765E-05 | 0.0034398 | 0.45749 |
| 64_58 | A_21_P0011433 | NM_001139457 | BCAP31 | 0.190406883 | 0.40186851 | 0.805323603 | 1 | 0.583875436 | 0.043190221 | 0.2153193 | 1 |
| 101_47 | A_33_P3369034 | NM_001170714 | BCAR1 | 0.142396533 | 0.295488387 | 0.732603053 | 1 | -0.016180462 | 0.883071485 | 0.9596293 | 1 |
| 126_120 | A_23_P34930 | NM_005872 | BCAS2 | 0.414508952 | 0.012494906 | 0.265846757 | 1 | -0.157760684 | 0.174576599 | 0.4531417 | 1 |
| 7_85 | A_21_P0007218 | ENST00000463943 | BCAS4 | 0.151314782 | 0.566579301 | 0.880639801 | 1 | 0.125581027 | 0.618763123 | 0.8271011 | 1 |
| 349_31 | A_23_P338233 | NM_181708 | BCDIN3D | -0.059845482 | 0.635412263 | 0.902449007 | 1 | -0.074418029 | 0.606671448 | 0.8206139 | 1 |
| 58_145 | A_33_P3302305 | NM_000709 | BCKDHA | -0.256105444 | 0.088771157 | 0.486413374 | 1 | -0.012170092 | 0.916127601 | 0.9710383 | 1 |
| 87_105 | A_23_P3823 | NM_001122957 | BCKDK | 0.127128362 | 0.313419259 | 0.74498319 | 1 | -0.297489096 | 0.016835785 | 0.1245737 | 1 |
| 245_44 | A_23_P321703 | NM_004049 | BCL2A1 | 0.078096233 | 0.597906788 | 0.89144727 | 1 | 0.231093377 | 0.114054204 | 0.366122 | 1 |
| 167_133 | A_24_P122921 | NM_138621 | BCL2L11 | -0.008469858 | 0.945913935 | 0.99002169 | 1 | -0.093110834 | 0.524294726 | 0.7692073 | 1 |
| 17_55 | A_33_P3251932 | NM_207002 | BCL2L11 | 0.0307925 | 0.802569974 | 0.952164507 | 1 | 0.30122101 | 0.040422512 | 0.207216 | 1 |
| 269_125 | A_33_P3217123 | NM_138639 | BCL2L12 | -0.023956285 | 0.850342546 | 0.965069985 | 1 | 0.062933101 | 0.565098777 | 0.7976902 | 1 |
| 365_40 | A_33_P3244322 | NM_001282521 | BCL2L12 | 0.038755168 | 0.766743113 | 0.945351992 | 1 | -0.019320592 | 0.897255977 | 0.9635078 | 1 |
| 81_27 | A_23_P418373 | NM_004050 | BCL2L2 | -0.11933156 | 0.350714114 | 0.771478158 | 1 | -0.035230411 | 0.78761139 | 0.915867 | 1 |
| 356_18 | A_23_P4662 | NM_005178 | BCL3 | -0.488082981 | 0.004164182 | 0.189007893 | 1 | -0.235997801 | 0.156478665 | 0.4272677 | 1 |
| 298_154 | A_23_P118289 | NM_004765 | BCL7C | 0.033405256 | 0.799778362 | 0.951548003 | 1 | -0.144877293 | 0.245185678 | 0.5323559 | 1 |
| 251_94 | A_33_P3405957 | NM_182557 | BCL9L | -0.071654604 | 0.65379549 | 0.909297483 | 1 | 0.07360059 | 0.516648581 | 0.7632663 | 1 |
| 261_37 | A_24_P89512 | NM_014739 | BCLAF1 | -0.213146158 | 0.202157599 | 0.64879651 | 1 | -0.296305674 | 0.032000983 | 0.1824202 | 1 |
| 342_124 | A_23_P111343 | NM_014739 | BCLAF1 | -0.056685234 | 0.700266439 | 0.925289905 | 1 | -0.178127449 | 0.209847731 | 0.4940669 | 1 |
| 113_83 | A_23_P147495 | NM_021946 | BCORL1 | -0.195858948 | 0.17683153 | 0.616374573 | 1 | 0.416477518 | 0.00443353 | 0.055964 | 1 |
| 108_69 | A_24_P873764 | NM_004327 | BCR | -0.209761439 | 0.150810018 | 0.587524032 | 1 | -0.323470219 | 0.021458029 | 0.1431902 | 1 |
| 342_74 | A_23_P154086 | NM_004328 | BCS1L | 0.107180543 | 0.443764668 | 0.829470233 | 1 | 0.389655353 | 0.005856319 | 0.0669736 | 1 |
| 175_58 | A_33_P3252359 | NM_203314 | BDH1 | -0.115515145 | 0.419162124 | 0.81680004 | 1 | -0.340272888 | 0.062281025 | 0.2641307 | 1 |
| 155_163 | A_23_P89410 | NM_003766 | BECN1 | 0.125478424 | 0.434842866 | 0.825069738 | 1 | 0.044237322 | 0.688153536 | 0.8689581 | 1 |
| 359_29 | A_23_P359854 | NM_001080450 | BEND3 | -0.191317538 | 0.225128639 | 0.669559835 | 1 | 0.101822904 | 0.507573242 | 0.758225 | 1 |
| 172_65 | A_33_P3245178 | NM_001168399 | BEX2 | 0.267860567 | 0.187689412 | 0.630397286 | 1 | 1.051718751 | 7.37766E-06 | 0.0013829 | 0.08298 |
| 63_60 | A_23_P45524 | NM_014380 | BEX3 | 0.075622697 | 0.590749233 | 0.88758105 | 1 | 0.546377034 | 0.001859256 | 0.0323701 | 1 |
| 12_47 | A_23_P65963 | NM_016561 | BFAR | 0.113577202 | 0.370016409 | 0.783919981 | 1 | -0.057095265 | 0.760826973 | 0.9042308 | 1 |
| 210_53 | A_23_P109171 | NM_001195 | BFSP1 | 0.079931734 | 0.691077475 | 0.923085623 | 1 | 0.362492677 | 0.016368898 | 0.1228159 | 1 |
| 300_23 | A_24_P336551 | NM_199173 | BGLAP | -0.139474527 | 0.310560402 | 0.743121622 | 1 | 0.012404173 | 0.922676378 | 0.9722916 | 1 |
| 331_155 | A_24_P57898 | NM_080606 | BHLHE23 | -0.069272045 | 0.708682671 | 0.929497449 | 1 | 0.263392568 | 0.336394895 | 0.6226527 | 1 |
| 360_53 | A_24_P187948 | NM_197966 | BID | 0.002222309 | 0.988914268 | 0.99652193 | 1 | -0.295935966 | 0.034203311 | 0.1895932 | 1 |
| 102_42 | A_33_P3227041 | NM_197966 | BID | 0.023837166 | 0.859954128 | 0.966684616 | 1 | -0.364249824 | 0.008655609 | 0.0848733 | 1 |
| 272_33 | A_23_P118815 | NM_001012271 | BIRC5 | -0.364948124 | 0.028904942 | 0.336406827 | 1 | -0.044010264 | 0.713818941 | 0.8818455 | 1 |
| 267_31 | A_23_P118815 | NM_001012271 | BIRC5 | -0.353688009 | 0.023528693 | 0.316710379 | 1 | -0.050451317 | 0.65933904 | 0.854816 | 1 |
| 257_37 | A_23_P118815 | NM_001012271 | BIRC5 | -0.324878665 | 0.049264657 | 0.403864096 | 1 | -0.08884791 | 0.405688611 | 0.6832631 | 1 |
| 373_100 | A_23_P118815 | NM_001012271 | BIRC5 | -0.224820267 | 0.106101098 | 0.520758893 | 1 | -0.054038794 | 0.613649565 | 0.8240856 | 1 |
| 78_114 | A_23_P118815 | NM_001012271 | BIRC5 | -0.11342698 | 0.478766164 | 0.84100638 | 1 | -0.091067308 | 0.482481924 | 0.7408156 | 1 |
| 146_130 | A_23_P118815 | NM_001012271 | BIRC5 | -0.108686593 | 0.447352162 | 0.830485224 | 1 | -0.105929158 | 0.332730016 | 0.6193668 | 1 |
| 269_148 | A_23_P118815 | NM_001012271 | BIRC5 | -0.104289962 | 0.473048068 | 0.838824187 | 1 | -0.161749976 | 0.155596411 | 0.4256919 | 1 |
| 163_31 | A_23_P118815 | NM_001012271 | BIRC5 | -0.089480746 | 0.559841722 | 0.87762088 | 1 | -0.115442555 | 0.357104049 | 0.6417944 | 1 |
| 114_134 | A_23_P118815 | NM_001012271 | BIRC5 | -0.076172286 | 0.602255028 | 0.89334493 | 1 | -0.187512133 | 0.130294048 | 0.3913869 | 1 |
| 22_163 | A_23_P118815 | NM_001012271 | BIRC5 | -0.0271088 | 0.848426145 | 0.965069985 | 1 | -0.191964968 | 0.179405896 | 0.4577014 | 1 |
| 92_40 | A_23_P105833 | NM_017693 | BIVM | -2.95627E-06 | 0.999983039 | 0.999983039 | 1 | -0.246319989 | 0.0496531 | 0.2326868 | 1 |
| 4_113 | A_23_P88630 | NM_000057 | BLM | 0.297994512 | 0.119520439 | 0.542056525 | 1 | 0.196265995 | 0.166633311 | 0.4431817 | 1 |
| 51_154 | A_33_P3388870 | NM_001487 | BLOC1S1 | -0.371786092 | 0.012283461 | 0.265846757 | 1 | 0.069240927 | 0.6548435 | 0.8513931 | 1 |
| 232_135 | A_33_P3329597 | NM_212550 | BLOC1S3 | -0.19566141 | 0.287179368 | 0.72693776 | 1 | -0.024366565 | 0.848602013 | 0.9444119 | 1 |
| 92_161 | A_23_P69670 | NM_018366 | BLOC1S4 | -0.064849565 | 0.641300077 | 0.906091109 | 1 | -0.193503818 | 0.129639621 | 0.3906905 | 1 |
| 286_39 | A_24_P106591 | NM_201280 | BLOC1S5 | -0.20612486 | 0.130480909 | 0.559492585 | 1 | 0.316635387 | 0.047097517 | 0.2251193 | 1 |
| 31_5 | A_33_P3374833 | NM_012388 | BLOC1S6 | -0.070946371 | 0.659892593 | 0.911868366 | 1 | 0.038466339 | 0.788264933 | 0.9163904 | 1 |
| 178_82 | A_23_P71148 | NM_000712 | BLVRA | -0.141591038 | 0.31864861 | 0.750101906 | 1 | 0.0300118 | 0.859218207 | 0.9494623 | 1 |
| 344_73 | A_33_P3360728 | NM_000713 | BLVRB | -0.692310077 | 0.000522733 | 0.078653824 | 1 | 1.131077135 | 7.23537E-05 | 0.0046665 | 0.81376 |
| 377_83 | A_33_P3365878 | NM_001720 | BMP8B | -0.116382289 | 0.539076819 | 0.869166909 | 1 | 0.104186977 | 0.585973056 | 0.8110447 | 1 |
| 218_23 | A_19_P00805548 | NM_004329 | BMPR1A | -0.307954963 | 0.033702392 | 0.354850938 | 1 | 0.138414887 | 0.357647613 | 0.6423607 | 1 |
| 345_81 | A_33_P3219256 | NM_004329 | BMPR1A | -0.164516932 | 0.195994573 | 0.642117721 | 1 | 0.084581952 | 0.562962579 | 0.7958803 | 1 |
| 334_50 | A_24_P263672 | NM_014753 | BMS1 | 0.006119781 | 0.965305189 | 0.993672746 | 1 | 0.304264281 | 0.078550942 | 0.3019352 | 1 |
| 302_59 | A_23_P109420 | NM_014753 | BMS1 | 0.053922536 | 0.733695178 | 0.937867226 | 1 | 0.113222004 | 0.384670429 | 0.6656373 | 1 |
| 204_11 | A_33_P3400653 | ENST00000267859 | BNIP2 | -0.201700336 | 0.124205558 | 0.549010199 | 1 | -0.182534635 | 0.133520027 | 0.396819 | 1 |
| 45_85 | A_33_P3227467 | NM_004330 | BNIP2 | 0.043462428 | 0.716519981 | 0.933387402 | 1 | 0.109943579 | 0.404588451 | 0.6827157 | 1 |
| 265_26 | A_32_P93852 | NM_138369 | BOD1 | -0.048646583 | 0.722584698 | 0.935844012 | 1 | -0.147136586 | 0.202961401 | 0.485558 | 1 |
| 100_152 | A_33_P3407195 | NM_148894 | BOD1L1 | 0.02907965 | 0.894345953 | 0.975081715 | 1 | 0.209562333 | 0.118760818 | 0.3739065 | 1 |
| 92_13 | A_33_P3234487 | NM_001039182 | BOLA2B | -0.307457972 | 0.046792461 | 0.399349089 | 1 | 0.096298014 | 0.363489205 | 0.6477837 | 1 |
| 266_90 | A_32_P53486 | NM_001039182 | BOLA2B | -0.038000982 | 0.792062139 | 0.94877096 | 1 | 0.032153531 | 0.796476939 | 0.9198135 | 1 |
| 264_118 | A_33_P3234490 | NM_001039182 | BOLA2B | 0.017613102 | 0.899311922 | 0.97618995 | 1 | -0.06550228 | 0.561558641 | 0.7956475 | 1 |
| 135_9 | A_24_P77364 | NM_212552 | BOLA3 | -0.348465317 | 0.015595682 | 0.283139497 | 1 | 0.499694923 | 0.003197856 | 0.0458169 | 1 |
| 185_2 | A_33_P3368301 | NM_212552 | BOLA3 | -0.248622337 | 0.214244118 | 0.660776824 | 1 | 0.610846477 | 0.018518334 | 0.1324909 | 1 |
| 89_20 | A_22_P00000427 | NR_045637 | BOLA3-AS1 | -0.154341885 | 0.287543299 | 0.72693776 | 1 | 0.024305194 | 0.884425234 | 0.9603331 | 1 |
| 192_157 | A_23_P43800 | NM_015201 | BOP1 | 0.291052928 | 0.05799223 | 0.425190813 | 1 | -0.284424298 | 0.051786983 | 0.2380254 | 1 |
| 168_150 | A_23_P25626 | NM_024808 | BORA | 0.159864501 | 0.286577008 | 0.726625307 | 1 | -0.252387657 | 0.059905639 | 0.2574866 | 1 |
| 40_82 | A_24_P393470 | NM_001145783 | BORCS8 | -0.105824317 | 0.408776336 | 0.80997289 | 1 | -0.242104141 | 0.114097757 | 0.366122 | 1 |
| 224_106 | A_23_P70843 | NM_199186 | BPGM | 0.203843233 | 0.133603762 | 0.563559912 | 1 | -0.20482783 | 0.113551648 | 0.365803 | 1 |
| 82_60 | A_23_P42087 | NM_004332 | BPHL | -0.053973615 | 0.714217954 | 0.932448811 | 1 | 0.187802415 | 0.171092064 | 0.4488494 | 1 |
| 108_5 | A_23_P251893 | NM_152743 | BRAT1 | -0.38212968 | 0.026879385 | 0.329142336 | 1 | 0.135397754 | 0.624070671 | 0.8308399 | 1 |
| 163_34 | A_23_P207400 | NM_007300 | BRCA1 | -0.104795997 | 0.430660071 | 0.821617604 | 1 | 0.131323696 | 0.30434434 | 0.5936456 | 1 |
| 231_14 | A_23_P207400 | NM_007300 | BRCA1 | -0.075746349 | 0.576431429 | 0.883311119 | 1 | 0.10328997 | 0.392916791 | 0.6732649 | 1 |
| 43_116 | A_23_P207400 | NM_007300 | BRCA1 | -0.058536654 | 0.672791841 | 0.91758711 | 1 | 0.06544055 | 0.643163326 | 0.8447892 | 1 |
| 285_116 | A_23_P207400 | NM_007300 | BRCA1 | 0.004917249 | 0.971628274 | 0.994619938 | 1 | 0.085111008 | 0.491621887 | 0.7472998 | 1 |
| 249_137 | A_23_P207400 | NM_007300 | BRCA1 | 0.018572273 | 0.892025103 | 0.974513338 | 1 | 0.149583388 | 0.244270481 | 0.531909 | 1 |
| 186_163 | A_23_P207400 | NM_007300 | BRCA1 | 0.026605477 | 0.845131084 | 0.964376494 | 1 | 0.058243258 | 0.670529782 | 0.8610925 | 1 |
| 265_70 | A_23_P207400 | NM_007300 | BRCA1 | 0.040943398 | 0.749389558 | 0.941597731 | 1 | 0.167730337 | 0.181947607 | 0.4606308 | 1 |
| 263_5 | A_33_P3303940 | NM_024332 | BRCC3 | -0.215398715 | 0.109226055 | 0.526549982 | 1 | 0.549436503 | 0.001557456 | 0.0285934 | 1 |
| 229_54 | A_23_P217659 | NM_001018055 | BRCC3 | -0.087361568 | 0.520346668 | 0.862078486 | 1 | 0.958180375 | 5.83606E-05 | 0.0040769 | 0.65638 |
| 222_113 | A_23_P166536 | NM_014577 | BRD1 | 0.111445952 | 0.427227229 | 0.820875237 | 1 | 0.082412486 | 0.632319441 | 0.8363482 | 1 |
| 354_132 | A_24_P307014 | NM_005104 | BRD2 | -0.127638193 | 0.508367285 | 0.856078091 | 1 | 0.332638803 | 0.039119228 | 0.2035032 | 1 |
| 282_20 | A_33_P3240674 | NM_007371 | BRD3 | -0.208166253 | 0.136033441 | 0.566743973 | 1 | 0.046043067 | 0.691724012 | 0.871307 | 1 |
| 209_129 | A_33_P3373750 | NM_014299 | BRD4 | 0.229174043 | 0.15526933 | 0.59435532 | 1 | -0.264542658 | 0.065907135 | 0.2725212 | 1 |
| 297_111 | A_32_P18824 | NM_013263 | BRD7 | 0.10683525 | 0.500223776 | 0.852388591 | 1 | 0.129821336 | 0.275104162 | 0.5666843 | 1 |
| 132_150 | A_23_P145016 | NM_006696 | BRD8 | -0.304466359 | 0.070613054 | 0.455143645 | 1 | 0.066787187 | 0.618336423 | 0.8269473 | 1 |
| 277_97 | A_33_P3413463 | NM_001519 | BRF1 | -0.126971416 | 0.323286094 | 0.753815848 | 1 | 0.046575472 | 0.704430141 | 0.8766152 | 1 |
| 316_34 | A_33_P3363016 | NM_001242790 | BRF1 | -0.046060463 | 0.7835814 | 0.94877096 | 1 | -0.105879399 | 0.578201535 | 0.8054289 | 1 |
| 108_14 | A_33_P3348244 | NM_015379 | BRI3 | -0.14786751 | 0.320580188 | 0.752322837 | 1 | 0.157411026 | 0.312194321 | 0.6008481 | 1 |
| 74_125 | A_23_P122915 | NM_015379 | BRI3 | -0.059425807 | 0.688392229 | 0.922145825 | 1 | 0.169777796 | 0.217325238 | 0.5038667 | 1 |
| 16_108 | A_23_P252855 | NM_018321 | BRIX1 | 0.167999933 | 0.216800559 | 0.662917773 | 1 | -0.205885533 | 0.118600131 | 0.3737449 | 1 |
| 103_62 | A_23_P420361 | NM_018462 | BRK1 | -0.077506341 | 0.627677466 | 0.900268731 | 1 | -0.114871886 | 0.368476391 | 0.6525357 | 1 |
| 64_131 | A_19_P00802936 | NM_018462 | BRK1 | -0.045057418 | 0.730445643 | 0.936838487 | 1 | -0.08154795 | 0.528587175 | 0.771442 | 1 |
| 214_108 | A_23_P75500 | NM_001024957 | BRMS1 | 0.346503729 | 0.023579708 | 0.316710379 | 1 | -0.441938342 | 0.011403054 | 0.0993485 | 1 |
| 50_49 | A_23_P11705 | NM_018045 | BSDC1 | 0.03507519 | 0.780007752 | 0.94811958 | 1 | -0.00625641 | 0.963351538 | 0.9873043 | 1 |
| 337_35 | A_24_P134266 | NM_001728 | BSG | -0.316951851 | 0.063892497 | 0.437947257 | 1 | -0.042192182 | 0.805565597 | 0.9240105 | 1 |
| 286_145 | A_23_P205830 | NM_025238 | BTBD1 | 0.280277319 | 0.169209308 | 0.608706095 | 1 | 0.241453922 | 0.05180987 | 0.2380333 | 1 |
| 149_158 | A_23_P99614 | NM_033271 | BTBD6 | -0.018996294 | 0.873971461 | 0.970669135 | 1 | -0.223895605 | 0.085670193 | 0.314529 | 1 |
| 93_53 | A_32_P94722 | NM_052893 | BTBD9 | -0.395816335 | 0.01180833 | 0.26314611 | 1 | 0.108973124 | 0.409454484 | 0.6853899 | 1 |
| 218_18 | A_24_P183264 | NM_001037637 | BTF3 | -0.01513257 | 0.914551748 | 0.980806062 | 1 | -0.073065372 | 0.682792786 | 0.8679746 | 1 |
| 162_19 | A_23_P105900 | NR_026983 | BTF3P11 | 0.265586162 | 0.257452543 | 0.699370343 | 1 | -0.366379138 | 0.168420551 | 0.4455094 | 1 |
| 93_140 | A_23_P105900 | NR_026983 | BTF3P11 | 0.322018481 | 0.196636918 | 0.642839304 | 1 | -0.34890412 | 0.175321868 | 0.4542244 | 1 |
| 144_160 | A_23_P105900 | NR_026983 | BTF3P11 | 0.326271543 | 0.199225556 | 0.645792532 | 1 | -0.305087969 | 0.204510336 | 0.4872376 | 1 |
| 107_80 | A_23_P105900 | NR_026983 | BTF3P11 | 0.366321181 | 0.177075557 | 0.616374573 | 1 | -0.214474165 | 0.419314356 | 0.6952135 | 1 |
| 254_61 | A_23_P105900 | NR_026983 | BTF3P11 | 0.38958447 | 0.156035619 | 0.59549684 | 1 | -0.298704665 | 0.267382344 | 0.5578226 | 1 |
| 363_164 | A_23_P105900 | NR_026983 | BTF3P11 | 0.390780984 | 0.142075523 | 0.575849374 | 1 | -0.323061869 | 0.181258289 | 0.4597471 | 1 |
| 44_81 | A_23_P105900 | NR_026983 | BTF3P11 | 0.415614052 | 0.085425492 | 0.478901677 | 1 | -0.339716596 | 0.195487138 | 0.4776545 | 1 |
| 309_79 | A_23_P105900 | NR_026983 | BTF3P11 | 0.421032324 | 0.118391098 | 0.540937779 | 1 | -0.341391332 | 0.23120534 | 0.5195539 | 1 |
| 260_108 | A_23_P105900 | NR_026983 | BTF3P11 | 0.452212654 | 0.080157718 | 0.468656078 | 1 | -0.379493435 | 0.194957576 | 0.4770861 | 1 |
| 291_144 | A_23_P105900 | NR_026983 | BTF3P11 | 0.524623903 | 0.050200312 | 0.406002234 | 1 | -0.318121632 | 0.255119699 | 0.5439491 | 1 |
| 259_49 | A_22_P00013430 | NM_001731 | BTG1 | 0.094229167 | 0.65397309 | 0.909440661 | 1 | -0.144975665 | 0.449626856 | 0.7174001 | 1 |
| 221_77 | A_23_P80068 | NM_006806 | BTG3 | 0.542651792 | 0.008572926 | 0.235745789 | 1 | 0.305653897 | 0.037548908 | 0.1994693 | 1 |
| 157_119 | A_24_P252078 | NM_007047 | BTN3A2 | 0.033264788 | 0.807924835 | 0.953806288 | 1 | 0.003158595 | 0.976675783 | 0.991684 | 1 |
| 363_107 | A_23_P124417 | NM_004336 | BUB1 | -0.010226547 | 0.953236919 | 0.990760353 | 1 | 0.014222501 | 0.923622418 | 0.9724753 | 1 |
| 253_103 | A_23_P163481 | NM_001211 | BUB1B | 0.071570493 | 0.609975234 | 0.89709602 | 1 | -0.423024076 | 0.045901921 | 0.2216655 | 1 |
| 30_52 | A_23_P320658 | NM_004725 | BUB3 | -0.161567716 | 0.264895073 | 0.707490253 | 1 | 0.228860985 | 0.052558639 | 0.2403933 | 1 |
| 223_29 | A_23_P52826 | NM_032725 | BUD13 | 0.166731316 | 0.436586443 | 0.826037245 | 1 | -0.099764196 | 0.615602317 | 0.8253283 | 1 |
| 317_11 | A_23_P31602 | NM_003910 | BUD31 | 0.044794543 | 0.768909667 | 0.945351992 | 1 | -0.153998623 | 0.279433081 | 0.5704817 | 1 |
| 304_62 | A_33_P3318564 | lnc-LIN28B-1:4 | BVES-AS1 | 0.010190546 | 0.933856953 | 0.986418035 | 1 | -0.057346349 | 0.63974516 | 0.8424322 | 1 |
| 146_138 | A_23_P145197 | NM_004053 | BYSL | 0.280753236 | 0.099722127 | 0.506806404 | 1 | 0.085327686 | 0.598417397 | 0.8174528 | 1 |
| 298_32 | A_33_P3334548 | NM_001207068 | BZW1 | -0.005498992 | 0.967607648 | 0.994075478 | 1 | 0.525408031 | 0.000806841 | 0.0197273 | 1 |
| 269_21 | A_24_P713668 | NM_014670 | BZW1 | 0.09962052 | 0.466490426 | 0.83630364 | 1 | 0.341830042 | 0.04519002 | 0.2200434 | 1 |
| 276_51 | A_23_P157215 | NM_014038 | BZW2 | 0.135957425 | 0.435227014 | 0.825069738 | 1 | -0.303258284 | 0.036546588 | 0.1962504 | 1 |
| 331_164 | A_33_P3216237 | NM_001159767 | BZW2 | 0.14835951 | 0.297221552 | 0.733788339 | 1 | -0.349755165 | 0.007992024 | 0.0809058 | 1 |
| 78_43 | A_23_P369328 | NM_145306 | C10orf35 | -0.796352566 | 0.002229256 | 0.146096261 | 1 | 0.391090806 | 0.006041264 | 0.0681506 | 1 |
| 256_36 | A_24_P152404 | ENST00000311122 | C10orf76 | -0.235235356 | 0.074073855 | 0.459856398 | 1 | -0.100778967 | 0.46012957 | 0.7224734 | 1 |
| 90_17 | A_23_P86504 | NM_024541 | C10orf76 | -0.149819757 | 0.276017717 | 0.716856302 | 1 | -0.207101506 | 0.105932159 | 0.3531119 | 1 |
| 316_68 | A_33_P3360823 | NM_024942 | C10orf88 | -0.046365977 | 0.727628702 | 0.936348816 | 1 | 0.11632158 | 0.40931176 | 0.685253 | 1 |
| 205_157 | A_23_P150350 | NM_022761 | C11orf1 | -0.021946451 | 0.873176415 | 0.970202812 | 1 | 0.471726464 | 0.005646023 | 0.0656 | 1 |
| 86_40 | A_23_P12911 | NM_022338 | C11orf24 | -0.027503356 | 0.851316964 | 0.965069985 | 1 | 0.016402701 | 0.885613549 | 0.9608259 | 1 |
| 88_75 | A_23_P340318 | NM_170746 | C11orf31 | -0.030933422 | 0.799188218 | 0.951548003 | 1 | 0.083614791 | 0.521048967 | 0.766932 | 1 |
| 216_58 | A_33_P3306983 | NM_170746 | C11orf31 | 0.178350188 | 0.201444159 | 0.648025996 | 1 | -0.01418115 | 0.903927336 | 0.9663475 | 1 |
| 41_51 | A_33_P3292840 | NM_001003678 | C11orf49 | -0.258230839 | 0.085886556 | 0.479280818 | 1 | 0.101091558 | 0.367078128 | 0.6511873 | 1 |
| 141_90 | A_23_P147605 | NM_001003676 | C11orf49 | 0.016927173 | 0.889936194 | 0.973803066 | 1 | -0.195343263 | 0.142586809 | 0.4096229 | 1 |
| 196_87 | A_33_P3309636 | NM_014267 | C11orf58 | 0.177060969 | 0.304587414 | 0.738652994 | 1 | 0.088966914 | 0.554607487 | 0.7902788 | 1 |
| 233_76 | A_23_P150238 | NM_031450 | C11orf68 | 0.008532009 | 0.957346281 | 0.992062116 | 1 | -0.125169597 | 0.254251761 | 0.5427946 | 1 |
| 307_43 | A_33_P3256902 | ENST00000333139 | C11orf72 | -0.11865345 | 0.367810801 | 0.782103539 | 1 | 0.278158973 | 0.113685728 | 0.3658436 | 1 |
| 311_143 | A_23_P139396 | NM_016401 | C11orf73 | 0.242902513 | 0.097381018 | 0.500605281 | 1 | -0.390017263 | 0.031650871 | 0.1812512 | 1 |
| 104_158 | A_24_P252846 | NM_138787 | C11orf74 | -0.19489271 | 0.135680765 | 0.566743973 | 1 | 0.116478982 | 0.318357525 | 0.6051097 | 1 |
| 183_74 | A_33_P3285354 | NM_001144936 | C11orf95 | -0.118923498 | 0.541785078 | 0.869568619 | 1 | -0.876161111 | 5.22691E-05 | 0.0038676 | 0.58787 |
| 161_6 | A_23_P116797 | NM_021640 | C12orf10 | 0.019527195 | 0.873228982 | 0.970202812 | 1 | -0.396071839 | 0.022468991 | 0.1479559 | 1 |
| 18_72 | A_32_P46765 | NM_001009894 | C12orf29 | 0.096791348 | 0.476678418 | 0.840656336 | 1 | -0.175487554 | 0.162975983 | 0.437484 | 1 |
| 26_48 | A_23_P25433 | NM_020374 | C12orf4 | 0.184216287 | 0.18169671 | 0.622415463 | 1 | 0.243904996 | 0.067725424 | 0.2768376 | 1 |
| 252_122 | A_23_P128532 | NM_152318 | C12orf45 | 0.221950505 | 0.126186122 | 0.551701995 | 1 | -0.006412779 | 0.957256975 | 0.9851102 | 1 |
| 98_131 | A_23_P350551 | NM_138425 | C12orf57 | -0.325172442 | 0.026993646 | 0.329165762 | 1 | 0.241218105 | 0.044020764 | 0.2169301 | 1 |
| 153_104 | A_32_P11894 | NM_152269 | C12orf65 | 0.198313806 | 0.233118602 | 0.679464072 | 1 | -0.022189072 | 0.87694993 | 0.9573923 | 1 |
| 99_124 | A_24_P769672 | NM_001135570 | C12orf73 | 0.186887861 | 0.174064808 | 0.614243681 | 1 | 0.189817506 | 0.144538286 | 0.412176 | 1 |
| 170_132 | A_32_P163089 | NM_001145199 | C12orf75 | -0.176649298 | 0.295652752 | 0.732603053 | 1 | 0.164763546 | 0.160115925 | 0.4332151 | 1 |
| 330_90 | A_23_P14482 | NM_016039 | C14orf166 | 0.338408246 | 0.016836707 | 0.29259326 | 1 | 0.094261789 | 0.421477348 | 0.6970086 | 1 |
| 185_64 | A_23_P163117 | NM_024644 | C14orf169 | 0.235333619 | 0.089949195 | 0.48830708 | 1 | -0.02521308 | 0.85201164 | 0.9460316 | 1 |
| 18_146 | A_33_P3289780 | NM_001127393 | C14orf2 | 0.025432249 | 0.871982307 | 0.969992795 | 1 | -0.326984112 | 0.036659662 | 0.1966196 | 1 |
| 367_92 | A_33_P3256858 | NM_001134875 | C14orf80 | -0.411563689 | 0.009050146 | 0.240080283 | 1 | -0.076327993 | 0.601282649 | 0.8185598 | 1 |
| 250_10 | A_23_P431381 | NM_001134875 | C14orf80 | -0.34458992 | 0.016464323 | 0.290258157 | 1 | -0.159732417 | 0.220528458 | 0.5080791 | 1 |
| 224_117 | A_23_P48581 | NM_021944 | C14orf93 | -0.197621153 | 0.262956177 | 0.705424389 | 1 | 0.550431678 | 0.010670743 | 0.0954005 | 1 |
| 133_103 | A_23_P37514 | NM_015492 | C15orf39 | 0.429425088 | 0.020864372 | 0.306261998 | 1 | -0.43611453 | 0.019597224 | 0.1368156 | 1 |
| 376_12 | A_21_P0000026 | NM_001160115 | C15orf40 | 0.030132898 | 0.812380956 | 0.954976822 | 1 | 0.125267086 | 0.345637861 | 0.6320039 | 1 |
| 82_104 | A_23_P163467 | NM_207380 | C15orf52 | -0.086732201 | 0.662177585 | 0.913018245 | 1 | 0.216373048 | 0.274808547 | 0.5661791 | 1 |
| 317_150 | A_33_P3219055 | NM_001143936 | C15orf61 | 0.312125773 | 0.039074343 | 0.374228433 | 1 | 0.130527094 | 0.30700024 | 0.5957341 | 1 |
| 369_136 | A_33_P3344451 | NM_032366 | C16orf13 | 0.037734721 | 0.776036928 | 0.946647457 | 1 | -0.261277163 | 0.097287044 | 0.339338 | 1 |
| 274_164 | A_23_P54891 | NM_022744 | C16orf58 | 0.158992505 | 0.258338328 | 0.700376544 | 1 | -0.045768094 | 0.703829526 | 0.8766152 | 1 |
| 63_84 | A_23_P26557 | NM_025108 | C16orf59 | -0.053537428 | 0.693666927 | 0.924234842 | 1 | 0.052492379 | 0.641253383 | 0.8434308 | 1 |
| 36_35 | A_23_P206382 | NM_025187 | C16orf70 | 0.103527461 | 0.545090697 | 0.871327471 | 1 | 0.161073482 | 0.158856201 | 0.4312264 | 1 |
| 198_125 | A_32_P4403 | NM_014117 | C16orf72 | 0.002095477 | 0.988132818 | 0.99652193 | 1 | -0.27721999 | 0.061205774 | 0.2611462 | 1 |
| 176_81 | A_33_P3258191 | NM_001272051 | C16orf91 | 0.208540349 | 0.108003663 | 0.524183516 | 1 | -0.131932347 | 0.334683276 | 0.6209473 | 1 |
| 123_1 | A_23_P89030 | NM_001195124 | C16orf95 | -0.134632923 | 0.312138595 | 0.743773338 | 1 | 0.111990059 | 0.319309862 | 0.6059183 | 1 |
| 290_57 | A_23_P141520 | NM_174893 | C17orf49 | 0.0697845 | 0.633799773 | 0.90176931 | 1 | -0.259090917 | 0.067518317 | 0.2766473 | 1 |
| 126_149 | A_19_P00322906 | NM_001113434 | C17orf51 | 0.073997929 | 0.601496858 | 0.893153916 | 1 | 0.038900065 | 0.731757027 | 0.8913756 | 1 |
| 15_55 | A_24_P59607 | NM_001113434 | C17orf51 | 0.089978522 | 0.55803266 | 0.877108086 | 1 | 0.519580764 | 0.00077135 | 0.0192965 | 1 |
| 350_89 | A_32_P524614 | NM_175734 | C17orf74 | -0.134815584 | 0.278784854 | 0.719198817 | 1 | 0.092991623 | 0.440315842 | 0.7107107 | 1 |
| 286_141 | A_23_P207927 | NM_001288770 | C17orf80 | 0.157613779 | 0.246480153 | 0.691935623 | 1 | -0.009278792 | 0.946153595 | 0.9810445 | 1 |
| 11_51 | A_33_P3417195 | NM_203425 | C17orf82 | -0.162829209 | 0.239323392 | 0.684298985 | 1 | -0.293909612 | 0.024038635 | 0.1546423 | 1 |
| 155_9 | A_24_P653603 | NM_001086521 | C17orf89 | -0.122644383 | 0.435371262 | 0.825069738 | 1 | -0.321690524 | 0.013993407 | 0.1123368 | 1 |
| 307_31 | A_33_P3410935 | NM_001086521 | C17orf89 | -0.098726347 | 0.477038671 | 0.840874705 | 1 | -0.335508792 | 0.020328275 | 0.1388173 | 1 |
| 198_29 | A_33_P3393766 | NM_001130677 | C17orf96 | -0.14917791 | 0.289040467 | 0.727382494 | 1 | 0.127112031 | 0.321431006 | 0.6081989 | 1 |
| 13_51 | A_23_P50052 | XR_109479 | C18orf12 | 0.099290519 | 0.523712465 | 0.863661195 | 1 | 0.360736867 | 0.071134937 | 0.2840095 | 1 |
| 217_127 | A_23_P101237 | NM_031446 | C18orf21 | 0.174385194 | 0.191240295 | 0.635312046 | 1 | -0.141365643 | 0.238180073 | 0.5262263 | 1 |
| 106_75 | A_33_P3300680 | NM_001256046 | C19orf12 | -0.024573128 | 0.836491801 | 0.961943507 | 1 | 0.158821025 | 0.180341531 | 0.4589599 | 1 |
| 159_135 | A_33_P3839760 | NM_017914 | C19orf24 | -0.079846965 | 0.567842372 | 0.881090627 | 1 | 0.024078147 | 0.865378093 | 0.9523393 | 1 |
| 118_23 | A_23_P424597 | NM_152482 | C19orf25 | -0.071273621 | 0.570583367 | 0.882222216 | 1 | -0.179112997 | 0.127174612 | 0.3880638 | 1 |
| 324_136 | A_23_P208788 | NM_033520 | C19orf33 | -0.092606677 | 0.478546111 | 0.840976446 | 1 | 0.298822875 | 0.057718964 | 0.2535541 | 1 |
| 284_26 | A_23_P107801 | NM_032207 | C19orf44 | -0.291456762 | 0.058071765 | 0.425190813 | 1 | 0.132413664 | 0.314037122 | 0.6015453 | 1 |
| 154_85 | A_33_P3288754 | NM_001290149 | C19orf48 | 0.382573115 | 0.013819878 | 0.271538317 | 1 | -0.121531251 | 0.479528081 | 0.7379929 | 1 |
| 145_129 | A_23_P310532 | NM_138358 | C19orf52 | 0.29764987 | 0.038809215 | 0.374228433 | 1 | 0.022466529 | 0.845981698 | 0.9428953 | 1 |
| 70_42 | A_23_P67589 | NM_001100418 | C19orf60 | -0.157622844 | 0.286051983 | 0.726263094 | 1 | -0.282003499 | 0.036229944 | 0.1954332 | 1 |
| 16_43 | A_33_P3214705 | NM_001100418 | C19orf60 | -0.037713666 | 0.794274935 | 0.949358051 | 1 | -0.060124713 | 0.664093799 | 0.8566422 | 1 |
| 353_91 | A_33_P3641714 | NM_018381 | C19orf66 | -0.036137428 | 0.788582832 | 0.94877096 | 1 | 0.184048186 | 0.149829561 | 0.418314 | 1 |
| 29_10 | A_23_P4922 | NM_199341 | C19orf68 | -0.318183133 | 0.096578755 | 0.500009516 | 1 | 0.404114534 | 0.024315133 | 0.1550296 | 1 |
| 7_88 | A_24_P383581 | NM_205767 | C19orf70 | 0.055571723 | 0.668204765 | 0.916365018 | 1 | -0.020375429 | 0.873314413 | 0.9560217 | 1 |
| 3_129 | A_23_P67992 | NM_006333 | C1D | 0.001764264 | 0.988611499 | 0.99652193 | 1 | -0.060308028 | 0.63925054 | 0.8421438 | 1 |
| 337_155 | A_23_P252145 | NM_020156 | C1GALT1 | 0.1842908 | 0.214280547 | 0.660776824 | 1 | -0.140296369 | 0.240812582 | 0.528472 | 1 |
| 294_151 | A_23_P159839 | NM_152692 | C1GALT1C1 | 0.050664382 | 0.790769371 | 0.94877096 | 1 | 0.425671585 | 0.003871567 | 0.0513485 | 1 |
| 15_148 | A_23_P11862 | NM_018186 | C1orf112 | -0.17752281 | 0.247682085 | 0.693087358 | 1 | 0.049434652 | 0.736474032 | 0.8931957 | 1 |
| 285_124 | A_24_P637982 | NM_198446 | C1orf122 | 0.053943135 | 0.666844451 | 0.915839808 | 1 | 0.123265988 | 0.333777474 | 0.6205551 | 1 |
| 78_126 | A_23_P23017 | NM_017887 | C1orf123 | 0.096042097 | 0.459820476 | 0.834057926 | 1 | 0.035456734 | 0.74174911 | 0.8950651 | 1 |
| 286_81 | A_23_P74668 | NM_152290 | C1orf158 | -0.037035677 | 0.787992067 | 0.94877096 | 1 | 0.175998765 | 0.22692889 | 0.5148774 | 1 |
| 247_11 | A_33_P3416772 | NM_017891 | C1orf159 | -0.189353041 | 0.193400315 | 0.638483101 | 1 | -0.029554558 | 0.81162465 | 0.9264531 | 1 |
| 252_99 | A_24_P298877 | NM_207356 | C1orf174 | 0.120647163 | 0.356348193 | 0.774493075 | 1 | -0.332489317 | 0.021296523 | 0.1426499 | 1 |
| 258_1 | A_33_P3385148 | NM_001007544 | C1orf186 | -0.064965927 | 0.714224253 | 0.932448811 | 1 | -0.059248673 | 0.726270387 | 0.8883483 | 1 |
| 255_99 | A_32_P42574 | NM_032800 | C1orf198 | 0.144958754 | 0.245250061 | 0.691102531 | 1 | -0.000692427 | 0.996219407 | 0.9977042 | 1 |
| 204_65 | A_23_P103511 | NM_001085375 | C1orf226 | -0.194577952 | 0.172962527 | 0.612949273 | 1 | -0.53293495 | 0.001806614 | 0.0317981 | 1 |
| 253_135 | A_32_P148824 | NM_017847 | C1orf27 | -0.01070194 | 0.941281464 | 0.988519322 | 1 | 0.095855098 | 0.40531402 | 0.6828291 | 1 |
| 25_9 | A_33_P3263523 | NM_024319 | C1orf35 | -0.193935964 | 0.265003942 | 0.707490253 | 1 | 0.299886379 | 0.043537424 | 0.2158946 | 1 |
| 112_116 | A_23_P97328 | NM_024319 | C1orf35 | 0.209621235 | 0.108503598 | 0.524936785 | 1 | -0.093882319 | 0.373608543 | 0.6564137 | 1 |
| 6_53 | A_24_P393461 | NM_138740 | C1orf43 | 0.240606114 | 0.068522386 | 0.450747228 | 1 | -0.034695268 | 0.804185283 | 0.9231312 | 1 |
| 62_72 | A_23_P74435 | NM_024097 | C1orf50 | 0.086581756 | 0.491073821 | 0.847345792 | 1 | 0.131014757 | 0.257230792 | 0.5464818 | 1 |
| 329_81 | A_24_P195037 | NM_198077 | C1orf52 | -0.13513447 | 0.296541363 | 0.733049286 | 1 | 0.053992352 | 0.626841975 | 0.8327536 | 1 |
| 335_92 | A_32_P210572 | NM_001024594 | C1orf53 | -0.161965856 | 0.211837791 | 0.658654409 | 1 | 0.315144074 | 0.050958133 | 0.2358544 | 1 |
| 259_6 | A_33_P3381454 | NM_017860 | C1orf56 | -0.33754368 | 0.075452998 | 0.461718925 | 1 | 0.193941374 | 0.251821186 | 0.5396785 | 1 |
| 63_145 | A_24_P234116 | NM_017860 | C1orf56 | -0.123717782 | 0.727127364 | 0.936348816 | 1 | 0.072106253 | 0.775591559 | 0.9104559 | 1 |
| 127_27 | A_23_P370434 | NM_001212 | C1QBP | 0.222052359 | 0.118513782 | 0.540937779 | 1 | -0.093980995 | 0.499538243 | 0.7538316 | 1 |
| 129_139 | A_33_P3393821 | NM_001733 | C1R | -0.420030281 | 0.008352306 | 0.234464792 | 1 | -0.111374247 | 0.56082192 | 0.7948116 | 1 |
| 382_57 | A_33_P3251462 | NM_080739 | C20orf141 | 0.276397509 | 0.054474704 | 0.418919 | 1 | 0.041098114 | 0.850139949 | 0.9450948 | 1 |
| 289_15 | A_23_P102582 | NM_018840 | C20orf24 | -0.004990611 | 0.969483881 | 0.994075478 | 1 | 0.056695919 | 0.614283595 | 0.8244448 | 1 |
| 273_15 | A_23_P102582 | NM_018840 | C20orf24 | 0.003142728 | 0.980364288 | 0.995429381 | 1 | -0.087971691 | 0.425574518 | 0.6996668 | 1 |
| 334_47 | A_23_P102582 | NM_018840 | C20orf24 | 0.060223442 | 0.651703014 | 0.908340249 | 1 | -0.066620009 | 0.62668734 | 0.8327449 | 1 |
| 347_41 | A_23_P102582 | NM_018840 | C20orf24 | 0.072384264 | 0.594819133 | 0.889988626 | 1 | -0.101277619 | 0.45755469 | 0.7209467 | 1 |
| 382_86 | A_23_P102582 | NM_018840 | C20orf24 | 0.1282632 | 0.352883532 | 0.772753403 | 1 | -0.133532052 | 0.244697516 | 0.5321113 | 1 |
| 336_111 | A_23_P102582 | NM_018840 | C20orf24 | 0.176738948 | 0.193548453 | 0.638501051 | 1 | -0.092453427 | 0.438615508 | 0.7095956 | 1 |
| 22_110 | A_23_P102582 | NM_018840 | C20orf24 | 0.186909796 | 0.19667231 | 0.642839304 | 1 | -0.165808385 | 0.18700563 | 0.4671431 | 1 |
| 346_101 | A_23_P102582 | NM_018840 | C20orf24 | 0.187205971 | 0.2072002 | 0.654291494 | 1 | -0.084731006 | 0.45307197 | 0.7200368 | 1 |
| 77_133 | A_23_P102582 | NM_018840 | C20orf24 | 0.187464649 | 0.208390338 | 0.655712085 | 1 | -0.09523956 | 0.384247217 | 0.6656373 | 1 |
| 47_141 | A_23_P102582 | NM_018840 | C20orf24 | 0.251673609 | 0.070435875 | 0.455123502 | 1 | -0.170776107 | 0.193577317 | 0.4756749 | 1 |
| 279_37 | A_33_P3399433 | NM_001258430 | C20orf27 | -0.305791836 | 0.056843656 | 0.423246544 | 1 | 0.112378371 | 0.388984802 | 0.6699575 | 1 |
| 57_1 | A_22_P00002740 | NM_001271441 | C21orf2 | -0.406654007 | 0.014383312 | 0.275053663 | 1 | 0.137005433 | 0.483817505 | 0.7417524 | 1 |
| 326_65 | A_23_P109333 | NM_004649 | C21orf33 | -0.174189787 | 0.29781804 | 0.734102656 | 1 | -0.048918641 | 0.687506593 | 0.8689581 | 1 |
| 73_125 | A_23_P109333 | NM_004649 | C21orf33 | -0.136144144 | 0.366006099 | 0.781778189 | 1 | -0.139345755 | 0.320642086 | 0.6071147 | 1 |
| 382_140 | A_23_P109333 | NM_004649 | C21orf33 | -0.118723356 | 0.379933295 | 0.790179752 | 1 | -0.135296868 | 0.285117777 | 0.5766099 | 1 |
| 318_84 | A_23_P109333 | NM_004649 | C21orf33 | -0.11721968 | 0.440534584 | 0.827546746 | 1 | -0.014230555 | 0.906697401 | 0.9674331 | 1 |
| 100_127 | A_23_P109333 | NM_004649 | C21orf33 | -0.114899063 | 0.421435362 | 0.817774645 | 1 | -0.130838693 | 0.283352035 | 0.5742688 | 1 |
| 113_76 | A_23_P109333 | NM_004649 | C21orf33 | -0.070465054 | 0.60552465 | 0.894904051 | 1 | -0.094901635 | 0.513795132 | 0.761652 | 1 |
| 20_8 | A_23_P109333 | NM_004649 | C21orf33 | -0.018274786 | 0.902385523 | 0.976816041 | 1 | -0.047511431 | 0.683616577 | 0.8680858 | 1 |
| 120_71 | A_23_P109333 | NM_004649 | C21orf33 | -0.015092747 | 0.915524273 | 0.98101793 | 1 | -0.10611874 | 0.42545318 | 0.6995719 | 1 |
| 22_159 | A_23_P109333 | NM_004649 | C21orf33 | 0.004816136 | 0.971201123 | 0.994466847 | 1 | -0.220187293 | 0.099204958 | 0.3427828 | 1 |
| 35_157 | A_23_P109333 | NM_004649 | C21orf33 | 0.017506592 | 0.900234141 | 0.97618995 | 1 | -0.188818933 | 0.161173152 | 0.4350167 | 1 |
| 309_82 | A_23_P91491 | NM_021254 | C21orf59 | -0.088400927 | 0.520322976 | 0.862078486 | 1 | -0.191899583 | 0.153775081 | 0.4224383 | 1 |
| 100_79 | A_33_P3297205 | NM_024627 | C22orf29 | -0.183215794 | 0.21750201 | 0.663744403 | 1 | -0.048543451 | 0.698649881 | 0.874605 | 1 |
| 54_100 | A_23_P353056 | NM_014807 | C2CD2L | 0.001168043 | 0.993810186 | 0.998527818 | 1 | 0.064356384 | 0.654427526 | 0.8511836 | 1 |
| 340_66 | A_23_P150741 | NM_001286577 | C2CD3 | -0.154370692 | 0.256484315 | 0.699039445 | 1 | 0.079697573 | 0.597428643 | 0.816967 | 1 |
| 84_9 | A_33_P3215412 | NM_001136263 | C2CD4C | -0.049743019 | 0.701994996 | 0.926034391 | 1 | 0.13700965 | 0.271472166 | 0.5622923 | 1 |
| 315_147 | A_23_P367628 | NM_014802 | C2CD5 | 0.152552603 | 0.271293997 | 0.714790846 | 1 | -0.421997518 | 0.016856107 | 0.1246421 | 1 |
| 16_129 | A_23_P131526 | NM_024520 | C2orf47 | 0.192331669 | 0.214984301 | 0.661348172 | 1 | 0.384437275 | 0.006420127 | 0.0706839 | 1 |
| 135_28 | A_33_P3386132 | NM_024093 | C2orf49 | -0.019618088 | 0.895078542 | 0.975299356 | 1 | 0.032180087 | 0.780720116 | 0.9131405 | 1 |
| 287_67 | A_23_P165698 | NM_024093 | C2orf49 | -0.011695292 | 0.932219579 | 0.986046619 | 1 | -0.107920151 | 0.406022837 | 0.6834356 | 1 |
| 85_125 | A_33_P3398998 | NM_182500 | C2orf50 | 0.139334759 | 0.45741709 | 0.83358833 | 1 | 0.064839442 | 0.722759659 | 0.8867705 | 1 |
| 180_15 | A_24_P413941 | NM_153689 | C2orf69 | -0.139446847 | 0.304629178 | 0.738652994 | 1 | 0.255075494 | 0.088665752 | 0.3211671 | 1 |
| 263_151 | A_23_P29655 | NM_020685 | C3orf14 | 0.188367328 | 0.197173589 | 0.643786831 | 1 | -0.063835606 | 0.677010862 | 0.8649712 | 1 |
| 276_140 | A_23_P388433 | NM_001170330 | C4orf3 | -0.271721863 | 0.101184831 | 0.509810899 | 1 | 0.005248082 | 0.967386918 | 0.988121 | 1 |
| 26_139 | A_33_P3285824 | NM_001170330 | C4orf3 | 0.313261652 | 0.101430245 | 0.510474111 | 1 | 0.039885245 | 0.768441696 | 0.9077701 | 1 |
| 78_128 | A_33_P3344243 | NM_001008393 | C4orf46 | -0.217039763 | 0.107234886 | 0.52274325 | 1 | 0.108049161 | 0.434969868 | 0.7078419 | 1 |
| 95_81 | A_33_P3377691 | NM_001008393 | C4orf46 | -0.174659539 | 0.167902298 | 0.607928988 | 1 | 0.167751354 | 0.150816554 | 0.4187198 | 1 |
| 106_96 | A_32_P90047 | NM_001141936 | C4orf48 | -0.191244127 | 0.156341425 | 0.595754329 | 1 | 0.10875709 | 0.385212174 | 0.6659209 | 1 |
| 373_71 | A_33_P3868357 | ENST00000507936 | C5orf17 | -0.135765599 | 0.333848542 | 0.761658896 | 1 | 0.043592989 | 0.77143387 | 0.9087179 | 1 |
| 272_14 | A_23_P122007 | NM_033211 | C5orf30 | -0.457232348 | 0.003249791 | 0.171659439 | 1 | 0.237008748 | 0.090436575 | 0.3245605 | 1 |
| 67_5 | A_23_P122007 | NM_033211 | C5orf30 | -0.315319033 | 0.027479189 | 0.332289262 | 1 | 0.183588978 | 0.195894488 | 0.4780268 | 1 |
| 216_33 | A_23_P122007 | NM_033211 | C5orf30 | -0.31284783 | 0.047522052 | 0.399787213 | 1 | 0.227447823 | 0.125331244 | 0.3851159 | 1 |
| 296_105 | A_23_P122007 | NM_033211 | C5orf30 | -0.299572348 | 0.048572649 | 0.402931587 | 1 | 0.300353433 | 0.025645502 | 0.1603307 | 1 |
| 188_136 | A_23_P122007 | NM_033211 | C5orf30 | -0.291296511 | 0.03810831 | 0.371467292 | 1 | 0.228174518 | 0.074639078 | 0.2919881 | 1 |
| 368_156 | A_23_P122007 | NM_033211 | C5orf30 | -0.275382432 | 0.050749328 | 0.406194685 | 1 | 0.240499952 | 0.071333274 | 0.2842704 | 1 |
| 13_82 | A_23_P122007 | NM_033211 | C5orf30 | -0.17998992 | 0.227339025 | 0.672939321 | 1 | 0.220733423 | 0.084191948 | 0.3126844 | 1 |
| 67_86 | A_23_P122007 | NM_033211 | C5orf30 | -0.1732557 | 0.220019374 | 0.666483098 | 1 | 0.228667735 | 0.079504161 | 0.3037182 | 1 |
| 20_82 | A_23_P122007 | NM_033211 | C5orf30 | -0.134764309 | 0.292949675 | 0.730694705 | 1 | 0.136222441 | 0.318958965 | 0.6058658 | 1 |
| 167_70 | A_23_P122007 | NM_033211 | C5orf30 | -0.068185943 | 0.629789823 | 0.90073429 | 1 | 0.27298622 | 0.028351503 | 0.1689372 | 1 |
| 152_146 | A_24_P363087 | NM_016175 | C5orf45 | -0.061817638 | 0.649460955 | 0.907984842 | 1 | 0.105205268 | 0.385640113 | 0.6664558 | 1 |
| 115_162 | A_33_P3296198 | NM_001164479 | C5orf63 | -0.113876117 | 0.349818128 | 0.771176658 | 1 | 0.110686244 | 0.476125445 | 0.7355842 | 1 |
| 223_67 | A_23_P81993 | NM_178508 | C6orf1 | 0.304352881 | 0.037903873 | 0.371467292 | 1 | 0.274905889 | 0.13073562 | 0.3920492 | 1 |
| 12_80 | A_24_P320526 | NM_024294 | C6orf106 | 0.033126415 | 0.852625494 | 0.965079421 | 1 | -0.073192607 | 0.70912118 | 0.8794903 | 1 |
| 253_87 | A_23_P259333 | NM_016487 | C6orf203 | -0.123585417 | 0.377593337 | 0.789750068 | 1 | 0.100376391 | 0.436057426 | 0.7081054 | 1 |
| 286_154 | A_33_P3408898 | NM_001008739 | C6orf226 | -0.206641883 | 0.123478421 | 0.547584317 | 1 | 0.060554908 | 0.6915374 | 0.871307 | 1 |
| 272_47 | A_23_P122531 | NM_001040437 | C6orf48 | 0.073808406 | 0.634563055 | 0.902012501 | 1 | 0.163643008 | 0.184165857 | 0.4636525 | 1 |
| 219_18 | A_23_P122531 | NM_001040437 | C6orf48 | 0.138318449 | 0.351898162 | 0.771839653 | 1 | 0.1563268 | 0.169510576 | 0.4466929 | 1 |
| 335_33 | A_23_P122531 | NM_001040437 | C6orf48 | 0.163962673 | 0.303769916 | 0.738480367 | 1 | 0.195739402 | 0.200421554 | 0.4835138 | 1 |
| 38_27 | A_23_P122531 | NM_001040437 | C6orf48 | 0.167395441 | 0.276081455 | 0.716856302 | 1 | 0.160685409 | 0.151587706 | 0.4201348 | 1 |
| 177_34 | A_23_P122531 | NM_001040437 | C6orf48 | 0.195232497 | 0.175437498 | 0.616022131 | 1 | 0.112333341 | 0.355513258 | 0.640368 | 1 |
| 83_163 | A_23_P122531 | NM_001040437 | C6orf48 | 0.196045404 | 0.20944433 | 0.656068612 | 1 | 0.117031008 | 0.289465424 | 0.5808417 | 1 |
| 275_101 | A_23_P122531 | NM_001040437 | C6orf48 | 0.230593784 | 0.077875835 | 0.46525114 | 1 | 0.122217249 | 0.306711598 | 0.5955776 | 1 |
| 243_97 | A_23_P122531 | NM_001040437 | C6orf48 | 0.245573591 | 0.073506688 | 0.459463791 | 1 | 0.041805784 | 0.80384137 | 0.9231312 | 1 |
| 219_63 | A_23_P122531 | NM_001040437 | C6orf48 | 0.387501395 | 0.012722918 | 0.265846757 | 1 | 0.111412929 | 0.504546055 | 0.7573621 | 1 |
| 67_78 | A_33_P3353051 | NM_001287483 | C6orf48 | 0.456338065 | 0.025031032 | 0.320639364 | 1 | 0.035441964 | 0.819050735 | 0.9306793 | 1 |
| 175_119 | A_23_P122531 | NM_001040437 | C6orf48 | 0.462467461 | 0.00484202 | 0.196694236 | 1 | 0.017055841 | 0.89031925 | 0.961904 | 1 |
| 343_80 | A_23_P42514 | NM_030939 | C6orf62 | -0.148851989 | 0.251928098 | 0.695888548 | 1 | 0.040053972 | 0.779204399 | 0.9126015 | 1 |
| 228_65 | A_33_P3215023 | NM_152734 | C6orf89 | -0.09133997 | 0.513235388 | 0.8585521 | 1 | 0.232611759 | 0.089729895 | 0.3231483 | 1 |
| 255_8 | A_33_P3215028 | NM_152734 | C6orf89 | 0.084127342 | 0.55352914 | 0.875569848 | 1 | 0.194502185 | 0.148660478 | 0.4170577 | 1 |
| 254_113 | A_23_P168541 | NM_024067 | C7orf26 | 0.096715606 | 0.442243784 | 0.828716248 | 1 | -0.316762982 | 0.015045914 | 0.1174333 | 1 |
| 277_101 | A_23_P134477 | NM_032350 | C7orf50 | 0.294881093 | 0.040113968 | 0.377933764 | 1 | -0.195556444 | 0.088336542 | 0.3206432 | 1 |
| 306_80 | A_33_P3422812 | NM_001130929 | C7orf73 | 0.032637592 | 0.802776407 | 0.952164507 | 1 | -0.035030853 | 0.817652585 | 0.9295602 | 1 |
| 167_22 | A_33_P3375613 | NM_001204173 | C8orf44-SGK3 | -0.067701085 | 0.732412882 | 0.937415492 | 1 | 0.041613778 | 0.693778501 | 0.8723227 | 1 |
| 167_27 | A_23_P310483 | NM_001013842 | C8orf58 | -0.120815172 | 0.362987596 | 0.779115119 | 1 | 0.23920625 | 0.103136436 | 0.3487599 | 1 |
| 110_138 | A_33_P3410849 | NM_001013842 | C8orf58 | 0.168980309 | 0.512281699 | 0.85794362 | 1 | 0.07192026 | 0.775410108 | 0.9103379 | 1 |
| 241_20 | A_33_P3422258 | NM_001293320 | C8orf59 | -0.053325204 | 0.69819268 | 0.925106933 | 1 | 0.148128929 | 0.263923381 | 0.5542004 | 1 |
| 271_15 | A_23_P31747 | NM_032847 | C8orf76 | -0.196925057 | 0.128500495 | 0.556728556 | 1 | -0.092984721 | 0.507152007 | 0.7580871 | 1 |
| 229_154 | A_33_P3387050 | NM_001001795 | C8orf82 | -0.303806946 | 0.028156048 | 0.333724089 | 1 | -0.138799582 | 0.430237109 | 0.7033743 | 1 |
| 224_96 | A_23_P422115 | NM_001048265 | C9orf116 | -0.056556765 | 0.695094571 | 0.924516002 | 1 | -0.31900687 | 0.044736152 | 0.2189715 | 1 |
| 193_2 | A_23_P312646 | NM_183241 | C9orf142 | -0.035528172 | 0.791734632 | 0.94877096 | 1 | -0.09341658 | 0.419514212 | 0.6953367 | 1 |
| 8_90 | A_23_P331092 | NM_152571 | C9orf163 | 0.220824281 | 0.207256717 | 0.654291494 | 1 | 0.019254966 | 0.88619764 | 0.9608259 | 1 |
| 133_1 | A_23_P73012 | NM_032823 | C9orf3 | 0.137536783 | 0.432727682 | 0.823111139 | 1 | -0.033062968 | 0.797353251 | 0.9204689 | 1 |
| 252_106 | A_21_P0014236 | ENST00000482056 | C9orf3 | 0.588594091 | 0.02950602 | 0.338940999 | 1 | 0.166402802 | 0.242915648 | 0.5306909 | 1 |
| 139_140 | A_24_P43876 | NM_017998 | C9orf40 | 0.018644562 | 0.902088014 | 0.976669407 | 1 | 0.053428679 | 0.657229503 | 0.8531695 | 1 |
| 278_105 | A_24_P303874 | NM_173520 | C9orf62 | -0.000523232 | 0.997235029 | 0.999485471 | 1 | 0.2831457 | 0.061662493 | 0.2625155 | 1 |
| 80_73 | A_23_P9443 | NM_016520 | C9orf78 | 0.048212665 | 0.715974698 | 0.933034207 | 1 | -0.118405419 | 0.459472471 | 0.722045 | 1 |
| 148_86 | A_19_P00320384 | ENST00000380336 | CA5BP1 | 0.008527869 | 0.949775987 | 0.99027625 | 1 | -0.028438734 | 0.851177982 | 0.9453144 | 1 |
| 1_92 | A_24_P336113 | NM_012295 | CABIN1 | 0.055409568 | 0.660210063 | 0.911868366 | 1 | -0.09885202 | 0.458499801 | 0.7211225 | 1 |
| 341_22 | A_23_P422851 | NM_138375 | CABLES1 | 0.544837733 | 0.063061509 | 0.435697175 | 1 | -0.186891284 | 0.271899651 | 0.5625611 | 1 |
| 214_43 | A_23_P21747 | NM_019855 | CABP5 | 0.060291225 | 0.678501935 | 0.919181603 | 1 | 0.018834756 | 0.919549644 | 0.9713137 | 1 |
| 209_15 | A_33_P3218625 | NM_001242369 | CACFD1 | -0.393602012 | 0.014393239 | 0.275053663 | 1 | -0.364452156 | 0.020588643 | 0.1402547 | 1 |
| 222_84 | A_23_P373031 | NM_000719 | CACNA1C | 0.106210039 | 0.437158276 | 0.826037245 | 1 | 0.06011913 | 0.659673099 | 0.8549601 | 1 |
| 30_75 | A_23_P372144 | NM_021231 | CACTIN | 0.036234055 | 0.778943367 | 0.947866157 | 1 | -0.035566582 | 0.743572087 | 0.896351 | 1 |
| 373_46 | A_24_P45379 | NM_014412 | CACYBP | -0.11024846 | 0.462333044 | 0.835753249 | 1 | -0.029192082 | 0.842037039 | 0.9412708 | 1 |
| 234_66 | A_32_P114574 | NM_014412 | CACYBP | 0.0572684 | 0.679287878 | 0.919351731 | 1 | -0.069443994 | 0.602475439 | 0.8190176 | 1 |
| 243_130 | A_24_P84428 | NM_014412 | CACYBP | 0.159021069 | 0.232885971 | 0.679199641 | 1 | -0.200222662 | 0.119727627 | 0.3754047 | 1 |
| 326_119 | A_24_P921366 | NM_033138 | CALD1 | 0.228032418 | 0.117414134 | 0.53981859 | 1 | 0.07183029 | 0.553685509 | 0.7895652 | 1 |
| 45_23 | A_33_P3212782 | NM_001743 | CALM2 | 0.222816401 | 0.525991414 | 0.864679678 | 1 | 0.371872905 | 0.110911099 | 0.3614654 | 1 |
| 111_106 | A_23_P326170 | NM_001743 | CALM2 | 0.582039905 | 0.086453551 | 0.480683325 | 1 | 0.053488887 | 0.828471798 | 0.9346797 | 1 |
| 85_11 | A_24_P219785 | NM_005184 | CALM3 | -0.065237628 | 0.621636094 | 0.89867047 | 1 | 0.113881369 | 0.378265614 | 0.6602038 | 1 |
| 179_148 | A_23_P4944 | NM_005184 | CALM3 | -0.041581502 | 0.757621621 | 0.942483836 | 1 | -0.043171373 | 0.734752395 | 0.8930898 | 1 |
| 129_18 | A_33_P3292854 | NM_004343 | CALR | -0.197964295 | 0.188393611 | 0.631364777 | 1 | 0.190091868 | 0.181804626 | 0.4605308 | 1 |
| 318_49 | A_33_P3318646 | NM_015722 | CALY | -0.337271675 | 0.026940471 | 0.329165762 | 1 | 0.076513916 | 0.600694149 | 0.8184139 | 1 |
| 118_149 | A_24_P141332 | NM_172171 | CAMK2G | 0.052691966 | 0.680000216 | 0.919668885 | 1 | -0.06461333 | 0.575601379 | 0.8043972 | 1 |
| 45_68 | A_33_P3244283 | NM_033259 | CAMK2N2 | -0.460730989 | 0.004097213 | 0.188782732 | 1 | 0.237976161 | 0.100529821 | 0.3442932 | 1 |
| 366_24 | A_33_P3242099 | NM_001297707 | CAMSAP2 | -0.406319283 | 0.020636655 | 0.306188729 | 1 | -0.031593003 | 0.773802132 | 0.9095369 | 1 |
| 61_150 | A_21_P0000131 | NM_001195563 | CAMTA1 | -0.027706854 | 0.849539823 | 0.965069985 | 1 | 0.274543971 | 0.040279467 | 0.2066848 | 1 |
| 219_125 | A_22_P00019562 | ENST00000545606 | CAND1 | 0.048635677 | 0.743113483 | 0.941128659 | 1 | -0.124681326 | 0.335522951 | 0.6217872 | 1 |
| 149_140 | A_33_P3389634 | NM_001747 | CAPG | -0.3862119 | 0.108767787 | 0.525797295 | 1 | -0.191242924 | 0.108785744 | 0.3577524 | 1 |
| 333_69 | A_23_P341349 | NM_023083 | CAPN10 | -0.214988616 | 0.164097215 | 0.603672445 | 1 | 0.330974696 | 0.069347509 | 0.2802556 | 1 |
| 130_164 | A_24_P282210 | NM_005632 | CAPN15 | 0.052312709 | 0.715884746 | 0.933016795 | 1 | -0.218415873 | 0.084339274 | 0.3127421 | 1 |
| 60_84 | A_23_P23924 | NM_001748 | CAPN2 | 0.212048783 | 0.15150293 | 0.589086719 | 1 | -0.178494697 | 0.12897546 | 0.3900476 | 1 |
| 304_52 | A_23_P67648 | NM_001749 | CAPNS1 | 0.171454553 | 0.282607851 | 0.724220286 | 1 | -0.164335967 | 0.420531965 | 0.6963668 | 1 |
| 109_113 | A_33_P3302115 | NM_005898 | CAPRIN1 | -0.041381027 | 0.791187734 | 0.94877096 | 1 | -0.126425017 | 0.426173017 | 0.7000391 | 1 |
| 325_135 | A_24_P310894 | NM_006135 | CAPZA1 | -0.004710039 | 0.970791926 | 0.994466847 | 1 | -0.007863422 | 0.955155497 | 0.9843677 | 1 |
| 238_92 | A_33_P3235568 | NM_001206541 | CAPZB | 0.14520821 | 0.290347867 | 0.728090176 | 1 | -0.257762637 | 0.0798104 | 0.3040769 | 1 |
| 203_96 | A_23_P434890 | NM_014550 | CARD10 | 0.335252598 | 0.095164438 | 0.498686523 | 1 | -0.618338813 | 0.005008935 | 0.0601875 | 1 |
| 322_83 | A_33_P3228315 | NM_052819 | CARD14 | -0.010326898 | 0.940566186 | 0.988335297 | 1 | -0.022171289 | 0.884703704 | 0.9605428 | 1 |
| 315_137 | A_23_P146367 | NM_032310 | CARD19 | 0.006364937 | 0.965510284 | 0.993734901 | 1 | -0.144720459 | 0.200166556 | 0.4832215 | 1 |
| 352_8 | A_33_P3260307 | NM_001042476 | CARHSP1 | -0.457660858 | 0.00572975 | 0.211438012 | 1 | 0.007516777 | 0.966891794 | 0.988064 | 1 |
| 299_150 | A_33_P3256785 | NM_199141 | CARM1 | 0.101327579 | 0.472402021 | 0.838686606 | 1 | -0.196285505 | 0.174373956 | 0.452983 | 1 |
| 184_78 | A_33_P3236591 | NM_001013838 | CARMIL2 | -0.014464199 | 0.919171081 | 0.981666418 | 1 | 0.196242667 | 0.271208394 | 0.561953 | 1 |
| 322_11 | A_23_P128624 | NM_024537 | CARS2 | -0.310175574 | 0.069392496 | 0.452634056 | 1 | 0.072165585 | 0.59878374 | 0.8174528 | 1 |
| 134_53 | A_19_P00809119 | NR_015410 | CASC15 | -0.031144636 | 0.89670895 | 0.975698398 | 1 | 0.545943932 | 0.003836843 | 0.0513485 | 1 |
| 278_20 | A_23_P374288 | NM_007359 | CASC3 | 0.137594507 | 0.359109224 | 0.776265051 | 1 | -0.184760582 | 0.263716324 | 0.5540851 | 1 |
| 78_1 | A_24_P398940 | NM_138423 | CASC4 | -0.051654236 | 0.682840932 | 0.920314282 | 1 | -0.079278767 | 0.553451946 | 0.7894323 | 1 |
| 202_149 | A_23_P100127 | NM_170589 | CASC5 | -0.292975243 | 0.04299515 | 0.390137659 | 1 | -0.228874594 | 0.097577878 | 0.3397704 | 1 |
| 241_133 | A_32_P168464 | NM_003688 | CASK | -0.136734419 | 0.326322031 | 0.756901818 | 1 | -0.574545433 | 0.000784205 | 0.0193845 | 1 |
| 35_36 | A_33_P3349552 | NM_020764 | CASKIN1 | -0.155050674 | 0.371637159 | 0.78563284 | 1 | 0.315384508 | 0.029723911 | 0.1747542 | 1 |
| 77_163 | A_33_P3383283 | NM_032977 | CASP10 | 0.228544926 | 0.137449956 | 0.569141863 | 1 | 0.117088826 | 0.339984614 | 0.6266474 | 1 |
| 37_19 | A_33_P3308387 | NM_032982 | CASP2 | 0.033029965 | 0.841821114 | 0.96336917 | 1 | 0.025693057 | 0.877865728 | 0.9580202 | 1 |
| 349_141 | A_23_P387943 | NM_032982 | CASP2 | 0.056062878 | 0.690833679 | 0.923085623 | 1 | -0.103032014 | 0.35439008 | 0.6392599 | 1 |
| 258_121 | A_23_P92410 | NM_004346 | CASP3 | 0.229465 | 0.122678072 | 0.546432456 | 1 | -0.123525341 | 0.399493718 | 0.677591 | 1 |
| 248_17 | A_23_P35912 | NM_033306 | CASP4 | -0.488674645 | 0.038937676 | 0.374228433 | 1 | 0.189093773 | 0.185018765 | 0.4644394 | 1 |
| 369_75 | A_23_P47304 | NM_004347 | CASP5 | -0.352613706 | 0.148212887 | 0.585260741 | 1 | -0.027357302 | 0.856263737 | 0.9484339 | 1 |
| 230_36 | A_23_P12572 | NM_033338 | CASP7 | -0.012408125 | 0.924819937 | 0.983686063 | 1 | -0.289686562 | 0.024163888 | 0.1546659 | 1 |
| 366_7 | A_23_P213518 | NM_001042440 | CAST | -0.371681521 | 0.020633605 | 0.306188729 | 1 | -0.369003851 | 0.033824621 | 0.1882363 | 1 |
| 177_55 | A_23_P434352 | NM_001042440 | CAST | 0.182720895 | 0.209979669 | 0.656226868 | 1 | 0.2160142 | 0.107590733 | 0.3560071 | 1 |
| 241_121 | A_33_P8814326 | ENST00000429276 | CATSPER2P1 | 0.022841422 | 0.884804837 | 0.972822545 | 1 | -0.054914489 | 0.627650499 | 0.8333068 | 1 |
| 326_46 | A_33_P3620488 | NM_021185 | CATSPERG | -0.005198222 | 0.973059151 | 0.99478068 | 1 | -0.094548508 | 0.457226503 | 0.7207865 | 1 |
| 73_136 | A_33_P3265956 | JF432662 | CBFA2T2 | -0.139741702 | 0.407686052 | 0.808758102 | 1 | -0.061052966 | 0.709525098 | 0.8795214 | 1 |
| 120_99 | A_23_P113634 | NM_001755 | CBFB | 0.176271496 | 0.208456096 | 0.655712085 | 1 | -0.187265473 | 0.149714646 | 0.4182858 | 1 |
| 316_110 | A_23_P113634 | NM_001755 | CBFB | 0.198429896 | 0.131599366 | 0.561823086 | 1 | -0.151907754 | 0.257631015 | 0.5471068 | 1 |
| 236_88 | A_23_P113634 | NM_001755 | CBFB | 0.199750118 | 0.130970617 | 0.560349642 | 1 | -0.163980578 | 0.344266188 | 0.6308182 | 1 |
| 112_106 | A_23_P113634 | NM_001755 | CBFB | 0.220781628 | 0.097922984 | 0.501898955 | 1 | -0.099818928 | 0.407566277 | 0.6846748 | 1 |
| 208_90 | A_23_P113634 | NM_001755 | CBFB | 0.228196904 | 0.091704671 | 0.49154825 | 1 | -0.158307588 | 0.267479099 | 0.5578226 | 1 |
| 304_83 | A_23_P29046 | NM_001757 | CBR1 | -0.238238623 | 0.109773505 | 0.527106118 | 1 | 0.246216047 | 0.049860849 | 0.2335631 | 1 |
| 62_11 | A_33_P3234804 | NM_032783 | CBR4 | -0.240165429 | 0.076888714 | 0.464296843 | 1 | 0.259091525 | 0.059565896 | 0.2565828 | 1 |
| 76_157 | A_32_P35220 | NM_001024916 | CBWD5 | 0.020951325 | 0.88054556 | 0.972272183 | 1 | -0.380640913 | 0.010518768 | 0.0943418 | 1 |
| 187_69 | A_33_P3333975 | NM_001286835 | CBWD5 | 0.147660368 | 0.356341787 | 0.774493075 | 1 | -0.179067612 | 0.217395204 | 0.5038667 | 1 |
| 167_43 | A_24_P298174 | NM_006807 | CBX1 | -0.512534796 | 0.001746372 | 0.134760981 | 1 | 0.052117908 | 0.721425158 | 0.8860556 | 1 |
| 234_28 | A_33_P3332006 | NM_006807 | CBX1 | 0.214883935 | 0.338626997 | 0.764199026 | 1 | -0.00094701 | 0.996037117 | 0.9977042 | 1 |
| 211_95 | A_24_P296568 | NM_006807 | CBX1 | 0.355024631 | 0.118401089 | 0.540937779 | 1 | -0.038036842 | 0.839568169 | 0.9395448 | 1 |
| 47_40 | A_33_P3423949 | NM_005189 | CBX2 | 0.022744133 | 0.859822829 | 0.966684616 | 1 | -0.450230776 | 0.004331407 | 0.0551282 | 1 |
| 111_56 | A_23_P31315 | NM_016587 | CBX3 | 0.30013787 | 0.030307119 | 0.340591633 | 1 | -0.516504794 | 0.001444757 | 0.0274077 | 1 |
| 189_40 | A_23_P2355 | NM_012117 | CBX5 | -0.100253864 | 0.435228945 | 0.825069738 | 1 | -0.101265042 | 0.372193645 | 0.6550958 | 1 |
| 228_99 | A_24_P664995 | NM_001127322 | CBX5 | -0.087932398 | 0.489601359 | 0.846353913 | 1 | 0.326966746 | 0.043926793 | 0.2166862 | 1 |
| 213_30 | A_33_P3270485 | ENST00000407418 | CBX6 | -0.193739033 | 0.158350192 | 0.597283004 | 1 | 0.090931871 | 0.47549547 | 0.7354095 | 1 |
| 364_98 | A_33_P3343845 | NM_175709 | CBX7 | -0.025355491 | 0.860853151 | 0.967090315 | 1 | 0.054759645 | 0.68445307 | 0.8683976 | 1 |
| 381_51 | A_23_P55873 | NM_017721 | CC2D1A | -0.08750024 | 0.574181319 | 0.882765809 | 1 | -0.205424261 | 0.111362919 | 0.3620985 | 1 |
| 5_129 | A_23_P135690 | ENST00000589138 | CC2D1A | 0.175864349 | 0.230161742 | 0.676515722 | 1 | 0.047285233 | 0.702246781 | 0.8760444 | 1 |
| 357_144 | A_23_P115842 | NM_018237 | CCAR1 | -0.082450574 | 0.564213033 | 0.880056743 | 1 | -0.069115698 | 0.59330912 | 0.8148672 | 1 |
| 373_137 | A_23_P115842 | NM_018237 | CCAR1 | -0.022684135 | 0.87045824 | 0.969701843 | 1 | -0.105848668 | 0.453714618 | 0.720549 | 1 |
| 367_120 | A_23_P115842 | NM_018237 | CCAR1 | -0.012048479 | 0.932076808 | 0.986009751 | 1 | -0.021039268 | 0.882833201 | 0.9596236 | 1 |
| 65_11 | A_23_P115842 | NM_018237 | CCAR1 | 0.014985445 | 0.917073501 | 0.981100324 | 1 | -0.113353513 | 0.424639882 | 0.6991546 | 1 |
| 351_90 | A_23_P115842 | NM_018237 | CCAR1 | 0.032356327 | 0.812879989 | 0.955007122 | 1 | -0.030393422 | 0.830014159 | 0.9353877 | 1 |
| 30_101 | A_23_P115842 | NM_018237 | CCAR1 | 0.043336987 | 0.744630046 | 0.941128659 | 1 | -0.047352433 | 0.757734929 | 0.9028348 | 1 |
| 167_48 | A_23_P115842 | NM_018237 | CCAR1 | 0.04784248 | 0.727463536 | 0.936348816 | 1 | 0.029080245 | 0.81888734 | 0.930661 | 1 |
| 184_164 | A_23_P115842 | NM_018237 | CCAR1 | 0.09079527 | 0.477842083 | 0.840908892 | 1 | -0.076440515 | 0.583592729 | 0.8092304 | 1 |
| 2_80 | A_23_P115842 | NM_018237 | CCAR1 | 0.161648947 | 0.254929311 | 0.698346946 | 1 | -0.050686863 | 0.724855266 | 0.8878061 | 1 |
| 206_66 | A_23_P115842 | NM_018237 | CCAR1 | 0.244344009 | 0.073782049 | 0.459750854 | 1 | 0.007068928 | 0.966358681 | 0.9878782 | 1 |
| 121_118 | A_33_P3379726 | NM_013301 | CCDC106 | -0.20687683 | 0.137216942 | 0.568908642 | 1 | 0.09332871 | 0.508086769 | 0.7585891 | 1 |
| 139_17 | A_33_P3312877 | NM_174923 | CCDC107 | -0.530767279 | 0.002158495 | 0.145205992 | 1 | 0.719288766 | 0.002345805 | 0.0372119 | 1 |
| 135_160 | A_23_P387045 | NM_174923 | CCDC107 | -0.098060898 | 0.605775431 | 0.894904051 | 1 | 0.068264915 | 0.597452978 | 0.816967 | 1 |
| 300_122 | A_23_P255376 | NM_017918 | CCDC109B | 0.217835648 | 0.157706126 | 0.595969916 | 1 | -0.127366264 | 0.293490465 | 0.5853656 | 1 |
| 40_46 | A_23_P372334 | NM_144716 | CCDC12 | -0.03225947 | 0.794970742 | 0.949723799 | 1 | -0.110712377 | 0.369660782 | 0.6535012 | 1 |
| 175_93 | A_33_P3417141 | NM_138442 | CCDC124 | 0.325765194 | 0.112462863 | 0.531428468 | 1 | -0.0969462 | 0.489545453 | 0.7463719 | 1 |
| 111_83 | A_23_P432591 | NM_176816 | CCDC125 | -0.107881661 | 0.405587783 | 0.80733176 | 1 | 0.178470299 | 0.130159564 | 0.3913137 | 1 |
| 88_118 | A_33_P3271599 | NM_176816 | CCDC125 | 0.04470878 | 0.721958779 | 0.935627856 | 1 | -0.01330399 | 0.930838596 | 0.9749085 | 1 |
| 72_80 | A_23_P41976 | NM_145265 | CCDC127 | 0.089064428 | 0.478839292 | 0.84100638 | 1 | 0.391063637 | 0.006837392 | 0.0736591 | 1 |
| 272_55 | A_23_P101461 | NM_030818 | CCDC130 | -0.179961144 | 0.21223528 | 0.659049242 | 1 | 0.031371191 | 0.808885449 | 0.9253621 | 1 |
| 256_66 | A_23_P101461 | NM_030818 | CCDC130 | -0.169159151 | 0.215511123 | 0.662132787 | 1 | 0.018790851 | 0.890762438 | 0.9619458 | 1 |
| 19_15 | A_23_P101461 | NM_030818 | CCDC130 | -0.158418116 | 0.359273724 | 0.776265051 | 1 | 0.169469964 | 0.174305059 | 0.452983 | 1 |
| 165_153 | A_23_P101461 | NM_030818 | CCDC130 | -0.157275707 | 0.26415459 | 0.706771627 | 1 | 0.016849342 | 0.904513127 | 0.966562 | 1 |
| 171_40 | A_23_P101461 | NM_030818 | CCDC130 | -0.152799533 | 0.31351839 | 0.74498319 | 1 | 0.138517228 | 0.320791557 | 0.6072955 | 1 |
| 35_82 | A_23_P101461 | NM_030818 | CCDC130 | -0.152154053 | 0.317239157 | 0.748229895 | 1 | -0.002131038 | 0.989219563 | 0.9967442 | 1 |
| 54_67 | A_23_P101461 | NM_030818 | CCDC130 | -0.144357155 | 0.323581599 | 0.753815848 | 1 | 0.090476919 | 0.480632062 | 0.7388831 | 1 |
| 144_124 | A_23_P101461 | NM_030818 | CCDC130 | -0.081510126 | 0.565948732 | 0.880639801 | 1 | -0.013927967 | 0.919409326 | 0.9713137 | 1 |
| 134_128 | A_23_P101461 | NM_030818 | CCDC130 | -0.078969871 | 0.524411578 | 0.864032081 | 1 | 0.064035391 | 0.63913954 | 0.8421277 | 1 |
| 2_98 | A_23_P101461 | NM_030818 | CCDC130 | -0.039036427 | 0.807897558 | 0.953806288 | 1 | 0.045178495 | 0.74457665 | 0.8968891 | 1 |
| 178_76 | A_33_P3287825 | NM_022742 | CCDC136 | -0.584062864 | 0.002676635 | 0.154236866 | 1 | 0.217723715 | 0.139811617 | 0.4055147 | 1 |
| 351_79 | A_24_P230176 | NM_199287 | CCDC137 | -0.131512722 | 0.294232603 | 0.732395091 | 1 | -0.192109028 | 0.299200108 | 0.5896609 | 1 |
| 324_61 | A_23_P384056 | NM_022757 | CCDC14 | -0.1218547 | 0.404111809 | 0.806502302 | 1 | 0.136830467 | 0.361893436 | 0.6467032 | 1 |
| 292_20 | A_33_P3260100 | NM_138493 | CCDC167 | -0.229397628 | 0.078342982 | 0.466003029 | 1 | 0.085297147 | 0.549773199 | 0.787379 | 1 |
| 207_111 | A_23_P133770 | NM_138493 | CCDC167 | 0.264533953 | 0.12472422 | 0.550105158 | 1 | -0.147741863 | 0.34800044 | 0.6336908 | 1 |
| 259_68 | A_33_P3291454 | NM_198515 | CCDC172 | 0.094621382 | 0.661888919 | 0.912956463 | 1 | 0.008602254 | 0.965095041 | 0.9873043 | 1 |
| 243_1 | A_33_P3294881 | NM_016474 | CCDC174 | -0.096869618 | 0.463960931 | 0.836223448 | 1 | 0.016376747 | 0.898775698 | 0.9645009 | 1 |
| 67_130 | A_33_P3362521 | NM_016474 | CCDC174 | -0.07445028 | 0.593280804 | 0.889095491 | 1 | 0.062339918 | 0.651519617 | 0.8490894 | 1 |
| 375_30 | A_33_P3327165 | NM_206886 | CCDC18 | -0.27036799 | 0.053193026 | 0.41409558 | 1 | 0.144307037 | 0.379209357 | 0.6613578 | 1 |
| 122_102 | A_23_P31085 | NM_015439 | CCDC28A | 0.120536517 | 0.336925599 | 0.763046598 | 1 | 0.007272987 | 0.953746469 | 0.9840186 | 1 |
| 353_97 | A_23_P62764 | NM_024296 | CCDC28B | -0.386865281 | 0.029299189 | 0.338472773 | 1 | -0.171997515 | 0.15047701 | 0.4187073 | 1 |
| 342_159 | A_33_P3313796 | NM_030771 | CCDC34 | 0.156083426 | 0.234130451 | 0.680457734 | 1 | -0.155427185 | 0.200939655 | 0.4835834 | 1 |
| 350_162 | A_24_P910733 | NM_178335 | CCDC50 | 0.19798605 | 0.209797452 | 0.656194814 | 1 | 0.059401924 | 0.617574281 | 0.8265926 | 1 |
| 156_155 | A_23_P69362 | NM_024661 | CCDC51 | 0.240381575 | 0.178930642 | 0.618890709 | 1 | 0.007798185 | 0.959397673 | 0.9858263 | 1 |
| 232_129 | A_23_P53567 | NM_016053 | CCDC53 | -0.097883357 | 0.468489731 | 0.836476951 | 1 | -0.245471309 | 0.138218624 | 0.4035245 | 1 |
| 26_133 | A_33_P3351664 | NM_198082 | CCDC57 | -0.076855709 | 0.598590272 | 0.89144727 | 1 | -0.027568163 | 0.805088128 | 0.9235849 | 1 |
| 222_133 | A_33_P3402570 | NM_014167 | CCDC59 | 0.181525486 | 0.187426328 | 0.630397286 | 1 | -0.089852857 | 0.498682741 | 0.7530458 | 1 |
| 248_133 | A_23_P105664 | NM_014167 | CCDC59 | 0.263189391 | 0.098529198 | 0.502841512 | 1 | -0.102023152 | 0.464153213 | 0.7265597 | 1 |
| 268_73 | A_32_P184279 | NM_005436 | CCDC6 | -0.053273329 | 0.669534682 | 0.916686207 | 1 | 0.126741462 | 0.289730525 | 0.5811663 | 1 |
| 21_35 | A_33_P3355247 | NM_015621 | CCDC69 | 0.031585404 | 0.811390467 | 0.954780472 | 1 | 0.207798354 | 0.238493438 | 0.526393 | 1 |
| 345_2 | A_23_P99172 | NM_032358 | CCDC77 | -0.181617199 | 0.1654317 | 0.604465796 | 1 | -0.09201256 | 0.494693875 | 0.7507029 | 1 |
| 26_63 | A_33_P3246613 | NM_001031737 | CCDC78 | -0.263184664 | 0.116714577 | 0.539588985 | 1 | 0.204965681 | 0.172362578 | 0.4501833 | 1 |
| 43_156 | A_24_P636332 | NM_198489 | CCDC84 | -0.235601552 | 0.113511346 | 0.533531628 | 1 | -0.016499456 | 0.88722727 | 0.9609879 | 1 |
| 49_61 | A_23_P150249 | NM_006848 | CCDC85B | 0.287488583 | 0.138918493 | 0.571531604 | 1 | -0.109047815 | 0.453908418 | 0.7206533 | 1 |
| 230_146 | A_23_P37391 | NM_001144995 | CCDC85C | -0.192236572 | 0.180755851 | 0.621649775 | 1 | -0.171455731 | 0.188226552 | 0.4689818 | 1 |
| 201_50 | A_23_P161918 | NM_024098 | CCDC86 | 0.320795606 | 0.115804905 | 0.537023243 | 1 | 0.009613229 | 0.948605566 | 0.9819574 | 1 |
| 76_41 | A_23_P24384 | NM_032251 | CCDC88B | -0.625988119 | 0.000866153 | 0.101456497 | 1 | 0.005519545 | 0.96868751 | 0.9888209 | 1 |
| 331_11 | A_23_P379945 | NM_001080414 | CCDC88C | -0.283508137 | 0.064008631 | 0.437947257 | 1 | -0.073320541 | 0.598709967 | 0.8174528 | 1 |
| 156_101 | A_33_P3345936 | NM_015603 | CCDC9 | 0.141807585 | 0.560722516 | 0.877949216 | 1 | 0.038674956 | 0.842692177 | 0.9413745 | 1 |
| 305_129 | A_23_P162127 | NM_021825 | CCDC90B | -0.191215888 | 0.149917902 | 0.586681674 | 1 | 0.158614416 | 0.195038221 | 0.4771797 | 1 |
| 325_106 | A_23_P162279 | NM_018318 | CCDC91 | 0.020303517 | 0.871455604 | 0.969992795 | 1 | 0.138024369 | 0.223381819 | 0.5117556 | 1 |
| 382_48 | A_23_P79661 | NM_019044 | CCDC93 | 0.067627201 | 0.620265552 | 0.89867047 | 1 | -0.05097693 | 0.644855597 | 0.8457094 | 1 |
| 131_92 | A_33_P3369696 | NM_018074 | CCDC94 | 0.091683922 | 0.556296162 | 0.876234787 | 1 | -0.214606051 | 0.195559328 | 0.4777271 | 1 |
| 117_74 | A_23_P398044 | NM_052848 | CCDC97 | 0.027473529 | 0.826690426 | 0.959621444 | 1 | 0.04655918 | 0.666037861 | 0.8579691 | 1 |
| 284_2 | A_23_P145330 | NM_019052 | CCHCR1 | -0.179218264 | 0.268546531 | 0.711309285 | 1 | 0.047305899 | 0.684781453 | 0.8683976 | 1 |
| 184_21 | A_33_P3292478 | ENST00000621559 | CCL16 | -0.06246536 | 0.7037709 | 0.926847254 | 1 | -0.144284069 | 0.400067087 | 0.6780522 | 1 |
| 226_20 | A_23_P215491 | NM_002991 | CCL24 | 0.033114108 | 0.857770789 | 0.965745263 | 1 | 0.406164369 | 0.009554689 | 0.0892538 | 1 |
| 152_93 | A_33_P3209476 | NM_031443 | CCM2 | -0.20755725 | 0.159429765 | 0.597775747 | 1 | -0.006527491 | 0.959687984 | 0.9858263 | 1 |
| 127_57 | A_33_P3380772 | NM_080625 | CCM2L | -0.265143324 | 0.159840034 | 0.598752215 | 1 | 0.207109783 | 0.434674353 | 0.7075962 | 1 |
| 35_66 | A_33_P3401621 | NM_031966 | CCNB1 | -0.379716833 | 0.030477278 | 0.34142035 | 1 | -0.034479991 | 0.786198846 | 0.9154381 | 1 |
| 253_59 | A_23_P122197 | NM_031966 | CCNB1 | -0.157055305 | 0.267825043 | 0.710951213 | 1 | -0.427340474 | 0.007226286 | 0.0764572 | 1 |
| 353_130 | A_23_P76882 | NM_182852 | CCNB1IP1 | 0.45787232 | 0.009435685 | 0.241777062 | 1 | -0.461591018 | 0.017409196 | 0.1275578 | 1 |
| 183_83 | A_23_P65757 | NM_004701 | CCNB2 | -0.087877162 | 0.610435498 | 0.897313273 | 1 | -0.195066313 | 0.194484442 | 0.4765504 | 1 |
| 208_75 | A_33_P3392977 | NM_012142 | CCNDBP1 | 0.097095329 | 0.476211445 | 0.840419295 | 1 | 0.078282194 | 0.603486891 | 0.8191729 | 1 |
| 317_76 | A_23_P209200 | NM_001238 | CCNE1 | -0.106310762 | 0.499124248 | 0.851671485 | 1 | 0.847721591 | 0.000208678 | 0.0080931 | 1 |
| 266_18 | A_33_P3247022 | NM_057749 | CCNE2 | -0.099951037 | 0.453821473 | 0.832937561 | 1 | -0.186256673 | 0.118784372 | 0.3739065 | 1 |
| 372_88 | A_23_P30338 | NM_001239 | CCNH | 0.000743554 | 0.995404141 | 0.998909536 | 1 | -0.012574983 | 0.918984743 | 0.9713137 | 1 |
| 193_89 | A_23_P69521 | NM_006835 | CCNI | 0.018333255 | 0.938192724 | 0.987648096 | 1 | 0.10197348 | 0.606895736 | 0.8206139 | 1 |
| 260_140 | A_23_P24176 | NM_019084 | CCNJ | -0.24034573 | 0.211237866 | 0.657798949 | 1 | -0.039040006 | 0.77879654 | 0.9123138 | 1 |
| 185_77 | A_24_P348925 | NM_001099402 | CCNK | -0.041008334 | 0.77725412 | 0.946928757 | 1 | -0.06214455 | 0.596991747 | 0.8167335 | 1 |
| 361_53 | A_24_P376339 | NM_001039577 | CCNL2 | -0.041095124 | 0.802718927 | 0.952164507 | 1 | -0.355757184 | 0.059341448 | 0.2562669 | 1 |
| 276_63 | A_23_P92860 | NM_021147 | CCNO | -0.258847527 | 0.0655283 | 0.443209074 | 1 | 0.599415575 | 0.00089051 | 0.0210336 | 1 |
| 326_96 | A_22_P00017762 | NR_036549 | CCNT2-AS1 | -0.1945056 | 0.498762037 | 0.8515654 | 1 | 0.45168889 | 0.003195806 | 0.0458169 | 1 |
| 379_110 | A_23_P127475 | NM_005125 | CCS | 0.033224667 | 0.785796584 | 0.94877096 | 1 | -0.126949122 | 0.239538312 | 0.5271155 | 1 |
| 131_58 | A_33_P3359753 | NM_145257 | CCSAP | -0.246132246 | 0.10001521 | 0.507331785 | 1 | 0.006718742 | 0.954772167 | 0.9842642 | 1 |
| 339_42 | A_23_P105392 | NM_006431 | CCT2 | 0.099642828 | 0.474830995 | 0.839626091 | 1 | -0.204197517 | 0.204234641 | 0.4868646 | 1 |
| 325_47 | A_23_P105392 | NM_006431 | CCT2 | 0.108508416 | 0.465846409 | 0.836223448 | 1 | -0.051188464 | 0.722238018 | 0.8864082 | 1 |
| 72_117 | A_23_P105392 | NM_006431 | CCT2 | 0.16851616 | 0.233611375 | 0.679769612 | 1 | -0.167134074 | 0.194202586 | 0.4763073 | 1 |
| 98_133 | A_23_P105392 | NM_006431 | CCT2 | 0.177403853 | 0.228909756 | 0.675109425 | 1 | -0.238143676 | 0.125380172 | 0.3851159 | 1 |
| 116_59 | A_23_P105392 | NM_006431 | CCT2 | 0.206594151 | 0.120199513 | 0.542925647 | 1 | -0.172621369 | 0.33657039 | 0.6228047 | 1 |
| 79_91 | A_23_P105392 | NM_006431 | CCT2 | 0.218375395 | 0.094291066 | 0.498286526 | 1 | -0.154373288 | 0.238209212 | 0.5262263 | 1 |
| 161_131 | A_23_P105392 | NM_006431 | CCT2 | 0.223330047 | 0.103483749 | 0.515809751 | 1 | -0.187341887 | 0.20239261 | 0.4851248 | 1 |
| 318_79 | A_23_P105392 | NM_006431 | CCT2 | 0.293128372 | 0.039025735 | 0.374228433 | 1 | -0.084078275 | 0.539690566 | 0.7802931 | 1 |
| 68_99 | A_23_P105392 | NM_006431 | CCT2 | 0.322757047 | 0.024852191 | 0.319019375 | 1 | -0.168361437 | 0.162390165 | 0.4369185 | 1 |
| 213_104 | A_23_P105392 | NM_006431 | CCT2 | 0.400195124 | 0.016037346 | 0.286184492 | 1 | -0.223386317 | 0.204781207 | 0.4876507 | 1 |
| 59_40 | A_23_P160631 | NM_005998 | CCT3 | 0.173658627 | 0.248363991 | 0.693938104 | 1 | -0.510370469 | 0.039936569 | 0.2060703 | 1 |
| 151_8 | A_23_P102420 | NM_006430 | CCT4 | -0.289684256 | 0.077404897 | 0.464963734 | 1 | 0.734373054 | 0.152551824 | 0.4209397 | 1 |
| 353_42 | A_33_P3229397 | NM_006430 | CCT4 | -0.157208994 | 0.223525138 | 0.668162676 | 1 | -0.211898669 | 0.22805986 | 0.5159722 | 1 |
| 120_23 | A_32_P54544 | NM_001762 | CCT6A | 0.41564504 | 0.023736231 | 0.316876636 | 1 | -0.52694974 | 0.0660572 | 0.272802 | 1 |
| 338_43 | A_23_P102404 | NM_006429 | CCT7 | -0.139044063 | 0.457160199 | 0.83358833 | 1 | -0.155355309 | 0.449348734 | 0.7172616 | 1 |
| 108_38 | A_23_P102404 | NM_006429 | CCT7 | -0.138001121 | 0.398953315 | 0.802385551 | 1 | -0.214439984 | 0.365511676 | 0.6497408 | 1 |
| 31_74 | A_23_P102404 | NM_006429 | CCT7 | -0.070473135 | 0.725244429 | 0.936348816 | 1 | -0.233475114 | 0.294323425 | 0.5853942 | 1 |
| 299_52 | A_23_P102404 | NM_006429 | CCT7 | -0.069665343 | 0.682775318 | 0.920314282 | 1 | -0.100566709 | 0.600642537 | 0.8184139 | 1 |
| 11_83 | A_23_P102404 | NM_006429 | CCT7 | -0.069538683 | 0.711361828 | 0.930633257 | 1 | -0.156727911 | 0.479155621 | 0.7375206 | 1 |
| 139_40 | A_23_P102404 | NM_006429 | CCT7 | -0.036601761 | 0.829320698 | 0.959690637 | 1 | -0.156704235 | 0.485188383 | 0.742768 | 1 |
| 169_160 | A_23_P102404 | NM_006429 | CCT7 | -0.005086922 | 0.980607725 | 0.995429381 | 1 | -0.333140668 | 0.175585641 | 0.4542244 | 1 |
| 71_117 | A_23_P102404 | NM_006429 | CCT7 | 0.004696618 | 0.981461035 | 0.995429381 | 1 | -0.218655848 | 0.271833295 | 0.5625611 | 1 |
| 227_58 | A_23_P102404 | NM_006429 | CCT7 | 0.105833551 | 0.584129743 | 0.884306572 | 1 | -0.278799093 | 0.287976927 | 0.5792383 | 1 |
| 132_89 | A_23_P102404 | NM_006429 | CCT7 | 0.21216875 | 0.295058265 | 0.732603053 | 1 | -0.281873602 | 0.276324926 | 0.567847 | 1 |
| 282_54 | A_23_P102876 | NM_006585 | CCT8 | 0.38580986 | 0.023762469 | 0.316876636 | 1 | -0.288637837 | 0.186106155 | 0.4660735 | 1 |
| 212_22 | A_21_P0013340 | ENST00000478672 | CCZ1 | -0.114505567 | 0.444126313 | 0.829470233 | 1 | 0.133718047 | 0.281066508 | 0.5719477 | 1 |
| 207_154 | A_24_P377489 | NM_015622 | CCZ1 | 0.173088416 | 0.294923483 | 0.732603053 | 1 | -0.528617541 | 0.001437322 | 0.0274077 | 1 |
| 67_26 | A_33_P3229196 | NM_004357 | CD151 | -0.16605103 | 0.311633778 | 0.743773338 | 1 | -0.061507075 | 0.606908658 | 0.8206139 | 1 |
| 192_4 | A_33_P3222917 | NM_001024736 | CD276 | -0.210989949 | 0.218607224 | 0.665527575 | 1 | -0.059920051 | 0.670389136 | 0.8610213 | 1 |
| 87_160 | A_33_P3358099 | NM_181449 | CD300E | 0.079367058 | 0.567622309 | 0.881090627 | 1 | 0.008713924 | 0.934073462 | 0.9765392 | 1 |
| 56_112 | A_23_P119698 | NM_016579 | CD320 | 0.137515182 | 0.301906131 | 0.736931323 | 1 | -0.38631817 | 0.021399532 | 0.1429219 | 1 |
| 11_157 | A_23_P208310 | NM_012099 | CD3EAP | 0.089930448 | 0.558995718 | 0.877439019 | 1 | 0.050572421 | 0.722772779 | 0.8867705 | 1 |
| 163_91 | A_24_P295999 | NM_000616 | CD4 | 0.185057435 | 0.346286158 | 0.768529418 | 1 | 0.028261639 | 0.876299845 | 0.9572584 | 1 |
| 75_96 | A_33_P3294509 | NM_000610 | CD44 | 0.058616578 | 0.691367035 | 0.923085623 | 1 | -0.351843553 | 0.006935298 | 0.0740753 | 1 |
| 84_103 | A_23_P6935 | NM_198793 | CD47 | 0.105936182 | 0.618865665 | 0.89867047 | 1 | 0.18946463 | 0.171127064 | 0.4488494 | 1 |
| 344_27 | A_33_P3289356 | NM_001779 | CD58 | 0.077456506 | 0.572944222 | 0.882600723 | 1 | 0.093304095 | 0.582732954 | 0.8088721 | 1 |
| 381_62 | A_33_P3209096 | NM_001779 | CD58 | 0.079105285 | 0.603122924 | 0.893532545 | 1 | 0.09306911 | 0.503766558 | 0.7571515 | 1 |
| 215_4 | A_24_P270144 | NM_001257389 | CD63 | -0.109745585 | 0.566450222 | 0.880639801 | 1 | -0.450958894 | 0.042098662 | 0.2126054 | 1 |
| 72_135 | A_33_P3417222 | ENST00000378430 | CD72 | 0.058795561 | 0.735482841 | 0.938795048 | 1 | 0.24562169 | 0.05228705 | 0.2395807 | 1 |
| 277_30 | A_23_P107735 | NM_001783 | CD79A | -0.140510679 | 0.313610072 | 0.74498319 | 1 | 0.098698375 | 0.502136667 | 0.7563734 | 1 |
| 204_45 | A_23_P13425 | NM_004356 | CD81 | 0.148976349 | 0.250259889 | 0.694615167 | 1 | -0.368376769 | 0.059663729 | 0.2568075 | 1 |
| 52_111 | A_33_P3373375 | NM_004356 | CD81 | 0.249471148 | 0.096554177 | 0.500009516 | 1 | -0.311686787 | 0.087814979 | 0.3196295 | 1 |
| 293_65 | A_23_P70670 | NM_004233 | CD83 | 0.328576071 | 0.040113255 | 0.377933764 | 1 | 0.404774602 | 0.00596904 | 0.0677435 | 1 |
| 171_161 | A_24_P131589 | NM_006889 | CD86 | -0.079600151 | 0.75611184 | 0.942463951 | 1 | 0.386101952 | 0.013799813 | 0.111703 | 1 |
| 95_83 | A_23_P253052 | NM_031462 | CD99L2 | -0.290069369 | 0.051656613 | 0.409955354 | 1 | 1.157899595 | 0.000141237 | 0.0065696 | 1 |
| 80_93 | A_33_P3419545 | NM_001193478 | CDADC1 | 0.105668917 | 0.388077625 | 0.795892662 | 1 | -0.259524954 | 0.034522635 | 0.1907991 | 1 |
| 214_11 | A_24_P303974 | NM_006023 | CDC123 | -0.244273397 | 0.19304218 | 0.637819322 | 1 | -0.256664682 | 0.085607867 | 0.314529 | 1 |
| 234_133 | A_33_P3296067 | NR_003595 | CDC14C | 0.163491336 | 0.33732549 | 0.763334677 | 1 | 0.069727995 | 0.65618733 | 0.8523521 | 1 |
| 165_24 | A_33_P3392325 | NM_001078645 | CDC16 | -0.312440971 | 0.042499971 | 0.388398087 | 1 | -0.3069323 | 0.05721852 | 0.25244 | 1 |
| 3_57 | A_23_P149200 | NM_001255 | CDC20 | 0.377820142 | 0.04064549 | 0.380963513 | 1 | -0.413284824 | 0.118274039 | 0.3734686 | 1 |
| 362_69 | A_23_P133629 | NM_004661 | CDC23 | 0.043493623 | 0.727893634 | 0.936348816 | 1 | -0.149832725 | 0.325420078 | 0.6114301 | 1 |
| 281_37 | A_32_P228501 | NM_139286 | CDC26 | -0.060914969 | 0.617096474 | 0.89867047 | 1 | -0.020295076 | 0.864867037 | 0.9520563 | 1 |
| 321_149 | A_23_P66777 | NM_001256 | CDC27 | -0.036082205 | 0.818437677 | 0.956466595 | 1 | 0.041424493 | 0.725999547 | 0.8882103 | 1 |
| 271_105 | A_23_P164999 | NM_004359 | CDC34 | 0.006686784 | 0.967830596 | 0.994075478 | 1 | -0.04854274 | 0.729587987 | 0.8900831 | 1 |
| 349_44 | A_24_P414786 | NM_015891 | CDC40 | -0.299989947 | 0.061534665 | 0.431984295 | 1 | 0.158948682 | 0.312355278 | 0.6008481 | 1 |
| 365_68 | A_23_P200560 | NM_001039802 | CDC42 | -0.048444417 | 0.698564751 | 0.925106933 | 1 | -0.198135747 | 0.17407638 | 0.4528885 | 1 |
| 244_57 | A_33_P3257150 | NM_001039802 | CDC42 | 0.127407506 | 0.324706339 | 0.755456957 | 1 | 0.076580013 | 0.623307183 | 0.8304147 | 1 |
| 62_78 | A_23_P380766 | NM_006035 | CDC42BPB | -0.05616637 | 0.660072474 | 0.911868366 | 1 | 0.06177776 | 0.622946651 | 0.830297 | 1 |
| 51_4 | A_33_P3407424 | NM_152243 | CDC42EP1 | -0.260847242 | 0.207480549 | 0.654291494 | 1 | -0.368059931 | 0.019355585 | 0.1359727 | 1 |
| 238_71 | A_23_P1602 | NM_006779 | CDC42EP2 | 0.156666908 | 0.251330874 | 0.695211787 | 1 | -0.04290079 | 0.72695256 | 0.888579 | 1 |
| 217_97 | A_33_P3342235 | NM_145057 | CDC42EP5 | 0.02621331 | 0.845472086 | 0.964376494 | 1 | -0.106000859 | 0.482262382 | 0.7406807 | 1 |
| 77_125 | A_22_P00003672 | ENST00000491825 | CDC42SE1 | -0.036764593 | 0.789086557 | 0.94877096 | 1 | 0.263963228 | 0.177223524 | 0.4555961 | 1 |
| 108_132 | A_24_P409042 | NM_020240 | CDC42SE2 | 0.160812342 | 0.275827682 | 0.716856302 | 1 | -0.159327229 | 0.249615924 | 0.5368329 | 1 |
| 361_2 | A_23_P57379 | NM_003504 | CDC45 | -0.16611044 | 0.269055512 | 0.711601009 | 1 | -0.030560802 | 0.799481572 | 0.9212674 | 1 |
| 130_118 | A_23_P156471 | NM_001253 | CDC5L | 0.008499113 | 0.948103956 | 0.990231079 | 1 | 0.037955621 | 0.744231292 | 0.8966652 | 1 |
| 298_30 | A_23_P148807 | NM_003503 | CDC7 | -0.290338823 | 0.056558178 | 0.423246544 | 1 | 0.244708768 | 0.088888973 | 0.321458 | 1 |
| 224_73 | A_23_P385861 | NM_152562 | CDCA2 | -0.170296402 | 0.201081706 | 0.648025996 | 1 | 0.109912001 | 0.437925142 | 0.7090907 | 1 |
| 229_2 | A_24_P218979 | NM_031299 | CDCA3 | -0.20763749 | 0.154821505 | 0.593652999 | 1 | -0.399903987 | 0.007964012 | 0.0807676 | 1 |
| 174_61 | A_23_P104651 | NM_080668 | CDCA5 | 0.130478683 | 0.333152222 | 0.76120935 | 1 | 0.025385741 | 0.850363285 | 0.9451344 | 1 |
| 318_141 | A_23_P251421 | NM_031942 | CDCA7 | 0.136916019 | 0.309632546 | 0.742362464 | 1 | 0.556448939 | 0.001211772 | 0.0253208 | 1 |
| 305_29 | A_19_P00808208 | NM_001127370 | CDCA7L | 0.10709218 | 0.424775655 | 0.819650834 | 1 | -0.075942322 | 0.574638695 | 0.803906 | 1 |
| 284_82 | A_24_P274795 | NM_018719 | CDCA7L | 0.29021412 | 0.075358424 | 0.461718925 | 1 | -0.377795455 | 0.024849828 | 0.1577235 | 1 |
| 191_32 | A_23_P375 | NM_018101 | CDCA8 | -0.162850425 | 0.317562928 | 0.748484528 | 1 | -0.071558085 | 0.529940116 | 0.7722514 | 1 |
| 173_109 | A_23_P113613 | NM_022842 | CDCP1 | 0.379797896 | 0.034068016 | 0.355727847 | 1 | -0.249896241 | 0.100651887 | 0.3445015 | 1 |
| 175_18 | A_23_P40192 | NM_021248 | CDH22 | -0.030630904 | 0.865686589 | 0.968303556 | 1 | 0.098882754 | 0.44809283 | 0.7166534 | 1 |
| 21_112 | A_33_P3333360 | NM_001199054 | CDIP1 | -0.036156068 | 0.779121797 | 0.947918804 | 1 | 0.045641836 | 0.783594146 | 0.9143719 | 1 |
| 375_1 | A_23_P138507 | NM_001786 | CDK1 | -0.305794781 | 0.057404657 | 0.423696939 | 1 | 0.107238338 | 0.474075061 | 0.7344245 | 1 |
| 157_6 | A_23_P138507 | NM_001786 | CDK1 | -0.153485241 | 0.287124873 | 0.72693776 | 1 | -0.054394063 | 0.717556537 | 0.8837108 | 1 |
| 330_5 | A_23_P138507 | NM_001786 | CDK1 | -0.152798194 | 0.234242012 | 0.680457734 | 1 | 0.060937435 | 0.678415219 | 0.8658802 | 1 |
| 58_122 | A_23_P138507 | NM_001786 | CDK1 | -0.008293528 | 0.947192809 | 0.99002169 | 1 | -0.001439218 | 0.991628026 | 0.9974469 | 1 |
| 275_115 | A_23_P138507 | NM_001786 | CDK1 | -0.005053384 | 0.971498594 | 0.994619938 | 1 | 0.030138755 | 0.794024227 | 0.9190059 | 1 |
| 312_77 | A_23_P138507 | NM_001786 | CDK1 | 0.047961215 | 0.70596795 | 0.927945366 | 1 | 0.032959847 | 0.793804669 | 0.9188885 | 1 |
| 320_154 | A_23_P138507 | NM_001786 | CDK1 | 0.064120131 | 0.626481124 | 0.900187904 | 1 | -0.035450559 | 0.7884694 | 0.9163904 | 1 |
| 103_107 | A_23_P138507 | NM_001786 | CDK1 | 0.070263724 | 0.575588804 | 0.883311119 | 1 | -0.031746729 | 0.798973875 | 0.9211015 | 1 |
| 217_77 | A_23_P138507 | NM_001786 | CDK1 | 0.080911114 | 0.609450516 | 0.896766057 | 1 | 0.081570739 | 0.602973729 | 0.8190176 | 1 |
| 159_101 | A_23_P138507 | NM_001786 | CDK1 | 0.210565 | 0.179944105 | 0.620483827 | 1 | -0.112295903 | 0.474651165 | 0.7346553 | 1 |
| 50_60 | A_33_P3230688 | NM_052988 | CDK10 | -0.212887185 | 0.181844115 | 0.622415463 | 1 | 0.113935266 | 0.505069671 | 0.7574025 | 1 |
| 153_61 | A_33_P3227264 | NM_024011 | CDK11A | 0.231385557 | 0.105328765 | 0.519100989 | 1 | 0.022692549 | 0.852837152 | 0.9462939 | 1 |
| 146_96 | A_33_P3313595 | NM_033487 | CDK11B | -0.093640155 | 0.478828467 | 0.84100638 | 1 | -0.017341359 | 0.909820131 | 0.9683682 | 1 |
| 254_14 | A_33_P3341239 | NM_006201 | CDK16 | -0.513938624 | 0.013882011 | 0.271753414 | 1 | -1.13192096 | 1.89996E-05 | 0.0021369 | 0.21369 |
| 185_98 | A_33_P3328026 | NM_015076 | CDK19 | -0.019288567 | 0.896928167 | 0.975758314 | 1 | 0.20038484 | 0.14169715 | 0.4083187 | 1 |
| 375_112 | A_23_P20752 | NM_001039803 | CDK20 | -0.324784617 | 0.028041791 | 0.333708589 | 1 | 0.010468428 | 0.932255861 | 0.9756731 | 1 |
| 236_68 | A_23_P139486 | NM_004642 | CDK2AP1 | 0.50876435 | 0.011494052 | 0.260745878 | 1 | -0.086214976 | 0.692668893 | 0.8719224 | 1 |
| 76_73 | A_33_P3329839 | NM_005851 | CDK2AP2 | -0.329031309 | 0.046417968 | 0.398494494 | 1 | -0.047178258 | 0.702843078 | 0.8761778 | 1 |
| 375_15 | A_22_P00023587 | NM_001258 | CDK3 | -0.664704055 | 0.013556691 | 0.270290692 | 1 | 0.14272495 | 0.503659456 | 0.7571515 | 1 |
| 240_55 | A_23_P24997 | NM_000075 | CDK4 | 0.202579546 | 0.231089256 | 0.677668275 | 1 | -0.469194168 | 0.105635527 | 0.3528191 | 1 |
| 174_104 | A_33_P3226985 | NM_003885 | CDK5R1 | 0.199175409 | 0.120245784 | 0.542933337 | 1 | -0.176033069 | 0.178327189 | 0.4568562 | 1 |
| 278_135 | A_33_P3213752 | NM_016408 | CDK5RAP1 | -0.079604987 | 0.61809427 | 0.89867047 | 1 | 0.185623052 | 0.230677344 | 0.5189894 | 1 |
| 20_123 | A_23_P83110 | NM_018249 | CDK5RAP2 | 0.038085731 | 0.782860461 | 0.948707004 | 1 | -0.136676767 | 0.28100271 | 0.5719214 | 1 |
| 42_77 | A_24_P166663 | NM_001259 | CDK6 | 0.57703519 | 0.023899854 | 0.317154707 | 1 | -0.277249072 | 0.030936046 | 0.1785211 | 1 |
| 123_126 | A_23_P133585 | NM_001799 | CDK7 | 0.12018316 | 0.427743715 | 0.820875237 | 1 | -0.379029809 | 0.036138946 | 0.1954109 | 1 |
| 55_74 | A_23_P428129 | NM_000076 | CDKN1C | 0.124264115 | 0.403569059 | 0.80585685 | 1 | 0.346218742 | 0.043875205 | 0.2165267 | 1 |
| 102_68 | A_33_P3355090 | NM_080656 | CDKN2AIPNL | -0.089910736 | 0.473470072 | 0.838824187 | 1 | 0.272829478 | 0.08018056 | 0.304762 | 1 |
| 323_94 | A_23_P250644 | NM_080656 | CDKN2AIPNL | 0.021487025 | 0.862630846 | 0.967572108 | 1 | 0.373525038 | 0.032995769 | 0.1854794 | 1 |
| 284_9 | A_33_P3292540 | NM_078626 | CDKN2C | -0.237639191 | 0.118646072 | 0.540937779 | 1 | -0.372932009 | 0.005209195 | 0.0618951 | 1 |
| 146_131 | A_23_P89941 | NM_001800 | CDKN2D | -0.392645368 | 0.013248611 | 0.268903942 | 1 | -0.318429249 | 0.022428695 | 0.1477771 | 1 |
| 165_161 | A_23_P48669 | NM_005192 | CDKN3 | 0.220028212 | 0.130996832 | 0.560349642 | 1 | -0.476084612 | 0.002575228 | 0.0397852 | 1 |
| 336_9 | A_33_P3281616 | NM_207327 | CDPF1 | 0.083747149 | 0.536326924 | 0.867927559 | 1 | -0.01679474 | 0.920350862 | 0.9713951 | 1 |
| 198_146 | A_33_P3288859 | NM_207327 | CDPF1 | 0.095913668 | 0.51585311 | 0.860004647 | 1 | -0.112499521 | 0.527600498 | 0.7708395 | 1 |
| 333_105 | A_23_P3963 | NM_014603 | CDR2L | 0.005007932 | 0.973714874 | 0.994783177 | 1 | -0.274374894 | 0.13508421 | 0.398959 | 1 |
| 231_159 | A_24_P271363 | NM_003818 | CDS2 | -0.026087895 | 0.856295737 | 0.965512529 | 1 | 0.08921688 | 0.507796555 | 0.7584579 | 1 |
| 35_161 | A_33_P3386262 | NM_030928 | CDT1 | -0.242599516 | 0.146501809 | 0.58152005 | 1 | 0.122881325 | 0.33011193 | 0.6166366 | 1 |
| 324_118 | A_24_P163113 | NM_017548 | CDV3 | -0.095671939 | 0.619368328 | 0.89867047 | 1 | 0.042876563 | 0.783079521 | 0.9143719 | 1 |
| 274_32 | A_23_P259413 | NM_017548 | CDV3 | 0.129460279 | 0.32435993 | 0.755083635 | 1 | -0.039034495 | 0.780671058 | 0.9131405 | 1 |
| 346_59 | A_33_P3289848 | NM_001804 | CDX1 | -0.031375918 | 0.798204063 | 0.951453193 | 1 | 0.143583938 | 0.226437151 | 0.5147763 | 1 |
| 269_63 | A_19_P00320729 | ENST00000570137 | CDYL2 | -0.062614354 | 0.68256219 | 0.920314282 | 1 | -0.83281519 | 9.1579E-05 | 0.0053092 | 1 |
| 71_97 | A_23_P78526 | NM_020219 | CEACAM19 | 0.064830424 | 0.737673298 | 0.939993317 | 1 | 0.180905905 | 0.352708094 | 0.6373932 | 1 |
| 352_14 | A_23_P411296 | NM_005194 | CEBPB | 0.162984505 | 0.408335435 | 0.809362557 | 1 | 0.232450801 | 0.063436304 | 0.2664375 | 1 |
| 296_9 | A_23_P411296 | NM_005194 | CEBPB | 0.191011136 | 0.32021398 | 0.752143943 | 1 | 0.303553791 | 0.045696974 | 0.2212239 | 1 |
| 87_38 | A_23_P411296 | NM_005194 | CEBPB | 0.210545594 | 0.28718574 | 0.72693776 | 1 | 0.306433135 | 0.043563332 | 0.2158946 | 1 |
| 353_47 | A_23_P411296 | NM_005194 | CEBPB | 0.235755294 | 0.210444967 | 0.65671122 | 1 | 0.253354877 | 0.065244182 | 0.2705757 | 1 |
| 108_10 | A_23_P411296 | NM_005194 | CEBPB | 0.275812093 | 0.169778297 | 0.609201536 | 1 | 0.251285382 | 0.145696111 | 0.4134858 | 1 |
| 286_48 | A_23_P411296 | NM_005194 | CEBPB | 0.281153609 | 0.145965453 | 0.58060515 | 1 | 0.29918347 | 0.026360417 | 0.1628369 | 1 |
| 102_142 | A_23_P411296 | NM_005194 | CEBPB | 0.345242762 | 0.113649813 | 0.533724756 | 1 | 0.203729583 | 0.123879714 | 0.3827679 | 1 |
| 38_155 | A_23_P411296 | NM_005194 | CEBPB | 0.444384787 | 0.028063074 | 0.333708589 | 1 | 0.16971037 | 0.150377992 | 0.4186389 | 1 |
| 311_118 | A_23_P411296 | NM_005194 | CEBPB | 0.447746003 | 0.021324852 | 0.308342332 | 1 | 0.235311972 | 0.062679077 | 0.2651191 | 1 |
| 39_153 | A_23_P411296 | NM_005194 | CEBPB | 0.478490204 | 0.023829224 | 0.316876636 | 1 | 0.18809638 | 0.12955809 | 0.3906541 | 1 |
| 329_46 | A_33_P3253804 | NM_005195 | CEBPD | -0.141163195 | 0.269556863 | 0.711900688 | 1 | 0.225186746 | 0.251113786 | 0.539087 | 1 |
| 236_142 | A_33_P3253807 | NM_001806 | CEBPG | -0.018425932 | 0.918303254 | 0.981381721 | 1 | -0.114842873 | 0.346051188 | 0.6321631 | 1 |
| 362_5 | A_23_P119964 | NM_005760 | CEBPZ | -0.187040488 | 0.164024033 | 0.603672445 | 1 | -0.018688176 | 0.885949983 | 0.9608259 | 1 |
| 136_6 | A_23_P119964 | NM_005760 | CEBPZ | -0.1769762 | 0.20146704 | 0.648025996 | 1 | -0.117760931 | 0.359610947 | 0.6442553 | 1 |
| 286_52 | A_23_P119964 | NM_005760 | CEBPZ | -0.146369384 | 0.321111418 | 0.752820065 | 1 | 0.048099996 | 0.747356303 | 0.8982827 | 1 |
| 241_32 | A_23_P119964 | NM_005760 | CEBPZ | -0.112521348 | 0.379773482 | 0.790179752 | 1 | -0.009799305 | 0.930870225 | 0.9749085 | 1 |
| 370_97 | A_23_P119964 | NM_005760 | CEBPZ | -0.028234814 | 0.836757728 | 0.962093689 | 1 | -0.097858017 | 0.400609105 | 0.6785094 | 1 |
| 141_137 | A_23_P119964 | NM_005760 | CEBPZ | 0.049142681 | 0.745865172 | 0.941470372 | 1 | -0.123172809 | 0.389362918 | 0.6700198 | 1 |
| 132_47 | A_23_P119964 | NM_005760 | CEBPZ | 0.076285824 | 0.560803289 | 0.877949216 | 1 | -0.048746403 | 0.688166632 | 0.8689581 | 1 |
| 47_59 | A_23_P119964 | NM_005760 | CEBPZ | 0.078194006 | 0.540912997 | 0.869568619 | 1 | -0.164751735 | 0.206463412 | 0.4896172 | 1 |
| 319_95 | A_23_P119964 | NM_005760 | CEBPZ | 0.091825159 | 0.514640925 | 0.859127182 | 1 | -0.076381694 | 0.542029262 | 0.7821558 | 1 |
| 268_85 | A_23_P119964 | NM_005760 | CEBPZ | 0.129810859 | 0.362194728 | 0.778646623 | 1 | -0.076619764 | 0.495310297 | 0.7507756 | 1 |
| 122_120 | A_33_P3272352 | ENST00000397064 | CEBPZOS | 0.140408156 | 0.38601038 | 0.795277691 | 1 | 0.504296862 | 0.05919082 | 0.2562669 | 1 |
| 86_115 | A_22_P00018851 | NM_001172684 | CELF6 | 0.065165267 | 0.650142531 | 0.908295601 | 1 | -0.083770284 | 0.552143044 | 0.788426 | 1 |
| 354_126 | A_33_P3328659 | NM_014246 | CELSR1 | -0.014369115 | 0.911574567 | 0.979345742 | 1 | 0.32450675 | 0.042092207 | 0.2126054 | 1 |
| 227_73 | A_23_P206454 | NM_145039 | CENPBD1 | 0.014551806 | 0.909153433 | 0.978706968 | 1 | 0.319450032 | 0.01994441 | 0.137768 | 1 |
| 122_134 | A_33_P3318187 | NM_145039 | CENPBD1 | 0.090368403 | 0.529346685 | 0.865814747 | 1 | 0.193139365 | 0.111930472 | 0.3631041 | 1 |
| 171_164 | A_21_P0011773 | ENST00000492393 | CENPBD1P1 | 0.030711053 | 0.808662696 | 0.953834349 | 1 | -0.206756537 | 0.10826484 | 0.3571312 | 1 |
| 254_155 | A_23_P253524 | NM_001813 | CENPE | -0.300384371 | 0.114104828 | 0.534625006 | 1 | -0.113782416 | 0.384565286 | 0.6656373 | 1 |
| 43_112 | A_23_P253524 | NM_001813 | CENPE | -0.173262533 | 0.304754688 | 0.738663272 | 1 | -0.048789097 | 0.71625358 | 0.883204 | 1 |
| 343_138 | A_23_P253524 | NM_001813 | CENPE | -0.166971909 | 0.323544354 | 0.753815848 | 1 | -0.151690988 | 0.259065416 | 0.5488243 | 1 |
| 303_138 | A_23_P253524 | NM_001813 | CENPE | -0.124404077 | 0.514686437 | 0.859127182 | 1 | -0.047256606 | 0.743843268 | 0.8965225 | 1 |
| 323_63 | A_23_P253524 | NM_001813 | CENPE | -0.123206856 | 0.477777644 | 0.840908892 | 1 | -0.01348129 | 0.925975176 | 0.9731305 | 1 |
| 12_3 | A_23_P253524 | NM_001813 | CENPE | -0.118479338 | 0.367535589 | 0.781989605 | 1 | -0.04164605 | 0.771247376 | 0.9087179 | 1 |
| 71_121 | A_23_P253524 | NM_001813 | CENPE | -0.099733431 | 0.573221287 | 0.882689231 | 1 | -0.103649246 | 0.436919666 | 0.7086536 | 1 |
| 27_152 | A_23_P253524 | NM_001813 | CENPE | -0.059028364 | 0.729983142 | 0.936838487 | 1 | -0.108625519 | 0.47141318 | 0.7325204 | 1 |
| 182_162 | A_23_P253524 | NM_001813 | CENPE | -0.027846111 | 0.869644773 | 0.969618777 | 1 | -0.144726502 | 0.27680683 | 0.5682143 | 1 |
| 275_120 | A_23_P253524 | NM_001813 | CENPE | 0.03258202 | 0.850748036 | 0.965069985 | 1 | -0.157797687 | 0.251309322 | 0.5393009 | 1 |
| 384_131 | A_23_P401 | NM_016343 | CENPF | -0.247222424 | 0.360434676 | 0.776988349 | 1 | 0.208750764 | 0.151141639 | 0.4192084 | 1 |
| 254_125 | A_32_P219116 | NM_018451 | CENPJ | 0.01740264 | 0.905900225 | 0.978062713 | 1 | -0.541172165 | 0.001649468 | 0.0298257 | 1 |
| 132_37 | A_23_P155989 | NM_022145 | CENPK | 0.11446449 | 0.376690681 | 0.789546778 | 1 | -0.022634959 | 0.852985463 | 0.9462939 | 1 |
| 300_8 | A_33_P3387831 | ENST00000402338 | CENPM | -0.313737641 | 0.053897987 | 0.417174905 | 1 | -0.210209945 | 0.137478404 | 0.4021377 | 1 |
| 62_64 | A_22_P00001744 | NM_001100624 | CENPN | 0.078843553 | 0.536227523 | 0.867927559 | 1 | -0.05773177 | 0.691176619 | 0.8712719 | 1 |
| 164_54 | A_23_P88740 | NM_018455 | CENPN | 0.369863418 | 0.036726375 | 0.369070022 | 1 | -0.108025387 | 0.347327745 | 0.6332299 | 1 |
| 372_34 | A_33_P3387931 | NM_001012267 | CENPP | -0.0853746 | 0.502402643 | 0.853951304 | 1 | -7.90144E-05 | 0.999484335 | 0.9995732 | 1 |
| 173_116 | A_33_P3245321 | NM_001012267 | CENPP | 0.298854642 | 0.075064109 | 0.461367795 | 1 | -0.073793968 | 0.495635275 | 0.7509404 | 1 |
| 66_161 | A_23_P254733 | NM_024629 | CENPU | -0.043597451 | 0.756066901 | 0.942463951 | 1 | 0.198778313 | 0.118032206 | 0.3730003 | 1 |
| 216_115 | A_24_P462899 | NM_001012507 | CENPW | 0.080417857 | 0.571647797 | 0.882279506 | 1 | -0.527495702 | 0.009525458 | 0.0890547 | 1 |
| 305_106 | A_23_P319270 | NM_014984 | CEP131 | -0.351927448 | 0.016644212 | 0.29132154 | 1 | -0.009734977 | 0.933745299 | 0.9764606 | 1 |
| 207_22 | A_23_P48550 | NM_015005 | CEP170B | -0.377213082 | 0.016600349 | 0.29132154 | 1 | -0.454750584 | 0.004690835 | 0.0577219 | 1 |
| 164_65 | A_23_P89710 | NM_032142 | CEP192 | 0.121655099 | 0.374756025 | 0.788476321 | 1 | 0.001126133 | 0.992298726 | 0.9974469 | 1 |
| 305_67 | A_23_P102832 | NM_007186 | CEP250 | -0.09175524 | 0.455046228 | 0.832937561 | 1 | 0.206730308 | 0.084976203 | 0.3139087 | 1 |
| 189_83 | A_23_P36865 | NM_025114 | CEP290 | -0.155008986 | 0.229145145 | 0.675404345 | 1 | 0.467783279 | 0.004915111 | 0.0596335 | 1 |
| 161_118 | A_23_P313734 | NM_033395 | CEP295 | 0.090657857 | 0.494515783 | 0.849672223 | 1 | 0.112975759 | 0.408417886 | 0.6851844 | 1 |
| 258_13 | A_23_P215070 | NM_018718 | CEP41 | -0.089845384 | 0.462471371 | 0.835837671 | 1 | -0.012488109 | 0.919866882 | 0.9713137 | 1 |
| 179_9 | A_23_P115872 | NM_018131 | CEP55 | -0.219580232 | 0.120777865 | 0.543077746 | 1 | -0.069375429 | 0.622234517 | 0.8294739 | 1 |
| 326_52 | A_23_P86822 | NM_014679 | CEP57 | -0.084297766 | 0.549524129 | 0.873756475 | 1 | 0.115155772 | 0.419260642 | 0.6952135 | 1 |
| 208_159 | A_23_P21409 | NM_001042384 | CEP63 | 0.182658381 | 0.17420175 | 0.614396559 | 1 | -0.164660157 | 0.251461187 | 0.5393181 | 1 |
| 271_156 | A_23_P302654 | NM_018140 | CEP72 | 0.070782156 | 0.614116162 | 0.897816866 | 1 | -0.59367446 | 0.000914689 | 0.0212154 | 1 |
| 121_61 | A_23_P307400 | NM_138363 | CEP95 | 0.226579363 | 0.104361815 | 0.517420159 | 1 | 0.073284465 | 0.557845956 | 0.792684 | 1 |
| 50_155 | A_33_P3260575 | NM_016174 | CERCAM | -0.570467427 | 0.001332944 | 0.120457642 | 1 | 0.147643493 | 0.377418079 | 0.6592361 | 1 |
| 378_154 | A_33_P3253832 | A_33_P3253832 | CERCAM | -0.546180519 | 0.003895387 | 0.184644372 | 1 | 0.092197245 | 0.511021171 | 0.7601476 | 1 |
| 161_41 | A_33_P3408320 | NM_198207 | CERS1 | 0.065549786 | 0.743673321 | 0.941128659 | 1 | 0.198976611 | 0.245482575 | 0.5327303 | 1 |
| 197_64 | A_33_P3408305 | NM_178842 | CERS3 | 0.039593003 | 0.786465055 | 0.94877096 | 1 | 0.070162698 | 0.67176799 | 0.8618742 | 1 |
| 232_111 | A_33_P3285038 | NM_001281731 | CERS5 | -0.142834444 | 0.26689117 | 0.710504423 | 1 | 0.133191071 | 0.406964417 | 0.683921 | 1 |
| 45_24 | A_33_P3399870 | NM_001256126 | CERS6 | -0.044655138 | 0.716849283 | 0.933574724 | 1 | 0.489797891 | 0.000989631 | 0.0221446 | 1 |
| 322_102 | A_23_P73493 | NM_004344 | CETN2 | 0.051179341 | 0.755280628 | 0.942463951 | 1 | 0.603547169 | 0.002049201 | 0.0342803 | 1 |
| 341_75 | A_23_P7732 | NM_004365 | CETN3 | -0.078803934 | 0.56322485 | 0.879190815 | 1 | 0.09184045 | 0.490561868 | 0.7464713 | 1 |
| 285_15 | A_23_P3514 | NM_013242 | CFAP20 | 0.171091683 | 0.198246436 | 0.644907571 | 1 | -0.104837893 | 0.485856136 | 0.7428526 | 1 |
| 288_25 | A_23_P68327 | NM_080667 | CFAP36 | -0.818692178 | 5.50913E-05 | 0.033556111 | 0.671122 | 0.073253859 | 0.591543821 | 0.8141328 | 1 |
| 159_45 | A_33_P3233160 | NM_001282761 | CFAP36 | -0.412415914 | 0.022642095 | 0.313438642 | 1 | 0.006020017 | 0.967225692 | 0.988121 | 1 |
| 86_97 | A_23_P318581 | NM_020827 | CFAP97 | 0.212078079 | 0.133358417 | 0.563305212 | 1 | 0.118333705 | 0.404566941 | 0.6827157 | 1 |
| 345_60 | A_23_P119562 | NM_001928 | CFD | -0.111412831 | 0.363655596 | 0.779606169 | 1 | -0.641770811 | 0.001299042 | 0.0259848 | 1 |
| 110_124 | A_23_P89123 | NM_006324 | CFDP1 | 0.12725671 | 0.313176813 | 0.744849655 | 1 | 0.07199535 | 0.506639825 | 0.7578372 | 1 |
| 198_19 | A_23_P35820 | NM_005507 | CFL1 | -0.259525175 | 0.086739393 | 0.481406415 | 1 | 0.248892436 | 0.075803801 | 0.2952096 | 1 |
| 313_90 | A_32_P148710 | NM_005507 | CFL1 | -0.132450312 | 0.347508497 | 0.769202821 | 1 | 0.102406781 | 0.485668769 | 0.7428526 | 1 |
| 155_117 | A_33_P3329974 | NM_020770 | CGN | 0.100202067 | 0.493837109 | 0.849586735 | 1 | 0.102376747 | 0.533218894 | 0.7741207 | 1 |
| 60_131 | A_21_P0000061 | NM_001301324 | CGREF1 | 0.113529773 | 0.421566724 | 0.817849907 | 1 | 0.350835626 | 0.037877247 | 0.2002846 | 1 |
| 68_100 | A_33_P3376971 | NM_024111 | CHAC1 | -0.22647625 | 0.271289584 | 0.714790846 | 1 | 0.331826084 | 0.145463606 | 0.4129301 | 1 |
| 164_67 | A_24_P53519 | NM_005483 | CHAF1A | 0.143419278 | 0.287654175 | 0.72693776 | 1 | -0.136348525 | 0.490612248 | 0.7464713 | 1 |
| 175_16 | A_23_P57306 | NM_005441 | CHAF1B | -0.054539566 | 0.695762563 | 0.924669503 | 1 | 0.117105045 | 0.28030166 | 0.5712948 | 1 |
| 315_36 | A_23_P57306 | NM_005441 | CHAF1B | -0.032868097 | 0.811658616 | 0.954788206 | 1 | 0.099945396 | 0.465237859 | 0.7272453 | 1 |
| 323_141 | A_23_P57306 | NM_005441 | CHAF1B | 0.029096778 | 0.820193064 | 0.956865726 | 1 | 0.059950804 | 0.584739627 | 0.8103212 | 1 |
| 100_73 | A_23_P57306 | NM_005441 | CHAF1B | 0.116582164 | 0.348739144 | 0.770211834 | 1 | 0.058395665 | 0.640061942 | 0.8426761 | 1 |
| 373_123 | A_23_P57306 | NM_005441 | CHAF1B | 0.121951001 | 0.351407485 | 0.771778562 | 1 | 0.003508311 | 0.973505123 | 0.9906597 | 1 |
| 298_144 | A_23_P57306 | NM_005441 | CHAF1B | 0.141034887 | 0.276145374 | 0.716856302 | 1 | 0.069891848 | 0.576623606 | 0.8048695 | 1 |
| 119_120 | A_23_P57306 | NM_005441 | CHAF1B | 0.15439742 | 0.215874635 | 0.662310915 | 1 | 0.044017253 | 0.690649994 | 0.8709206 | 1 |
| 332_69 | A_23_P57306 | NM_005441 | CHAF1B | 0.16179171 | 0.272652298 | 0.714985188 | 1 | 0.113416586 | 0.333048424 | 0.6197544 | 1 |
| 160_45 | A_23_P57306 | NM_005441 | CHAF1B | 0.166560962 | 0.244664397 | 0.69013282 | 1 | 0.113850809 | 0.316438796 | 0.6036274 | 1 |
| 10_113 | A_23_P57306 | NM_005441 | CHAF1B | 0.179861103 | 0.304923063 | 0.738777396 | 1 | 0.002761171 | 0.981525162 | 0.9934423 | 1 |
| 154_133 | A_23_P371613 | NM_203298 | CHCHD1 | 0.027673988 | 0.838392435 | 0.962281738 | 1 | -0.107744492 | 0.36187034 | 0.6467032 | 1 |
| 147_56 | A_24_P6083 | NM_213720 | CHCHD10 | 0.134121763 | 0.330682566 | 0.760452185 | 1 | -0.086368082 | 0.503073434 | 0.7567296 | 1 |
| 210_26 | A_24_P400376 | NM_016139 | CHCHD2 | 0.151374562 | 0.324804404 | 0.755540815 | 1 | -0.191463037 | 0.089424646 | 0.3226689 | 1 |
| 152_106 | A_23_P428849 | NM_144636 | CHCHD4 | 0.463721628 | 0.00714658 | 0.228180198 | 1 | 0.115212905 | 0.465598876 | 0.7276074 | 1 |
| 169_153 | A_23_P170626 | NM_001011667 | CHCHD7 | -0.237102715 | 0.10643924 | 0.521311656 | 1 | -0.258424628 | 0.038026008 | 0.2006938 | 1 |
| 108_112 | A_23_P338603 | NM_001011667 | CHCHD7 | -0.223097428 | 0.090473732 | 0.489192631 | 1 | 0.236518084 | 0.117714537 | 0.3722056 | 1 |
| 231_75 | A_33_P3239287 | NM_001005271 | CHD3 | -0.257283396 | 0.111392642 | 0.529451878 | 1 | 0.074071117 | 0.652047625 | 0.8490979 | 1 |
| 47_114 | A_24_P361167 | NM_020920 | CHD8 | -0.069929149 | 0.615346374 | 0.898145217 | 1 | 0.269014109 | 0.032999404 | 0.1854794 | 1 |
| 252_81 | A_33_P3349536 | NM_001114121 | CHEK1 | 0.142087944 | 0.262171573 | 0.704586552 | 1 | -0.382231381 | 0.037453102 | 0.1991655 | 1 |
| 164_156 | A_33_P3388501 | NM_003465 | CHIT1 | -0.061922298 | 0.694026222 | 0.924234842 | 1 | -0.11426246 | 0.424501391 | 0.6991498 | 1 |
| 297_24 | A_23_P124742 | NM_001277 | CHKA | -0.011891922 | 0.929772143 | 0.985082992 | 1 | -0.206605151 | 0.135577162 | 0.3996405 | 1 |
| 231_81 | A_24_P712350 | NM_001821 | CHML | -0.067126297 | 0.71644486 | 0.933387402 | 1 | -0.158979858 | 0.253133108 | 0.5411496 | 1 |
| 38_47 | A_23_P106694 | NM_002768 | CHMP1A | 0.09994261 | 0.478146908 | 0.840908892 | 1 | -0.027441817 | 0.863025029 | 0.9504938 | 1 |
| 122_138 | A_33_P3276475 | NM_020412 | CHMP1B | -0.176094977 | 0.184934092 | 0.627864254 | 1 | -0.063995354 | 0.598855655 | 0.8174528 | 1 |
| 211_30 | A_23_P153236 | NM_014453 | CHMP2A | -0.194775975 | 0.223810979 | 0.668378563 | 1 | 0.169624064 | 0.252157245 | 0.5401929 | 1 |
| 166_76 | A_24_P240065 | NM_016079 | CHMP3 | 0.150578815 | 0.4417179 | 0.828388614 | 1 | 0.258469769 | 0.100155389 | 0.3435195 | 1 |
| 320_88 | A_24_P142024 | NM_014169 | CHMP4A | 0.102945211 | 0.449338614 | 0.831477605 | 1 | -0.018815275 | 0.883326377 | 0.9596959 | 1 |
| 180_63 | A_23_P28969 | NM_176812 | CHMP4B | 0.264172257 | 0.126465807 | 0.551701995 | 1 | -0.052113325 | 0.663282552 | 0.8565307 | 1 |
| 306_19 | A_33_P3296303 | NM_016410 | CHMP5 | 0.043545732 | 0.88516168 | 0.972822545 | 1 | 0.294759364 | 0.180263242 | 0.4589599 | 1 |
| 93_108 | A_23_P83278 | NM_016410 | CHMP5 | 0.351439111 | 0.289765996 | 0.727621555 | 1 | 0.187945476 | 0.389136475 | 0.6699575 | 1 |
| 341_146 | A_23_P138805 | NM_012124 | CHORDC1 | 0.398885013 | 0.016281608 | 0.288487582 | 1 | -0.266348035 | 0.093334608 | 0.3316513 | 1 |
| 298_16 | A_23_P112801 | NM_007236 | CHP1 | -0.578991411 | 0.001036 | 0.112683515 | 1 | 0.252525376 | 0.119020947 | 0.3744416 | 1 |
| 83_40 | A_23_P112801 | NM_007236 | CHP1 | -0.525060766 | 0.001202286 | 0.116240035 | 1 | 0.099556098 | 0.484325607 | 0.7420402 | 1 |
| 209_11 | A_23_P112801 | NM_007236 | CHP1 | -0.399381516 | 0.011353896 | 0.25949936 | 1 | 0.054568164 | 0.757097518 | 0.9026672 | 1 |
| 238_142 | A_23_P112801 | NM_007236 | CHP1 | -0.381112345 | 0.018696336 | 0.299872244 | 1 | 0.022764713 | 0.878940552 | 0.9582221 | 1 |
| 298_61 | A_23_P112801 | NM_007236 | CHP1 | -0.379730711 | 0.012643877 | 0.265846757 | 1 | 0.19525258 | 0.244682848 | 0.5321113 | 1 |
| 283_145 | A_23_P112801 | NM_007236 | CHP1 | -0.371469274 | 0.012491906 | 0.265846757 | 1 | 0.07336607 | 0.641611315 | 0.8436649 | 1 |
| 358_94 | A_23_P112801 | NM_007236 | CHP1 | -0.305347687 | 0.034176443 | 0.355727847 | 1 | 0.149424872 | 0.331780798 | 0.6185212 | 1 |
| 229_68 | A_23_P112801 | NM_007236 | CHP1 | -0.259671457 | 0.071596134 | 0.455959826 | 1 | 0.149418054 | 0.396588437 | 0.6759357 | 1 |
| 206_69 | A_23_P112801 | NM_007236 | CHP1 | -0.23099927 | 0.120901892 | 0.543077746 | 1 | 0.172904955 | 0.35810512 | 0.6429771 | 1 |
| 128_88 | A_23_P112801 | NM_007236 | CHP1 | -0.192529248 | 0.135408124 | 0.566463518 | 1 | 0.078111987 | 0.617798283 | 0.8266957 | 1 |
| 377_102 | A_33_P3316878 | NM_024536 | CHPF | -0.391400805 | 0.011671663 | 0.26314611 | 1 | 0.554400501 | 0.001163163 | 0.0244983 | 1 |
| 83_92 | A_33_P3262012 | THC2603411 | CHPF | -0.033687085 | 0.792861553 | 0.94877096 | 1 | 0.349380874 | 0.027232502 | 0.1658278 | 1 |
| 159_150 | A_33_P3400263 | NM_017444 | CHRAC1 | -0.124926299 | 0.360269696 | 0.776779723 | 1 | -0.014866206 | 0.914784558 | 0.9704901 | 1 |
| 28_49 | A_33_P3373459 | NM_018641 | CHST12 | 0.037978573 | 0.758374136 | 0.942483836 | 1 | -0.010451629 | 0.936592033 | 0.9776195 | 1 |
| 208_49 | A_23_P80473 | NM_152889 | CHST13 | 0.029051214 | 0.826779978 | 0.959621444 | 1 | 0.031701256 | 0.7981422 | 0.9207822 | 1 |
| 221_74 | A_23_P383986 | NM_015892 | CHST15 | 0.351410641 | 0.021071168 | 0.308342332 | 1 | 0.278239687 | 0.025675849 | 0.1603422 | 1 |
| 109_129 | A_33_P3366903 | NM_021615 | CHST6 | 0.084319863 | 0.537578295 | 0.868287105 | 1 | 0.123226853 | 0.327413196 | 0.6136338 | 1 |
| 24_84 | A_23_P319617 | NM_019886 | CHST7 | 0.257624024 | 0.127458902 | 0.553674154 | 1 | -0.336546584 | 0.009978102 | 0.0914619 | 1 |
| 21_151 | A_23_P37484 | NM_014918 | CHSY1 | 0.413493718 | 0.071125944 | 0.455789714 | 1 | 0.115215162 | 0.481696315 | 0.7400134 | 1 |
| 40_114 | A_23_P354297 | NM_022092 | CHTF18 | -0.038810778 | 0.830218337 | 0.959829153 | 1 | -0.339484415 | 0.092793195 | 0.3305766 | 1 |
| 69_53 | A_21_P0013760 | NM_001039690 | CHTF8 | 0.021931929 | 0.883627755 | 0.972820007 | 1 | 0.048020136 | 0.690966241 | 0.871124 | 1 |
| 79_1 | A_23_P138253 | NM_015607 | CHTOP | -0.02412527 | 0.864987187 | 0.968067563 | 1 | -0.211669596 | 0.105956945 | 0.3531119 | 1 |
| 67_162 | A_33_P3258141 | NM_001278 | CHUK | -0.104669177 | 0.468092488 | 0.836476951 | 1 | -0.024196441 | 0.85717818 | 0.9488469 | 1 |
| 139_92 | A_33_P3306397 | NM_004804 | CIAO1 | 0.095623464 | 0.503660901 | 0.85467065 | 1 | -0.017371896 | 0.90282589 | 0.965676 | 1 |
| 148_114 | A_23_P88781 | NM_020313 | CIAPIN1 | 0.382096662 | 0.009933188 | 0.245874392 | 1 | -0.053003191 | 0.726977637 | 0.888579 | 1 |
| 363_31 | A_24_P44514 | NM_006384 | CIB1 | 0.224213057 | 0.119172492 | 0.541632465 | 1 | -0.195960473 | 0.1194765 | 0.3751402 | 1 |
| 166_39 | A_19_P00810474 | ENST00000572681 | CIC | -0.07093326 | 0.568646432 | 0.881307748 | 1 | 0.050204071 | 0.698958714 | 0.874605 | 1 |
| 17_71 | A_33_P3240767 | NM_022094 | CIDEC | 0.185115465 | 0.254681714 | 0.698225406 | 1 | -0.013301269 | 0.909385213 | 0.9680883 | 1 |
| 344_58 | A_23_P372925 | NM_033426 | CIPC | 0.144502964 | 0.336275935 | 0.762411026 | 1 | -0.146137848 | 0.261942644 | 0.5525038 | 1 |
| 312_64 | A_23_P39814 | NM_004882 | CIR1 | -0.111817487 | 0.361945757 | 0.77846455 | 1 | 0.46026757 | 0.00206571 | 0.0342805 | 1 |
| 289_76 | A_23_P142322 | NM_001280 | CIRBP | -0.079264631 | 0.533284533 | 0.866842045 | 1 | 0.23357637 | 0.120233335 | 0.3763608 | 1 |
| 218_148 | A_33_P3851788 | NR_027271 | CIRBP-AS1 | 0.194985227 | 0.149969359 | 0.586681674 | 1 | -0.281970482 | 0.049087619 | 0.2311088 | 1 |
| 370_13 | A_23_P35467 | NM_018464 | CISD1 | -0.22142152 | 0.135912026 | 0.566743973 | 1 | 0.026521872 | 0.843074934 | 0.9414469 | 1 |
| 44_49 | A_33_P3362641 | NM_001008388 | CISD2 | -0.120685655 | 0.356445175 | 0.774565665 | 1 | 0.103670752 | 0.396993269 | 0.6759879 | 1 |
| 198_50 | A_33_P3362636 | NM_001136498 | CISD3 | -0.056953444 | 0.730309282 | 0.936838487 | 1 | 0.140178558 | 0.276005146 | 0.5678047 | 1 |
| 39_2 | A_23_P420551 | NM_007174 | CIT | -0.579486914 | 0.005120155 | 0.202512103 | 1 | -0.023026849 | 0.872551211 | 0.9556513 | 1 |
| 148_81 | A_32_P209230 | NM_133467 | CITED4 | -0.323008949 | 0.050608945 | 0.406194685 | 1 | 0.324449731 | 0.027828829 | 0.1673964 | 1 |
| 253_19 | A_23_P32223 | NM_012127 | CIZ1 | 0.041300533 | 0.768641018 | 0.945351992 | 1 | -0.251372004 | 0.063674687 | 0.2665237 | 1 |
| 207_5 | A_23_P151405 | NM_018204 | CKAP2 | -0.374269197 | 0.01977175 | 0.306116626 | 1 | -0.13055877 | 0.350527119 | 0.635765 | 1 |
| 276_161 | A_23_P135977 | NM_001008938 | CKAP5 | 0.209416732 | 0.222334275 | 0.667441137 | 1 | -0.249187551 | 0.194367203 | 0.4763669 | 1 |
| 319_14 | A_23_P206396 | NM_001040138 | CKLF | -0.180838375 | 0.177246479 | 0.616390694 | 1 | 0.053559178 | 0.641662064 | 0.8436649 | 1 |
| 293_74 | A_23_P118061 | NM_181641 | CKLF | 0.051641906 | 0.711921064 | 0.93083851 | 1 | 0.03812962 | 0.746011137 | 0.8976273 | 1 |
| 275_122 | A_33_P3316348 | THC2631252 | CKMT1A; CKMT1B | 0.047220083 | 0.81530066 | 0.95582645 | 1 | -0.107368827 | 0.586986121 | 0.8110447 | 1 |
| 240_82 | A_23_P45917 | NM_001826 | CKS1B | 0.084739998 | 0.5549295 | 0.875569848 | 1 | 0.154120043 | 0.358494695 | 0.6432023 | 1 |
| 292_122 | A_23_P71727 | NM_001827 | CKS2 | 0.130035183 | 0.429116101 | 0.821087055 | 1 | -0.257256014 | 0.040260099 | 0.2066848 | 1 |
| 233_58 | A_23_P44295 | NM_015097 | CLASP2 | -0.032977923 | 0.810847413 | 0.954555778 | 1 | 0.148648879 | 0.254711033 | 0.5434266 | 1 |
| 83_102 | A_33_P3236403 | NM_007056 | CLASRP | -0.2171399 | 0.290493242 | 0.728295672 | 1 | 0.085215136 | 0.594177467 | 0.8154033 | 1 |
| 77_158 | A_24_P302374 | NM_001286 | CLCN6 | -0.065027451 | 0.595054782 | 0.889988626 | 1 | 0.004622651 | 0.964844232 | 0.9873043 | 1 |
| 225_111 | A_33_P3299739 | NM_001123395 | CLDN19 | 0.186778362 | 0.3276332 | 0.758096222 | 1 | 0.023763693 | 0.903969225 | 0.9663475 | 1 |
| 282_137 | A_33_P3285545 | NM_001305 | CLDN4 | -0.028912107 | 0.875178861 | 0.970750688 | 1 | -0.247495582 | 0.134781348 | 0.3986026 | 1 |
| 328_65 | A_23_P155556 | NM_001040199 | CLDND1 | 0.144917919 | 0.277656909 | 0.717982693 | 1 | 0.02890966 | 0.796271417 | 0.9198135 | 1 |
| 199_33 | A_32_P194246 | NM_015226 | CLEC16A | -0.020028655 | 0.878126735 | 0.971550769 | 1 | -0.000976894 | 0.993145966 | 0.9974469 | 1 |
| 113_62 | A_32_P80068 | NM_001004419 | CLEC2D | -0.066949872 | 0.668329278 | 0.916432605 | 1 | -0.080171352 | 0.551733083 | 0.7883804 | 1 |
| 249_87 | A_19_P00800467 | NM_001004419 | CLEC2D | 0.525729781 | 0.096002728 | 0.499788561 | 1 | -0.399217497 | 0.153554879 | 0.4221273 | 1 |
| 255_35 | A_33_P3274105 | ENST00000403506 | CLHC1 | 0.033203515 | 0.835895907 | 0.961943507 | 1 | 0.043559378 | 0.803798686 | 0.9231312 | 1 |
| 70_65 | A_23_P30884 | NM_001288 | CLIC1 | 0.223600327 | 0.237684976 | 0.683441761 | 1 | -0.49423529 | 0.051786977 | 0.2380254 | 1 |
| 191_102 | A_33_P3336686 | NM_004669 | CLIC3 | -0.239938761 | 0.168067359 | 0.607928988 | 1 | 0.76082177 | 0.002894399 | 0.043003 | 1 |
| 290_8 | A_23_P259189 | NM_013943 | CLIC4 | -0.300026134 | 0.036250114 | 0.367344178 | 1 | 0.188272285 | 0.223412449 | 0.5117556 | 1 |
| 53_136 | A_33_P3373364 | NM_013943 | CLIC4 | 0.126957741 | 0.371856752 | 0.78563284 | 1 | 0.328194395 | 0.094261255 | 0.3327635 | 1 |
| 184_140 | A_33_P3392177 | NM_001256023 | CLIC5 | 0.359110181 | 0.094827382 | 0.498686523 | 1 | -0.432730495 | 0.030901795 | 0.1784713 | 1 |
| 89_130 | A_23_P133345 | NM_014666 | CLINT1 | -0.058540281 | 0.677119732 | 0.919041495 | 1 | 0.034878244 | 0.745187256 | 0.8974324 | 1 |
| 327_137 | A_23_P25003 | NM_002956 | CLIP1 | -0.221170148 | 0.116355745 | 0.538543195 | 1 | 0.031355462 | 0.826961478 | 0.9338627 | 1 |
| 380_147 | A_32_P20691 | NM_003992 | CLK3 | -0.022575387 | 0.895866748 | 0.975468897 | 1 | -0.088116559 | 0.54099068 | 0.7812689 | 1 |
| 269_99 | A_33_P3365805 | NM_006493 | CLN5 | -0.050667439 | 0.714570643 | 0.932537343 | 1 | -0.241564754 | 0.131847388 | 0.3936521 | 1 |
| 183_40 | A_23_P127995 | NM_001293 | CLNS1A | 0.180386799 | 0.223914266 | 0.668378563 | 1 | 0.07577979 | 0.525098 | 0.769761 | 1 |
| 181_65 | A_23_P75978 | NM_030813 | CLPB | 0.111371453 | 0.479307548 | 0.841586126 | 1 | -0.15303671 | 0.43520061 | 0.7078419 | 1 |
| 165_112 | A_23_P4754 | NM_006012 | CLPP | 0.179105192 | 0.216004044 | 0.662310915 | 1 | -0.171697524 | 0.272644498 | 0.5632683 | 1 |
| 66_135 | A_23_P140511 | NM_006660 | CLPX | 0.179471173 | 0.236173476 | 0.682092291 | 1 | -0.112547809 | 0.344818455 | 0.6313683 | 1 |
| 86_100 | A_33_P3394347 | NR_024066 | CLRN1-AS1 | 0.004733192 | 0.976022608 | 0.995150467 | 1 | 0.095879481 | 0.612777632 | 0.8237014 | 1 |
| 337_129 | A_24_P191067 | NM_001009566 | CLSTN1 | 0.147497447 | 0.288518237 | 0.727382494 | 1 | -0.025896291 | 0.819571382 | 0.9307067 | 1 |
| 321_59 | A_33_P3269803 | NM_014718 | CLSTN3 | -0.633114689 | 0.000830853 | 0.098266543 | 1 | 0.559080458 | 0.012498934 | 0.1048289 | 1 |
| 111_66 | A_23_P219144 | NM_007096 | CLTA | 0.207250275 | 0.3120526 | 0.743773338 | 1 | -0.264049845 | 0.167836584 | 0.4448345 | 1 |
| 369_115 | A_23_P77714 | NM_024793 | CLUAP1 | -0.072328297 | 0.647187637 | 0.907045535 | 1 | 0.233765572 | 0.100738109 | 0.3446917 | 1 |
| 41_84 | A_24_P61753 | NM_015229 | CLUH | 0.30244044 | 0.173737918 | 0.613752707 | 1 | 0.0499188 | 0.783708483 | 0.9143719 | 1 |
| 239_105 | A_24_P408740 | NM_182523 | CMC1 | 0.07860328 | 0.582192041 | 0.883808109 | 1 | 0.316699304 | 0.081183204 | 0.3071199 | 1 |
| 96_103 | A_23_P106544 | NM_020188 | CMC2 | 0.195630772 | 0.157507251 | 0.595969916 | 1 | -0.315500425 | 0.025820551 | 0.1607994 | 1 |
| 336_29 | A_23_P11295 | NM_001018024 | CMC4 | -0.208141863 | 0.120621836 | 0.543014125 | 1 | 0.542295543 | 0.001186338 | 0.0249397 | 1 |
| 245_135 | A_23_P11295 | NM_001018024 | CMC4 | -0.173728757 | 0.27448443 | 0.716566978 | 1 | 0.478878752 | 0.001286354 | 0.0259848 | 1 |
| 283_32 | A_23_P11295 | NM_001018024 | CMC4 | -0.16734291 | 0.219713267 | 0.666470871 | 1 | 0.568416405 | 0.000259779 | 0.0093346 | 1 |
| 362_121 | A_23_P11295 | NM_001018024 | CMC4 | -0.157470508 | 0.309557041 | 0.742327535 | 1 | 0.571732992 | 0.000332847 | 0.0108824 | 1 |
| 21_40 | A_23_P11295 | NM_001018024 | CMC4 | -0.077241226 | 0.567702494 | 0.881090627 | 1 | 0.615746216 | 0.000511431 | 0.0145622 | 1 |
| 165_160 | A_23_P11295 | NM_001018024 | CMC4 | -0.063418898 | 0.616484694 | 0.89867047 | 1 | 0.467170305 | 0.001747061 | 0.0310042 | 1 |
| 235_85 | A_23_P11295 | NM_001018024 | CMC4 | -0.058032539 | 0.705975838 | 0.927945366 | 1 | 0.752291464 | 5.79283E-05 | 0.004072 | 0.65152 |
| 248_143 | A_23_P11295 | NM_001018024 | CMC4 | -0.049295293 | 0.703487736 | 0.926847254 | 1 | 0.50547033 | 0.000940767 | 0.0215057 | 1 |
| 117_51 | A_23_P11295 | NM_001018024 | CMC4 | -0.02599541 | 0.863866567 | 0.967689428 | 1 | 0.57104374 | 0.000611916 | 0.0165603 | 1 |
| 98_138 | A_23_P11295 | NM_001018024 | CMC4 | 0.028351987 | 0.858189303 | 0.965948636 | 1 | 0.386800276 | 0.005116684 | 0.0610258 | 1 |
| 372_85 | A_23_P132874 | NM_032359 | CMSS1 | 0.003800616 | 0.978444103 | 0.995429381 | 1 | 0.137038919 | 0.34327661 | 0.6297231 | 1 |
| 233_120 | A_33_P3340342 | NM_144601 | CMTM3 | 0.359133164 | 0.026032062 | 0.325922491 | 1 | 0.103159547 | 0.404639696 | 0.6827157 | 1 |
| 229_70 | A_23_P88865 | NM_144601 | CMTM3 | 0.382352859 | 0.012902336 | 0.267459687 | 1 | 0.448497016 | 0.078981124 | 0.3028642 | 1 |
| 275_3 | A_32_P84009 | NM_181521 | CMTM4 | -0.024889631 | 0.855162005 | 0.965512529 | 1 | -0.307184923 | 0.065183449 | 0.2704236 | 1 |
| 75_26 | A_23_P256413 | NM_138410 | CMTM7 | 0.021073726 | 0.877231675 | 0.971233905 | 1 | -0.042549016 | 0.711959617 | 0.8804727 | 1 |
| 225_36 | A_23_P84651 | NM_003418 | CNBP | 0.281474713 | 0.122039383 | 0.544772359 | 1 | -0.045649178 | 0.78541737 | 0.9151101 | 1 |
| 291_148 | A_33_P3411848 | NM_032488 | CNFN | -0.400652222 | 0.02039858 | 0.306188729 | 1 | 0.12837191 | 0.312364106 | 0.6008481 | 1 |
| 304_90 | A_23_P14473 | NM_005776 | CNIH1 | 0.392963383 | 0.019984097 | 0.306116626 | 1 | 0.141846265 | 0.25288195 | 0.5409211 | 1 |
| 384_147 | A_33_P3318746 | NM_001277200 | CNIH4 | -0.123802468 | 0.377489889 | 0.789727259 | 1 | -0.07211008 | 0.616442632 | 0.826061 | 1 |
| 158_43 | A_23_P200507 | NM_014184 | CNIH4 | -0.030186477 | 0.861219081 | 0.967258331 | 1 | -0.140375097 | 0.248531898 | 0.5355889 | 1 |
| 329_76 | A_23_P134085 | NM_173515 | CNKSR3 | -0.055436299 | 0.654656814 | 0.909665926 | 1 | -0.173905204 | 0.194559245 | 0.4766182 | 1 |
| 175_7 | A_24_P142743 | ENST00000564572 | CNN2 | -0.696527293 | 0.000376015 | 0.067362008 | 1 | 0.008321463 | 0.950677218 | 0.9828354 | 1 |
| 217_31 | A_23_P138168 | NM_001839 | CNN3 | -0.098487792 | 0.44420231 | 0.829470233 | 1 | -0.336553924 | 0.00900414 | 0.0864813 | 1 |
| 355_7 | A_33_P3255274 | NM_017623 | CNNM3 | -0.234495247 | 0.212749527 | 0.659525123 | 1 | 0.240848421 | 0.180307005 | 0.4589599 | 1 |
| 87_37 | A_23_P119923 | NM_020184 | CNNM4 | -0.252902675 | 0.072347226 | 0.457123394 | 1 | 0.137914593 | 0.234511112 | 0.522183 | 1 |
| 49_35 | A_23_P119923 | NM_020184 | CNNM4 | -0.177485414 | 0.18579059 | 0.629215338 | 1 | 0.039034581 | 0.730322457 | 0.8904962 | 1 |
| 122_41 | A_23_P119923 | NM_020184 | CNNM4 | -0.148295885 | 0.292223396 | 0.729622538 | 1 | 0.098024474 | 0.381435979 | 0.6634721 | 1 |
| 277_104 | A_23_P119923 | NM_020184 | CNNM4 | -0.092009174 | 0.531953641 | 0.86624761 | 1 | 0.074988192 | 0.478744848 | 0.7373874 | 1 |
| 177_77 | A_23_P119923 | NM_020184 | CNNM4 | -0.08205308 | 0.597325403 | 0.89144727 | 1 | 0.150545004 | 0.241592398 | 0.5293736 | 1 |
| 109_89 | A_23_P119923 | NM_020184 | CNNM4 | -0.041980079 | 0.751663242 | 0.942109358 | 1 | 0.1725208 | 0.145349146 | 0.4127832 | 1 |
| 266_89 | A_23_P119923 | NM_020184 | CNNM4 | -0.038105721 | 0.791748518 | 0.94877096 | 1 | 0.097095947 | 0.404783632 | 0.682719 | 1 |
| 39_107 | A_23_P119923 | NM_020184 | CNNM4 | -0.013894534 | 0.911705521 | 0.979400058 | 1 | 0.070528021 | 0.533755155 | 0.7744394 | 1 |
| 18_97 | A_23_P119923 | NM_020184 | CNNM4 | 0.080133447 | 0.589304125 | 0.88697963 | 1 | 0.088233567 | 0.475724153 | 0.7355282 | 1 |
| 168_109 | A_23_P119923 | NM_020184 | CNNM4 | 0.119353273 | 0.345999963 | 0.768529418 | 1 | 0.017832225 | 0.893246632 | 0.9630927 | 1 |
| 384_17 | A_23_P112652 | NM_015442 | CNOT10 | -0.325569296 | 0.076864715 | 0.464296843 | 1 | 0.203118814 | 0.204149898 | 0.4868207 | 1 |
| 357_130 | A_23_P112652 | NM_015442 | CNOT10 | -0.292329127 | 0.075131373 | 0.461548351 | 1 | 0.161636848 | 0.181918525 | 0.4606308 | 1 |
| 362_37 | A_23_P112652 | NM_015442 | CNOT10 | -0.288206988 | 0.071739943 | 0.456118548 | 1 | 0.169784495 | 0.235776495 | 0.5235083 | 1 |
| 369_141 | A_23_P112652 | NM_015442 | CNOT10 | -0.233694802 | 0.121589319 | 0.544159104 | 1 | 0.058068035 | 0.613213865 | 0.8239879 | 1 |
| 350_147 | A_23_P112652 | NM_015442 | CNOT10 | -0.227136479 | 0.124325411 | 0.549340643 | 1 | 0.063182208 | 0.623616447 | 0.8306489 | 1 |
| 316_149 | A_23_P112652 | NM_015442 | CNOT10 | -0.221339806 | 0.10883002 | 0.525889451 | 1 | 0.116898651 | 0.354851121 | 0.6398927 | 1 |
| 254_150 | A_23_P112652 | NM_015442 | CNOT10 | -0.205444729 | 0.212778529 | 0.659525123 | 1 | 0.081510428 | 0.527046909 | 0.7704311 | 1 |
| 83_61 | A_23_P112652 | NM_015442 | CNOT10 | -0.192275906 | 0.226489437 | 0.671475863 | 1 | 0.172033216 | 0.145338715 | 0.4127832 | 1 |
| 117_162 | A_23_P112652 | NM_015442 | CNOT10 | -0.168330846 | 0.192887316 | 0.637653537 | 1 | 0.095449963 | 0.423592505 | 0.6991498 | 1 |
| 96_107 | A_23_P112652 | NM_015442 | CNOT10 | -0.137617658 | 0.347142294 | 0.768954722 | 1 | 0.053999911 | 0.700434969 | 0.8753102 | 1 |
| 370_18 | A_23_P108761 | NM_017546 | CNOT11 | -0.147111472 | 0.306631312 | 0.739887567 | 1 | -0.015678871 | 0.896873218 | 0.963432 | 1 |
| 322_70 | A_23_P108761 | NM_017546 | CNOT11 | -0.140078467 | 0.314209071 | 0.745268067 | 1 | -0.089632118 | 0.502798417 | 0.7566195 | 1 |
| 78_160 | A_23_P108761 | NM_017546 | CNOT11 | -0.119622108 | 0.442715944 | 0.829030718 | 1 | -0.082412407 | 0.468193279 | 0.7297579 | 1 |
| 333_66 | A_23_P108761 | NM_017546 | CNOT11 | -0.113936356 | 0.4129814 | 0.812009855 | 1 | 0.097499038 | 0.476131702 | 0.7355842 | 1 |
| 205_79 | A_23_P108761 | NM_017546 | CNOT11 | -0.084065471 | 0.519505125 | 0.861504415 | 1 | 0.034466111 | 0.760351554 | 0.9039933 | 1 |
| 101_161 | A_23_P108761 | NM_017546 | CNOT11 | -0.075577684 | 0.575664632 | 0.883311119 | 1 | -0.07702545 | 0.491335291 | 0.7470324 | 1 |
| 238_88 | A_23_P108761 | NM_017546 | CNOT11 | -0.029069102 | 0.827719331 | 0.959690637 | 1 | 0.034125743 | 0.758182654 | 0.9031132 | 1 |
| 101_125 | A_23_P108761 | NM_017546 | CNOT11 | -0.011696648 | 0.926508078 | 0.984072534 | 1 | -0.03440404 | 0.768754972 | 0.9077701 | 1 |
| 174_65 | A_23_P108761 | NM_017546 | CNOT11 | -0.00771804 | 0.948693634 | 0.990231079 | 1 | 0.070365605 | 0.555613434 | 0.7907104 | 1 |
| 229_143 | A_23_P108761 | NM_017546 | CNOT11 | 0.097257614 | 0.505172678 | 0.85467065 | 1 | 0.023381329 | 0.838992014 | 0.9393908 | 1 |
| 135_4 | A_23_P162336 | NM_014515 | CNOT2 | -0.253090016 | 0.100309723 | 0.507674719 | 1 | 0.187758636 | 0.201316636 | 0.4835985 | 1 |
| 264_47 | A_33_P3287685 | NM_014516 | CNOT3 | -0.192598051 | 0.173230113 | 0.613354412 | 1 | 0.112048683 | 0.319215294 | 0.6059183 | 1 |
| 320_57 | A_23_P300781 | NM_013316 | CNOT4 | -0.091254301 | 0.471445021 | 0.838282962 | 1 | 0.08787388 | 0.490250255 | 0.7464713 | 1 |
| 301_55 | A_33_P3249214 | NM_001286790 | CNOT6L | -0.053897708 | 0.754105102 | 0.942109358 | 1 | 0.044704649 | 0.738470595 | 0.8939381 | 1 |
| 155_34 | A_23_P394166 | NM_013354 | CNOT7 | 0.028544894 | 0.850879701 | 0.965069985 | 1 | -0.05230817 | 0.650650965 | 0.8485472 | 1 |
| 226_116 | A_32_P141262 | NM_054026 | CNOT7 | 0.094315543 | 0.456073142 | 0.833432512 | 1 | -0.098532096 | 0.441661549 | 0.7115701 | 1 |
| 99_85 | A_23_P21838 | NM_033133 | CNP | -0.078626351 | 0.528782668 | 0.865741443 | 1 | -0.191465136 | 0.172365954 | 0.4501833 | 1 |
| 51_155 | A_23_P53288 | NM_014255 | CNPY2 | -0.011159079 | 0.933492309 | 0.986418035 | 1 | -0.150957016 | 0.231029011 | 0.5195151 | 1 |
| 313_10 | A_21_P0000115 | NM_001190991 | CNPY2 | 0.143199657 | 0.284345405 | 0.725024946 | 1 | -0.194832646 | 0.118878095 | 0.3740968 | 1 |
| 332_91 | A_33_P3424467 | NM_152609 | CNST | 0.012794768 | 0.920617432 | 0.981873714 | 1 | 0.121250376 | 0.307574627 | 0.5961655 | 1 |
| 271_79 | A_23_P9761 | NM_001037144 | CNTROB | -0.18064559 | 0.194927579 | 0.640909226 | 1 | -0.08725714 | 0.425673291 | 0.6996668 | 1 |
| 17_17 | A_23_P215675 | NM_018224 | COA1 | 0.254529313 | 0.069028715 | 0.452344164 | 1 | -0.306852025 | 0.026310743 | 0.1627414 | 1 |
| 313_133 | A_24_P356453 | NM_001040431 | COA3 | 0.311928995 | 0.032926802 | 0.351238438 | 1 | -0.315082438 | 0.059897379 | 0.2574866 | 1 |
| 70_60 | A_33_P3278435 | NM_016565 | COA4 | 0.163586188 | 0.240601634 | 0.685616166 | 1 | -0.038516308 | 0.744464972 | 0.8968506 | 1 |
| 199_122 | A_33_P3351982 | NM_001008215 | COA5 | 0.152087953 | 0.226998206 | 0.672329722 | 1 | -0.029440569 | 0.803571235 | 0.9231312 | 1 |
| 259_60 | A_23_P63459 | NM_001012985 | COA6 | 0.274648712 | 0.062754937 | 0.434858155 | 1 | -0.030738059 | 0.782258154 | 0.9139162 | 1 |
| 156_98 | A_23_P347508 | NM_023077 | COA7 | 0.348250389 | 0.017777135 | 0.296237697 | 1 | 0.045083345 | 0.790851318 | 0.9172898 | 1 |
| 322_46 | A_23_P4144 | NM_025233 | COASY | 0.069307253 | 0.632012681 | 0.901332063 | 1 | -0.277856386 | 0.061902733 | 0.2630223 | 1 |
| 133_93 | A_33_P3238215 | NM_001278458 | COBLL1 | -0.107561642 | 0.393653679 | 0.799115001 | 1 | 0.129508534 | 0.288975154 | 0.5801684 | 1 |
| 271_5 | A_33_P3372580 | NM_018714 | COG1 | -0.314622572 | 0.032817704 | 0.350972091 | 1 | 0.068824992 | 0.573741331 | 0.803395 | 1 |
| 230_134 | A_23_P160809 | NM_007357 | COG2 | 0.253846447 | 0.080287776 | 0.468656078 | 1 | -0.312004418 | 0.040000191 | 0.2061069 | 1 |
| 101_24 | A_23_P134614 | NM_006348 | COG5 | 0.035195432 | 0.788733537 | 0.94877096 | 1 | 0.198939517 | 0.119123334 | 0.3746589 | 1 |
| 344_149 | A_32_P92399 | NM_032382 | COG8 | 0.335897699 | 0.037246228 | 0.371171976 | 1 | -0.443885494 | 0.006973803 | 0.0744159 | 1 |
| 286_75 | A_33_P3315410 | NM_004645 | COIL | -0.025263484 | 0.860728251 | 0.967090315 | 1 | 0.209745353 | 0.100796805 | 0.3447876 | 1 |
| 324_117 | A_33_P3210647 | NM_001856 | COL16A1 | -0.133671885 | 0.311557111 | 0.743773338 | 1 | 0.088745277 | 0.4377459 | 0.7090907 | 1 |
| 233_66 | A_23_P160318 | NM_001856 | COL16A1 | 0.020856702 | 0.874451708 | 0.970677357 | 1 | 0.407009999 | 0.02707729 | 0.1652405 | 1 |
| 315_136 | A_23_P211212 | NM_030582 | COL18A1 | -0.648068278 | 0.001566765 | 0.127946984 | 1 | -0.689358348 | 0.001106322 | 0.0237067 | 1 |
| 109_7 | A_24_P363896 | NM_032888 | COL27A1 | -0.049539254 | 0.732462191 | 0.937415492 | 1 | -0.083536964 | 0.44406392 | 0.7134548 | 1 |
| 237_147 | A_23_P205031 | NM_001846 | COL4A2 | 0.192599756 | 0.275542561 | 0.716856302 | 1 | -0.09211407 | 0.462706609 | 0.7253899 | 1 |
| 116_132 | A_33_P3271445 | NM_001130105 | COL4A3BP | -0.061494533 | 0.63624659 | 0.902930563 | 1 | -0.00759686 | 0.944651051 | 0.980365 | 1 |
| 321_162 | A_24_P29277 | NM_001130105 | COL4A3BP | -0.048958234 | 0.713907564 | 0.932235175 | 1 | 0.0204406 | 0.854080459 | 0.947135 | 1 |
| 84_98 | A_32_P32254 | NM_001848 | COL6A1 | -0.175128028 | 0.228870012 | 0.675109425 | 1 | -0.388240811 | 0.00493396 | 0.0596931 | 1 |
| 142_72 | A_23_P389525 | NM_152516 | COMMD1 | 0.064605348 | 0.679204665 | 0.919351731 | 1 | 0.063120611 | 0.628771488 | 0.834233 | 1 |
| 26_99 | A_23_P138514 | NM_012071 | COMMD3 | -0.142348345 | 0.311465038 | 0.743773338 | 1 | -0.06541185 | 0.579722507 | 0.806848 | 1 |
| 132_147 | A_24_P331904 | NM_017828 | COMMD4 | 0.084666356 | 0.486970862 | 0.845656313 | 1 | 0.207361842 | 0.077312766 | 0.2988099 | 1 |
| 195_143 | A_24_P93371 | NM_017828 | COMMD4 | 0.397492751 | 0.010504024 | 0.253442612 | 1 | 0.061500246 | 0.608956636 | 0.8215262 | 1 |
| 192_25 | A_23_P20255 | NM_014066 | COMMD5 | 0.057425347 | 0.682463829 | 0.920314282 | 1 | -0.095500199 | 0.37987353 | 0.6619829 | 1 |
| 160_158 | A_32_P114215 | NM_203497 | COMMD6 | -0.107826985 | 0.433980878 | 0.823995489 | 1 | 0.111834157 | 0.369409391 | 0.6534271 | 1 |
| 279_89 | A_23_P44257 | NM_017845 | COMMD8 | 0.06564549 | 0.630580349 | 0.901004617 | 1 | 0.144288434 | 0.235558919 | 0.5233849 | 1 |
| 121_141 | A_33_P3257222 | NM_144589 | COMTD1 | -0.153513441 | 0.25572632 | 0.699039445 | 1 | 0.222913609 | 0.082287774 | 0.3088408 | 1 |
| 178_87 | A_32_P20454 | NM_001098398 | COPA | -0.0501161 | 0.681980179 | 0.920314282 | 1 | 0.029968728 | 0.797543316 | 0.9204689 | 1 |
| 260_157 | A_33_P3287263 | NM_016451 | COPB1 | 0.031393904 | 0.800179045 | 0.951838797 | 1 | -0.247204963 | 0.076267425 | 0.2962306 | 1 |
| 343_28 | A_23_P91891 | NM_004766 | COPB2 | -0.045016808 | 0.786606431 | 0.94877096 | 1 | -0.065231372 | 0.634575753 | 0.8383735 | 1 |
| 327_48 | A_23_P61280 | NM_012133 | COPG2 | -0.151086273 | 0.281309277 | 0.722214883 | 1 | -0.011969857 | 0.933343803 | 0.9762222 | 1 |
| 113_142 | A_23_P152858 | NM_018405 | COPRS | 0.08908851 | 0.505219758 | 0.85467065 | 1 | 0.015128963 | 0.895777655 | 0.9633162 | 1 |
| 351_146 | A_23_P26021 | NM_004236 | COPS2 | 0.211366925 | 0.114770787 | 0.535890277 | 1 | -0.106290333 | 0.443307942 | 0.7128802 | 1 |
| 63_113 | A_23_P89199 | NM_003653 | COPS3 | 0.186494909 | 0.199952543 | 0.646494862 | 1 | -0.453394305 | 0.002545156 | 0.0395071 | 1 |
| 183_104 | A_23_P43779 | NM_016129 | COPS4 | 0.401963741 | 0.023623662 | 0.316794251 | 1 | -0.236219576 | 0.153468673 | 0.4221273 | 1 |
| 43_143 | A_23_P71419 | NM_006837 | COPS5 | 6.46468E-05 | 0.999677687 | 0.999983039 | 1 | -0.375724201 | 0.034980272 | 0.1921821 | 1 |
| 18_88 | A_23_P8432 | NM_006833 | COPS6 | 0.258065568 | 0.104007607 | 0.516834727 | 1 | -0.252068711 | 0.088704464 | 0.3211781 | 1 |
| 114_106 | A_33_P3289145 | NM_198189 | COPS8 | 0.159254027 | 0.215992823 | 0.662310915 | 1 | 0.241360465 | 0.057238228 | 0.25244 | 1 |
| 312_137 | A_24_P915371 | NM_001163424 | COPS9 | 0.049298478 | 0.713012975 | 0.931866116 | 1 | 0.645154292 | 0.0001759 | 0.0072203 | 1 |
| 291_65 | A_24_P389959 | NM_016057 | COPZ1 | 0.17307948 | 0.360621583 | 0.776988349 | 1 | 0.655409978 | 0.000359498 | 0.0114217 | 1 |
| 135_57 | A_23_P354798 | NM_144576 | COQ10A | 0.131808022 | 0.361838189 | 0.77846455 | 1 | 0.009870216 | 0.936029217 | 0.9771228 | 1 |
| 76_95 | A_33_P3221843 | ENST00000254759 | COQ3 | -0.044217742 | 0.757630514 | 0.942483836 | 1 | -0.042751668 | 0.72547937 | 0.8880569 | 1 |
| 292_7 | A_33_P3243128 | NM_032314 | COQ5 | 0.027727278 | 0.828431717 | 0.959690637 | 1 | 0.051822233 | 0.663471966 | 0.8565307 | 1 |
| 332_131 | A_23_P105625 | NM_032314 | COQ5 | 0.166505309 | 0.208568684 | 0.655712085 | 1 | -0.024183534 | 0.856679118 | 0.9486138 | 1 |
| 185_80 | A_24_P398972 | NM_016138 | COQ7 | -0.06551013 | 0.589650549 | 0.887046702 | 1 | -0.107392433 | 0.369560297 | 0.6534271 | 1 |
| 235_19 | A_22_P00025286 | NM_020312 | COQ9 | 0.080793789 | 0.581217328 | 0.883808109 | 1 | 0.043295235 | 0.694690214 | 0.8728201 | 1 |
| 222_157 | A_23_P14928 | NM_020312 | COQ9 | 0.369950746 | 0.021486216 | 0.308342332 | 1 | -0.157664159 | 0.244135 | 0.5318463 | 1 |
| 253_7 | A_33_P3343295 | NM_024535 | CORO7 | -0.589782026 | 0.004678275 | 0.195110893 | 1 | 0.062055793 | 0.648921924 | 0.8473939 | 1 |
| 113_140 | A_24_P416131 | NM_021149 | COTL1 | 0.758878289 | 0.124900002 | 0.550681078 | 1 | 0.128611154 | 0.643585211 | 0.8448198 | 1 |
| 204_20 | A_23_P301925 | ENST00000361624 | COX1 | -0.253239832 | 0.455641879 | 0.83317763 | 1 | -0.443946573 | 0.012459474 | 0.104654 | 1 |
| 93_107 | A_21_P0000034 | NM_004375 | COX11 | 0.030515469 | 0.833723704 | 0.961426617 | 1 | -0.102142619 | 0.397398451 | 0.6759879 | 1 |
| 233_150 | A_23_P152666 | NM_004375 | COX11 | 0.166947143 | 0.198021194 | 0.644653712 | 1 | -0.153661648 | 0.185883948 | 0.4658282 | 1 |
| 97_137 | A_23_P151280 | NM_032901 | COX14 | -0.10889662 | 0.404876441 | 0.806612891 | 1 | -0.143983529 | 0.266590707 | 0.5572023 | 1 |
| 201_158 | A_23_P53957 | NM_016468 | COX16 | 0.021640219 | 0.883926853 | 0.972820007 | 1 | 0.00246952 | 0.984911583 | 0.9949293 | 1 |
| 7_34 | A_23_P65157 | NM_005694 | COX17 | -0.311075914 | 0.044393134 | 0.392255259 | 1 | 0.180863857 | 0.155728765 | 0.4257644 | 1 |
| 56_2 | A_32_P9468 | NM_001031617 | COX19 | 0.048612281 | 0.737373089 | 0.939807384 | 1 | -0.062710819 | 0.58144209 | 0.8080221 | 1 |
| 142_141 | A_23_P402751 | ENST00000361739 | COX2; COX1 | -0.436203604 | 0.075777529 | 0.462072146 | 1 | -0.685108761 | 0.000335458 | 0.0109359 | 1 |
| 13_138 | A_33_P3233947 | NM_198076 | COX20 | 0.097811509 | 0.506020992 | 0.854870853 | 1 | 0.23748582 | 0.231692076 | 0.519713 | 1 |
| 163_135 | A_33_P3336696 | ENST00000361335 | COX3; ND3; ND4L | -0.465875149 | 0.09696019 | 0.500440449 | 1 | -0.56616015 | 0.007429307 | 0.077368 | 1 |
| 322_164 | A_23_P141032 | NM_001861 | COX4I1 | 0.434367554 | 0.028250267 | 0.333724089 | 1 | -0.029336631 | 0.798306064 | 0.9207823 | 1 |
| 238_65 | A_32_P98502 | NM_004255 | COX5A | 0.323230484 | 0.07194247 | 0.456574115 | 1 | -0.077751347 | 0.511064466 | 0.7601476 | 1 |
| 283_104 | A_19_P00808453 | NM_004255 | COX5A | 0.394344517 | 0.014167032 | 0.274111634 | 1 | -0.059111604 | 0.580937913 | 0.8079397 | 1 |
| 372_56 | A_33_P3274851 | NM_001862 | COX5B | -0.016422001 | 0.895643159 | 0.975468897 | 1 | 0.264462169 | 0.087005242 | 0.3180201 | 1 |
| 225_145 | A_32_P168247 | NM_004373 | COX6A1 | -0.156253487 | 0.233590569 | 0.679769612 | 1 | 0.034708183 | 0.768409267 | 0.9077701 | 1 |
| 115_138 | A_23_P108244 | NM_001863 | COX6B1 | -0.015228718 | 0.933282068 | 0.986314058 | 1 | 0.060023793 | 0.657104937 | 0.8531062 | 1 |
| 313_146 | A_33_P3378284 | NM_144613 | COX6B2 | 0.140611586 | 0.391255571 | 0.79716932 | 1 | 0.599186265 | 0.046615574 | 0.2235758 | 1 |
| 172_28 | A_23_P8900 | NM_004374 | COX6C | -0.253468917 | 0.089173162 | 0.486413374 | 1 | 0.286188992 | 0.098226559 | 0.3406581 | 1 |
| 57_101 | A_23_P81690 | NM_001865 | COX7A2 | 0.027970862 | 0.835963605 | 0.961943507 | 1 | 0.110850527 | 0.448228433 | 0.7166534 | 1 |
| 307_23 | A_23_P210348 | NM_004718 | COX7A2L | 0.011154834 | 0.934791605 | 0.986454551 | 1 | 0.005280093 | 0.977444305 | 0.9917899 | 1 |
| 266_63 | A_23_P159650 | NM_001866 | COX7B | -0.058559316 | 0.670447028 | 0.916686207 | 1 | 0.009330825 | 0.932756763 | 0.975699 | 1 |
| 120_6 | A_23_P110811 | NM_001867 | COX7C | -0.45575614 | 0.007846757 | 0.233143633 | 1 | 0.427630574 | 0.068498246 | 0.2783027 | 1 |
| 322_6 | A_23_P110811 | NM_001867 | COX7C | -0.442748746 | 0.007593446 | 0.230091297 | 1 | 0.187504267 | 0.171984041 | 0.450018 | 1 |
| 253_21 | A_23_P110811 | NM_001867 | COX7C | -0.404543656 | 0.009166089 | 0.240131822 | 1 | 0.090340285 | 0.514444513 | 0.7618114 | 1 |
| 80_12 | A_23_P110811 | NM_001867 | COX7C | -0.382123679 | 0.024475245 | 0.318700868 | 1 | 0.144102764 | 0.302214874 | 0.5927818 | 1 |
| 67_145 | A_23_P110811 | NM_001867 | COX7C | -0.276748992 | 0.048201101 | 0.401357357 | 1 | 0.058554828 | 0.582850872 | 0.8088721 | 1 |
| 114_71 | A_23_P110811 | NM_001867 | COX7C | -0.203908593 | 0.200892065 | 0.647608464 | 1 | 0.102896691 | 0.456120151 | 0.7207865 | 1 |
| 226_79 | A_23_P110811 | NM_001867 | COX7C | -0.193926663 | 0.200570508 | 0.647493287 | 1 | 0.14350063 | 0.371781178 | 0.6550662 | 1 |
| 219_57 | A_23_P110811 | NM_001867 | COX7C | -0.111367058 | 0.491720824 | 0.848221902 | 1 | 0.088896018 | 0.489551457 | 0.7463719 | 1 |
| 8_143 | A_23_P110811 | NM_001867 | COX7C | -0.097508901 | 0.441308831 | 0.82834854 | 1 | 0.107659205 | 0.399254245 | 0.677591 | 1 |
| 196_106 | A_23_P110811 | NM_001867 | COX7C | 0.036118369 | 0.789006791 | 0.94877096 | 1 | 0.071903508 | 0.697497591 | 0.8739701 | 1 |
| 59_79 | A_23_P52639 | NM_004074 | COX8A | -0.02082772 | 0.881967245 | 0.972820007 | 1 | 0.029323494 | 0.834516176 | 0.9375417 | 1 |
| 134_99 | A_22_P00011339 | NM_004074 | COX8A | 0.182427806 | 0.251917512 | 0.695888548 | 1 | -0.179023919 | 0.12744199 | 0.388439 | 1 |
| 234_127 | A_33_P3262205 | NM_003915 | CPNE1 | 0.100891865 | 0.508721923 | 0.856078091 | 1 | -0.28489783 | 0.167789543 | 0.4448345 | 1 |
| 160_118 | A_33_P3290888 | NM_003915 | CPNE1 | 0.537217366 | 0.063014765 | 0.435667346 | 1 | 0.022003171 | 0.905636684 | 0.9670246 | 1 |
| 252_38 | A_33_P3262191 | NM_014427 | CPNE7 | -0.121745869 | 0.351011991 | 0.771478158 | 1 | 0.628684722 | 0.001364873 | 0.0266044 | 1 |
| 30_23 | A_33_P3301025 | NM_000097 | CPOX | -0.086377134 | 0.628177472 | 0.900268731 | 1 | 0.075177994 | 0.568868933 | 0.7999586 | 1 |
| 157_83 | A_23_P28688 | NM_016207 | CPSF3 | 0.156776428 | 0.257464054 | 0.699370343 | 1 | -0.180212844 | 0.20123583 | 0.4835834 | 1 |
| 261_29 | A_33_P3270802 | NM_001256456 | CPSF3L | -0.371186722 | 0.029751162 | 0.339352673 | 1 | 0.089486065 | 0.425994511 | 0.6999026 | 1 |
| 234_4 | A_23_P42997 | NM_006693 | CPSF4 | 0.159427491 | 0.258285818 | 0.700376544 | 1 | -0.181034564 | 0.144100675 | 0.4117633 | 1 |
| 128_61 | A_24_P121271 | NM_007007 | CPSF6 | 0.131121696 | 0.307790899 | 0.740336569 | 1 | -0.224668865 | 0.132965514 | 0.3955205 | 1 |
| 55_112 | A_33_P3309039 | NM_024811 | CPSF7 | -0.0076973 | 0.96267773 | 0.993286853 | 1 | 0.058186577 | 0.696445877 | 0.8734794 | 1 |
| 70_99 | A_33_P3266396 | NM_001029885 | CPTP | -0.187956255 | 0.185814872 | 0.629215338 | 1 | -0.139410723 | 0.2483795 | 0.5355889 | 1 |
| 163_77 | A_33_P3343220 | NM_001029885 | CPTP | 0.05430819 | 0.725687635 | 0.936348816 | 1 | -0.088673517 | 0.613894664 | 0.8242179 | 1 |
| 117_36 | A_23_P432545 | NM_173584 | CRACR2B | -0.05330154 | 0.69874401 | 0.925106933 | 1 | 0.152940928 | 0.187950349 | 0.4686051 | 1 |
| 6_117 | A_32_P29806 | NM_003805 | CRADD | -0.999571013 | 0.000222397 | 0.054706987 | 1 | -0.081283441 | 0.525926129 | 0.770081 | 1 |
| 200_91 | A_33_P3280531 | NM_001257363 | CRAT | -0.586915373 | 0.004428698 | 0.192941552 | 1 | -0.352480861 | 0.077177227 | 0.2986113 | 1 |
| 204_74 | A_22_P00013055 | ENST00000579474 | CRAT40 | 0.130364552 | 0.352485606 | 0.772383489 | 1 | 0.074795624 | 0.695180817 | 0.8731685 | 1 |
| 111_10 | A_33_P3398401 | NM_016302 | CRBN | -0.062734433 | 0.690077198 | 0.923073947 | 1 | 0.299452929 | 0.027763097 | 0.1673964 | 1 |
| 356_66 | A_23_P150407 | NM_052854 | CREB3L1 | 0.00937718 | 0.942128939 | 0.988968248 | 1 | 0.190027748 | 0.132016343 | 0.3937385 | 1 |
| 75_17 | A_33_P3211739 | NM_194071 | CREB3L2 | -0.098242985 | 0.455101182 | 0.832937561 | 1 | 0.217464883 | 0.143599292 | 0.4109571 | 1 |
| 83_1 | A_23_P63232 | NM_130898 | CREB3L4 | -0.255140934 | 0.118993126 | 0.54143244 | 1 | 0.428130678 | 0.005417561 | 0.0635432 | 1 |
| 376_98 | A_33_P3240229 | NM_004380 | CREBBP | -0.181749195 | 0.237457414 | 0.683441761 | 1 | -0.093728824 | 0.409155708 | 0.685253 | 1 |
| 323_85 | A_33_P3231297 | NM_003851 | CREG1 | -0.089969122 | 0.489060896 | 0.846298319 | 1 | 0.766242015 | 0.000370687 | 0.0116131 | 1 |
| 31_35 | A_23_P155376 | NM_015513 | CRELD1 | -0.04288242 | 0.77132693 | 0.945351992 | 1 | 0.074051366 | 0.598711089 | 0.8174528 | 1 |
| 130_161 | A_23_P33465 | NM_024324 | CRELD2 | 0.21725043 | 0.143469276 | 0.577957249 | 1 | -0.310220708 | 0.035051857 | 0.1922632 | 1 |
| 122_1 | A_33_P3251989 | NM_014171 | CRIPT | -0.086264461 | 0.517497877 | 0.860401138 | 1 | 0.170379129 | 0.246853876 | 0.5341018 | 1 |
| 270_2 | A_33_P3502315 | NM_019095 | CRLS1 | -0.053765041 | 0.699944509 | 0.925289905 | 1 | 0.109279435 | 0.349066934 | 0.6342423 | 1 |
| 370_107 | A_22_P00008271 | NR_110454 | CRNDE | -0.003365061 | 0.981060746 | 0.995429381 | 1 | -0.236156071 | 0.044963852 | 0.2193011 | 1 |
| 7_17 | A_19_P00322533 | NR_110453 | CRNDE | 0.08594492 | 0.479586518 | 0.84183328 | 1 | -0.1363214 | 0.224093515 | 0.5124806 | 1 |
| 102_119 | A_32_P104063 | NR_110453 | CRNDE | 0.133678361 | 0.29783355 | 0.734102656 | 1 | -0.170896964 | 0.17639493 | 0.4549252 | 1 |
| 187_97 | A_22_P00008272 | NR_110453 | CRNDE | 0.236761507 | 0.134135939 | 0.56431697 | 1 | -0.211581998 | 0.146574114 | 0.4149625 | 1 |
| 65_56 | A_33_P3663705 | NM_014675 | CROCC | -0.409573418 | 0.012012039 | 0.2646124 | 1 | 0.421978876 | 0.010777528 | 0.0958055 | 1 |
| 23_100 | A_23_P126486 | NR_026752 | CROCCP2 | -0.108173352 | 0.485468656 | 0.844371668 | 1 | 0.145442121 | 0.425697529 | 0.6996668 | 1 |
| 233_12 | A_23_P12173 | NM_181715 | CRTC2 | -0.49318102 | 0.009826007 | 0.244710004 | 1 | 0.115542094 | 0.30289504 | 0.593208 | 1 |
| 333_12 | A_23_P12173 | NM_181715 | CRTC2 | -0.486762336 | 0.010619114 | 0.255151975 | 1 | 0.135577551 | 0.256616936 | 0.5457612 | 1 |
| 119_8 | A_23_P12173 | NM_181715 | CRTC2 | -0.467015587 | 0.010494782 | 0.253442612 | 1 | 0.11228535 | 0.311910016 | 0.600591 | 1 |
| 76_34 | A_23_P12173 | NM_181715 | CRTC2 | -0.45015588 | 0.018112716 | 0.29646832 | 1 | 0.10867751 | 0.366549017 | 0.6506815 | 1 |
| 205_22 | A_23_P12173 | NM_181715 | CRTC2 | -0.382550649 | 0.026452512 | 0.327817397 | 1 | 0.124840709 | 0.320944669 | 0.6074831 | 1 |
| 347_80 | A_23_P12173 | NM_181715 | CRTC2 | -0.377505089 | 0.024122649 | 0.318034123 | 1 | 0.089212006 | 0.469214784 | 0.7304164 | 1 |
| 178_45 | A_23_P12173 | NM_181715 | CRTC2 | -0.364224646 | 0.021995534 | 0.310846393 | 1 | 0.112000147 | 0.319802265 | 0.606239 | 1 |
| 45_152 | A_23_P12173 | NM_181715 | CRTC2 | -0.296124448 | 0.057205519 | 0.423633819 | 1 | -0.017947187 | 0.881081745 | 0.9588224 | 1 |
| 277_96 | A_23_P12173 | NM_181715 | CRTC2 | -0.272216593 | 0.047547502 | 0.399787213 | 1 | 0.137166051 | 0.214012182 | 0.4998951 | 1 |
| 25_133 | A_23_P12173 | NM_181715 | CRTC2 | -0.269668231 | 0.119464738 | 0.542056525 | 1 | -0.00455801 | 0.973745032 | 0.9906597 | 1 |
| 144_91 | A_23_P127394 | NM_021117 | CRY2 | -0.024801907 | 0.853482594 | 0.965079421 | 1 | 0.050058087 | 0.729772473 | 0.8901815 | 1 |
| 53_92 | A_33_P3358158 | NR_033733 | CRYBB2P1 | -0.1970983 | 0.160168148 | 0.598752215 | 1 | 0.172058121 | 0.295455256 | 0.5861835 | 1 |
| 169_144 | A_33_P3323559 | NM_000394 | CRYAA | 0.151659597 | 0.560090382 | 0.87771117 | 1 | -0.021331661 | 0.932598344 | 0.975699 | 1 |
| 50_68 | A_33_P3215078 | NM_004077 | CS | -0.042068189 | 0.800846659 | 0.952164507 | 1 | -0.127722659 | 0.316644356 | 0.6036347 | 1 |
| 54_41 | A_21_P0011678 | NM_004077 | CS | 0.113293994 | 0.457142301 | 0.83358833 | 1 | -0.133008616 | 0.286026102 | 0.5772057 | 1 |
| 350_59 | A_32_P73821 | NM_001007553 | CSDE1 | -0.046047497 | 0.768376427 | 0.945351992 | 1 | 0.246000568 | 0.158514916 | 0.4307362 | 1 |
| 131_10 | A_23_P17393 | NM_001316 | CSE1L | -0.000237837 | 0.998410368 | 0.999808735 | 1 | -0.407776896 | 0.016411414 | 0.1230528 | 1 |
| 30_17 | A_33_P3284029 | NM_001316 | CSE1L | 0.042905662 | 0.748269645 | 0.941597731 | 1 | -0.502562801 | 0.009160106 | 0.0870133 | 1 |
| 327_112 | A_33_P3265185 | ENST00000403904 | CSNK1E; LOC400927-CSNK1E | -0.172640067 | 0.231525111 | 0.677668275 | 1 | 0.388480674 | 0.018418619 | 0.1319635 | 1 |
| 328_95 | A_33_P3259017 | NM_001319 | CSNK1G2 | -0.088879572 | 0.638283673 | 0.904241389 | 1 | 0.006911023 | 0.959139874 | 0.9858263 | 1 |
| 291_113 | A_23_P502575 | NM_177559 | CSNK2A1 | -0.004731654 | 0.974398793 | 0.994877235 | 1 | 0.003921031 | 0.972394371 | 0.9903576 | 1 |
| 97_162 | A_21_P0014329 | ENST00000563307 | CSNK2A2 | 0.276096227 | 0.220205627 | 0.666636417 | 1 | -0.369829092 | 0.039836129 | 0.2058048 | 1 |
| 145_42 | A_33_P3318027 | NM_001320 | CSNK2B | 0.319794486 | 0.056781138 | 0.423246544 | 1 | -0.51131282 | 0.099386359 | 0.3430934 | 1 |
| 231_100 | A_24_P97931 | NM_001320 | CSNK2B | 0.507442411 | 0.029666246 | 0.339352673 | 1 | -0.420167545 | 0.123464222 | 0.3816938 | 1 |
| 181_129 | A_23_P44724 | NM_001321 | CSRP2 | -0.200787919 | 0.288664461 | 0.727382494 | 1 | -0.720031725 | 0.000930656 | 0.0214194 | 1 |
| 331_104 | A_33_P3228266 | NM_000099 | CST3 | 0.30501899 | 0.058162991 | 0.425190813 | 1 | -0.337176855 | 0.075896845 | 0.2954696 | 1 |
| 123_106 | A_23_P170453 | NM_001900 | CST5 | 0.203180108 | 0.208712429 | 0.655712085 | 1 | -0.13881933 | 0.266683436 | 0.5572023 | 1 |
| 35_61 | A_23_P154894 | NM_000100 | CSTB | -0.157055948 | 0.471727443 | 0.838394791 | 1 | -0.263360887 | 0.116873772 | 0.3709903 | 1 |
| 101_20 | A_23_P362824 | NM_001324 | CSTF1 | -0.173517426 | 0.192809185 | 0.637568267 | 1 | 0.142495481 | 0.27059266 | 0.5610981 | 1 |
| 294_43 | A_23_P148372 | NM_001325 | CSTF2 | 0.135300519 | 0.280913832 | 0.721960401 | 1 | -0.037699234 | 0.836646985 | 0.9384016 | 1 |
| 64_67 | A_24_P941051 | NM_015235 | CSTF2T | -0.037078094 | 0.789341447 | 0.94877096 | 1 | -0.293506308 | 0.031017118 | 0.1787029 | 1 |
| 233_149 | A_23_P98310 | NM_001326 | CSTF3 | 0.248121796 | 0.069000159 | 0.452344164 | 1 | -0.078730382 | 0.509351785 | 0.7595703 | 1 |
| 176_136 | A_32_P207124 | NM_173571 | CT47A11 | 0.063956774 | 0.633375766 | 0.901716027 | 1 | 0.008982646 | 0.938391387 | 0.9784988 | 1 |
| 75_72 | A_24_P332623 | NR_027466 | CTAGE11P | -0.010285932 | 0.93807702 | 0.987634793 | 1 | 0.247203853 | 0.10413778 | 0.3506699 | 1 |
| 51_100 | A_24_P169773 | NM_198495 | CTAGE4 | -0.003093557 | 0.982691233 | 0.995672988 | 1 | 0.222305014 | 0.093388439 | 0.3316513 | 1 |
| 334_106 | A_23_P63897 | NM_022802 | CTBP2 | -0.13447401 | 0.287550078 | 0.72693776 | 1 | 0.033972747 | 0.781108114 | 0.9133558 | 1 |
| 343_21 | A_22_P00016776 | NR_109909 | CTC-338M12.4 | 0.112257208 | 0.469389615 | 0.836960042 | 1 | -0.009610904 | 0.944747145 | 0.980365 | 1 |
| 27_93 | A_21_P0014494 | NR_109909 | CTC-338M12.4 | 0.132431947 | 0.412001074 | 0.812009855 | 1 | -0.077951115 | 0.578105358 | 0.8054289 | 1 |
| 368_71 | A_33_P3384825 | NM_015343 | CTDNEP1 | 0.102134239 | 0.445368387 | 0.829565274 | 1 | -0.066254482 | 0.710472711 | 0.8795214 | 1 |
| 283_107 | A_33_P3401841 | ENST00000613122 | CTDP1 | -0.008590472 | 0.942527801 | 0.988968248 | 1 | -0.012000745 | 0.923163223 | 0.9723203 | 1 |
| 286_131 | A_23_P28263 | NM_021198 | CTDSP1 | -0.197755415 | 0.181600549 | 0.622415463 | 1 | 0.368806796 | 0.008871526 | 0.0860156 | 1 |
| 194_100 | A_23_P81880 | NM_005730 | CTDSP2 | 0.240111054 | 0.07557573 | 0.461954037 | 1 | -0.220917239 | 0.256648974 | 0.5457612 | 1 |
| 347_14 | A_24_P251534 | NM_001008392 | CTDSPL | -0.187333197 | 0.203832594 | 0.650388028 | 1 | 0.293568428 | 0.078749646 | 0.3023184 | 1 |
| 170_68 | A_32_P76060 | NM_016396 | CTDSPL2 | 0.060486483 | 0.644773257 | 0.906600677 | 1 | 0.123004917 | 0.313395921 | 0.6015453 | 1 |
| 169_143 | A_23_P58647 | NM_001903 | CTNNA1 | 0.122476636 | 0.549358857 | 0.873756475 | 1 | -0.238413846 | 0.202043799 | 0.4847576 | 1 |
| 163_67 | A_33_P3260605 | NM_003798 | CTNNAL1 | -0.071707765 | 0.690501923 | 0.923073947 | 1 | -0.207994118 | 0.268622238 | 0.5590663 | 1 |
| 372_99 | A_23_P157795 | NM_003798 | CTNNAL1 | -0.028483241 | 0.839071643 | 0.962281738 | 1 | -0.256782908 | 0.062209762 | 0.2640276 | 1 |
| 272_21 | A_23_P29495 | NM_001904 | CTNNB1 | 0.090625424 | 0.591599027 | 0.888218675 | 1 | -0.116898363 | 0.510075798 | 0.7600454 | 1 |
| 156_15 | A_23_P29495 | NM_001904 | CTNNB1 | 0.126510501 | 0.430521475 | 0.821617604 | 1 | -0.176884334 | 0.295161605 | 0.5861021 | 1 |
| 320_28 | A_23_P29495 | NM_001904 | CTNNB1 | 0.13697157 | 0.410918166 | 0.81197163 | 1 | -0.06630324 | 0.728246552 | 0.8891217 | 1 |
| 84_53 | A_23_P29495 | NM_001904 | CTNNB1 | 0.244122167 | 0.213961539 | 0.660704553 | 1 | -0.189117456 | 0.339144134 | 0.6259021 | 1 |
| 245_92 | A_23_P29495 | NM_001904 | CTNNB1 | 0.249213116 | 0.29485753 | 0.732603053 | 1 | -0.204101766 | 0.347541787 | 0.6333121 | 1 |
| 9_112 | A_23_P29495 | NM_001904 | CTNNB1 | 0.328649164 | 0.05403891 | 0.417174905 | 1 | -0.184104973 | 0.290711953 | 0.582269 | 1 |
| 170_155 | A_23_P29495 | NM_001904 | CTNNB1 | 0.329442641 | 0.110920491 | 0.529168197 | 1 | -0.162050754 | 0.388388136 | 0.669251 | 1 |
| 186_158 | A_23_P29495 | NM_001904 | CTNNB1 | 0.344954958 | 0.072913057 | 0.459040517 | 1 | -0.211522766 | 0.272431984 | 0.5630361 | 1 |
| 102_113 | A_23_P29495 | NM_001904 | CTNNB1 | 0.372255361 | 0.045067186 | 0.394119497 | 1 | -0.243366796 | 0.133642371 | 0.3969041 | 1 |
| 145_105 | A_23_P29495 | NM_001904 | CTNNB1 | 0.43277083 | 0.037864012 | 0.371467292 | 1 | -0.224621972 | 0.242719653 | 0.5304835 | 1 |
| 247_157 | A_23_P23584 | NM_020248 | CTNNBIP1 | -0.237628492 | 0.113756322 | 0.533813374 | 1 | -0.769936395 | 0.000164323 | 0.0069741 | 1 |
| 254_80 | A_33_P3272361 | NM_001281495 | CTNNBL1 | -0.038275525 | 0.758440199 | 0.942483836 | 1 | 0.134653034 | 0.386327751 | 0.6670292 | 1 |
| 264_18 | A_23_P251316 | NM_001331 | CTNND1 | -0.11058955 | 0.424569495 | 0.819650834 | 1 | -0.196006194 | 0.130117204 | 0.391291 | 1 |
| 139_152 | A_33_P3322430 | A_33_P3322430 | CTNS | -0.027829341 | 0.844387909 | 0.964376494 | 1 | 0.191081243 | 0.163535575 | 0.4386159 | 1 |
| 227_26 | A_33_P3235217 | NM_001905 | CTPS1 | -0.047354174 | 0.790400106 | 0.94877096 | 1 | -0.078206957 | 0.495929115 | 0.750974 | 1 |
| 286_135 | A_23_P21706 | NM_001905 | CTPS1 | 0.281467285 | 0.065581312 | 0.443209074 | 1 | -0.121644353 | 0.364469694 | 0.6486061 | 1 |
| 340_128 | A_33_P3272593 | NM_001144002 | CTPS2 | -0.189626873 | 0.177043898 | 0.616374573 | 1 | -0.794124264 | 0.000142907 | 0.0065911 | 1 |
| 69_72 | A_23_P127676 | NM_014633 | CTR9 | 0.04095719 | 0.748749297 | 0.941597731 | 1 | -0.044035625 | 0.68590808 | 0.8685753 | 1 |
| 214_123 | A_32_P86150 | NM_001025200 | CTRB2 | 0.150934942 | 0.275585153 | 0.716856302 | 1 | 0.129038792 | 0.353637631 | 0.6383948 | 1 |
| 346_97 | A_24_P74371 | NM_000308 | CTSA | -0.227695134 | 0.095512349 | 0.498995654 | 1 | 0.172139085 | 0.167477704 | 0.4442504 | 1 |
| 28_126 | A_33_P3287631 | NM_147780 | CTSB | -0.325888458 | 0.056541097 | 0.423246544 | 1 | -0.327032347 | 0.041136604 | 0.2096329 | 1 |
| 183_98 | A_33_P3283480 | NM_148170 | CTSC | 0.368671934 | 0.121344878 | 0.543714677 | 1 | 0.069972138 | 0.671258948 | 0.8616372 | 1 |
| 130_137 | A_33_P3268472 | NM_001114173 | CTSC | 0.698948546 | 0.006021135 | 0.215393444 | 1 | 0.098027577 | 0.506083263 | 0.7577939 | 1 |
| 299_66 | A_23_P14774 | NM_004390 | CTSH | 0.55758656 | 0.012737288 | 0.265846757 | 1 | 0.153580501 | 0.366149141 | 0.65036 | 1 |
| 383_117 | A_33_P3299898 | NR_033407 | CTSLP2 | -0.006070704 | 0.968460675 | 0.994075478 | 1 | -0.053002877 | 0.717797354 | 0.8837108 | 1 |
| 94_33 | A_23_P146456 | NM_001333 | CTSV | -0.624968234 | 0.004292511 | 0.191543479 | 1 | 0.267075586 | 0.067816058 | 0.276852 | 1 |
| 290_118 | A_23_P40240 | NM_001336 | CTSZ | -0.247007966 | 0.10982398 | 0.527106118 | 1 | -0.110088027 | 0.458043229 | 0.7210094 | 1 |
| 182_5 | A_33_P3250398 | ENST00000503833 | CTSZ | -0.236489394 | 0.153691313 | 0.592394196 | 1 | -0.017432479 | 0.895691508 | 0.9633162 | 1 |
| 77_136 | A_33_P3310780 | NM_005231 | CTTN | -0.110691218 | 0.436542976 | 0.826037245 | 1 | -0.354604265 | 0.01823725 | 0.1314092 | 1 |
| 136_32 | A_33_P3395971 | NM_145232 | CTU1 | -0.141872473 | 0.34405849 | 0.767496713 | 1 | 0.070104613 | 0.576648657 | 0.8048695 | 1 |
| 117_161 | A_33_P3395976 | NM_145232 | CTU1 | 0.082939926 | 0.59472456 | 0.889988626 | 1 | 0.280684624 | 0.038920702 | 0.2028615 | 1 |
| 270_74 | A_33_P3320953 | NM_206833 | CTXN1 | -0.14478495 | 0.295913359 | 0.732686289 | 1 | 0.164633586 | 0.252284443 | 0.5402595 | 1 |
| 215_58 | A_23_P127088 | NM_024040 | CUEDC2 | -0.106241746 | 0.572507533 | 0.882516221 | 1 | -0.130254914 | 0.32202598 | 0.6084864 | 1 |
| 244_36 | A_33_P3321150 | NM_003590 | CUL3 | -0.120451114 | 0.370589623 | 0.784043555 | 1 | 0.493594306 | 0.001923469 | 0.0329775 | 1 |
| 286_151 | A_23_P151471 | NM_001008895 | CUL4A | 0.163872324 | 0.29454322 | 0.732574292 | 1 | -0.170708653 | 0.247535946 | 0.535083 | 1 |
| 323_86 | A_33_P3301286 | NM_003478 | CUL5 | 0.010714077 | 0.938099014 | 0.987634793 | 1 | -0.124596037 | 0.27231345 | 0.5630361 | 1 |
| 174_28 | A_23_P311740 | NM_015089 | CUL9 | -0.616955128 | 0.000660551 | 0.090235068 | 1 | 0.617322786 | 0.001498531 | 0.0278739 | 1 |
| 352_105 | A_23_P214678 | NM_015921 | CUTA | -0.134676853 | 0.467579725 | 0.83630364 | 1 | 0.113453006 | 0.309460687 | 0.5976141 | 1 |
| 197_65 | A_23_P253375 | NM_001913 | CUX1 | -0.137507549 | 0.351822299 | 0.771839653 | 1 | -0.223621675 | 0.056508859 | 0.2505416 | 1 |
| 266_11 | A_33_P7363082 | NM_016403 | CWC15 | -0.010901225 | 0.944382186 | 0.989683091 | 1 | -0.10016245 | 0.499767285 | 0.7538737 | 1 |
| 289_22 | A_33_P6607913 | ENST00000542165 | CWC15 | 0.018366657 | 0.897439103 | 0.975758314 | 1 | -0.011960915 | 0.92731653 | 0.9737222 | 1 |
| 308_105 | A_22_P00014361 | NM_016403 | CWC15 | 0.126248792 | 0.353630565 | 0.772780895 | 1 | 0.107942943 | 0.349694987 | 0.63487 | 1 |
| 101_109 | A_23_P92629 | NM_005869 | CWC27 | 0.088227431 | 0.502348124 | 0.853951304 | 1 | -0.265932514 | 0.038496908 | 0.2020377 | 1 |
| 108_155 | A_23_P7144 | NM_001511 | CXCL1 | 0.576894735 | 0.013948467 | 0.271872368 | 1 | -0.449451737 | 0.052720815 | 0.2408412 | 1 |
| 209_83 | A_23_P102000 | NM_001008540 | CXCR4 | -1.357239374 | 4.08027E-05 | 0.03290912 | 0.497059 | 0.352217306 | 0.026783732 | 0.1638046 | 1 |
| 189_84 | A_23_P102000 | NM_001008540 | CXCR4 | -1.354423197 | 3.15473E-05 | 0.03290912 | 0.38431 | 0.324166283 | 0.016182609 | 0.1221508 | 1 |
| 277_45 | A_24_P36745 | NM_144970 | CXorf38 | 0.060041015 | 0.635690844 | 0.902503717 | 1 | -0.361223255 | 0.020269782 | 0.138502 | 1 |
| 170_125 | A_23_P306919 | NM_001013845 | CXorf40B | 0.316612849 | 0.037417771 | 0.371171976 | 1 | 0.187187473 | 0.2125255 | 0.4976628 | 1 |
| 223_90 | A_23_P316239 | NM_001013845 | CXorf40B | 0.342929514 | 0.056771778 | 0.423246544 | 1 | 0.323406987 | 0.135341236 | 0.3993135 | 1 |
| 276_148 | A_23_P96369 | NM_018015 | CXorf57 | 0.429619566 | 0.060982253 | 0.430408929 | 1 | 0.271405435 | 0.067243049 | 0.2762172 | 1 |
| 56_10 | A_23_P399001 | NM_016463 | CXXC5 | -0.332097736 | 0.03381367 | 0.355237115 | 1 | 0.130061383 | 0.292675917 | 0.5846631 | 1 |
| 1_24 | A_23_P218331 | NM_001017916 | CYB561 | -0.491549955 | 0.012482084 | 0.265846757 | 1 | -0.14871857 | 0.313348025 | 0.6015453 | 1 |
| 89_11 | A_33_P3377110 | NM_001134404 | CYB561D1 | -0.257392295 | 0.105591262 | 0.519670274 | 1 | -0.01160602 | 0.934327528 | 0.9765392 | 1 |
| 347_27 | A_23_P121326 | NM_007022 | CYB561D2 | -0.308801229 | 0.031817259 | 0.346378775 | 1 | -0.330402734 | 0.013899947 | 0.1119862 | 1 |
| 367_1 | A_23_P101208 | NM_001914 | CYB5A | -0.021670747 | 0.864995671 | 0.968067563 | 1 | 0.38888948 | 0.025850256 | 0.1608063 | 1 |
| 21_9 | A_23_P101208 | NM_001914 | CYB5A | 0.077784421 | 0.595411744 | 0.890194633 | 1 | 0.269371797 | 0.042294897 | 0.2130863 | 1 |
| 112_33 | A_23_P101208 | NM_001914 | CYB5A | 0.122176893 | 0.352022748 | 0.771839653 | 1 | 0.25637077 | 0.073324889 | 0.2892786 | 1 |
| 328_129 | A_23_P101208 | NM_001914 | CYB5A | 0.185630939 | 0.144768708 | 0.579741093 | 1 | 0.361966612 | 0.012525675 | 0.1048967 | 1 |
| 121_47 | A_23_P101208 | NM_001914 | CYB5A | 0.190652832 | 0.198626798 | 0.645245776 | 1 | 0.275829783 | 0.053401446 | 0.2422776 | 1 |
| 160_162 | A_23_P101208 | NM_001914 | CYB5A | 0.203329986 | 0.119744944 | 0.542056525 | 1 | 0.35364079 | 0.019061881 | 0.1344516 | 1 |
| 142_160 | A_23_P101208 | NM_001914 | CYB5A | 0.203682548 | 0.117560841 | 0.53981859 | 1 | 0.251306267 | 0.066422112 | 0.2738451 | 1 |
| 246_67 | A_23_P101208 | NM_001914 | CYB5A | 0.230626898 | 0.118127193 | 0.540783714 | 1 | 0.400376137 | 0.005099417 | 0.0608844 | 1 |
| 105_108 | A_23_P101208 | NM_001914 | CYB5A | 0.242924696 | 0.072213205 | 0.457123394 | 1 | 0.381393808 | 0.008262651 | 0.0825311 | 1 |
| 196_89 | A_23_P101208 | NM_001914 | CYB5A | 0.246676158 | 0.072997046 | 0.45908622 | 1 | 0.332416497 | 0.022820878 | 0.1495725 | 1 |
| 275_161 | A_33_P3311076 | NM_001190807 | CYB5A | 0.377469183 | 0.034751006 | 0.359292987 | 1 | 0.201679058 | 0.17547906 | 0.4542244 | 1 |
| 70_97 | A_23_P52101 | NM_016243 | CYB5R1 | -0.02277608 | 0.860777257 | 0.967090315 | 1 | -0.135826306 | 0.412425068 | 0.6883135 | 1 |
| 182_35 | A_23_P2181 | NM_016229 | CYB5R2 | -1.101852367 | 0.000805576 | 0.096211064 | 1 | 1.789357169 | 1.86054E-07 | 0.0002616 | 0.00209 |
| 357_160 | A_23_P502224 | NM_007326 | CYB5R3 | -0.235885767 | 0.106296576 | 0.521088485 | 1 | -0.13281571 | 0.301449159 | 0.5924163 | 1 |
| 119_150 | A_33_P3372332 | NM_000101 | CYBA | -0.088269619 | 0.646862146 | 0.907045535 | 1 | -0.025347246 | 0.869961919 | 0.9542093 | 1 |
| 216_86 | A_23_P20980 | NM_001916 | CYC1 | 0.648623874 | 0.007634908 | 0.230091297 | 1 | -0.401004946 | 0.156575028 | 0.4273918 | 1 |
| 116_131 | A_33_P3313401 | NM_018947 | CYCS | 0.224238493 | 0.13569212 | 0.566743973 | 1 | -0.211485148 | 0.136919667 | 0.4016525 | 1 |
| 80_42 | A_24_P376556 | NM_018947 | CYCS | 0.271815752 | 0.133446839 | 0.563483326 | 1 | -0.335614069 | 0.030735328 | 0.1783696 | 1 |
| 182_115 | A_23_P140648 | NM_014608 | CYFIP1 | 0.139409667 | 0.288289416 | 0.727382494 | 1 | 0.008134104 | 0.95102329 | 0.9829826 | 1 |
| 284_34 | A_33_P3285868 | NM_134268 | CYGB | -0.276623379 | 0.040466846 | 0.380082585 | 1 | 0.355133068 | 0.107134233 | 0.3550202 | 1 |
| 56_93 | A_23_P318604 | ENST00000528663 | CYHR1 | -0.12240886 | 0.457875597 | 0.833745766 | 1 | 0.388415231 | 0.017493413 | 0.1280081 | 1 |
| 292_4 | A_33_P3371175 | NM_177538 | CYP20A1 | -0.163023857 | 0.221040902 | 0.667340087 | 1 | 0.360050439 | 0.022378379 | 0.147532 | 1 |
| 182_142 | A_33_P3361422 | NM_000784 | CYP27A1 | -0.219043065 | 0.134665741 | 0.56452101 | 1 | 0.189658882 | 0.178228782 | 0.4568562 | 1 |
| 208_30 | A_23_P103486 | NM_000775 | CYP2J2 | -0.091363479 | 0.487313444 | 0.846010029 | 1 | 1.145591562 | 1.06009E-05 | 0.0015289 | 0.11923 |
| 3_58 | A_23_P202860 | NM_024514 | CYP2R1 | -0.101100081 | 0.46495283 | 0.836223448 | 1 | 0.392256459 | 0.004795759 | 0.0586282 | 1 |
| 87_140 | A_33_P3252612 | NM_017781 | CYP2W1 | -0.029156336 | 0.836030771 | 0.961943507 | 1 | 0.025699031 | 0.841213716 | 0.9408443 | 1 |
| 243_60 | A_23_P259506 | NM_032412 | CYSTM1 | 0.049418426 | 0.743277377 | 0.941128659 | 1 | -0.200632795 | 0.249758842 | 0.5369982 | 1 |
| 349_42 | A_24_P551842 | ENST00000361789 | CYTB | -0.701592094 | 0.058382495 | 0.42530091 | 1 | -0.769643723 | 0.001598383 | 0.0292309 | 1 |
| 250_138 | A_23_P83781 | NM_004762 | CYTH1 | -0.16630866 | 0.221095435 | 0.667340087 | 1 | -0.026404891 | 0.839463685 | 0.9395411 | 1 |
| 317_138 | A_33_P3380405 | ENST00000586299 | CYTH1 | -0.110501313 | 0.449287247 | 0.831477605 | 1 | -0.00495802 | 0.96892221 | 0.9889135 | 1 |
| 351_14 | A_33_P3271051 | NM_004227 | CYTH3 | -0.35295393 | 0.078153885 | 0.465862262 | 1 | 0.147579445 | 0.438135833 | 0.7091284 | 1 |
| 67_129 | A_33_P3220242 | NM_001167575 | CAAP1 | -0.080119274 | 0.569436034 | 0.881307748 | 1 | -0.301659438 | 0.063549755 | 0.2664409 | 1 |
| 93_29 | A_23_P376627 | NM_152783 | D2HGDH | -0.31799882 | 0.030196668 | 0.340535444 | 1 | 0.445280648 | 0.001452386 | 0.0274077 | 1 |
| 260_1 | A_33_P3338928 | NM_001343 | DAB2 | -0.147372791 | 0.345379687 | 0.7684824 | 1 | -0.026124675 | 0.882324102 | 0.9592556 | 1 |
| 91_12 | A_33_P3316786 | NM_080759 | DACH1 | 0.004310004 | 0.972602291 | 0.994758336 | 1 | 1.010630861 | 5.35919E-06 | 0.0013178 | 0.06027 |
| 262_47 | A_23_P106056 | NM_001344 | DAD1 | -0.095266236 | 0.505484406 | 0.85467065 | 1 | -0.06166777 | 0.650243123 | 0.8484089 | 1 |
| 230_32 | A_23_P106056 | NM_001344 | DAD1 | -0.044449504 | 0.772038723 | 0.945508769 | 1 | -0.100645188 | 0.393577584 | 0.6739026 | 1 |
| 286_44 | A_23_P106056 | NM_001344 | DAD1 | -0.030362451 | 0.827278484 | 0.959690637 | 1 | -0.002390488 | 0.982479177 | 0.9938607 | 1 |
| 290_61 | A_23_P106056 | NM_001344 | DAD1 | 0.064140395 | 0.673662685 | 0.918062293 | 1 | 0.031516075 | 0.794422089 | 0.9190412 | 1 |
| 266_61 | A_23_P106056 | NM_001344 | DAD1 | 0.067538794 | 0.669735549 | 0.916686207 | 1 | -0.027821063 | 0.842928463 | 0.9414469 | 1 |
| 73_95 | A_23_P106056 | NM_001344 | DAD1 | 0.070157078 | 0.613165479 | 0.897816866 | 1 | -0.027015127 | 0.835166866 | 0.9377181 | 1 |
| 114_50 | A_23_P106056 | NM_001344 | DAD1 | 0.102717221 | 0.515011773 | 0.85937493 | 1 | -0.00241476 | 0.986016949 | 0.9951304 | 1 |
| 268_86 | A_23_P106056 | NM_001344 | DAD1 | 0.117564458 | 0.469854699 | 0.836960042 | 1 | -0.150235096 | 0.341694509 | 0.6282554 | 1 |
| 208_140 | A_23_P106056 | NM_001344 | DAD1 | 0.177669622 | 0.196017999 | 0.642117721 | 1 | -0.108174342 | 0.351182715 | 0.6362217 | 1 |
| 153_71 | A_23_P106056 | NM_001344 | DAD1 | 0.259046565 | 0.17382096 | 0.613764328 | 1 | -0.078843727 | 0.698952145 | 0.874605 | 1 |
| 323_38 | A_22_P00012866 | NR_024031 | DANCR | 0.039802426 | 0.765763826 | 0.944948837 | 1 | -0.352226408 | 0.067606043 | 0.2767912 | 1 |
| 40_79 | A_23_P63067 | NM_033657 | DAP3 | 0.216231118 | 0.149010732 | 0.585876178 | 1 | 0.041997206 | 0.709674363 | 0.8795214 | 1 |
| 206_95 | A_33_P3224045 | NM_001348 | DAPK3 | 0.059377487 | 0.649032557 | 0.90775139 | 1 | 0.075373018 | 0.6455106 | 0.8457094 | 1 |
| 200_152 | A_33_P3531857 | NM_001349 | DARS | 0.272733115 | 0.14718694 | 0.582720606 | 1 | -0.414237227 | 0.004871364 | 0.0593589 | 1 |
| 85_110 | A_23_P122579 | NM_001350 | DAXX | 0.041933611 | 0.743996399 | 0.941128659 | 1 | -0.173965154 | 0.143332793 | 0.4104032 | 1 |
| 206_153 | A_33_P3423825 | THC2681947 | DAXX | 0.060487423 | 0.647499538 | 0.907204968 | 1 | -0.050997557 | 0.685750191 | 0.8685753 | 1 |
| 141_79 | A_23_P165247 | NM_170711 | DAZAP1 | 0.185866022 | 0.276152947 | 0.716856302 | 1 | 0.13754794 | 0.468075645 | 0.7297542 | 1 |
| 6_66 | A_23_P40025 | NM_014764 | DAZAP2 | 0.038366377 | 0.759724813 | 0.942653053 | 1 | -0.164971917 | 0.23340533 | 0.5211417 | 1 |
| 87_126 | A_33_P3682006 | NR_102735 | DBH-AS1 | -0.163218548 | 0.311838794 | 0.743773338 | 1 | -0.058444197 | 0.75200816 | 0.9007747 | 1 |
| 76_4 | A_33_P3244931 | NM_001079863 | DBI | -0.12037835 | 0.458598548 | 0.833745766 | 1 | 0.263388588 | 0.097972703 | 0.3406027 | 1 |
| 345_128 | A_23_P156284 | NM_080881 | DBN1 | -0.135819957 | 0.343618611 | 0.767452043 | 1 | -0.331189614 | 0.011871081 | 0.1018414 | 1 |
| 186_20 | A_23_P28772 | NM_001048226 | DBNDD2 | 0.065172474 | 0.614439909 | 0.897816866 | 1 | -0.025127994 | 0.849630865 | 0.9450036 | 1 |
| 215_39 | A_33_P3308949 | NM_001918 | DBT | -0.059659792 | 0.713469123 | 0.93206229 | 1 | 0.048525154 | 0.72507989 | 0.8878578 | 1 |
| 157_90 | A_23_P215875 | NM_015420 | DCAF13 | 0.242926568 | 0.093637566 | 0.496537113 | 1 | 0.032549453 | 0.825858266 | 0.9336456 | 1 |
| 214_22 | A_33_P3420426 | NR_027642 | DCAF13P3 | -0.063514955 | 0.678293596 | 0.91912932 | 1 | 0.186898137 | 0.128337862 | 0.389219 | 1 |
| 333_78 | A_33_P3296308 | NM_017741 | DCAF16 | -0.248716219 | 0.100028343 | 0.507331785 | 1 | 0.47076728 | 0.012382414 | 0.1043184 | 1 |
| 245_69 | A_24_P241318 | NM_181340 | DCAF4 | 0.093413922 | 0.531144098 | 0.865842607 | 1 | -0.135401714 | 0.245133939 | 0.5323463 | 1 |
| 59_107 | A_24_P916141 | NM_005828 | DCAF7 | 0.063808235 | 0.62150104 | 0.89867047 | 1 | -0.107097695 | 0.437749435 | 0.7090907 | 1 |
| 111_73 | A_24_P51061 | NM_080927 | DCBLD2 | -0.439326821 | 0.022531853 | 0.31323834 | 1 | 0.310061131 | 0.032582803 | 0.1844282 | 1 |
| 271_98 | A_24_P137434 | NM_080927 | DCBLD2 | -0.226496419 | 0.269139768 | 0.711669341 | 1 | -0.164992507 | 0.477327632 | 0.7363193 | 1 |
| 61_105 | A_23_P75038 | NM_014881 | DCLRE1A | 0.075942177 | 0.583775559 | 0.884262315 | 1 | -0.021038883 | 0.868868772 | 0.9535682 | 1 |
| 314_94 | A_23_P86632 | NM_001033858 | DCLRE1C | -0.130575067 | 0.336722115 | 0.762727557 | 1 | -0.006989369 | 0.951968177 | 0.983258 | 1 |
| 134_6 | A_23_P166826 | NM_018403 | DCP1A | -0.001971336 | 0.988477762 | 0.99652193 | 1 | 0.111908122 | 0.384638371 | 0.6656373 | 1 |
| 370_111 | A_24_P576174 | NM_001290205 | DCP1A | 0.228694485 | 0.100033734 | 0.507331785 | 1 | 0.05149511 | 0.672063768 | 0.8618742 | 1 |
| 298_143 | A_23_P313512 | NM_152640 | DCP1B | -0.209241051 | 0.106414006 | 0.521311656 | 1 | 0.219048009 | 0.142000799 | 0.4088465 | 1 |
| 172_108 | A_23_P52738 | NM_014026 | DCPS | 0.120340333 | 0.484318887 | 0.843975968 | 1 | -0.067318268 | 0.648527743 | 0.8473011 | 1 |
| 360_38 | A_33_P3264577 | NM_004082 | DCTN1 | -0.043149392 | 0.790430482 | 0.94877096 | 1 | 0.104184265 | 0.440913395 | 0.7114015 | 1 |
| 259_58 | A_23_P360167 | NM_006400 | DCTN2 | -0.102909172 | 0.441408034 | 0.82834854 | 1 | -0.105924249 | 0.495013627 | 0.7507029 | 1 |
| 154_126 | A_23_P158024 | NM_007234 | DCTN3 | 0.148002064 | 0.361755045 | 0.77846455 | 1 | -0.296938033 | 0.037074071 | 0.1977108 | 1 |
| 153_5 | A_23_P43049 | NM_006571 | DCTN6 | 0.164540898 | 0.318992861 | 0.750332309 | 1 | -0.222625947 | 0.136667587 | 0.4012883 | 1 |
| 302_6 | A_23_P33613 | NM_024096 | DCTPP1 | 0.096423466 | 0.447316028 | 0.830485224 | 1 | -0.318072701 | 0.104550027 | 0.3512642 | 1 |
| 180_142 | A_32_P104000 | NM_173475 | DCUN1D3 | 0.063038512 | 0.616432328 | 0.89867047 | 1 | -0.005888306 | 0.96244539 | 0.9869879 | 1 |
| 155_10 | A_23_P127533 | NM_032299 | DCUN1D5 | 0.068104731 | 0.799695396 | 0.951548003 | 1 | 0.087856593 | 0.710300568 | 0.8795214 | 1 |
| 281_125 | A_23_P44166 | NM_016286 | DCXR | 0.102539621 | 0.49504872 | 0.849990629 | 1 | -0.554945869 | 0.010131339 | 0.0922632 | 1 |
| 279_96 | A_23_P201386 | NM_012137 | DDAH1 | 0.140398487 | 0.444370519 | 0.829470233 | 1 | 0.096986371 | 0.399183882 | 0.6775764 | 1 |
| 92_117 | A_23_P19482 | NM_013974 | DDAH2 | -0.650005127 | 0.000279984 | 0.058806327 | 1 | -0.121492321 | 0.617897252 | 0.8267298 | 1 |
| 36_42 | A_23_P258246 | NM_001923 | DDB1 | 0.253735065 | 0.113259441 | 0.533330694 | 1 | -0.436317995 | 0.051103279 | 0.2362345 | 1 |
| 1_48 | A_23_P52610 | NM_000107 | DDB2 | 0.439820472 | 0.016550749 | 0.290939723 | 1 | -0.031393766 | 0.789373059 | 0.9167033 | 1 |
| 335_126 | A_23_P429491 | NM_145018 | DDIAS | 0.12412365 | 0.3379916 | 0.764040391 | 1 | -0.109600524 | 0.356423882 | 0.6412167 | 1 |
| 303_78 | A_23_P149099 | NM_005216 | DDOST | 0.225906356 | 0.120665664 | 0.543014125 | 1 | -0.055050845 | 0.735772009 | 0.893156 | 1 |
| 336_118 | A_33_P3268338 | NM_023935 | DDRGK1 | -0.088163622 | 0.509913499 | 0.856625408 | 1 | 0.151814805 | 0.213549957 | 0.4991166 | 1 |
| 299_17 | A_23_P17769 | NM_001355 | DDT | -0.046620893 | 0.723249145 | 0.936289665 | 1 | -0.107041453 | 0.376264219 | 0.6580382 | 1 |
| 107_6 | A_23_P108554 | NM_004939 | DDX1 | 0.025870164 | 0.836548146 | 0.961943507 | 1 | -0.187174997 | 0.129545118 | 0.3906541 | 1 |
| 345_133 | A_23_P108554 | NM_004939 | DDX1 | 0.102144437 | 0.4564767 | 0.83358833 | 1 | -0.280140876 | 0.055304497 | 0.2478126 | 1 |
| 57_77 | A_23_P108554 | NM_004939 | DDX1 | 0.13086369 | 0.314493859 | 0.745268067 | 1 | -0.288574847 | 0.030809457 | 0.1784713 | 1 |
| 41_70 | A_23_P108554 | NM_004939 | DDX1 | 0.148926811 | 0.242063199 | 0.68817127 | 1 | -0.21839122 | 0.099104239 | 0.3425401 | 1 |
| 223_94 | A_23_P108554 | NM_004939 | DDX1 | 0.176551516 | 0.280783624 | 0.721874328 | 1 | -0.264565384 | 0.128428509 | 0.3892586 | 1 |
| 194_144 | A_23_P108554 | NM_004939 | DDX1 | 0.180594203 | 0.198783823 | 0.645258298 | 1 | -0.310017934 | 0.035053497 | 0.1922632 | 1 |
| 298_77 | A_23_P108554 | NM_004939 | DDX1 | 0.203175276 | 0.124970593 | 0.550792967 | 1 | -0.222285441 | 0.085982531 | 0.3153067 | 1 |
| 161_123 | A_23_P108554 | NM_004939 | DDX1 | 0.209527451 | 0.127569293 | 0.553674154 | 1 | -0.382866594 | 0.015241716 | 0.1182102 | 1 |
| 266_87 | A_23_P108554 | NM_004939 | DDX1 | 0.224442055 | 0.10986065 | 0.527106118 | 1 | -0.282104341 | 0.058269871 | 0.2548316 | 1 |
| 147_153 | A_23_P108554 | NM_004939 | DDX1 | 0.260632912 | 0.106026291 | 0.520758893 | 1 | -0.320691734 | 0.060870657 | 0.2602099 | 1 |
| 56_72 | A_23_P32938 | NM_004398 | DDX10 | 0.059133121 | 0.769753722 | 0.945351992 | 1 | -0.165935036 | 0.303422062 | 0.593208 | 1 |
| 333_92 | A_23_P203949 | NM_030653 | DDX11 | -0.002981746 | 0.982803043 | 0.995672988 | 1 | -0.040681958 | 0.706330194 | 0.8776067 | 1 |
| 153_83 | A_23_P203947 | NM_030653 | DDX11 | 0.029811171 | 0.857762471 | 0.965745263 | 1 | -0.079418975 | 0.678746276 | 0.8659096 | 1 |
| 348_47 | A_33_P3235340 | NM_006773 | DDX18 | -0.096921576 | 0.507185384 | 0.855587859 | 1 | -0.002113125 | 0.988760681 | 0.9965581 | 1 |
| 160_138 | A_33_P3242080 | NM_018332 | DDX19A | 0.196899622 | 0.187690934 | 0.630397286 | 1 | -0.005739201 | 0.956082617 | 0.9847126 | 1 |
| 42_151 | A_23_P63153 | NM_007204 | DDX20 | 0.211782489 | 0.117409492 | 0.53981859 | 1 | -0.100051551 | 0.390305952 | 0.6711162 | 1 |
| 27_92 | A_33_P3287815 | NM_001256910 | DDX21 | 0.30025535 | 0.071891874 | 0.456574115 | 1 | 0.045889551 | 0.825286979 | 0.9336255 | 1 |
| 268_83 | A_23_P64770 | NM_004818 | DDX23 | 0.057760343 | 0.693818683 | 0.924234842 | 1 | -0.251466208 | 0.16796957 | 0.4450303 | 1 |
| 110_93 | A_23_P40194 | NM_017895 | DDX27 | 0.180680546 | 0.165734047 | 0.604663721 | 1 | -0.002715052 | 0.981176038 | 0.9934423 | 1 |
| 370_7 | A_23_P106633 | NM_018380 | DDX28 | -0.013810025 | 0.950434994 | 0.99027625 | 1 | 0.029578638 | 0.827407232 | 0.9341346 | 1 |
| 378_19 | A_23_P106633 | NM_018380 | DDX28 | 0.009866525 | 0.963865762 | 0.993394139 | 1 | 0.102139171 | 0.473849081 | 0.7341756 | 1 |
| 373_33 | A_23_P106633 | NM_018380 | DDX28 | 0.054390853 | 0.822912573 | 0.957894143 | 1 | 0.096850015 | 0.503814052 | 0.7571515 | 1 |
| 181_35 | A_23_P106633 | NM_018380 | DDX28 | 0.198139329 | 0.237397921 | 0.683441761 | 1 | -0.084122627 | 0.514954156 | 0.7618492 | 1 |
| 33_66 | A_23_P106633 | NM_018380 | DDX28 | 0.264419248 | 0.178704634 | 0.618460184 | 1 | -0.060916166 | 0.630739621 | 0.8354644 | 1 |
| 195_80 | A_23_P106633 | NM_018380 | DDX28 | 0.269638424 | 0.123970353 | 0.548567688 | 1 | 0.039203246 | 0.787448814 | 0.9158446 | 1 |
| 36_130 | A_23_P106633 | NM_018380 | DDX28 | 0.304562805 | 0.204267861 | 0.650587432 | 1 | -0.095127791 | 0.51332311 | 0.7612651 | 1 |
| 19_33 | A_23_P106633 | NM_018380 | DDX28 | 0.306361534 | 0.118681325 | 0.540937779 | 1 | -0.01561088 | 0.893638196 | 0.9630927 | 1 |
| 25_91 | A_23_P106633 | NM_018380 | DDX28 | 0.322440818 | 0.162153418 | 0.602072342 | 1 | -0.031672664 | 0.821728531 | 0.9316513 | 1 |
| 221_97 | A_23_P106633 | NM_018380 | DDX28 | 0.464071394 | 0.009447207 | 0.241777062 | 1 | -0.087747984 | 0.659184095 | 0.8547476 | 1 |
| 252_162 | A_33_P3305368 | NM_138620 | DDX31 | 0.104559158 | 0.419986128 | 0.817246292 | 1 | -0.010910673 | 0.932051301 | 0.9756722 | 1 |
| 197_62 | A_23_P78664 | NM_005804 | DDX39A | 0.17410041 | 0.25295311 | 0.696693372 | 1 | -0.25803468 | 0.280838866 | 0.5717743 | 1 |
| 36_26 | A_23_P122116 | NM_016222 | DDX41 | 0.062294547 | 0.611287043 | 0.89742483 | 1 | -0.042014659 | 0.796369189 | 0.9198135 | 1 |
| 262_143 | A_23_P65089 | NM_016355 | DDX47 | 0.250617717 | 0.072344462 | 0.457123394 | 1 | -0.149833674 | 0.242458331 | 0.5300153 | 1 |
| 78_47 | A_23_P16573 | NM_019070 | DDX49 | 0.141836866 | 0.358976762 | 0.776265051 | 1 | -0.280462651 | 0.186743434 | 0.4667341 | 1 |
| 9_152 | A_23_P15511 | NM_004396 | DDX5 | 0.541465068 | 0.047995585 | 0.400741754 | 1 | -0.190335426 | 0.543548833 | 0.7832535 | 1 |
| 260_153 | A_33_P3372886 | NM_175066 | DDX51 | 0.300148908 | 0.036840834 | 0.369146715 | 1 | -0.215202869 | 0.089286573 | 0.3222741 | 1 |
| 280_142 | A_33_P3372859 | NM_001111322 | DDX54 | -0.011007534 | 0.936579088 | 0.987099233 | 1 | -0.258320863 | 0.081714106 | 0.3077445 | 1 |
| 189_71 | A_32_P430359 | NM_024072 | DDX54 | 0.089096933 | 0.512166203 | 0.85794362 | 1 | 0.161716322 | 0.30557022 | 0.59474 | 1 |
| 19_151 | A_23_P47839 | NM_020936 | DDX55 | 0.115093394 | 0.395524094 | 0.800591379 | 1 | -0.006510434 | 0.958211729 | 0.9855517 | 1 |
| 67_140 | A_33_P3357062 | NM_020936 | DDX55 | 0.201741468 | 0.14207105 | 0.575849374 | 1 | -0.044860443 | 0.679523949 | 0.8663124 | 1 |
| 100_123 | A_33_P3372844 | NM_019082 | DDX56 | 0.220594948 | 0.088125581 | 0.484889713 | 1 | -0.390963937 | 0.004189359 | 0.0542206 | 1 |
| 201_107 | A_32_P80255 | NM_001257191 | DDX6 | 0.015025217 | 0.90335909 | 0.976981573 | 1 | -0.153193253 | 0.179989155 | 0.4584099 | 1 |
| 308_103 | A_23_P124044 | NM_021008 | DEAF1 | -0.071284839 | 0.633138777 | 0.901716027 | 1 | -0.167559357 | 0.276569788 | 0.568134 | 1 |
| 278_45 | A_23_P100315 | NM_020664 | DECR2 | -0.260539279 | 0.074283718 | 0.460521251 | 1 | 0.133639581 | 0.242956104 | 0.5306909 | 1 |
| 266_69 | A_24_P207479 | NM_133328 | DEDD2 | 0.118573135 | 0.368236151 | 0.782211132 | 1 | -0.158310229 | 0.319646954 | 0.6061659 | 1 |
| 331_99 | A_24_P193582 | NM_017702 | DEF8 | 0.09104126 | 0.511094369 | 0.856978323 | 1 | 0.163452129 | 0.20460603 | 0.4873367 | 1 |
| 177_72 | A_23_P88893 | NM_017702 | DEF8 | 0.127389429 | 0.366808043 | 0.781989605 | 1 | 0.583192671 | 0.005054492 | 0.0605409 | 1 |
| 208_127 | A_33_P3290179 | NM_003676 | DEGS1 | 0.073401977 | 0.622913111 | 0.899250294 | 1 | 0.049106389 | 0.67891725 | 0.8660295 | 1 |
| 125_133 | A_33_P3290174 | NM_206918 | DEGS2 | 0.230468477 | 0.411771199 | 0.812009855 | 1 | -0.043783309 | 0.882312361 | 0.9592556 | 1 |
| 168_65 | A_23_P254702 | NM_003472 | DEK | 0.483993454 | 0.024684752 | 0.318700868 | 1 | -0.140690685 | 0.30246094 | 0.5929491 | 1 |
| 366_99 | A_32_P175301 | NM_014957 | DENND3 | -0.333266812 | 0.021535336 | 0.308342332 | 1 | 0.155317552 | 0.19675053 | 0.4790873 | 1 |
| 221_67 | A_33_P3238573 | NM_014856 | DENND4B | 0.118521457 | 0.363143626 | 0.779115119 | 1 | 0.029775373 | 0.850365006 | 0.9451344 | 1 |
| 369_43 | A_33_P3293668 | XM_006716804 | DENND4C | -0.152102386 | 0.276397476 | 0.716856302 | 1 | -0.246346936 | 0.067110779 | 0.2758753 | 1 |
| 184_76 | A_32_P118586 | NM_152678 | DENND6A | 0.042510507 | 0.750451694 | 0.942041156 | 1 | -0.04946647 | 0.736405891 | 0.8931957 | 1 |
| 87_64 | A_23_P200310 | NM_017779 | DEPDC1 | -0.164103961 | 0.356871409 | 0.774963546 | 1 | 0.17400689 | 0.122682063 | 0.3802855 | 1 |
| 257_99 | A_23_P25253 | NM_015954 | DERA | 0.086319651 | 0.528675043 | 0.865741443 | 1 | 0.428429523 | 0.010384144 | 0.0936571 | 1 |
| 99_44 | A_23_P118749 | NM_016041 | DERL2 | 0.17912222 | 0.240009166 | 0.68521014 | 1 | -0.071334454 | 0.61955281 | 0.8275666 | 1 |
| 187_77 | A_33_P3724155 | NM_198440 | DERL3 | -0.078493313 | 0.601757644 | 0.893172034 | 1 | 0.197503142 | 0.137240034 | 0.4017539 | 1 |
| 285_27 | A_33_P3275801 | NM_001927 | DES | -0.099907146 | 0.413955362 | 0.812514622 | 1 | 0.363276384 | 0.005883338 | 0.0669736 | 1 |
| 144_34 | A_23_P356330 | NM_015704 | DESI1 | 0.104742171 | 0.459670689 | 0.833986451 | 1 | -0.000797761 | 0.994857425 | 0.9975355 | 1 |
| 203_111 | A_33_P3876985 | NM_016076 | DESI2 | 0.110165823 | 0.390573097 | 0.796961054 | 1 | -0.16597005 | 0.169745977 | 0.4470604 | 1 |
| 14_158 | A_23_P201445 | NM_016076 | DESI2 | 0.315357857 | 0.056509454 | 0.423246544 | 1 | -0.249040509 | 0.130982459 | 0.392253 | 1 |
| 366_42 | A_23_P26184 | NM_017996 | DET1 | 0.078589903 | 0.630535913 | 0.901004617 | 1 | 0.268185366 | 0.096940802 | 0.3386183 | 1 |
| 3_151 | A_24_P144377 | NM_014015 | DEXI | 0.100574922 | 0.458050343 | 0.833745766 | 1 | -0.15268043 | 0.246203885 | 0.5330231 | 1 |
| 171_77 | A_23_P12189 | NM_213566 | DFFA | -0.114096069 | 0.377285744 | 0.789702212 | 1 | 0.039152149 | 0.743929587 | 0.8965225 | 1 |
| 192_149 | A_22_P00005087 | NM_012079 | DGAT1 | -0.209053649 | 0.261930872 | 0.704586552 | 1 | 0.058489662 | 0.69642078 | 0.8734794 | 1 |
| 108_18 | A_24_P125881 | NM_005137 | DGCR2 | -0.139582429 | 0.270088656 | 0.713030987 | 1 | -0.20900249 | 0.115689187 | 0.3685404 | 1 |
| 286_125 | A_21_P0012248 | NR_110533 | DGCR5 | -0.08522503 | 0.484398609 | 0.843975968 | 1 | 0.020040678 | 0.873024928 | 0.9558909 | 1 |
| 127_46 | A_23_P211355 | NM_022720 | DGCR8 | -0.003377205 | 0.980495934 | 0.995429381 | 1 | 0.101655868 | 0.46998706 | 0.731315 | 1 |
| 55_113 | A_23_P210253 | NM_152879 | DGKD | 0.106919913 | 0.588657357 | 0.88673919 | 1 | 0.071282633 | 0.613638824 | 0.8240856 | 1 |
| 80_21 | A_33_P3216427 | NM_001347 | DGKQ | -0.388656396 | 0.0356602 | 0.363957443 | 1 | -0.078486107 | 0.549780044 | 0.787379 | 1 |
| 204_72 | A_33_P3239338 | NM_001105540 | DGKZ | -0.0628362 | 0.607815433 | 0.895626057 | 1 | -0.170845675 | 0.390623073 | 0.6713536 | 1 |
| 67_64 | A_24_P285623 | NM_080916 | DGUOK | 0.168673619 | 0.216676894 | 0.662917773 | 1 | 0.16196551 | 0.191967614 | 0.4737897 | 1 |
| 193_160 | A_22_P00002126 | NR_104030 | DGUOK-AS1 | -0.615710539 | 0.009786428 | 0.244299724 | 1 | 0.281448544 | 0.038104744 | 0.2007326 | 1 |
| 4_114 | A_23_P379475 | NM_014762 | DHCR24 | 0.023468924 | 0.854254935 | 0.965445182 | 1 | 0.170129138 | 0.298915374 | 0.5892903 | 1 |
| 237_152 | A_23_P24444 | NM_001360 | DHCR7 | -0.116122881 | 0.458882271 | 0.833745766 | 1 | 0.191548929 | 0.224056089 | 0.5124806 | 1 |
| 236_45 | A_32_P211045 | NM_000791 | DHFR | -0.366015369 | 0.053040537 | 0.414085647 | 1 | 0.028597427 | 0.886245921 | 0.9608259 | 1 |
| 13_147 | A_24_P343095 | NM_000791 | DHFR | 0.107951213 | 0.423211034 | 0.818992346 | 1 | 0.215065684 | 0.134882219 | 0.3986168 | 1 |
| 298_36 | A_23_P15202 | NM_001361 | DHODH | 0.032255718 | 0.849434016 | 0.965069985 | 1 | 0.242560489 | 0.058084581 | 0.2544906 | 1 |
| 335_124 | A_23_P66719 | NM_144683 | DHRS13 | 0.105270043 | 0.493331268 | 0.849586735 | 1 | -0.456132613 | 0.003110553 | 0.045025 | 1 |
| 191_44 | A_33_P3213997 | NM_001193636 | DHRS4L2 | -0.512300326 | 0.014271108 | 0.274645554 | 1 | 0.256466399 | 0.196494111 | 0.4787628 | 1 |
| 309_131 | A_33_P3383436 | NM_001193636 | DHRS4L2 | 0.005956084 | 0.962428155 | 0.993286853 | 1 | -0.043797523 | 0.753899494 | 0.9012175 | 1 |
| 272_10 | A_23_P117506 | NM_016029 | DHRS7 | -0.017885623 | 0.893504799 | 0.975081715 | 1 | 0.070097744 | 0.562760086 | 0.7958803 | 1 |
| 188_70 | A_23_P117506 | NM_016029 | DHRS7 | 0.000139349 | 0.99924298 | 0.999924748 | 1 | 0.165848534 | 0.150446158 | 0.4187073 | 1 |
| 185_106 | A_23_P117506 | NM_016029 | DHRS7 | 0.005149466 | 0.969257704 | 0.994075478 | 1 | 0.075792421 | 0.472352228 | 0.7329059 | 1 |
| 222_70 | A_23_P117506 | NM_016029 | DHRS7 | 0.043452044 | 0.751268518 | 0.942061365 | 1 | 0.205570916 | 0.112105429 | 0.3634581 | 1 |
| 76_67 | A_23_P117506 | NM_016029 | DHRS7 | 0.046191576 | 0.699282035 | 0.92523784 | 1 | 0.188247078 | 0.139861647 | 0.4055147 | 1 |
| 211_96 | A_23_P117506 | NM_016029 | DHRS7 | 0.098891333 | 0.439806624 | 0.827447767 | 1 | 0.115080602 | 0.340837472 | 0.6275003 | 1 |
| 343_85 | A_23_P117506 | NM_016029 | DHRS7 | 0.11345978 | 0.374896552 | 0.788505348 | 1 | 0.050953948 | 0.668312163 | 0.8596188 | 1 |
| 195_116 | A_23_P117506 | NM_016029 | DHRS7 | 0.139465556 | 0.281545621 | 0.722517117 | 1 | -0.002508586 | 0.982653382 | 0.9938607 | 1 |
| 155_7 | A_23_P141208 | NM_015510 | DHRS7B | -0.205667796 | 0.115347346 | 0.537023243 | 1 | 0.019747578 | 0.860179566 | 0.9500287 | 1 |
| 258_43 | A_24_P378987 | NM_145177 | DHRSX | -0.234044079 | 0.089398673 | 0.486837117 | 1 | -1.107310666 | 1.66231E-06 | 0.0008319 | 0.0187 |
| 160_133 | A_23_P168062 | NM_003587 | DHX16 | 0.121981911 | 0.390183103 | 0.796961054 | 1 | -0.152862557 | 0.234712217 | 0.522424 | 1 |
| 367_10 | A_33_P3315874 | NM_014966 | DHX30 | -0.265698919 | 0.107764885 | 0.523859467 | 1 | 0.294152377 | 0.040341821 | 0.2068967 | 1 |
| 196_47 | A_33_P3315856 | NM_014681 | DHX34 | -0.136118855 | 0.386123293 | 0.795277691 | 1 | 0.179384075 | 0.237697067 | 0.5258707 | 1 |
| 352_107 | A_23_P5945 | NM_021931 | DHX35 | 0.097324903 | 0.439660813 | 0.827404459 | 1 | -0.025894596 | 0.82246668 | 0.9320184 | 1 |
| 19_140 | A_23_P57697 | NM_020865 | DHX36 | 0.137267006 | 0.271389392 | 0.714790846 | 1 | 0.046204666 | 0.699090623 | 0.874605 | 1 |
| 60_154 | A_23_P13914 | NM_032656 | DHX37 | 0.155371326 | 0.314402242 | 0.745268067 | 1 | -0.15341718 | 0.278679983 | 0.5696383 | 1 |
| 374_5 | A_33_P3330125 | NM_001278302 | DIABLO | -0.378119501 | 0.018699681 | 0.299872244 | 1 | 0.336484072 | 0.114535924 | 0.3665867 | 1 |
| 300_74 | A_23_P47800 | NM_019887 | DIABLO | -0.06956464 | 0.568257691 | 0.881160458 | 1 | -0.022002687 | 0.843928854 | 0.9414469 | 1 |
| 332_102 | A_24_P323104 | NM_005219 | DIAPH1 | -0.385028779 | 0.022749335 | 0.313925645 | 1 | 0.222775593 | 0.082944807 | 0.3097583 | 1 |
| 62_50 | A_32_P150891 | NM_001042517 | DIAPH3 | -0.351639789 | 0.020317773 | 0.306188729 | 1 | 0.008898417 | 0.942409274 | 0.9799185 | 1 |
| 73_42 | A_33_P3212490 | NM_001195573 | DICER1 | 0.077449226 | 0.602763364 | 0.893514876 | 1 | 0.134165993 | 0.23797816 | 0.5259462 | 1 |
| 94_64 | A_23_P388190 | NM_033081 | DIDO1 | -0.133189324 | 0.315393386 | 0.746188042 | 1 | -0.04201473 | 0.725789801 | 0.8881185 | 1 |
| 121_85 | A_23_P58529 | NM_014473 | DIMT1 | 0.257743321 | 0.074972602 | 0.461185525 | 1 | -0.001081867 | 0.993628167 | 0.9974469 | 1 |
| 295_94 | A_23_P131139 | NM_052952 | DIRC1 | 0.487511461 | 0.03965315 | 0.376797721 | 1 | 0.171115373 | 0.346597869 | 0.6325201 | 1 |
| 293_32 | A_23_P80778 | NM_032839 | DIRC2 | -0.16300454 | 0.307147783 | 0.740176924 | 1 | 0.06990067 | 0.587436718 | 0.8111603 | 1 |
| 235_91 | A_23_P65741 | NM_133375 | DIS3L | 0.425760574 | 0.014645314 | 0.276603442 | 1 | -0.144363055 | 0.346600995 | 0.6325201 | 1 |
| 354_30 | A_23_P324340 | NM_033510 | DISP2 | -0.191996652 | 0.271521144 | 0.714790846 | 1 | 0.649957373 | 0.009884109 | 0.0911201 | 1 |
| 342_126 | A_23_P137143 | NM_001363 | DKC1 | 0.445064621 | 0.006600514 | 0.221508151 | 1 | 0.330377012 | 0.1284377 | 0.3892586 | 1 |
| 373_60 | A_23_P203030 | NM_001931 | DLAT | -0.219290037 | 0.109042316 | 0.526512446 | 1 | -0.372723234 | 0.010728539 | 0.0956098 | 1 |
| 213_34 | A_23_P151337 | NR_002605 | DLEU1 | 0.06170743 | 0.649438204 | 0.907984842 | 1 | -0.306073414 | 0.035508091 | 0.1933008 | 1 |
| 370_115 | A_33_P3286218 | NR_002771 | DLEU2L | -0.128097329 | 0.312135091 | 0.743773338 | 1 | -0.155043884 | 0.257656834 | 0.5471068 | 1 |
| 172_76 | A_33_P3224380 | NM_001098424 | DLG1 | -0.128133285 | 0.384565241 | 0.793491491 | 1 | -0.062028385 | 0.603030533 | 0.8190176 | 1 |
| 352_121 | A_24_P945283 | NM_021120 | DLG3 | -0.265529672 | 0.048873603 | 0.403099681 | 1 | -0.174424529 | 0.182793415 | 0.4617957 | 1 |
| 4_130 | A_24_P129632 | NM_004747 | DLG5 | 0.114119783 | 0.43070514 | 0.821617604 | 1 | -0.138566312 | 0.23770351 | 0.5258707 | 1 |
| 24_52 | A_33_P3368785 | NR_024101 | DLGAP1-AS1 | -0.119859244 | 0.392667175 | 0.798048073 | 1 | 0.478514498 | 0.007376747 | 0.0771974 | 1 |
| 29_129 | A_22_P00016054 | NR_024101 | DLGAP1-AS1 | -0.060019278 | 0.621345691 | 0.89867047 | 1 | 0.155075339 | 0.3572918 | 0.6420292 | 1 |
| 229_48 | A_24_P216253 | NM_014902 | DLGAP4 | -0.325205442 | 0.060053098 | 0.428867769 | 1 | -0.231212831 | 0.099874908 | 0.3434127 | 1 |
| 151_15 | A_23_P88331 | NM_014750 | DLGAP5 | 0.006980407 | 0.964649475 | 0.993394139 | 1 | -0.216216132 | 0.19979426 | 0.4832215 | 1 |
| 382_95 | A_33_P3254460 | NM_206539 | DLK2 | 0.136464312 | 0.362357897 | 0.778723103 | 1 | 0.103653247 | 0.378869887 | 0.6609916 | 1 |
| 93_71 | A_23_P205697 | NM_001933 | DLST | 0.147522126 | 0.308121628 | 0.740628314 | 1 | -0.348130388 | 0.090604359 | 0.324932 | 1 |
| 45_3 | A_33_P3246885 | NM_001190348 | DMKN | -0.004515735 | 0.974526411 | 0.994877235 | 1 | 0.008374854 | 0.939938823 | 0.9788735 | 1 |
| 290_162 | A_23_P8664 | NM_021145 | DMTF1 | 0.074225908 | 0.581428403 | 0.883808109 | 1 | -0.136729938 | 0.295108774 | 0.5861007 | 1 |
| 187_98 | A_32_P19806 | NM_001277115 | DNAH11 | -0.0498502 | 0.757413152 | 0.942483836 | 1 | 0.054639388 | 0.724032624 | 0.8873483 | 1 |
| 52_12 | A_32_P87531 | NM_001145154 | DNAH14 | 0.057604289 | 0.637097127 | 0.903216232 | 1 | -0.177781081 | 0.131316161 | 0.3929007 | 1 |
| 238_144 | A_23_P333951 | NM_144989 | DNAH14 | 0.100246769 | 0.417198744 | 0.814996006 | 1 | -0.200704059 | 0.137006965 | 0.4016897 | 1 |
| 273_48 | A_24_P9671 | NM_001539 | DNAJA1 | 0.321903558 | 0.0624488 | 0.433893961 | 1 | -0.213562565 | 0.237710205 | 0.5258707 | 1 |
| 122_56 | A_33_P3414574 | NM_005880 | DNAJA2 | -0.008442948 | 0.953025554 | 0.990760353 | 1 | -0.162696379 | 0.205302848 | 0.487832 | 1 |
| 331_22 | A_23_P118306 | NM_005147 | DNAJA3 | 0.049177439 | 0.719610636 | 0.934805056 | 1 | -0.283605674 | 0.079527891 | 0.3037182 | 1 |
| 117_34 | A_23_P118306 | NM_005147 | DNAJA3 | 0.18549515 | 0.176744238 | 0.616374573 | 1 | -0.235743828 | 0.142961954 | 0.4102453 | 1 |
| 341_46 | A_23_P118306 | NM_005147 | DNAJA3 | 0.252625167 | 0.085135872 | 0.478632531 | 1 | -0.242216526 | 0.113938853 | 0.366122 | 1 |
| 118_44 | A_23_P118306 | NM_005147 | DNAJA3 | 0.264145265 | 0.057094028 | 0.423322858 | 1 | -0.228233922 | 0.147669893 | 0.4163547 | 1 |
| 136_129 | A_23_P118306 | NM_005147 | DNAJA3 | 0.306700758 | 0.03080875 | 0.343045838 | 1 | -0.24325616 | 0.120024976 | 0.3759178 | 1 |
| 200_150 | A_23_P118306 | NM_005147 | DNAJA3 | 0.318569036 | 0.031343007 | 0.344912707 | 1 | -0.305299862 | 0.035469891 | 0.1932862 | 1 |
| 262_94 | A_23_P118306 | NM_005147 | DNAJA3 | 0.331607312 | 0.02688365 | 0.329142336 | 1 | -0.334234594 | 0.079717096 | 0.3040752 | 1 |
| 316_98 | A_23_P118306 | NM_005147 | DNAJA3 | 0.344338468 | 0.017467528 | 0.294775747 | 1 | -0.282336322 | 0.087572062 | 0.3189517 | 1 |
| 151_118 | A_23_P118306 | NM_005147 | DNAJA3 | 0.377396681 | 0.014394117 | 0.275053663 | 1 | -0.413534637 | 0.012022843 | 0.1024814 | 1 |
| 14_118 | A_23_P118306 | NM_005147 | DNAJA3 | 0.518597264 | 0.002646471 | 0.154236866 | 1 | -0.36713856 | 0.022118653 | 0.1462484 | 1 |
| 209_80 | A_23_P90062 | NM_006145 | DNAJB1 | 0.160145663 | 0.219665319 | 0.666470871 | 1 | -0.186425582 | 0.161263347 | 0.4351557 | 1 |
| 64_122 | A_23_P166899 | NM_016306 | DNAJB11 | -0.233030342 | 0.144086515 | 0.578530626 | 1 | 0.269248257 | 0.110049574 | 0.3597 | 1 |
| 277_50 | A_24_P244100 | NM_001002762 | DNAJB12 | -0.226148382 | 0.159018724 | 0.597775747 | 1 | -0.044157124 | 0.686722221 | 0.8687924 | 1 |
| 26_47 | A_24_P102981 | NM_006736 | DNAJB2 | 0.058575382 | 0.730559728 | 0.936838487 | 1 | 0.649098621 | 0.000357735 | 0.0113978 | 1 |
| 162_91 | A_23_P112241 | NM_012266 | DNAJB5 | -0.263795585 | 0.117279834 | 0.53981859 | 1 | 0.083427763 | 0.430088261 | 0.7032862 | 1 |
| 153_39 | A_32_P229746 | NM_005494 | DNAJB6 | 0.564196364 | 0.007647342 | 0.230091297 | 1 | -0.204996786 | 0.152340011 | 0.4207682 | 1 |
| 113_27 | A_33_P3290687 | NM_022365 | DNAJC1 | -0.361190762 | 0.043855586 | 0.390521637 | 1 | -0.049927221 | 0.66048336 | 0.8551551 | 1 |
| 84_162 | A_23_P127128 | NM_022365 | DNAJC1 | -0.326625772 | 0.151913216 | 0.590116963 | 1 | -0.185736845 | 0.153808554 | 0.4224383 | 1 |
| 98_110 | A_23_P319133 | NM_018981 | DNAJC10 | -0.020236646 | 0.881563239 | 0.972820007 | 1 | 0.685691004 | 0.000913395 | 0.0212154 | 1 |
| 384_62 | A_23_P103942 | NM_018198 | DNAJC11 | -0.009437186 | 0.943172365 | 0.989214443 | 1 | -0.241465226 | 0.063440887 | 0.2664375 | 1 |
| 112_147 | A_23_P121396 | NM_145261 | DNAJC19 | 0.085148765 | 0.50559358 | 0.854723979 | 1 | -0.096691492 | 0.428926652 | 0.7022927 | 1 |
| 350_164 | A_33_P3362869 | NM_001190233 | DNAJC19 | 0.225656182 | 0.120379549 | 0.543014125 | 1 | -0.103073383 | 0.446101927 | 0.7151353 | 1 |
| 311_68 | A_23_P254573 | NM_014377 | DNAJC2 | -0.027282632 | 0.866008601 | 0.968485888 | 1 | -0.038526491 | 0.746189077 | 0.8976273 | 1 |
| 305_73 | A_33_P3369939 | NM_194283 | DNAJC21 | -0.241921332 | 0.085670733 | 0.479213799 | 1 | -0.17604088 | 0.25143497 | 0.5393181 | 1 |
| 305_151 | A_33_P3310533 | NM_001012339 | DNAJC21 | -0.170430044 | 0.246258775 | 0.691627025 | 1 | 0.041561417 | 0.745766012 | 0.8976273 | 1 |
| 235_79 | A_33_P3462960 | NM_006260 | DNAJC3 | 0.010484985 | 0.934543821 | 0.986418035 | 1 | -0.199930702 | 0.083404058 | 0.3109199 | 1 |
| 109_137 | A_23_P202769 | NM_005528 | DNAJC4 | 0.145605567 | 0.386390582 | 0.795277691 | 1 | 0.002784523 | 0.985519215 | 0.9949293 | 1 |
| 121_34 | A_33_P3363153 | NM_025219 | DNAJC5 | -0.174616058 | 0.343628395 | 0.767452043 | 1 | 0.037892473 | 0.769622992 | 0.9081774 | 1 |
| 133_26 | A_33_P3216714 | NM_001256864 | DNAJC6 | -0.390577803 | 0.008246256 | 0.234464792 | 1 | -0.047531815 | 0.675728381 | 0.8643983 | 1 |
| 63_20 | A_23_P46455 | NM_014280 | DNAJC8 | -0.115464324 | 0.541191195 | 0.869568619 | 1 | 0.049654818 | 0.710565643 | 0.8795214 | 1 |
| 93_19 | A_23_P104372 | NM_015190 | DNAJC9 | -0.095911113 | 0.526601423 | 0.864679678 | 1 | 0.01255619 | 0.929136292 | 0.9744494 | 1 |
| 368_66 | A_23_P104372 | NM_015190 | DNAJC9 | -0.0100742 | 0.947111204 | 0.99002169 | 1 | 0.038222177 | 0.775321262 | 0.9103379 | 1 |
| 53_1 | A_23_P104372 | NM_015190 | DNAJC9 | 0.008261963 | 0.963471204 | 0.993314675 | 1 | 0.075625288 | 0.637422871 | 0.8405552 | 1 |
| 267_74 | A_23_P104372 | NM_015190 | DNAJC9 | 0.058750897 | 0.667170428 | 0.916076943 | 1 | 0.060501457 | 0.659983047 | 0.8551551 | 1 |
| 155_33 | A_23_P104372 | NM_015190 | DNAJC9 | 0.059390504 | 0.708473548 | 0.9294233 | 1 | 0.021659803 | 0.879406545 | 0.9582221 | 1 |
| 355_77 | A_23_P104372 | NM_015190 | DNAJC9 | 0.069780063 | 0.611069188 | 0.89742483 | 1 | 0.012308371 | 0.934203598 | 0.9765392 | 1 |
| 123_140 | A_23_P104372 | NM_015190 | DNAJC9 | 0.085314439 | 0.499731006 | 0.8517872 | 1 | -0.023386399 | 0.835642652 | 0.9377842 | 1 |
| 104_91 | A_23_P104372 | NM_015190 | DNAJC9 | 0.11542316 | 0.378659312 | 0.790179752 | 1 | 0.019094049 | 0.893299039 | 0.9630927 | 1 |
| 269_92 | A_23_P104372 | NM_015190 | DNAJC9 | 0.160618903 | 0.231617701 | 0.677668275 | 1 | 0.039963093 | 0.792470465 | 0.9182892 | 1 |
| 9_47 | A_23_P104372 | NM_015190 | DNAJC9 | 0.164839821 | 0.275814414 | 0.716856302 | 1 | 0.134075113 | 0.294128932 | 0.5853942 | 1 |
| 265_123 | A_23_P25913 | NM_031427 | DNAL1 | -0.091065724 | 0.477412795 | 0.840908892 | 1 | 0.295537772 | 0.061078412 | 0.2609689 | 1 |
| 166_34 | A_32_P161166 | AY927536 | DNASE1L1 | -0.194356904 | 0.54369569 | 0.871017839 | 1 | 1.140905832 | 0.003107662 | 0.045025 | 1 |
| 19_83 | A_32_P42054 | NM_194249 | DND1 | -0.324866822 | 0.036714537 | 0.369070022 | 1 | 0.11930311 | 0.533180571 | 0.7741207 | 1 |
| 294_93 | A_33_P3358403 | NM_001080849 | DNLZ | -0.067755084 | 0.575082229 | 0.883213781 | 1 | 0.214460121 | 0.142464357 | 0.409585 | 1 |
| 164_93 | A_33_P3243264 | NM_001080849 | DNLZ | 0.237721283 | 0.135095808 | 0.565739819 | 1 | -0.051826026 | 0.705702362 | 0.8771922 | 1 |
| 226_7 | A_33_P3329419 | NM_001288739 | DNM1 | -0.124500893 | 0.341611171 | 0.766714725 | 1 | 0.192255103 | 0.161985017 | 0.4364747 | 1 |
| 144_93 | A_23_P204324 | NM_012062 | DNM1L | 0.105053938 | 0.397494574 | 0.801548559 | 1 | -0.042745649 | 0.717870506 | 0.8837108 | 1 |
| 183_35 | A_23_P407074 | NM_001005360 | DNM2 | 0.204999152 | 0.178097042 | 0.617761436 | 1 | -0.159029721 | 0.245244069 | 0.5323799 | 1 |
| 187_21 | A_21_P0000785 | NR_038397 | DNM3OS | -0.095058943 | 0.449740873 | 0.83175092 | 1 | 0.012712166 | 0.915265233 | 0.9704901 | 1 |
| 76_47 | A_33_P3329187 | NM_001130823 | DNMT1 | 0.013815982 | 0.916286679 | 0.98101793 | 1 | -0.028798227 | 0.807627563 | 0.9249885 | 1 |
| 277_29 | A_23_P28953 | NM_175850 | DNMT3B | -0.39634834 | 0.01679774 | 0.29259326 | 1 | -0.102552262 | 0.484403909 | 0.7420445 | 1 |
| 192_23 | A_33_P3372451 | NM_199184 | DNPH1 | -0.453229187 | 0.011984555 | 0.2646124 | 1 | -0.168903908 | 0.384768049 | 0.6656373 | 1 |
| 286_86 | A_23_P131816 | NM_052951 | DNTTIP1 | 0.158992051 | 0.290693899 | 0.728413293 | 1 | -0.086097082 | 0.66361571 | 0.8565307 | 1 |
| 129_16 | A_23_P97584 | NM_014597 | DNTTIP2 | -0.113582256 | 0.376065535 | 0.78918168 | 1 | -0.090907578 | 0.529701517 | 0.7721038 | 1 |
| 187_52 | A_33_P3379801 | NM_001083908 | DNAAF2 | -0.092690578 | 0.516531849 | 0.860004647 | 1 | -0.110112207 | 0.314020872 | 0.6015453 | 1 |
| 161_97 | A_23_P258689 | NM_017802 | DNAAF5 | 0.495442678 | 0.015594386 | 0.283139497 | 1 | -0.52837869 | 0.070913396 | 0.2833261 | 1 |
| 230_132 | A_23_P5601 | NM_001381 | DOK1 | -0.387318787 | 0.011205385 | 0.256586468 | 1 | 0.449084816 | 0.0076571 | 0.0789362 | 1 |
| 90_45 | A_23_P10870 | NM_014908 | DOLK | -0.020587814 | 0.882389574 | 0.972820007 | 1 | -0.131774765 | 0.31521515 | 0.6026219 | 1 |
| 39_104 | A_23_P10870 | NM_014908 | DOLK | -0.019314296 | 0.87635736 | 0.971233905 | 1 | -0.217134227 | 0.079318658 | 0.3036132 | 1 |
| 273_69 | A_23_P10870 | NM_014908 | DOLK | 0.009208146 | 0.9425295 | 0.988968248 | 1 | -0.174335883 | 0.15225844 | 0.4206462 | 1 |
| 175_132 | A_23_P10870 | NM_014908 | DOLK | 0.027135905 | 0.826636561 | 0.959621444 | 1 | -0.194668651 | 0.113254527 | 0.3657078 | 1 |
| 234_147 | A_23_P10870 | NM_014908 | DOLK | 0.063070959 | 0.616873327 | 0.89867047 | 1 | -0.156465337 | 0.183196195 | 0.4623895 | 1 |
| 24_67 | A_23_P10870 | NM_014908 | DOLK | 0.065542466 | 0.611290835 | 0.89742483 | 1 | -0.141093501 | 0.218724435 | 0.5052005 | 1 |
| 381_142 | A_23_P10870 | NM_014908 | DOLK | 0.071895265 | 0.61732081 | 0.89867047 | 1 | -0.172525824 | 0.147144937 | 0.4161275 | 1 |
| 340_62 | A_23_P10870 | NM_014908 | DOLK | 0.1282448 | 0.318614864 | 0.750101906 | 1 | -0.098886929 | 0.36661942 | 0.6506815 | 1 |
| 149_87 | A_23_P10870 | NM_014908 | DOLK | 0.130567308 | 0.332498002 | 0.76120935 | 1 | -0.175702845 | 0.157475225 | 0.4290513 | 1 |
| 138_100 | A_23_P10870 | NM_014908 | DOLK | 0.252537408 | 0.084696866 | 0.478632531 | 1 | -0.263220581 | 0.053552585 | 0.242796 | 1 |
| 176_22 | A_23_P386764 | NM_020438 | DOLPP1 | 0.111920904 | 0.392014229 | 0.797647793 | 1 | -0.131132297 | 0.30453176 | 0.5937483 | 1 |
| 81_11 | A_23_P425502 | NM_017613 | DONSON | -0.016183938 | 0.906844826 | 0.978222335 | 1 | -0.024145746 | 0.835557451 | 0.9377842 | 1 |
| 227_71 | A_33_P3240200 | NM_015448 | DPCD | -0.021875505 | 0.865517287 | 0.968292001 | 1 | 0.088336732 | 0.570857579 | 0.801432 | 1 |
| 229_65 | A_33_P3286046 | NM_015448 | DPCD | 0.139058331 | 0.355199447 | 0.774004626 | 1 | -0.147323881 | 0.223842454 | 0.5122189 | 1 |
| 58_61 | A_33_P3679768 | AW303581 | DPCR1 | -0.179086815 | 0.220893945 | 0.667332686 | 1 | 0.13610106 | 0.229611831 | 0.5176276 | 1 |
| 98_88 | A_24_P393844 | NM_001384 | DPH2 | 0.130304781 | 0.391734011 | 0.797477226 | 1 | 0.089931579 | 0.490196506 | 0.7464713 | 1 |
| 236_90 | A_23_P69188 | NM_206831 | DPH3 | 0.269527422 | 0.08484601 | 0.478632531 | 1 | 0.335367396 | 0.064586093 | 0.2689253 | 1 |
| 75_46 | A_23_P258814 | NM_080750 | DPH3P1 | 0.156795969 | 0.231941581 | 0.677743425 | 1 | 0.249859719 | 0.07427645 | 0.2913803 | 1 |
| 62_43 | A_23_P148821 | NM_001077394 | DPH5 | 0.30095113 | 0.043805235 | 0.390521637 | 1 | 0.084205946 | 0.516343818 | 0.7630494 | 1 |
| 19_50 | A_23_P68472 | NM_003859 | DPM1 | 0.226795783 | 0.101848785 | 0.510939533 | 1 | 0.102131409 | 0.435568483 | 0.7078419 | 1 |
| 373_78 | A_23_P217079 | NM_003863 | DPM2 | 0.036308488 | 0.798449023 | 0.951453193 | 1 | -0.287250283 | 0.032388595 | 0.1837914 | 1 |
| 231_126 | A_33_P3257460 | NM_018973 | DPM3 | -0.188128759 | 0.19576444 | 0.64211158 | 1 | -0.139348223 | 0.292242477 | 0.5842211 | 1 |
| 49_83 | A_33_P3577671 | NM_130434 | DPP8 | 0.112711389 | 0.467988541 | 0.836476951 | 1 | 0.027811307 | 0.839162392 | 0.9393908 | 1 |
| 258_90 | A_33_P3292028 | NM_139159 | DPP9 | 0.033545396 | 0.788162012 | 0.94877096 | 1 | -0.019651194 | 0.885426529 | 0.9608259 | 1 |
| 160_121 | A_33_P3347040 | NM_001242901 | DPP9-AS1 | -0.175189173 | 0.405161871 | 0.806763544 | 1 | -0.212300973 | 0.211175465 | 0.4959471 | 1 |
| 350_30 | A_23_P394448 | NM_015283 | DPY19L1 | -0.126394914 | 0.351034594 | 0.771478158 | 1 | -0.136399935 | 0.374295871 | 0.6567611 | 1 |
| 321_139 | A_23_P5389 | NM_032574 | DPY30 | 0.069243272 | 0.708116581 | 0.929339527 | 1 | 0.165401362 | 0.198482749 | 0.4812105 | 1 |
| 97_34 | A_33_P3410296 | NM_006426 | DPYSL4 | -0.049390138 | 0.815670444 | 0.955983966 | 1 | 0.208017674 | 0.272168579 | 0.563009 | 1 |
| 150_29 | A_23_P391725 | NM_001938 | DR1 | -0.140775427 | 0.391156792 | 0.797101378 | 1 | -0.120635848 | 0.370838648 | 0.6547717 | 1 |
| 58_92 | A_23_P99163 | NM_018370 | DRAM1 | -0.054250495 | 0.754337997 | 0.942207062 | 1 | -0.129438951 | 0.323676446 | 0.6102538 | 1 |
| 135_89 | A_23_P138856 | NM_006442 | DRAP1 | -0.207452664 | 0.152879892 | 0.591420402 | 1 | -0.063722519 | 0.657819196 | 0.8536394 | 1 |
| 164_161 | A_23_P68884 | NM_004147 | DRG1 | 0.157161697 | 0.288330709 | 0.727382494 | 1 | -0.270175516 | 0.042658311 | 0.2137096 | 1 |
| 23_72 | A_23_P141656 | NM_001388 | DRG2 | 0.127458324 | 0.411622463 | 0.812009855 | 1 | -0.097146419 | 0.424587596 | 0.6991546 | 1 |
| 191_131 | A_23_P133596 | NM_013235 | DROSHA | -0.014953412 | 0.907364058 | 0.978283157 | 1 | 0.195447968 | 0.096581057 | 0.3382574 | 1 |
| 315_156 | A_33_P3244753 | NM_001939 | DRP2 | 0.000967505 | 0.994635154 | 0.99865206 | 1 | -0.038148809 | 0.798992284 | 0.9211015 | 1 |
| 293_89 | A_23_P252740 | NM_024094 | DSCC1 | 0.106911621 | 0.384656832 | 0.793546067 | 1 | 0.241715027 | 0.08436558 | 0.3127421 | 1 |
| 202_29 | A_23_P154874 | NM_006052 | DSCR3 | -0.099808203 | 0.44945313 | 0.831477605 | 1 | 0.100159865 | 0.486090337 | 0.7430087 | 1 |
| 9_55 | A_24_P254346 | NM_006052 | DSCR3 | 0.019669064 | 0.885895393 | 0.973024604 | 1 | -0.144051719 | 0.354002522 | 0.6387721 | 1 |
| 348_102 | A_23_P165937 | NM_024918 | DSN1 | 0.055427188 | 0.725650831 | 0.936348816 | 1 | -0.12790432 | 0.265505877 | 0.5559755 | 1 |
| 241_66 | A_32_P157945 | NM_004415 | DSP | 0.033312763 | 0.838644904 | 0.962281738 | 1 | -0.282598311 | 0.214792728 | 0.5008861 | 1 |
| 253_88 | A_23_P408095 | NM_001011546 | DSTN | 0.125356328 | 0.346236282 | 0.768529418 | 1 | -0.039901828 | 0.78540396 | 0.9151101 | 1 |
| 285_87 | A_23_P17512 | NM_080820 | DTD1 | 0.197008336 | 0.252460506 | 0.696364465 | 1 | 0.078072659 | 0.654083708 | 0.8510504 | 1 |
| 353_33 | A_23_P10385 | NM_016448 | DTL | -0.240211394 | 0.069145015 | 0.452356175 | 1 | 0.034042578 | 0.77429883 | 0.9097197 | 1 |
| 378_50 | A_23_P10385 | NM_016448 | DTL | -0.202555255 | 0.184751939 | 0.62763442 | 1 | -0.002858351 | 0.984518823 | 0.9949293 | 1 |
| 86_38 | A_23_P10385 | NM_016448 | DTL | -0.188063912 | 0.171172645 | 0.611486797 | 1 | 0.036692605 | 0.739578359 | 0.8946865 | 1 |
| 340_7 | A_23_P10385 | NM_016448 | DTL | -0.134137244 | 0.331017067 | 0.760452185 | 1 | -0.093411342 | 0.462035604 | 0.7245761 | 1 |
| 246_44 | A_23_P10385 | NM_016448 | DTL | -0.09840271 | 0.426143839 | 0.820625513 | 1 | -0.048067362 | 0.66227775 | 0.8562637 | 1 |
| 279_154 | A_23_P10385 | NM_016448 | DTL | -0.050074631 | 0.708648095 | 0.929497449 | 1 | -0.070632341 | 0.532019637 | 0.7734338 | 1 |
| 10_20 | A_23_P10385 | NM_016448 | DTL | -0.039442247 | 0.767605223 | 0.945351992 | 1 | 0.076070121 | 0.495812068 | 0.750974 | 1 |
| 81_106 | A_23_P10385 | NM_016448 | DTL | -0.025389677 | 0.841553423 | 0.963334317 | 1 | -0.041853567 | 0.723697049 | 0.8871476 | 1 |
| 130_70 | A_23_P10385 | NM_016448 | DTL | 0.070737507 | 0.631356359 | 0.901004617 | 1 | -0.137482963 | 0.227134461 | 0.5151404 | 1 |
| 25_86 | A_23_P10385 | NM_016448 | DTL | 0.082214 | 0.640587346 | 0.905712044 | 1 | -0.036869423 | 0.762197043 | 0.9053173 | 1 |
| 137_113 | A_33_P3371785 | NM_001128175 | DTNA | 0.124448228 | 0.480427464 | 0.842220317 | 1 | -0.070663541 | 0.704783913 | 0.87673 | 1 |
| 90_115 | A_23_P3212 | NM_020234 | DTWD1 | 0.125316563 | 0.384555721 | 0.793491491 | 1 | -0.0874628 | 0.544641387 | 0.7838236 | 1 |
| 379_3 | A_23_P123974 | NM_012145 | DTYMK | -0.376765282 | 0.021568044 | 0.308342332 | 1 | 0.610050551 | 0.001300673 | 0.0259848 | 1 |
| 346_34 | A_33_P3266530 | NM_022156 | DUS1L | -0.050653782 | 0.757968894 | 0.942483836 | 1 | -0.370155635 | 0.009189546 | 0.0872193 | 1 |
| 325_5 | A_23_P255569 | NM_022156 | DUS1L | 0.034318009 | 0.78222132 | 0.948624895 | 1 | -0.388214388 | 0.027105982 | 0.1653259 | 1 |
| 8_53 | A_33_P3344264 | NM_020175 | DUS3L | 0.104700269 | 0.414060973 | 0.812514622 | 1 | -0.015389739 | 0.917270324 | 0.9710598 | 1 |
| 126_79 | A_23_P51508 | NM_007240 | DUSP12 | 0.211173437 | 0.12387382 | 0.548482971 | 1 | -0.063668537 | 0.631225964 | 0.8356345 | 1 |
| 150_152 | A_23_P207537 | NM_007026 | DUSP14 | 0.303560885 | 0.118693702 | 0.540937779 | 1 | -0.227998693 | 0.119217713 | 0.3748509 | 1 |
| 79_134 | A_23_P154771 | NM_080611 | DUSP15 | -0.001632951 | 0.989223248 | 0.996750836 | 1 | 0.252596586 | 0.162393793 | 0.4369185 | 1 |
| 82_126 | A_23_P120254 | NM_020185 | DUSP22 | 0.00840289 | 0.944360814 | 0.989683091 | 1 | -0.144111883 | 0.207334359 | 0.4909241 | 1 |
| 295_84 | A_33_P3272698 | NM_017823 | DUSP23 | -0.505031215 | 0.001571288 | 0.127946984 | 1 | 0.589388523 | 0.000412047 | 0.0125591 | 1 |
| 15_142 | A_33_P3383656 | NM_001033575 | DUSP28 | -0.050793896 | 0.726119723 | 0.936348816 | 1 | 0.395835159 | 0.046368532 | 0.2231523 | 1 |
| 156_162 | A_23_P129956 | NM_004090 | DUSP3 | 0.015067221 | 0.915295233 | 0.98101793 | 1 | -0.185994748 | 0.108490364 | 0.3573957 | 1 |
| 49_137 | A_23_P134935 | NM_001394 | DUSP4 | 0.044101746 | 0.748229414 | 0.941597731 | 1 | 0.278533818 | 0.030250882 | 0.1766912 | 1 |
| 274_159 | A_23_P139704 | NM_001946 | DUSP6 | -0.161420433 | 0.333103654 | 0.76120935 | 1 | 0.293594111 | 0.030891188 | 0.1784713 | 1 |
| 355_59 | A_33_P3359012 | NM_004420 | DUSP8 | -0.054827488 | 0.685242168 | 0.921676062 | 1 | 0.165540929 | 0.24473926 | 0.5321113 | 1 |
| 307_27 | A_24_P160874 | NM_001025248 | DUT | -0.185363779 | 0.214979528 | 0.661348172 | 1 | 0.409877937 | 0.004785657 | 0.0585683 | 1 |
| 47_139 | A_23_P88484 | NM_001025248 | DUT | -0.044959338 | 0.752005344 | 0.942109358 | 1 | 0.391621555 | 0.014785252 | 0.1160431 | 1 |
| 325_140 | A_23_P104413 | NM_001293798 | DUX4 | -0.061018965 | 0.652095326 | 0.908340249 | 1 | 0.351841172 | 0.1256236 | 0.3853351 | 1 |
| 65_48 | A_23_P201342 | NM_004421 | DVL1 | -0.198508834 | 0.208803297 | 0.655712085 | 1 | -0.073936633 | 0.586477762 | 0.8110447 | 1 |
| 308_163 | A_23_P55342 | NM_004422 | DVL2 | 0.07473598 | 0.581233908 | 0.883808109 | 1 | -0.078381268 | 0.49057434 | 0.7464713 | 1 |
| 14_116 | A_32_P181020 | NM_005510 | DXO | 0.08682857 | 0.594698166 | 0.889988626 | 1 | -0.290024198 | 0.030754887 | 0.178391 | 1 |
| 379_35 | A_23_P170518 | NM_017653 | DYM | -0.086174876 | 0.653066389 | 0.908802233 | 1 | -0.172035942 | 0.31223713 | 0.6008481 | 1 |
| 23_37 | A_23_P128706 | NM_001376 | DYNC1H1 | 0.080989948 | 0.757273023 | 0.942483836 | 1 | 0.214986491 | 0.418910194 | 0.6952135 | 1 |
| 271_44 | A_23_P154108 | NM_001378 | DYNC1I2 | -0.22064527 | 0.133957247 | 0.564269428 | 1 | 0.26927836 | 0.06189154 | 0.2630223 | 1 |
| 195_45 | A_24_P124672 | NM_001037494 | DYNLL1 | 0.178800468 | 0.243444183 | 0.689446137 | 1 | -0.196548548 | 0.144475279 | 0.4121485 | 1 |
| 249_106 | A_23_P65031 | NM_001037494 | DYNLL1 | 0.229586665 | 0.134622952 | 0.56452101 | 1 | -0.232335134 | 0.116995883 | 0.3709903 | 1 |
| 258_149 | A_23_P54991 | NM_080677 | DYNLL2 | 0.198754957 | 0.219182979 | 0.666463497 | 1 | 0.30169159 | 0.016470029 | 0.1231179 | 1 |
| 273_70 | A_24_P143440 | NM_014183 | DYNLRB1 | 0.1028734 | 0.466913644 | 0.83630364 | 1 | -0.031329828 | 0.804299112 | 0.9231312 | 1 |
| 58_98 | A_23_P8185 | NM_006519 | DYNLT1 | 0.042676363 | 0.749487117 | 0.941597731 | 1 | -0.627101191 | 0.000250134 | 0.0091339 | 1 |
| 50_81 | A_24_P91852 | NM_006520 | DYNLT3 | -0.131877866 | 0.298457906 | 0.73421127 | 1 | -0.793615567 | 0.000188532 | 0.0075192 | 1 |
| 196_115 | A_24_P100368 | NM_006520 | DYNLT3 | 0.178383178 | 0.189732199 | 0.632544514 | 1 | -0.266963108 | 0.057740829 | 0.2535541 | 1 |
| 260_24 | A_33_P3318861 | NM_001093730 | DYTN | -0.05429455 | 0.788585872 | 0.94877096 | 1 | -0.027236598 | 0.881003322 | 0.9588224 | 1 |
| 80_74 | A_23_P144165 | NM_014648 | DZIP3 | -0.088084825 | 0.483329066 | 0.843540785 | 1 | 0.360240449 | 0.016212321 | 0.1221508 | 1 |
| 57_26 | A_23_P80032 | NM_005225 | E2F1 | -0.294870359 | 0.17055569 | 0.611161363 | 1 | 0.046530173 | 0.791498306 | 0.9175409 | 1 |
| 349_103 | A_23_P80032 | NM_005225 | E2F1 | -0.243742213 | 0.133665291 | 0.56362429 | 1 | 0.00731317 | 0.964100893 | 0.9873043 | 1 |
| 336_36 | A_23_P80032 | NM_005225 | E2F1 | -0.217286125 | 0.247830042 | 0.693087358 | 1 | 0.096266238 | 0.604408409 | 0.819588 | 1 |
| 94_57 | A_23_P80032 | NM_005225 | E2F1 | -0.174746832 | 0.251607008 | 0.695501831 | 1 | 0.087121705 | 0.543426905 | 0.7831782 | 1 |
| 307_123 | A_23_P80032 | NM_005225 | E2F1 | -0.158074945 | 0.355942382 | 0.774004626 | 1 | 0.024860132 | 0.84979193 | 0.9450036 | 1 |
| 55_27 | A_23_P80032 | NM_005225 | E2F1 | -0.129947487 | 0.419809208 | 0.817246292 | 1 | 0.038038599 | 0.771516393 | 0.9087179 | 1 |
| 273_114 | A_23_P80032 | NM_005225 | E2F1 | -0.120570027 | 0.523448722 | 0.863661195 | 1 | 0.010941514 | 0.95232221 | 0.983361 | 1 |
| 311_146 | A_23_P80032 | NM_005225 | E2F1 | -0.08255476 | 0.638018133 | 0.90407548 | 1 | -0.023302218 | 0.869768827 | 0.9542093 | 1 |
| 276_118 | A_23_P80032 | NM_005225 | E2F1 | -0.048825019 | 0.782253627 | 0.948624895 | 1 | -0.019482179 | 0.914191909 | 0.9704901 | 1 |
| 13_19 | A_23_P80032 | NM_005225 | E2F1 | -0.035449059 | 0.843211135 | 0.963873328 | 1 | 0.103215443 | 0.483215787 | 0.7413351 | 1 |
| 333_153 | A_23_P408955 | NM_004091 | E2F2 | -0.125100837 | 0.361152419 | 0.777994476 | 1 | -0.133066701 | 0.373383234 | 0.6561627 | 1 |
| 152_73 | A_23_P385034 | NM_001949 | E2F3 | 0.445192978 | 0.008852587 | 0.239118 | 1 | -0.123397924 | 0.346315316 | 0.6324245 | 1 |
| 139_13 | A_23_P170774 | NM_198256 | E2F6 | -0.336536253 | 0.017470702 | 0.294775747 | 1 | 0.203070202 | 0.096649553 | 0.3382574 | 1 |
| 122_151 | A_32_P210202 | NM_203394 | E2F7 | -0.079479239 | 0.610899646 | 0.89742483 | 1 | -0.084117741 | 0.502552696 | 0.7563734 | 1 |
| 299_109 | A_23_P128783 | NM_018453 | EAPP | -0.072412866 | 0.589257816 | 0.88697963 | 1 | 0.289660494 | 0.072342029 | 0.2870963 | 1 |
| 208_117 | A_23_P6561 | NM_018029 | EBLN2 | -0.04645707 | 0.733567747 | 0.937867226 | 1 | -0.025315983 | 0.867990652 | 0.9533206 | 1 |
| 318_35 | A_23_P103631 | NM_006824 | EBNA1BP2 | -0.130311573 | 0.407927506 | 0.808864993 | 1 | 0.070836365 | 0.634208941 | 0.8379873 | 1 |
| 239_32 | A_23_P103631 | NM_006824 | EBNA1BP2 | -0.125446452 | 0.35172814 | 0.771839653 | 1 | 0.020626466 | 0.86347554 | 0.9508117 | 1 |
| 252_4 | A_23_P103631 | NM_006824 | EBNA1BP2 | -0.096607266 | 0.509953001 | 0.856625408 | 1 | 0.051142878 | 0.689846057 | 0.8705008 | 1 |
| 371_137 | A_23_P103631 | NM_006824 | EBNA1BP2 | -0.027538178 | 0.842838223 | 0.963718343 | 1 | -0.018347988 | 0.877330913 | 0.9576224 | 1 |
| 301_74 | A_23_P103631 | NM_006824 | EBNA1BP2 | 0.002689737 | 0.985421598 | 0.995672988 | 1 | 0.03867837 | 0.751524718 | 0.9007747 | 1 |
| 297_119 | A_23_P103631 | NM_006824 | EBNA1BP2 | 0.008752127 | 0.952675129 | 0.990752085 | 1 | -0.028718519 | 0.826906671 | 0.9338627 | 1 |
| 100_140 | A_23_P103631 | NM_006824 | EBNA1BP2 | 0.021024243 | 0.880220539 | 0.972152911 | 1 | -0.067777692 | 0.60233385 | 0.8190176 | 1 |
| 104_125 | A_23_P103631 | NM_006824 | EBNA1BP2 | 0.07828153 | 0.597416543 | 0.89144727 | 1 | -0.081118917 | 0.52074512 | 0.7668003 | 1 |
| 72_120 | A_23_P103631 | NM_006824 | EBNA1BP2 | 0.087314259 | 0.611390184 | 0.89742483 | 1 | -0.057189612 | 0.674774446 | 0.8639786 | 1 |
| 223_102 | A_23_P103631 | NM_006824 | EBNA1BP2 | 0.249975455 | 0.059407547 | 0.428428001 | 1 | -0.093530511 | 0.625523069 | 0.8320976 | 1 |
| 294_125 | A_23_P171077 | NM_006579 | EBP | -0.675808644 | 0.000801244 | 0.096211064 | 1 | -1.103870076 | 2.81517E-06 | 0.0010909 | 0.03166 |
| 229_102 | A_33_P3366073 | NR_103802 | EBPL | -0.005795921 | 0.962020223 | 0.993286853 | 1 | 0.054465034 | 0.734910838 | 0.893156 | 1 |
| 255_83 | A_23_P128554 | NM_032565 | EBPL | 0.109182393 | 0.44306466 | 0.829344657 | 1 | -0.063861992 | 0.610650465 | 0.8225878 | 1 |
| 99_1 | A_23_P115762 | NM_007265 | ECD | -0.038955633 | 0.767035105 | 0.945351992 | 1 | 0.038709295 | 0.732321619 | 0.8914971 | 1 |
| 354_101 | A_23_P115762 | NM_007265 | ECD | 0.090354013 | 0.476339857 | 0.840494226 | 1 | -0.043351904 | 0.731512776 | 0.8912712 | 1 |
| 9_13 | A_23_P115762 | NM_007265 | ECD | 0.108559702 | 0.440304046 | 0.827546746 | 1 | 0.060278164 | 0.61942441 | 0.8275406 | 1 |
| 258_51 | A_23_P115762 | NM_007265 | ECD | 0.115983323 | 0.407184805 | 0.808397213 | 1 | -0.034575836 | 0.753410162 | 0.9012104 | 1 |
| 191_152 | A_23_P115762 | NM_007265 | ECD | 0.15220532 | 0.253497998 | 0.697590309 | 1 | -0.001385405 | 0.989808857 | 0.9969964 | 1 |
| 119_95 | A_23_P115762 | NM_007265 | ECD | 0.209315029 | 0.126036993 | 0.551700557 | 1 | 0.078644696 | 0.531062037 | 0.7728849 | 1 |
| 26_135 | A_23_P115762 | NM_007265 | ECD | 0.209744983 | 0.199908794 | 0.646494862 | 1 | 0.016264512 | 0.902422207 | 0.9656324 | 1 |
| 164_142 | A_23_P115762 | NM_007265 | ECD | 0.230093729 | 0.092763403 | 0.49411621 | 1 | 0.08677135 | 0.527709075 | 0.770898 | 1 |
| 223_101 | A_23_P115762 | NM_007265 | ECD | 0.262508335 | 0.066871667 | 0.446719314 | 1 | 0.015467154 | 0.915243253 | 0.9704901 | 1 |
| 281_120 | A_23_P115762 | NM_007265 | ECD | 0.318775258 | 0.058670308 | 0.425950115 | 1 | -0.032357134 | 0.767020478 | 0.9073239 | 1 |
| 268_15 | A_23_P92261 | NM_032331 | ECE2 | -0.013523133 | 0.922791668 | 0.983082909 | 1 | 0.240337844 | 0.04857758 | 0.2299461 | 1 |
| 93_45 | A_23_P215051 | NM_018479 | ECHDC1 | -0.012973375 | 0.93125152 | 0.9855868 | 1 | -0.332270633 | 0.01213557 | 0.1030882 | 1 |
| 140_42 | A_23_P82206 | NM_018479 | ECHDC1 | 0.17569525 | 0.167801786 | 0.607928988 | 1 | -0.276782428 | 0.042706049 | 0.2137583 | 1 |
| 343_154 | A_23_P200203 | NM_018281 | ECHDC2 | -0.626890319 | 0.00158622 | 0.127946984 | 1 | -0.247164598 | 0.094929847 | 0.3345898 | 1 |
| 155_140 | A_23_P104362 | NM_004092 | ECHS1 | 0.269513311 | 0.246033851 | 0.691627025 | 1 | -0.278324521 | 0.154152152 | 0.4231072 | 1 |
| 360_16 | A_23_P163647 | NM_001919 | ECI1 | -0.408387248 | 0.012446861 | 0.265846757 | 1 | 0.177104521 | 0.125594344 | 0.3853351 | 1 |
| 250_95 | A_23_P156852 | NM_206836 | ECI2 | -0.096684777 | 0.606742255 | 0.895268187 | 1 | 0.237718734 | 0.127921311 | 0.389219 | 1 |
| 161_163 | A_23_P119295 | NM_016581 | ECSIT | 0.133870456 | 0.339588931 | 0.764812785 | 1 | -0.413565235 | 0.006854024 | 0.0736971 | 1 |
| 208_83 | A_23_P503233 | NM_080738 | EDARADD | 0.482571131 | 0.054818566 | 0.420264176 | 1 | -0.517515012 | 0.19270448 | 0.4742761 | 1 |
| 100_82 | A_24_P338992 | NM_025083 | EDC3 | 0.245871893 | 0.146868163 | 0.582026011 | 1 | -0.097902636 | 0.450901307 | 0.7185093 | 1 |
| 58_33 | A_33_P3294392 | NM_014329 | EDC4 | 0.160574996 | 0.339809403 | 0.765167866 | 1 | 0.050433152 | 0.65397017 | 0.8510011 | 1 |
| 318_123 | A_24_P208045 | NM_025191 | EDEM3 | 0.082076556 | 0.584132564 | 0.884306572 | 1 | 0.006647659 | 0.956629137 | 0.9850131 | 1 |
| 362_13 | A_23_P60376 | NM_153200 | EDF1 | -0.156318721 | 0.280028708 | 0.72078101 | 1 | -0.349981516 | 0.024532238 | 0.1561483 | 1 |
| 56_1 | A_23_P214821 | NM_001955 | EDN1 | 0.434257064 | 0.095166698 | 0.498686523 | 1 | 0.166856614 | 0.494775726 | 0.7507029 | 1 |
| 334_101 | A_23_P53217 | NM_152991 | EED | 0.229632619 | 0.092214941 | 0.492270996 | 1 | -0.036707372 | 0.786355534 | 0.9154381 | 1 |
| 91_81 | A_32_P44316 | NM_001402 | EEF1A1 | -0.027765897 | 0.823798916 | 0.958162003 | 1 | 0.158105432 | 0.165679569 | 0.4417392 | 1 |
| 14_86 | A_32_P47701 | NM_001402 | EEF1A1 | 0.022248932 | 0.883923195 | 0.972820007 | 1 | 0.199371011 | 0.087349799 | 0.3184516 | 1 |
| 326_14 | A_32_P15320 | NM_001402 | EEF1A1 | 0.051081229 | 0.687458057 | 0.922029409 | 1 | 0.15600564 | 0.416686377 | 0.6928551 | 1 |
| 79_68 | A_33_P3387272 | NM_001402 | EEF1A1 | 0.079234619 | 0.62700601 | 0.900268731 | 1 | 0.393623422 | 0.006853289 | 0.0736971 | 1 |
| 353_87 | A_24_P763243 | NM_001402 | EEF1A1 | 0.871873899 | 0.045474815 | 0.394573324 | 1 | -0.206604969 | 0.657731761 | 0.8536394 | 1 |
| 326_131 | A_23_P151368 | NM_174928 | EEF1AKMT1 | 0.087311381 | 0.561829995 | 0.87849363 | 1 | -0.198233594 | 0.115064298 | 0.367337 | 1 |
| 65_110 | A_32_P49616 | NM_001959 | EEF1B2 | 0.01957297 | 0.879674439 | 0.972104134 | 1 | 0.537602685 | 0.003459246 | 0.0482108 | 1 |
| 112_69 | A_23_P31840 | NM_032378 | EEF1D | 0.172962345 | 0.460935364 | 0.834778335 | 1 | -0.268191689 | 0.238989374 | 0.5265257 | 1 |
| 356_122 | A_23_P156842 | NM_004280 | EEF1E1 | 0.46064651 | 0.031147846 | 0.344912707 | 1 | -0.210506441 | 0.192476148 | 0.4742761 | 1 |
| 349_99 | A_21_P0011842 | NM_001135650 | EEF1E1 | 0.470879118 | 0.035727017 | 0.363957443 | 1 | -0.23477959 | 0.138747505 | 0.4038543 | 1 |
| 351_13 | A_23_P13344 | NM_001404 | EEF1G | -0.147969612 | 0.329787471 | 0.760452185 | 1 | -0.225635347 | 0.204494477 | 0.4872376 | 1 |
| 70_101 | A_33_P3342410 | NM_013302 | EEF2K | 0.073779531 | 0.621448119 | 0.89867047 | 1 | -0.5966167 | 0.000603331 | 0.0164302 | 1 |
| 244_21 | A_33_P3386932 | NM_201400 | EEF2KMT | -0.045598864 | 0.70744271 | 0.929032427 | 1 | 0.329282615 | 0.01314519 | 0.1083529 | 1 |
| 205_141 | A_33_P3222045 | NM_201400 | EEF2KMT | 0.076433756 | 0.533879714 | 0.866842045 | 1 | -0.168674718 | 0.147487804 | 0.4163547 | 1 |
| 125_35 | A_23_P129014 | NM_145231 | EFCAB11 | -0.210411204 | 0.152445453 | 0.59042598 | 1 | 0.288404419 | 0.05042632 | 0.2346012 | 1 |
| 229_146 | A_32_P137266 | ENST00000461039 | EFCAB7 | -0.259527363 | 0.070651505 | 0.455143645 | 1 | 0.136339229 | 0.304227126 | 0.5935399 | 1 |
| 130_142 | A_23_P23443 | NM_024329 | EFHD2 | 0.305442871 | 0.085144878 | 0.478632531 | 1 | -0.44543607 | 0.008581486 | 0.0846426 | 1 |
| 186_81 | A_23_P152055 | NM_024580 | EFL1 | 0.176796409 | 0.17816568 | 0.617823602 | 1 | 0.039343855 | 0.753554625 | 0.9012104 | 1 |
| 190_52 | A_24_P365807 | NM_004429 | EFNB1 | -0.18277592 | 0.191095659 | 0.635004725 | 1 | 1.316633225 | 1.16199E-06 | 0.0007688 | 0.01307 |
| 56_121 | A_24_P355944 | NM_004093 | EFNB2 | 0.106453066 | 0.557499142 | 0.87699568 | 1 | -0.146212703 | 0.39360419 | 0.6739026 | 1 |
| 162_97 | A_32_P83049 | NM_014971 | EFR3B | -0.458489449 | 0.006196993 | 0.218184309 | 1 | -0.284253322 | 0.160823912 | 0.4344911 | 1 |
| 130_51 | A_23_P55190 | NM_004247 | EFTUD2 | 0.100895754 | 0.468553549 | 0.836476951 | 1 | 0.028355495 | 0.809060134 | 0.9253621 | 1 |
| 164_1 | A_23_P424582 | NM_030652 | EGFL8 | -0.267064455 | 0.189471503 | 0.632291984 | 1 | 0.050783959 | 0.726770155 | 0.888579 | 1 |
| 100_122 | A_23_P343935 | NM_022051 | EGLN1 | -0.223952281 | 0.169268484 | 0.608706095 | 1 | 0.240055614 | 0.100015885 | 0.3434127 | 1 |
| 252_29 | A_23_P214080 | NM_001964 | EGR1 | -0.457920613 | 0.008711261 | 0.237406225 | 1 | -0.129505858 | 0.391531088 | 0.67199 | 1 |
| 35_4 | A_24_P194886 | NM_015252 | EHBP1 | -0.140788772 | 0.271931254 | 0.714985188 | 1 | 0.095189586 | 0.446097472 | 0.7151353 | 1 |
| 15_138 | A_23_P52647 | NM_006795 | EHD1 | 0.03485379 | 0.779518366 | 0.948012382 | 1 | -0.589524871 | 0.00022745 | 0.0085271 | 1 |
| 349_30 | A_22_P00008393 | NR_120332 | EHD4-AS1 | -0.003994223 | 0.983933349 | 0.995672988 | 1 | 0.038077344 | 0.794697439 | 0.9190412 | 1 |
| 336_117 | A_23_P365844 | NM_153232 | EID2 | -0.131500218 | 0.45897427 | 0.833745766 | 1 | -0.142043036 | 0.228500306 | 0.5163118 | 1 |
| 308_160 | A_23_P45945 | NM_005801 | EIF1 | 0.150655699 | 0.351635572 | 0.77182424 | 1 | 0.027010992 | 0.839154582 | 0.9393908 | 1 |
| 24_60 | A_32_P749354 | NM_032325 | EIF1AD | 0.33419784 | 0.039004752 | 0.374228433 | 1 | -0.177367215 | 0.202473876 | 0.4851248 | 1 |
| 113_37 | A_24_P237389 | NM_001412 | EIF1AX | 0.090210551 | 0.473930155 | 0.839074131 | 1 | -1.223268549 | 1.59637E-06 | 0.0008319 | 0.01795 |
| 124_34 | A_33_P3222105 | NM_001412 | EIF1AX | 0.231260952 | 0.143852918 | 0.578207176 | 1 | -0.992369847 | 4.67313E-05 | 0.0035513 | 0.52559 |
| 253_33 | A_33_P3361393 | NM_001412 | EIF1AX | 0.240848859 | 0.106513275 | 0.521311656 | 1 | -0.991605435 | 3.17761E-05 | 0.0029056 | 0.35739 |
| 311_3 | A_23_P148629 | NM_004681 | EIF1AY | 0.153328646 | 0.448632791 | 0.830710542 | 1 | -0.86482369 | 5.56633E-05 | 0.0039623 | 0.62605 |
| 207_89 | A_23_P251173 | NM_014413 | EIF2AK1 | 0.082785476 | 0.525423292 | 0.864256909 | 1 | -0.324301127 | 0.045711453 | 0.2212239 | 1 |
| 320_143 | A_23_P142750 | NM_002759 | EIF2AK2 | -0.369877039 | 0.025844979 | 0.324915933 | 1 | 0.056721208 | 0.686164467 | 0.8685753 | 1 |
| 92_22 | A_23_P105313 | NM_001414 | EIF2B1 | 0.032181671 | 0.848166523 | 0.965069985 | 1 | -0.146335299 | 0.239325337 | 0.5270593 | 1 |
| 214_48 | A_23_P105313 | NM_001414 | EIF2B1 | 0.053906349 | 0.702038095 | 0.926034391 | 1 | -0.087774733 | 0.42898115 | 0.7022927 | 1 |
| 288_66 | A_23_P105313 | NM_001414 | EIF2B1 | 0.090410737 | 0.543495985 | 0.870823107 | 1 | -0.102490442 | 0.389155536 | 0.6699575 | 1 |
| 32_154 | A_23_P105313 | NM_001414 | EIF2B1 | 0.117429273 | 0.38305237 | 0.79197963 | 1 | -0.189213507 | 0.139794506 | 0.4055147 | 1 |
| 308_68 | A_23_P105313 | NM_001414 | EIF2B1 | 0.117633526 | 0.375849496 | 0.789123966 | 1 | -0.169036169 | 0.200646239 | 0.4835834 | 1 |
| 213_131 | A_23_P105313 | NM_001414 | EIF2B1 | 0.153698529 | 0.249433818 | 0.694615167 | 1 | -0.095297923 | 0.375578913 | 0.657828 | 1 |
| 313_128 | A_23_P105313 | NM_001414 | EIF2B1 | 0.162107009 | 0.224354754 | 0.668801096 | 1 | -0.098324226 | 0.4019479 | 0.6799547 | 1 |
| 174_51 | A_23_P105313 | NM_001414 | EIF2B1 | 0.179663398 | 0.262020586 | 0.704586552 | 1 | -0.093134187 | 0.389289592 | 0.6700198 | 1 |
| 303_143 | A_23_P105313 | NM_001414 | EIF2B1 | 0.209884774 | 0.148850292 | 0.585876178 | 1 | -0.093380369 | 0.395337263 | 0.675533 | 1 |
| 13_103 | A_23_P105313 | NM_001414 | EIF2B1 | 0.252623005 | 0.11126204 | 0.529450848 | 1 | -0.123802185 | 0.288151338 | 0.5792383 | 1 |
| 77_146 | A_23_P25929 | NM_014239 | EIF2B2 | 0.176044156 | 0.207760397 | 0.655004439 | 1 | -0.14559124 | 0.358063297 | 0.6429771 | 1 |
| 227_42 | A_33_P3305482 | NM_020365 | EIF2B3 | -0.184038685 | 0.173620833 | 0.613752707 | 1 | -0.070795192 | 0.647649791 | 0.8465966 | 1 |
| 121_96 | A_33_P3296372 | NM_020365 | EIF2B3 | -0.084136432 | 0.572709435 | 0.882573857 | 1 | -0.07066159 | 0.679575141 | 0.8663124 | 1 |
| 372_51 | A_23_P154058 | NM_001034116 | EIF2B4 | 0.073278363 | 0.590702138 | 0.88758105 | 1 | -0.187767711 | 0.114022478 | 0.366122 | 1 |
| 50_87 | A_23_P115346 | NM_006893 | EIF2D | -0.061544367 | 0.662492174 | 0.913260118 | 1 | -0.058531808 | 0.61568957 | 0.8253469 | 1 |
| 342_59 | A_33_P3402171 | NM_004094 | EIF2S1 | -0.138513416 | 0.29816079 | 0.734106423 | 1 | -0.255819767 | 0.153048887 | 0.4216274 | 1 |
| 332_125 | A_32_P157965 | NM_003908 | EIF2S2 | 0.480017 | 0.056560807 | 0.423246544 | 1 | -0.135082323 | 0.416299649 | 0.6925192 | 1 |
| 356_163 | A_23_P86550 | NM_003750 | EIF3A | -0.129916599 | 0.386288391 | 0.795277691 | 1 | 0.02766366 | 0.811414687 | 0.9263311 | 1 |
| 231_134 | A_23_P157072 | NM_001037283 | EIF3B | 0.383250038 | 0.053062106 | 0.414085647 | 1 | -0.810893122 | 0.000757818 | 0.0190675 | 1 |
| 220_63 | A_33_P3239222 | NM_001037283 | EIF3B | 0.679232038 | 0.001354679 | 0.120457642 | 1 | -0.815864685 | 0.009314768 | 0.0876679 | 1 |
| 193_124 | A_33_P3421733 | NM_001199142 | EIF3C | -0.259335751 | 0.056923296 | 0.423246544 | 1 | -0.214071914 | 0.243690612 | 0.5312535 | 1 |
| 382_107 | A_23_P77568 | NM_001037808 | EIF3C | -0.207332527 | 0.128562864 | 0.556755353 | 1 | -0.163346754 | 0.148188771 | 0.4167494 | 1 |
| 199_76 | A_23_P91702 | NM_003753 | EIF3D | 0.045787358 | 0.758118216 | 0.942483836 | 1 | -0.114936139 | 0.4749609 | 0.7348302 | 1 |
| 23_93 | A_23_P43141 | NM_001568 | EIF3E | 0.297180425 | 0.040237967 | 0.378516532 | 1 | -0.252107363 | 0.069216503 | 0.2798267 | 1 |
| 127_42 | A_23_P142776 | NM_003754 | EIF3F | 0.023596113 | 0.851847549 | 0.965069985 | 1 | -0.106666407 | 0.42451264 | 0.6991498 | 1 |
| 344_120 | A_33_P3369581 | NM_003755 | EIF3G | 0.372167057 | 0.076218423 | 0.46283673 | 1 | -0.241424373 | 0.242875387 | 0.5306909 | 1 |
| 58_37 | A_23_P9061 | NM_003756 | EIF3H | -0.142416447 | 0.262627886 | 0.705020251 | 1 | 0.128816948 | 0.256584814 | 0.5457612 | 1 |
| 95_38 | A_23_P85560 | NM_003757 | EIF3I | 0.191890738 | 0.215174998 | 0.661767691 | 1 | -0.216717187 | 0.283122314 | 0.5742688 | 1 |
| 364_9 | A_33_P3369631 | NM_003758 | EIF3J | 0.050213952 | 0.721800997 | 0.935522901 | 1 | 0.119829491 | 0.393517989 | 0.6739026 | 1 |
| 219_97 | A_21_P0012407 | NM_003758 | EIF3J | 0.243670055 | 0.074427997 | 0.460523107 | 1 | -0.210174155 | 0.191587763 | 0.4734453 | 1 |
| 155_131 | A_22_P00015296 | NR_034170 | EIF3J-AS1 | -0.129775777 | 0.304382419 | 0.738652994 | 1 | 0.091666732 | 0.464643438 | 0.7267202 | 1 |
| 135_17 | A_24_P330971 | NM_013234 | EIF3K | 0.139271892 | 0.400142248 | 0.803667875 | 1 | -0.127778818 | 0.441379344 | 0.7115701 | 1 |
| 227_163 | A_23_P57521 | NM_016091 | EIF3L | 0.325777481 | 0.046728809 | 0.399349089 | 1 | -0.256202754 | 0.070069092 | 0.2815977 | 1 |
| 245_138 | A_24_P138361 | NM_006360 | EIF3M | 0.136952299 | 0.298868717 | 0.735073433 | 1 | -0.103912659 | 0.380710812 | 0.6629284 | 1 |
| 286_36 | A_21_P0011135 | NM_001416 | EIF4A1 | 0.391683549 | 0.08850559 | 0.485883323 | 1 | -0.073091456 | 0.817164203 | 0.9293077 | 1 |
| 124_73 | A_23_P121250 | NM_001967 | EIF4A2 | 0.444479899 | 0.079175618 | 0.468212318 | 1 | 0.185551766 | 0.417771764 | 0.6939417 | 1 |
| 58_93 | A_23_P141636 | NM_014740 | EIF4A3 | 0.236794498 | 0.071757218 | 0.456118548 | 1 | -0.203320928 | 0.080891463 | 0.3066511 | 1 |
| 225_77 | A_33_P3294133 | NM_001300821 | EIF4B | -0.019489323 | 0.884787747 | 0.972822545 | 1 | 0.654189898 | 0.00235235 | 0.0372632 | 1 |
| 309_73 | A_23_P118643 | NM_001968 | EIF4E | -0.12612074 | 0.455373843 | 0.832937561 | 1 | 0.020994881 | 0.878655076 | 0.9582221 | 1 |
| 33_92 | A_32_P203300 | NM_001130679 | EIF4E | 0.23353289 | 0.097453301 | 0.500605281 | 1 | -0.124515132 | 0.367917631 | 0.6520028 | 1 |
| 211_85 | A_33_P3308055 | NM_001282958 | EIF4E2 | -0.119251574 | 0.348655547 | 0.770211834 | 1 | 0.377509135 | 0.009152092 | 0.0870106 | 1 |
| 16_52 | A_21_P0014711 | NM_001282958 | EIF4E2 | 0.157377172 | 0.21713347 | 0.663269793 | 1 | 0.407080157 | 0.049499077 | 0.2321139 | 1 |
| 171_28 | A_33_P3308050 | NM_004846 | EIF4E2 | 0.183790287 | 0.202745288 | 0.649115486 | 1 | -0.182885367 | 0.16438901 | 0.4400837 | 1 |
| 265_117 | A_23_P165722 | NM_004846 | EIF4E2 | 0.250321429 | 0.096433769 | 0.500009516 | 1 | 0.054723622 | 0.686432232 | 0.868621 | 1 |
| 118_85 | A_23_P22224 | NM_004095 | EIF4EBP1 | 0.049143077 | 0.745188684 | 0.941232856 | 1 | 0.012627404 | 0.947946833 | 0.9816413 | 1 |
| 32_103 | A_24_P115621 | NM_004096 | EIF4EBP2 | -0.160559065 | 0.234230714 | 0.680457734 | 1 | -0.169903955 | 0.261873131 | 0.5525038 | 1 |
| 135_46 | A_23_P115922 | NM_004096 | EIF4EBP2 | -0.045515813 | 0.744850617 | 0.941128659 | 1 | 0.201562615 | 0.182675986 | 0.4617957 | 1 |
| 382_121 | A_23_P104892 | NM_001418 | EIF4G2 | 0.416062474 | 0.06571273 | 0.443562076 | 1 | -0.566017636 | 0.063607535 | 0.2664409 | 1 |
| 18_75 | A_23_P126241 | NM_003760 | EIF4G3 | -0.038718025 | 0.769979965 | 0.945351992 | 1 | 0.112808542 | 0.314000696 | 0.6015453 | 1 |
| 190_130 | A_21_P0013292 | NM_022170 | EIF4H | 0.083012457 | 0.497489028 | 0.851056251 | 1 | -0.087920506 | 0.435712658 | 0.7079544 | 1 |
| 129_156 | A_32_P168886 | NM_022170 | EIF4H | 0.175367966 | 0.246538469 | 0.691935623 | 1 | -0.200601822 | 0.102194422 | 0.3475203 | 1 |
| 101_156 | A_24_P398810 | NM_001969 | EIF5 | -0.011410854 | 0.948618408 | 0.990231079 | 1 | -0.631799787 | 0.001327895 | 0.0262937 | 1 |
| 126_14 | A_23_P218608 | NM_015904 | EIF5B | -0.039298898 | 0.773113757 | 0.945964253 | 1 | -0.059795243 | 0.647874515 | 0.8466935 | 1 |
| 70_31 | A_23_P218608 | NM_015904 | EIF5B | -0.03041952 | 0.829148114 | 0.959690637 | 1 | -0.1228156 | 0.383405347 | 0.6650744 | 1 |
| 94_52 | A_23_P218608 | NM_015904 | EIF5B | 0.0032363 | 0.980883289 | 0.995429381 | 1 | -0.136294886 | 0.412630314 | 0.688489 | 1 |
| 186_40 | A_23_P218608 | NM_015904 | EIF5B | 0.094427713 | 0.478144311 | 0.840908892 | 1 | -0.147144258 | 0.24106791 | 0.5286587 | 1 |
| 360_98 | A_23_P218608 | NM_015904 | EIF5B | 0.125378035 | 0.412626873 | 0.812009855 | 1 | -0.211356363 | 0.135813096 | 0.3999712 | 1 |
| 320_162 | A_23_P218608 | NM_015904 | EIF5B | 0.132047493 | 0.42626363 | 0.820625513 | 1 | -0.263284292 | 0.048936167 | 0.230776 | 1 |
| 243_113 | A_23_P218608 | NM_015904 | EIF5B | 0.165874702 | 0.302082608 | 0.736931323 | 1 | -0.218720034 | 0.087340664 | 0.3184516 | 1 |
| 78_108 | A_23_P218608 | NM_015904 | EIF5B | 0.189304472 | 0.159113068 | 0.597775747 | 1 | -0.162347982 | 0.208947489 | 0.4935701 | 1 |
| 131_99 | A_23_P218608 | NM_015904 | EIF5B | 0.214545885 | 0.156413519 | 0.595754329 | 1 | -0.230525207 | 0.089197901 | 0.3221608 | 1 |
| 184_104 | A_23_P218608 | NM_015904 | EIF5B | 0.290032723 | 0.093702819 | 0.496537113 | 1 | -0.254465143 | 0.135594211 | 0.3996405 | 1 |
| 1_55 | A_23_P210939 | NM_181468 | EIF6 | 0.461654296 | 0.021590544 | 0.308342332 | 1 | -0.551219708 | 0.021641239 | 0.143609 | 1 |
| 192_67 | A_23_P38254 | NM_018127 | ELAC2 | 0.328409282 | 0.021394438 | 0.308342332 | 1 | 0.07703882 | 0.680414756 | 0.8666619 | 1 |
| 312_9 | A_33_P3278068 | NM_001145353 | ELF1 | 0.168654756 | 0.332572342 | 0.76120935 | 1 | -0.311534412 | 0.011557891 | 0.0998759 | 1 |
| 151_146 | A_24_P340066 | NM_001421 | ELF4 | -0.058015435 | 0.68441843 | 0.921231232 | 1 | 0.392389412 | 0.006391557 | 0.0705614 | 1 |
| 185_132 | A_33_P3210848 | NM_001128636 | ELFN1 | -0.029655846 | 0.841135454 | 0.963184063 | 1 | 0.301792356 | 0.041023401 | 0.2093422 | 1 |
| 202_155 | A_23_P58506 | NM_012081 | ELL2 | 0.074986263 | 0.625964707 | 0.900094215 | 1 | -1.083350754 | 5.75543E-06 | 0.0013178 | 0.06473 |
| 193_44 | A_24_P322635 | NM_182764 | ELMO2 | -0.130463859 | 0.296116292 | 0.732735576 | 1 | 0.179076099 | 0.172132797 | 0.450018 | 1 |
| 313_41 | A_23_P7282 | NM_153702 | ELMOD2 | -0.348844489 | 0.025107842 | 0.320948304 | 1 | 0.123534998 | 0.426467956 | 0.7002383 | 1 |
| 234_106 | A_23_P154256 | NM_032213 | ELMOD3 | -0.101633955 | 0.409317694 | 0.810123176 | 1 | 0.030325012 | 0.808247184 | 0.9252271 | 1 |
| 255_27 | A_33_P3235204 | NM_032213 | ELMOD3 | -0.005528652 | 0.971684058 | 0.994619938 | 1 | -0.118062946 | 0.508711839 | 0.7590186 | 1 |
| 23_12 | A_23_P156497 | NM_021814 | ELOVL5 | 0.091529854 | 0.490391801 | 0.846768664 | 1 | -0.13537156 | 0.378795915 | 0.6609916 | 1 |
| 327_81 | A_33_P6579294 | ENST00000540766 | ELP2 | -0.059665372 | 0.697021728 | 0.925106933 | 1 | 0.260154053 | 0.319297814 | 0.6059183 | 1 |
| 111_25 | A_23_P78438 | NM_018255 | ELP2 | 0.061763759 | 0.722315145 | 0.935829302 | 1 | 0.237017486 | 0.1015649 | 0.3464318 | 1 |
| 114_39 | A_23_P43034 | NM_018091 | ELP3 | 0.124405987 | 0.340662672 | 0.765673924 | 1 | 0.432115895 | 0.00277401 | 0.041991 | 1 |
| 102_131 | A_33_P3394494 | NM_203415 | ELP5 | 0.048127501 | 0.713401797 | 0.93206229 | 1 | 0.005114309 | 0.966114851 | 0.9877187 | 1 |
| 320_24 | A_33_P3348313 | NM_001031703 | ELP6 | 0.043769121 | 0.748339681 | 0.941597731 | 1 | -0.186085655 | 0.124373432 | 0.3833798 | 1 |
| 232_124 | A_23_P212310 | NM_001031703 | ELP6 | 0.072798342 | 0.566536069 | 0.880639801 | 1 | -0.235493842 | 0.04492976 | 0.2193011 | 1 |
| 359_109 | A_23_P208674 | NM_175063 | EMC10 | 0.318256932 | 0.057201917 | 0.423633819 | 1 | -0.163938709 | 0.176849951 | 0.4552601 | 1 |
| 156_1 | A_23_P60002 | NM_014673 | EMC2 | 0.023442891 | 0.876380957 | 0.971233905 | 1 | 0.169450983 | 0.227438771 | 0.5154415 | 1 |
| 264_8 | A_23_P106481 | NM_016454 | EMC4 | 0.037928239 | 0.801111536 | 0.952164507 | 1 | -0.219384137 | 0.248471988 | 0.5355889 | 1 |
| 285_129 | A_24_P100673 | NM_016454 | EMC4 | 0.226865514 | 0.117156099 | 0.53981859 | 1 | 0.084346851 | 0.476604122 | 0.7358085 | 1 |
| 3_59 | A_23_P78134 | NM_001014764 | EMC6 | 0.412594127 | 0.01276153 | 0.265846757 | 1 | -0.078992244 | 0.618353912 | 0.8269473 | 1 |
| 275_67 | A_23_P88680 | NM_020154 | EMC7 | 0.079373749 | 0.539558113 | 0.869429199 | 1 | 0.117729537 | 0.300776986 | 0.5916122 | 1 |
| 245_45 | A_23_P106859 | NM_006067 | EMC8 | 0.078948654 | 0.687979531 | 0.922029409 | 1 | 0.171187678 | 0.130813369 | 0.3920957 | 1 |
| 32_12 | A_23_P106859 | NM_006067 | EMC8 | 0.132648784 | 0.551761504 | 0.87513796 | 1 | 0.126815416 | 0.286035743 | 0.5772057 | 1 |
| 37_32 | A_23_P106859 | NM_006067 | EMC8 | 0.165986382 | 0.384187092 | 0.79324867 | 1 | 0.245624645 | 0.045951554 | 0.2218099 | 1 |
| 5_7 | A_23_P106859 | NM_006067 | EMC8 | 0.18858726 | 0.387688544 | 0.795891784 | 1 | 0.119941286 | 0.345519279 | 0.6319817 | 1 |
| 301_73 | A_23_P106859 | NM_006067 | EMC8 | 0.222230294 | 0.278658271 | 0.719094153 | 1 | 0.223760339 | 0.066977778 | 0.2754293 | 1 |
| 147_145 | A_23_P106859 | NM_006067 | EMC8 | 0.242224975 | 0.248046504 | 0.693369094 | 1 | 0.085181628 | 0.506455789 | 0.7577939 | 1 |
| 37_50 | A_23_P106859 | NM_006067 | EMC8 | 0.259612171 | 0.145953482 | 0.58060515 | 1 | 0.259812392 | 0.04774961 | 0.2268983 | 1 |
| 301_149 | A_23_P106859 | NM_006067 | EMC8 | 0.288108956 | 0.205929434 | 0.653112569 | 1 | 0.188424302 | 0.15683915 | 0.4277328 | 1 |
| 122_63 | A_23_P106859 | NM_006067 | EMC8 | 0.290226366 | 0.093788392 | 0.496736838 | 1 | 0.237245962 | 0.078835381 | 0.3024084 | 1 |
| 204_61 | A_23_P106859 | NM_006067 | EMC8 | 0.486356226 | 0.017832404 | 0.296237697 | 1 | 0.197265355 | 0.132932747 | 0.3955205 | 1 |
| 266_43 | A_23_P88234 | NM_016049 | EMC9 | -0.358044211 | 0.01265419 | 0.265846757 | 1 | -0.143515105 | 0.239722372 | 0.5272111 | 1 |
| 303_37 | A_23_P85171 | NM_000117 | EMD | -0.018292997 | 0.892835542 | 0.975035641 | 1 | 0.338566126 | 0.027163937 | 0.1654999 | 1 |
| 126_121 | A_23_P368225 | NM_152463 | EME1 | -0.025338575 | 0.865157738 | 0.968067563 | 1 | 0.416567282 | 0.010562653 | 0.0946149 | 1 |
| 40_59 | A_24_P253215 | NM_006331 | EMG1 | 0.500110623 | 0.006751272 | 0.224098097 | 1 | -0.337160113 | 0.063756083 | 0.2667107 | 1 |
| 113_123 | A_22_P00017108 | NM_006331 | EMG1 | 0.54072033 | 0.002087355 | 0.144478193 | 1 | -0.420254853 | 0.028187519 | 0.1683617 | 1 |
| 95_52 | A_23_P27315 | NM_032048 | EMILIN2 | -0.001832569 | 0.98885783 | 0.99652193 | 1 | 0.145384509 | 0.280295558 | 0.5712948 | 1 |
| 367_87 | A_23_P143127 | ENST00000318522 | EML4 | -0.208289398 | 0.226595471 | 0.67162677 | 1 | 0.211554041 | 0.101802715 | 0.346647 | 1 |
| 109_150 | A_24_P273413 | ENST00000409040 | EML4 | -0.169311263 | 0.334989827 | 0.762359625 | 1 | -0.005020577 | 0.971667985 | 0.9898074 | 1 |
| 148_138 | A_23_P76488 | NM_001423 | EMP1 | 0.409611481 | 0.019961991 | 0.306116626 | 1 | 1.140880311 | 6.50877E-05 | 0.0043316 | 0.73204 |
| 202_33 | A_23_P106682 | NM_001424 | EMP2 | -0.477253751 | 0.003536627 | 0.17583436 | 1 | 0.103665817 | 0.391975486 | 0.6724448 | 1 |
| 337_111 | A_23_P304682 | NM_001424 | EMP2 | -0.451333024 | 0.017027695 | 0.29259326 | 1 | -0.079056652 | 0.486013848 | 0.7429928 | 1 |
| 131_115 | A_23_P119362 | NM_001425 | EMP3 | 0.183471405 | 0.241837883 | 0.687691199 | 1 | -0.101594831 | 0.372856005 | 0.6556459 | 1 |
| 7_62 | A_23_P315122 | NM_004097 | EMX1 | -0.03415612 | 0.775786976 | 0.946564916 | 1 | 0.123246749 | 0.386096555 | 0.6669328 | 1 |
| 15_34 | A_23_P134433 | NM_001427 | EN2 | 0.21764286 | 0.325746955 | 0.756433359 | 1 | 0.134862107 | 0.386804282 | 0.6677495 | 1 |
| 86_88 | A_23_P51397 | NM_001008493 | ENAH | -0.119469398 | 0.355690193 | 0.774004626 | 1 | -0.334749254 | 0.017680688 | 0.1287444 | 1 |
| 169_102 | A_23_P83266 | NM_004435 | ENDOG | 0.176760422 | 0.288863968 | 0.727382494 | 1 | -0.374014817 | 0.055010704 | 0.2472708 | 1 |
| 96_41 | A_23_P14975 | NM_032140 | ENKD1 | -0.074827986 | 0.590707927 | 0.88758105 | 1 | -0.25264672 | 0.061508338 | 0.2621388 | 1 |
| 82_91 | A_23_P137391 | NM_001428 | ENO1 | 0.3632384 | 0.078452133 | 0.466225857 | 1 | -0.282122615 | 0.377288841 | 0.6592151 | 1 |
| 303_140 | A_23_P130149 | NM_001976 | ENO3 | -0.216799242 | 0.112182285 | 0.530566305 | 1 | -0.296318405 | 0.061159771 | 0.2610489 | 1 |
| 243_7 | A_23_P121806 | NM_021204 | ENOPH1 | 0.016638509 | 0.902759357 | 0.976914095 | 1 | -0.227601211 | 0.197816304 | 0.4807347 | 1 |
| 60_1 | A_23_P121806 | NM_021204 | ENOPH1 | 0.093571344 | 0.510720689 | 0.856969619 | 1 | -0.216222128 | 0.108081356 | 0.3567922 | 1 |
| 24_17 | A_23_P121806 | NM_021204 | ENOPH1 | 0.172867821 | 0.193934528 | 0.639380357 | 1 | -0.16365582 | 0.314203823 | 0.6015453 | 1 |
| 305_49 | A_23_P121806 | NM_021204 | ENOPH1 | 0.180451487 | 0.157237091 | 0.595969916 | 1 | -0.191611713 | 0.238868952 | 0.5264594 | 1 |
| 298_90 | A_23_P121806 | NM_021204 | ENOPH1 | 0.204855355 | 0.151285283 | 0.588804256 | 1 | -0.171126152 | 0.265955273 | 0.5566057 | 1 |
| 145_28 | A_23_P121806 | NM_021204 | ENOPH1 | 0.228491151 | 0.10353192 | 0.515838795 | 1 | -0.0938003 | 0.468559917 | 0.7297579 | 1 |
| 132_143 | A_23_P121806 | NM_021204 | ENOPH1 | 0.233599424 | 0.142855566 | 0.576629059 | 1 | -0.197671164 | 0.182275955 | 0.4612102 | 1 |
| 45_139 | A_23_P121806 | NM_021204 | ENOPH1 | 0.265111794 | 0.073827064 | 0.459796162 | 1 | -0.319139732 | 0.050609306 | 0.2349166 | 1 |
| 1_112 | A_23_P121806 | NM_021204 | ENOPH1 | 0.280125057 | 0.055484547 | 0.422185511 | 1 | -0.255642741 | 0.128878873 | 0.3900476 | 1 |
| 143_63 | A_23_P121806 | NM_021204 | ENOPH1 | 0.356158816 | 0.027326332 | 0.331563124 | 1 | -0.222460453 | 0.137612011 | 0.4024239 | 1 |
| 185_8 | A_33_P3261902 | NM_001126123 | ENOSF1 | -0.068337443 | 0.658639589 | 0.911390964 | 1 | -0.009784856 | 0.947059781 | 0.9813523 | 1 |
| 236_106 | A_23_P258251 | NM_182314 | ENOX2 | 0.028836284 | 0.823219369 | 0.957894143 | 1 | 0.500435775 | 0.005743829 | 0.0663253 | 1 |
| 18_56 | A_23_P70318 | NM_014936 | ENPP4 | 0.080551005 | 0.565970748 | 0.880639801 | 1 | 0.218478977 | 0.113791595 | 0.3660795 | 1 |
| 131_39 | A_33_P3377209 | NM_207168 | ENSA | -0.008759771 | 0.943331127 | 0.98929578 | 1 | -0.260661155 | 0.072427365 | 0.2872322 | 1 |
| 282_59 | A_23_P301340 | NM_144679 | ENTHD2 | 0.013867258 | 0.93530127 | 0.986637903 | 1 | 0.093073052 | 0.457379415 | 0.7207865 | 1 |
| 241_70 | A_33_P3262580 | ENST00000371206 | ENTPD1 | 0.04041275 | 0.789832628 | 0.94877096 | 1 | 0.083994761 | 0.508914047 | 0.7592196 | 1 |
| 142_13 | A_23_P79999 | NM_001247 | ENTPD6 | 0.025536846 | 0.845566391 | 0.964393762 | 1 | -0.313357767 | 0.026600852 | 0.1632186 | 1 |
| 324_41 | A_23_P82748 | NM_020189 | ENY2 | -0.00449641 | 0.973639102 | 0.994783177 | 1 | 0.021680583 | 0.888584167 | 0.9612584 | 1 |
| 60_45 | A_23_P154806 | NM_012156 | EPB41L1 | -1.312184961 | 4.59466E-05 | 0.03290912 | 0.559722 | 0.879099299 | 0.000234258 | 0.0087242 | 1 |
| 183_16 | A_23_P58538 | NR_015370 | EPB41L4A-AS1 | -0.199927212 | 0.26816167 | 0.711309285 | 1 | 0.266573094 | 0.065714527 | 0.2721249 | 1 |
| 202_14 | A_33_P3399755 | ENST00000570199 | EPB42 | 0.12613985 | 0.439087038 | 0.827404459 | 1 | 0.034699646 | 0.805709923 | 0.9240154 | 1 |
| 15_64 | A_23_P359738 | NM_015630 | EPC2 | 0.006769307 | 0.959746207 | 0.992684122 | 1 | 0.140059713 | 0.260183152 | 0.5497426 | 1 |
| 170_17 | A_23_P91081 | NM_002354 | EPCAM | 0.450371172 | 0.053988489 | 0.417174905 | 1 | -0.249397357 | 0.066001012 | 0.272802 | 1 |
| 340_105 | A_24_P166613 | NM_017549 | EPDR1 | 0.163222078 | 0.249380111 | 0.694615167 | 1 | -0.209705351 | 0.104850931 | 0.3515976 | 1 |
| 92_125 | A_33_P3370787 | NM_004442 | EPHB2 | -0.013994612 | 0.919491474 | 0.981666418 | 1 | -0.721478799 | 0.002259382 | 0.03625 | 1 |
| 144_5 | A_33_P3216532 | NM_004444 | EPHB4 | 0.516326545 | 0.085995274 | 0.479590373 | 1 | 0.204177363 | 0.699039514 | 0.874605 | 1 |
| 357_85 | A_23_P119593 | NM_024794 | EPHX3 | 0.116831798 | 0.547892224 | 0.872939551 | 1 | -0.066544801 | 0.697121215 | 0.8739634 | 1 |
| 144_37 | A_33_P3370284 | NM_001130071 | EPN1 | -0.189610918 | 0.147918957 | 0.584668633 | 1 | 0.069169398 | 0.5800529 | 0.8071081 | 1 |
| 295_139 | A_23_P89310 | NM_014964 | EPN2 | -0.401465802 | 0.006728429 | 0.224098097 | 1 | 0.093854007 | 0.408667807 | 0.685198 | 1 |
| 179_40 | A_23_P97632 | NM_004446 | EPRS | 0.169796528 | 0.254565251 | 0.698225406 | 1 | -0.028100286 | 0.880535032 | 0.9586078 | 1 |
| 40_23 | A_33_P3322328 | NM_001981 | EPS15 | -0.139295503 | 0.390826247 | 0.796961054 | 1 | 0.061425292 | 0.672268335 | 0.8618742 | 1 |
| 332_148 | A_33_P3350828 | NM_021235 | EPS15L1 | 0.048599506 | 0.70136893 | 0.925829227 | 1 | -0.0195292 | 0.855424151 | 0.9477644 | 1 |
| 36_137 | A_33_P3220698 | NM_133180 | EPS8L1 | 0.101628023 | 0.57150021 | 0.882279506 | 1 | 0.60427553 | 0.002116659 | 0.0346968 | 1 |
| 46_30 | A_23_P147822 | NM_022772 | EPS8L2 | -0.278482992 | 0.061701911 | 0.431984295 | 1 | 0.181195253 | 0.148895418 | 0.41729 | 1 |
| 212_131 | A_24_P940803 | NM_033505 | EPT1 | 0.131758744 | 0.301630797 | 0.736808978 | 1 | -0.204690018 | 0.078361765 | 0.3015172 | 1 |
| 360_59 | A_23_P71981 | NM_005702 | ERAL1 | 0.151515326 | 0.262631908 | 0.705020251 | 1 | -0.17757105 | 0.155738834 | 0.4257644 | 1 |
| 325_17 | A_23_P89249 | NM_001005862 | ERBB2 | -0.513561974 | 0.010470657 | 0.253442612 | 1 | 0.220746086 | 0.17212194 | 0.450018 | 1 |
| 318_42 | A_23_P89249 | NM_001005862 | ERBB2 | -0.475764793 | 0.016637408 | 0.29132154 | 1 | 0.234408413 | 0.143763514 | 0.4111132 | 1 |
| 115_3 | A_23_P89249 | NM_001005862 | ERBB2 | -0.465594972 | 0.023392587 | 0.315579733 | 1 | 0.318387993 | 0.088208403 | 0.3204392 | 1 |
| 125_9 | A_23_P89249 | NM_001005862 | ERBB2 | -0.445854815 | 0.029911522 | 0.33959195 | 1 | 0.268988008 | 0.151922065 | 0.4205074 | 1 |
| 373_69 | A_23_P89249 | NM_001005862 | ERBB2 | -0.431572973 | 0.028727589 | 0.33575246 | 1 | 0.203581216 | 0.260072363 | 0.5496995 | 1 |
| 39_44 | A_23_P89249 | NM_001005862 | ERBB2 | -0.409895843 | 0.015889244 | 0.28586509 | 1 | 0.241550474 | 0.116275041 | 0.3698375 | 1 |
| 342_1 | A_23_P89249 | NM_001005862 | ERBB2 | -0.395158023 | 0.029436714 | 0.338697583 | 1 | 0.221932055 | 0.212246454 | 0.4973251 | 1 |
| 70_63 | A_23_P89249 | NM_001005862 | ERBB2 | -0.375404878 | 0.020839507 | 0.306261998 | 1 | 0.329991527 | 0.033504417 | 0.1871953 | 1 |
| 185_129 | A_23_P89249 | NM_001005862 | ERBB2 | -0.289776074 | 0.088927945 | 0.486413374 | 1 | 0.120277743 | 0.428165265 | 0.7019788 | 1 |
| 7_136 | A_23_P89249 | NM_001005862 | ERBB2 | -0.21045129 | 0.304069444 | 0.738634903 | 1 | 0.18845619 | 0.248496616 | 0.5355889 | 1 |
| 268_65 | A_23_P30175 | NM_018695 | ERBIN | -0.040836383 | 0.838177469 | 0.962281738 | 1 | -0.175125651 | 0.143751244 | 0.4111132 | 1 |
| 17_8 | A_33_P3228128 | NM_202001 | ERCC1 | -0.011270603 | 0.929613321 | 0.985082992 | 1 | 0.066552712 | 0.574625325 | 0.803906 | 1 |
| 28_74 | A_33_P3365037 | NM_202001 | ERCC1 | 0.175737841 | 0.267287807 | 0.710938878 | 1 | 0.061232055 | 0.61359842 | 0.8240856 | 1 |
| 336_7 | A_23_P117225 | NM_000123 | ERCC5 | -0.188385891 | 0.242963469 | 0.688962053 | 1 | -0.139506155 | 0.349673312 | 0.63487 | 1 |
| 218_14 | A_23_P117225 | NM_000123 | ERCC5 | -0.175289848 | 0.201621118 | 0.648060281 | 1 | -0.096426343 | 0.423039942 | 0.6985354 | 1 |
| 87_68 | A_23_P117225 | NM_000123 | ERCC5 | -0.120411337 | 0.421196211 | 0.817635849 | 1 | -0.136297153 | 0.396096957 | 0.6759357 | 1 |
| 245_95 | A_23_P117225 | NM_000123 | ERCC5 | -0.087368652 | 0.623866045 | 0.899294303 | 1 | -0.162542974 | 0.22907947 | 0.5166346 | 1 |
| 75_69 | A_23_P117225 | NM_000123 | ERCC5 | -0.084388751 | 0.567340238 | 0.881043427 | 1 | -0.263805973 | 0.077854174 | 0.2998719 | 1 |
| 379_49 | A_23_P117225 | NM_000123 | ERCC5 | -0.07153357 | 0.594941527 | 0.889988626 | 1 | -0.121114309 | 0.459572552 | 0.722045 | 1 |
| 28_118 | A_23_P117225 | NM_000123 | ERCC5 | 0.006995289 | 0.95951134 | 0.992682116 | 1 | -0.25220192 | 0.074939963 | 0.2927152 | 1 |
| 13_155 | A_23_P117225 | NM_000123 | ERCC5 | 0.034640371 | 0.812818267 | 0.955007122 | 1 | -0.203112069 | 0.216212771 | 0.5028916 | 1 |
| 202_118 | A_23_P117225 | NM_000123 | ERCC5 | 0.046789734 | 0.731645832 | 0.937415492 | 1 | -0.285174814 | 0.043427035 | 0.2157349 | 1 |
| 141_68 | A_23_P117225 | NM_000123 | ERCC5 | 0.079686837 | 0.605376865 | 0.894904051 | 1 | -0.09684735 | 0.503892499 | 0.7571515 | 1 |
| 115_161 | A_23_P333218 | NM_001031711 | ERGIC1 | -0.176538921 | 0.175745042 | 0.616271187 | 1 | 0.333587198 | 0.030652812 | 0.1780745 | 1 |
| 189_30 | A_23_P139509 | NM_016570 | ERGIC2 | -0.047616222 | 0.713687092 | 0.932151097 | 1 | 0.031671239 | 0.799578107 | 0.9212674 | 1 |
| 184_34 | A_23_P139509 | NM_016570 | ERGIC2 | 0.009257606 | 0.950262665 | 0.99027625 | 1 | 0.040144743 | 0.752819269 | 0.9010572 | 1 |
| 83_116 | A_23_P139509 | NM_016570 | ERGIC2 | 0.063426748 | 0.623432473 | 0.899250294 | 1 | 0.004321339 | 0.971533679 | 0.9898074 | 1 |
| 72_81 | A_23_P139509 | NM_016570 | ERGIC2 | 0.102160339 | 0.472627441 | 0.838773065 | 1 | 0.035598037 | 0.783710416 | 0.9143719 | 1 |
| 146_97 | A_23_P139509 | NM_016570 | ERGIC2 | 0.155953605 | 0.271694134 | 0.714790846 | 1 | 0.086323308 | 0.544614412 | 0.7838236 | 1 |
| 166_94 | A_23_P139509 | NM_016570 | ERGIC2 | 0.168230703 | 0.25823503 | 0.700376544 | 1 | 0.014043421 | 0.912466991 | 0.9694337 | 1 |
| 191_148 | A_23_P139509 | NM_016570 | ERGIC2 | 0.215190414 | 0.135812683 | 0.566743973 | 1 | -0.070689591 | 0.535853361 | 0.7766421 | 1 |
| 102_55 | A_23_P128734 | NM_004450 | ERH | -0.028220335 | 0.858528309 | 0.966062429 | 1 | 0.202571138 | 0.18635884 | 0.4663947 | 1 |
| 144_137 | A_33_P3315504 | NM_153332 | ERI1 | 0.239301071 | 0.133579407 | 0.563559912 | 1 | -0.15966586 | 0.218888757 | 0.5052005 | 1 |
| 175_111 | A_22_P00006418 | NR_073397 | ERICH1-AS1 | 0.009435821 | 0.939774739 | 0.988335297 | 1 | -0.065724173 | 0.637121016 | 0.8403542 | 1 |
| 222_99 | A_32_P197340 | NM_001290030 | ERICH2 | -0.285099079 | 0.060037616 | 0.428867769 | 1 | 0.6999306 | 0.000362883 | 0.0114872 | 1 |
| 361_88 | A_22_P00000261 | NM_001290031 | ERICH2 | 0.034832917 | 0.855292169 | 0.965512529 | 1 | 0.015759362 | 0.920670299 | 0.9716411 | 1 |
| 333_45 | A_33_P3325634 | NM_001130514 | ERICH4 | -0.057620099 | 0.656753051 | 0.910923281 | 1 | 0.050481772 | 0.674221382 | 0.8635654 | 1 |
| 116_69 | A_22_P00018539 | NR_121674 | ERICH6-AS1 | -0.024619712 | 0.843034595 | 0.963852411 | 1 | 0.443050035 | 0.002364109 | 0.0372919 | 1 |
| 93_68 | A_33_P3325018 | NM_015701 | ERLEC1 | -0.285114537 | 0.066734934 | 0.446719314 | 1 | 0.232686709 | 0.073162281 | 0.2890257 | 1 |
| 186_100 | A_33_P3325023 | NM_015701 | ERLEC1 | 0.12076955 | 0.373277431 | 0.787188577 | 1 | 0.234573735 | 0.116983845 | 0.3709903 | 1 |
| 60_113 | A_32_P129527 | NM_018341 | ERMARD | 0.011225288 | 0.946813829 | 0.99002169 | 1 | -0.488886136 | 0.019478614 | 0.1364109 | 1 |
| 335_116 | A_22_P00005863 | ENST00000433197 | ERN1 | 0.123246417 | 0.377398927 | 0.789702212 | 1 | 0.027813031 | 0.819293445 | 0.9307067 | 1 |
| 286_49 | A_23_P106145 | ENST00000555069 | ERO1A | 0.100815451 | 0.492275551 | 0.848612902 | 1 | 0.019125874 | 0.899911105 | 0.9647888 | 1 |
| 155_13 | A_23_P106145 | ENST00000555069 | ERO1A | 0.257152587 | 0.084639132 | 0.478632531 | 1 | -0.117677545 | 0.428728848 | 0.702194 | 1 |
| 53_44 | A_23_P106145 | ENST00000555069 | ERO1A | 0.348305506 | 0.022394644 | 0.312907932 | 1 | -0.005586525 | 0.974805959 | 0.9908408 | 1 |
| 336_149 | A_23_P106145 | ENST00000555069 | ERO1A | 0.38605082 | 0.048781144 | 0.403099681 | 1 | -0.089767191 | 0.50701125 | 0.7580871 | 1 |
| 130_147 | A_23_P106145 | ENST00000555069 | ERO1A | 0.399922471 | 0.023644115 | 0.316794251 | 1 | 0.055787258 | 0.721282453 | 0.8860556 | 1 |
| 327_115 | A_23_P106145 | ENST00000555069 | ERO1A | 0.420544019 | 0.020379878 | 0.306188729 | 1 | 0.008763579 | 0.955922657 | 0.984638 | 1 |
| 108_154 | A_23_P106145 | ENST00000555069 | ERO1A | 0.436059895 | 0.035039537 | 0.360884378 | 1 | -0.163023396 | 0.237382296 | 0.5257658 | 1 |
| 60_82 | A_23_P106145 | ENST00000555069 | ERO1A | 0.465317242 | 0.006132184 | 0.2165283 | 1 | -0.043317842 | 0.756014224 | 0.9022593 | 1 |
| 3_91 | A_23_P106145 | ENST00000555069 | ERO1A | 0.522893388 | 0.00297077 | 0.162286645 | 1 | 0.088525039 | 0.621379159 | 0.8289574 | 1 |
| 154_96 | A_23_P106145 | ENST00000555069 | ERO1A | 0.543246001 | 0.002728331 | 0.154588512 | 1 | -0.131606391 | 0.44130819 | 0.7115701 | 1 |
| 199_87 | A_23_P139929 | NM_006817 | ERP29 | 0.100111361 | 0.71369014 | 0.932151097 | 1 | 0.033436145 | 0.845451815 | 0.9425849 | 1 |
| 331_88 | A_21_P0014820 | ENST00000413518 | ERVK3-1 | 0.225446405 | 0.129462735 | 0.559062404 | 1 | -0.317626422 | 0.014681252 | 0.1157113 | 1 |
| 230_39 | A_23_P87964 | NM_001984 | ESD | 0.161055626 | 0.220396483 | 0.66688275 | 1 | -0.484391055 | 0.000890789 | 0.0210336 | 1 |
| 10_89 | A_33_P3354955 | NM_016649 | ESF1 | -0.064536451 | 0.600872093 | 0.892899691 | 1 | 0.09093512 | 0.452995647 | 0.7200368 | 1 |
| 156_122 | A_23_P32707 | NM_012291 | ESPL1 | 0.043603269 | 0.745331462 | 0.941243599 | 1 | -0.572984092 | 0.034122696 | 0.1894264 | 1 |
| 308_41 | A_23_P100220 | NM_024939 | ESRP2 | -0.260902483 | 0.073256511 | 0.459292518 | 1 | -0.018482926 | 0.904363472 | 0.966562 | 1 |
| 28_58 | A_23_P100220 | NM_024939 | ESRP2 | -0.208765716 | 0.166779378 | 0.60666061 | 1 | -0.068071675 | 0.64685222 | 0.8464394 | 1 |
| 370_145 | A_23_P100220 | NM_024939 | ESRP2 | -0.178586525 | 0.185982068 | 0.629342652 | 1 | -0.108729513 | 0.457222632 | 0.7207865 | 1 |
| 248_48 | A_23_P100220 | NM_024939 | ESRP2 | -0.154631354 | 0.235930395 | 0.681859997 | 1 | -0.065225064 | 0.649782653 | 0.8479064 | 1 |
| 298_58 | A_23_P100220 | NM_024939 | ESRP2 | -0.150370526 | 0.314692658 | 0.745268067 | 1 | -0.032117322 | 0.844181177 | 0.9415416 | 1 |
| 348_41 | A_23_P100220 | NM_024939 | ESRP2 | -0.142779185 | 0.303934865 | 0.738634903 | 1 | -0.02152414 | 0.909471092 | 0.9680883 | 1 |
| 10_111 | A_23_P100220 | NM_024939 | ESRP2 | -0.116748319 | 0.368171721 | 0.782211132 | 1 | -0.09472594 | 0.594118207 | 0.8154033 | 1 |
| 80_144 | A_23_P100220 | NM_024939 | ESRP2 | -0.081784926 | 0.574006138 | 0.882765809 | 1 | -0.122046625 | 0.351231513 | 0.6362217 | 1 |
| 90_86 | A_23_P100220 | NM_024939 | ESRP2 | -0.044189146 | 0.765110238 | 0.944948837 | 1 | -0.064923173 | 0.662216988 | 0.8562637 | 1 |
| 144_162 | A_23_P100220 | NM_024939 | ESRP2 | -0.002041285 | 0.988543631 | 0.99652193 | 1 | -0.070842245 | 0.612544262 | 0.8236831 | 1 |
| 332_138 | A_23_P1585 | NM_004451 | ESRRA | 0.045219928 | 0.774533282 | 0.946321166 | 1 | -0.152860582 | 0.180714981 | 0.4591655 | 1 |
| 308_9 | A_24_P132470 | NM_020728 | ESYT2 | 0.170011074 | 0.208689855 | 0.655712085 | 1 | -0.242082929 | 0.041021263 | 0.2093422 | 1 |
| 314_128 | A_23_P107653 | NM_001014763 | ETFB | -0.458406583 | 0.022620914 | 0.313438642 | 1 | 0.091951426 | 0.538713601 | 0.7793815 | 1 |
| 4_81 | A_23_P61447 | NM_004453 | ETFDH | -0.19605996 | 0.133254943 | 0.563083669 | 1 | 0.137103155 | 0.326065598 | 0.6120838 | 1 |
| 47_50 | A_23_P142294 | NM_014297 | ETHE1 | 0.014353864 | 0.910297677 | 0.979010003 | 1 | -0.209614018 | 0.190708744 | 0.4723557 | 1 |
| 154_84 | A_33_P3351559 | NM_018638 | ETNK1 | 0.169057667 | 0.197754183 | 0.644449456 | 1 | 0.041211528 | 0.804610614 | 0.9231312 | 1 |
| 380_101 | A_33_P3351554 | NM_001297760 | ETNK2 | -0.521737687 | 0.011159258 | 0.256586468 | 1 | 0.137357653 | 0.324682789 | 0.6109806 | 1 |
| 223_129 | A_33_P3343106 | NM_014209 | ETV2 | -0.041720476 | 0.769859198 | 0.945351992 | 1 | -0.005976386 | 0.965861395 | 0.9876934 | 1 |
| 114_92 | A_33_P3250028 | NM_145245 | EVI5L | -0.118523417 | 0.402526936 | 0.805674814 | 1 | 0.169231189 | 0.282164979 | 0.5730425 | 1 |
| 256_18 | A_24_P104512 | NM_001988 | EVPL | 0.041157769 | 0.825665929 | 0.959235369 | 1 | 0.093304795 | 0.683082582 | 0.8680858 | 1 |
| 149_1 | A_22_P00013715 | ENST00000559914 | EWSAT1 | -0.069249834 | 0.614949592 | 0.898023966 | 1 | 0.208144715 | 0.076029043 | 0.2958228 | 1 |
| 295_148 | A_33_P3271126 | A_33_P3271126 | EXOC3L2 | -0.090511523 | 0.58298219 | 0.883876284 | 1 | 0.173548304 | 0.236430543 | 0.5244841 | 1 |
| 103_41 | A_23_P169576 | NM_019053 | EXOC6 | -0.254001123 | 0.058285382 | 0.42530091 | 1 | -0.103629827 | 0.41229469 | 0.688198 | 1 |
| 94_5 | A_33_P3390032 | NM_001145297 | EXOC7 | -0.382719483 | 0.026205601 | 0.326755269 | 1 | 0.103981162 | 0.640548066 | 0.8428974 | 1 |
| 354_151 | A_23_P89163 | NM_001013839 | EXOC7 | -0.065289686 | 0.651414435 | 0.908340249 | 1 | -0.189457813 | 0.105231454 | 0.352034 | 1 |
| 131_90 | A_23_P35591 | NM_016046 | EXOSC1 | 0.254538394 | 0.054585907 | 0.419272083 | 1 | 0.045703753 | 0.682825675 | 0.8679746 | 1 |
| 206_157 | A_23_P62868 | ENST00000490565 | EXOSC10 | 0.550087439 | 0.013124749 | 0.267459687 | 1 | -0.217760842 | 0.067951277 | 0.2771023 | 1 |
| 93_156 | A_33_P7718819 | NM_014285 | EXOSC2 | -0.005243217 | 0.969478878 | 0.994075478 | 1 | 0.047594823 | 0.705198098 | 0.8767812 | 1 |
| 179_139 | A_23_P216396 | NM_014285 | EXOSC2 | 0.064920714 | 0.619402222 | 0.89867047 | 1 | -0.058596295 | 0.670331112 | 0.8610213 | 1 |
| 106_139 | A_33_P3347132 | NM_014285 | EXOSC2 | 0.09672367 | 0.546420723 | 0.871862139 | 1 | -0.106731542 | 0.380991483 | 0.6631091 | 1 |
| 150_3 | A_23_P123905 | NM_016042 | EXOSC3 | 0.078570068 | 0.612375942 | 0.897709232 | 1 | -0.052457703 | 0.76447158 | 0.9061031 | 1 |
| 248_27 | A_23_P123905 | NM_016042 | EXOSC3 | 0.114105764 | 0.501181838 | 0.853119221 | 1 | -0.13566066 | 0.396241089 | 0.6759357 | 1 |
| 369_119 | A_23_P123905 | NM_016042 | EXOSC3 | 0.267886467 | 0.149372073 | 0.58620836 | 1 | -0.072114883 | 0.69471728 | 0.8728201 | 1 |
| 351_163 | A_23_P123905 | NM_016042 | EXOSC3 | 0.27320543 | 0.108174272 | 0.524570001 | 1 | -0.196057526 | 0.215269028 | 0.5015537 | 1 |
| 250_83 | A_23_P123905 | NM_016042 | EXOSC3 | 0.326329618 | 0.094403157 | 0.498453904 | 1 | -0.096669193 | 0.604470747 | 0.819588 | 1 |
| 377_148 | A_23_P123905 | NM_016042 | EXOSC3 | 0.353377858 | 0.055245155 | 0.422185511 | 1 | -0.090060709 | 0.593948585 | 0.8153472 | 1 |
| 58_138 | A_23_P123905 | NM_016042 | EXOSC3 | 0.361482631 | 0.040186179 | 0.378321505 | 1 | -0.207221159 | 0.136802469 | 0.4014134 | 1 |
| 342_96 | A_23_P123905 | NM_016042 | EXOSC3 | 0.378434538 | 0.043583278 | 0.390521637 | 1 | -0.121627582 | 0.476593047 | 0.7358085 | 1 |
| 164_140 | A_23_P123905 | NM_016042 | EXOSC3 | 0.407557136 | 0.042078348 | 0.38570236 | 1 | -0.084514768 | 0.588578707 | 0.8120393 | 1 |
| 290_112 | A_23_P123905 | NM_016042 | EXOSC3 | 0.421712122 | 0.031474112 | 0.345065816 | 1 | -0.165140764 | 0.312738927 | 0.6012606 | 1 |
| 10_151 | A_33_P3301410 | NM_019037 | EXOSC4 | 0.275048109 | 0.051699224 | 0.410026009 | 1 | -0.016980426 | 0.901454614 | 0.9651271 | 1 |
| 341_49 | A_23_P58102 | NM_015004 | EXOSC7 | 0.148536529 | 0.42014907 | 0.817246292 | 1 | -0.007964392 | 0.954300569 | 0.9841389 | 1 |
| 210_52 | A_24_P241276 | NM_181503 | EXOSC8 | 0.53682726 | 0.013653854 | 0.270772576 | 1 | -0.522795789 | 0.000612525 | 0.0165603 | 1 |
| 71_22 | A_23_P81121 | NM_005033 | EXOSC9 | -0.189851999 | 0.256545928 | 0.699039445 | 1 | -0.231503486 | 0.062502621 | 0.2646738 | 1 |
| 47_104 | A_23_P13183 | NM_000401 | EXT2 | 0.203671203 | 0.160034285 | 0.598752215 | 1 | -0.157189245 | 0.222450994 | 0.5104889 | 1 |
| 270_28 | A_33_P3291294 | NM_001440 | EXTL3 | -0.413622827 | 0.008081465 | 0.234464792 | 1 | 0.321901329 | 0.016474835 | 0.1231179 | 1 |
| 116_109 | A_33_P3276813 | NM_001142800 | EYS | 0.140260276 | 0.47819014 | 0.840908892 | 1 | -0.056695053 | 0.802754757 | 0.9228701 | 1 |
| 120_81 | A_33_P3252196 | NM_004456 | EZH2 | 0.322139309 | 0.027538538 | 0.332453123 | 1 | 0.127215585 | 0.334596836 | 0.6209473 | 1 |
| 363_144 | A_23_P19590 | NM_003379 | EZR | 0.175361193 | 0.332642665 | 0.76120935 | 1 | -0.077310197 | 0.483178644 | 0.7413351 | 1 |
| 29_24 | A_24_P319364 | NM_016946 | F11R | -0.704741041 | 0.000207392 | 0.053546208 | 1 | -0.111665388 | 0.382849888 | 0.6648005 | 1 |
| 136_159 | A_33_P3233871 | NM_000505 | F12 | -0.407220266 | 0.009125659 | 0.240105343 | 1 | 0.226616321 | 0.202396557 | 0.4851248 | 1 |
| 66_30 | A_23_P11262 | NM_012151 | F8A1 | -0.35754334 | 0.039026079 | 0.374228433 | 1 | 0.893296233 | 9.25412E-05 | 0.0053375 | 1 |
| 256_26 | A_23_P11262 | NM_012151 | F8A1 | -0.35119004 | 0.063126376 | 0.435697175 | 1 | 0.839034356 | 6.34286E-05 | 0.0042975 | 0.71338 |
| 72_50 | A_23_P11262 | NM_012151 | F8A1 | -0.232093516 | 0.164383939 | 0.604262266 | 1 | 0.992812094 | 3.52308E-05 | 0.00312 | 0.39624 |
| 112_44 | A_23_P11262 | NM_012151 | F8A1 | -0.185106291 | 0.20695922 | 0.654291494 | 1 | 0.849711901 | 7.88513E-05 | 0.0048198 | 0.88684 |
| 71_140 | A_23_P11262 | NM_012151 | F8A1 | -0.17548951 | 0.306756946 | 0.739887567 | 1 | 0.795833965 | 0.000132356 | 0.0064003 | 1 |
| 174_22 | A_23_P11262 | NM_012151 | F8A1 | -0.159684466 | 0.307863442 | 0.740336569 | 1 | 0.772104326 | 0.000103258 | 0.0057122 | 1 |
| 370_150 | A_23_P11262 | NM_012151 | F8A1 | -0.13779406 | 0.423864604 | 0.819346019 | 1 | 0.869915603 | 4.30934E-05 | 0.0034531 | 0.48467 |
| 319_76 | A_23_P11262 | NM_012151 | F8A1 | -0.133797907 | 0.397606398 | 0.801548559 | 1 | 0.950881489 | 9.88001E-06 | 0.0015122 | 0.11112 |
| 26_119 | A_23_P11262 | NM_012151 | F8A1 | -0.072287686 | 0.556587681 | 0.87635403 | 1 | 0.779401008 | 5.34447E-05 | 0.003878 | 0.60109 |
| 212_77 | A_23_P11262 | NM_012151 | F8A1 | -0.033169069 | 0.874193826 | 0.970677357 | 1 | 0.87222027 | 0.000177438 | 0.0072569 | 1 |
| 206_144 | A_33_P3242234 | NM_001007523 | F8A2 | -0.09405761 | 0.505466258 | 0.85467065 | 1 | 0.416694348 | 0.002803237 | 0.0423195 | 1 |
| 160_63 | A_32_P204676 | NM_001444 | FABP5 | 0.490873708 | 0.00331144 | 0.171659439 | 1 | 0.114289822 | 0.704008715 | 0.8766152 | 1 |
| 182_144 | A_24_P673063 | NM_001444 | FABP5 | 0.588151207 | 0.002512121 | 0.154236866 | 1 | 0.020711826 | 0.934430055 | 0.9765411 | 1 |
| 34_88 | A_23_P59877 | NM_001444 | FABP5 | 0.968452574 | 0.001094699 | 0.115389404 | 1 | 0.148756036 | 0.563496795 | 0.7964872 | 1 |
| 340_111 | A_23_P86917 | NM_003824 | FADD | 0.065556919 | 0.638562673 | 0.904531451 | 1 | -0.104579634 | 0.39541001 | 0.6755547 | 1 |
| 18_45 | A_24_P192994 | NM_013402 | FADS1 | 0.017713834 | 0.899942592 | 0.97618995 | 1 | 0.419478272 | 0.025278374 | 0.1590078 | 1 |
| 371_95 | A_23_P98580 | NM_004265 | FADS2 | -0.373961581 | 0.011398515 | 0.259951804 | 1 | 0.776648064 | 0.000218379 | 0.0082697 | 1 |
| 144_10 | A_23_P96853 | NM_007051 | FAF1 | 0.038086466 | 0.771619199 | 0.945351992 | 1 | -0.178828703 | 0.23019949 | 0.5182253 | 1 |
| 217_48 | A_32_P81357 | NM_016044 | FAHD2A | -0.028826816 | 0.828486224 | 0.959690637 | 1 | -0.289732557 | 0.05517812 | 0.247486 | 1 |
| 22_35 | A_23_P253932 | NM_001033030 | FAIM | 0.012921461 | 0.917695884 | 0.981346939 | 1 | -0.014771831 | 0.928016901 | 0.9738557 | 1 |
| 221_9 | A_23_P112531 | NM_001035254 | FAM102A | -0.53101985 | 0.004106668 | 0.188782732 | 1 | -0.049084843 | 0.688068921 | 0.8689581 | 1 |
| 138_12 | A_23_P112531 | NM_001035254 | FAM102A | -0.4795999 | 0.006347157 | 0.219275219 | 1 | -0.073820653 | 0.547543248 | 0.7858193 | 1 |
| 153_15 | A_23_P112531 | NM_001035254 | FAM102A | -0.463800416 | 0.011797315 | 0.26314611 | 1 | -0.049867503 | 0.699090503 | 0.874605 | 1 |
| 96_36 | A_23_P112531 | NM_001035254 | FAM102A | -0.461270981 | 0.020699791 | 0.306231219 | 1 | -0.097030222 | 0.428795962 | 0.702194 | 1 |
| 220_134 | A_23_P112531 | NM_001035254 | FAM102A | -0.377698942 | 0.031310579 | 0.344912707 | 1 | -0.144469322 | 0.228389556 | 0.5163118 | 1 |
| 13_70 | A_23_P112531 | NM_001035254 | FAM102A | -0.335380943 | 0.047145777 | 0.399787213 | 1 | -0.071877622 | 0.532355324 | 0.773466 | 1 |
| 334_63 | A_23_P112531 | NM_001035254 | FAM102A | -0.324989191 | 0.060967638 | 0.430408929 | 1 | -0.09916271 | 0.520664712 | 0.7668003 | 1 |
| 44_134 | A_23_P112531 | NM_001035254 | FAM102A | -0.317754723 | 0.122326161 | 0.545652616 | 1 | -0.149486067 | 0.260548539 | 0.5502451 | 1 |
| 17_148 | A_23_P112531 | NM_001035254 | FAM102A | -0.314378342 | 0.058953142 | 0.426618235 | 1 | -0.16095729 | 0.256588788 | 0.5457612 | 1 |
| 258_117 | A_23_P112531 | NM_001035254 | FAM102A | -0.199574849 | 0.183944623 | 0.626744723 | 1 | -0.182999994 | 0.238912487 | 0.5264594 | 1 |
| 34_44 | A_23_P65712 | NM_031452 | FAM103A1 | 0.23490398 | 0.096578113 | 0.500009516 | 1 | 0.020073689 | 0.876273726 | 0.9572584 | 1 |
| 40_128 | A_23_P78289 | NM_032837 | FAM104A | -0.098175219 | 0.510167054 | 0.856630606 | 1 | 0.064826757 | 0.658584045 | 0.8542377 | 1 |
| 90_113 | A_23_P386241 | NM_001042353 | FAM110A | -0.351685326 | 0.04287574 | 0.390076378 | 1 | 0.228229303 | 0.181290877 | 0.4597471 | 1 |
| 181_115 | A_33_P3270657 | NM_198947 | FAM111B | -0.105892184 | 0.487410691 | 0.846035145 | 1 | -0.32398243 | 0.026400185 | 0.1628369 | 1 |
| 293_161 | A_33_P3424861 | NM_001104595 | FAM118A | 0.118650808 | 0.506533106 | 0.855340657 | 1 | 0.482237441 | 0.129284025 | 0.3904558 | 1 |
| 233_123 | A_24_P941505 | NM_014612 | FAM120A | 0.137012654 | 0.32506443 | 0.755713128 | 1 | -0.280736131 | 0.043236255 | 0.2153579 | 1 |
| 149_88 | A_24_P138022 | NM_014612 | FAM120A | 0.242622483 | 0.099865815 | 0.507325002 | 1 | -0.112849566 | 0.393931645 | 0.6741782 | 1 |
| 134_88 | A_24_P832156 | NM_198841 | FAM120AOS | 0.070874638 | 0.583894768 | 0.884262315 | 1 | 0.373005348 | 0.030103048 | 0.1763026 | 1 |
| 195_104 | A_32_P67623 | NM_001300788 | FAM120C | 0.105173081 | 0.449456578 | 0.831477605 | 1 | -0.483562782 | 0.040223717 | 0.2066838 | 1 |
| 152_158 | A_33_P3303865 | NM_198456 | FAM120C | 0.109767512 | 0.464500921 | 0.836223448 | 1 | 0.145918461 | 0.250317026 | 0.5379406 | 1 |
| 188_2 | A_33_P3234118 | NM_001166599 | FAM122B | -0.283230469 | 0.039461006 | 0.375851431 | 1 | 0.42052447 | 0.015222162 | 0.1182102 | 1 |
| 252_66 | A_33_P3226167 | NM_022833 | FAM129B | 0.060315854 | 0.670369756 | 0.916686207 | 1 | -0.387571377 | 0.01941623 | 0.1362006 | 1 |
| 299_27 | A_24_P169073 | NM_182623 | FAM131C | 0.024062848 | 0.906126965 | 0.978062713 | 1 | 0.123914868 | 0.592483239 | 0.8147278 | 1 |
| 155_35 | A_24_P158065 | NM_001040057 | FAM133B | 0.070120415 | 0.570820143 | 0.882279506 | 1 | -0.080020745 | 0.503300403 | 0.7567923 | 1 |
| 341_23 | A_21_P0014809 | NM_032822 | FAM136A | -0.050973925 | 0.699282942 | 0.92523784 | 1 | 0.823568541 | 0.000123821 | 0.0062449 | 1 |
| 23_51 | A_23_P108641 | NM_032822 | FAM136A | 0.176201553 | 0.216579434 | 0.662917773 | 1 | 0.009561782 | 0.93699413 | 0.9778578 | 1 |
| 186_71 | A_22_P00009106 | NR_046848 | FAM155A-IT1 | -0.100226173 | 0.72271539 | 0.935844012 | 1 | 0.710168019 | 0.000276665 | 0.0097544 | 1 |
| 157_91 | A_23_P148519 | NM_014138 | FAM156A | 0.015952186 | 0.908288881 | 0.978414074 | 1 | -0.783154211 | 0.000887163 | 0.0210336 | 1 |
| 351_141 | A_23_P24796 | NM_032127 | FAM160A2 | -0.034549525 | 0.792350177 | 0.94877096 | 1 | -0.279804079 | 0.024591461 | 0.1564367 | 1 |
| 356_47 | A_23_P256542 | NM_014367 | FAM162A | -0.131921452 | 0.317592885 | 0.748484528 | 1 | 0.244585928 | 0.072650701 | 0.2878135 | 1 |
| 328_134 | A_33_P3295870 | NM_001001710 | FAM166A | -0.208242231 | 0.218637445 | 0.665527575 | 1 | 0.300604842 | 0.127310965 | 0.3883554 | 1 |
| 176_100 | A_23_P54728 | NM_023933 | FAM173A | 0.041008631 | 0.785816505 | 0.94877096 | 1 | -0.370158593 | 0.02307612 | 0.1503691 | 1 |
| 113_23 | A_23_P133279 | NM_199133 | FAM173B | 0.03317755 | 0.799647179 | 0.951548003 | 1 | -0.140538217 | 0.227247945 | 0.51519 | 1 |
| 220_106 | A_23_P30283 | ENST00000312637 | FAM174A | -0.07269745 | 0.598142637 | 0.89144727 | 1 | -0.147065119 | 0.277609689 | 0.5687994 | 1 |
| 201_68 | A_23_P100001 | NM_207446 | FAM174B | 0.344348982 | 0.041616708 | 0.383781023 | 1 | -0.35352387 | 0.012867148 | 0.1070391 | 1 |
| 286_163 | A_23_P253464 | NM_139076 | FAM175A | -0.106816343 | 0.466938311 | 0.83630364 | 1 | 0.160662972 | 0.226928432 | 0.5148774 | 1 |
| 99_5 | A_23_P322704 | NM_001079519 | FAM177A1 | -0.411860366 | 0.009036926 | 0.240080283 | 1 | -0.261901933 | 0.181532089 | 0.4600975 | 1 |
| 43_149 | A_33_P3287119 | NM_001122646 | FAM178B | 0.031752844 | 0.90550124 | 0.977909229 | 1 | -0.044242152 | 0.843802552 | 0.9414469 | 1 |
| 154_93 | A_23_P23983 | NM_024948 | FAM188A | -0.106141089 | 0.409262274 | 0.810123176 | 1 | 0.065535966 | 0.557283045 | 0.7923846 | 1 |
| 269_50 | A_23_P103690 | NM_006589 | FAM189B | -0.104194115 | 0.485785587 | 0.844801343 | 1 | -0.426729122 | 0.010566051 | 0.0946149 | 1 |
| 17_51 | A_33_P3359160 | NM_001267608 | FAM189B | -0.021535185 | 0.891028491 | 0.974300487 | 1 | -0.498847518 | 0.021791811 | 0.1444269 | 1 |
| 57_139 | A_23_P100441 | NM_024946 | FAM192A | 0.160909582 | 0.250887196 | 0.695211787 | 1 | -0.217651409 | 0.084960856 | 0.3139087 | 1 |
| 9_156 | A_23_P45294 | NM_207318 | FAM199X | 0.035333921 | 0.795371907 | 0.949830465 | 1 | 0.682803063 | 0.000237223 | 0.0087765 | 1 |
| 172_6 | A_23_P115743 | NM_022063 | FAM204A | -0.132739509 | 0.35545371 | 0.774004626 | 1 | -0.098353282 | 0.404356976 | 0.6826483 | 1 |
| 355_139 | A_33_P3297642 | NM_022063 | FAM204A | 0.049514602 | 0.680494383 | 0.919745252 | 1 | -0.320940395 | 0.022177527 | 0.1465515 | 1 |
| 279_7 | A_23_P216568 | NM_017832 | FAM206A | 0.069051544 | 0.587749517 | 0.886487993 | 1 | 0.100813907 | 0.385351055 | 0.6660586 | 1 |
| 72_146 | A_33_P3705907 | NM_058190 | FAM207A | 0.100095976 | 0.460424159 | 0.834778335 | 1 | -0.043748566 | 0.724201115 | 0.887458 | 1 |
| 70_55 | A_33_P3288700 | NM_058190 | FAM207A | 0.119256028 | 0.342191885 | 0.767209337 | 1 | 0.042658182 | 0.758535797 | 0.9031132 | 1 |
| 163_66 | A_23_P61202 | NM_058190 | FAM207A | 0.244446424 | 0.16257601 | 0.602835407 | 1 | -0.06021846 | 0.751846853 | 0.9007747 | 1 |
| 186_99 | A_21_P0000195 | NM_017782 | FAM208B | 0.279154228 | 0.043391263 | 0.390521637 | 1 | 0.199744756 | 0.231987472 | 0.5198175 | 1 |
| 221_132 | A_24_P148043 | NM_014864 | FAM20B | -0.107394246 | 0.414694348 | 0.812829671 | 1 | 0.250194083 | 0.105602801 | 0.3528191 | 1 |
| 15_39 | A_24_P898945 | NM_001098801 | FAM210A | 0.017743523 | 0.900141442 | 0.97618995 | 1 | 0.162463781 | 0.210514841 | 0.4949123 | 1 |
| 167_13 | A_23_P68486 | NM_080821 | FAM210B | -0.21302904 | 0.161238038 | 0.600715378 | 1 | -0.175618154 | 0.1902124 | 0.4719433 | 1 |
| 193_130 | A_33_P3239267 | NM_203370 | FAM212A | -0.00938705 | 0.947439053 | 0.990109165 | 1 | 0.166915167 | 0.240770316 | 0.528472 | 1 |
| 78_118 | A_24_P336577 | NM_019099 | FAM212B | -0.045191078 | 0.719065135 | 0.93440569 | 1 | -0.108936287 | 0.333873013 | 0.6205551 | 1 |
| 67_54 | A_23_P63660 | NM_032333 | FAM213A | -0.017895938 | 0.930480594 | 0.98549075 | 1 | 0.613880539 | 0.000656484 | 0.0174138 | 1 |
| 382_92 | A_33_P3367332 | NM_001195736 | FAM213B | -0.294229533 | 0.03800317 | 0.371467292 | 1 | 0.042423814 | 0.699701661 | 0.8748868 | 1 |
| 5_76 | A_33_P3238052 | NM_013300 | FAM216A | 0.165399765 | 0.25204838 | 0.695932312 | 1 | 0.007985208 | 0.950492012 | 0.982768 | 1 |
| 222_52 | A_23_P68505 | NM_001190826 | FAM217B | 0.240460892 | 0.118678739 | 0.540937779 | 1 | 0.14909826 | 0.199551642 | 0.4829691 | 1 |
| 122_126 | A_23_P20804 | NM_147202 | FAM219A | 0.039999899 | 0.780507624 | 0.94834868 | 1 | -0.045056189 | 0.708004004 | 0.8787993 | 1 |
| 315_15 | A_23_P376799 | NM_015262 | FAM21C | -0.238774871 | 0.07748549 | 0.464989282 | 1 | 0.364578476 | 0.015207536 | 0.1182102 | 1 |
| 298_19 | A_33_P3279124 | NM_001169107 | FAM21C | -0.060535591 | 0.751910324 | 0.942109358 | 1 | 0.578543691 | 0.014262265 | 0.1132023 | 1 |
| 148_43 | A_23_P128375 | NM_032829 | FAM222A | 0.02606078 | 0.837072065 | 0.962281738 | 1 | -0.207077873 | 0.093365874 | 0.3316513 | 1 |
| 342_140 | A_23_P141484 | NM_018182 | FAM222B | -0.044353163 | 0.753787716 | 0.942109358 | 1 | 0.192853076 | 0.184989944 | 0.4644394 | 1 |
| 377_137 | A_22_P00002166 | NM_001167676 | FAM229A | 0.035107338 | 0.783526709 | 0.94877096 | 1 | 0.219444289 | 0.19864055 | 0.4814893 | 1 |
| 143_55 | A_33_P3403733 | NM_001282321 | FAM231A | -0.092791521 | 0.473770929 | 0.839074131 | 1 | 0.060363987 | 0.659016678 | 0.8547003 | 1 |
| 143_90 | A_23_P66117 | NM_032039 | FAM234A | -0.066992251 | 0.666644159 | 0.915764478 | 1 | -0.187167769 | 0.258295335 | 0.5479154 | 1 |
| 337_89 | A_23_P52531 | NM_152644 | FAM24B | 0.399708874 | 0.012531024 | 0.265846757 | 1 | -0.109733997 | 0.59553296 | 0.8162347 | 1 |
| 239_58 | A_32_P34138 | NM_001146157 | FAM25A | -0.288591433 | 0.049229178 | 0.403845012 | 1 | 0.296914269 | 0.041058633 | 0.209427 | 1 |
| 285_101 | A_24_P342096 | NR_027421 | FAM27C | 0.291616745 | 0.045600589 | 0.394867293 | 1 | 0.089553671 | 0.466060981 | 0.7280261 | 1 |
| 18_141 | A_32_P112623 | NR_103714 | FAM27E2 | -0.124881071 | 0.440606485 | 0.827546746 | 1 | -0.29292537 | 0.112201157 | 0.3634581 | 1 |
| 241_146 | A_23_P50331 | ENST00000585831 | FAM32A | -0.030648009 | 0.815047448 | 0.955726321 | 1 | 0.475502634 | 0.019457173 | 0.1363457 | 1 |
| 16_77 | A_33_P3410589 | NM_153690 | FAM43A | 0.089320528 | 0.469586979 | 0.836960042 | 1 | -0.626147981 | 0.001341212 | 0.026419 | 1 |
| 144_74 | A_32_P138004 | NM_207009 | FAM45A | -0.082866149 | 0.611518923 | 0.89742483 | 1 | 0.118353245 | 0.330254888 | 0.6168012 | 1 |
| 98_104 | A_23_P43255 | NM_016623 | FAM49B | 0.107091705 | 0.4464173 | 0.830151023 | 1 | 0.047586576 | 0.686347599 | 0.8686116 | 1 |
| 116_94 | A_23_P34107 | NM_004699 | FAM50A | 0.216862959 | 0.141857997 | 0.575520124 | 1 | 0.316255112 | 0.04463971 | 0.2186685 | 1 |
| 309_24 | A_33_P3209895 | NM_001130997 | FAM58A | -0.041363709 | 0.785856139 | 0.94877096 | 1 | 0.070823291 | 0.645305174 | 0.8457094 | 1 |
| 29_91 | A_23_P404005 | NM_152274 | FAM58A | -0.002834872 | 0.986221954 | 0.995873498 | 1 | 1.052780893 | 1.19818E-05 | 0.001567 | 0.13476 |
| 348_94 | A_23_P13663 | NM_021238 | FAM60A | -0.13999825 | 0.258824419 | 0.700691339 | 1 | 0.285336422 | 0.042135456 | 0.2126054 | 1 |
| 289_24 | A_32_P103837 | NM_021238 | FAM60A | -0.070520095 | 0.605804014 | 0.894904051 | 1 | 0.091516092 | 0.51948826 | 0.7656512 | 1 |
| 50_47 | A_23_P160546 | NM_001040217 | FAM63A | -0.041094576 | 0.752224314 | 0.942109358 | 1 | -0.114189914 | 0.372074871 | 0.6550958 | 1 |
| 367_130 | A_32_P230868 | NM_024519 | FAM65A | -0.091558963 | 0.550578164 | 0.874464562 | 1 | -0.072667854 | 0.640080927 | 0.8426761 | 1 |
| 172_152 | A_33_P3334443 | NM_001252271 | FAM69A | -0.074796699 | 0.581140296 | 0.883808109 | 1 | 0.157772416 | 0.201267362 | 0.4835834 | 1 |
| 311_26 | A_33_P3242952 | NM_001123168 | FAM72A | -0.071413012 | 0.603167559 | 0.893532545 | 1 | -0.016321347 | 0.910885753 | 0.9687669 | 1 |
| 104_75 | A_33_P3379436 | NR_110998 | FAM74A4 | -0.056041228 | 0.713402595 | 0.93206229 | 1 | 0.057434094 | 0.696430057 | 0.8734794 | 1 |
| 125_44 | A_32_P19752 | NM_144664 | FAM76B | 0.086699008 | 0.556526361 | 0.87635403 | 1 | -0.268565153 | 0.059013801 | 0.2562669 | 1 |
| 175_12 | A_33_P3335386 | ENST00000388995 | FAM83G | -0.173835779 | 0.310952933 | 0.74326753 | 1 | -0.032576722 | 0.790041618 | 0.9168577 | 1 |
| 14_107 | A_33_P3390950 | ENST00000534398 | FAM83H-AS1 | 0.054286471 | 0.689441889 | 0.92263881 | 1 | -0.047516962 | 0.79897107 | 0.9211015 | 1 |
| 61_43 | A_24_P413920 | NM_145175 | FAM84A | -0.013481898 | 0.911552925 | 0.979345742 | 1 | 0.104995218 | 0.363140492 | 0.6475142 | 1 |
| 351_66 | A_24_P280868 | NM_001137610 | FAM86B2 | 0.110326381 | 0.402512816 | 0.805674814 | 1 | 0.331259725 | 0.016911731 | 0.1249712 | 1 |
| 20_159 | A_33_P3310430 | NM_001137610 | FAM86B2 | 0.276467202 | 0.055379848 | 0.422185511 | 1 | 0.120743257 | 0.31606144 | 0.6034316 | 1 |
| 190_65 | A_33_P3269740 | NM_001137610 | FAM86B2 | 0.307836526 | 0.042872832 | 0.390076378 | 1 | 0.288174927 | 0.053573396 | 0.242796 | 1 |
| 153_110 | A_33_P3220612 | NM_001098784 | FAM89B | 0.064722522 | 0.625497527 | 0.899925451 | 1 | 0.067269023 | 0.617043748 | 0.8260752 | 1 |
| 32_97 | A_32_P103695 | NM_145269 | FAM92A1 | 0.28344794 | 0.057489608 | 0.423696939 | 1 | -0.36508007 | 0.011230624 | 0.0987575 | 1 |
| 299_119 | A_33_P3416037 | NM_001014812 | FAM96A | 0.332983373 | 0.096460904 | 0.500009516 | 1 | -0.045647406 | 0.814755051 | 0.9279544 | 1 |
| 204_88 | A_23_P77455 | NM_016062 | FAM96B | 0.574957268 | 0.01783784 | 0.296237697 | 1 | -0.243260661 | 0.421028602 | 0.6965338 | 1 |
| 94_37 | A_23_P369983 | NM_174905 | FAM98C | -0.274368453 | 0.076042051 | 0.462709424 | 1 | 0.016741925 | 0.885386276 | 0.9608259 | 1 |
| 120_137 | A_33_P3378785 | ENST00000538190 | FAM99A | 0.04593273 | 0.739969852 | 0.940558508 | 1 | 0.089573259 | 0.497128578 | 0.7522138 | 1 |
| 179_137 | A_23_P377888 | NM_014967 | FAN1 | -0.032886186 | 0.788868301 | 0.94877096 | 1 | -0.008778996 | 0.938647475 | 0.9786325 | 1 |
| 348_22 | A_23_P206441 | NM_000135 | FANCA | -0.249387008 | 0.179896103 | 0.620483827 | 1 | 0.075628112 | 0.662494508 | 0.8564455 | 1 |
| 344_4 | A_23_P206441 | NM_000135 | FANCA | -0.175158848 | 0.333456937 | 0.76120935 | 1 | 0.07684064 | 0.605770178 | 0.8203609 | 1 |
| 50_8 | A_23_P206441 | NM_000135 | FANCA | -0.093624579 | 0.658088037 | 0.911390964 | 1 | 0.078366822 | 0.570563458 | 0.8011395 | 1 |
| 245_39 | A_33_P3286422 | NM_001018112 | FANCA | -0.079745634 | 0.567078065 | 0.880916218 | 1 | -0.098062234 | 0.466861374 | 0.7287703 | 1 |
| 373_146 | A_23_P206441 | NM_000135 | FANCA | -0.034669824 | 0.839062712 | 0.962281738 | 1 | 0.032273773 | 0.804483222 | 0.9231312 | 1 |
| 86_144 | A_23_P206441 | NM_000135 | FANCA | -0.030349484 | 0.853370193 | 0.965079421 | 1 | -0.076704835 | 0.598246057 | 0.8174528 | 1 |
| 99_128 | A_23_P206441 | NM_000135 | FANCA | -0.018587066 | 0.908777805 | 0.97860781 | 1 | -0.015281923 | 0.920187388 | 0.9713137 | 1 |
| 30_83 | A_23_P206441 | NM_000135 | FANCA | -0.006131817 | 0.974177425 | 0.994877235 | 1 | 0.085778596 | 0.602444444 | 0.8190176 | 1 |
| 178_43 | A_23_P206441 | NM_000135 | FANCA | 0.007591025 | 0.96598403 | 0.993916368 | 1 | 0.140638885 | 0.2982324 | 0.5882532 | 1 |
| 50_136 | A_23_P206441 | NM_000135 | FANCA | 0.02569666 | 0.895765406 | 0.975468897 | 1 | 0.010937233 | 0.940493083 | 0.9790998 | 1 |
| 203_115 | A_23_P206441 | NM_000135 | FANCA | 0.055112538 | 0.804167952 | 0.952398794 | 1 | -0.002277445 | 0.9875904 | 0.996182 | 1 |
| 65_12 | A_23_P32021 | NM_000136 | FANCC | 0.101713664 | 0.459374617 | 0.833745766 | 1 | -0.155153295 | 0.154483569 | 0.423465 | 1 |
| 231_98 | A_33_P3257808 | NM_001018115 | FANCD2 | -0.105135434 | 0.421711712 | 0.817849907 | 1 | 0.100582093 | 0.493300496 | 0.749345 | 1 |
| 152_42 | A_23_P42335 | NM_021922 | FANCE | 0.00029639 | 0.998537769 | 0.999808735 | 1 | 0.16279739 | 0.252823036 | 0.5409211 | 1 |
| 23_60 | A_33_P3386364 | NM_022725 | FANCF | 0.081818813 | 0.581917288 | 0.883808109 | 1 | -0.012274175 | 0.922000484 | 0.9721327 | 1 |
| 315_155 | A_23_P71644 | NM_004629 | FANCG | 0.158133843 | 0.250194726 | 0.694615167 | 1 | -0.091868585 | 0.446697178 | 0.715568 | 1 |
| 217_50 | A_32_P95729 | NM_018193 | FANCI | 0.19753776 | 0.187494498 | 0.630397286 | 1 | 0.205108962 | 0.091394008 | 0.3267816 | 1 |
| 123_58 | A_23_P131383 | NM_018062 | FANCL | -0.26566211 | 0.086948571 | 0.482041914 | 1 | 0.350948552 | 0.037392641 | 0.1989381 | 1 |
| 259_130 | A_32_P106732 | NM_020937 | FANCM | -0.204503891 | 0.196029864 | 0.642117721 | 1 | 0.068881479 | 0.56819894 | 0.7993163 | 1 |
| 70_107 | A_23_P78685 | NM_004461 | FARSA | 0.417281372 | 0.015583334 | 0.283139497 | 1 | -0.248492532 | 0.116413259 | 0.3698864 | 1 |
| 152_131 | A_33_P3329344 | NM_004104 | FASN | 0.138106419 | 0.387405804 | 0.79582353 | 1 | 0.176168059 | 0.341457999 | 0.6281272 | 1 |
| 233_28 | A_23_P502930 | NM_006712 | FASTK | 0.133952512 | 0.428983175 | 0.821032685 | 1 | 0.071881651 | 0.647429254 | 0.8465051 | 1 |
| 260_102 | A_23_P345830 | NM_014929 | FASTKD2 | 0.131497763 | 0.31039819 | 0.743121622 | 1 | 0.564948885 | 0.000368449 | 0.0115753 | 1 |
| 192_153 | A_23_P58489 | NM_024091 | FASTKD3 | 0.241465269 | 0.109612808 | 0.526954706 | 1 | -0.198239829 | 0.180734915 | 0.4591655 | 1 |
| 137_1 | A_23_P380724 | NM_021826 | FASTKD5 | -0.304728021 | 0.080995423 | 0.470809839 | 1 | -0.216292373 | 0.125637547 | 0.3853351 | 1 |
| 357_111 | A_33_P3257513 | NM_001008781 | FAT3 | -0.236038554 | 0.152696202 | 0.591085204 | 1 | 0.296942215 | 0.157284124 | 0.4286345 | 1 |
| 277_5 | A_32_P11471 | NM_001997 | FAU | -0.241845508 | 0.067117071 | 0.447031252 | 1 | -0.06045146 | 0.572100839 | 0.8016968 | 1 |
| 337_55 | A_23_P78888 | NM_001436 | FBL | 0.019213099 | 0.884816085 | 0.972822545 | 1 | 0.0056662 | 0.968387111 | 0.9887791 | 1 |
| 78_83 | A_23_P211631 | NM_006486 | FBLN1 | -0.286634829 | 0.170361446 | 0.610754308 | 1 | -0.115091464 | 0.612468979 | 0.8236831 | 1 |
| 139_60 | A_33_P3249872 | NM_001996 | FBLN1 | -0.100052303 | 0.461162513 | 0.834781157 | 1 | 0.026417784 | 0.856968862 | 0.9488412 | 1 |
| 353_111 | A_33_P3329549 | NM_001105079 | FBRS | -0.055125879 | 0.688153948 | 0.922029409 | 1 | 0.077184192 | 0.540769542 | 0.7811501 | 1 |
| 251_101 | A_23_P38015 | NM_001105079 | FBRS | 0.072747561 | 0.561370428 | 0.87849363 | 1 | 0.047340281 | 0.735471549 | 0.893156 | 1 |
| 374_35 | A_23_P12635 | NM_024326 | FBXL15 | -0.361291719 | 0.014574647 | 0.276124961 | 1 | -0.121173674 | 0.30197267 | 0.5927818 | 1 |
| 48_92 | A_33_P3239839 | NM_024963 | FBXL18 | -0.120932515 | 0.360043444 | 0.776462619 | 1 | -0.217016653 | 0.083632156 | 0.3113575 | 1 |
| 216_43 | A_33_P3211404 | XM_005249853 | FBXL18 | 0.029805189 | 0.861223025 | 0.967258331 | 1 | -0.024585882 | 0.875609865 | 0.9570191 | 1 |
| 187_161 | A_23_P338168 | NM_001099784 | FBXL19 | 0.225958379 | 0.084369873 | 0.478632531 | 1 | -0.060192302 | 0.601137207 | 0.8185598 | 1 |
| 20_122 | A_33_P3333587 | NM_032875 | FBXL20 | -0.136130996 | 0.333580105 | 0.761272543 | 1 | 0.227529996 | 0.208989549 | 0.4935701 | 1 |
| 66_37 | A_23_P214739 | NM_012160 | FBXL4 | -0.036390069 | 0.774907567 | 0.946321166 | 1 | 0.098123387 | 0.38466264 | 0.6656373 | 1 |
| 178_137 | A_33_P3284646 | NM_012162 | FBXL6 | 0.493954082 | 0.003550751 | 0.17583436 | 1 | -0.110078597 | 0.332945646 | 0.6196657 | 1 |
| 144_15 | A_33_P3259507 | NM_012166 | FBXO10 | -0.215736419 | 0.17233413 | 0.612419595 | 1 | 0.098330114 | 0.510857645 | 0.7601476 | 1 |
| 330_27 | A_23_P395911 | ENST00000601394 | FBXO17 | 0.176549164 | 0.48369821 | 0.843654204 | 1 | 0.001929135 | 0.994643616 | 0.9975355 | 1 |
| 22_48 | A_23_P53736 | NM_033624 | FBXO21 | 0.20145487 | 0.154050713 | 0.593311979 | 1 | 0.242978546 | 0.203489324 | 0.486015 | 1 |
| 84_149 | A_23_P65870 | NM_012170 | FBXO22 | 0.248476689 | 0.064835174 | 0.44148803 | 1 | -0.084022139 | 0.457298502 | 0.7207865 | 1 |
| 151_48 | A_23_P94159 | NM_183421 | FBXO25 | -0.472917881 | 0.004832708 | 0.196694236 | 1 | 0.470262778 | 0.002133183 | 0.0348213 | 1 |
| 102_11 | A_24_P41021 | NM_203301 | FBXO33 | -0.087816949 | 0.495718683 | 0.850005476 | 1 | 0.016808819 | 0.891199913 | 0.9619458 | 1 |
| 19_26 | A_23_P349676 | NM_001080410 | FBXO41 | -0.046617433 | 0.736460174 | 0.939291435 | 1 | 0.357346729 | 0.008797081 | 0.0856548 | 1 |
| 1_18 | A_24_P333494 | NM_018994 | FBXO42 | -0.161573587 | 0.229735092 | 0.675835036 | 1 | 0.109933701 | 0.349067289 | 0.6342423 | 1 |
| 94_21 | A_33_P3384871 | NM_001142522 | FBXO5 | 0.022499439 | 0.877285199 | 0.971233905 | 1 | -0.29725897 | 0.062773811 | 0.2651944 | 1 |
| 97_43 | A_23_P29225 | NM_012179 | FBXO7 | 0.048169631 | 0.701248178 | 0.925829227 | 1 | -0.084626539 | 0.518373174 | 0.7648565 | 1 |
| 377_132 | A_23_P254120 | NM_033480 | FBXO9 | 0.101044371 | 0.597447073 | 0.89144727 | 1 | -0.49948005 | 0.001542928 | 0.0284015 | 1 |
| 346_100 | A_23_P214046 | NM_012300 | FBXW11 | -0.133453742 | 0.406483629 | 0.807799728 | 1 | -0.024947804 | 0.852648242 | 0.9462939 | 1 |
| 210_55 | A_21_P0000598 | NM_022039 | FBXW4 | 0.013074101 | 0.935566819 | 0.986674313 | 1 | -0.181675673 | 0.135783463 | 0.3999712 | 1 |
| 368_106 | A_23_P112397 | NM_018998 | FBXW5 | 0.030678832 | 0.852496972 | 0.965069985 | 1 | -0.28241674 | 0.086553183 | 0.3170187 | 1 |
| 208_96 | A_23_P160849 | NM_004106 | FCER1G | 0.171513275 | 0.196211433 | 0.642454933 | 1 | -0.019391015 | 0.890879121 | 0.9619458 | 1 |
| 380_67 | A_33_P3264505 | NM_015962 | FCF1 | -0.014893372 | 0.917867921 | 0.981346939 | 1 | 0.072819429 | 0.619805806 | 0.8276155 | 1 |
| 295_119 | A_23_P55936 | NM_004107 | FCGRT | -0.17869377 | 0.169679854 | 0.609201536 | 1 | -0.01882066 | 0.929901685 | 0.9744555 | 1 |
| 308_36 | A_33_P3255509 | NM_001161357 | FCHO1 | -1.04166778 | 8.56402E-05 | 0.035819662 | 1 | 0.328009087 | 0.028127186 | 0.1682694 | 1 |
| 209_18 | A_23_P71319 | NM_004462 | FDFT1 | -0.109642754 | 0.426878888 | 0.820875237 | 1 | 0.490562829 | 0.001952348 | 0.0332195 | 1 |
| 73_60 | A_24_P114183 | NM_002004 | FDPS | -0.077271118 | 0.574421563 | 0.882856304 | 1 | -0.068642959 | 0.610512423 | 0.8225878 | 1 |
| 177_60 | A_32_P95223 | NR_003262 | FDPSP2 | 0.06066369 | 0.675686724 | 0.918536046 | 1 | -0.136837764 | 0.228706805 | 0.5163118 | 1 |
| 88_29 | A_24_P416660 | NM_001031734 | FDX1L | 0.168289789 | 0.251167537 | 0.695211787 | 1 | -0.05385977 | 0.685051223 | 0.8683976 | 1 |
| 143_103 | A_33_P3320748 | NM_001031734 | FDX1L | 0.209149657 | 0.25600158 | 0.699039445 | 1 | 0.172012304 | 0.216396098 | 0.5028916 | 1 |
| 155_58 | A_23_P38154 | NM_004110 | FDXR | -0.372394305 | 0.016045313 | 0.286184492 | 1 | 0.449238485 | 0.0050804 | 0.0607218 | 1 |
| 230_29 | A_33_P3383871 | NM_020177 | FEM1C | -0.364896529 | 0.013897749 | 0.271753414 | 1 | 0.262623215 | 0.080492623 | 0.3057415 | 1 |
| 25_65 | A_23_P131935 | NM_017671 | FERMT1 | 0.634793368 | 0.006879473 | 0.225693626 | 1 | -0.049273207 | 0.754215882 | 0.9012175 | 1 |
| 381_24 | A_33_P3344477 | NM_001134999 | FERMT2 | 0.013429858 | 0.952357298 | 0.990752085 | 1 | 0.299670961 | 0.138239515 | 0.4035245 | 1 |
| 313_28 | A_21_P0014483 | NR_046251 | FGD5-AS1 | -0.163292361 | 0.234645367 | 0.680907543 | 1 | 0.171043556 | 0.16037252 | 0.4336883 | 1 |
| 99_7 | A_23_P301304 | NM_023110 | FGFR1 | -0.192992095 | 0.154702307 | 0.593652999 | 1 | -0.195353027 | 0.14033979 | 0.4064902 | 1 |
| 191_35 | A_23_P145134 | NM_007045 | FGFR1OP | -0.068341763 | 0.62523527 | 0.899887301 | 1 | 0.063289001 | 0.616733568 | 0.8260752 | 1 |
| 208_31 | A_33_P3369258 | NM_001171888 | FGFR1OP2 | -0.087181127 | 0.511147964 | 0.856978323 | 1 | -0.177265315 | 0.241316158 | 0.529061 | 1 |
| 74_127 | A_32_P209094 | NM_018291 | FGGY | -0.506864628 | 0.001803305 | 0.137299166 | 1 | -0.216520345 | 0.365766378 | 0.6499881 | 1 |
| 224_25 | A_23_P34733 | NM_000143 | FH | 0.107942896 | 0.586926224 | 0.886416406 | 1 | -0.233645623 | 0.151949689 | 0.4205074 | 1 |
| 121_105 | A_23_P108751 | NM_001039492 | FHL2 | -0.072380842 | 0.620493377 | 0.89867047 | 1 | 0.560239827 | 0.004320451 | 0.0551282 | 1 |
| 184_18 | A_32_P34444 | NM_025135 | FHOD3 | 0.0726708 | 0.74877074 | 0.941597731 | 1 | 0.388075452 | 0.031939457 | 0.1821618 | 1 |
| 175_151 | A_33_P3319581 | NM_001013690 | FIGNL2 | 0.141920445 | 0.455728949 | 0.833211774 | 1 | 0.028306779 | 0.860727364 | 0.9501054 | 1 |
| 207_81 | A_23_P58337 | NM_030917 | FIP1L1 | 0.378920176 | 0.029735198 | 0.339352673 | 1 | 0.026165316 | 0.871767596 | 0.9551761 | 1 |
| 138_71 | A_24_P277955 | NM_016068 | FIS1 | -0.060768728 | 0.660949196 | 0.912353222 | 1 | -0.215602409 | 0.118678518 | 0.3738872 | 1 |
| 370_143 | A_23_P150693 | NM_014344 | FJX1 | 0.075543806 | 0.551691101 | 0.87513796 | 1 | -0.033972524 | 0.820292348 | 0.9309091 | 1 |
| 99_155 | A_33_P3267296 | NM_016594 | FKBP11 | -0.16309206 | 0.244540721 | 0.69010536 | 1 | -0.449848692 | 0.013142949 | 0.1083529 | 1 |
| 382_90 | A_32_P224149 | NM_015258 | FKBP15 | -0.064038841 | 0.700232114 | 0.925289905 | 1 | -0.084846222 | 0.467746356 | 0.7295442 | 1 |
| 294_61 | A_32_P50522 | NM_000801 | FKBP1A | 0.220959126 | 0.185081312 | 0.627864254 | 1 | -0.120990914 | 0.33543925 | 0.6217345 | 1 |
| 81_70 | A_33_P3403615 | NM_000801 | FKBP1A | 0.309940338 | 0.039106288 | 0.374228433 | 1 | -0.193571865 | 0.153293829 | 0.4219648 | 1 |
| 263_28 | A_33_P3273020 | NM_004470 | FKBP2 | -0.486556805 | 0.004045389 | 0.188782732 | 1 | 0.297875327 | 0.043583059 | 0.2158946 | 1 |
| 274_4 | A_23_P117558 | NM_002013 | FKBP3 | -0.034330857 | 0.850561692 | 0.965069985 | 1 | 0.037245814 | 0.79938832 | 0.9212674 | 1 |
| 73_9 | A_23_P117558 | NM_002013 | FKBP3 | -0.000623159 | 0.997098259 | 0.999477577 | 1 | 0.068100895 | 0.60163913 | 0.8189078 | 1 |
| 367_59 | A_23_P117558 | NM_002013 | FKBP3 | 0.052763963 | 0.73823711 | 0.940179103 | 1 | 0.076603102 | 0.61865899 | 0.8270602 | 1 |
| 208_12 | A_23_P117558 | NM_002013 | FKBP3 | 0.056425033 | 0.743287131 | 0.941128659 | 1 | 0.078043125 | 0.561977195 | 0.7958803 | 1 |
| 81_54 | A_23_P117558 | NM_002013 | FKBP3 | 0.145792759 | 0.436320122 | 0.825909907 | 1 | 0.06861064 | 0.683766982 | 0.8681716 | 1 |
| 327_142 | A_23_P117558 | NM_002013 | FKBP3 | 0.183629989 | 0.277105128 | 0.717374603 | 1 | 0.085865593 | 0.581547888 | 0.8080221 | 1 |
| 204_84 | A_23_P117558 | NM_002013 | FKBP3 | 0.193784784 | 0.318792641 | 0.750205467 | 1 | 0.168285553 | 0.278353061 | 0.5695244 | 1 |
| 198_52 | A_23_P117558 | NM_002013 | FKBP3 | 0.234344438 | 0.194779298 | 0.640909226 | 1 | 0.087457088 | 0.486777217 | 0.7438564 | 1 |
| 310_114 | A_23_P117558 | NM_002013 | FKBP3 | 0.247966502 | 0.214896023 | 0.661348172 | 1 | 0.032616617 | 0.829721851 | 0.9352457 | 1 |
| 156_67 | A_23_P117558 | NM_002013 | FKBP3 | 0.371075243 | 0.093657461 | 0.496537113 | 1 | 0.083287799 | 0.654286429 | 0.8511836 | 1 |
| 170_62 | A_23_P128372 | NM_002014 | FKBP4 | 0.069736734 | 0.601269412 | 0.893142784 | 1 | 0.298583363 | 0.044848626 | 0.2192145 | 1 |
| 348_53 | A_33_P3272990 | NM_012181 | FKBP8 | -0.099845465 | 0.441802727 | 0.828388614 | 1 | 0.057964487 | 0.624080695 | 0.8308399 | 1 |
| 115_112 | A_23_P334709 | NM_007270 | FKBP9 | -0.112578254 | 0.370203529 | 0.783949459 | 1 | -0.493200123 | 0.008576273 | 0.0846426 | 1 |
| 44_89 | A_23_P414308 | NM_144606 | FLCN | 0.257764969 | 0.079659375 | 0.468528124 | 1 | -0.451891565 | 0.001432886 | 0.0274077 | 1 |
| 372_157 | A_23_P4425 | NM_002018 | FLII | 0.183539225 | 0.34565208 | 0.7684824 | 1 | -0.218627873 | 0.164839641 | 0.4405759 | 1 |
| 83_18 | A_33_P3402600 | A_33_P3402600 | FLJ20021 | -0.176554938 | 0.184377443 | 0.627048022 | 1 | 0.225153925 | 0.085559857 | 0.314529 | 1 |
| 124_14 | A_33_P3813684 | NR_033966 | FLJ31662 | 0.095255414 | 0.43060645 | 0.821617604 | 1 | 0.240386013 | 0.081319534 | 0.3073255 | 1 |
| 312_120 | A_19_P00320314 | NR_104643 | FLJ32255 | 0.027588233 | 0.852105801 | 0.965069985 | 1 | 0.032097443 | 0.821040083 | 0.9312462 | 1 |
| 94_132 | A_19_P00319721 | NR_026835 | FLJ37201 | 0.150392782 | 0.548573965 | 0.873756475 | 1 | 0.032384787 | 0.867767597 | 0.9533012 | 1 |
| 274_114 | A_32_P100258 | NR_024279 | FLJ37453 | -0.25384962 | 0.063654422 | 0.437788275 | 1 | -0.118606786 | 0.364265833 | 0.6486061 | 1 |
| 313_154 | A_23_P302134 | NR_024279 | FLJ37453 | -0.200537507 | 0.118831623 | 0.541161433 | 1 | -0.066146786 | 0.632082697 | 0.8362229 | 1 |
| 51_3 | A_33_P3296991 | NR_024413 | FLJ42393 | -0.086141818 | 0.623593595 | 0.899250294 | 1 | 0.019917508 | 0.894440266 | 0.9633162 | 1 |
| 214_66 | A_21_P0010773 | NR_033856 | FLJ43315 | 0.394326829 | 0.07443151 | 0.460523107 | 1 | 0.039603979 | 0.834120537 | 0.9374603 | 1 |
| 71_35 | A_33_P3366120 | NM_001110556 | FLNA | 0.083482806 | 0.593931032 | 0.88929054 | 1 | 0.816206538 | 0.003940825 | 0.0520828 | 1 |
| 352_139 | A_23_P214603 | NM_005803 | FLOT1 | 0.071412164 | 0.661972126 | 0.912956463 | 1 | -0.251058771 | 0.114136517 | 0.3661247 | 1 |
| 4_156 | A_23_P55319 | NM_004475 | FLOT2 | 0.143020091 | 0.411996089 | 0.812009855 | 1 | -0.241550968 | 0.143261249 | 0.4103028 | 1 |
| 297_45 | A_23_P12113 | NM_014053 | FLVCR1 | -0.207662053 | 0.189389286 | 0.632266453 | 1 | 0.086691733 | 0.503872411 | 0.7571515 | 1 |
| 261_140 | A_23_P12113 | NM_014053 | FLVCR1 | -0.13727731 | 0.337475371 | 0.763334677 | 1 | 0.01202512 | 0.928199979 | 0.9738557 | 1 |
| 111_1 | A_23_P12113 | NM_014053 | FLVCR1 | -0.122634337 | 0.365525381 | 0.781583876 | 1 | 0.115480353 | 0.374482864 | 0.6567611 | 1 |
| 103_55 | A_23_P12113 | NM_014053 | FLVCR1 | -0.11495299 | 0.446951165 | 0.830246888 | 1 | 0.2729335 | 0.039050187 | 0.2032381 | 1 |
| 350_130 | A_23_P12113 | NM_014053 | FLVCR1 | -0.092319285 | 0.471091681 | 0.837786694 | 1 | 0.098331901 | 0.392564352 | 0.6729418 | 1 |
| 48_45 | A_23_P12113 | NM_014053 | FLVCR1 | -0.088742015 | 0.524504781 | 0.864032081 | 1 | 0.226357066 | 0.064768868 | 0.2692996 | 1 |
| 381_82 | A_23_P12113 | NM_014053 | FLVCR1 | -0.041003307 | 0.748733474 | 0.941597731 | 1 | 0.021256526 | 0.849888062 | 0.9450036 | 1 |
| 374_155 | A_23_P12113 | NM_014053 | FLVCR1 | -0.037110996 | 0.784291313 | 0.94877096 | 1 | 0.090407015 | 0.449210028 | 0.7172616 | 1 |
| 216_145 | A_23_P12113 | NM_014053 | FLVCR1 | -0.018609737 | 0.897541986 | 0.975758314 | 1 | 0.061210796 | 0.602713517 | 0.8190176 | 1 |
| 293_158 | A_23_P12113 | NM_014053 | FLVCR1 | 0.020500278 | 0.887263631 | 0.973024604 | 1 | 0.130159806 | 0.312267356 | 0.6008481 | 1 |
| 364_131 | A_33_P3415923 | NM_175736 | FMNL3 | -0.078021388 | 0.575621219 | 0.883311119 | 1 | 0.192533085 | 0.123280578 | 0.3812498 | 1 |
| 119_72 | A_23_P77813 | NM_024619 | FN3KRP | 0.162902563 | 0.289391788 | 0.727382494 | 1 | -0.078961065 | 0.565332626 | 0.7976902 | 1 |
| 262_53 | A_33_P3367565 | XM_005251815 | FNBP1 | -0.316702635 | 0.031346896 | 0.344912707 | 1 | 0.314190279 | 0.078830526 | 0.3024084 | 1 |
| 110_136 | A_23_P417942 | NM_001024948 | FNBP1L | 0.111936158 | 0.382082145 | 0.791182167 | 1 | -0.139186317 | 0.29681361 | 0.587256 | 1 |
| 332_9 | A_23_P395595 | NM_015308 | FNBP4 | 0.072394194 | 0.558136443 | 0.877108086 | 1 | -0.040086199 | 0.754119205 | 0.9012175 | 1 |
| 319_12 | A_23_P25503 | NM_001079673 | FNDC3A | -0.229413411 | 0.08289366 | 0.475499013 | 1 | -0.373047927 | 0.009872608 | 0.0910888 | 1 |
| 150_18 | A_33_P3805090 | NM_020840 | FNIP2 | 0.002320401 | 0.986227046 | 0.995873498 | 1 | 0.09122412 | 0.474658384 | 0.7346553 | 1 |
| 162_153 | A_23_P24926 | NM_002027 | FNTA | 0.024111615 | 0.862649866 | 0.967572108 | 1 | 0.013773607 | 0.915000024 | 0.9704901 | 1 |
| 50_131 | A_23_P53176 | NM_016725 | FOLR1 | -0.127302866 | 0.46886181 | 0.836630229 | 1 | -0.576809127 | 0.001115916 | 0.0238152 | 1 |
| 309_27 | A_23_P390744 | NM_144600 | FOPNL | -0.225538896 | 0.182749402 | 0.623994615 | 1 | 0.396109516 | 0.005870363 | 0.0669736 | 1 |
| 262_142 | A_24_P365515 | NM_021784 | FOXA2 | -0.28500578 | 0.072253308 | 0.457123394 | 1 | -0.378192548 | 0.031870514 | 0.1820665 | 1 |
| 203_86 | A_23_P140527 | NM_012182 | FOXB1 | -0.157979334 | 0.205092899 | 0.65172692 | 1 | 0.05919435 | 0.630471343 | 0.8354644 | 1 |
| 274_157 | A_22_P00008308 | NR_121635 | FOXD3-AS1 | 0.007087683 | 0.970504606 | 0.994466847 | 1 | 0.097365191 | 0.721608482 | 0.8860556 | 1 |
| 167_9 | A_33_P3269408 | NM_207426 | FOXI2 | -0.257814211 | 0.212189669 | 0.659049242 | 1 | 0.163957026 | 0.268552935 | 0.5590255 | 1 |
| 221_26 | A_23_P566 | NM_014947 | FOXJ3 | -0.098745911 | 0.421234821 | 0.817635849 | 1 | -0.064185066 | 0.577012794 | 0.8048695 | 1 |
| 217_60 | A_33_P3392952 | NM_004514 | FOXK2 | 0.093664457 | 0.495545184 | 0.850005476 | 1 | -0.21556143 | 0.100642584 | 0.3445015 | 1 |
| 343_10 | A_23_P151150 | NM_202002 | FOXM1 | 0.059884397 | 0.625237737 | 0.899887301 | 1 | -0.206128495 | 0.138467368 | 0.4036889 | 1 |
| 168_7 | A_32_P140898 | NM_002158 | FOXN2 | -0.327215724 | 0.023199397 | 0.315256111 | 1 | -0.108851714 | 0.313644596 | 0.6015453 | 1 |
| 16_155 | A_33_P3278475 | NM_002158 | FOXN2 | 0.011239238 | 0.931296136 | 0.9855868 | 1 | -0.125077089 | 0.304202622 | 0.5935399 | 1 |
| 103_40 | A_33_P3240392 | NM_001455 | FOXO3 | -0.042980164 | 0.771763481 | 0.945351992 | 1 | 0.304275869 | 0.023813479 | 0.153837 | 1 |
| 289_163 | A_33_P3375665 | NM_001455 | FOXO3 | 0.079686872 | 0.682728338 | 0.920314282 | 1 | 0.162430592 | 0.171536232 | 0.4496526 | 1 |
| 92_73 | A_33_P3408757 | NM_001291281 | FOXO6 | -0.318613682 | 0.047502898 | 0.399787213 | 1 | 0.046084036 | 0.730769801 | 0.8908485 | 1 |
| 295_127 | A_33_P3214303 | NM_001244808 | FOXP1 | -0.083083059 | 0.583740633 | 0.884262315 | 1 | 0.054390474 | 0.685062222 | 0.8683976 | 1 |
| 170_79 | A_23_P155257 | NM_032682 | FOXP1 | 0.084433407 | 0.569471529 | 0.881307748 | 1 | -0.526942262 | 0.002696503 | 0.0410943 | 1 |
| 179_115 | A_33_P3284019 | NM_001012426 | FOXP4 | 0.31750814 | 0.052450895 | 0.412612017 | 1 | 0.037359416 | 0.787109521 | 0.9157435 | 1 |
| 56_21 | A_32_P226768 | NR_126415 | FOXP4-AS1 | -0.033023249 | 0.844674153 | 0.964376494 | 1 | 0.108189979 | 0.453821805 | 0.7206175 | 1 |
| 165_124 | A_21_P0014162 | NR_126415 | FOXP4-AS1 | 0.152648673 | 0.273877489 | 0.71613505 | 1 | -0.035251316 | 0.778227313 | 0.912217 | 1 |
| 227_4 | A_32_P164246 | NM_033260 | FOXQ1 | 0.50168007 | 0.030492968 | 0.34142035 | 1 | -0.493809543 | 0.029485262 | 0.1737144 | 1 |
| 339_101 | A_24_P147765 | NM_024955 | FOXRED2 | 0.0219292 | 0.872791144 | 0.970133819 | 1 | -0.015542626 | 0.900623944 | 0.9647888 | 1 |
| 239_34 | A_23_P9465 | NM_004957 | FPGS | 0.053947183 | 0.6627724 | 0.913361766 | 1 | -0.275556122 | 0.031835394 | 0.1820665 | 1 |
| 246_86 | A_33_P3280030 | NM_145246 | FRA10AC1 | -0.060487833 | 0.644786428 | 0.906600677 | 1 | 0.078021232 | 0.54253858 | 0.7823992 | 1 |
| 64_26 | A_32_P107876 | NM_025074 | FRAS1 | 0.159189227 | 0.289590183 | 0.727621555 | 1 | -0.098171784 | 0.375608595 | 0.657828 | 1 |
| 299_36 | A_23_P12784 | NM_012083 | FRAT2 | -0.15589719 | 0.297020674 | 0.733787437 | 1 | 0.040486117 | 0.740718416 | 0.8949226 | 1 |
| 162_13 | A_32_P43050 | NM_004477 | FRG1 | -0.13808998 | 0.282270328 | 0.724071834 | 1 | 0.4320629 | 0.004076513 | 0.0530654 | 1 |
| 243_51 | A_21_P0013728 | NM_004477 | FRG1 | -0.044790303 | 0.746792662 | 0.941470372 | 1 | 0.142855009 | 0.228585562 | 0.5163118 | 1 |
| 25_42 | A_24_P417526 | NR_003579 | FRG1BP | 0.033254075 | 0.792475518 | 0.94877096 | 1 | 0.11229054 | 0.373073299 | 0.655823 | 1 |
| 251_143 | A_23_P81717 | NM_024919 | FRMD1 | 0.182575748 | 0.467562072 | 0.83630364 | 1 | -0.02260058 | 0.919851423 | 0.9713137 | 1 |
| 168_146 | A_23_P26037 | NM_032892 | FRMD5 | 0.303123597 | 0.056736119 | 0.423246544 | 1 | -0.093172874 | 0.406995904 | 0.683921 | 1 |
| 340_59 | A_24_P67988 | NM_031904 | FRMD8 | -0.162803284 | 0.383336953 | 0.792131185 | 1 | -0.148789898 | 0.372731004 | 0.6556459 | 1 |
| 130_113 | A_33_P3411075 | NM_003088 | FSCN1 | -0.029443466 | 0.85771631 | 0.965745263 | 1 | -1.223000475 | 1.06422E-06 | 0.0007488 | 0.01197 |
| 211_1 | A_33_P3414789 | NM_024333 | FSD1 | 0.253478322 | 0.412079397 | 0.812009855 | 1 | 0.016337248 | 0.949402603 | 0.9823418 | 1 |
| 317_100 | A_33_P3318796 | NM_005860 | FSTL3 | -0.275250796 | 0.108390971 | 0.52460024 | 1 | -0.12898887 | 0.463324207 | 0.7258681 | 1 |
| 257_33 | A_24_P58337 | NM_002032 | FTH1 | 0.002838073 | 0.987970213 | 0.99652193 | 1 | -0.273207579 | 0.192284099 | 0.4739468 | 1 |
| 55_75 | A_32_P342064 | NM_002032 | FTH1 | 0.072631175 | 0.623572829 | 0.899250294 | 1 | 0.029204621 | 0.806441336 | 0.9242719 | 1 |
| 161_144 | A_33_P3253249 | NM_002032 | FTH1 | 0.11691096 | 0.479452906 | 0.841720032 | 1 | -0.115095137 | 0.415254481 | 0.691359 | 1 |
| 286_101 | A_23_P148410 | NM_031894 | FTHL17 | -0.032894547 | 0.819568685 | 0.95679041 | 1 | -0.291320696 | 0.070691967 | 0.2828433 | 1 |
| 147_24 | A_23_P50504 | NM_000146 | FTL | 0.210881517 | 0.236316613 | 0.682343916 | 1 | -0.049820486 | 0.777353642 | 0.911573 | 1 |
| 283_13 | A_32_P155247 | NM_000146 | FTL | 0.390342986 | 0.106686402 | 0.521872604 | 1 | -0.223358874 | 0.247281592 | 0.5348416 | 1 |
| 147_104 | A_19_P00812340 | NM_000146 | FTL | 0.490510756 | 0.043503485 | 0.390521637 | 1 | -0.230992264 | 0.084914305 | 0.3139087 | 1 |
| 58_89 | A_23_P113184 | NM_001080432 | FTO | 0.183409479 | 0.200647444 | 0.647493287 | 1 | -0.154958075 | 0.171378319 | 0.4494036 | 1 |
| 145_80 | A_33_P3216337 | NM_177439 | FTSJ1 | 0.40628947 | 0.01105525 | 0.256586468 | 1 | -1.178618868 | 2.71183E-05 | 0.0027477 | 0.305 |
| 179_151 | A_23_P55091 | NM_017647 | FTSJ3 | -0.086960592 | 0.589918898 | 0.887099372 | 1 | -0.14795288 | 0.273679651 | 0.5646808 | 1 |
| 284_7 | A_32_P18250 | NR_003663 | FUNDC2P2 | -0.03510067 | 0.802954029 | 0.952164507 | 1 | 0.565105794 | 0.000439367 | 0.0131076 | 1 |
| 339_156 | A_23_P107963 | NM_000148 | FUT1 | -0.068052335 | 0.647023683 | 0.907045535 | 1 | 0.212783247 | 0.195254408 | 0.4773651 | 1 |
| 185_62 | A_33_P3286536 | NM_002033 | FUT4 | 0.027728644 | 0.833213062 | 0.961375405 | 1 | -0.4578855 | 0.006465668 | 0.0709457 | 1 |
| 47_27 | A_33_P3210288 | NM_000150 | FUT6 | -0.065104495 | 0.699434655 | 0.925289905 | 1 | 0.046524219 | 0.668886702 | 0.8601611 | 1 |
| 172_35 | A_23_P60517 | NM_181425 | FXN | 0.109920987 | 0.381149436 | 0.790460066 | 1 | 0.080026169 | 0.489685851 | 0.7464713 | 1 |
| 68_22 | A_23_P132784 | NM_001013439 | FXR1 | -0.11294715 | 0.447870665 | 0.830710542 | 1 | 0.15039471 | 0.240681596 | 0.528472 | 1 |
| 203_76 | A_33_P3387300 | NM_001013438 | FXR1 | 0.116739893 | 0.38745119 | 0.79582353 | 1 | -0.057025525 | 0.707351163 | 0.8784831 | 1 |
| 59_105 | A_23_P212339 | NM_024513 | FYCO1 | -0.05170721 | 0.705158116 | 0.927376665 | 1 | -0.105961719 | 0.334280009 | 0.6208136 | 1 |
| 76_115 | A_23_P502142 | NM_002037 | FYN | 0.028487014 | 0.823842367 | 0.958162003 | 1 | -0.201900019 | 0.088572516 | 0.3210361 | 1 |
| 266_137 | A_23_P80827 | NM_001011537 | FYTTD1 | 0.062346081 | 0.672222642 | 0.917536832 | 1 | 0.020594596 | 0.848086011 | 0.9441179 | 1 |
| 247_69 | A_23_P64617 | NM_012193 | FZD4 | 0.08460868 | 0.49452355 | 0.849672223 | 1 | 0.260236398 | 0.030812401 | 0.1784713 | 1 |
| 29_161 | A_33_P3268368 | NM_016263 | FZR1 | -0.307576773 | 0.071391572 | 0.455959826 | 1 | -0.3865439 | 0.023804278 | 0.153837 | 1 |
| 339_64 | A_33_P3359344 | NM_182533 | FAAP20 | -0.07786634 | 0.551247591 | 0.87507143 | 1 | 0.065532136 | 0.592895455 | 0.8148067 | 1 |
| 101_111 | A_23_P74609 | NM_015714 | G0S2 | 0.096179484 | 0.616632669 | 0.89867047 | 1 | -0.737187274 | 0.001927786 | 0.0330012 | 1 |
| 272_76 | A_33_P3331242 | NM_005754 | G3BP1 | -0.232997204 | 0.153625294 | 0.592394196 | 1 | 0.189130633 | 0.222236039 | 0.5104122 | 1 |
| 231_41 | A_24_P380132 | NM_203505 | G3BP2 | 0.012278384 | 0.927752997 | 0.984656474 | 1 | -0.088010698 | 0.443092879 | 0.712821 | 1 |
| 343_130 | A_23_P27075 | NM_007278 | GABARAP | 0.292030029 | 0.091776219 | 0.49154825 | 1 | -0.307317278 | 0.139894823 | 0.4055147 | 1 |
| 102_116 | A_24_P356338 | NM_007285 | GABARAPL2 | 0.132800334 | 0.364715664 | 0.78112338 | 1 | -0.018281719 | 0.886088329 | 0.9608259 | 1 |
| 188_142 | A_23_P205789 | NM_002041 | GABPB1 | 0.181275948 | 0.195345154 | 0.641427135 | 1 | -0.046271583 | 0.705824305 | 0.8771922 | 1 |
| 153_138 | A_22_P00017350 | NR_024490 | GABPB1-AS1 | -0.264450761 | 0.060000527 | 0.428867769 | 1 | 0.284750108 | 0.056565564 | 0.2505683 | 1 |
| 273_49 | A_23_P73208 | NM_144618 | GABPB2 | -0.079855314 | 0.528166144 | 0.865573817 | 1 | 0.118559335 | 0.419538966 | 0.6953367 | 1 |
| 224_142 | A_23_P23221 | NM_001924 | GADD45A | -0.046749358 | 0.762027201 | 0.943540273 | 1 | -0.141271995 | 0.274062448 | 0.5649908 | 1 |
| 184_152 | A_24_P239606 | NM_015675 | GADD45B | 0.196379102 | 0.130291827 | 0.559492585 | 1 | 0.07411698 | 0.664993029 | 0.8572122 | 1 |
| 228_11 | A_33_P3273148 | NM_052850 | GADD45GIP1 | 0.067358317 | 0.618234851 | 0.89867047 | 1 | -0.167639346 | 0.225844142 | 0.5146007 | 1 |
| 261_114 | A_21_P0006496 | NM_001098411 | GAGE2B | 0.09430098 | 0.591992716 | 0.888531018 | 1 | -0.381609238 | 0.006021941 | 0.0680691 | 1 |
| 44_42 | A_23_P48807 | NM_001001556 | GALK2 | 0.06896586 | 0.598250576 | 0.89144727 | 1 | 0.016656437 | 0.900488317 | 0.9647888 | 1 |
| 244_144 | A_23_P139418 | NM_198516 | GALNT18 | 0.251367427 | 0.096344639 | 0.500009516 | 1 | 0.20542509 | 0.139072584 | 0.4043819 | 1 |
| 14_59 | A_33_P3215768 | NM_007210 | GALNT6 | 0.344238566 | 0.024589716 | 0.318700868 | 1 | -0.286075044 | 0.07022648 | 0.2818834 | 1 |
| 160_27 | A_23_P144384 | NM_017423 | GALNT7 | -0.075168682 | 0.544995465 | 0.871327471 | 1 | 0.228677355 | 0.069045919 | 0.2793379 | 1 |
| 189_11 | A_23_P256663 | NM_003614 | GALR3 | -0.035601604 | 0.774056354 | 0.946321166 | 1 | 0.295073087 | 0.051706635 | 0.2379308 | 1 |
| 87_36 | A_23_P203406 | NM_198335 | GANAB | -0.134634085 | 0.348621679 | 0.770211834 | 1 | -0.071057819 | 0.592000609 | 0.8144146 | 1 |
| 227_9 | A_23_P13899 | NM_002046 | GAPDH | 0.288096869 | 0.207464608 | 0.654291494 | 1 | -0.485848715 | 0.135946189 | 0.4002583 | 1 |
| 48_8 | A_23_P13899 | NM_002046 | GAPDH | 0.293657532 | 0.095460127 | 0.498995654 | 1 | -0.488214829 | 0.112376321 | 0.3638159 | 1 |
| 226_35 | A_23_P13899 | NM_002046 | GAPDH | 0.309809635 | 0.154206042 | 0.593534915 | 1 | -0.513820979 | 0.111261881 | 0.362084 | 1 |
| 100_136 | A_23_P13899 | NM_002046 | GAPDH | 0.406780293 | 0.120007528 | 0.542460744 | 1 | -0.549058838 | 0.118781844 | 0.3739065 | 1 |
| 148_137 | A_23_P13899 | NM_002046 | GAPDH | 0.408482821 | 0.132636992 | 0.561910966 | 1 | -0.547021904 | 0.127491588 | 0.3884849 | 1 |
| 56_83 | A_23_P13899 | NM_002046 | GAPDH | 0.418773022 | 0.083262477 | 0.476422496 | 1 | -0.438678371 | 0.157985983 | 0.429961 | 1 |
| 294_88 | A_23_P13899 | NM_002046 | GAPDH | 0.423434071 | 0.109447326 | 0.526954706 | 1 | -0.446668037 | 0.184348926 | 0.4637334 | 1 |
| 117_141 | A_23_P13899 | NM_002046 | GAPDH | 0.453459492 | 0.109505691 | 0.526954706 | 1 | -0.523199952 | 0.149767661 | 0.4182858 | 1 |
| 6_73 | A_23_P13899 | NM_002046 | GAPDH | 0.505749567 | 0.045389947 | 0.394498032 | 1 | -0.482886471 | 0.144720533 | 0.4122776 | 1 |
| 176_93 | A_23_P13899 | NM_002046 | GAPDH | 0.636672099 | 0.044406141 | 0.392255259 | 1 | -0.479677529 | 0.176295945 | 0.4549252 | 1 |
| 79_153 | A_23_P58280 | NM_018983 | GAR1 | 0.283406704 | 0.06331277 | 0.436242178 | 1 | -0.201237319 | 0.110691678 | 0.3611689 | 1 |
| 230_8 | A_23_P66948 | NM_022751 | GAREM1 | -0.254487362 | 0.066478463 | 0.445702059 | 1 | 0.165684007 | 0.222523124 | 0.5105503 | 1 |
| 211_157 | A_24_P154948 | NM_002047 | GARS | 0.232892624 | 0.111580647 | 0.529511546 | 1 | -0.406906138 | 0.006741769 | 0.0729054 | 1 |
| 287_107 | A_23_P80098 | NM_000819 | GART | 0.081554424 | 0.576889289 | 0.883311119 | 1 | 0.096481348 | 0.497787733 | 0.752609 | 1 |
| 303_123 | A_33_P3418597 | NM_152236 | GAS2L1 | 0.095591106 | 0.468569916 | 0.836476951 | 1 | 0.069454154 | 0.521848518 | 0.7670191 | 1 |
| 91_23 | A_22_P00014325 | ENST00000425771 | GAS5 | 0.23914644 | 0.096685767 | 0.500138436 | 1 | 0.181933733 | 0.103480692 | 0.3496087 | 1 |
| 218_57 | A_33_P3326285 | NR_002578 | GAS5 | 0.593927876 | 0.002564467 | 0.154236866 | 1 | 0.280312848 | 0.042316597 | 0.2130863 | 1 |
| 250_151 | A_32_P230828 | NR_002578 | GAS5 | 0.795743262 | 0.000147652 | 0.045030562 | 1 | -0.020493712 | 0.896582402 | 0.9633493 | 1 |
| 105_116 | A_22_P00006264 | NR_044995 | GAS6-AS1 | 0.104847679 | 0.414186511 | 0.812630066 | 1 | 0.092883202 | 0.406160193 | 0.6834356 | 1 |
| 244_139 | A_33_P3405921 | NM_001286205 | GAS8 | -0.295637871 | 0.050325988 | 0.406166182 | 1 | -0.10547393 | 0.481087098 | 0.7393686 | 1 |
| 315_59 | A_24_P305467 | NM_017660 | GATAD2A | -0.06024925 | 0.725214998 | 0.936348816 | 1 | -0.349036434 | 0.036905158 | 0.1975594 | 1 |
| 140_102 | A_23_P76435 | NM_176818 | GATC | 0.315819894 | 0.029596853 | 0.339352673 | 1 | 0.067557843 | 0.581329023 | 0.8080221 | 1 |
| 125_158 | A_33_P3276329 | NM_001145064 | GATSL2 | 0.052531671 | 0.738808192 | 0.940375814 | 1 | 0.097853296 | 0.530027757 | 0.7722791 | 1 |
| 296_118 | A_33_P3227716 | NM_001037666 | GATSL3 | -0.227315275 | 0.096621946 | 0.500020625 | 1 | 0.066605066 | 0.596825136 | 0.8166049 | 1 |
| 295_28 | A_23_P121082 | NM_000158 | GBE1 | -0.28420533 | 0.059959719 | 0.428867769 | 1 | 0.173899431 | 0.201993404 | 0.4847576 | 1 |
| 275_49 | A_23_P121082 | NM_000158 | GBE1 | -0.239668808 | 0.124559269 | 0.549974996 | 1 | 0.237267949 | 0.092508349 | 0.32988 | 1 |
| 349_27 | A_23_P121082 | NM_000158 | GBE1 | -0.21298045 | 0.176261584 | 0.616374573 | 1 | 0.156124026 | 0.299850041 | 0.5905108 | 1 |
| 384_86 | A_23_P121082 | NM_000158 | GBE1 | -0.167679661 | 0.243898098 | 0.690103124 | 1 | 0.141271498 | 0.26322845 | 0.5535922 | 1 |
| 252_68 | A_23_P121082 | NM_000158 | GBE1 | -0.133815707 | 0.383547076 | 0.79219574 | 1 | 0.107213449 | 0.384793433 | 0.6656373 | 1 |
| 374_164 | A_23_P121082 | NM_000158 | GBE1 | -0.122535483 | 0.44816135 | 0.830710542 | 1 | 0.062681341 | 0.663290989 | 0.8565307 | 1 |
| 351_132 | A_23_P121082 | NM_000158 | GBE1 | -0.114030441 | 0.478026474 | 0.840908892 | 1 | 0.156966641 | 0.315772454 | 0.6031747 | 1 |
| 210_164 | A_23_P121082 | NM_000158 | GBE1 | -0.059175895 | 0.686093299 | 0.921904762 | 1 | 0.134052505 | 0.293865433 | 0.5853887 | 1 |
| 196_88 | A_23_P121082 | NM_000158 | GBE1 | -0.030698862 | 0.817836318 | 0.956101362 | 1 | 0.130616347 | 0.351583549 | 0.6366578 | 1 |
| 157_100 | A_23_P121082 | NM_000158 | GBE1 | 0.168638885 | 0.239781584 | 0.68475359 | 1 | 0.168571485 | 0.2621554 | 0.5525603 | 1 |
| 146_64 | A_23_P161237 | NM_004193 | GBF1 | 0.04930579 | 0.684238339 | 0.921139512 | 1 | 0.161676389 | 0.202999775 | 0.485558 | 1 |
| 101_56 | A_33_P3322373 | NM_001282632 | GBGT1 | 0.044242427 | 0.731341547 | 0.937364492 | 1 | 0.151268249 | 0.304237352 | 0.5935399 | 1 |
| 356_36 | A_33_P3247933 | NM_181453 | GCC2 | -0.174344234 | 0.168933456 | 0.608706095 | 1 | -0.28965056 | 0.035965742 | 0.1946615 | 1 |
| 378_119 | A_23_P90089 | NM_013976 | GCDH | 0.093467921 | 0.494384114 | 0.849672223 | 1 | -0.064458112 | 0.555591569 | 0.7907104 | 1 |
| 290_116 | A_33_P3390873 | ENST00000464444 | GCDH | 0.11771938 | 0.430340103 | 0.821617604 | 1 | -0.618849899 | 0.000166442 | 0.0070112 | 1 |
| 42_16 | A_23_P120062 | NM_003203 | GCFC2 | -0.253945642 | 0.085073687 | 0.478632531 | 1 | -0.002304218 | 0.985356434 | 0.9949293 | 1 |
| 322_99 | A_21_P0000149 | NM_001201335 | GCFC2 | 0.080576844 | 0.588023773 | 0.886650994 | 1 | 0.052326352 | 0.648638182 | 0.8473011 | 1 |
| 66_54 | A_33_P3265606 | NM_002061 | GCLM | -0.061604916 | 0.736981009 | 0.93960258 | 1 | 0.130268349 | 0.332528096 | 0.6192561 | 1 |
| 74_88 | A_23_P406105 | NM_006836 | GCN1 | -0.091928489 | 0.58762633 | 0.886487993 | 1 | 0.376249835 | 0.013762546 | 0.1115987 | 1 |
| 348_124 | A_23_P117933 | NM_004483 | GCSH | 0.314372017 | 0.031399458 | 0.344912707 | 1 | -0.043378595 | 0.727756473 | 0.8888613 | 1 |
| 276_160 | A_23_P11915 | NM_017686 | GDAP2 | -0.006347054 | 0.963843789 | 0.993394139 | 1 | 0.030188602 | 0.838901568 | 0.9393908 | 1 |
| 171_158 | A_23_P54758 | NM_016641 | GDE1 | 0.056830961 | 0.695969026 | 0.924669503 | 1 | -0.181817891 | 0.15182911 | 0.4204156 | 1 |
| 6_38 | A_23_P16523 | NM_004864 | GDF15 | 0.189033275 | 0.264258394 | 0.706894107 | 1 | 0.090732719 | 0.718515944 | 0.8841519 | 1 |
| 151_82 | A_23_P45496 | NM_001493 | GDI1 | -0.003419451 | 0.980373931 | 0.995429381 | 1 | 0.454878476 | 0.046008403 | 0.2219891 | 1 |
| 80_103 | A_23_P66872 | NM_015721 | GEMIN4 | 0.2456012 | 0.083812752 | 0.47777583 | 1 | -0.204333966 | 0.22135706 | 0.5088091 | 1 |
| 282_126 | A_23_P56567 | NM_024775 | GEMIN6 | -0.152508663 | 0.359137168 | 0.776265051 | 1 | 0.192572048 | 0.148928769 | 0.41729 | 1 |
| 311_24 | A_23_P89910 | NM_024707 | GEMIN7 | 0.018118815 | 0.89336348 | 0.975081715 | 1 | -0.119621386 | 0.339668617 | 0.6264161 | 1 |
| 229_106 | A_24_P400355 | NM_015949 | GET4 | 0.395596493 | 0.013801867 | 0.271538317 | 1 | -0.558428641 | 0.003275257 | 0.0464525 | 1 |
| 49_159 | A_24_P31003 | NM_005262 | GFER | 0.068323912 | 0.664908611 | 0.914369234 | 1 | -0.097969503 | 0.426816107 | 0.7004817 | 1 |
| 254_157 | A_23_P155288 | NM_024996 | GFM1 | 0.078636945 | 0.603211061 | 0.893532545 | 1 | -0.263264072 | 0.034767791 | 0.191777 | 1 |
| 142_20 | A_23_P213431 | NM_032380 | GFM2 | -0.221796082 | 0.117650497 | 0.53981859 | 1 | -0.010549285 | 0.932397066 | 0.9756857 | 1 |
| 278_5 | A_23_P122662 | NM_018988 | GFOD1 | -0.262392466 | 0.058176754 | 0.425190813 | 1 | 0.135668975 | 0.263652091 | 0.5540536 | 1 |
| 343_23 | A_23_P122662 | NM_018988 | GFOD1 | -0.251313101 | 0.066853654 | 0.446719314 | 1 | 0.237741445 | 0.182733585 | 0.4617957 | 1 |
| 317_64 | A_23_P122662 | NM_018988 | GFOD1 | -0.206161681 | 0.144198787 | 0.578597845 | 1 | 0.26693742 | 0.064687001 | 0.2690587 | 1 |
| 298_40 | A_23_P122662 | NM_018988 | GFOD1 | -0.193594953 | 0.193479558 | 0.638501051 | 1 | 0.370922645 | 0.035946627 | 0.1946518 | 1 |
| 97_71 | A_23_P122662 | NM_018988 | GFOD1 | -0.163640613 | 0.286069739 | 0.726263094 | 1 | 0.269534033 | 0.05086538 | 0.235619 | 1 |
| 323_104 | A_23_P122662 | NM_018988 | GFOD1 | -0.114932096 | 0.414001704 | 0.812514622 | 1 | 0.265788571 | 0.056202322 | 0.2496475 | 1 |
| 17_136 | A_23_P122662 | NM_018988 | GFOD1 | -0.054925753 | 0.719209152 | 0.934450287 | 1 | 0.186983771 | 0.252124709 | 0.5401929 | 1 |
| 65_54 | A_23_P122662 | NM_018988 | GFOD1 | -0.040804239 | 0.751231832 | 0.942061365 | 1 | 0.200429045 | 0.169115144 | 0.4461736 | 1 |
| 75_94 | A_23_P122662 | NM_018988 | GFOD1 | -0.040658343 | 0.785014175 | 0.94877096 | 1 | 0.226499292 | 0.122366842 | 0.3795532 | 1 |
| 144_89 | A_23_P122662 | NM_018988 | GFOD1 | 0.02972715 | 0.822007226 | 0.957581096 | 1 | 0.282981793 | 0.083000559 | 0.3097887 | 1 |
| 278_2 | A_23_P3574 | NM_030819 | GFOD2 | -0.022315034 | 0.875581852 | 0.970902796 | 1 | -0.120163611 | 0.309274052 | 0.5975615 | 1 |
| 243_108 | A_33_P3243153 | NM_001244710 | GFPT1 | 0.041520531 | 0.727881685 | 0.936348816 | 1 | -0.057637158 | 0.672119959 | 0.8618742 | 1 |
| 332_117 | A_23_P132294 | NM_001001560 | GGA1 | -0.169408955 | 0.23426693 | 0.680457734 | 1 | 0.027809085 | 0.84324583 | 0.9414469 | 1 |
| 15_77 | A_24_P416257 | NM_015044 | GGA2 | -0.038593493 | 0.794677377 | 0.949559569 | 1 | -0.1090347 | 0.382252886 | 0.664174 | 1 |
| 95_106 | A_23_P38181 | NM_138619 | GGA3 | 0.069202702 | 0.571448322 | 0.882279506 | 1 | -0.062779716 | 0.562783113 | 0.7958803 | 1 |
| 15_106 | A_23_P42695 | NM_024051 | GGCT | 0.20595396 | 0.226802243 | 0.671912677 | 1 | -0.492118626 | 0.004427042 | 0.0559449 | 1 |
| 42_65 | A_32_P181103 | NM_000821 | GGCX | -0.106812417 | 0.411649655 | 0.812009855 | 1 | -0.029137704 | 0.800770464 | 0.9221117 | 1 |
| 164_57 | A_33_P3308105 | NM_003878 | GGH | -0.116992824 | 0.400273618 | 0.803667875 | 1 | -0.290593073 | 0.057780662 | 0.2535541 | 1 |
| 47_20 | A_33_P3329984 | NM_152657 | GGN | -0.163515448 | 0.216717025 | 0.662917773 | 1 | -0.079038521 | 0.498574269 | 0.7529831 | 1 |
| 193_137 | A_23_P38408 | NM_024835 | GGNBP2 | 0.337972189 | 0.044078743 | 0.390521637 | 1 | -0.061609364 | 0.596200215 | 0.8165394 | 1 |
| 372_154 | A_21_P0012336 | NM_178311 | GGTLC1 | -0.279922067 | 0.045398963 | 0.394498032 | 1 | 0.068561951 | 0.690817959 | 0.8710347 | 1 |
| 10_107 | A_23_P57199 | NM_178311 | GGTLC1 | -0.252085349 | 0.120197825 | 0.542925647 | 1 | 0.14309816 | 0.363399099 | 0.6477258 | 1 |
| 73_133 | A_33_P3217073 | NM_014394 | GHITM | 0.012570015 | 0.932436932 | 0.986083644 | 1 | -0.193550221 | 0.170429796 | 0.4483799 | 1 |
| 48_151 | A_33_P3381771 | NM_024052 | GID4 | -0.226068782 | 0.224536066 | 0.668801096 | 1 | 0.092260698 | 0.4852064 | 0.742768 | 1 |
| 26_29 | A_24_P219971 | NM_017896 | GID8 | 0.228964331 | 0.173551524 | 0.613752707 | 1 | -0.126671262 | 0.399681099 | 0.6776434 | 1 |
| 155_147 | A_23_P142950 | NM_015575 | GIGYF2 | -0.190469937 | 0.20446256 | 0.650601019 | 1 | 0.705314422 | 7.33344E-05 | 0.0046665 | 0.82479 |
| 92_64 | A_23_P31109 | NM_138785 | GINM1 | -0.162716604 | 0.334855006 | 0.762359625 | 1 | 0.032665204 | 0.795937623 | 0.9196649 | 1 |
| 75_138 | A_33_P3340025 | NM_021067 | GINS1 | 0.027168259 | 0.844768736 | 0.964376494 | 1 | -0.174846429 | 0.149238966 | 0.4176389 | 1 |
| 314_52 | A_23_P118246 | NM_016095 | GINS2 | 0.080674714 | 0.597538543 | 0.89144727 | 1 | 0.122922465 | 0.284064687 | 0.5750998 | 1 |
| 32_7 | A_23_P118246 | NM_016095 | GINS2 | 0.088012105 | 0.582078757 | 0.883808109 | 1 | 0.125225026 | 0.294336649 | 0.5853942 | 1 |
| 60_148 | A_23_P118246 | NM_016095 | GINS2 | 0.173401794 | 0.273084041 | 0.715575346 | 1 | 0.074149789 | 0.540722704 | 0.7811501 | 1 |
| 12_40 | A_23_P118246 | NM_016095 | GINS2 | 0.217181355 | 0.150356439 | 0.587064789 | 1 | 0.178101433 | 0.269437838 | 0.5596246 | 1 |
| 93_105 | A_23_P118246 | NM_016095 | GINS2 | 0.235447925 | 0.088043314 | 0.484655968 | 1 | 0.051424621 | 0.714663311 | 0.8822828 | 1 |
| 137_124 | A_23_P118246 | NM_016095 | GINS2 | 0.294987712 | 0.046583588 | 0.399349089 | 1 | -0.006028655 | 0.964384538 | 0.9873043 | 1 |
| 2_70 | A_23_P118246 | NM_016095 | GINS2 | 0.306633387 | 0.089725835 | 0.487746597 | 1 | 0.072544386 | 0.660249433 | 0.8551551 | 1 |
| 275_97 | A_23_P118246 | NM_016095 | GINS2 | 0.319204788 | 0.02722761 | 0.330694664 | 1 | 0.059720942 | 0.664642988 | 0.8568592 | 1 |
| 246_144 | A_23_P118246 | NM_016095 | GINS2 | 0.340035921 | 0.04997833 | 0.406002234 | 1 | -0.045437144 | 0.68437749 | 0.8683976 | 1 |
| 204_98 | A_23_P118246 | NM_016095 | GINS2 | 0.542590587 | 0.00360684 | 0.176459918 | 1 | 0.052302013 | 0.807849862 | 0.9250547 | 1 |
| 193_26 | A_23_P152136 | NM_022770 | GINS3 | -0.067706801 | 0.68708123 | 0.922029409 | 1 | -0.075254279 | 0.511749633 | 0.7605243 | 1 |
| 197_68 | A_33_P3340040 | NM_032336 | GINS4 | -0.073072923 | 0.618491439 | 0.89867047 | 1 | 0.240768331 | 0.070345623 | 0.2820635 | 1 |
| 184_126 | A_33_P3390102 | NM_005716 | GIPC1 | -0.239383 | 0.107900552 | 0.523891801 | 1 | -0.20684295 | 0.13045362 | 0.3914684 | 1 |
| 40_2 | A_23_P335848 | NM_057169 | GIT2 | -0.310742837 | 0.060475783 | 0.430408929 | 1 | 0.09897634 | 0.437526226 | 0.7090573 | 1 |
| 118_14 | A_33_P3422822 | NM_020435 | GJC2 | -0.009244054 | 0.944594098 | 0.989683091 | 1 | 0.551591535 | 0.014205146 | 0.1130681 | 1 |
| 281_50 | A_33_P3353496 | NM_153368 | GJD4 | -0.089148958 | 0.502238347 | 0.853951304 | 1 | 0.061203514 | 0.584215006 | 0.8098234 | 1 |
| 319_46 | A_23_P45475 | NM_000169 | GLA | -0.253102579 | 0.089936512 | 0.48830708 | 1 | 0.338629822 | 0.038588374 | 0.2020377 | 1 |
| 362_138 | A_23_P61531 | NM_000404 | GLB1 | -0.066592848 | 0.614620362 | 0.897816866 | 1 | 0.160839422 | 0.184512053 | 0.4637334 | 1 |
| 31_156 | A_23_P206510 | NM_012201 | GLG1 | 0.069827805 | 0.667850913 | 0.916365018 | 1 | 0.148774335 | 0.218788966 | 0.5052005 | 1 |
| 222_2 | A_33_P3285715 | ENST00000523812 | GLI4 | -0.101210608 | 0.483580802 | 0.843617547 | 1 | 0.316970874 | 0.021173986 | 0.1422665 | 1 |
| 148_83 | A_33_P3252794 | NM_138465 | GLI4 | 0.147656397 | 0.248604262 | 0.694131818 | 1 | 0.037603434 | 0.772900289 | 0.9091946 | 1 |
| 308_72 | A_33_P3226439 | NR_126046 | GLIDR | -0.246958693 | 0.093707012 | 0.496537113 | 1 | 0.128215004 | 0.396355497 | 0.6759357 | 1 |
| 210_9 | A_23_P78268 | NM_016080 | GLOD4 | 0.052005766 | 0.711428005 | 0.930633257 | 1 | 0.073132033 | 0.566626931 | 0.798763 | 1 |
| 68_6 | A_23_P69908 | NM_002064 | GLRX | -0.219036187 | 0.281145051 | 0.72209762 | 1 | 0.279595135 | 0.057324835 | 0.2525391 | 1 |
| 363_48 | A_23_P160503 | NM_016066 | GLRX2 | 0.012353199 | 0.918145643 | 0.981353558 | 1 | -0.040067859 | 0.751385106 | 0.9007747 | 1 |
| 108_7 | A_24_P381029 | NM_006541 | GLRX3 | 0.025576562 | 0.841150723 | 0.963184063 | 1 | -0.225577074 | 0.099692274 | 0.3434127 | 1 |
| 360_79 | A_23_P65370 | NM_016417 | GLRX5 | 0.236978371 | 0.145375449 | 0.580482169 | 1 | 0.591904855 | 0.003181537 | 0.0458166 | 1 |
| 366_106 | A_23_P39766 | NM_014905 | GLS | 0.118275316 | 0.379388581 | 0.790179752 | 1 | 0.586575201 | 0.000931279 | 0.0214194 | 1 |
| 92_39 | A_33_P3275290 | NM_001010983 | GLT8D1 | 0.045237153 | 0.765669608 | 0.944948837 | 1 | -0.051113032 | 0.686948746 | 0.8689581 | 1 |
| 102_64 | A_23_P132669 | NM_001010983 | GLT8D1 | 0.122169185 | 0.45746772 | 0.83358833 | 1 | -0.172958143 | 0.195273507 | 0.4773651 | 1 |
| 199_32 | A_33_P3393694 | NM_015710 | GLTSCR2 | -0.009673083 | 0.937169312 | 0.987424023 | 1 | -0.02376433 | 0.862472664 | 0.9501724 | 1 |
| 107_84 | A_23_P138665 | NM_005271 | GLUD1 | 0.052826928 | 0.671080491 | 0.916686207 | 1 | 0.179545017 | 0.137759221 | 0.4027497 | 1 |
| 268_28 | A_33_P3295148 | NR_048575 | GLUD1P3 | -0.145166506 | 0.310897564 | 0.74326753 | 1 | 0.085929603 | 0.423900331 | 0.6991498 | 1 |
| 175_2 | A_21_P0010921 | NR_111968 | GLUD1P7 | -0.129908932 | 0.452824996 | 0.832651186 | 1 | 0.022372156 | 0.860742358 | 0.9501054 | 1 |
| 97_97 | A_33_P3405334 | NM_000405 | GM2A | -0.661824599 | 0.006233358 | 0.218832172 | 1 | 1.35460691 | 3.16704E-06 | 0.0010909 | 0.03562 |
| 229_19 | A_23_P137586 | NM_006582 | GMEB1 | -0.21845623 | 0.115081995 | 0.536726212 | 1 | -0.045688156 | 0.715370923 | 0.8827861 | 1 |
| 326_44 | A_24_P172768 | NM_004124 | GMFB | 0.339637385 | 0.019654663 | 0.306116626 | 1 | -0.210530927 | 0.120538102 | 0.3765811 | 1 |
| 27_113 | A_23_P56228 | NM_016573 | GMIP | -0.100341254 | 0.439403399 | 0.827404459 | 1 | -0.14736634 | 0.23209673 | 0.5198175 | 1 |
| 159_61 | A_23_P19712 | NM_015895 | GMNN | 0.383001753 | 0.032105661 | 0.347345614 | 1 | -0.318047048 | 0.027472043 | 0.1664752 | 1 |
| 281_159 | A_24_P56467 | NM_001002000 | GMPR2 | -0.371183686 | 0.011125902 | 0.256586468 | 1 | 0.140407614 | 0.314202095 | 0.6015453 | 1 |
| 364_119 | A_23_P21033 | NM_003875 | GMPS | -0.100533245 | 0.502808047 | 0.854163663 | 1 | 0.155714798 | 0.183900897 | 0.4632326 | 1 |
| 20_18 | A_24_P98086 | NM_007353 | GNA12 | 0.170321662 | 0.181379469 | 0.622415463 | 1 | -0.107360191 | 0.371225473 | 0.6550318 | 1 |
| 189_46 | A_24_P941441 | NM_006572 | GNA13 | 0.108406201 | 0.377921216 | 0.790086881 | 1 | -0.302104551 | 0.023425234 | 0.1522911 | 1 |
| 55_109 | A_33_P3585268 | NM_001282619 | GNAI2 | -0.367325223 | 0.024677313 | 0.318700868 | 1 | 0.0343499 | 0.799414541 | 0.9212674 | 1 |
| 96_115 | A_33_P3419334 | NM_006496 | GNAI3 | -0.030499243 | 0.815994257 | 0.956087529 | 1 | -0.190894807 | 0.120720628 | 0.3768373 | 1 |
| 147_119 | A_24_P273666 | NM_001077489 | GNAS | 0.011301169 | 0.946395054 | 0.99002169 | 1 | 0.018180414 | 0.934608674 | 0.9765462 | 1 |
| 94_105 | A_24_P418809 | NM_001077489 | GNAS | 0.313909812 | 0.169414805 | 0.608706095 | 1 | -0.176627651 | 0.607093356 | 0.8206139 | 1 |
| 37_110 | A_23_P22926 | NM_002074 | GNB1 | 0.127382916 | 0.473841463 | 0.839074131 | 1 | -0.14625631 | 0.291386337 | 0.5828245 | 1 |
| 339_27 | A_23_P218751 | NM_053004 | GNB1L | -0.165913114 | 0.294598919 | 0.732574292 | 1 | 0.244782785 | 0.043823758 | 0.2164628 | 1 |
| 116_61 | A_23_P216489 | NM_005476 | GNE | -0.049876816 | 0.712738038 | 0.931606735 | 1 | 0.509673084 | 0.007386051 | 0.0771974 | 1 |
| 363_54 | A_23_P112251 | NM_001017998 | GNG10 | -0.231502922 | 0.104028793 | 0.516834727 | 1 | 0.256559135 | 0.089727244 | 0.3231483 | 1 |
| 153_41 | A_23_P148513 | NM_005274 | GNG5 | 0.120203666 | 0.440483089 | 0.827546746 | 1 | -0.071783815 | 0.668543074 | 0.8598175 | 1 |
| 11_129 | A_23_P34578 | NM_013285 | GNL2 | 0.072651141 | 0.695510059 | 0.924602922 | 1 | -0.304262915 | 0.027911163 | 0.1676011 | 1 |
| 225_64 | A_23_P41025 | NM_014366 | GNL3 | 0.318900563 | 0.034578721 | 0.358580478 | 1 | -0.162425132 | 0.251797106 | 0.5396785 | 1 |
| 264_69 | A_24_P100351 | NM_019067 | GNL3L | -0.047045699 | 0.742370623 | 0.941128659 | 1 | -0.848814575 | 2.40143E-05 | 0.0025668 | 0.27009 |
| 366_46 | A_23_P85777 | NM_014236 | GNPAT | 0.066901194 | 0.58191055 | 0.883808109 | 1 | -0.253280442 | 0.079191046 | 0.3034622 | 1 |
| 235_125 | A_23_P429184 | NM_198066 | GNPNAT1 | 0.373550615 | 0.089260753 | 0.486413374 | 1 | -0.017875936 | 0.883094427 | 0.9596293 | 1 |
| 37_65 | A_24_P281975 | NM_024312 | GNPTAB | -0.176968055 | 0.186445903 | 0.629687828 | 1 | 0.123611917 | 0.316555318 | 0.6036347 | 1 |
| 32_67 | A_23_P14886 | NM_032520 | GNPTG | -0.037392167 | 0.802062919 | 0.952164507 | 1 | -0.286172763 | 0.080825895 | 0.3065932 | 1 |
| 29_108 | A_33_P3261167 | NM_002076 | GNS | -0.208923523 | 0.106927619 | 0.522238202 | 1 | 0.117784669 | 0.420175147 | 0.6961362 | 1 |
| 94_19 | A_33_P3326733 | NR_120609 | GOLGA2P6 | -0.146687388 | 0.338986625 | 0.764199026 | 1 | 0.112014408 | 0.401093631 | 0.6788713 | 1 |
| 14_108 | A_23_P398275 | NR_027001 | GOLGA2P7 | -0.112746876 | 0.412788277 | 0.812009855 | 1 | -0.274342558 | 0.072052316 | 0.2862495 | 1 |
| 327_93 | A_23_P129103 | NR_027001 | GOLGA2P7 | -0.086359555 | 0.515601713 | 0.859949613 | 1 | -0.199596609 | 0.128282338 | 0.389219 | 1 |
| 205_153 | A_23_P203819 | NM_005895 | GOLGA3 | 0.401652882 | 0.024208901 | 0.318480383 | 1 | -0.262777074 | 0.03849427 | 0.2020377 | 1 |
| 97_17 | A_33_P3239102 | A_33_P3239102 | GOLGA6L10 | -0.059678257 | 0.790137826 | 0.94877096 | 1 | 0.098764321 | 0.442958454 | 0.7127439 | 1 |
| 215_53 | A_33_P3243878 | NM_001267536 | GOLGA6L4 | -0.334205874 | 0.051520773 | 0.40994517 | 1 | 0.037997108 | 0.764091387 | 0.9059793 | 1 |
| 360_113 | A_23_P71440 | NM_001002296 | GOLGA7 | 0.20495344 | 0.163682667 | 0.603505525 | 1 | 0.024846759 | 0.861386327 | 0.9501724 | 1 |
| 292_61 | A_24_P191664 | NM_014498 | GOLIM4 | -0.089263104 | 0.498467377 | 0.851182961 | 1 | -0.263891329 | 0.03799187 | 0.2006938 | 1 |
| 11_140 | A_24_P345377 | NM_018178 | GOLPH3L | -0.110543779 | 0.495766244 | 0.850005476 | 1 | 0.15453387 | 0.324514872 | 0.6109806 | 1 |
| 199_141 | A_23_P97250 | NM_001282860 | GON4L | -0.083571571 | 0.61862139 | 0.89867047 | 1 | 0.441139173 | 0.003289703 | 0.0465986 | 1 |
| 365_114 | A_33_P3221119 | NM_001282861 | GON4L | -0.027291747 | 0.840802833 | 0.963108614 | 1 | -0.037765505 | 0.775366978 | 0.9103379 | 1 |
| 24_13 | A_23_P58877 | NM_020399 | GOPC | 0.227469702 | 0.132254554 | 0.561823086 | 1 | -0.15408613 | 0.282957827 | 0.5741343 | 1 |
| 238_140 | A_23_P165494 | NM_015530 | GORASP2 | 0.082589705 | 0.560115468 | 0.87771117 | 1 | 0.437669522 | 0.001540196 | 0.0283977 | 1 |
| 49_101 | A_23_P252641 | NM_001007024 | GOSR1 | -0.168702575 | 0.264788283 | 0.707490253 | 1 | 0.050465614 | 0.680233578 | 0.8665293 | 1 |
| 11_106 | A_33_P3279431 | NM_001007024 | GOSR1 | 0.098454501 | 0.49846817 | 0.851182961 | 1 | 0.033879136 | 0.831803727 | 0.9362787 | 1 |
| 282_160 | A_24_P108242 | NM_004287 | GOSR2 | -0.133536991 | 0.31556938 | 0.74636302 | 1 | 0.062918281 | 0.586952459 | 0.8110447 | 1 |
| 37_43 | A_33_P3279441 | NM_001012511 | GOSR2 | 0.072980177 | 0.588056442 | 0.886650994 | 1 | -0.203222949 | 0.095474773 | 0.3358789 | 1 |
| 185_162 | A_23_P63825 | NM_002079 | GOT1 | 0.034344018 | 0.832377148 | 0.961349486 | 1 | -0.224599067 | 0.204937871 | 0.4876514 | 1 |
| 72_100 | A_33_P3414482 | NM_002080 | GOT2 | 0.134611846 | 0.433779379 | 0.823905778 | 1 | 0.012976139 | 0.908417187 | 0.9677558 | 1 |
| 364_76 | A_33_P3265030 | NM_000407 | GP1BB | -0.027355886 | 0.8622313 | 0.967548056 | 1 | -0.050435009 | 0.665143366 | 0.8573078 | 1 |
| 121_3 | A_24_P227069 | NM_020918 | GPAM | -0.086158902 | 0.512029841 | 0.85794362 | 1 | 0.036229152 | 0.7572235 | 0.9026672 | 1 |
| 179_81 | A_23_P133923 | NM_033177 | GPANK1 | 0.282476524 | 0.042231461 | 0.386814779 | 1 | -0.070795313 | 0.605473892 | 0.8203033 | 1 |
| 264_108 | A_33_P3415551 | NM_207328 | GPAT2 | -0.579700685 | 0.001496424 | 0.124859164 | 1 | -1.145056218 | 0.000661761 | 0.0174449 | 1 |
| 198_55 | A_23_P68240 | NM_207328 | GPAT2 | -0.502574815 | 0.008950078 | 0.240080283 | 1 | -0.877109647 | 0.001072492 | 0.0231968 | 1 |
| 61_146 | A_23_P69810 | NM_032717 | GPAT3 | 0.004607239 | 0.973433456 | 0.994783177 | 1 | -0.233322072 | 0.093626743 | 0.3319735 | 1 |
| 151_43 | A_23_P339633 | NM_174931 | GPATCH11 | -0.127158879 | 0.313873179 | 0.74498319 | 1 | 0.037590381 | 0.736666088 | 0.8931957 | 1 |
| 130_23 | A_23_P97265 | NM_182679 | GPATCH4 | 0.143371575 | 0.329940781 | 0.760452185 | 1 | 0.019794078 | 0.896556576 | 0.9633493 | 1 |
| 326_147 | A_33_P3211263 | NM_182679 | GPATCH4 | 0.246400058 | 0.091390921 | 0.491100222 | 1 | -0.243600428 | 0.045450227 | 0.2207162 | 1 |
| 371_14 | A_23_P364465 | NM_022913 | GPBP1 | -0.376984078 | 0.010764338 | 0.255742129 | 1 | 0.228862117 | 0.129107809 | 0.390238 | 1 |
| 67_144 | A_23_P209904 | NM_002081 | GPC1 | -0.08587074 | 0.507858823 | 0.855904935 | 1 | 0.213342455 | 0.188207472 | 0.4689818 | 1 |
| 361_50 | A_23_P251795 | ENST00000471050 | GPC2 | -0.092510685 | 0.523271879 | 0.863653225 | 1 | -0.07287403 | 0.569414028 | 0.8003249 | 1 |
| 149_147 | A_23_P91350 | NM_019593 | GPCPD1 | 0.193291121 | 0.207110133 | 0.654291494 | 1 | -0.287573619 | 0.034436342 | 0.1904868 | 1 |
| 155_22 | A_23_P318284 | NM_015141 | GPD1L | 0.018196365 | 0.884080993 | 0.972822545 | 1 | 0.138101298 | 0.248257298 | 0.5355889 | 1 |
| 266_77 | A_33_P3354569 | NM_001083112 | GPD2 | -0.061240034 | 0.624960263 | 0.899887301 | 1 | 0.126188455 | 0.398502071 | 0.6770186 | 1 |
| 241_18 | A_33_P3345816 | NM_001039966 | GPER1 | 0.375024975 | 0.03670109 | 0.369070022 | 1 | -0.22709586 | 0.100058977 | 0.3434127 | 1 |
| 71_59 | A_23_P8640 | NM_001039966 | GPER1 | 0.613189112 | 0.024477487 | 0.318700868 | 1 | -0.123489509 | 0.346789123 | 0.6326581 | 1 |
| 223_35 | A_23_P336678 | NM_145171 | GPHB5 | 0.061697922 | 0.631274715 | 0.901004617 | 1 | -0.014367711 | 0.898054736 | 0.9639647 | 1 |
| 118_57 | A_33_P3414422 | NM_020806 | GPHN | -0.098846187 | 0.448579403 | 0.830710542 | 1 | 0.119762144 | 0.307160841 | 0.5958327 | 1 |
| 88_164 | A_24_P108451 | NM_000175 | GPI | 0.19612705 | 0.164911998 | 0.60427748 | 1 | 0.157203582 | 0.278599002 | 0.5696383 | 1 |
| 192_3 | A_23_P114155 | NM_015698 | GPKOW | -0.359474264 | 0.012916271 | 0.267459687 | 1 | -0.693298255 | 0.000169073 | 0.0070606 | 1 |
| 345_30 | A_23_P114155 | NM_015698 | GPKOW | -0.212197536 | 0.130073446 | 0.559492585 | 1 | -0.633344521 | 0.000590078 | 0.0162073 | 1 |
| 28_9 | A_23_P114155 | NM_015698 | GPKOW | -0.203864225 | 0.147318655 | 0.583052583 | 1 | -0.696235606 | 0.000210871 | 0.00815 | 1 |
| 67_76 | A_23_P114155 | NM_015698 | GPKOW | -0.175986375 | 0.172770224 | 0.612857861 | 1 | -0.70518148 | 0.000137789 | 0.0064571 | 1 |
| 65_149 | A_23_P114155 | NM_015698 | GPKOW | -0.174966193 | 0.167093513 | 0.607621843 | 1 | -0.736775515 | 8.83967E-05 | 0.0051781 | 0.9942 |
| 344_129 | A_23_P114155 | NM_015698 | GPKOW | -0.076268221 | 0.557434103 | 0.87699568 | 1 | -0.673903814 | 0.000314363 | 0.0106495 | 1 |
| 124_117 | A_23_P114155 | NM_015698 | GPKOW | -0.066440851 | 0.581909147 | 0.883808109 | 1 | -0.835196783 | 3.33625E-05 | 0.0030018 | 0.37523 |
| 326_133 | A_23_P114155 | NM_015698 | GPKOW | -0.05985331 | 0.630635382 | 0.901004617 | 1 | -0.761949836 | 7.41828E-05 | 0.0046862 | 0.83433 |
| 101_98 | A_23_P114155 | NM_015698 | GPKOW | -0.008894454 | 0.940831735 | 0.988335297 | 1 | -0.7418022 | 0.000143577 | 0.0065911 | 1 |
| 170_102 | A_23_P114155 | NM_015698 | GPKOW | 0.149323556 | 0.274357714 | 0.71644709 | 1 | -0.741305665 | 0.001141342 | 0.0241745 | 1 |
| 369_91 | A_23_P17144 | NM_007266 | GPN1 | 0.07151739 | 0.620799775 | 0.89867047 | 1 | 0.077858066 | 0.475768461 | 0.7355282 | 1 |
| 89_73 | A_33_P3291394 | NM_007266 | GPN1 | 0.365410485 | 0.03412666 | 0.355727847 | 1 | -0.238432558 | 0.138964086 | 0.4043801 | 1 |
| 96_14 | A_23_P138025 | NM_018066 | GPN2 | -0.039223127 | 0.827400286 | 0.959690637 | 1 | 0.262027647 | 0.054056607 | 0.2441095 | 1 |
| 161_111 | A_33_P3389837 | NM_001164372 | GPN3 | 0.261182941 | 0.086453482 | 0.480683325 | 1 | 0.269932586 | 0.103389457 | 0.3494054 | 1 |
| 373_151 | A_24_P37887 | NM_199243 | GPR150 | -0.002491563 | 0.984886947 | 0.995672988 | 1 | 0.148603373 | 0.286071873 | 0.5772057 | 1 |
[truncated: 972,450 more chars]
