# Supplementary material for: Tumor endothelial marker 8 promotes cancer progression and metastasis
Source: Oncotarget. 2018 Jul 10;9(53):30173–88. doi: 10.18632/oncotarget.25734 (PMC6059023; doi:10.18632/oncotarget.25734)
Supplement: Supplementary file 3 [file oncotarget-09-30173-s003.docx]

| **Supplementary Table 2**. Functional enrichment analysis of co-clustered genes | | | | | | | |
| --- | --- | --- | --- | --- | --- | --- | --- |
| **Cluster** | **Category** | **Term Accession** | **Term Name** | **Genes** | **Count (genes per term)** | **Fold enrichment** | **Bonferroni corrected P value** |
| 1 | GO biological process | GO:0006396 | RNA processing | HNRNPA1L2, POLR2G, FIP1L1, ELAC2, STRAP, CWC15, INTS1, HNRNPM, DCAF13, TYW1, WDR77, SBDSP1, RPL11, RPL10A, SNRPA1, EXOSC7, EFTUD2, HEATR1, EXOSC1, RBMX, FBL, RPS7, RSRC1, ZRANB2, ERN1, RBM39, SNRPE, SSU72 | 28 | 3.35 | 7.76E-05 |
| 3 | GO biological process | GO:0006412 | translation | EIF6, RPL18, TUFM, MRPS17, MRPL41, EIF5B, MRPL11, EIF3D, MRPL12, EIF3B, MRPL36, RPL3, MRPL18, EIF3I, MRPL32, EIF2B4, RPS24, MRPL3, DARS, AIMP2, PAIP1, MRPS24, GARS, MRRF, RPS5, RPL28, MRPL24, RSL1D1, EIF4G2, RPS19, RPL18A, MRPL28, COG8, MRPL27, EIF4H, MTRF1, MRPL49, RPS13, RPS11, RPL19P12, EEF1D | 41 | 3.71 | 2.47E-09 |
| 3 | GO biological process | GO:0006396 | RNA processing | RP9, SYNCRIP, WTAP, WBP4, YBX1, SBDS, TARDBP, PRMT5, LSM5, LSM3, DEDD2, PABPN1, PRPF31, EXOSC8, TRMT61A, SRPK1, HNRNPU, SMN1, RSL1D1, WDR83, PA2G4, RPS19, CELF6, SNRPB, CPSF6, SNRPA, CPSF4, CPSF3, POLR2F, PUSL1, BOP1, SF3B5, SF3B2, RRAGC, SRRT, PPP2CA, METTL1, HNRNPD, DHX16, PPWD1, RPS24, TSEN54, DDX1, SNW1, CASC3, INTS10, RPF1, DDX56, GTF2F1, GTF2F2, HNRNPH1 | 51 | 2.79 | 1.39E-07 |
| 3 | GO biological process | GO:0008380 | RNA splicing | POLR2F, RP9, SYNCRIP, SF3B5, WTAP, WBP4, YBX1, SF3B2, RRAGC, PRMT5, TARDBP, PPP2CA, HNRNPD, LSM5, DHX16, LSM3, PPWD1, PABPN1, PRPF31, TSEN54, DDX1, SNW1, CASC3, SRPK1, SMN1, HNRNPU, WDR83, GTF2F1, GTF2F2, SNRPB, SNRPA, HNRNPH1, CPSF3 | 33 | 3.48 | 2.81E-06 |
| 3 | GO biological process | GO:0006397 | mRNA processing | POLR2F, SYNCRIP, SF3B5, WTAP, WBP4, YBX1, SF3B2, PRMT5, TARDBP, HNRNPD, LSM5, DHX16, LSM3, PPWD1, PABPN1, PRPF31, TSEN54, DDX1, SNW1, CASC3, SRPK1, SMN1, HNRNPU, WDR83, CELF6, GTF2F1, GTF2F2, SNRPB, CPSF6, SNRPA, CPSF4, HNRNPH1, CPSF3 | 33 | 3.08 | 5.61E-05 |
| 3 | GO biological process | GO:0016071 | mRNA metabolic process | POLR2F, SYNCRIP, HSPA1B, SF3B5, WTAP, WBP4, YBX1, SF3B2, PRMT5, TARDBP, HNRNPD, LSM5, DHX16, LSM3, PPWD1, PABPN1, PRPF31, TSEN54, PAIP1, DDX1, SNW1, CASC3, SRPK1, SMN1, HNRNPU, WDR83, CELF6, GTF2F1, GTF2F2, SNRPB, CPSF6, SNRPA, CPSF4, HNRNPH1, CPSF3 | 35 | 2.83 | 1.55E-04 |
| 3 | GO biological process | GO:0010608 | posttranscriptional regulation of gene expression | METAP1, PAIP2, PAIP1, DDX1, IREB2, EIF5B, SYNCRIP, CASC3, CDK4, RPS5, HNRNPU, YBX1, AURKAPS1, EIF4G2, SRRT, PA2G4, EIF3B, MTRF1, EIF4H, HNRNPD, NGDN, DNAJA3, EIF2B4, FBXO7 | 24 | 3.40 | 1.06E-03 |
| 3 | GO biological process | GO:0000278 | mitotic cell cycle | KIF22, PRC1, CAMK2G, PSMA7, AURKAPS1, PSMB5, PSMB6, TARDBP, PSMD3, NUP37, NUDC, CKAP5, DSN1, TPX2, PAPD7, TBRG4, NUSAP1, UBE2I, CDC20, PMF1, DCTN3, CDK4, CDKN3, PPP1CB, MIS12, PSMA2, PSMA1, PSMD14, PPM1D, CUL4A, PSMA3, PSME3, AKAP8 | 33 | 2.67 | 1.46E-03 |
| 3 | GO biological process | GO:0007049 | cell cycle | KIF22, MRPL41, PRC1, JAG2, WTAP, SBDS, TARDBP, VPS4B, STAG3, PSMD3, VPS4A, NUP37, ARL2, DSN1, TPX2, PAPD7, TBRG4, NUSAP1, UBE2I, MCM2, DCTN3, CDK4, PPP1CB, PSMA2, EIF4G2, PSMA1, PA2G4, PPM1D, PSMA3, PSME3, AKAP8, CAMK2G, ITGAE, PSMA7, AURKAPS1, PSMB5, GADD45GIP1, PSMB6, THAP1, NUDC, GPS1, CKAP5, PCNP, CDC20, PMF1, CDKN3, MIS12, NAE1, PSMD14, CUL4A, DMTF1, CKS2, CHAF1A | 53 | 2.04 | 1.97E-03 |
| 3 | GO biological process | GO:0000377 | RNA splicing, via transesterification reactions with bulged adenosine as nucleophile | PABPN1, PRPF31, POLR2F, DDX1, SNW1, SF3B5, HNRNPU, SMN1, YBX1, SF3B2, WDR83, GTF2F1, PRMT5, GTF2F2, HNRNPD, SNRPB, SNRPA, HNRNPH1, CPSF3 | 19 | 3.72 | 6.05E-03 |
| 3 | GO biological process | GO:0000375 | RNA splicing, via transesterification reactions | PABPN1, PRPF31, POLR2F, DDX1, SNW1, SF3B5, HNRNPU, SMN1, YBX1, SF3B2, WDR83, GTF2F1, PRMT5, GTF2F2, HNRNPD, SNRPB, SNRPA, HNRNPH1, CPSF3 | 19 | 3.72 | 6.05E-03 |
| 3 | GO biological process | GO:0000398 | nuclear mRNA splicing, via spliceosome | PABPN1, PRPF31, POLR2F, DDX1, SNW1, SF3B5, HNRNPU, SMN1, YBX1, SF3B2, WDR83, GTF2F1, PRMT5, GTF2F2, HNRNPD, SNRPB, SNRPA, HNRNPH1, CPSF3 | 19 | 3.72 | 6.05E-03 |
| 3 | GO biological process | GO:0006511 | ubiquitin-dependent protein catabolic process | SYVN1, DDB1, PCNP, UBE2I, CDC20, UBE2L3, PSMA7, PSMB5, PSMA2, PSMA1, UBE2D3, ARIH2, PSMD14, PSMB6, UBE2D2, CUL4A, UBE2K, PSMA3, USP38, PSMD3, PSME3, AMFR, FBXO7 | 23 | 2.84 | 3.55E-02 |
| 4 | GO biological process | GO:0006412 | translation | ABCF1, RPL17, MRPS15, MRPS12, RPL15, RPL35, RPS2, MRPL20, RPS3, WARS, MRPL10, MRPL15, EIF3G, RPL6, RPS3A, EIF3E, RPLP0, TRMT6, RPSAP58, EIF3K, EIF3L, RPL5, RPL7A, RPL4, EIF2B2, EIF3M, MRPL34, EIF1AD, RPS9, MRPS7, RPSAP9, GTF2B, EIF2B1, RPL29, EIF4E, EEF1E1, RPS15, FARSA, UBB | 38 | 5.04 | 1.11E-12 |
| 4 | GO biological process | GO:0006396 | RNA processing | PUS3, GAR1, SNRPD3, POLR2I, SNRPB2, LSM7, ERI1, SKIV2L2, IVNS1ABP, POLR2C, KIN, RBM8A, PCBP1, TRMT6, PRPF8, RPL5, DDX20, DDX41, IMP4, GEMIN4, MTO1, CCAR1, KHDRBS1, EMG1, SF1, EXOSC2, RRP8, PRPF3, RRP9, MRM1, PRPF6, SLBP, EIF4A3, HNRNPH3, RPS15, KHSRP, LSM10, NOP56, RBM14, SNRPF, SNRNP25, DDX51 | 42 | 3.37 | 2.24E-08 |
| 4 | GO biological process | GO:0006413 | translational initiation | EIF4E, EIF3G, RPS3A, EIF1AD, TRMT6, EIF3E, EIF3K, EIF3L, EIF2B2, EIF2B1, GTF2B, EIF3M, RPS3 | 13 | 12.69 | 3.68E-07 |
| 4 | GO biological process | GO:0022613 | ribonucleoprotein complex biogenesis | EMG1, GAR1, SNRPD3, SF1, EXOSC2, ERI1, RRP8, RRP9, PRPF6, EIF4A3, RPLP0, RPS15, NPM1, RPL5, AATF, DDX20, RPL7A, NOP56, SNRPF, IMP4, GEMIN4, DDX51 | 22 | 5.37 | 1.29E-06 |
| 4 | GO biological process | GO:0006414 | translational elongation | RPL17, RPL15, RPL35, RPS9, RPSAP9, RPS2, RPS3, RPL29, RPL6, RPS3A, RPLP0, RPSAP58, RPS15, RPL5, UBB, RPL7A, RPL4 | 16 | 6.96 | 1.32E-05 |
| 4 | GO biological process | GO:0042254 | ribosome biogenesis | EMG1, GAR1, EXOSC2, ERI1, RRP8, RRP9, EIF4A3, RPLP0, RPS15, NPM1, RPL5, AATF, RPL7A, NOP56, IMP4, GEMIN4, DDX51 | 17 | 6.12 | 2.62E-05 |
| 4 | GO biological process | GO:0006397 | mRNA processing | KHDRBS1, SNRPD3, POLR2I, SNRPB2, LSM7, SF1, SKIV2L2, PRPF3, POLR2C, KIN, PRPF6, SLBP, EIF4A3, HNRNPH3, RBM8A, PCBP1, PRPF8, KHSRP, LSM10, DDX20, RBM14, DDX41, SNRPF, GEMIN4, SNRNP25, CCAR1 | 26 | 3.56 | 1.33E-04 |
| 4 | GO biological process | GO:0016071 | mRNA metabolic process | SNRPD3, SNRPB2, LSM7, POLR2I, SKIV2L2, KIN, POLR2C, DCPS, RBM8A, PRPF8, PCBP1, EIF3E, DDX20, DDX41, GEMIN4, CCAR1, KHDRBS1, SF1, PRPF3, PRPF6, SLBP, EIF4A3, HNRNPH3, KHSRP, LSM10, SNRPF, RBM14, SNRNP25 | 28 | 3.32 | 1.52E-04 |
| 4 | GO biological process | GO:0008380 | RNA splicing | SNRPD3, LSM7, POLR2I, SNRPB2, SF1, SKIV2L2, PRPF3, IVNS1ABP, POLR2C, PRPF6, EIF4A3, HNRNPH3, RBM8A, PRPF8, PCBP1, KHSRP, LSM10, DDX20, SNRPF, DDX41, RBM14, SNRNP25, GEMIN4, CCAR1 | 24 | 3.71 | 2.22E-04 |
| 4 | GO biological process | GO:0006364 | rRNA processing | EIF4A3, GAR1, EMG1, RPS15, EXOSC2, ERI1, RPL5, RRP8, RRP9, NOP56, IMP4, GEMIN4, DDX51 | 13 | 6.21 | 1.79E-03 |
| 4 | GO biological process | GO:0016072 | rRNA metabolic process | EIF4A3, GAR1, EMG1, RPS15, EXOSC2, ERI1, RPL5, RRP8, RRP9, NOP56, IMP4, GEMIN4, DDX51 | 13 | 5.95 | 2.84E-03 |
| 4 | GO biological process | GO:0000375 | RNA splicing, via transesterification reactions | SNRPD3, LSM7, SNRPB2, POLR2I, SF1, PRPF3, POLR2C, PRPF6, HNRNPH3, RBM8A, PCBP1, PRPF8, DDX20, SNRPF, GEMIN4, CCAR1 | 16 | 4.59 | 3.40E-03 |
| 4 | GO biological process | GO:0000398 | nuclear mRNA splicing, via spliceosome | SNRPD3, LSM7, SNRPB2, POLR2I, SF1, PRPF3, POLR2C, PRPF6, HNRNPH3, RBM8A, PCBP1, PRPF8, DDX20, SNRPF, GEMIN4, CCAR1 | 16 | 4.59 | 3.40E-03 |
| 4 | GO biological process | GO:0000377 | RNA splicing, via transesterification reactions with bulged adenosine as nucleophile | SNRPD3, LSM7, SNRPB2, POLR2I, SF1, PRPF3, POLR2C, PRPF6, HNRNPH3, RBM8A, PCBP1, PRPF8, DDX20, SNRPF, GEMIN4, CCAR1 | 16 | 4.59 | 3.40E-03 |
| 4 | GO biological process | GO:0032269 | negative regulation of cellular protein metabolic process | HSP90AB1, ANAPC1, SRP14, FKBP1A, ANAPC10, EIF2B1, EIF4A3, PSMB4, PSMA6, PSMD11, PSMA5, EIF3E, PSMC2, UBB, PSMD6, PSMD7, SRP9 | 17 | 4.15 | 5.67E-03 |
| 4 | GO biological process | GO:0051248 | negative regulation of protein metabolic process | HSP90AB1, ANAPC1, SRP14, FKBP1A, ANAPC10, EIF2B1, EIF4A3, PSMB4, PSMA6, PSMD11, PSMA5, EIF3E, PSMC2, UBB, PSMD6, PSMD7, SRP9 | 17 | 3.99 | 9.27E-03 |
| 4 | GO biological process | GO:0031145 | anaphase-promoting complex-dependent proteasomal ubiquitin-dependent protein catabolic process | ANAPC1, PSMB4, PSMA6, PSMD11, PSMA5, PSMC2, ANAPC10, UBB, PSMD6, PSMD7 | 10 | 6.76 | 2.44E-02 |
| 4 | GO biological process | GO:0051436 | negative regulation of ubiquitin-protein ligase activity during mitotic cell cycle | ANAPC1, PSMB4, PSMA6, PSMD11, PSMA5, PSMC2, ANAPC10, UBB, PSMD6, PSMD7 | 10 | 6.76 | 2.44E-02 |
| 4 | GO biological process | GO:0051444 | negative regulation of ubiquitin-protein ligase activity | ANAPC1, PSMB4, PSMA6, PSMD11, PSMA5, PSMC2, ANAPC10, UBB, PSMD6, PSMD7 | 10 | 6.56 | 3.13E-02 |
| 4 | GO biological process | GO:0051352 | negative regulation of ligase activity | ANAPC1, PSMB4, PSMA6, PSMD11, PSMA5, PSMC2, ANAPC10, UBB, PSMD6, PSMD7 | 10 | 6.56 | 3.13E-02 |
| 4 | GO biological process | GO:0043161 | proteasomal ubiquitin-dependent protein catabolic process | ANAPC1, PSMB4, SEC61B, PSMA6, PSMD11, PSMA5, PSMC2, FOXRED2, ANAPC10, UBB, PSMD6, PSMD7 | 12 | 5.17 | 3.19E-02 |
| 4 | GO biological process | GO:0010498 | proteasomal protein catabolic process | ANAPC1, PSMB4, SEC61B, PSMA6, PSMD11, PSMA5, PSMC2, FOXRED2, ANAPC10, UBB, PSMD6, PSMD7 | 12 | 5.17 | 3.19E-02 |
| 4 | GO biological process | GO:0031398 | positive regulation of protein ubiquitination | ANAPC1, PSMB4, PSMA6, PSMD11, PSMA5, PSMC2, FKBP1A, ANAPC10, UBB, PSMD6, PSMD7 | 11 | 5.75 | 3.20E-02 |
| 4 | GO biological process | GO:0034660 | ncRNA metabolic process | PUS3, EMG1, GAR1, EXOSC2, ERI1, RRP8, RRP9, WARS, EIF4A3, TRMT6, RPS15, RPL5, FARSA, NOP56, IMP4, MTO1, GEMIN4, DDX51 | 18 | 3.44 | 3.27E-02 |
| 4 | GO biological process | GO:0051437 | positive regulation of ubiquitin-protein ligase activity during mitotic cell cycle | ANAPC1, PSMB4, PSMA6, PSMD11, PSMA5, PSMC2, ANAPC10, UBB, PSMD6, PSMD7 | 10 | 6.46 | 3.53E-02 |
| 4 | GO biological process | GO:0034470 | ncRNA processing | PUS3, EMG1, GAR1, EXOSC2, ERI1, RRP8, RRP9, EIF4A3, TRMT6, RPS15, RPL5, NOP56, IMP4, MTO1, GEMIN4, DDX51 | 16 | 3.76 | 3.87E-02 |
| 4 | GO biological process | GO:0010605 | negative regulation of macromolecule metabolic process | HSP90AB1, SRP14, COPS2, ERI1, FKBP1A, ANAPC10, DAXX, VPS72, CTNNB1, RPS3, PSMB4, EIF3E, NPM1, AATF, LRRFIP1, DDX20, PSMD6, PSMD7, ENO1, KHDRBS1, ANAPC1, GMNN, PHB, ILF3, MBD1, EIF2B1, PURB, EIF4A3, PSMA6, PSMA5, PSMD11, PSMC2, MLX, ADRA1B, UBB, SRP9 | 36 | 2.15 | 4.14E-02 |
| 4 | GO biological process | GO:0051443 | positive regulation of ubiquitin-protein ligase activity | ANAPC1, PSMB4, PSMA6, PSMD11, PSMA5, PSMC2, ANAPC10, UBB, PSMD6, PSMD7 | 10 | 6.27 | 4.45E-02 |
| 4 | GO biological process | GO:0051439 | regulation of ubiquitin-protein ligase activity during mitotic cell cycle | ANAPC1, PSMB4, PSMA6, PSMD11, PSMA5, PSMC2, ANAPC10, UBB, PSMD6, PSMD7 | 10 | 6.19 | 4.98E-02 |
| 5 | GO biological process | GO:0034660 | ncRNA metabolic process | UTP23, NARS, AIMP1, UTP18, EXOSC4, UTP6, EPRS, EXOSC3, WARS2, NOP10, KARS, EXOSC10, TARS, IMP3, RPL7, RPS17, RPS14, TFB2M, POP4, AARS2, POP7, DUS3L | 22 | 5.02 | 4.02E-06 |
| 5 | GO biological process | GO:0022613 | ribonucleoprotein complex biogenesis | UTP23, NCBP2, UTP18, EXOSC4, UTP6, SNRPD1, SNRPD2, EXOSC3, NOP10, MRTO4, EXOSC10, IMP3, RPL7, RPS17, RPS14, TFB2M, RSL24D1, POP4, TXNL4A | 19 | 5.53 | 1.36E-05 |
| 5 | GO biological process | GO:0006396 | RNA processing | POLR2H, NCBP2, POLR2K, UTP18, U2AF2, PPIL1, UTP6, SNRPD1, SNRPD2, POLR2B, EXOSC10, IMP3, RPL7, U2AF1, TFB2M, DHX35, LSM2, DUS3L, BCAS2, UTP23, CSTF3, CSTF2, EXOSC4, EXOSC3, DDX5, NOP10, RPS17, RPS14, POP4, RBM39, PDCD7, POP7, TXNL4A | 33 | 3.16 | 2.08E-05 |
| 5 | GO biological process | GO:0042254 | ribosome biogenesis | UTP23, UTP18, EXOSC4, UTP6, EXOSC3, NOP10, MRTO4, EXOSC10, IMP3, RPL7, RPS17, RPS14, TFB2M, POP4, RSL24D1 | 15 | 6.45 | 1.12E-04 |
| 5 | GO biological process | GO:0006412 | translation | C12ORF65, SNORA7B, NARS, AIMP1, MRPS11, RPL36, EPRS, WARS2, MRPS2, KARS, TARS, RPL30, MRPS9, RPL7, RPL13A, RPS17, RPS14, EIF4A1, EIF2S2, RPS10, RSL24D1, EIF3J, AARS2, TNIP1 | 24 | 3.80 | 1.24E-04 |
| 5 | GO biological process | GO:0006364 | rRNA processing | UTP23, EXOSC10, IMP3, RPL7, UTP18, RPS17, EXOSC4, RPS14, UTP6, TFB2M, EXOSC3, POP4, NOP10 | 13 | 7.41 | 2.34E-04 |
| 5 | GO biological process | GO:0016072 | rRNA metabolic process | UTP23, EXOSC10, IMP3, RPL7, UTP18, RPS17, EXOSC4, RPS14, UTP6, TFB2M, EXOSC3, POP4, NOP10 | 13 | 7.10 | 3.77E-04 |
| 5 | GO biological process | GO:0016071 | mRNA metabolic process | NCBP2, BCAS2, POLR2H, CSTF3, CSTF2, UPF1, POLR2K, SMG5, PPIL1, U2AF2, SNRPD1, SNRPD2, DDX5, POLR2B, EXOSC10, PARN, U2AF1, PABPC3, DHX35, LSM2, RBM39, POP4, TXNL4A | 23 | 3.26 | 3.32E-03 |
| 5 | GO biological process | GO:0034470 | ncRNA processing | UTP23, UTP18, EXOSC4, UTP6, EXOSC3, NOP10, EXOSC10, IMP3, RPL7, RPS17, RPS14, TFB2M, POP4, POP7, DUS3L | 15 | 4.21 | 1.93E-02 |
| 5 | GO biological process | GO:0008380 | RNA splicing | NCBP2, POLR2H, BCAS2, CSTF3, CSTF2, POLR2K, PPIL1, U2AF2, SNRPD1, SNRPD2, DDX5, POLR2B, U2AF1, DHX35, LSM2, RBM39, PDCD7, TXNL4A | 18 | 3.32 | 4.35E-02 |
| 5 | GO biological process | GO:0043933 | macromolecular complex subunit organization | NCBP2, POLR2H, TRAF2, E2F3, POLR2K, SNRPD1, HR, SNRPD2, NDUFAF1, POLR2B, DSTN, TAPBP, MTMR2, TFAM, GTF2A2, H2AFZ, HSPA4, HIST3H3, TUBA1A, SCO2, SCO1, C12ORF65, HIST1H1E, CRYAA, VHL, EPRS, TUBA8, TBCA, RPS14, HIST1H3C, HIST1H3G, TXNL4A | 31 | 2.29 | 4.77E-02 |
| 10 | GO biological process | GO:0022904 | respiratory electron transport chain | NDUFA4, ND3, NDUFC2, SDHA, NDUFS7, ND4L, NDUFS5, NDUFS4, COX3, COX2, UQCRH, COX1, ND6, ATP8, ATP6 | 13 | 10.25 | 5.49E-06 |
| 10 | GO biological process | GO:0042775 | mitochondrial ATP synthesis coupled electron transport | NDUFA4, ND3, NDUFC2, NDUFS7, ND4L, NDUFS5, NDUFS4, COX3, COX2, UQCRH, COX1, ND6, ATP8, ATP6 | 12 | 10.82 | 1.49E-05 |
| 10 | GO biological process | GO:0042773 | ATP synthesis coupled electron transport | NDUFA4, ND3, NDUFC2, NDUFS7, ND4L, NDUFS5, NDUFS4, COX3, COX2, UQCRH, COX1, ND6, ATP8, ATP6 | 12 | 10.82 | 1.49E-05 |
| 10 | GO biological process | GO:0045333 | cellular respiration | NDUFA4, SUCLG1, ND3, NDUFC2, SDHA, NDUFS7, ND4L, NDUFS5, NDUFS4, COX3, COX2, UQCRH, COX1, ATP8, ND6, ATP6 | 14 | 7.29 | 8.35E-05 |
| 10 | GO biological process | GO:0022900 | electron transport chain | NDUFA4, ND3, NDUFC2, NDUFA13, SDHA, NDUFS7, ND4L, NDUFS5, NDUFS4, COX3, COX2, UQCRH, COX1, ATP8, ND6, ATP6 | 14 | 6.20 | 5.76E-04 |
| 10 | GO biological process | GO:0006119 | oxidative phosphorylation | NDUFA4, MSH2, ND3, NDUFC2, NDUFS7, ND4L, NDUFS5, NDUFS4, COX3, COX2, UQCRH, COX1, ATP8, ND6, ATP6 | 13 | 6.70 | 7.48E-04 |
| 10 | GO biological process | GO:0015980 | energy derivation by oxidation of organic compounds | NDUFA4, SUCLG1, ND3, NDUFC2, SDHA, NDUFS7, ND4L, NDUFS5, NDUFS4, COX3, COX2, UQCRH, COX1, ATP8, ND6, ATP6 | 14 | 4.91 | 8.24E-03 |
| 10 | GO biological process | GO:0000087 | M phase of mitotic cell cycle | SSSCA1, BOD1, CCNK, FZR1, KIF11, USP9X, SIRT7, PBK, KIF2C, CCNB2, SAC3D1, SPAG5, KATNA1, KIF20B, MAD2L2, SMC1A, CDCA3 | 17 | 3.83 | 1.45E-02 |
| 10 | GO biological process | GO:0000278 | mitotic cell cycle | SSSCA1, BOD1, CCNK, FZR1, KIF11, USP9X, SIRT7, PBK, KIF2C, CCNB2, SAC3D1, CDKN2C, SPAG5, KATNA1, KIF20B, MAD2L2, SMC1A, KPNA2, DNAJC2, GADD45A, CDCA3, MAP3K11 | 22 | 3.00 | 2.17E-02 |
| 10 | GO biological process | GO:0006091 | generation of precursor metabolites and energy | NDUFA4, ACOX1, MSH2, SUCLG1, ND3, NDUFC2, NDUFA13, DLAT, SDHA, NDUFS7, ND4L, NDUFS5, NDUFS4, COX3, COX2, UQCRH, COX1, ENO3, ATP8, ND6, ATP6, ACAA1 | 20 | 3.23 | 2.21E-02 |
| 10 | GO biological process | GO:0006120 | mitochondrial electron transport, NADH to ubiquinone | NDUFA4, NDUFS7, ND4L, NDUFS5, NDUFS4, ND3, NDUFC2, ND6 | 8 | 9.61 | 2.42E-02 |
| 10 | GO biological process | GO:0007067 | mitosis | SSSCA1, BOD1, CCNK, FZR1, KIF11, USP9X, PBK, KIF2C, CCNB2, SAC3D1, SPAG5, KATNA1, KIF20B, MAD2L2, SMC1A, CDCA3 | 16 | 3.67 | 4.68E-02 |
| 10 | GO biological process | GO:0000280 | nuclear division | SSSCA1, BOD1, CCNK, FZR1, KIF11, USP9X, PBK, KIF2C, CCNB2, SAC3D1, SPAG5, KATNA1, KIF20B, MAD2L2, SMC1A, CDCA3 | 16 | 3.67 | 4.68E-02 |
| 11 | GO biological process | GO:0007049 | cell cycle | ADCY3, KIF22, CEP72, TSG101, TUBB2A, PTTG2, DAXX, NDE1, CUL5, DDX11, ILK, H2AFX, USP16, SUPT5H, PSMD8, ZW10, PSMD9, ZC3HC1, CCNH, KIF15, UBR2, ESPL1, RB1, CDK7, PIM2, MAPK1, PFDN1, BUB1B, PELO, G0S2, STMN1, SIAH2, UBA52, HAUS6, HAUS2, FOXM1, POLA1, CHEK1, CDC34, TUBB, PSMB1, FBXO5, LFNG, HELLS, ZNF830, PCNT, DLGAP5, PSRC1, SMAD3, CDC23, RAD54L, CENPJ, AVPI1, SUV39H2, PLK3, NOLC1, PSMC3, MAPK13, PLK1, PSMC1, CHTF18, SMC1A | 62 | 2.07 | 1.68E-04 |
| 11 | GO biological process | GO:0022402 | cell cycle process | ADCY3, KIF22, CEP72, HAUS6, TSG101, TUBB2A, HAUS2, POLA1, CHEK1, PTTG2, CDC34, DAXX, TUBB, CUL5, NDE1, DDX11, PSMB1, ILK, FBXO5, H2AFX, USP16, LFNG, PSMD8, HELLS, ZW10, PSMD9, ZC3HC1, ZNF830, PCNT, DLGAP5, KIF15, SMAD3, CDC23, UBR2, ESPL1, RB1, PIM2, RAD54L, CENPJ, SUV39H2, NOLC1, PSMC3, PLK1, PSMC1, BUB1B, STMN1, SMC1A, UBA52 | 48 | 2.20 | 1.21E-03 |
| 11 | GO biological process | GO:0006412 | translation | NACA, COPS5, EIF5, IARS2, VARS, RPS2, MTIF3, RPS26, GFM1, RPL9, ICT1, EIF1AX, EIF3F, EIF1AY, EEF2K, MRPL37, MRPL33, MRPL52, MRPL51, RARS2, LARS2, LRRC47, RPS6, RPS4X, MRPL23, MRPL22, MRPL21, RPS16, MRPS18A, RARS, RPL21, HARS, PELO, UBA52 | 34 | 2.66 | 1.28E-03 |
| 11 | GO biological process | GO:0000279 | M phase | ADCY3, KIF22, HAUS6, TUBB2A, HAUS2, CHEK1, PTTG2, NDE1, TUBB, DDX11, FBXO5, H2AFX, USP16, LFNG, HELLS, ZW10, ZC3HC1, ZNF830, PCNT, DLGAP5, KIF15, CDC23, ESPL1, UBR2, RB1, PIM2, RAD54L, SUV39H2, NOLC1, PLK1, BUB1B, STMN1, SMC1A | 33 | 2.60 | 3.27E-03 |
| 11 | GO biological process | GO:0000278 | mitotic cell cycle | KIF22, HAUS6, TUBB2A, HAUS2, POLA1, CHEK1, PTTG2, CDC34, CUL5, NDE1, TUBB, PSMB1, DDX11, FBXO5, USP16, HELLS, PSMD8, ZW10, PSMD9, ZC3HC1, ZNF830, DLGAP5, KIF15, CDC23, ESPL1, RB1, NOLC1, PLK1, PSMC3, PSMC1, BUB1B, STMN1, SMC1A, UBA52 | 34 | 2.38 | 1.47E-02 |
| 11 | GO biological process | GO:0022403 | cell cycle phase | ADCY3, KIF22, HAUS6, TUBB2A, HAUS2, POLA1, CHEK1, PTTG2, CDC34, CUL5, NDE1, TUBB, DDX11, FBXO5, H2AFX, USP16, LFNG, HELLS, ZW10, ZC3HC1, ZNF830, PCNT, DLGAP5, KIF15, CDC23, ESPL1, UBR2, RB1, PIM2, RAD54L, SUV39H2, NOLC1, PLK1, BUB1B, STMN1, SMC1A | 36 | 2.25 | 2.50E-02 |
| 11 | GO biological process | GO:0051603 | proteolysis involved in cellular protein catabolic process | OTUD5, TSG101, IDE, UBE2V1, RNF216, CDC34, UBAC1, STUB1, UBE2R2, CUL5, FBXW5, PSMB1, FBXW4, CACYBP, USP11, FBXO5, TRAF7, USP16, FBXO9, PSMD8, USP13, PSMD9, NPLOC4, ZC3HC1, SPSB1, UBE4B, RING1, CDC23, UBR2, TMEM189, UBE2Q1, URM1, HSP90B1, RNF5, PSMC3, OTUB1, UBA1, BACE2, MED8, PSMC1, USP47, TCEB2, BUB1B, UCHL3, SIAH2, FAF1, UBA52 | 46 | 1.99 | 3.21E-02 |
| 11 | GO biological process | GO:0046907 | intracellular transport | GNPTG, BID, ATP5D, ARFGAP3, TSPO, TIMM17B, TIMM10, PEX7, BAK1, NDE1, ACD, CSE1L, TOMM5, STX18, COPB1, PEX16, NUP50, RANBP3, MKKS, TMED10, CCS, SLC25A1, WIPF1, EHD1, SEC24C, ZW10, MCM3AP, FAM160A2, HSP90AA1, RPGR, NUDT4, SLC25A6, PICK1, YWHAB, RPH3AL, SMG1, HNRNPA1, PREB, MAPK1, UXT, AAAS, ATP2A2, YWHAQ, PTTG1IP, USO1, TOM1, GOSR2, SH3D19, TRAPPC2 | 49 | 1.93 | 3.30E-02 |
| 11 | GO biological process | GO:0030163 | protein catabolic process | OTUD5, TSG101, IDE, UBE2V1, RNF216, CDC34, UBAC1, STUB1, UBE2R2, CUL5, FBXW5, PSMB1, FBXW4, CACYBP, USP11, FBXO5, TRAF7, USP16, FBXO9, PSMD8, USP13, PSMD9, NPLOC4, ZC3HC1, SPSB1, UBE4B, RING1, CDC23, UBR2, TMEM189, UBE2Q1, URM1, HSP90B1, RNF5, PSMC3, OTUB1, UBA1, BACE2, MED8, PSMC1, USP47, TCEB2, BUB1B, UCHL3, SIAH2, FAF1, CLN5, UBA52 | 47 | 1.96 | 3.65E-02 |
| 11 | GO biological process | GO:0044257 | cellular protein catabolic process | OTUD5, TSG101, IDE, UBE2V1, RNF216, CDC34, UBAC1, STUB1, UBE2R2, CUL5, FBXW5, PSMB1, FBXW4, CACYBP, USP11, FBXO5, TRAF7, USP16, FBXO9, PSMD8, USP13, PSMD9, NPLOC4, ZC3HC1, SPSB1, UBE4B, RING1, CDC23, UBR2, TMEM189, UBE2Q1, URM1, HSP90B1, RNF5, PSMC3, OTUB1, UBA1, BACE2, MED8, PSMC1, USP47, TCEB2, BUB1B, UCHL3, SIAH2, FAF1, UBA52 | 46 | 1.98 | 3.67E-02 |
| 11 | GO biological process | GO:0044265 | cellular macromolecule catabolic process | OTUD5, TSG101, IDE, UBE2V1, RNF216, HSPA1A, CDC34, UBAC1, STUB1, UBE2R2, CUL5, FBXW5, PSMB1, FBXW4, CACYBP, FBXO5, USP11, TRAF7, USP16, FBXO9, PSMD8, PSMD9, USP13, NPLOC4, ZC3HC1, SPSB1, CCNH, UBE4B, RING1, CDC23, SMG1, UBR2, CDK7, TMEM189, UBE2Q1, URM1, HSP90B1, OTUB1, RNF5, UBA1, PSMC3, BACE2, MED8, PSMC1, POP1, USP47, TCEB2, TDG, BUB1B, UCHL3, SIAH2, FAF1, UBA52 | 52 | 1.86 | 4.97E-02 |
| 15 | GO biological process | GO:0016192 | vesicle-mediated transport | RAB7A, SEC31B, SEC31A, CYTH1, AP2S1, TXLNA, STX12, TMED2, BLOC1S3, ZFYVE16, BLOC1S1, NECAP2, DNAJC6, AP2M1, STX4, VTI1A, COPG2, LAT2, RABEP2, SCYL1, ARRB2, HGS, GGA1, ARAP3, SYTL1, SLC17A9 | 26 | 3.05 | 1.42E-03 |
| 15 | GO biological process | GO:0006412 | translation | MRPS36, RPLP2, RPL23A, IGF2BP3, GFM2, TRNAU1AP, MRPS18C, EIF4E, EIF4EBP2, RPL23, MRPL14, RPS14, TSFM, MRPL16, MRPL45, EIF2AK2, RPS27A | 17 | 3.47 | 3.62E-02 |
| 1 | GO cellular component | GO:0043232 | intracellular non-membrane-bounded organelle | S100A4, KIF23, HMGN1, SPIN1, ATP5B, XRCC6, RPS27L, CBX1, KRTAP5-2, PNP, PRIM1, HIST2H2AB, DCAF13, HIST1H2BM, DDX28, SEH1L, GSN, HIST1H2BI, URB2, RHOA, RPL11, DDX21, CDCA5, MRPL2, GTPBP4, EXOSC7, BYSL, APTX, EXOSC1, KRTAP10-9, RAD51, NOC2L, RFC4, SGCE, RBM39, KRTAP1-4, MEAF6, MTDH, ACTR3B, CALD1, THAP6, MRPS10, TIPIN, RPS15A, C14ORF166, ARPC5, TRIB2, TPM4, KRTAP10-10, JRK, RPA1, HNRNPM, NOM1, RPS29, MRPL17, RPL8, SBDSP1, CC2D1A, RPL10A, RPS21, RPS10P7, HIST1H2BB, EPB42, CEP192, MYO1E, HIST1H2BH, HEATR1, MYL12A, COTL1, FOXP4, FBL, RPS7, SMC4, FSD1, TUBBP5, PSMD10, HIST1H3A, SEC13, KRTAP19-2, DAP3 | 80 | 2.00 | 2.58E-08 |
| 1 | GO cellular component | GO:0043228 | non-membrane-bounded organelle | S100A4, KIF23, HMGN1, SPIN1, ATP5B, XRCC6, RPS27L, CBX1, KRTAP5-2, PNP, PRIM1, HIST2H2AB, DCAF13, HIST1H2BM, DDX28, SEH1L, GSN, HIST1H2BI, URB2, RHOA, RPL11, DDX21, CDCA5, MRPL2, GTPBP4, EXOSC7, BYSL, APTX, EXOSC1, KRTAP10-9, RAD51, NOC2L, RFC4, SGCE, RBM39, KRTAP1-4, MEAF6, MTDH, ACTR3B, CALD1, THAP6, MRPS10, TIPIN, RPS15A, C14ORF166, ARPC5, TRIB2, TPM4, KRTAP10-10, JRK, RPA1, HNRNPM, NOM1, RPS29, MRPL17, RPL8, SBDSP1, CC2D1A, RPL10A, RPS21, RPS10P7, HIST1H2BB, EPB42, CEP192, MYO1E, HIST1H2BH, HEATR1, MYL12A, COTL1, FOXP4, FBL, RPS7, SMC4, FSD1, TUBBP5, PSMD10, HIST1H3A, SEC13, KRTAP19-2, DAP3 | 80 | 2.00 | 2.58E-08 |
| 1 | GO cellular component | GO:0030529 | ribonucleoprotein complex | HNRNPA1L2, STRAP, CWC15, MRPS10, EDC4, RPS15A, RPS27L, JRK, HNRNPM, DCAF13, RPS29, MRPL17, RPL8, RPL11, RPL10A, RPS21, MRPL2, RPS10P7, SNRPA1, EFTUD2, HEATR1, RBMX, FBL, RPS7, DCP1A, SNRPE, DAP3 | 27 | 3.40 | 2.37E-05 |
| 1 | GO cellular component | GO:0031967 | organelle envelope | NXT1, NDUFAF4, ALDH18A1, HTATIP2, MTDH, NDUFB6, ATP5B, TIMM17A, CHCHD4, GOT2, UQCR10, DDX19A, SEH1L, MRPL17, TIMM9, SHISA5, COX16, OCIAD2, NDUFV3, UQCRH, IPO7, ERN1, TOMM20, SEC13, TOMM22, NLN, KPNA4 | 27 | 2.83 | 8.15E-04 |
| 1 | GO cellular component | GO:0031975 | envelope | NXT1, NDUFAF4, ALDH18A1, HTATIP2, MTDH, NDUFB6, ATP5B, TIMM17A, CHCHD4, GOT2, UQCR10, DDX19A, SEH1L, MRPL17, TIMM9, SHISA5, COX16, OCIAD2, NDUFV3, UQCRH, IPO7, ERN1, TOMM20, SEC13, TOMM22, NLN, KPNA4 | 27 | 2.82 | 8.65E-04 |
| 1 | GO cellular component | GO:0044427 | chromosomal part | HMGN1, HIST1H2BB, HIST1H2BH, XRCC6, TIPIN, APTX, CBX1, SMC4, RPA1, PRIM1, JRK, HIST2H2AB, HIST1H2BM, RFC4, SEH1L, HIST1H2BI, HIST1H3A, SEC13, CDCA5 | 19 | 3.19 | 7.66E-03 |
| 1 | GO cellular component | GO:0031974 | membrane-enclosed lumen | KIF23, S100A4, ATP5B, XRCC6, PRKAG2, INTS1, CBX1, RLIM, MED20, GOT2, PRIM1, DCAF13, DDX28, URB2, TIMM9, DDX21, RPL11, GTPBP4, EXOSC7, BYSL, ERP29, RBL1, APTX, EXOSC1, NOC2L, RAD51, RFC4, FDX1L, RBM39, SNRPE, POLR2G, MEAF6, MTDH, NFYA, CHCHD4, EDEM3, RPA1, HNRNPM, NOM1, SBDSP1, CD4, ACTL6A, CC2D1A, HEATR1, FOXP4, FBL, RPS7, RSRC1, NLN, MBTPS1, DAP3 | 51 | 1.78 | 9.22E-03 |
| 1 | GO cellular component | GO:0032993 | protein-DNA complex | RPA1, PRIM1, HIST1H2BB, HIST2H2AB, HIST1H2BM, HIST1H2BI, HIST1H2BH, XRCC6, HIST1H3A | 9 | 6.79 | 1.41E-02 |
| 1 | GO cellular component | GO:0005694 | chromosome | HMGN1, HIST1H2BB, HIST1H2BH, XRCC6, TIPIN, APTX, CBX1, SMC4, RAD51, RPA1, PRIM1, JRK, HIST2H2AB, HIST1H2BM, RFC4, SEH1L, HIST1H2BI, HIST1H3A, SEC13, CDCA5 | 20 | 2.82 | 2.34E-02 |
| 1 | GO cellular component | GO:0070013 | intracellular organelle lumen | KIF23, S100A4, POLR2G, MEAF6, MTDH, ATP5B, XRCC6, PRKAG2, INTS1, CBX1, NFYA, EDEM3, RLIM, MED20, GOT2, PRIM1, RPA1, HNRNPM, DCAF13, NOM1, DDX28, URB2, SBDSP1, CD4, DDX21, ACTL6A, RPL11, CC2D1A, GTPBP4, EXOSC7, BYSL, RBL1, ERP29, APTX, HEATR1, EXOSC1, FOXP4, FBL, NOC2L, RPS7, RAD51, RFC4, RSRC1, FDX1L, RBM39, SNRPE, MBTPS1, DAP3 | 48 | 1.75 | 2.75E-02 |
| 1 | GO cellular component | GO:0005840 | ribosome | RPS10P7, MRPL2, MRPS10, RPS15A, RPS27L, RPS7, RPS29, MRPL17, RPL8, RPL11, RPL10A, RPS21, DAP3 | 13 | 3.92 | 3.27E-02 |
| 1 | GO cellular component | GO:0043233 | organelle lumen | KIF23, S100A4, POLR2G, MEAF6, MTDH, ATP5B, XRCC6, PRKAG2, INTS1, CBX1, NFYA, EDEM3, RLIM, MED20, GOT2, PRIM1, RPA1, HNRNPM, DCAF13, NOM1, DDX28, URB2, SBDSP1, CD4, DDX21, ACTL6A, RPL11, CC2D1A, GTPBP4, EXOSC7, BYSL, RBL1, ERP29, APTX, HEATR1, EXOSC1, FOXP4, FBL, NOC2L, RPS7, RAD51, RFC4, RSRC1, FDX1L, RBM39, SNRPE, MBTPS1, DAP3 | 48 | 1.71 | 4.72E-02 |
| 2 | GO cellular component | GO:0030529 | ribonucleoprotein complex | XPO1, MRPS33, LSM6, PABPC4, SF3B3, MRPL13, DKC1, BTBD1, RPLP1, RPL26L1, PPIL3, LSM1, HSPA8, MRPL35, RPL35A, RPL27, MRPS6, RPL24, HNRNPR, RPS8, SF3A3, PPIH, ILF2, MRPS18B, UBC, WDR3, MRPL47, RPP40 | 28 | 3.10 | 9.36E-05 |
| 2 | GO cellular component | GO:0043232 | intracellular non-membrane-bounded organelle | NKAP, XPO1, TAF1A, MRPS33, CDC14C, KRTAP20-2, HIST1H2BO, ACTG1, FNTA, DKC1, HIST1H2BK, FANCI, HIST1H2BL, RPLP1, RPL26L1, TSEN2, MRPL35, KRTAP2-4, CLNS1A, RPL35A, POLR1D, NIP7, TBCE, UBR4, MRPS6, RAD50, KRTAP10-8, CD3EAP, ARPC1A, NVL, KRTAP5-4, KRT18, MRPS18B, CGN, UBC, TUBA4A, KRTAP5-10, KRTAP1-3, MRPL47, ARL8B, KRTAP5-11, SGCB, PARVA, SMARCAD1, HMGB2, BLM, HIST1H2AD, PXN, DAZAP1, VDR, MRPL13, VRK1, PTK2B, PPP2CB, NUDT21, ZWILCH, ERCC1, PLEC, KRTAP9-6, VPS18, SUB1, PAK1IP1, PTPN14, RPL27, RPL24, RPS8, HIST2H3D, LSP1, ILF2, HDAC1, PTP4A1, KRTAP4-11, GAS2L1, WDR3, H3F3A, CACNA1C, RPP40, CALM2 | 77 | 1.69 | 2.68E-04 |
| 2 | GO cellular component | GO:0043228 | non-membrane-bounded organelle | NKAP, XPO1, TAF1A, MRPS33, CDC14C, KRTAP20-2, HIST1H2BO, ACTG1, FNTA, DKC1, HIST1H2BK, FANCI, HIST1H2BL, RPLP1, RPL26L1, TSEN2, MRPL35, KRTAP2-4, CLNS1A, RPL35A, POLR1D, NIP7, TBCE, UBR4, MRPS6, RAD50, KRTAP10-8, CD3EAP, ARPC1A, NVL, KRTAP5-4, KRT18, MRPS18B, CGN, UBC, TUBA4A, KRTAP5-10, KRTAP1-3, MRPL47, ARL8B, KRTAP5-11, SGCB, PARVA, SMARCAD1, HMGB2, BLM, HIST1H2AD, PXN, DAZAP1, VDR, MRPL13, VRK1, PTK2B, PPP2CB, NUDT21, ZWILCH, ERCC1, PLEC, KRTAP9-6, VPS18, SUB1, PAK1IP1, PTPN14, RPL27, RPL24, RPS8, HIST2H3D, LSP1, ILF2, HDAC1, PTP4A1, KRTAP4-11, GAS2L1, WDR3, H3F3A, CACNA1C, RPP40, CALM2 | 77 | 1.69 | 2.68E-04 |
| 2 | GO cellular component | GO:0045095 | keratin filament | KRTAP2-4, KRTAP5-4, KRTAP9-6, KRT18, KRTAP4-11, KRTAP5-10, KRTAP1-3, KRTAP5-11, KRTAP10-8 | 9 | 5.84 | 4.24E-02 |
| 3 | GO cellular component | GO:0030529 | ribonucleoprotein complex | RPL18, MRPS34, MRPL41, SYNCRIP, RPS6KB1, WBP4, YBX1, MRPL36, LSM5, LSM3, MRPL32, PABPN1, PRPF31, MRPL3, HNRNPU, SMN1, WDR83, RSL1D1, PSMA1, PA2G4, PIH1D1, RPS19, SNRPB, MRPL49, CPSF6, RPS13, SNRPA, RPS11, MRPS17, HSPA1B, BOP1, SF3B5, STAU1, SF3B2, MRPL11, MRPL12, RPL3, HNRNPD, MRPL18, PPWD1, RPS24, MRPS26, MRPS24, DDX1, SNW1, CASC3, LARP4B, RPS5, RPL28, MRPL24, RPL18A, MRPL28, MRPL27, RPL19P12, HNRNPH1 | 55 | 3.35 | 2.71E-12 |
| 3 | GO cellular component | GO:0070013 | intracellular organelle lumen | EIF6, MRPL41, SYNCRIP, MED22, WTAP, CNOT7, EBNA1BP2, MAK16, TARDBP, SMARCD1, MRPL36, HADH, MRPL32, TBPL1, PABPN1, MTA1, ARID1B, PPP1CB, RSL1D1, PA2G4, RPS19, CLPP, SNRPB, FLII, RPS13, NEU1, PAF1, AKAP8, MYBBP1A, MRPS17, SYVN1, BOP1, MUT, HIST1H4A, BRIX1, RPL3, HIST1H4D, DNAJA3, FH, MRPS26, TSEN54, MRPS24, GARS, SNW1, CDC20, CASC3, PMF1, VDAC2, INTS10, RPF1, CTR9, DDX56, PPIB, NOP16, GTF2F1, GTF2F2, DNAJB1, PARP1, HNRNPH1, PARP2, PC, BCKDK, WBP4, ZNF207, SBDS, BRD4, DEDD2, PRPF31, EXOSC8, MRPL3, DDB1, CYCS, TPX2, NUSAP1, MBD4, UBE2I, MCM2, CDK4, UBN1, MCM5, SMN1, XPA, MED4, ANKRD28, IPO5, MED9, CPSF6, CPSF3, WDR43, TUFM, POLR2F, ZBTB11, ECHS1, TSPYL1, MRPL11, SRRT, SET, MRPL12, TOR1A, GTF3C5, MRPL18, THAP1, PDHX, HSPA9, DLST, NDUFA9, TRIM28, POLR3A, ZNF22, SOD2, PKNOX1, MRPL28, MRPL27, POLDIP2, TCEB3 | 114 | 2.01 | 3.34E-11 |
| 3 | GO cellular component | GO:0043233 | organelle lumen | EIF6, MRPL41, SYNCRIP, MED22, WTAP, CNOT7, EBNA1BP2, MAK16, TARDBP, SMARCD1, MRPL36, HADH, MRPL32, TBPL1, PABPN1, MTA1, ARID1B, PPP1CB, RSL1D1, PA2G4, RPS19, CLPP, SNRPB, FLII, RPS13, NEU1, PAF1, AKAP8, MYBBP1A, MRPS17, SYVN1, BOP1, MUT, HIST1H4A, BRIX1, RPL3, HIST1H4D, DNAJA3, FH, MRPS26, TSEN54, MRPS24, GARS, SNW1, CDC20, CASC3, PMF1, VDAC2, INTS10, RPF1, CTR9, DDX56, PPIB, NOP16, GTF2F1, GTF2F2, DNAJB1, PARP1, HNRNPH1, PARP2, PC, BCKDK, WBP4, ZNF207, SBDS, SERPINA1, BRD4, DEDD2, PRPF31, EXOSC8, MRPL3, DDB1, CYCS, TPX2, NUSAP1, MBD4, UBE2I, MCM2, CDK4, UBN1, MCM5, SMN1, XPA, MED4, ANKRD28, IPO5, MED9, CPSF6, CPSF3, WDR43, TUFM, POLR2F, ZBTB11, ECHS1, TSPYL1, MRPL11, SRRT, SET, MRPL12, TOR1A, GTF3C5, MRPL18, THAP1, PDHX, HSPA9, DLST, NDUFA9, TRIM28, POLR3A, ZNF22, SOD2, PKNOX1, MRPL28, MRPL27, POLDIP2, TCEB3 | 115 | 1.98 | 6.46E-11 |
| 3 | GO cellular component | GO:0031974 | membrane-enclosed lumen | EIF6, MRPL41, SYNCRIP, MED22, CNOT7, WTAP, EBNA1BP2, MAK16, HTRA2, TARDBP, SMARCD1, MRPL36, HADH, MRPL32, TBPL1, PABPN1, MTA1, ARID1B, PPP1CB, RSL1D1, PA2G4, RPS19, CLPP, SNRPB, FLII, RPS13, NEU1, PAF1, AKAP8, MYBBP1A, MRPS17, SYVN1, BOP1, MUT, HIST1H4A, BRIX1, RPL3, HIST1H4D, DNAJA3, FH, MRPS26, TSEN54, MRPS24, GARS, SNW1, CDC20, CASC3, PMF1, VDAC2, INTS10, RPF1, CTR9, DDX56, PPIB, NOP16, GTF2F1, GTF2F2, DNAJB1, PARP1, HNRNPH1, PARP2, PC, BCKDK, WBP4, ZNF207, SBDS, SERPINA1, BRD4, DEDD2, PRPF31, EXOSC8, MRPL3, DDB1, CYCS, TPX2, NUSAP1, MBD4, UBE2I, MCM2, CDK4, UBN1, MCM5, SMN1, XPA, MED4, ANKRD28, IPO5, MED9, CPSF6, CPSF3, WDR43, TUFM, POLR2F, ZBTB11, ECHS1, TSPYL1, MRPL11, SRRT, SET, MRPL12, TOR1A, GTF3C5, MRPL18, THAP1, PDHX, HSPA9, DLST, NDUFA9, TRIM28, POLR3A, ZNF22, SOD2, PKNOX1, MRPL28, MRPL27, POLDIP2, TCEB3 | 116 | 1.96 | 1.01E-10 |
| 3 | GO cellular component | GO:0005739 | mitochondrion | BCKDK, MRPS34, COX11, MRPL41, CKMT1B, UQCRC1, GFER, GLT8D1, NDUFS6, TRIAP1, HTRA2, SLC25A22, MRPL36, ATP5L, SHC1, NDUFS3, HADH, MRPL32, OMA1, DNAJC19, RTN4IP1, PABPN1, MRPL3, NDUFB10, CYCS, NDUFC2, ISCA1, PYCR1, KIF1B, MTRF1, CLPP, MRPL49, MPST, TUFM, MRPS17, NDUFB7, SAMM50, KIAA0101, CHCHD2, ECHS1, ATP5G1, HSPA1B, AGMAT, TACO1, MRPL11, MUT, MRPL12, MTCH2, SH3GLB1, PPP2CA, MTCH1, MRPL18, PDHX, DNAJA3, FH, HSPA9, ATP5L2, DLST, MRPS26, ATP5J2, NDUFA9, MMADHC, MRPS24, MCAT, GARS, MUL1, MRRF, VDAC2, VDAC3, SOD2, MRPL24, METTL12, SDHB, CKMT1A, MRPL28, COG8, MRPL27, PHB2, ENDOG, POLDIP2, SLC25A15, PC | 81 | 2.33 | 2.46E-10 |
| 3 | GO cellular component | GO:0044429 | mitochondrial part | BCKDK, COX11, MRPL41, UQCRC1, CKMT1B, NDUFS6, HTRA2, SLC25A22, MRPL36, ATP5L, NDUFS3, HADH, MRPL32, DNAJC19, MRPL3, NDUFB10, CYCS, NDUFC2, CLPP, TUFM, MRPS17, SAMM50, NDUFB7, ECHS1, ATP5G1, MRPL11, MUT, MRPL12, MTCH2, SH3GLB1, MTCH1, MRPL18, PDHX, DNAJA3, FH, HSPA9, ATP5L2, MRPS26, DLST, ATP5J2, NDUFA9, MRPS24, GARS, MUL1, VDAC2, VDAC3, SOD2, SDHB, CKMT1A, MRPL28, MRPL27, PHB2, POLDIP2, SLC25A15, PC | 54 | 2.84 | 3.36E-09 |
| 3 | GO cellular component | GO:0005759 | mitochondrial matrix | TUFM, MRPS17, BCKDK, MRPL41, ECHS1, MRPL11, MUT, MRPL12, MRPL36, MRPL18, MRPL32, HADH, PDHX, DNAJA3, HSPA9, FH, DLST, MRPS26, MRPL3, NDUFA9, MRPS24, CYCS, GARS, VDAC2, SOD2, MRPL28, MRPL27, CLPP, POLDIP2, PC | 30 | 4.14 | 6.67E-08 |
| 3 | GO cellular component | GO:0031980 | mitochondrial lumen | TUFM, MRPS17, BCKDK, MRPL41, ECHS1, MRPL11, MUT, MRPL12, MRPL36, MRPL18, MRPL32, HADH, PDHX, DNAJA3, HSPA9, FH, DLST, MRPS26, MRPL3, NDUFA9, MRPS24, CYCS, GARS, VDAC2, SOD2, MRPL28, MRPL27, CLPP, POLDIP2, PC | 30 | 4.14 | 6.67E-08 |
| 3 | GO cellular component | GO:0005840 | ribosome | RPL18, MRPS17, MRPS34, MRPL41, RPS6KB1, MRPL11, MRPL12, RPL3, MRPL36, MRPL18, MRPL32, RPS24, MRPS26, MRPL3, MRPS24, RPS5, RPL28, RSL1D1, MRPL24, RPS19, MRPL28, RPL18A, MRPL27, MRPL49, RPS13, RPS11, RPL19P12 | 27 | 3.93 | 1.98E-06 |
| 3 | GO cellular component | GO:0033279 | ribosomal subunit | RPL18, MRPS17, MRPS26, MRPL3, MRPL41, MRPS24, RPS5, RPL28, MRPL11, MRPL12, RPS19, RPL18A, MRPL27, MRPL36, RPL3, RPS13, RPS11, RPL19P12, MRPL32, RPS24 | 20 | 4.90 | 8.75E-06 |
| 3 | GO cellular component | GO:0043228 | non-membrane-bounded organelle | EIF6, RPL18, MRPS34, MRPL41, PRC1, HIRA, CBX3, RPS6KB1, WTAP, VCL, EBNA1BP2, MAK16, RAE1, TARDBP, SMARCD1, TPT1, MRPL36, STAG3, NUP37, MRPL32, PABPN1, MTA1, DCTN6, ARID1B, DCTN3, CTNNA1, RSL1D1, PA2G4, RPS19, MAD2L1BP, FNBP1L, IGBP1, KRT15, PSMA3, MRPL49, FLII, RPS13, ARL8A, AKAP8, RPS11, MAP7D1, MYBBP1A, MAP6D1, MYL6, ALDOA, MRPS17, SYVN1, DNAH14, BOP1, CAPZB, HIST1H4A, BRIX1, HNRNPD, RPL3, HIST1H4D, ARHGDIA, DNAJA3, NUDC, RPS24, MRPS26, TSEN54, CKAP5, MRPS24, SNW1, CDC20, PMF1, VDAC2, RPS5, RPF1, DDX56, RPL18A, CDC42SE2, NOP16, GTF2F2, DNAJB1, PARP1, HNRNPH1, PARP2, KIF22, LIMA1, ZNF207, SBDS, DYNLL1, BRD4, DEDD2, FANCC, ARL2, EXOSC8, MRPL3, DSN1, TPX2, NUSAP1, MBD4, UBE2I, MCM2, SLC9A3R1, MED4, KIF1B, TBCB, ARPC5L, PFDN5, CLIC5, IPO5, PLA2G6, WDR43, TUFM, POLR2F, ZBTB11, STAU1, AURKAPS1, TSPYL1, MRPL11, MRPL12, PPP2CA, TOR1A, WIPF2, MRPL18, HSPA9, TRIM28, DRG1, GABARAP, MIS12, ZNF22, RPL28, MRPL24, MRPL28, MRPL27, POLDIP2, RPL19P12 | 128 | 1.54 | 3.63E-05 |
| 3 | GO cellular component | GO:0043232 | intracellular non-membrane-bounded organelle | EIF6, RPL18, MRPS34, MRPL41, PRC1, HIRA, CBX3, RPS6KB1, WTAP, VCL, EBNA1BP2, MAK16, RAE1, TARDBP, SMARCD1, TPT1, MRPL36, STAG3, NUP37, MRPL32, PABPN1, MTA1, DCTN6, ARID1B, DCTN3, CTNNA1, RSL1D1, PA2G4, RPS19, MAD2L1BP, FNBP1L, IGBP1, KRT15, PSMA3, MRPL49, FLII, RPS13, ARL8A, AKAP8, RPS11, MAP7D1, MYBBP1A, MAP6D1, MYL6, ALDOA, MRPS17, SYVN1, DNAH14, BOP1, CAPZB, HIST1H4A, BRIX1, HNRNPD, RPL3, HIST1H4D, ARHGDIA, DNAJA3, NUDC, RPS24, MRPS26, TSEN54, CKAP5, MRPS24, SNW1, CDC20, PMF1, VDAC2, RPS5, RPF1, DDX56, RPL18A, CDC42SE2, NOP16, GTF2F2, DNAJB1, PARP1, HNRNPH1, PARP2, KIF22, LIMA1, ZNF207, SBDS, DYNLL1, BRD4, DEDD2, FANCC, ARL2, EXOSC8, MRPL3, DSN1, TPX2, NUSAP1, MBD4, UBE2I, MCM2, SLC9A3R1, MED4, KIF1B, TBCB, ARPC5L, PFDN5, CLIC5, IPO5, PLA2G6, WDR43, TUFM, POLR2F, ZBTB11, STAU1, AURKAPS1, TSPYL1, MRPL11, MRPL12, PPP2CA, TOR1A, WIPF2, MRPL18, HSPA9, TRIM28, DRG1, GABARAP, MIS12, ZNF22, RPL28, MRPL24, MRPL28, MRPL27, POLDIP2, RPL19P12 | 128 | 1.54 | 3.63E-05 |
| 3 | GO cellular component | GO:0005761 | mitochondrial ribosome | MRPL11, MRPS26, MRPS17, MRPL12, MRPL3, MRPL41, MRPL28, MRPL27, MRPS24, MRPL36, MRPL18, MRPL32 | 12 | 7.83 | 9.49E-05 |
| 3 | GO cellular component | GO:0000313 | organellar ribosome | MRPL11, MRPS26, MRPS17, MRPL12, MRPL3, MRPL41, MRPL28, MRPL27, MRPS24, MRPL36, MRPL18, MRPL32 | 12 | 7.83 | 9.49E-05 |
| 3 | GO cellular component | GO:0005829 | cytosol | RPL18, LDHA, AP1G1, RPS6KB1, AIP, VCL, CDC42, DYNLL1, VPS4B, PSMD3, SHC1, PDRG1, COX19, IMPDH1, EDARADD, NT5C, FANCC, ARL2, DARS, CYCS, FADD, CCT6A, CLIC1, DCTN3, CDK4, RPTOR, SMN1, PSMA2, PSMA1, RPS19, PFDN6, ITGB1BP1, PFDN5, PSMA3, SNRPB, ADSL, RPS13, RPS11, EEF1D, GGCT, UPP1, PSMA7, FTH1, PSMB5, EIF3D, SET, EIF3B, PSMB6, PPP2CA, RPL3, HNRNPD, EIF3I, DNAJA3, ARHGDIA, RPS24, ODC1, AIMP2, CKAP5, IREB2, GARS, CSNK2B, CDC20, ARFIP1, VAV2, ATG3, RPS5, RPL28, PPA1, PSMD14, PKNOX1, RPL18A, PPIA, PRKAR1B, EIF4H, RPL19P12, IKBKB, GUK1 | 77 | 1.81 | 1.04E-04 |
| 3 | GO cellular component | GO:0031981 | nuclear lumen | EIF6, SYNCRIP, MED22, CNOT7, WTAP, WBP4, ZNF207, EBNA1BP2, SBDS, MAK16, TARDBP, SMARCD1, BRD4, DEDD2, TBPL1, PABPN1, PRPF31, EXOSC8, DDB1, TPX2, NUSAP1, MTA1, MBD4, UBE2I, ARID1B, MCM2, CDK4, PPP1CB, UBN1, MCM5, SMN1, RSL1D1, XPA, MED4, PA2G4, RPS19, ANKRD28, IPO5, MED9, SNRPB, FLII, RPS13, CPSF6, PAF1, AKAP8, CPSF3, MYBBP1A, WDR43, POLR2F, SYVN1, ZBTB11, BOP1, TSPYL1, SRRT, SET, HIST1H4A, BRIX1, RPL3, GTF3C5, THAP1, HIST1H4D, TSEN54, TRIM28, CDC20, SNW1, POLR3A, PMF1, CASC3, INTS10, ZNF22, RPF1, CTR9, DDX56, PKNOX1, GTF2F1, NOP16, GTF2F2, TCEB3, DNAJB1, HNRNPH1, PARP1, PARP2 | 81 | 1.75 | 1.98E-04 |
| 3 | GO cellular component | GO:0031967 | organelle envelope | EIF6, COX11, CKMT1B, UQCRC1, SAMM50, NDUFB7, CBX3, ATP5G1, WTAP, NDUFS6, HTRA2, MTCH2, SH3GLB1, RAE1, TOR1A, SLC25A22, MTCH1, ATP5L, NUP37, NDUFS3, HADH, TNPO1, DNAJC19, ATP5L2, ATP5J2, TMCO6, NDUFB10, NDUFA9, CYCS, NDUFC2, MUL1, CLIC1, NUP155, VDAC2, SIGMAR1, VDAC3, SOD2, SENP2, SDHB, CKMT1A, PHB2, IPO5, PARP1, KPNA3, SLC25A15, PC | 45 | 2.27 | 1.99E-04 |
| 3 | GO cellular component | GO:0031975 | envelope | EIF6, COX11, CKMT1B, UQCRC1, SAMM50, NDUFB7, CBX3, ATP5G1, WTAP, NDUFS6, HTRA2, MTCH2, SH3GLB1, RAE1, TOR1A, SLC25A22, MTCH1, ATP5L, NUP37, NDUFS3, HADH, TNPO1, DNAJC19, ATP5L2, ATP5J2, TMCO6, NDUFB10, NDUFA9, CYCS, NDUFC2, MUL1, CLIC1, NUP155, VDAC2, SIGMAR1, VDAC3, SOD2, SENP2, SDHB, CKMT1A, PHB2, IPO5, PARP1, KPNA3, SLC25A15, PC | 45 | 2.27 | 2.19E-04 |
| 3 | GO cellular component | GO:0015934 | large ribosomal subunit | MRPL11, RPL18, MRPL3, MRPL12, MRPL41, RPL18A, MRPL27, MRPS24, MRPL36, RPL3, RPL19P12, MRPL32, RPL28 | 13 | 6.08 | 4.62E-04 |
| 3 | GO cellular component | GO:0005762 | mitochondrial large ribosomal subunit | MRPL11, MRPL12, MRPL3, MRPL41, MRPL27, MRPS24, MRPL36 | 7 | 12.18 | 5.47E-03 |
| 3 | GO cellular component | GO:0000315 | organellar large ribosomal subunit | MRPL11, MRPL12, MRPL3, MRPL41, MRPL27, MRPS24, MRPL36 | 7 | 12.18 | 5.47E-03 |
| 3 | GO cellular component | GO:0019866 | organelle inner membrane | EIF6, COX11, UQCRC1, CKMT1B, NDUFB7, CBX3, ATP5G1, NDUFS6, MTCH2, MTCH1, SLC25A22, ATP5L, NDUFS3, HADH, DNAJC19, ATP5L2, ATP5J2, NDUFB10, NDUFA9, NDUFC2, SIGMAR1, VDAC3, SOD2, SDHB, CKMT1A, PHB2, SLC25A15, PC | 27 | 2.57 | 7.76E-03 |
| 3 | GO cellular component | GO:0005730 | nucleolus | EIF6, POLR2F, SYVN1, ZBTB11, BOP1, WTAP, ZNF207, TSPYL1, EBNA1BP2, SBDS, MAK16, TARDBP, SMARCD1, BRIX1, RPL3, BRD4, DEDD2, PABPN1, EXOSC8, TSEN54, TPX2, MTA1, NUSAP1, MBD4, SNW1, ARID1B, RPF1, ZNF22, RSL1D1, DDX56, PA2G4, MED4, RPS19, NOP16, IPO5, RPS13, FLII, DNAJB1, AKAP8, HNRNPH1, PARP1, PARP2, MYBBP1A, WDR43 | 44 | 1.97 | 9.81E-03 |
| 3 | GO cellular component | GO:0005740 | mitochondrial envelope | COX11, UQCRC1, CKMT1B, SAMM50, NDUFB7, ATP5G1, NDUFS6, MTCH2, HTRA2, SH3GLB1, MTCH1, SLC25A22, ATP5L, NDUFS3, HADH, DNAJC19, ATP5L2, ATP5J2, NDUFB10, NDUFA9, CYCS, NDUFC2, MUL1, VDAC2, VDAC3, SOD2, SDHB, CKMT1A, PHB2, SLC25A15, PC | 30 | 2.24 | 2.96E-02 |
| 3 | GO cellular component | GO:0031090 | organelle membrane | EIF6, COX11, CLTA, UQCRC1, CKMT1B, AP1G1, VAPB, RP9, CBX3, PEX3, WTAP, NDUFS6, HTRA2, RAE1, SLC25A22, VPS4B, ATP5L, VPS4A, NDUFS3, ATP6V0D1, HADH, DNAJC19, NDUFB10, NDUFC2, KRTCAP2, SIGMAR1, KIF1B, CHST7, ARL8A, AMFR, DOLPP1, SAMM50, NDUFB7, CAMK2G, ATP5G1, MTCH2, SH3GLB1, SERINC1, MTCH1, TOR1A, TRAM1, ATP5L2, PLP2, ATP5J2, NDUFA9, MUL1, ARFIP1, VDAC2, VDAC3, GABARAP, DOLK, SOD2, SDHB, CKMT1A, PHB2, SPCS1, SLC25A15, SSR2, PC, COG2 | 59 | 1.69 | 3.13E-02 |
| 3 | GO cellular component | GO:0033177 | proton-transporting two-sector ATPase complex, proton-transporting domain | ATP6V0C, ATP5J2, ATP5L, ATP5G1, ATP6V0D1, ATP6V0B, ATP5L2 | 7 | 9.14 | 3.32E-02 |
| 3 | GO cellular component | GO:0044445 | cytosolic part | RPL18, CCT6A, RPS5, RPL28, RPS19, RPL18A, PFDN6, PFDN5, RPL3, RPS13, PDRG1, RPS11, RPL19P12, IKBKB, RPS24, EDARADD | 16 | 3.30 | 4.04E-02 |
| 3 | GO cellular component | GO:0000502 | proteasome complex | PSMA2, PSMB5, ADRM1, PSMA1, PSMD14, PSMB6, PSMA3, PSMD3, PSME3, PSMA7 | 10 | 5.14 | 4.89E-02 |
| 3 | GO cellular component | GO:0005743 | mitochondrial inner membrane | COX11, CKMT1B, UQCRC1, NDUFB7, ATP5G1, NDUFS6, MTCH2, SLC25A22, MTCH1, ATP5L, HADH, NDUFS3, DNAJC19, ATP5L2, ATP5J2, NDUFB10, NDUFA9, NDUFC2, VDAC3, SOD2, SDHB, CKMT1A, PHB2, SLC25A15, PC | 24 | 2.46 | 4.91E-02 |
| 4 | GO cellular component | GO:0030529 | ribonucleoprotein complex | ABCF1, RPL17, SRP14, GAR1, SNRPD3, RPL15, LSM7, SKIV2L2, RPS2, MRPS31, RPS3, RBM8A, RPS3A, PCBP1, RPLP0, DDX20, IMP4, MRPL34, FTL, EMG1, SF1, PRPF3, RRP9, SRPRB, MRPS7, PRPF6, EIF4A3, RPS15, UBB, SNRPF, MRPL46, SRP9, SERP1, MRPS15, MRPS12, SNRPB2, RPL35, IVNS1ABP, MRPL20, MRPL10, TROVE2, MRPL15, RPL6, RPSAP58, PRPF8, NPM1, RPL5, RPL4, RPL7A, DDX41, GEMIN4, RPS9, ILF3, RPSAP9, RPL29, FXR1, SLBP, HNRNPH3, RHEB, LSM10, NOP56, RBM14, SNRNP25 | 62 | 5.13 | 3.99E-24 |
| 4 | GO cellular component | GO:0070013 | intracellular organelle lumen | ITGB3BP, PDP2, SNRPD3, NDUFAB1, CCT2, CTNNB1, WDR74, GTF2E1, RBM8A, DDX20, IMP4, MRPL34, CIB1, GNL3, ANAPC1, EMG1, USP1, ACTN1, RRP8, RRP9, PPP1CC, CLPX, RPS15, NLE1, SNRPF, MRPS15, MRPS12, ANAPC10, ATF1, DDX47, ZNF326, EIF3E, NPM1, TEAD4, EIF3L, NAT10, RPL5, GCDH, YEATS4, PHB, FOXRED2, IDH3B, RPS9, VDAC2, RPF2, VDAC1, FXR1, DDX51, COPS2, GAR1, LYAR, SKIV2L2, RPS2, DAXX, RPS19BP1, CASP3, MCM7, GRWD1, RPS3A, EXOSC2, SF1, PRPF3, POLR1B, ZFR, HMGA1, MBD1, GTF2B, MCM4, GTF2H1, EIF4A3, C1QBP, ZMIZ1, NOL11, UBB, RBM34, NUP98, POLR2I, RPL35, ERI1, IVNS1ABP, KIN, SMUG1, POLR2C, MRPL20, RPA2, MRPL10, NPAS2, PRPF8, LSG1, AATF, POU3F1, GEMIN4, POLR3K, GMNN, ATP5F1, ILF3, PWP2, SMC3, NOP56, ATP5A1, RBM14, RCN1 | 102 | 2.44 | 2.59E-16 |
| 4 | GO cellular component | GO:0043233 | organelle lumen | ITGB3BP, PDP2, SNRPD3, NDUFAB1, CCT2, CTNNB1, WDR74, GTF2E1, RBM8A, DDX20, IMP4, MRPL34, CIB1, GNL3, ANAPC1, EMG1, USP1, ACTN1, RRP8, RRP9, PPP1CC, CLPX, RPS15, NLE1, SNRPF, MRPS15, MRPS12, ANAPC10, ATF1, DDX47, ZNF326, EIF3E, NPM1, TEAD4, EIF3L, NAT10, RPL5, GCDH, YEATS4, PHB, FOXRED2, IDH3B, RPS9, VDAC2, RPF2, VDAC1, FXR1, DDX51, COPS2, GAR1, LYAR, SKIV2L2, RPS2, DAXX, RPS19BP1, CASP3, MCM7, GRWD1, RPS3A, EXOSC2, SF1, PRPF3, POLR1B, ZFR, HMGA1, MBD1, GTF2B, MCM4, GTF2H1, EIF4A3, C1QBP, ZMIZ1, NOL11, UBB, RBM34, NUP98, POLR2I, RPL35, ERI1, IVNS1ABP, KIN, SMUG1, POLR2C, MRPL20, RPA2, MRPL10, NPAS2, PRPF8, LSG1, AATF, POU3F1, GEMIN4, POLR3K, GMNN, ATP5F1, ILF3, PWP2, SMC3, NOP56, ATP5A1, RBM14, RCN1 | 102 | 2.39 | 1.31E-15 |
| 4 | GO cellular component | GO:0031974 | membrane-enclosed lumen | ITGB3BP, PDP2, SNRPD3, NDUFAB1, CCT2, CTNNB1, WDR74, GTF2E1, RBM8A, DDX20, IMP4, MRPL34, CIB1, GNL3, ANAPC1, EMG1, USP1, ACTN1, RRP8, RRP9, PPP1CC, CLPX, RPS15, NLE1, SNRPF, MRPS15, MRPS12, ANAPC10, ATF1, DDX47, ZNF326, EIF3E, NPM1, TEAD4, EIF3L, NAT10, RPL5, GCDH, YEATS4, PHB, FOXRED2, IDH3B, RPS9, VDAC2, RPF2, VDAC1, FXR1, DDX51, COPS2, GAR1, LYAR, SKIV2L2, RPS2, DAXX, RPS19BP1, CASP3, MCM7, GRWD1, RPS3A, EXOSC2, SF1, PRPF3, POLR1B, TIMM23, ZFR, HMGA1, MBD1, GTF2B, MCM4, GTF2H1, EIF4A3, C1QBP, ZMIZ1, NOL11, UBB, RBM34, NUP98, POLR2I, RPL35, ERI1, IVNS1ABP, KIN, SMUG1, POLR2C, MRPL20, RPA2, MRPL10, NPAS2, PRPF8, LSG1, AATF, POU3F1, GEMIN4, POLR3K, GMNN, ATP5F1, ILF3, SMC3, PWP2, NOP56, ATP5A1, RBM14, RCN1 | 103 | 2.36 | 1.65E-15 |
| 4 | GO cellular component | GO:0031981 | nuclear lumen | ITGB3BP, COPS2, GAR1, LYAR, SNRPD3, SKIV2L2, CCT2, RPS2, DAXX, RPS19BP1, CTNNB1, WDR74, GTF2E1, CASP3, MCM7, RPS3A, GRWD1, RBM8A, DDX20, IMP4, CIB1, GNL3, ANAPC1, EMG1, USP1, EXOSC2, SF1, ACTN1, PRPF3, RRP8, RRP9, POLR1B, PPP1CC, MCM4, ZFR, HMGA1, MBD1, GTF2B, GTF2H1, EIF4A3, ZMIZ1, RPS15, NLE1, NOL11, UBB, SNRPF, RBM34, NUP98, POLR2I, RPL35, ERI1, ANAPC10, IVNS1ABP, KIN, SMUG1, POLR2C, ATF1, RPA2, NPAS2, DDX47, ZNF326, EIF3E, PRPF8, NPM1, TEAD4, LSG1, EIF3L, NAT10, AATF, RPL5, POU3F1, GEMIN4, YEATS4, POLR3K, PHB, GMNN, RPS9, ILF3, RPF2, SMC3, PWP2, FXR1, NOP56, RBM14, DDX51 | 85 | 2.50 | 2.88E-13 |
| 4 | GO cellular component | GO:0005840 | ribosome | ABCF1, RPL17, MRPS15, MRPS12, RPL15, RPL35, RPS2, MRPS31, MRPL20, RPS3, MRPL10, MRPL15, RPL6, RPS3A, RPSAP58, RPLP0, NPM1, RPL5, RPL4, RPL7A, MRPL34, FTL, SF1, RPS9, MRPS7, RPSAP9, RPL29, RPS15, UBB, MRPL46, SERP1 | 30 | 5.95 | 6.82E-12 |
| 4 | GO cellular component | GO:0033279 | ribosomal subunit | RPL17, MRPS15, MRPS12, RPL35, RPS9, RPSAP9, MRPS7, RPS2, RPS3, RPL29, MRPL20, MRPL10, MRPL15, RPS3A, RPL6, RPSAP58, RPLP0, RPS15, NPM1, RPL5, UBB, RPL4, RPL7A, MRPL34, FTL | 24 | 7.99 | 7.44E-12 |
| 4 | GO cellular component | GO:0043228 | non-membrane-bounded organelle | ITGB3BP, RPL17, MPZL2, SNRPD3, RPL15, CCT2, AURKA, MRPS31, CTNNB1, WDR74, H2AFV, RPLP0, DDX20, IMP4, MRPL34, FTL, GNL3, EMG1, ACTN1, RRP8, RRP9, CLPX, NME1, RPS15, NLE1, MRPL46, MRPS15, MRPS12, BANF1, PSMB4, DDX47, RPL6, EIF3E, RPSAP58, NPM1, EIF3L, NAT10, RPL5, RPL7A, RPL4, TRAF5, RPS9, RPSAP9, VDAC2, RPF2, FXR1, VDAC1, H3F3C, DDX51, ABCF1, COPS2, GAR1, TLN2, LYAR, SKIV2L2, RPS2, DAXX, RPS19BP1, RPS3, ACTR3, CASP3, MCM7, BAG1, RPS3A, GRWD1, MAP1LC3B, GOPC, SYNJ2, TUBB8, LRRFIP1, EXOSC2, SF1, POLR1B, MRPS7, MCM4, ZFR, HMGA1, PURB, NOL11, UBB, MAPRE1, LIN54, SERP1, RBM34, NUP98, PLEK2, HAUS1, RPL35, ERI1, IVNS1ABP, SMUG1, MRPL20, SUMO3, RPA2, MRPL10, EZR, MRPL15, TUBA3C, AATF, GEMIN4, CENPN, ILF3, CEP63, SMC3, PWP2, RPL29, NOP56, RBM14, SSNA1, DNM2 | 109 | 1.79 | 3.99E-08 |
| 4 | GO cellular component | GO:0043232 | intracellular non-membrane-bounded organelle | ITGB3BP, RPL17, MPZL2, SNRPD3, RPL15, CCT2, AURKA, MRPS31, CTNNB1, WDR74, H2AFV, RPLP0, DDX20, IMP4, MRPL34, FTL, GNL3, EMG1, ACTN1, RRP8, RRP9, CLPX, NME1, RPS15, NLE1, MRPL46, MRPS15, MRPS12, BANF1, PSMB4, DDX47, RPL6, EIF3E, RPSAP58, NPM1, EIF3L, NAT10, RPL5, RPL7A, RPL4, TRAF5, RPS9, RPSAP9, VDAC2, RPF2, FXR1, VDAC1, H3F3C, DDX51, ABCF1, COPS2, GAR1, TLN2, LYAR, SKIV2L2, RPS2, DAXX, RPS19BP1, RPS3, ACTR3, CASP3, MCM7, BAG1, RPS3A, GRWD1, MAP1LC3B, GOPC, SYNJ2, TUBB8, LRRFIP1, EXOSC2, SF1, POLR1B, MRPS7, MCM4, ZFR, HMGA1, PURB, NOL11, UBB, MAPRE1, LIN54, SERP1, RBM34, NUP98, PLEK2, HAUS1, RPL35, ERI1, IVNS1ABP, SMUG1, MRPL20, SUMO3, RPA2, MRPL10, EZR, MRPL15, TUBA3C, AATF, GEMIN4, CENPN, ILF3, CEP63, SMC3, PWP2, RPL29, NOP56, RBM14, SSNA1, DNM2 | 109 | 1.79 | 3.99E-08 |
| 4 | GO cellular component | GO:0022626 | cytosolic ribosome | RPL35, RPS9, RPSAP9, MRPS7, RPS2, RPS3, RPL29, RPL6, RPS3A, RPLP0, RPSAP58, RPS15, RPL5, UBB, RPL7A, RPL4, FTL | 16 | 8.42 | 1.82E-07 |
| 4 | GO cellular component | GO:0005829 | cytosol | ITGB3BP, RPL17, SNRPD3, RPL15, CCT2, RPS2, RPS3, CTNNB1, CASP3, BAG1, RPS3A, RPLP0, RAB24, SPG21, DPP9, DDX20, PSMD6, CASP2, FTL, ANAPC1, MRPS7, HMGA1, PSMA5, NME1, TXN, RPS15, FARSA, MAPRE1, UBB, SNRPF, PREP, TALDO1, SRM, RPL35, MAPKAPK3, ANAPC10, FKBP1A, ATP6V1G1, BANF1, EZR, EIF3G, RPL6, EIF3E, RPSAP58, NPM1, EIF3K, PPP3CC, VPS35, UCK1, RPL5, RPL4, RPL7A, RPIA, GEMIN4, ENO1, PDZD11, RPS9, RPSAP9, CEP63, RPL29, APRT, YWHAG, EIF4E, PSMD11, EEF1E1, CCT8, RHEB, SSNA1, PHPT1 | 68 | 2.18 | 2.86E-07 |
| 4 | GO cellular component | GO:0005654 | nucleoplasm | ITGB3BP, GAR1, SNRPD3, RPS2, DAXX, RPS19BP1, CTNNB1, GTF2E1, CASP3, MCM7, RBM8A, DDX20, GNL3, CIB1, ANAPC1, USP1, PRPF3, POLR1B, PPP1CC, MBD1, HMGA1, GTF2B, MCM4, GTF2H1, EIF4A3, ZMIZ1, RPS15, UBB, SNRPF, NUP98, POLR2I, ANAPC10, IVNS1ABP, POLR2C, SMUG1, ATF1, RPA2, NPAS2, PRPF8, EIF3E, TEAD4, NPM1, LSG1, EIF3L, POU3F1, GEMIN4, YEATS4, POLR3K, PHB, GMNN, RBM14 | 51 | 2.46 | 1.43E-06 |
| 4 | GO cellular component | GO:0005730 | nucleolus | RBM34, COPS2, GAR1, LYAR, SNRPD3, RPL35, ERI1, SKIV2L2, CCT2, RPS2, SMUG1, RPS19BP1, WDR74, CASP3, DDX47, GRWD1, RPS3A, NPM1, EIF3L, RPL5, AATF, NAT10, IMP4, GEMIN4, GNL3, EMG1, SF1, EXOSC2, ACTN1, RPS9, RRP8, ILF3, RRP9, POLR1B, RPF2, MCM4, ZFR, PWP2, FXR1, NLE1, NOL11, NOP56, RBM14, DDX51 | 44 | 2.69 | 1.81E-06 |
| 4 | GO cellular component | GO:0044445 | cytosolic part | RPL35, RPS9, CCT2, RPSAP9, MRPS7, RPS2, RPS3, RPL29, CTNNB1, RPS3A, RPL6, RPSAP58, RPLP0, RPS15, RPL5, UBB, RPL7A, RPL4, ENO1, FTL | 19 | 5.33 | 5.95E-06 |
| 4 | GO cellular component | GO:0015934 | large ribosomal subunit | MRPL10, RPL17, MRPL15, RPL6, RPLP0, NPM1, RPL35, RPL5, RPL4, RPL7A, MRPL34, RPL29, MRPL20 | 13 | 8.27 | 1.51E-05 |
| 4 | GO cellular component | GO:0015935 | small ribosomal subunit | MRPS15, MRPS12, RPS9, MRPS7, RPSAP9, RPS2, RPS3, RPS3A, RPSAP58, NPM1, RPS15, UBB, FTL | 12 | 8.12 | 7.37E-05 |
| 4 | GO cellular component | GO:0005681 | spliceosome | SNRPD3, SNRPB2, SF1, SKIV2L2, PRPF3, IVNS1ABP, PRPF6, EIF4A3, RBM8A, PRPF8, RHEB, DDX20, DDX41, SNRPF, GEMIN4, SNRNP25 | 16 | 5.16 | 1.65E-04 |
| 4 | GO cellular component | GO:0022627 | cytosolic small ribosomal subunit | RPS3A, RPSAP58, RPS15, RPS9, UBB, RPSAP9, MRPS7, RPS2, RPS3, FTL | 9 | 9.59 | 1.21E-03 |
| 4 | GO cellular component | GO:0005739 | mitochondrion | MRPS15, PDP2, MRPS12, CYC1, NDUFAB1, TOMM20L, MRPS31, MTHFD1L, MRPL20, MTHFD1, MRPL10, CASP3, MRPL15, ARG2, AKR7A2, SYNJ2, MLXIP, FANCG, MRPL34, MTO1, DNAJC19, GCDH, PHB, COX8A, LYRM4, ATP5F1, IDH3B, ILF3, MRPS7, TIMM23, VDAC2, ABCB7, PPP1CC, MRM1, ABCB6, CLPX, VDAC1, NDUFA11, TMEM186, CHCHD10, PSMA6, C1QBP, SLC25A33, TXN, NDUFV2, SLC25A37, SLC25A19, ATP5A1, MRPL46 | 49 | 1.92 | 4.65E-03 |
| 4 | GO cellular component | GO:0044451 | nucleoplasm part | GAR1, POLR2I, IVNS1ABP, DAXX, POLR2C, ATF1, CTNNB1, GTF2E1, NPAS2, MCM7, RBM8A, PRPF8, EIF3E, TEAD4, NPM1, LSG1, DDX20, POU3F1, GEMIN4, YEATS4, POLR3K, PRPF3, PPP1CC, HMGA1, MBD1, GTF2B, GTF2H1, EIF4A3, ZMIZ1, RBM14 | 30 | 2.30 | 1.60E-02 |
| 4 | GO cellular component | GO:0044429 | mitochondrial part | MRPS15, PDP2, MRPS12, CYC1, NDUFAB1, TOMM20L, MRPL20, MRPL10, SYNJ2, MLXIP, DNAJC19, MRPL34, GCDH, PHB, COX8A, ATP5F1, IDH3B, PPP1CC, ABCB7, VDAC2, TIMM23, ABCB6, CLPX, NDUFA11, VDAC1, C1QBP, SLC25A33, NDUFV2, SLC25A37, SLC25A19, ATP5A1 | 31 | 2.22 | 2.28E-02 |
| 5 | GO cellular component | GO:0070013 | intracellular organelle lumen | XRCC5, NCBP2, MRPL40, E2F3, GRPEL1, UTP18, HR, SNRPD1, SNRPD2, NFKB1, TERF2IP, SENP5, IQGAP1, IMP3, PARN, GTF2A2, U2AF1, TFB2M, MRPL39, TWISTNB, HIST3H3, ZNF593, ACTN4, POLG, RAN, EXOSC4, POLR1A, EXOSC3, POLR1C, SKP1, UBE2C, MRTO4, MCM6, RFC5, CHMP1A, RPAIN, HIF1A, MRPS9, RCC2, RPS14, JUN, DDB2, NOL10, DDX31, RBM39, AARS2, LRPPRC, POLR2H, POLR2K, MRPS11, UTP6, C14ORF169, RPL36, WARS2, ZNRD1, POLR2B, KARS, EXOSC10, TFAM, SQSTM1, HSD17B4, RSL24D1, NUP54, TFDP1, BCAS2, UTP23, CSTF2, DDX5, NOP10, PPIF, MPG, PHF2, HDAC2, PCNA, POP4, RBPJ, HDAC8, POP7, VPS25 | 79 | 2.50 | 7.69E-13 |
| 5 | GO cellular component | GO:0031974 | membrane-enclosed lumen | XRCC5, NCBP2, MRPL40, E2F3, GRPEL1, UTP18, HR, SNRPD1, SNRPD2, NFKB1, TERF2IP, SENP5, IQGAP1, ARL2BP, IMP3, PARN, GTF2A2, U2AF1, TFB2M, MRPL39, TWISTNB, HIST3H3, ZNF593, ACTN4, POLG, RAN, EXOSC4, POLR1A, EXOSC3, POLR1C, SKP1, UBE2C, MRTO4, MCM6, RFC5, CHMP1A, RPAIN, HIF1A, MRPS9, RCC2, RPS14, JUN, DDB2, NOL10, DDX31, RBM39, AARS2, LRPPRC, POLR2H, POLR2K, MRPS11, UTP6, C14ORF169, RPL36, WARS2, ZNRD1, POLR2B, KARS, EXOSC10, TFAM, SQSTM1, HSD17B4, RSL24D1, NUP54, TFDP1, BCAS2, UTP23, CSTF2, DDX5, NOP10, PPIF, MPG, PHF2, HDAC2, PCNA, POP4, RBPJ, HDAC8, POP7, VPS25 | 80 | 2.43 | 2.44E-12 |
| 5 | GO cellular component | GO:0043233 | organelle lumen | XRCC5, NCBP2, MRPL40, E2F3, GRPEL1, UTP18, HR, SNRPD1, SNRPD2, NFKB1, TERF2IP, SENP5, IQGAP1, IMP3, PARN, GTF2A2, U2AF1, TFB2M, MRPL39, TWISTNB, HIST3H3, ZNF593, ACTN4, POLG, RAN, EXOSC4, POLR1A, EXOSC3, POLR1C, SKP1, UBE2C, MRTO4, MCM6, RFC5, CHMP1A, RPAIN, HIF1A, MRPS9, RCC2, RPS14, JUN, DDB2, NOL10, DDX31, RBM39, AARS2, LRPPRC, POLR2H, POLR2K, MRPS11, UTP6, C14ORF169, RPL36, WARS2, ZNRD1, POLR2B, KARS, EXOSC10, TFAM, SQSTM1, HSD17B4, RSL24D1, NUP54, TFDP1, BCAS2, UTP23, CSTF2, DDX5, NOP10, PPIF, MPG, PHF2, HDAC2, PCNA, POP4, RBPJ, HDAC8, POP7, VPS25 | 79 | 2.44 | 2.71E-12 |
| 5 | GO cellular component | GO:0031981 | nuclear lumen | XRCC5, NCBP2, E2F3, UTP18, HR, SNRPD1, SNRPD2, NFKB1, TERF2IP, SENP5, IQGAP1, IMP3, PARN, GTF2A2, U2AF1, TWISTNB, HIST3H3, ZNF593, ACTN4, RAN, EXOSC4, POLR1A, EXOSC3, POLR1C, SKP1, UBE2C, MRTO4, MCM6, RFC5, CHMP1A, RPAIN, HIF1A, RCC2, JUN, RPS14, NOL10, DDB2, DDX31, RBM39, LRPPRC, POLR2H, POLR2K, UTP6, C14ORF169, RPL36, ZNRD1, POLR2B, EXOSC10, SQSTM1, NUP54, RSL24D1, TFDP1, UTP23, BCAS2, CSTF2, DDX5, NOP10, MPG, HDAC2, PHF2, PCNA, POP4, RBPJ, HDAC8, POP7, VPS25 | 66 | 2.56 | 1.49E-10 |
| 5 | GO cellular component | GO:0030529 | ribonucleoprotein complex | MRPL40, UTP18, U2AF2, PPIL1, MRPS11, SNRPD1, RPL36, SNRPD2, IMP3, RPL30, RPL7, EDC3, U2AF1, DHX35, LSM2, RSL24D1, MRPL39, BCAS2, SNORA7B, MRPL50, UPF1, MRPS23, ACTN4, DDX5, NOP10, MRPS2, MRPS9, RPL13A, RPS17, RPS14, RPS10, POP4, PDCD7, POP7, TXNL4A | 35 | 3.83 | 7.93E-09 |
| 5 | GO cellular component | GO:0043228 | non-membrane-bounded organelle | XRCC5, MRPL40, UTP18, CCDC85B, FERMT1, TERF2IP, SENP5, ARL2BP, IQGAP1, FRMD1, DSTN, IMP3, PARN, FRMD5, KRT80, H2AFZ, SYNJ2, TFB2M, RANBP1, MRPL39, TWISTNB, HIST3H3, TUBA1A, HIST1H1E, SNORA7B, ZNF593, MRPL50, KIF5B, ACTN4, POLG, RAN, EXOSC4, POLR1A, EXOSC3, POLR1C, MRPS2, MRTO4, RFC5, CHMP1A, HIF1A, MRPS9, RCC2, RPS17, TBCA, ZWINT, RPS14, JUN, NOL10, DDX31, RPS10, RBM39, DYNLRB1, LRPPRC, KRTAP5-11, POLR2H, MRPS11, UTP6, C14ORF169, RPL36, ZNRD1, EXOSC10, TFAM, CDC42EP2, RPL30, RPL7, RSL24D1, BCAS2, UTP23, CENPN, CSTF2, UPF1, MRPS23, CENPP, NUF2, CENPK, DDX5, NOP10, TUBA8, PHF2, HDAC2, RPL13A, PCNA, HIST1H3C, SEC13, POP4, RBPJ, HIST1H3G, HDAC8, POP7 | 88 | 1.91 | 5.39E-08 |
| 5 | GO cellular component | GO:0043232 | intracellular non-membrane-bounded organelle | XRCC5, MRPL40, UTP18, CCDC85B, FERMT1, TERF2IP, SENP5, ARL2BP, IQGAP1, FRMD1, DSTN, IMP3, PARN, FRMD5, KRT80, H2AFZ, SYNJ2, TFB2M, RANBP1, MRPL39, TWISTNB, HIST3H3, TUBA1A, HIST1H1E, SNORA7B, ZNF593, MRPL50, KIF5B, ACTN4, POLG, RAN, EXOSC4, POLR1A, EXOSC3, POLR1C, MRPS2, MRTO4, RFC5, CHMP1A, HIF1A, MRPS9, RCC2, RPS17, TBCA, ZWINT, RPS14, JUN, NOL10, DDX31, RPS10, RBM39, DYNLRB1, LRPPRC, KRTAP5-11, POLR2H, MRPS11, UTP6, C14ORF169, RPL36, ZNRD1, EXOSC10, TFAM, CDC42EP2, RPL30, RPL7, RSL24D1, BCAS2, UTP23, CENPN, CSTF2, UPF1, MRPS23, CENPP, NUF2, CENPK, DDX5, NOP10, TUBA8, PHF2, HDAC2, RPL13A, PCNA, HIST1H3C, SEC13, POP4, RBPJ, HIST1H3G, HDAC8, POP7 | 88 | 1.91 | 5.39E-08 |
| 5 | GO cellular component | GO:0005730 | nucleolus | POLR2H, UTP18, UTP6, RPL36, C14ORF169, ZNRD1, SENP5, IQGAP1, EXOSC10, IMP3, PARN, RSL24D1, TWISTNB, BCAS2, UTP23, ZNF593, CSTF2, ACTN4, EXOSC4, POLR1A, EXOSC3, POLR1C, DDX5, NOP10, MRTO4, PHF2, HIF1A, RCC2, RPS14, NOL10, DDX31, POP4, RBPJ, POP7 | 34 | 2.74 | 6.45E-05 |
| 5 | GO cellular component | GO:0005829 | cytosol | NCBP2, TRAF2, CNBP, NARS, CHMP4B, BCAR1, UBE2G2, SNRPD1, SNRPD2, HK1, RPL36, PPT1, NFKB1, ILDR1, KARS, LOC407835, PSMF1, GPX1, PSMB7, RPL30, RPL7, SQSTM1, EIF3J, TUBA1A, CABLES1, IMPDH2, GABARAPL2, SNORA7B, DNM1L, AIMP1, VHL, RAN, EPRS, SKP1, UBE2C, PARK7, PTPN12, CIDEC, TARS, PFDN2, PSMD12, RPL13A, RPS17, RPS14, JUN, PFDN4, EIF2S2, EIF4A1, PGM1, RPS10 | 50 | 2.12 | 1.28E-04 |
| 5 | GO cellular component | GO:0005840 | ribosome | MRPL40, SNORA7B, MRPL50, MRPS23, MRPS11, RPL36, MRPS2, IMP3, RPL30, MRPS9, RPL7, RPL13A, RPS17, RPS14, RPS10, MRPL39, RSL24D1 | 17 | 4.45 | 4.04E-04 |
| 5 | GO cellular component | GO:0005654 | nucleoplasm | XRCC5, POLR2H, NCBP2, E2F3, POLR2K, HR, SNRPD1, SNRPD2, C14ORF169, NFKB1, TERF2IP, POLR2B, SQSTM1, GTF2A2, U2AF1, NUP54, HIST3H3, TFDP1, RAN, POLR1A, POLR1C, SKP1, UBE2C, NOP10, MCM6, RFC5, MPG, RPAIN, HDAC2, HIF1A, JUN, PCNA, DDB2, RBM39, HDAC8, LRPPRC, VPS25 | 37 | 2.36 | 5.87E-04 |
| 5 | GO cellular component | GO:0005739 | mitochondrion | MRPL40, GRPEL1, MRPS11, HK1, SFXN4, ATP5G2, WARS2, COX5A, NDUFAF1, ARL2BP, KARS, TFAM, GPX1, TOMM6, SYNJ2, TFB2M, GCSH, HSD17B4, MRPL39, SCO2, SCO1, DNM1L, MRPL50, MRPS23, POLG, VHL, COX4I1, TMEM126A, COQ9, ISOC2, PARK7, MRPS2, COQ5, PPIF, PRELID1, MRPS9, YME1L1, AARS2, LRPPRC, MGST1, UQCRB, VPS25 | 42 | 2.18 | 7.69E-04 |
| 5 | GO cellular component | GO:0044429 | mitochondrial part | MRPL40, GRPEL1, MRPS11, HK1, WARS2, ATP5G2, COX5A, NDUFAF1, KARS, ARL2BP, TFAM, TOMM6, TFB2M, SYNJ2, MRPL39, SCO2, SCO1, DNM1L, POLG, COX4I1, PPIF, MRPS9, AARS2, LRPPRC, MGST1, UQCRB | 26 | 2.46 | 1.57E-02 |
| 5 | GO cellular component | GO:0005694 | chromosome | XRCC5, CENPN, HIST1H1E, UPF1, RAN, CENPP, NUF2, TERF2IP, CENPK, RFC5, CHMP1A, HDAC2, RCC2, ZWINT, JUN, PCNA, H2AFZ, SEC13, HIST1H3C, HIST3H3, HDAC8, HIST1H3G, LRPPRC | 22 | 2.69 | 2.01E-02 |
| 5 | GO cellular component | GO:0033279 | ribosomal subunit | RPL30, SNORA7B, MRPS9, RPL7, RPL13A, RPS17, RPS14, MRPS11, RPL36, RPS10, MRPS2 | 11 | 4.84 | 2.67E-02 |
| 5 | GO cellular component | GO:0005681 | spliceosome | BCAS2, U2AF2, PPIL1, SNRPD1, U2AF1, SNRPD2, DHX35, LSM2, DDX5, PDCD7, TXNL4A | 11 | 4.69 | 3.44E-02 |
| 6 | GO cellular component | GO:0031974 | membrane-enclosed lumen | TAF1C, MEF2B, ELF4, TCOF1, YLPM1, DNAJC10, HMGN5, MED21, SRP19, APOBEC3F, RTN3, SMNDC1, WDR75, CCNE1, ZNF350, MUTYH, P4HA2, DGCR8, MCCC1, HMOX1, PRIM2, CREG1, NDUFS1, NUDT16, ELP3, ELP2, RBBP4, LBX1, AIFM1, YY1, APTX, CDC5L, NDUFA10, TIMM8A, TAF10, TAF12, NAV2, PRKRIP1, SMARCA5, PCCA, ING5, ME3, MRPS14, GLUD1, FKBP4, NOC3L, SETD1A, COIL, BMS1, POLR2A, HNRNPA3, SUMO1, WDR55, POU2F1, HSPE1, TCEA1, USP36, COX6B2, FAM32A, PDK1, NKRF, MYO1C, EME1, TRIM27, SIRT5, SSB, TRIM25, IGF2, DACH1, MED13L, PHAX, DUSP4, ATF4, ATF3, GLS, SFPQ, SMARCC2, SUMF1, NOP58 | 79 | 1.72 | 3.47E-04 |
| 6 | GO cellular component | GO:0070013 | intracellular organelle lumen | TAF1C, MEF2B, ELF4, TCOF1, YLPM1, DNAJC10, HMGN5, MED21, SRP19, APOBEC3F, SMNDC1, RTN3, WDR75, CCNE1, ZNF350, MUTYH, P4HA2, DGCR8, MCCC1, HMOX1, PRIM2, CREG1, NUDT16, ELP3, ELP2, RBBP4, LBX1, AIFM1, YY1, APTX, CDC5L, NDUFA10, TAF10, TAF12, NAV2, PRKRIP1, SMARCA5, PCCA, ING5, ME3, MRPS14, GLUD1, FKBP4, NOC3L, SETD1A, COIL, BMS1, POLR2A, HNRNPA3, SUMO1, WDR55, POU2F1, HSPE1, TCEA1, USP36, FAM32A, PDK1, NKRF, MYO1C, EME1, TRIM27, SIRT5, SSB, TRIM25, IGF2, DACH1, MED13L, PHAX, DUSP4, ATF4, ATF3, GLS, SFPQ, SMARCC2, SUMF1, NOP58 | 76 | 1.72 | 5.36E-04 |
| 6 | GO cellular component | GO:0043233 | organelle lumen | TAF1C, MEF2B, ELF4, TCOF1, YLPM1, DNAJC10, HMGN5, MED21, SRP19, APOBEC3F, SMNDC1, RTN3, WDR75, CCNE1, ZNF350, MUTYH, P4HA2, DGCR8, MCCC1, HMOX1, PRIM2, CREG1, NUDT16, ELP3, ELP2, RBBP4, LBX1, AIFM1, YY1, APTX, CDC5L, NDUFA10, TAF10, TAF12, NAV2, PRKRIP1, SMARCA5, PCCA, ING5, ME3, MRPS14, GLUD1, FKBP4, NOC3L, SETD1A, COIL, BMS1, POLR2A, HNRNPA3, SUMO1, WDR55, POU2F1, HSPE1, TCEA1, USP36, FAM32A, PDK1, NKRF, MYO1C, EME1, TRIM27, SIRT5, SSB, TRIM25, IGF2, DACH1, MED13L, PHAX, DUSP4, ATF4, ATF3, GLS, SFPQ, SMARCC2, SUMF1, NOP58 | 76 | 1.68 | 1.27E-03 |
| 6 | GO cellular component | GO:0031981 | nuclear lumen | MEF2B, TAF1C, ELF4, YLPM1, TCOF1, HMGN5, MED21, SRP19, APOBEC3F, SMNDC1, RTN3, WDR75, CCNE1, ZNF350, MUTYH, DGCR8, HMOX1, PRIM2, CREG1, NUDT16, ELP3, ELP2, RBBP4, LBX1, YY1, APTX, CDC5L, TAF10, TAF12, NAV2, PRKRIP1, SMARCA5, ING5, FKBP4, SETD1A, NOC3L, COIL, BMS1, POLR2A, HNRNPA3, SUMO1, WDR55, POU2F1, TCEA1, USP36, FAM32A, NKRF, MYO1C, EME1, TRIM27, SSB, TRIM25, DACH1, MED13L, DUSP4, PHAX, ATF4, ATF3, SFPQ, SMARCC2, NOP58 | 61 | 1.70 | 1.36E-02 |
| 6 | GO cellular component | GO:0005730 | nucleolus | FKBP4, NOC3L, TCOF1, HMGN5, SRP19, COIL, BMS1, APOBEC3F, RTN3, POLR2A, HNRNPA3, WDR75, ZNF350, WDR55, DGCR8, HMOX1, USP36, FAM32A, NKRF, NUDT16, ELP3, ELP2, MYO1C, EME1, APTX, SSB, TRIM25, CDC5L, ATF3, TAF12, NAV2, PRKRIP1, SMARCC2, SMARCA5, NOP58 | 35 | 2.02 | 4.24E-02 |
| 9 | GO cellular component | GO:0031974 | membrane-enclosed lumen | ATP5E, PDIA3, QARS, PDHB, AKT1, ESF1, KLHL7, PSKH1, CSNK2A1, DNAJB11, CPOX, SRRM2, RPP30, POLG2, DHX30, TPR, TSEN15, ATF7IP, TFIP11, CDC7, SYMPK, CBR4, TOP1MT, TOX4, SP140, TIMM8B, SUZ12, CYP27A1, FANCD2, CFL1, SIX1, UGGT2, HIST1H4L, ZCRB1, CALR, NR2C2, WDR55, METTL3, HJURP, IVD, GTF3C6, MRPL55, SUPT4H1, GEMIN6, NSUN2, RBM28, BUB3, TSR1, TAF6, MRPS22, NLK, TP53BP1, FDXR, CENPF, ATR, CDC27, FXR1, NOP14, MNAT1, SON, PPIG, SP1, POLD1, RNF2, NCOR2 | 65 | 1.83 | 3.03E-04 |
| 9 | GO cellular component | GO:0070013 | intracellular organelle lumen | ATP5E, PDIA3, QARS, PDHB, AKT1, ESF1, KLHL7, PSKH1, CSNK2A1, DNAJB11, SRRM2, RPP30, POLG2, DHX30, TPR, TSEN15, ATF7IP, TFIP11, CDC7, SYMPK, CBR4, TOP1MT, TOX4, SP140, SUZ12, CYP27A1, FANCD2, CFL1, SIX1, UGGT2, HIST1H4L, ZCRB1, CALR, NR2C2, WDR55, METTL3, HJURP, IVD, GTF3C6, MRPL55, SUPT4H1, GEMIN6, NSUN2, RBM28, BUB3, TSR1, TAF6, MRPS22, NLK, TP53BP1, FDXR, CENPF, ATR, CDC27, FXR1, NOP14, MNAT1, SON, PPIG, SP1, POLD1, RNF2, NCOR2 | 63 | 1.85 | 3.32E-04 |
| 9 | GO cellular component | GO:0043233 | organelle lumen | ATP5E, PDIA3, QARS, PDHB, AKT1, ESF1, KLHL7, PSKH1, CSNK2A1, DNAJB11, SRRM2, RPP30, POLG2, DHX30, TPR, TSEN15, ATF7IP, TFIP11, CDC7, SYMPK, CBR4, TOP1MT, TOX4, SP140, SUZ12, CYP27A1, FANCD2, CFL1, SIX1, UGGT2, HIST1H4L, ZCRB1, CALR, NR2C2, WDR55, METTL3, HJURP, IVD, GTF3C6, MRPL55, SUPT4H1, GEMIN6, NSUN2, RBM28, BUB3, TSR1, TAF6, MRPS22, NLK, TP53BP1, FDXR, CENPF, ATR, CDC27, FXR1, NOP14, MNAT1, SON, PPIG, SP1, POLD1, RNF2, NCOR2 | 63 | 1.81 | 7.20E-04 |
| 9 | GO cellular component | GO:0043228 | non-membrane-bounded organelle | PRPF4B, CROCC, RHOQ, AKT1, ESF1, KLHL7, PSKH1, CSNK2A1, RPP30, POLG2, TPR, DHX30, TSEN15, SYMPK, NUP133, TMSB10, TOP1MT, H2AFJ, HMGA2, TOX4, SP140, ELMO2, EML4, SUZ12, TNNT1, RPL41, BAZ1B, FANCD2, FGFR1OP, TBCD, CFL1, SIX1, SEMA4C, DYNC1I2, DNAH11, HIST1H4L, HAUS5, SHROOM4, BBS9, CETN3, ARPC5, RPL13AP3, KRTAP10-12, NR2C2, GPHN, WDR55, CEP57, HJURP, RAC3, BUB1, MRPL55, RBM28, NSUN2, BUB3, SMCHD1, TSR1, MRPS22, NLK, TP53BP1, ALMS1, CENPF, ATR, MRPL30, CDC27, FXR1, TRADD, NOP14, MYO10, FNBP1, SYNE2, SP1, SPRR1A, RNF2, NCOR2, DNM1, CDC42BPB | 76 | 1.53 | 1.99E-02 |
| 9 | GO cellular component | GO:0043232 | intracellular non-membrane-bounded organelle | PRPF4B, CROCC, RHOQ, AKT1, ESF1, KLHL7, PSKH1, CSNK2A1, RPP30, POLG2, TPR, DHX30, TSEN15, SYMPK, NUP133, TMSB10, TOP1MT, H2AFJ, HMGA2, TOX4, SP140, ELMO2, EML4, SUZ12, TNNT1, RPL41, BAZ1B, FANCD2, FGFR1OP, TBCD, CFL1, SIX1, SEMA4C, DYNC1I2, DNAH11, HIST1H4L, HAUS5, SHROOM4, BBS9, CETN3, ARPC5, RPL13AP3, KRTAP10-12, NR2C2, GPHN, WDR55, CEP57, HJURP, RAC3, BUB1, MRPL55, RBM28, NSUN2, BUB3, SMCHD1, TSR1, MRPS22, NLK, TP53BP1, ALMS1, CENPF, ATR, MRPL30, CDC27, FXR1, TRADD, NOP14, MYO10, FNBP1, SYNE2, SP1, SPRR1A, RNF2, NCOR2, DNM1, CDC42BPB | 76 | 1.53 | 1.99E-02 |
| 9 | GO cellular component | GO:0005829 | cytosol | PSMB10, CHMP2A, ATOX1, DTYMK, QARS, EGLN1, CALR, TK1, AKT1, TYMS, SPRY1, CEP57, EIF3H, PDE4A, TICAM1, DIABLO, PSMD4, PABPC1, GEMIN6, TPRKB, RASA4, BUB3, GLRX, PIK3C2B, SPHK1, DUSP23, ALMS1, SNAPIN, STXBP3, ACACB, TPMT, CDC27, GMPS, RALGDS, TRADD, NFU1, ISCU, NDOR1, CCT4, CAPN10, GBE1, RPL41, SERPINB6, RIPK1, FGFR1OP, DYNC1I2 | 46 | 1.80 | 2.84E-02 |
| 10 | GO cellular component | GO:0070469 | respiratory chain | SDHA, NDUFA4, NDUFS7, ND4L, NDUFS5, NDUFS4, UQCRH, COX2, COX1, ND3, NDUFC2, NDUFA13, ND6 | 13 | 9.31 | 4.00E-06 |
| 10 | GO cellular component | GO:0031966 | mitochondrial membrane | MAVS, GPAT2, NDUFS7, NDUFS5, NDUFS4, SLC25A25, GPX4, SLC25A46, SLC25A29, SLC25A44, ND6, BDH1, ATP8, ATP6, TOMM34, NDUFA4, SUCLG1, ND3, NDUFC2, NDUFA13, BAD, SDHA, TST, ND4L, COX3, UQCRH, COX2, COX1, TOMM40L | 27 | 3.68 | 6.64E-06 |
| 10 | GO cellular component | GO:0005740 | mitochondrial envelope | MAVS, GPAT2, NDUFS7, NDUFS5, NDUFS4, SLC25A25, GPX4, SLC25A46, SLC25A29, SLC25A44, ND6, BDH1, ATP8, ATP6, TOMM34, NDUFA4, SUCLG1, ND3, NDUFC2, NDUFA13, BAD, SDHA, TST, ND4L, COX3, UQCRH, COX2, COX1, TOMM40L | 27 | 3.46 | 2.30E-05 |
| 10 | GO cellular component | GO:0070013 | intracellular organelle lumen | TAF1B, MMS19, TCOF1, PDLIM3, SNRPD1, RPS6KB2, CCNE2, INTS4, INTS6, YAP1, SAP30L, FTSJ3, BRF1, EXOSC9, MTA2, SUCLG1, ADNP, MED11, NDUFA13, MED14, HES6, MBD2, SARS2, PTRF, SMARCE1, HIPK1, PIAS3, SH3KBP1, CSTB, EDF1, KPNA2, CTDP1, NOL12, ACAA1, DEAF1, GLTSCR2, HMGB1, FZR1, SDAD1, RPA2, ERCC5, KDELC1, SMARCB1, HARS2, MUS81, TAF9, CC2D1A, HSPA5, BDH1, ACTB, SMAD6, GOLIM4, DLAT, SIRT7, TST, POLD4, POLRMT, PAPOLA, NUP62, VCP, THRAP3, KIF20B, TBL1X, HDAC6 | 64 | 1.93 | 5.52E-05 |
| 10 | GO cellular component | GO:0005746 | mitochondrial respiratory chain | SDHA, NDUFA4, NDUFS7, ND4L, NDUFS5, NDUFS4, UQCRH, ND3, NDUFC2, NDUFA13, ND6 | 11 | 9.23 | 8.61E-05 |
| 10 | GO cellular component | GO:0005743 | mitochondrial inner membrane | NDUFA4, SUCLG1, ND3, NDUFC2, NDUFA13, SDHA, NDUFS7, TST, ND4L, NDUFS5, NDUFS4, COX3, SLC25A25, COX2, UQCRH, COX1, GPX4, SLC25A46, SLC25A29, SLC25A44, ATP8, ND6, BDH1, ATP6 | 22 | 3.86 | 8.69E-05 |
| 10 | GO cellular component | GO:0031090 | organelle membrane | MAVS, ARFGAP1, ACOX1, AP1B1, ALG6, GPAT2, NDUFS7, COPB2, NDUFS5, NDUFS4, SLC25A25, GP1BB, GPX4, SLC25A46, SLC25A29, SLC25A44, HSPA5, ATP8, FNDC3A, BDH1, ND6, RAB21, ATP6, TOMM34, B4GALNT1, NDUFA4, SCAMP3, SEC11C, SUCLG1, ND3, NDUFC2, NDUFA13, PIGT, BAD, SDHA, TST, SLC17A7, ND4L, AP2A2, COX3, NUP62, COX2, UQCRH, COX1, TOMM40L, NUCB2, CUX1, SLC27A5 | 46 | 2.25 | 9.55E-05 |
| 10 | GO cellular component | GO:0043233 | organelle lumen | TAF1B, MMS19, TCOF1, PDLIM3, SNRPD1, RPS6KB2, CCNE2, INTS4, INTS6, YAP1, SAP30L, FTSJ3, BRF1, EXOSC9, MTA2, SUCLG1, ADNP, MED11, NDUFA13, MED14, HES6, MBD2, SARS2, PTRF, SMARCE1, HIPK1, PIAS3, SH3KBP1, CSTB, EDF1, KPNA2, CTDP1, NOL12, ACAA1, DEAF1, GLTSCR2, HMGB1, FZR1, SDAD1, RPA2, ERCC5, KDELC1, SMARCB1, HARS2, MUS81, TAF9, CC2D1A, HSPA5, BDH1, ACTB, SMAD6, GOLIM4, DLAT, SIRT7, TST, POLD4, POLRMT, PAPOLA, NUP62, VCP, THRAP3, KIF20B, TBL1X, HDAC6 | 64 | 1.89 | 1.26E-04 |
| 10 | GO cellular component | GO:0044429 | mitochondrial part | MAVS, GPAT2, NDUFS7, NDUFS5, NDUFS4, SLC25A25, GPX4, HARS2, SLC25A46, SLC25A29, SLC25A44, ATP8, BDH1, ND6, ATP6, TOMM34, NDUFA4, SUCLG1, ND3, NDUFC2, NDUFA13, DLAT, BAD, SARS2, SDHA, TST, POLRMT, ND4L, COX3, UQCRH, COX2, COX1, TOMM40L | 31 | 2.80 | 2.02E-04 |
| 10 | GO cellular component | GO:0031974 | membrane-enclosed lumen | TAF1B, MMS19, TCOF1, PDLIM3, SNRPD1, RPS6KB2, CCNE2, INTS4, INTS6, YAP1, SAP30L, FTSJ3, BRF1, EXOSC9, MTA2, SUCLG1, ADNP, MED11, NDUFA13, MED14, HES6, MBD2, SARS2, PTRF, SMARCE1, HIPK1, PIAS3, SH3KBP1, CSTB, EDF1, KPNA2, CTDP1, NOL12, ACAA1, DEAF1, GLTSCR2, HMGB1, FZR1, SDAD1, RPA2, ERCC5, KDELC1, SMARCB1, HARS2, MUS81, TAF9, CC2D1A, HSPA5, BDH1, ACTB, SMAD6, GOLIM4, DLAT, SIRT7, TST, POLD4, POLRMT, PAPOLA, NUP62, VCP, THRAP3, KIF20B, TBL1X, HDAC6 | 64 | 1.85 | 2.53E-04 |
| 10 | GO cellular component | GO:0019866 | organelle inner membrane | NDUFA4, SUCLG1, ND3, NDUFC2, NDUFA13, SDHA, NDUFS7, TST, ND4L, NDUFS5, NDUFS4, COX3, SLC25A25, COX2, UQCRH, COX1, GPX4, SLC25A46, SLC25A29, SLC25A44, ATP8, ND6, BDH1, ATP6 | 22 | 3.59 | 2.88E-04 |
| 10 | GO cellular component | GO:0045271 | respiratory chain complex I | NDUFA4, NDUFS7, ND4L, NDUFS5, NDUFS4, ND3, NDUFC2, NDUFA13, ND6 | 9 | 11.51 | 2.93E-04 |
| 10 | GO cellular component | GO:0005747 | mitochondrial respiratory chain complex I | NDUFA4, NDUFS7, ND4L, NDUFS5, NDUFS4, ND3, NDUFC2, NDUFA13, ND6 | 9 | 11.51 | 2.93E-04 |
| 10 | GO cellular component | GO:0030964 | NADH dehydrogenase complex | NDUFA4, NDUFS7, ND4L, NDUFS5, NDUFS4, ND3, NDUFC2, NDUFA13, ND6 | 9 | 11.51 | 2.93E-04 |
| 10 | GO cellular component | GO:0031967 | organelle envelope | MAVS, GPAT2, NDUFS7, NDUFS5, NDUFS4, SLC25A25, GPX4, SLC25A46, SLC25A29, SLC25A44, ATP8, BDH1, ND6, ATP6, TOMM34, NDUFA4, SUCLG1, ND3, NDUFC2, NDUFA13, BAD, SDHA, TST, ND4L, COX3, NUP62, NRM, UQCRH, COX2, COX1, NUCB2, TOMM40L, KPNA2 | 31 | 2.69 | 4.76E-04 |
| 10 | GO cellular component | GO:0031975 | envelope | MAVS, GPAT2, NDUFS7, NDUFS5, NDUFS4, SLC25A25, GPX4, SLC25A46, SLC25A29, SLC25A44, ATP8, BDH1, ND6, ATP6, TOMM34, NDUFA4, SUCLG1, ND3, NDUFC2, NDUFA13, BAD, SDHA, TST, ND4L, COX3, NUP62, NRM, UQCRH, COX2, COX1, NUCB2, TOMM40L, KPNA2 | 31 | 2.68 | 5.08E-04 |
| 10 | GO cellular component | GO:0031981 | nuclear lumen | TAF1B, MMS19, TCOF1, SNRPD1, PDLIM3, RPS6KB2, CCNE2, INTS4, INTS6, YAP1, SAP30L, FTSJ3, BRF1, EXOSC9, MTA2, ADNP, NDUFA13, MED11, MED14, HES6, MBD2, PTRF, SMARCE1, HIPK1, PIAS3, SH3KBP1, CSTB, EDF1, KPNA2, CTDP1, NOL12, DEAF1, GLTSCR2, HMGB1, FZR1, SDAD1, RPA2, ERCC5, SMARCB1, MUS81, TAF9, CC2D1A, ACTB, SMAD6, SIRT7, POLD4, PAPOLA, NUP62, VCP, THRAP3, KIF20B, TBL1X, HDAC6 | 53 | 1.96 | 6.39E-04 |
| 10 | GO cellular component | GO:0005654 | nucleoplasm | DEAF1, TAF1B, MMS19, HMGB1, FZR1, SNRPD1, RPS6KB2, CCNE2, RPA2, ERCC5, INTS4, SMARCB1, INTS6, TAF9, YAP1, ACTB, BRF1, SMAD6, MTA2, MED11, NDUFA13, MED14, HES6, MBD2, POLD4, PTRF, SMARCE1, HIPK1, PIAS3, THRAP3, KIF20B, EDF1, TBL1X, KPNA2, CTDP1, HDAC6 | 36 | 2.19 | 5.16E-03 |
| 10 | GO cellular component | GO:0005739 | mitochondrion | MAVS, ACOX1, GPAT2, NDUFS7, NDUFS5, NDUFS4, SLC25A25, NUDT9, GPX4, HARS2, SLC25A46, MRPL54, SLC25A29, SLC25A44, ATP8, BDH1, ND6, ATP6, TOMM34, NDUFA4, AK1, SUCLG1, ND3, C21ORF33, NDUFC2, MRPS5, NDUFA13, BAD, SIRT7, DLAT, COQ7, SARS2, SDHA, TST, POLRMT, ND4L, PTRF, COX3, UQCRH, COX2, COX1, TOMM40L, MRPL48 | 41 | 2.03 | 6.94E-03 |
| 10 | GO cellular component | GO:0044455 | mitochondrial membrane part | NDUFA4, ND3, NDUFC2, NDUFA13, SDHA, NDUFS7, NDUFS5, ND4L, NDUFS4, COX3, UQCRH, ND6, ATP8, ATP6 | 12 | 5.16 | 7.00E-03 |
| 11 | GO cellular component | GO:0031974 | membrane-enclosed lumen | TGOLN2, SURF6, ARNT2, IDE, IARS2, DDX11, LRRC59, PQBP1, MRPL37, H2AFX, PDHA1, CFD, SUPT5H, GNL2, LUC7L3, MRPL52, MRPL51, STK24, HNRNPA2B1, ELL2, RCL1, MAPK1, MRPS18A, RARS, MED17, MDH2, UBA52, SIVA1, TXN2, CHEK1, UBAC1, PIN4, TIMP1, ACD, HNRNPK, RPL9, HNRNPF, CACYBP, NUP50, HOXA10, NPM3, FBXO5, GCDH, P4HB, RARS2, SRA1, AK3, CDC23, SMAD3, RPS6, HNRNPA1, FOXP1, NOLC1, PLK1, PSMC1, TDG, ALDH2, POP1, DDX54, ADAR, ATP5D, COPS5, TSG101, GNL3L, RPS2, DAXX, SAP30, TMEM109, GPKOW, NARF, CASP7, ZNF146, MSN, MYB, TOP2B, SARDH, PSMD8, SP100, LYN, POLR1D, CCNH, NOL7, RING1, TTF1, RB1, CDK7, MBD3, ECSIT, RBBP7, ZCCHC17, PRPF4, CCNL2, MED8, PMPCA, OAT, CLN5, ARL4A, C1D, MIDN, POLR2J, ETHE1, TIMM10, POLA1, NFS1, WBP11, NFYB, OXCT1, WDR13, GATAD2A, WIPF1, GEMIN7, NSA2, SSRP1, POLR3H, NF2, PSAP, CEBPG, CS, YWHAB, LMNA, LARS2, MRPL23, RPS6KA3, DUSP3, HSP90B1, SDF2L1, ANXA11, TCEB2, SH3D19, NCLN | 130 | 1.84 | 5.37E-10 |
| 11 | GO cellular component | GO:0070013 | intracellular organelle lumen | TGOLN2, SURF6, ARNT2, IDE, IARS2, DDX11, LRRC59, PQBP1, MRPL37, H2AFX, PDHA1, SUPT5H, GNL2, LUC7L3, MRPL52, MRPL51, STK24, HNRNPA2B1, ELL2, RCL1, MAPK1, MRPS18A, MED17, RARS, MDH2, UBA52, SIVA1, TXN2, CHEK1, UBAC1, PIN4, ACD, HNRNPK, RPL9, HNRNPF, NUP50, HOXA10, NPM3, FBXO5, GCDH, P4HB, RARS2, SRA1, AK3, CDC23, SMAD3, RPS6, HNRNPA1, FOXP1, NOLC1, PLK1, PSMC1, POP1, ALDH2, TDG, DDX54, ADAR, ATP5D, COPS5, TSG101, GNL3L, RPS2, DAXX, SAP30, TMEM109, GPKOW, NARF, CASP7, ZNF146, MSN, MYB, TOP2B, SARDH, PSMD8, SP100, POLR1D, CCNH, NOL7, RING1, TTF1, RB1, CDK7, MBD3, ECSIT, RBBP7, ZCCHC17, PRPF4, CCNL2, MED8, PMPCA, OAT, CLN5, ARL4A, C1D, MIDN, POLR2J, ETHE1, POLA1, NFS1, WBP11, NFYB, OXCT1, WDR13, GATAD2A, WIPF1, GEMIN7, NSA2, SSRP1, POLR3H, NF2, PSAP, CEBPG, CS, YWHAB, LMNA, LARS2, MRPL23, RPS6KA3, DUSP3, HSP90B1, SDF2L1, ANXA11, TCEB2, SH3D19, NCLN | 125 | 1.84 | 1.48E-09 |
| 11 | GO cellular component | GO:0043233 | organelle lumen | TGOLN2, SURF6, ARNT2, IDE, IARS2, DDX11, LRRC59, PQBP1, MRPL37, H2AFX, PDHA1, CFD, SUPT5H, GNL2, LUC7L3, MRPL52, MRPL51, STK24, HNRNPA2B1, ELL2, RCL1, MAPK1, MRPS18A, MED17, RARS, MDH2, UBA52, SIVA1, TXN2, CHEK1, UBAC1, PIN4, TIMP1, ACD, HNRNPK, RPL9, HNRNPF, NUP50, HOXA10, NPM3, FBXO5, GCDH, P4HB, RARS2, SRA1, AK3, CDC23, SMAD3, RPS6, HNRNPA1, FOXP1, NOLC1, PLK1, PSMC1, TDG, POP1, ALDH2, DDX54, ADAR, ATP5D, COPS5, TSG101, GNL3L, RPS2, DAXX, SAP30, TMEM109, GPKOW, NARF, CASP7, ZNF146, MSN, MYB, TOP2B, SARDH, PSMD8, SP100, POLR1D, CCNH, NOL7, RING1, TTF1, RB1, CDK7, MBD3, ECSIT, RBBP7, ZCCHC17, PRPF4, CCNL2, MED8, PMPCA, OAT, CLN5, ARL4A, C1D, MIDN, POLR2J, ETHE1, POLA1, NFS1, WBP11, NFYB, OXCT1, WDR13, GATAD2A, WIPF1, GEMIN7, NSA2, SSRP1, POLR3H, NF2, PSAP, CEBPG, CS, YWHAB, LMNA, LARS2, MRPL23, RPS6KA3, DUSP3, HSP90B1, SDF2L1, ANXA11, TCEB2, SH3D19, NCLN | 127 | 1.83 | 1.48E-09 |
| 11 | GO cellular component | GO:0005739 | mitochondrion | HCCS, ATP5D, TSPO, TIMM17B, PRDX4, PRDX5, IARS2, C14ORF2, NDUFAF2, ACOT9, BAK1, AGPAT5, MTG1, CASP7, DNAJC11, LRRC59, NDUFS8, SLC25A29, ABCB10, MRPL37, YRDC, SLC25A1, PDHA1, ATP5I, SARDH, MRPL33, PRKCA, MRPL52, MRPL51, NDUFB11, GTPBP3, LYN, AIFM2, SLC25A6, ND3, PICK1, MTPAP, CDK7, ECSIT, BCL2L11, MRPS18A, DSP, PEBP1, SLC25A39, AKAP1, OAT, PMPCA, MDH2, SIVA1, BID, HSD17B10, APEX2, TXN2, ETHE1, TIMM10, NFS1, HSPA1A, RSAD1, PTPMT1, PIN4, VARS, MTIF3, GLRX2, FIS1, TOMM5, C12ORF10, GFM1, NUDT8, OXCT1, PTS, GCDH, ATP5J2, RARS2, PSAP, CS, AK3, C21ORF33, NDFIP2, LARS2, MACROD1, MRPL23, MRPL22, MRPL21, FYN, HEBP2, ARAF, ALDH2, FPGS, PERP | 89 | 2.15 | 2.13E-09 |
| 11 | GO cellular component | GO:0005829 | cytosol | CEP72, EIF5, IDE, STAT5B, CASK, PTTG2, CCT3, BAK1, CUL5, GOT1, SCLY, EIF1AX, MLST8, MCM3AP, PDXK, STK24, BCL2L11, JUP, MAPK1, UXT, RPS16, RFK, RARS, HARS, USO1, SRXN1, UBA52, ARFGAP3, RPS26, TPI1, TUBB, PSMB1, RPL9, EIF3F, FBXO5, NDRG2, NPLOC4, ACY1, ABR, PCNT, SMAD3, CDC23, TKT, RPS6, CCT7, URM1, PLK1, PSMC1, FPGS, PRDX5, RPS2, PEX7, OFD1, NDE1, AGPAT5, COPB1, CASP7, ACTR1A, EEF2K, ZFAT, CDK5RAP2, TOP2B, AGPAT3, PSMD8, PRKCA, HSP90AA1, AIFM2, LYN, NUP88, PIK3C2A, RPS4X, PRKCD, ZCCHC17, PFDN1, OTOF, TOM1, BUB1B, PTMS, BID, HAUS2, NFS1, VARS, WDYHV1, GPSM1, GEMIN7, MLLT4, TCP1, YWHAB, TAB2, CENPJ, ICK, HSP90B1, CSNK1E, FYN, RPL21, ARAF, TCEB2, SH3D19, FAF1 | 99 | 1.95 | 2.55E-08 |
| 11 | GO cellular component | GO:0031981 | nuclear lumen | TGOLN2, SURF6, ARNT2, DDX11, PQBP1, H2AFX, GNL2, SUPT5H, LUC7L3, STK24, HNRNPA2B1, ELL2, RCL1, MAPK1, RARS, MED17, UBA52, SIVA1, TXN2, CHEK1, UBAC1, PIN4, HNRNPK, ACD, RPL9, HNRNPF, NUP50, HOXA10, NPM3, FBXO5, SRA1, CDC23, SMAD3, RPS6, HNRNPA1, FOXP1, NOLC1, PLK1, PSMC1, TDG, POP1, DDX54, ADAR, COPS5, TSG101, GNL3L, RPS2, DAXX, SAP30, TMEM109, GPKOW, CASP7, NARF, ZNF146, MSN, TOP2B, MYB, PSMD8, SP100, CCNH, POLR1D, NOL7, RING1, TTF1, RB1, MBD3, CDK7, ECSIT, RBBP7, PRPF4, ZCCHC17, CCNL2, MED8, C1D, ARL4A, MIDN, POLR2J, POLA1, NFYB, WBP11, WDR13, GATAD2A, WIPF1, GEMIN7, NSA2, SSRP1, POLR3H, NF2, CEBPG, LMNA, YWHAB, DUSP3, RPS6KA3, ANXA11, TCEB2, SH3D19, NCLN | 97 | 1.76 | 1.17E-05 |
| 11 | GO cellular component | GO:0044429 | mitochondrial part | ATP5D, HCCS, TSPO, TIMM17B, IARS2, BAK1, DNAJC11, CASP7, NDUFS8, LRRC59, SLC25A29, MRPL37, ABCB10, SLC25A1, PDHA1, ATP5I, SARDH, MRPL52, MRPL51, NDUFB11, AIFM2, LYN, SLC25A6, ND3, BCL2L11, MRPS18A, PEBP1, SLC25A39, AKAP1, PMPCA, OAT, MDH2, BID, HSD17B10, APEX2, ETHE1, NFS1, TIMM10, PTPMT1, PIN4, FIS1, TOMM5, OXCT1, GCDH, ATP5J2, RARS2, CS, AK3, LARS2, MRPL23, FYN, ALDH2 | 52 | 2.29 | 1.78E-05 |
| 11 | GO cellular component | GO:0031967 | organelle envelope | ATP5D, HCCS, TSPO, TIMM17B, LEMD2, RANGAP1, BAK1, TMEM109, DNAJC11, CASP7, NARF, NDUFS8, SLC25A29, ABCB10, SLC25A1, ATP5I, LBR, NDUFB11, AIFM2, LYN, NUP88, SLC25A6, ND3, NUP85, BCL2L11, AAAS, PEBP1, SLC25A39, AKAP1, PMPCA, MDH2, BID, HSD17B10, APEX2, POLA1, TIMM10, PTPMT1, FIS1, CSE1L, TOMM5, SHISA5, NUP50, CACYBP, FAM156A, GCDH, ATP5J2, LMNA, SMAD3, TREX1, FYN, ANXA11, FAF1 | 52 | 2.20 | 6.58E-05 |
| 11 | GO cellular component | GO:0031975 | envelope | ATP5D, HCCS, TSPO, TIMM17B, LEMD2, RANGAP1, BAK1, TMEM109, DNAJC11, CASP7, NARF, NDUFS8, SLC25A29, ABCB10, SLC25A1, ATP5I, LBR, NDUFB11, AIFM2, LYN, NUP88, SLC25A6, ND3, NUP85, BCL2L11, AAAS, PEBP1, SLC25A39, AKAP1, PMPCA, MDH2, BID, HSD17B10, APEX2, POLA1, TIMM10, PTPMT1, FIS1, CSE1L, TOMM5, SHISA5, NUP50, CACYBP, FAM156A, GCDH, ATP5J2, LMNA, SMAD3, TREX1, FYN, ANXA11, FAF1 | 52 | 2.19 | 7.24E-05 |
| 11 | GO cellular component | GO:0005730 | nucleolus | TGOLN2, COPS5, TSG101, SURF6, GNL3L, RPS2, TMEM109, GPKOW, DDX11, ZNF146, PQBP1, MSN, GNL2, TOP2B, PSMD8, SP100, POLR1D, NOL7, HNRNPA2B1, RING1, TTF1, ZCCHC17, MAPK1, RCL1, RARS, ARL4A, C1D, MIDN, TXN2, POLA1, UBAC1, PIN4, HNRNPK, RPL9, HNRNPF, WDR13, GATAD2A, NPM3, WIPF1, NSA2, NF2, CEBPG, RPS6, HNRNPA1, FOXP1, NOLC1, PSMC1, POP1, SH3D19, DDX54, NCLN, ADAR | 52 | 1.96 | 2.25E-03 |
| 11 | GO cellular component | GO:0000792 | heterochromatin | TCP1, RING1, H2AFY, H2AFX, MBD3, TOP2B, DAXX, HELLS, SMARCA4, SUV39H2 | 10 | 6.40 | 7.97E-03 |
| 11 | GO cellular component | GO:0031090 | organelle membrane | GNPTG, HCCS, ATP5D, TSPO, RAB9A, TIMM17B, LEMD2, SLC35A2, BAK1, TMEM109, CASP7, COPB1, NARF, DNAJC11, NDUFS8, SLC25A29, PIGC, ABCB10, SLC25A1, RPN2, SEC24C, ATP5I, LBR, SGPL1, NDUFB11, LYN, AIFM2, SLC25A6, PICK1, ND3, RPH3AL, NUP85, BCL2L11, ATP6V1F, MAN2A2, USO1, PEBP1, SLC25A39, MGAT5, AKAP1, PMPCA, CLN5, MDH2, BID, HSD17B10, APEX2, SLC37A4, GPAA1, TIMM10, PTPMT1, FIS1, TOMM5, SHISA5, PEX16, TMED10, ABCD3, LFNG, GCDH, ATP5J2, NOMO3, LMNA, SMAD3, CD63, PORCN, HSP90B1, ATP2A2, SLC18A3, DPM2, GOSR2, SPCS2 | 70 | 1.68 | 8.26E-03 |
| 11 | GO cellular component | GO:0005759 | mitochondrial matrix | ATP5D, GCDH, MRPL52, MRPL51, RARS2, ETHE1, CS, AK3, NFS1, LARS2, IARS2, PIN4, MRPL23, MRPS18A, OXCT1, LRRC59, ALDH2, MRPL37, PDHA1, OAT, PMPCA, SARDH, MDH2 | 23 | 2.66 | 2.37E-02 |
| 11 | GO cellular component | GO:0031980 | mitochondrial lumen | ATP5D, GCDH, MRPL52, MRPL51, RARS2, ETHE1, CS, AK3, NFS1, LARS2, IARS2, PIN4, MRPL23, MRPS18A, OXCT1, LRRC59, ALDH2, MRPL37, PDHA1, OAT, PMPCA, SARDH, MDH2 | 23 | 2.66 | 2.37E-02 |
| 11 | GO cellular component | GO:0030529 | ribonucleoprotein complex | HSPA1A, PIN4, RPS2, RPS26, HNRNPK, DDX23, RPL9, HNRNPF, MRPL37, HNRNPC, SNRNP35, GEMIN7, MRPL33, NSA2, MRPL52, MRPL51, HNRNPA2B1, SRA1, BTBD6, RPS6, RPS4X, SF3A2, HNRNPA1, PRPF4, ZCCHC17, HNRNPA0, DDX6, TARBP2, LARP6, MRPL23, MRPL22, MRPL21, RPS16, MRPS18A, RPL21, POP1, PUF60, UBA52, RBM17 | 39 | 1.99 | 3.02E-02 |
| 11 | GO cellular component | GO:0005654 | nucleoplasm | SURF6, ARNT2, RPS2, DAXX, SAP30, CASP7, H2AFX, TOP2B, SUPT5H, LUC7L3, SP100, CCNH, STK24, POLR1D, HNRNPA2B1, RING1, TTF1, RB1, MBD3, CDK7, RBBP7, ECSIT, PRPF4, CCNL2, ELL2, MAPK1, MED17, MED8, UBA52, C1D, SIVA1, POLR2J, POLA1, NFYB, WBP11, CHEK1, ACD, HNRNPK, HNRNPF, NUP50, GATAD2A, HOXA10, FBXO5, GEMIN7, SSRP1, POLR3H, SRA1, YWHAB, SMAD3, CDC23, HNRNPA1, DUSP3, RPS6KA3, PLK1, ANXA11, TDG, TCEB2 | 57 | 1.70 | 4.28E-02 |
| 11 | GO cellular component | GO:0031966 | mitochondrial membrane | ATP5D, BID, HCCS, HSD17B10, TSPO, APEX2, TIMM17B, TIMM10, PTPMT1, BAK1, FIS1, TOMM5, CASP7, DNAJC11, NDUFS8, SLC25A29, ABCB10, SLC25A1, ATP5I, GCDH, ATP5J2, NDUFB11, AIFM2, LYN, SLC25A6, ND3, BCL2L11, PEBP1, SLC25A39, AKAP1, PMPCA, MDH2 | 32 | 2.13 | 4.48E-02 |
| 13 | GO cellular component | GO:0000776 | kinetochore | CENPM, CASC5, SKA3, CENPE, NUP43, CBX5, DCTN2 | 7 | 11.17 | 8.26E-03 |
| 13 | GO cellular component | GO:0012505 | endomembrane system | SREBF1, SYS1, STX1A, HIP1R, LMNA, HLA-A, RER1, PIGS, GIPC1, NUPL2, PIGO, OS9, CBX5, BNIP2, BAX, DPM3, STX10, NUP43 | 18 | 2.83 | 3.65E-02 |
| 14 | GO cellular component | GO:0031967 | organelle envelope | MAVS, RTN4, FKBP8, SLC22A18, NDUFB9, COX7C, LEMD3, UQCRQ, HADHB, NDUFB2, TMEM173, UQCR10, TOMM7, RAC2, ETFDH, KPNB1, COX17, ATP5J, NDUFA5, MSTO1, NDUFA6, NDUFA7, SCRN1, NDUFC1, ITPR3, ACADVL, MFN1, NUP62, PTGDS, COX6A1, XPO7, GPAM, MATR3, MVP, SRGAP2 | 35 | 2.25 | 5.19E-03 |
| 14 | GO cellular component | GO:0044429 | mitochondrial part | MAVS, FKBP8, MRPS16, ACADSB, NDUFB9, COX7C, UQCRQ, HADHB, NDUFB2, TMEM173, UQCR10, TOMM7, PARS2, ETFDH, COX17, ATP5J, NDUFA5, MRPS28, MSTO1, ACADM, ACO2, NDUFA6, NDUFA7, MRPS21, NDUFC1, SIRT3, ACADVL, DBT, MFN1, COX6A1, CARS2, GPAM, MRPL43, SRGAP2 | 34 | 2.28 | 5.56E-03 |
| 14 | GO cellular component | GO:0031975 | envelope | MAVS, RTN4, FKBP8, SLC22A18, NDUFB9, COX7C, LEMD3, UQCRQ, HADHB, NDUFB2, TMEM173, UQCR10, TOMM7, RAC2, ETFDH, KPNB1, COX17, ATP5J, NDUFA5, MSTO1, NDUFA6, NDUFA7, SCRN1, NDUFC1, ITPR3, ACADVL, MFN1, NUP62, PTGDS, COX6A1, XPO7, GPAM, MATR3, MVP, SRGAP2 | 35 | 2.24 | 5.60E-03 |
| 14 | GO cellular component | GO:0070013 | intracellular organelle lumen | E2F1, NCBP1, E2F6, RBM5, PKMYT1, CDT1, INTS9, OIP5, GIT2, OGT, KPNB1, BRD8, CDK1, CTBP2, ACADM, BRF1, ACO2, DFFA, LIG1, PARP10, MED13, CERCAM, ACADVL, SLU7, WASL, NEK4, NHP2, MATR3, MRPL43, SNRPG, CPSF3L, MRPS16, ACADSB, POLR2E, UBE2Z, TFEB, ANAPC11, SERPINH1, HADHB, PRPF19, PARS2, REXO4, TFDP2, HIST1H4C, TINF2, RBM25, CHD3, TAF1, TBL3, MRPS28, TAF8, TAF7, MPHOSPH10, MRPS21, ITPR3, FOXP1, ZBTB43, SIRT3, MEF2D, DBT, HDAC3, ATXN3, NUP62, SAP130, SFPQ, RBM19, CALM3, CARS2, KDM4A, UTP20, ZNF768 | 71 | 1.59 | 2.14E-02 |
| 14 | GO cellular component | GO:0043233 | organelle lumen | E2F1, NCBP1, E2F6, PDGFA, RBM5, PKMYT1, CDT1, INTS9, OIP5, GIT2, OGT, KPNB1, BRD8, CDK1, CTBP2, BRF1, ACADM, ACO2, DFFA, LIG1, PARP10, MED13, CERCAM, ACADVL, SLU7, WASL, NEK4, NHP2, MATR3, MRPL43, SNRPG, CPSF3L, MRPS16, ACADSB, POLR2E, UBE2Z, TFEB, ANAPC11, SERPINH1, HADHB, PRPF19, PARS2, REXO4, TFDP2, HIST1H4C, TINF2, RBM25, CHD3, TAF1, TBL3, MRPS28, TAF8, TAF7, MPHOSPH10, MRPS21, ITPR3, FOXP1, ZBTB43, SIRT3, MEF2D, DBT, HDAC3, ATXN3, NUP62, SAP130, SFPQ, RBM19, CALM3, CARS2, KDM4A, UTP20, ZNF768 | 72 | 1.58 | 2.45E-02 |
| 14 | GO cellular component | GO:0031974 | membrane-enclosed lumen | E2F1, NCBP1, E2F6, PDGFA, RBM5, PKMYT1, CDT1, INTS9, OIP5, GIT2, OGT, COX17, KPNB1, BRD8, CDK1, CTBP2, BRF1, ACADM, ACO2, DFFA, LIG1, PARP10, MED13, CERCAM, ACADVL, SLU7, WASL, NEK4, NHP2, MATR3, MRPL43, SNRPG, CPSF3L, MRPS16, ACADSB, POLR2E, UBE2Z, TFEB, ANAPC11, SERPINH1, HADHB, PRPF19, PARS2, REXO4, TFDP2, HIST1H4C, TINF2, RBM25, CHD3, TAF1, TBL3, MRPS28, TAF8, TAF7, MPHOSPH10, MRPS21, ITPR3, FOXP1, ZBTB43, SIRT3, MEF2D, DBT, HDAC3, ATXN3, NUP62, SAP130, SFPQ, RBM19, CALM3, CARS2, KDM4A, UTP20, ZNF768 | 73 | 1.57 | 2.57E-02 |
| 15 | GO cellular component | GO:0005739 | mitochondrion | MRPS36, MRPS35, COX7B, NLRX1, PPOX, TIMM50, UQCRFS1, SDHAF1, GFM2, CISD1, MRPL14, NT5M, COL4A3BP, TRAK1, MRPL16, GSTZ1, MYCBP, GTF3C2, ETFB, AP2M1, ENOSF1, BCKDHA, TXNIP, ND1, BSG, PIGY, LIMK2, KIAA0141, MFN2, MRPS18C, TSFM, ATPIF1, MRPL45 | 33 | 2.26 | 5.23E-03 |
| 15 | GO cellular component | GO:0048475 | coated membrane | COPG2, AFTPH, SEC31A, SCYL1, AP2S1, NECAP2, GGA1, AP2M1 | 8 | 9.44 | 6.33E-03 |
| 15 | GO cellular component | GO:0030117 | membrane coat | COPG2, AFTPH, SEC31A, SCYL1, AP2S1, NECAP2, GGA1, AP2M1 | 8 | 9.44 | 6.33E-03 |
| 15 | GO cellular component | GO:0030529 | ribonucleoprotein complex | MRPS36, MRPS35, TBL3, RPLP2, RPL23A, PRPF18, NAA38, TTF2, MRPS18C, RPL23, MRPL14, RPS14, MRPL16, SNRNP200, SNRPC, RNPC3, MRPL45, RPS27A, TERT, ZCCHC8 | 20 | 2.89 | 1.88E-02 |
| 15 | GO cellular component | GO:0030120 | vesicle coat | COPG2, AFTPH, SEC31A, SCYL1, AP2S1, NECAP2 | 6 | 11.73 | 4.41E-02 |
